# Biginelli dihydropyrimidines: a tunable class of alkyl radical precursors

**DOI:** 10.1039/d6sc00376a

**Published:** 2026-03-13

**Authors:** Shahilan Ratnam, Shreya Unone, Nabeel Alia, Enyu Denny Hafeneger, Daniel Janssen-Müller

**Affiliations:** a Institut für Organische und Biomolekulare Chemie, Georg-August-Universität Göttingen Tammannstraße 2 37077 Göttingen Germany djansse@uni-goettingen.de

## Abstract

Alkyl-substituted dihydropyrimidines (DHPyms), synthesised *via* the Biginelli reaction, are introduced as tunable alternatives to 4-alkyl Hantzsch dihydropyridines (DHPs) in radical chemistry. Leveraging the modularity of the Biginelli reaction, we systematically explored the redox properties, UV/vis absorption, and synthetic potential of DHPyms in radical-mediated transformations, identifying dimethylamino-substituted DHPym as a highly reactive, bench-stable, and easily synthesised alkyl radical precursor.

## Abstract 2

Dihydropyrimidines (DHPyms) are introduced as bench-stable and tunable alkyl radical precursors. Derived through the Biginelli reaction, DHPyms are stronger excited state reductants than well-known Hantzsch dihydropyridines (DHPs).

## Graphical abstract

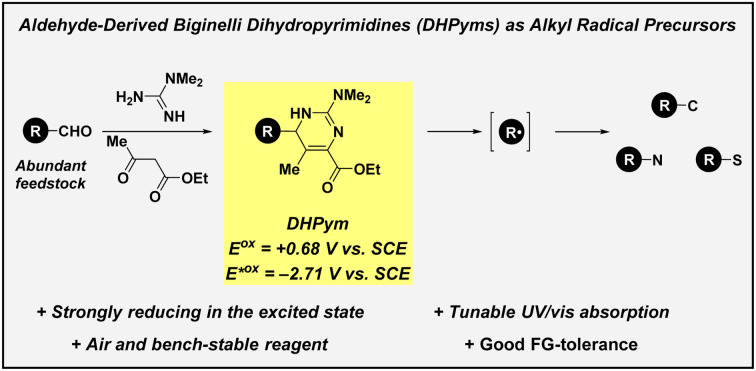

## Introduction

The development and use of organic precursors for the formation of alkyl radicals has emerged as a prominent area of research in organic synthesis. Notably, easily oxidizable redox-active heterocycles^1^ such as 4-alkyl Hantzsch esters, derived from aldehydes *via* the Hantzsch dihydropyridine (DHP) synthesis,^2^ have garnered significant attention due to their utility as precursors for radical-mediated transformations.^3^ These Hantzsch DHPs have enabled the utilization of aldehydes, a fundamental functional group present in a variety of synthetic and natural compounds, as substrates for alkyl radical formation. Radical formation strategies from these Hantzsch esters include thermal processes using peroxide reagents^4^ or photochemical activation through photoredox catalysis,^5^ sometimes in combination with transition-metal catalysis^6^ or organocatalysis.^7^ Moreover, photochemical activation without the need for a photocatalyst has been reported for Hantzsch DHPs.^8^

**Scheme 1:**
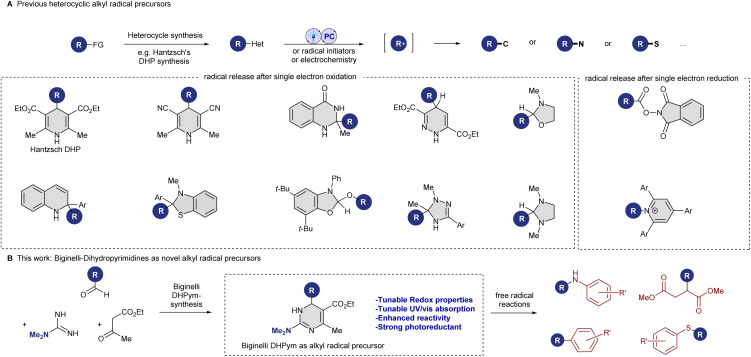
(A) Previous N-heterocyclic redox-active radical precursors. (B) Biginelli-dihydropyrimidines as alkyl radical precursors.

Chemists have recently also investigated different heterocyclic alkyl radical precursors. These alternative redox-active heterocycles include dihydroquinazolinones,^9^ triazolines,^10^ and benzothiazolines,^11^ derived from ketones,^12^ oxazolidines and imidazolidines derived from aldehydes,^13^ dihydropyridazines from alkenes,^14^ dihydroquinolines derived from organometallic reagents,^15^ and benzoxazolines derived from alcohols (see Scheme 1A).^16^ A common feature among these auxiliaries is the occurrence of aromatization or resonance as the driving force for the release of the alkyl radical. Mechanistically, these oxidizable auxiliaries all operate through a similar pathway: single-electron oxidation or hydrogen-atom abstraction of the pro-aromatic heterocycle leads to a high-energy intermediate, which induces homolytic C–C bond cleavage, releasing an alkyl radical and often an aromatic heterocycle.

Motivated by the success of existing heterocyclic auxiliaries and the desire to expand the synthetic toolbox, we sought to investigate the potential of dihydropyrimidines (DHPyms) as novel substitutes for DHPs. DHPyms, readily accessible *via* the Hideg modification^17^ of the Biginelli reaction,^18^ present an intriguing opportunity to broaden the scope of heterocyclic redox auxiliaries and introduce novel variation and tuning possibilities. Inspired by the modularity of the Biginelli reaction (see Scheme 1B), which allows for facile access to structurally diverse compounds, we hypothesized that DHPyms could also act as reducing alkyl radical precursors and offer enhanced reactivity and synthetic versatility, addressing current limitations in radical chemistry. DHPyms have been described in the literature as readily oxidized to aromatic pyrimidines under various conditions, and they usually exist in solution as a mixture of 1,4-dihydropyrimidine and 3,4-dihydropyrimidine tautomers, whose ratio depends on solvent and substitution pattern.^19^ Kappe reported that by the choice of oxidant, an exocyclic C–C bond could be cleaved (MnO_2_, CAN, KMnO_4_) or retained (chloranil and DDQ) during aromatization,^19*a*^ but so far their use as alkyl radical precursor is not described in the literature.

## Results and discussion

**Scheme 2:**
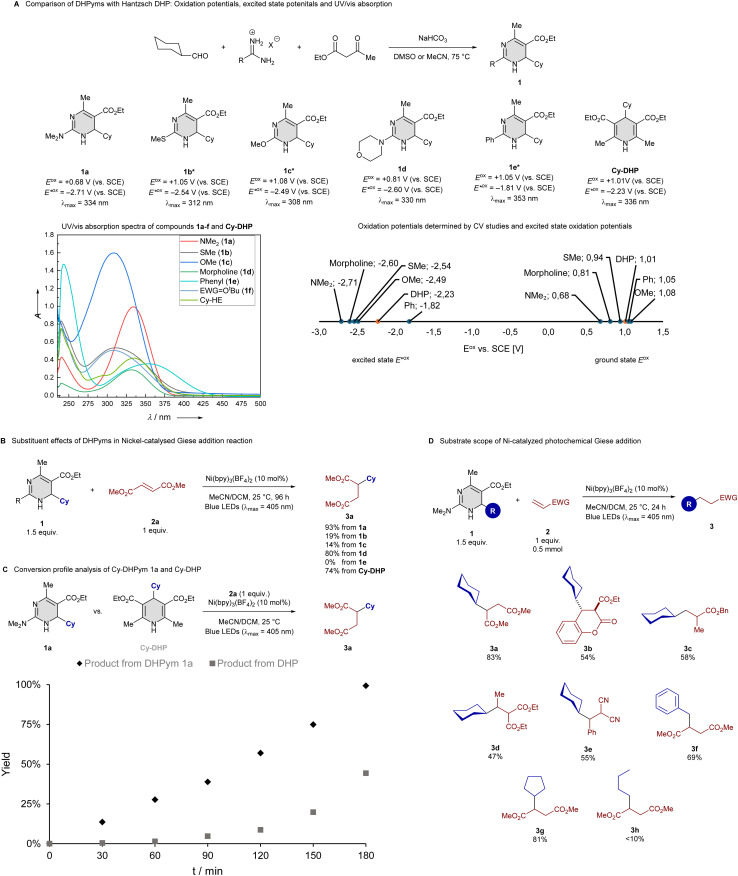
(A) Comparison of substituent effects of DHPyms with Hantzsch ester on the oxidation potential and UV/vis absorption spectra, determined by cyclic voltammetry and UV/vis spectroscopy. UV/vis recorded at 50 µM (1c) or 100 µM (others) concentration in CHCl_3_. Cyclic voltammetry recorded with *n*-Bu_4_NPF_6_ in MeCN under nitrogen at a scan rate of 100 mV s^−1^. *In solution, a tautomeric mixture of 1,4- and 3,4-dihydropyrimidine was observed. (B) Substituent effects in Giese addition. Yields were determined as GC yields with *n*-decane as internal standard. (C) Competition experiment between DHPym 1a and DHP. Yields were determined as GC yields with *n*-decane as internal standard. (D) Substrate scope of Giese addition. Isolated yields after column chromatography. Reaction conditions: alkene (0.5 mmol, 1.0 equiv.), 1 (0.75 mmol, 1.5 equiv.), Ni(bpy)_3_(BF_4_)_2_ (10 mol%), in degassed MeCN/DCM under argon for 24 h irradiated at 405 nm.

We started our investigation with the synthesis of some DHPyms, which was accomplished *via* the established Biginelli reaction, providing access to a diverse library with varying substitution patterns. We investigated cyclohexyl-substituted DHPyms 1a–e and compared them with cyclohexyl-substituted Hantzsch DHP by cyclic voltammetry experiments, which revealed a range of oxidation potentials (*E*^ox^) for DHPyms, spanning from 0.68 to 1.05 V, markedly lower than the typical *E*^ox^ range of DHPs (1.0–1.2 V), with amino-substituted DHPym 1a being the most reducing, probably *via* resonance stabilization of the radical cation through the electron-donating amino-substituent. This observation prompted further investigation into the synthetic utility of DHPyms as alkyl radical precursors and raised the question about their excited state oxidation potential, since DHPs are often used as photoreductants with the capability to generate an alkyl radical. This combination of photoreductant and radical precursor reactivity led to a variety of interesting combinations of photochemistry and low-valent metal catalysis. We therefore measured fluorescence spectra and used the Rehm–Weller equation^20^ for determining excited state potentials, which revealed that DHPym reagent 1a had an excited state oxidation potential 
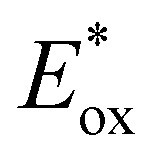
 of −2.71 V, much lower than that of the common DHP reagent (see Scheme 2A). It should be noted that 
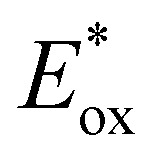
 values, calculated from maximum absorption peaks, are overestimated relative to actual photosynthesis conditions at 390 or 405 nm and should be used only for comparative redox assessment of 1a–e. To compare the radical transfer capabilities of DHPyms, we decided to employ them under Melchiorre's photocatalyst-free Giese addition reaction, developed for Hantzsch DHPs and which uses Ni(bpy)_3_(BF_4_)_2_ as an electron-mediator.^8*s*^ It turned out that amino-substituted DHPyms 1a and 1d were superior to the Hantzsch ester (see Scheme 2C). We then used DHPym 1a as our model reagent to study the conversion profile in comparison to the established DHP. We observed that our DHPym reagent 1a shows a strongly enhanced reactivity, most likely caused by its more negative excited state oxidation potential, which indicates high reducing power.

Motivated by this enhanced reactivity, we studied the substrate scope of the dimethylamino-substituted DHPym reagent in the Giese addition on 0.5 mmol scale with 24 h reaction time to ensure completion on the larger scale. We found that under Melchiorre's original conditions,^8*s*^ but using a weaker LED, a variety of electron-deficient alkenes could be employed as well as secondary and benzylic alkyl radicals derived from the DHPym. Slight losses of product 3a compared to the excellent yields form the kinetic study arose from chromatographic purification. Primary alkyl radicals were much less effective, giving less than 10% of hydroalkylation product. For the primary example 3h, a significant amount of aromatized alkyl-pyrimidine was observed together with unreacted DHPym reagent, suggesting that a competing hydrogen-transfer reactivity becomes dominant for primary alkyl-substituted DHPym, which might be controllable through additional fine-tuning of substituents. It has to be noted however that the generation of primary radicals from DHP reagents is a common limitation in a variety of protocols. After having studied the Giese addition with a small selection of examples, we aimed to employ our reagent in some synthetically relevant radical transformation.

Motivated by reports on the Ni-catalysed cross-coupling with alkyl-DHP reagents,^6*a*,*b*^ we discovered that our alkyl-DHPym reagents are also acting as competent coupling partners in a Ni-catalysed cross-coupling.

**Scheme 3:**
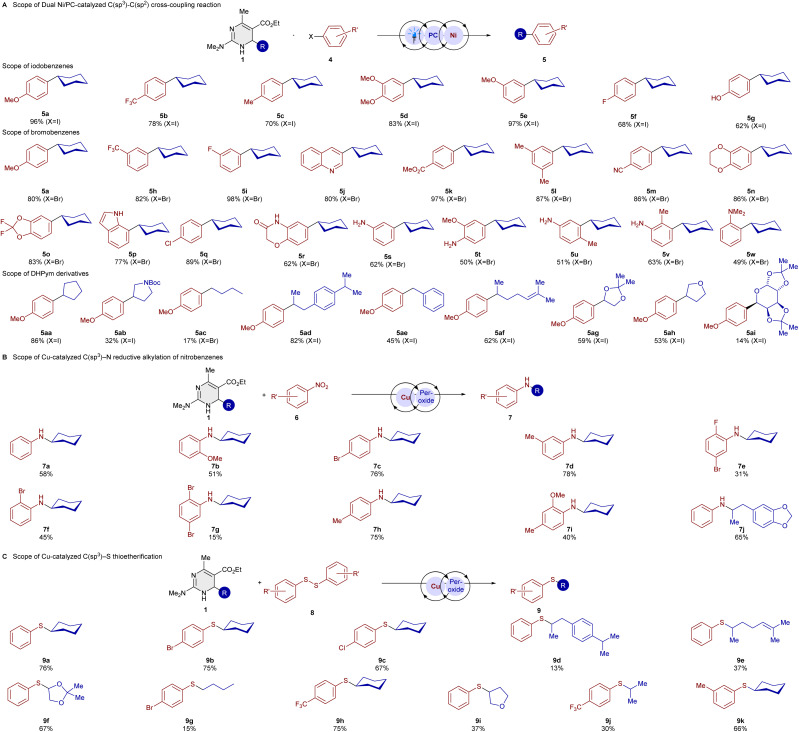
Substrate scope of DHPym-derived alkyl radicals. Isolated yields after column chromatography. Reaction conditions: (A) aryl iodide (0.5 mmol, 1.0 equiv.), 1 (1 mmol, 2.0 equiv. for X = I; 1.5 mmol, 3.0 equiv. for X = Br), 4CzIPN (1 mol%), NiCl_2_dme (10 mol%), dtbbpy (15 mol%), pyridine base (DMAP for X = I, 2,6-lutidine for X = Br) (1.5 equiv.) in degassed MeCN under argon for 24 h irradiated at 450 nm. Reaction conditions for unprotected amines: bromoaniline (0.5 mmol, 1.0 equiv.), 1 (1.5 mmol, 3.0 equiv.), *fac*-Ir(ppy)_3_ (1 mol%), NiCl_2_dme (20 mol%), dtbbpy (30 mol%), DMAP (1.5 equiv.) in degassed MeCN under argon for 24 h irradiated at 450 nm. (B) 1 (0.6 mmol, 2.0 equiv.), nitrobenzene derivative (0.2 mmol, 1.0 equiv.), Cu(TC) (10 mol%), dtbbpy (10 mol%), dicumyl peroxide (DCP) (1.5 mmol, 3 equiv.) in 1,4-dioxane (2.0 mL) under argon at 130 °C for 12 h. (C) 1 (0.2 mmol, 1.0 equiv.), disulfide (0.2 mmol, 1.0 equiv.), CuCl (10 mol%), TBHP (1.0 equiv.) in MeCN at 50 °C for 20 h.

After reaction optimization (see SI for information), numerous examples of both bromobenzenes and iodobenzenes were tolerated (see Scheme 3A), bearing common functional groups such as ether (5d and 5e), ester (5k), cyano (5m), F (5f and 5i), Cl (5q) or CF_3_ (5b and 5h). Also for this alkyl radical reaction, an increased rate when compared to the DHP was observed (see SI for information). The cross-coupling with heterocyclic aryl halides such as dioxanes, dioxolanes, indoles, and quinolines gave very good yields. It is worth mentioning that our developed cross-coupling method of aryl iodides using DHPyms works in various solvents with a broad polarity range, from MeOH to PhMe, with very good yields, which can be beneficial for substrates for which solubility in acetonitrile bears a limitation (for further details, see the SI).

A major limitation of previously known methods is the cross-coupling of haloarenes possessing amines, which we could overcome with our method. Since our DHPym 1a has a much lower oxidation potential compared to the Hantzsch ester, we found that under Ni/Ir dual photocatalysis, the cross-coupling of unprotected bromoanilines was performed with a good yield.

Since our DHPym reagents are excellent reductants compared to Hantzsch DHPs, we wondered if they could be applied to the reductive alkylation of nitrobenzene derivatives, which was reported previously for DHPs as radical precursors.^4*m*^ Our more reducing DHPym reagent turned out to be a competent alkylative reductant in this transformation (Scheme 3B). DHPym 1a with NMe_2_ and 1b with SMe-substitution had the same yield for aniline 7a in the reductive alkylation with 58%, whereas the OMe-DHPym 1c had a decreased yield with 39%. With dimethylamino-substituted DHPym we went on to study the substrate scope of this transformation. Good to very good yields could be obtained with 7c, 7d, and 7h, which did not show a clear tendency regarding electronic effects. Disubstitution with *ortho*–*para*- and *ortho*–*meta*-patterns were tolerated as well with good yields. Comparing products 7f and 7g, the disubstituted product had a decreased yield. The helional-derived DHPym reagent resulted in a good yield for 7j. We also became interested in a C–S bond-forming reaction with our DHPym reagents. C–S bond formation with Hantzsch esters is usually achieved with activated sulfur electrophiles such as TsSPh.^5*t*^

In our previous study on dihydroquinolines as alkyl radical precursors,^15^ we achieved a thiolation reaction with less reactive aryl disulfides under copper catalysis. With the even more reducing DHPym reagent 1a, we could achieve much higher yields of thiolation product 9a under similar reaction conditions as for our dihydroquinoline reagent (see Scheme 3C).

When studying the reaction scope of this transformation, we could demonstrate that multiple DHPym precursors to secondary alkyl radical can be utilized with moderate to good yields. A primary radical could be transferred (9g), even though a low yield was obtained.

**Scheme 4:**
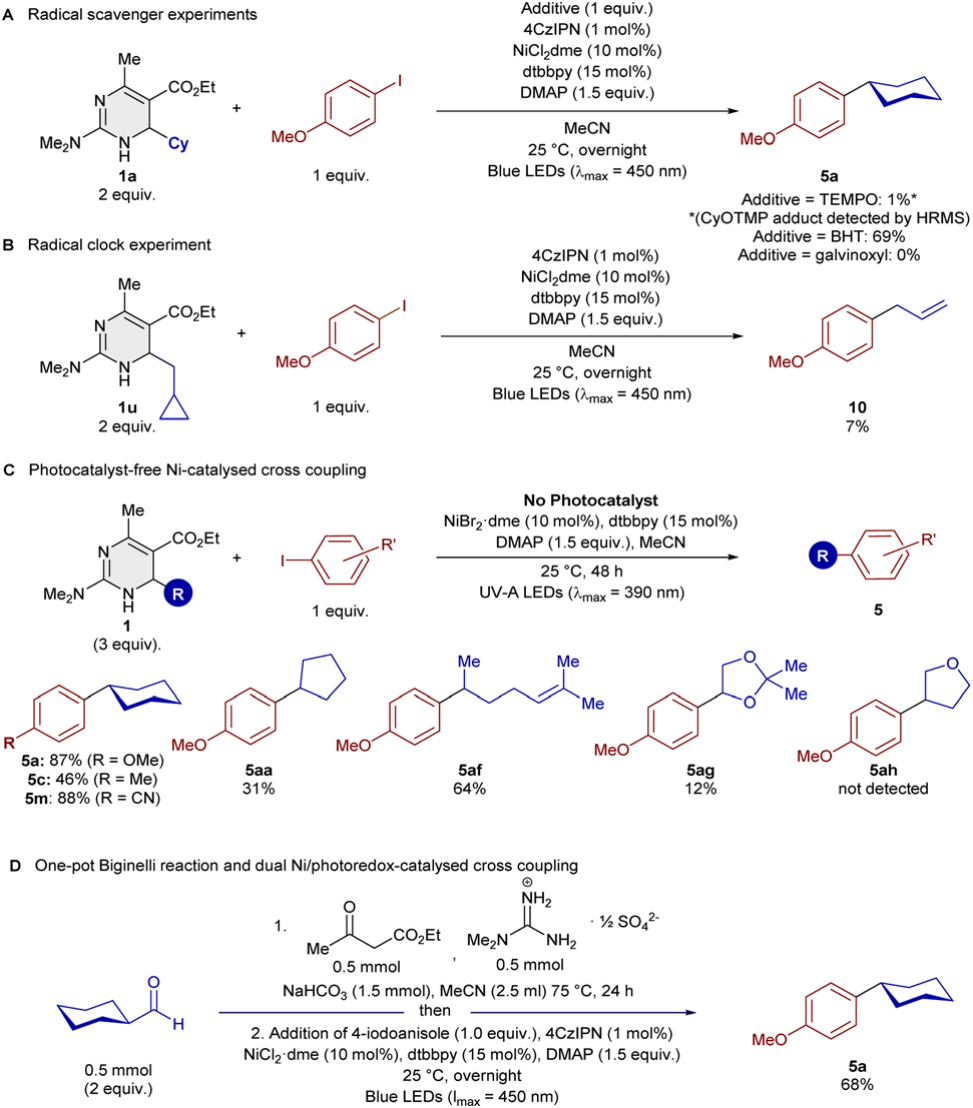
(A) Radical scavenger experiments. (B) Radical clock experiment. (C) Photocatalyst-free Ni-catalysed cross-coupling. (D) One-pot Biginelli cross-coupling reaction.

To validate the formation of an alkyl radical, we added radical scavengers to the Ni-catalysed cross-coupling and we observed that TEMPO and galvinoxyl led to complete shutdown of the reactivity, while BHT led to a slightly reduced yield of 69% (see Scheme 4A). In the case of TEMPO as additive, the expected cyclohexyl TEMPO adduct was formed as evidenced by GC-MS and ESI-HRMS. A radical clock experiment with cyclohexylmethyl-substituted DHPym 1u led to ring-opened cross-coupling product 10, further validating the formation of alkyl radicals (Scheme 4B).

A separate optimization of the Ni-catalyzed cross-coupling reaction, but without photocatalyst, led to reaction conditions with UV-A irradiation (390 nm), in which an excellent yield of 87% of cross-coupling product 5a was isolated (see Scheme 4C). A small scope study revealed some degree of generality, but addition of the 4CzIPN photocatalyst leads to higher yields whenever compared directly with the photocatalyst-free conditions.

We also attempted a one-pot approach successfully to the cross-coupling of DHPyms, in which cyclohexanecarboxaldehyde was first converted into the DHPym 1a*via* Biginelli reaction in MeCN before addition of the remaining starting materials for the cross-coupling in the same solvent and flask. Just a simple degassing step was performed after addition of the starting materials for the second step. This approach greatly improves the practicality of the process since the isolation of DHPyms after Biginelli reaction usually requires column chromatography.

## Conclusions

In conclusion, our study introduces alkyl DHPyms as novel heterocyclic redox auxiliaries for alkyl radical transfer reactions, which are easily synthesised from aldehyde precursors and are bench-stable solids. The modularity of the Biginelli reaction enables facile access to structurally diverse DHPyms with tunable redox properties, offering exciting opportunities for innovation in radical-mediated transformations. Notably, the cross-coupling with these more-reducing electroauxiliaries turned out to tolerate free unprotected amines as substrates and shows a high degree of generality. The tunable nature of these Biginelli-derived DHPyms, combined with their superior reactivity compared to traditional Hantzsch esters, positions them as versatile tools for radical-mediated transformations under mild conditions. Beyond the demonstrated applications, we envision their utility in late-stage functionalization of complex molecules, such as pharmaceuticals, natural product derivatives, or peptides, where precise alkyl radical delivery enables diversification without disrupting sensitive functional groups. Furthermore, the straightforward one-step synthesis from abundant aldehydes opens avenues for incorporation into total synthesis routes.

## Author contributions

S. R., S. U., N. A., E. D. H. and D. J.-M. performed experiments. S. R., S. U. and D. J.-M. conducted mechanistic experiments. D. J.-M. conceived the project, supervised the research and wrote the manuscript with contributions from all authors.

## Conflicts of interest

There are no conflicts to declare.

## Supplementary Material

SC-OLF-D6SC00376A-s001

SC-OLF-D6SC00376A-s002

## Data Availability

CCDC 2338773–2338775 contain the supplementary crystallographic data for this paper.^21*a*–*c*^ The data that support the findings of this study are available in the supplementary information (SI) of this article. Supplementary information: experimental procedures, characterization data, and NMR spectra. See DOI: https://doi.org/10.1039/d6sc00376a.
