# Supplementary material for: Biginelli dihydropyrimidines: a tunable class of alkyl radical precursors
Source: Chem Sci. 2026 Mar 13;17(18):8998–9005. doi: 10.1039/d6sc00376a (PMC13000759; doi:10.1039/d6sc00376a)
Supplement: SC-017-D6SC00376A-s001 [file SC-017-D6SC00376A-s001.pdf]

Supplementary Information (SI) for Chemical Science.
This journal is © The Royal Society of Chemistry 2026

Supporting Information

Biginelli Dihydropyrimidines: A Tunable Class of Alkyl Radical Precursors

Shahilan Ratnam, Shreya Unone, Nabeel Alia, Enyu Denny Hafeneger and Daniel Janssen-Müller*

Institut für Organische und Biomolekulare Chemie, Georg-August-Universität Göttingen, Tammannstraße 2, 37077 Göttingen, Germany. *E-mail: djansse@uni-goettingen.de

Table of Contents

General Remarks 2

Synthesis of dihydropyrimidine derivatives General procedure 1a (GP 1a) 4

Ni-catalyzed Giese addition: General procedure 2 (GP 2) 19

Optimization of Ni/photoredox dual catalyzed alkylation of haloarenes 24

Optimization of photocatalyst-free Ni/photoredox dual catalyzed alkylation of haloarenes 30

Dual Ni/PC-catalyzed coupling with iodobenzenes: General procedure 3a (GP 3a) 40

Dual Ni/PC-catalyzed coupling with Bromobenzenes: General procedure 3b (GP 3b) 40

Dual Ni/PC-catalyzed coupling with bromoanilines: General procedure 3c (GP 3c) 41

Photocatalyst-free Ni-catalyzed coupling with iodobenzenes: General procedure 3d (GP 3d) 41

Reductive alkylation of nitroarenes: General procedure 4 (GP 4) 61

Cu-catalyzed alkylation of disulfides: General procedure 5 (GP 5) 68

Mechanistic investigations 74

Conversion profile measurements 77

Pyrimidine byproduct 83

UV-vis spectroscopy 84

Luminescence emission spectroscopy 89

Cyclic voltammetry 92

Excited state oxidation potential 96

Luminescence quenching 101

Proposed mechanisms 103

Crystallographic Data 106

Spectral data 112

References 197

General Remarks

Catalytic reactions were performed under an argon atmosphere using pre-dried glassware and standard Schlenk techniques. For reactions with a total volume of less than 8 mL, Schlenk tubes with screw caps, PTFE seals and separate PTFE corner valves were used. Chemicals for dihydropyrimidine synthesis and coupling reactions, catalysts and solvents were obtained from commercial sources and were used without further purification, if not otherwise stated. 4CzIPN was synthesized according to a reported procedure.[1] $Ni(bpy)_3(BF_4)_2$ was synthesized according to a reported procedure.[2] Dry solvents were obtained from a solvent purification system (MBraun SPS-800) and dried with activated molecular sieves.

Analytical thin layer chromatography (TLC) was performed on silica gel 60 F254 aluminum plates (Merck) or POLYGRAM® SIL G/UV254 (MACHEREY-NAGEL). TLC plates were visualized by exposure to short wave ultraviolet light (254 nm, 366 nm) or were dipped into a solution of potassium permanganate or ninhydrin stain solution.

Flash Column chromatography was performed with silica gel Merck 60 (35-70 µm), using a Biotage Isolera One and MACHEREY-NAGEL CHROMABOND Flash BT cartridges.

NMR spectra were recorded on a Bruker Avance III HD 300 or Bruker Avance Neo 400 in the solvent indicated at ambient temperature, if not otherwise stated. Chemical shifts (δ) are provided in ppm.

High resolution mass spectrometry (HR-MS) was recorded on Bruker microTOF and maXis instrument.

IR spectra were recorded on a Bruker FT-IR alpha-P device. The wave numbers ($\tilde{\nu}$) of recorded IR-signals are quoted in cm^{-1}.

UV-vis spectra were recorded on a V-770 UV-VIS-NIR spectrophotometer in chloroform or DMSO.

Fluorescence emission spectra were recorded at the absorption maximum λ_{max} on a JASCO FP-8500 spectrofluorometer operated using the software Spectra Manager™. All measurements were conducted in MeCN or $CHCl_3$.

Cyclic voltammetry measurements were conducted with a Metrohm Autolab PGSTAT204 potentiostat and Nova 2.1 software. For all experiments, a glassy carbon working electrode (disk, diameter: 3 mm), a platinum wire counter electrode and a SCE reference electrode were used. *n*-Bu_4NPF_6 (100 mM) as conducting salt served as electrolyte for the measurements. The voltammograms were recorded under inert atmosphere in acetonitrile at a scan rate of

100 mV/s. The samples were measured in acetonitrile with an analyte concentration of 10 mM and degassed with nitrogen prior to measurement.

Gas chromatography (GC) was performed on an Agilent 7890A GC System or Agilent 7890B GC System equipped with an Agilent HP-5MS column (30 m × 0.25 mm diameter, 0.25 μm film thickness) and a flame-ionization detector (FID) using hydrogen as the carrier gas.

Gas chromatography coupled with mass-spectrometry (GC-MS) was performed on the same instrument coupled with Agilent 5875C Triple-Axis-Detector or Agilent 5977B MSD. Mass spectra were obtained with electron-ionization (EI) at 70 eV in positive ion mode. Temperature program: injection temperature 40 °C, hold for 1 min, rate 30 °C/min up to 120 °C, hold for 1 min, 35 °C/min up to 250 °C, hold for 2 min, 50 °C up to 290 °C, hold for 6 min.

Photochemical reactions were performed in the commercially purchased photoreactor HepatoChem EvoluChem™ PhotoRedOx Box Duo™ with the LEDs EvoluChem™ 390PF (18 W, λ_{max} = 390 nm), EvoluChem™ 405PF (18 W, λ_{max} = 405 nm) or EvoluChem™ 450PF (18 W, λ_{max} = 450 nm). The reactions were stirred at 600 min^{-1} and air-cooled by the integrated fans.

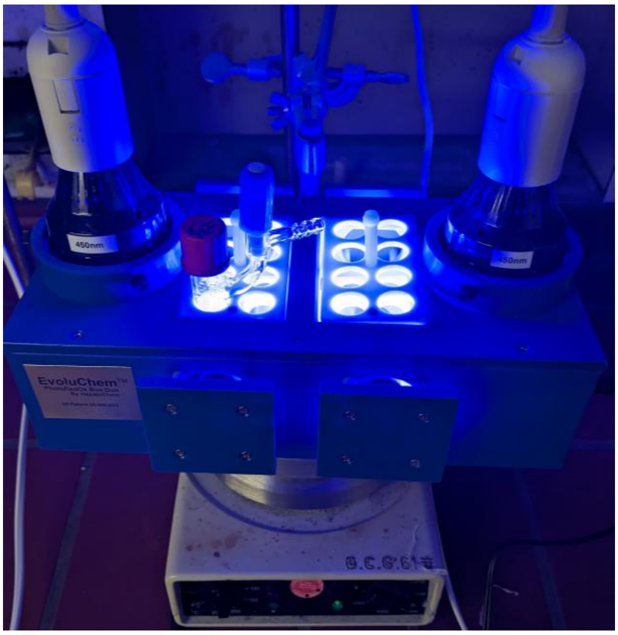

Synthesis of dihydropyrimidine derivatives

General procedure 1a (GP 1a)

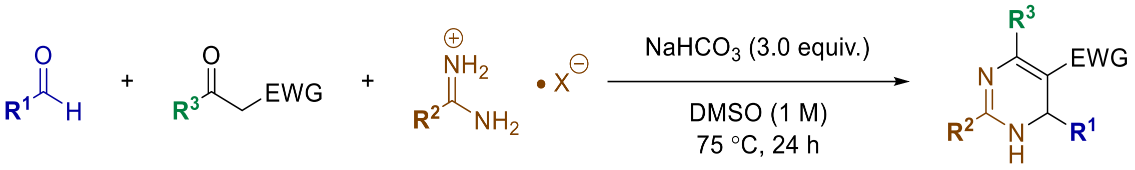

1

In analogy to the literature,[3,4] to a round-bottom flask equipped with a magnetic stir bar was added guanidine, amidine or (thio-)imidate salt (10 mmol, 1.0 equiv.) and $NaHCO_3$ (30 mmol, 3.0 equiv.). DMSO (10 mL, 1 M), aldehyde (10 mmol, 1.0 equiv.) and β-ketoester (10 mmol, 1.0 equiv.) were added while stirring. The reaction was allowed to stir at 75 °C for 24 h. Water and *tert*-butyl methyl ether were added to the reaction mixture and the aqueous phase was extracted with *tert*-butyl methyl ether three times. The organic phase was washed with sat. aq. $NaHCO_3$ solution and dried over $MgSO_4$. Column chromatography on silica gel (*n*-pentane/EtOAc/EtOH) afforded the product **1**.

General procedure 1b (GP 1b)

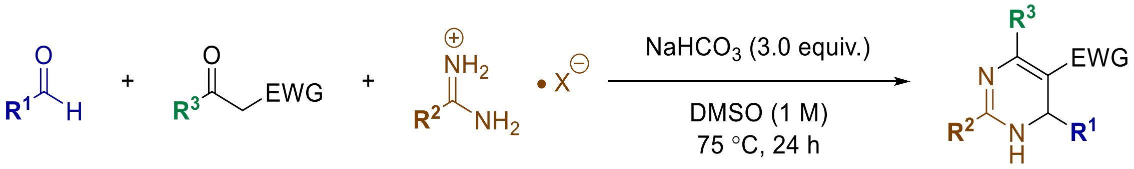

1

To a round-bottom flask equipped with a magnetic stir bar was added guanidine, amidine or (thio-)imidate salt (10 mmol, 1.0 equiv.) and $NaHCO_3$ (30 mmol, 3.0 equiv.). MeCN (10 mL), aldehyde (10 mmol, 1.0 equiv.) and β-ketoester (10 mmol, 1.0 equiv.) were added while stirring. The reaction was allowed to stir at 75 °C for 14 h. Volatiles were removed under vacuum and the crude mixture was subjected to column chromatography on silica gel (*n*-pentane/EtOAc/EtOH) afforded the product **1**.

Notes:

- The addition of 5 equiv. of water or running the Biginelli reaction under air gave nearly the same yield as the reaction under inert atmosphere with anhydrous solvents.

Ethyl 6-cyclohexyl-2-(dimethylamino)-4-methyl-1,6-dihydropyrimidine-5-carboxylate (1a)

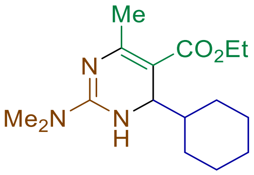

1a

Following GP 1a with cyclohexanecarboxaldehyde (12.1 mL, 100 mmol, 1.0 equiv.), ethyl acetoacetate (12.6 mL, 100 mmol, 1.0 equiv.) and 1,1-dimethylguanidine sulfate salt (2:1) (18.8 g, 50 mmol, 0.5 equiv.), compound **1a** was afforded as a beige solid (17.9 g, 61.0 mmol, 61%). Yield with GP 1b on 50 mmol scale: 67%

$\mathbf{R}_{\boldsymbol{f}}$ (EtOAc/EtOH = 3/1): 0.38.

^{1}H NMR (300 MHz, $CDCl_3$): δ/ppm = 4.87 (s, 1H), 4.24 – 4.02 (m, 3H), 3.03 (s, 6H), 2.32 (s, 3H), 1.80 – 1.34 (m, 6H), 1.25 (t, J = 7.1 Hz, 3H), 1.18 – 0.64 (m, 5H).

^{13}C NMR (75 MHz, $CDCl_3$): δ/ppm = 167.9, 160.7, 154.9, 143.4, 97.7, 59.1, 55.6, 44.6, 37.0, 29.3, 27.4, 26.7, 26.4, 26.2, 24.4, 14.7.

IR (ATR, cm^{-1}): 3508, 3150, 2919, 2850, 1595, 1207, 1068, 958, 714, 532.

HR-MS (ESI): m/z calcd for $[M+Na]^+$ 316.1995, found 316.1985.

Ethyl 6-cyclohexyl-4-methyl-2-(methylthio)-1,6-dihydropyrimidine-5-carboxylate & ethyl 4-cyclohexyl-6-methyl-2-(methylthio)-1,4-dihydropyrimidine-5-carboxylate (1b)

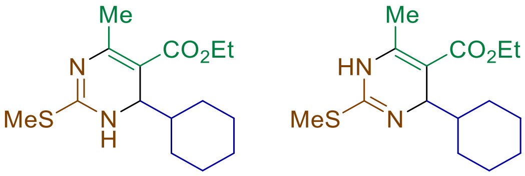

1b

Following GP 1a with cyclohexanecarboxaldehyde (12.1 mL, 100 mmol, 1.0 equiv.), ethyl acetoacetate (12.6 mL, 100 mmol, 1.0 equiv.) and methyl carbamimidothioate sulfate salt (2:1) (18.8 g, 50 mmol, 1.0 equiv.), compound **1b** was afforded as a colorless solid (21.9 g, 73.88 mmol, 74%). In the solid state, the 1,6-dihydropyrimidine was observed, in solution ($CDCl_3$) a tautomer ratio of 1 : 1.1 was observed by NMR.

$\mathbf{R}_{\boldsymbol{f}}$ (*n*-pentane/EtOAc = 2/1): 0.66.

^{1}**H NMR** (400 MHz, $CDCl_3$) of tautomer mixture: δ/ppm = 6.09 (s, 0.5H), 5.58 (s, 0.5H), 4.53 (d, J = 5.3 Hz, 0.5H), 4.28 – 4.07 (m, 2.5H), 2.46 (s, 1.4H), 2.44 (s, 1.3H), 2.33 (s, 1.5H), 2.24 (s, 2H), 1.82 – 1.43 (m, 6H), 1.27 (m, 3H), 1.20 – 0.80 (m, 5H).

^{13}**C NMR of major tautomer** (101 MHz, $CDCl_3$): δ/ppm = 167.8, 155.5, 149.5, 100.3, 61.3, 59.7, 45.5, 29.1, 28.0, 26.8, 26.7, 26.5, 18.8, 14.5, 13.8.

^{13}**C NMR of minor tautomer** (101 MHz, $CDCl_3$): δ/ppm = 167.8, 161.0, 144.2, 104.0, 59.9, 56.0, 44.6, 28.3, 27.1, 26.5, 26.3, 26.1, 23.2, 14.5, 13.8.

IR (ATR, cm^{-1}): 3487, 3282, 2924, 2849, 1642, 1476, 1266, 1096, 741, 602.

HR-MS (ESI): m/z calcd for $[M+Na]^+$ 319.1451, found 319.1451.

Ethyl 6-cyclohexyl-2-methoxy-4-methyl-1,6-dihydropyrimidine-5-carboxylate & ethyl 4-cyclohexyl-2-methoxy-6-methyl-1,4-dihydropyrimidine-5-carboxylate (1c)

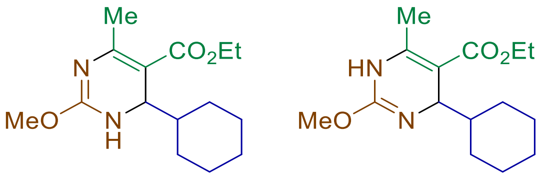

1c

Following GP 1a with cyclohexanecarboxaldehyde (3.6 mL, 30 mmol, 1.0 equiv.), ethyl acetoacetate (3.8 mL, 30 mmol, 1.0 equiv.) and methyl carbamimidate sulfate salt (1:1) (5.19 g, 30 mmol, 1.0 equiv.), compound **1c** was afforded as a yellow oil (3.24 g, 11.5 mmol, 39%). In solution ($CDCl_3$) a tautomer ratio of 1.4 : 1 was observed by NMR.

$\mathbf{R_f}$ (*n*-Pentane/EtOAc = 2/1): 0.56.

^{1}**H NMR** (400 MHz, $CDCl_3$) of tautomeric mixture: δ/ppm = 5.79 (s, 0.3H), 5.04 (s, 0.5H), 4.42 (d, J = 4.6 Hz, 0.4H), 4.26 (t, J = 3.7 Hz, 0.6H), 4.21 – 4.09 (m, 2H), 3.81 (s, 1.7H), 3.75 (s, 1.2H), 2.30 (s, 1.6H), 2.23 (s, 1.2H), 1.75 – 1.35 (m, 6H), 1.31 – 1.22 (m, 3H), 1.17 – 0.84 (m, 5H).

^{13}**C NMR of major tautomer** (75 MHz, $CDCl_3$): δ/ppm = 167.7, 158.5 145.5, 102.5, 59.6, 56.3, 54.3, 44.9, 28.5, 26.54, 26.45, 26.41, 26.2, 23.6, 14.53, 14.45.

^{13}**C NMR of minor tautomer** (75 MHz, $CDCl_3$): δ/ppm = 167.6, 158.3, 150.3, 100.5, 59.7 59.6, 56.2, 45.8, 29.1, 27.5, 26.81, 26.77, 26.56, 22.5, 18.7, 14.2.

IR (ATR, cm^{-1}): 3303, 2924, 2851, 1678, 1547, 1220, 978, 780, 654, 450.

HR-MS (ESI): m/z calcd for $[M+Na]^+$ 335.1941, found 335.1931.

Ethyl 6-cyclohexyl-4-methyl-2-morpholino-1,6-dihydropyrimidine-5-carboxylate (1d)

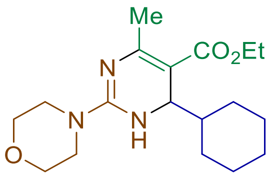

1d

Following GP 1a with cyclohexanecarboxaldehyde (1.2 mL,10 mmol, 1.0 equiv.), ethyl acetoacetate (1.3 mL,10 mmol, 1.0 equiv.) and 4-morpholinecarboximidamide hydrobromide salt (1:1) (2.10 g,10 mmol, 1.0 equiv.), compound **1d** was afforded as a brown solid (2.75 g, 8.2 mmol, 82%).

$\mathbf{R}_{\boldsymbol{f}}$ (EtOAc/EtOH = 3/1): 0.42.

^{1}H NMR (300 MHz, $CDCl_3$): δ/ppm = 5.44 (s, 1H), 4.20 – 3.99 (m, 3H), 3.74 – 3.46 (m, 6H), 3.40 – 3.30 (m, 2H), 2.23 (s, 3H), 1.76 – 1.26 (m, 6H), 1.19 (t, J = 7.1 Hz, 3H), 1.12 – 0.67 (m, 5H).

^{13}C NMR (75 MHz, $CDCl_3$): δ/ppm = 167.9, 159.9, 154.2, 99.1, 77.2, 66.5, 59.1, 55.1, 45.1, 44.2, 28.9, 27.4, 26.6, 26.2, 26.0, 23.9, 14.5.

IR (ATR, cm^{-1}): 3662, 2926, 2859, 1682, 1442, 1204, 1065, 957, 882, 702, 416.

HR-MS (ESI): m/z calcd for $[M+Na]^+$ 358.2101, found 358.2097.

Ethyl 6-cyclohexyl-4-methyl-2-phenyl-1,6-dihydropyrimidine-5-carboxylate (1e)

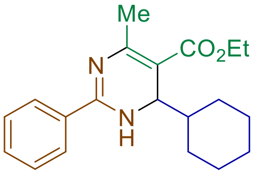

1e

Following GP 1a with cyclohexanecarboxaldehyde (1.2 mL,10 mmol, 1.0 equiv.), ethyl acetoacetate (1.3 mL,10 mmol, 1.0 equiv.) and benzimidamide hydrochloride salt (1.57 g, 10 mmol, 1.0 equiv.), compound **1e** was afforded as a yellowish crystalline solid (2.85 g, 8.70 mmol, 87%).

$\mathbf{R_f}$ (*n*-Pentane/EtOAc = 1/1): 0.41.

^{1}**H NMR** (400 MHz, MeOD): δ/ppm = 7.79 – 7.69 (m, 2H), 7.57– 7.42 (m, 3H), 4.48 – 4.42 (d, *J* = 5.5 Hz, 1H), 4.26 – 4.10 (m, 2H), 2.37 (s, 3H), 1.95 – 1.55 (m, 6H), 1.30 (t, *J* = 7.1 Hz, 4H), 1.24 – 0.96 (m, 4H).

^{13}**C NMR of major tautomer** (101 MHz, MeOD): δ/ppm = 169.2, 156.9, 135.0, 132.3, 129.6, 128.9, 101.2, 60.8, 58.3, 46.9, 29.7, 28.8, 27.8, 27.5, 27.3, 19.8, 14.7.

IR (ATR, cm^{-1}): 3330, 3058, 2979, 2923, 2850, 2162, 1739, 1697, 1695, 1676, 1656, 1602, 1579, 1503, 1482, 1446, 1370, 1343, 1329, 1293, 1240, 1095, 1090, 1029, 965, 884, 786, 776, 694, 672, 560, 533, 458.

HR-MS (ESI): m/z calcd for $[M+Na]^+$ 349.1886, found 349.1887.

***Tert*-butyl 6-cyclohexyl-4-methyl-2-(methylthio)-1,6-dihydropyrimidine-5-carboxylate & *tert*-butyl 4-cyclohexyl-6-methyl-2-(methylthio)-1,4-dihydropyrimidine-5-carboxylate (1f)**

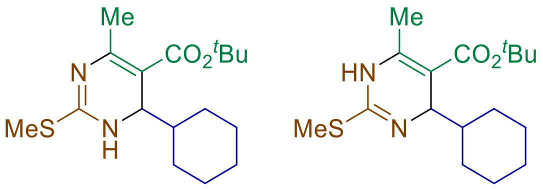

1f

Following GP 1a with cyclohexanecarboxaldehyde (1.2 mL,10 mmol, 1.0 equiv.), *tert*-butyl acetoacetate (1.7 mL, 10 mmol, 1.0 equiv.) and methyl carbamimidothioate sulfate salt (1.88 g, 10 mmol, 1.0 equiv.), compound **1f** was afforded as a colorless solid (1.71 g, 4.9 mmol, 49%). In solution ($CDCl_3$) a tautomer ratio of 1 : 1.4 was observed by NMR.

$\mathbf{R}_f$ (*n*-Pentane/EtOAc = 2/1): 0.56.

^{1}**H NMR** (400 MHz, $CDCl_3$) of tautomeric mixture: δ/ppm = 5.91 (s, 0.5H), 5.46 (s, 0.4H), 4.49 (d, *J* = 5.6 Hz, 0.6H), 4.14 (t, *J* = 4.6 Hz, 0.4H), 2.46 (s, 1.3H), 2.44 (s, 1.7H), 2.30 (s, 5H), 2.22 (s, 4H), 1.85 – 1.57 (m, 5H), 1.49 (s, 4H), 1.46 (s, 5H), 1.43 – 0.85 (m, 6H).

^{13}**C NMR of major tautomer** (75 MHz, $CDCl_3$): δ/ppm = 167.1, 149.9, 143.3, 101.5, 79.7, 61.4, 45.4, 28.4, 28.2, 26.7, 26.5, 26.3, 18.5, 16.3.

^{13}**C NMR of minor tautomer** (75 MHz, $CDCl_3$): δ/ppm = 167.1, 160.5, 154.1, 105.5, 79.7, 55.9, 44.5, 29.1, 28.4, 27.2, 26.7, 26.1, 23.1, 16.3.

IR (ATR, cm^{-1}): 3139, 2917, 2849, 1697, 1487, 1276, 1162, 1008, 666, 434.

HR-MS (ESI): m/z calcd for $[M+Na]^+$ 347.1764, found 347.1768.

Ethyl 2-(4-chlorophenyl)-6-cyclohexyl-4-methyl-1,6-dihydropyrimidine-5-carboxylate (1g)

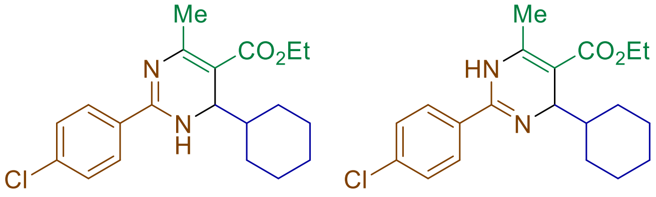

1g

Following GP 1a with cyclohexanecarboxaldehyde (1.2 mL,10 mmol, 1.0 equiv.), ethyl acetoacetate (1.3 mL, 10 mmol, 1.0 equiv.) and 4-chloro-benzenecarboximidamide hydrochloride (1:1) salt (1.91 g, 10 mmol, 1.0 equiv.), compound **1g** was afforded as a yellow solid (2.99 g, 8.3 mmol, 83%). In solution ($CDCl_3$) a tautomer ratio of 1 : 1 was observed by NMR.

R*f* (*n*-Pentane/EtOAc = 2/1): 0.53.

^{1}H NMR (400 MHz, $CDCl_3$) of tautomeric mixture: δ/ppm = 7.76 (d, *J* = 8.4 Hz, 1H), 7.65 (d, *J* = 8.7 Hz, 1H), 7.46 – 7.37 (m, 2H), 6.42 (s, 0.4H), 5.86 (s, 0.4H), 4.67 (d, *J* = 5.0 Hz, 0.4H), 4.44 – 4.40 (m, 0.4H), 4.26 – 4.13 (m, 2H), 2.42 (s, 1.5H), 2.35 (s, 1.5H), 1.89 – 1.62 (m, 6H), 1.30 (m, 3H), 1.25 – 0.84 (m, 5H).

^{13}C NMR (126 MHz, $CDCl_3$, 55 °C) of tautomeric mixture: δ/ppm = 167.7, 156.5, 155.8, 148.7, 144.0, 137.9, 136.8, 133.0, 129.2, 129.1, 129.00, 128.8, 127.9, 104.4, 99.8, 60.4, 59.8, 55.1, 46.0, 45.4, 29.0, 28.7, 28.2, 27.7, 27.3, 26.8, 26.6, 26.4, 23.3, 19.0, 14.5.

IR (ATR, cm^{-1}): 2929, 2854, 1688, 1482, 1227, 1091, 835, 670.

HR-MS (ESI): m/z calcd for $[M+H]^+$ 361.1677, found 361.1683.

Ethyl 5-cyclohexyl-7-methyl-5,8-dihydroimidazo[1,2-*a*]pyrimidine-6-carboxylate (1h)

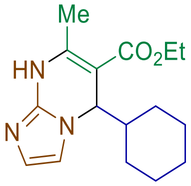

1h

Following GP 1a with cyclohexanecarboxaldehyde (1.2 mL,10 mmol, 1.0 equiv.), ethyl acetoacetate (1.3 mL,10 mmol, 1.0 equiv.) and 1*H*-imidazol-2-amine sulfate salt (2:1) (2.64 g, 10 mmol, 1.0 equiv.), compound **1h** was afforded as yellow crystals (1.20 g, 4.1 mmol, 41%).

R_f (*n*-Pentane /EtOAc = 2/1): 0.29.

^{1}H NMR (400 MHz, $CDCl_3$) δ/ppm = 12.00 (s, 1H), 6.74 (s, 1H), 6.58 (s, 1H), 5.04 (s, 1H), 4.27 – 4.12 (m, 2H), 2.51 (s, 3H), 1.80 – 1.69 (m, 2H), 1.69 – 1.52 (m, 4H), 1.30 (t, *J* = 7.1 Hz, 3H), 1.24 – 1.05 (m, 3H), 1.05 – 0.91 (m, 1H), 0.51 – 0.38 (m, 1H).

^{13}C NMR (101 MHz, $CDCl_3$) δ/ppm = 167.1, 149.0, 143.5, 123.8, 114.2, 94.4, 59.6, 59.4, 46.8, 32.1, 26.8, 26.4, 26.3, 26.2, 19.4, 14.6.

IR (ATR, cm^{-1}): 3111, 2930, 2851, 1683, 1528, 1254, 1118, 1066, 779, 717, 571.

HR-MS (ESI): m/z calcd for $[M+H]^+$ 290.1863, found 290.1871.

Ethyl 6-cyclopentyl-2-(dimethylamino)-4-methyl-1,6-dihydropyrimidine-5-carboxylate (1i)

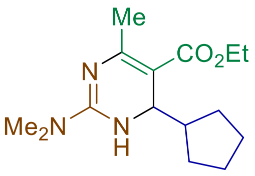

1i

Following GP 1a with cyclopentanecarbaldehyde (1.1 mL, 10.0 mmol, 1.0 equiv,), ethyl acetoacetate (1.3 mL,10 mmol, 1.0 equiv.) and 1,1-dimethylguanidine sulfate (2:1) salt (2.72 g, 10.0 mmol, 1.0 equiv.), compound **1i** was afforded as a colorless solid (1.59 g, 5.68 mmol, 57%).

R_f (EtOAc /EtOH = 3/1): 0.16.

^{1}H NMR (400 MHz, $CDCl_3$) δ/ppm = 4.91 (s, 1H), 4.31 – 4.24 (m, 1H), 4.21 – 4.08 (m, 2H), 3.04 (s, 6H), 2.33 (s, 3H), 2.12 – 2.02 (m, 1H), 1.70 – 1.42 (m, 7H), 1.29 – 1.23 (m, 4H).

^{13}C NMR (101 MHz, $CDCl_3$) δ/ppm = 167.9, 160.3, 154.7, 99.4, 59.1, 53.7, 46.8, 37.0, 28.5, 28.3, 25.3, 24.9, 24.3, 14.7.

IR (ATR, cm^{-1}): 2954, 2865, 1680, 1568, 1196, 1056, 961, 895, 692.

HR-MS (ESI): m/z calcd for $[M+H]^+$ 280.2020, found 280.2024.

Ethyl 6-(1-(benzo[*d*][1,3]dioxol-5-yl)propan-2-yl)-2-(dimethylamino)-4-methyl-1,6-dihydropyrimidine-5-carboxylate (1j)

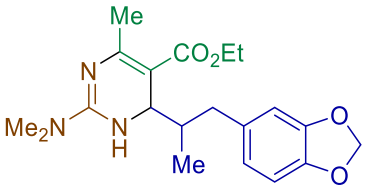

1j

Following GP 1a with 3-(benzo[*d*][1,3]dioxol-5-yl)-2-methylpropanal (1.6 mL, 10 mmol, 1.0 equiv.), ethyl acetoacetate (1.3 mL,10 mmol, 1.0 equiv.) and 1,1-dimethylguanidine sulfate (2:1) salt (2.72 g, 10.0 mmol, 1.0 equiv.), compound **1j** was afforded as a colorless solid in a diastereomeric mixture (2.34 g, 6.28 mmol, 63%) in a diastereomeric mixture (d.r. 1.4:1).

R_f (EtOAc/EtOH = 3/1): 0.55.

^{1}H NMR (400 MHz, $CDCl_3$) δ/ppm = 6.78 – 6.54 (m, 3H), 5.94 (s, 2H), 5.30 – 4.43 (m, 1H), 4.44 – 4.40 (m, 1H), 4.27 – 4.06 (m, 2H), 3.06 (d, *J* = 19.6 Hz, 6H), 2.63 – 2.44 (m, 1H), 2.39 (d, *J* = 8.0 Hz, 3H), 2.06 – 1.89 (m, 2H), 1.33 – 1.27 (m, 3H), 0.86 – 0.78 (m, 3H).

^{13}C NMR (101 MHz, $CDCl_3$) of diastereomeric mixture δ/ppm = 167.8, 167.6, 161.3, 161.0, 155.0, 154.7, 147.8, 147.5, 145.8, 145.5, 135.2, 134.5, 121.8, 121.6, 109.4, 108.2, 108.0, 100.9, 100.8, 97.5, 97.3, 60.5, 60.3, 59.1, 59.0, 55.8, 54.0, 42.2, 41.5, 39.7, 37.3, 37.0, 36.8, 24.3, 15.3, 14.7 (d, *J* = 3.8 Hz), 13.4.

IR (ATR, cm^{-1}): 3520, 3223, 2928, 1665, 1596, 1487, 1441, 1377, 1316, 1209, 1064, 802, 709.

HR-MS (ESI): m/z calcd for $[M+H]^+$ 374.2074, found 374.2078.

Ethyl 6-(2,2-dimethyl-1,3-dioxolan-4-yl)-2-(dimethylamino)-4-methyl-1,6-dihydropyrimidine-5-carboxylate (1k)

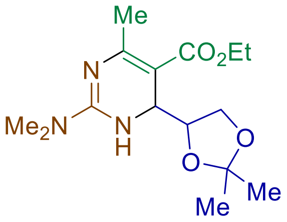

1k

Following GP 1a with (*R*)-2,2-dimethyl-1,3-dioxolane-4-carbaldehyde (1.2 mL, 10 mmol, 1.0 equiv.), ethyl acetoacetate (1.3 mL,10 mmol, 1.0 equiv.) and 1,1-dimethylguanidine sulfate

(2:1) salt (2.72 g, 10.0 mmol, 1.0 equiv.), compound **1k** was afforded as a yellowish solid (0.50g 1.61 mmol, 16%).

R*f* (DCM/MeOH = 4/1): 0.77.

^{1}H NMR (300 MHz, $CDCl_3$) δ/ppm = 5.34 (br s, 1H), 4.31 (d, *J* = 8.5 Hz, 1H), 4.15 (qt, *J* = 7.5, 3.8 Hz, 2H), 4.01 (dt, *J* = 8.4, 6.2 Hz, 1H), 3.85 (d, *J* = 6.3 Hz, 2H), 3.08 (s, 6H), 2.34 (s, 3H), 1.40 (s, 3H), 1.32 – 1.25 (m, 6H).

^{13}C NMR (101 MHz, $CDCl_3$) δ/ppm = 167.2, 163.0, 155.2, 108.9, 94.9, 66.3, 59.3, 53.1, 37.1, 27.0, 25.7, 24.5, 14.7.

IR (ATR, cm^{-1}): 3380, 2981, 2893, 1684, 1581, 1428, 1334, 1277, 1204, 1064, 954.

HR-MS (ESI): m/z calcd for $[M+H]^+$ 312.1918, found 312.1917.

Ethyl 2-(dimethylamino)-4-methyl-6-(tetrahydrofuran-3-yl)-1,6-dihydropyrimidine-5-carboxylate (1l)

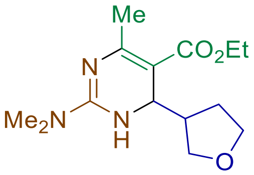

1l

Following GP 1a with tetrahydrofuran-3-carboxaldehyde (905 µL, 10 mmol, 1.0 equiv.), ethyl acetoacetate (1.3 mL,10 mmol, 1.0 equiv.) and 1,1-dimethylguanidine sulfate (2:1) salt (2.72 g, 10.0 mmol, 1.0 equiv.), compound **1l** was afforded as an orange solid (1.01 g, 3.59 mmol, 36%) in a diastereomeric mixture (d.r. 1.5:1).

R*f* (DCM/MeOH = 4/1): 0.43.

^{1}H NMR (300 MHz, $CDCl_3$) δ/ppm = 5.01 (br s, 1H), 4.52 – 4.37 (m, 1H), 4.25 – 4.04 (m, 2H), 3.95 – 3.76 (m, 1H), 3.68 (dddd, *J* = 15.1, 8.5, 6.7, 2.3 Hz, 3H), 3.04 (d, *J* = 1.8 Hz, 6H), 2.49 (dq, *J* = 14.1, 7.0 Hz, 1H), 2.34 (d, *J* = 3.2 Hz, 3H), 1.99 – 1.66 (m, 2H), 1.27 (td, *J* = 7.1, 0.9 Hz, 3H).

^{13}C NMR (101 MHz, $CDCl_3$) of diastereomeric mixture: δ/ppm = 167.6, 167.5, 154.8, 154.3, 98.8, 97.7, 70.9, 70.1, 68.3, 68.3, 59.3, 59.3, 53.3, 51.8, 45.9, 45.7, 37.2, 37.0, 28.4, 27.4, 24.5, 24.2, 14.7.

IR (ATR, cm^{-1}): 3500, 2971, 2866, 1663, 1580, 1472, 1307, 1207, 1126, 1066, 953.

HR-MS (ESI): m/z calcd for $[M+H]^+$ 282.1812, found 282.1823.

Ethyl 2-(dimethylamino)-6-isopropyl-4-methyl-1,6-dihydropyrimidine-5-carboxylate (1m)

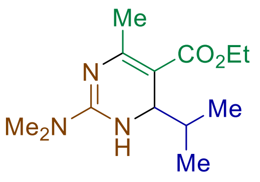

1m

Following GP 1a with isobutyraldehyde (912 µL, 10 mmol, 1.0 equiv.), ethyl acetoacetate (1.3 mL,10 mmol, 1.0 equiv.) and 1,1-dimethylguanidine sulfate (2:1) salt (2.72 g, 10.0 mmol, 1.0 equiv.), compound **1m** was afforded as a yellow solid (1.75 g, 6.91 mmol, 69%).

R_f (DCM/MeOH = 4/1): 0.50.

^{1}H NMR (300 MHz, $CDCl_3$) δ/ppm = 4.82 (br s, 1H), 4.21 (d, *J* = 4.4 Hz, 1H), 4.13 (p, *J* = 7.1 Hz, 2H), 3.04 (s, 6H), 2.33 (s, 3H), 1.79 (pd, *J* = 6.8, 4.3 Hz, 1H), 1.25 (t, *J* = 7.1 Hz, 3H), 0.86 (d, *J* = 7.0 Hz, 3H), 0.80 (d, *J* = 6.8 Hz, 3H).

^{13}C NMR (101 MHz, $CDCl_3$) δ/ppm = 167.9, 160.8, 154.9, 97.9, 59.0, 56.2, 36.9, 34.6, 24.4, 18.8, 16.6, 14.7.

IR (ATR, cm^{-1}): 3518, 3337, 2955, 2870, 1664, 1630, 1570, 1445, 1376, 1234, 1205, 1066.

HR-MS (ESI): m/z calcd for $[M+H]^+$ 254.1863, found 254.1868.

Ethyl 2-(dimethylamino)-4-methyl-6-(6-methylhept-5-en-2-yl)-1,6-dihydropyrimidine-5-carboxylate (1n)

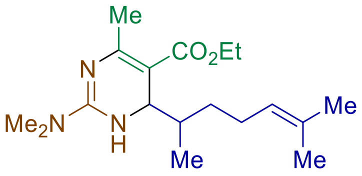

1n

Following GP 1a with 2,6-dimethyl-5-heptenal (1.6 mL, 10 mmol, 1.0 equiv.), ethyl acetoacetate (1.3 mL,10 mmol, 1.0 equiv.) and 1,1-dimethylguanidine sulfate (2:1) salt (2.72 g, 10.0 mmol, 1.0 equiv.), compound **1n** was afforded as a yellowish solid (1.88 g, 5.85 mmol, 59%) in a diastereomeric mixture (d.r. 1.6:1).

R_f (DCM/MeOH = 4/1): 0.54.

^{1}**H NMR** (300 MHz, $CDCl_3$) δ/ppm = 5.12 – 4.93 (m, 1H), 4.39 – 4.19 (m, 1H), 4.17 – 3.98 (m, 2H), 2.99 (d, *J* = 1.1 Hz, 6H), 2.29 (s, 3H), 2.05 – 1.71 (m, 2H), 1.66 – 1.59 (m, 4H), 1.54 (d, *J* = 10.4 Hz, 4H), 1.38 – 1.07 (m, 4H), 0.84 – 0.70 (m, 3H).

^{13}**C NMR (101 MHz, $CDCl_3$)** of diastereomeric mixture: δ/ppm = 167.9, 167.7, 161.0, 159.4, 155.1, 154.8, 131.8, 131.4, 124.9, 124.7, 97.9, 97.6, 59.0, 54.9, 39.7, 39.2, 37.0, 36.9, 33.5, 30.6, 25.9, 25.8, 25.8, 24.3, 17.8, 17.8, 15.6, 14.7, 14.6, 13.5.

IR (ATR, cm^{-1}): 3526, 3157, 2924, 1663, 1592, 1448, 1377, 1205, 1142, 1069, 955.

HR-MS (ESI): m/z calcd for $[M+H]^+$ 322.2489, found 322.2493.

Ethyl 2-(dimethylamino)-6-(1-(4-isopropylphenyl)propan-2-yl)-4-methyl-1,6-dihydropyrimidine-5-carboxylate (1o)

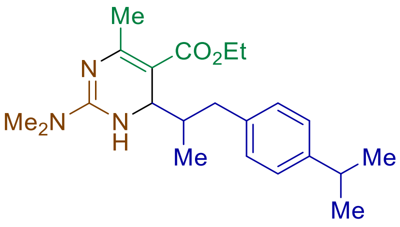

1o

Following GP 1a with 3-(4-Isopropylphenyl)-2-methylpropanal (2.0 mL, 10 mmol, 1.0 equiv.), ethyl acetoacetate (1.3 mL,10 mmol, 1.0 equiv.) and 1,1-dimethylguanidine sulfate (2:1) salt (2.72 g, 10.0 mmol, 1.0 equiv.), compound **1o** was afforded as a yellowish solid (2.34 g, 6.30 mmol, 63%) in a diastereomeric mixture (d.r. 1.2:1).

R*f* (DCM/MeOH = 4/1): 0.65.

^{1}**H NMR** (300 MHz, $CDCl_3$) δ/ppm = 7.18 – 7.06 (m, 3H), 7.01 (d, *J* = 8.1 Hz, 1H), 4.49 – 4.38 (m, 1H), 4.25 – 4.02 (m, 2H), 2.97 (s, 3H), 2.86 (s, 4H), 2.59 (d, *J* = 6.7 Hz, 1H), 2.34 (d, *J* = 8.8 Hz, 3H), 2.19 – 1.91 (m, 1H), 1.76 (s, 1H), 1.29 – 1.24 (m, 3H), 1.23 (d, *J* = 1.1 Hz, 3H), 1.20 (d, *J* = 1.1 Hz, 3H), 0.80 (dd, *J* = 12.0, 6.9 Hz, 3H).

^{13}**C NMR** (101 MHz, $CDCl_3$) of diastereomeric mixture: δ/ppm 167.9, 167.7, 161.4, 160.9, 155.0, 154.6, 146.8, 146.3, 138.8, 138.0, 129.2, 129.0, 126.8, 126.4, 97.7, 97.3, 59.1, 59.0, 55.9, 54.4, 41.9, 41.5, 40.1, 37.9, 36.9, 36.7, 33.8, 33.8, 24.5, 24.4, 24.2, 15.7, 14.7, 14.7, 13.3.

IR (ATR, cm^{-1}): 3513, 3152, 2927, 1663, 1593, 1149, 1206, 1142, 1068, 956.

HR-MS (ESI): m/z calcd for $[M+H]^+$ 372.2646, found 372.2645.

Ethyl 6-benzyl-2-(dimethylamino)-4-methyl-1,6-dihydropyrimidine-5-carboxylate (1p)

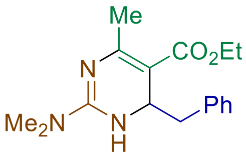

1p

Following GP 1a with phenylacetaldehyde (1.20 g, 10 mmol, 1.0 equiv.), ethyl acetoacetate (1.3 mL,10 mmol, 1.0 equiv.) and 1,1-dimethylguanidine sulfate (2:1) salt (2.72 g, 10.0 mmol, 1.0 equiv.), compound **1p** was afforded as a yellowish solid (0.55 g, 1.8 mmol, 18%).

$\mathbf{R_f}$ (EtOAc/EtOH = 3/1): 0.23.

^{1}H NMR (400 MHz, $CDCl_3$): δ/ppm = 7.35 – 7.28 (m, 2H), 7.26 – 7.21 (m, 2H), 7.20 – 7.14 (m, 2H), 4.47 (dd, *J* = 9.9, 3.2 Hz, 1H), 4.28 – 4.11 (m, 2H), 2.96 (s, 6H), 2.87 (dd, *J* = 13.2, 3.2 Hz, 1H), 2.63 (dd, *J* = 13.3, 10.0 Hz, 1H), 2.37 (s, 3H), 1.33 (t, *J* = 7.1 Hz, 3H).

^{13}C NMR (101 MHz, $CDCl_3$): δ/ppm = 167.0, 153.8, 138.5, 129.6, 128.8, 126.7, 126.6, 100.0, 59.5, 53.2, 42.0, 37.1, 14.7.

HR-MS (ESI): m/z calcd for $[M+Na]^+$ 324.1682, found 324.1682.

Ethyl 6-(*tert*-butyl)-2-(dimethylamino)-4-methyl-1,6-dihydropyrimidine-5-carboxylate (1q)

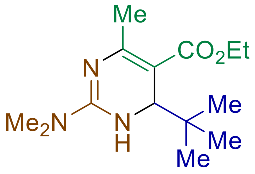

1q

Following GP 1a with pivaldehyde (1.1 mL, 10 mmol, 1.0 equiv.), ethyl acetoacetate (1.3 mL,10 mmol, 1.0 equiv.) and 1,1-dimethylguanidine sulfate (2:1) salt (2.72 g, 10.0 mmol, 1.0 equiv.), compound **1q** was afforded as a yellowish solid (1.54 g, 5.70 mol, 57%).

$\mathbf{R_f}$ (EtOAc/EtOH = 3/1): 0.28.

^{1}H NMR (400 MHz, $CDCl_3$): δ/ppm = 4.92 (br s,1H), 4.21 (s, 1H), 4.17 – 1.07 (dd, *J* = 7.1 Hz, *J* = 5.0 Hz, 2H), 3.05 (s, 6H), 2.33 (s, 3H) 1.26 (t, 3H), 0.80 (s, 9H).

^{13}C NMR (101 MHz, $CDCl_3$): δ/ppm = 168.8, 160.9, 160.2, 154.8, 96.6, 59.1, 58.7, 40.1, 37.0, 25.2, 14.6, 14.4.

IR (ATR, cm^{-1}): 3350, 2923, 2852, 1661, 1638, 1547, 1466, 1374, 1348, 1315, 1234, 1207, 1167, 1073, 1025, 956, 899, 871, 775, 749, 720, 615, 529, 473, 429, 387.

HR-MS (ESI): m/z calcd for $[M+Na]^+$ 268.2020, found 268.2016.

Ethyl 6-(1-(tert-butoxycarbonyl)pyrrolidin-3-yl)-2-(dimethylamino)-4-methyl-1,6-dihydropyrimidine-5-carboxylate (1r)

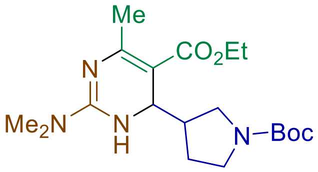

1r

Following GP 1a with *tert*-butyl 3-formylpyrrolidine-1-carboxylate (1.8 mL, 10 mmol, 1.0 equiv.), ethyl acetoacetate (1.3 mL,10 mmol, 1.0 equiv.) and 1,1-dimethylguanidine sulfate (2:1) salt (2.72 g, 10.0 mmol, 1.0 equiv.), compound **1r** was afforded as a yellowish solid (2.57 g, 6.76 mmol, 67%).

R_f (EtOAc/EtOH = 3/1): 0.25.

^{1}H NMR (400 MHz, $CDCl_3$) δ/ppm = 5.61 – 5.02 (m, 1H), 4.45 – 4.24 (m, 1H), 4.20 – 4.08 (m, 2H), 3.50 – 3.31 (m, 2H), 3.23 – 3.09 (m, 2H), 3.02 (d, *J* = 6.6 Hz, 7H), 2.33 (s, 3H), 1.80 – 1.61 (m, 2H), 1.43 (s, 9H), 1.26 (t, *J* = 7.1 Hz, 3H).

^{13}C NMR (101 MHz, $CDCl_3$) δ/ppm = 167.7, 161.6, 154.9, 154.7, 154.2, 98.4, 79.3, 59.2, 51.8, 48.9, 48.2, 46.1, 45.6 (d), 43.9, 37.2, 28.7, 27.8, 27.0, 24.4, 14.7.

IR (ATR, cm^{-1}): 3344, 2975, 2930, 2874, 1666, 1570, 1474, 1403, 1365, 1239, 1203, 1164, 1123, 1061, 955, 874, 749.

HR-MS (ESI): m/z calcd for $[M+H]^+$ 381.2496, found 381.2501.

Ethyl 6-butyl-2-(dimethylamino)-4-methyl-1,6-dihydropyrimidine-5-carboxylate (1s)

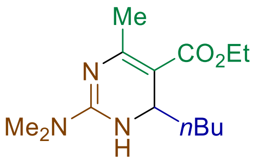

1s

Following GP 1b with pentanal (1.1 mL, 10 mmol, 1.0 equiv.), ethyl acetoacetate (1.3 mL,10 mmol, 1.0 equiv.) and 1,1-dimethylguanidine sulfate (2:1) salt (2.72 g, 10.0 mmol, 1.0 equiv.), compound **1s** was afforded as a yellowish oil (1.21 g, 4.52 mmol, 45%).

R_f (EtOAc/EtOH = 3/1): 0.40.

^{1}H NMR (300 MHz, $CDCl_3$) δ/ppm = 5.03 (s, 1H), 4.27 (dd, *J* = 8.3, 3.8 Hz, 1H), 4.20 – 4.06 (m, 2H), 3.01 (s, 6H), 2.29 (s, 3H), 1.51 – 1.13 (m, 9H), 0.90 – 0.80 (m, 3H).

^{13}C NMR (101 MHz, $CDCl_3$) δ/ppm = 167.4, 160.2, 154.3, 99.59, 59.0, 50.6, 36.9, 35.8, 27.3, 24.1, 22.5, 14.6, 14.1

IR (ATR, cm^{-1}): 3352, 2955, 2929, 2858, 1739, 1657, 1567, 1464, 1371, 1314, 1253, 1193, 1137, 1063, 953, 893, 774, 606.

HR-MS (ESI): m/z calcd for $[M+H]^+$ 268.2020, found 268.2022.

Ethyl 2-(dimethylamino)-4-methyl-6-((3a*S*,5a*R*,8a*R*,8b*S*)-2,2,7,7-tetramethyltetrahydro-5*H*-bis([1,3]dioxolo)[4,5-*b*:4',5'-*d*]pyran-5-yl)-1,6-dihydropyrimidine-5-carboxylate (1t)

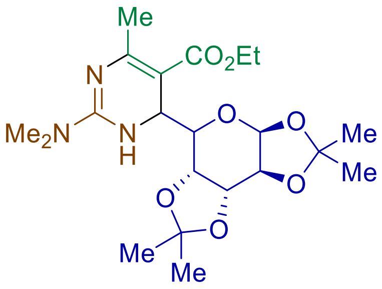

1t

Following GP 1a with (3a*S*,5a*S*,8a*R*,8b*S*)-2,2,7,7-tetramethyltetrahydro-5*H*-bis([1,3]dioxolo)[4,5-*b*:4',5'-*d*]pyran-5-carbaldehyde (1.92 g, 7.44 mmol, 1.0 equiv.), ethyl acetoacetate (949 µL, 7.44 mmol, 1.0 equiv.) and 1,1-dimethylguanidine sulfate (2:1) salt

(2.03 g, 7.44 mmol, 1.0 equiv.), compound **1t** was afforded as a yellowish solid (1.91 g, 4.35 mmol, 58%).

^{1}H NMR (400 MHz, $CDCl_3$) δ 5.50 (d, *J* = 4.8 Hz, 1H), 5.48 (s, 1H), 4.58 (dd, *J* = 7.2, 1.9 Hz, 1H), 4.47 (dd, *J* = 8.1, 2.0 Hz, 1H), 4.21 – 3.99 (m, 5H), 3.71 (dd, *J* = 7.0, 1.2 Hz, 1H), 3.05 (s, 6H), 2.32 (s, 3H), 1.46 (s, 3H), 1.37 (s, 3H), 1.29 (s, 3H), 1.28 – 1.26 (m, 5H).

^{13}C NMR (101 MHz, $CDCl_3$) δ 167.6, 162.3, 155.6, 108.9, 108.7, 96.7, 95.2, 71.7, 71.1, 70.9, 67.9, 59.2, 50.8, 37.0, 26.2, 26.2, 25.3, 24.0, 23.8, 14.6.

IR (ATR, cm^{-1}): 2986, 2933, 2900, 1738, 1684, 1682, 1566, 1562, 1478, 1472, 1436, 1432, 1370, 1369, 1341, 1309, 1254, 1253, 1205, 1166, 1165, 1139, 1064, 1062, 997, 959, 896, 874, 837, 773, 753, 619, 590, 573, 549, 516, 478, 424, 422, 399, 383.

HR-MS (ESI): m/z calcd for $[M+H]^+$ 440.2391, found 440.2402.

Ethyl 6-(cyclopropylmethyl)-2-(dimethylamino)-4-methyl-1,6-dihydropyrimidine-5-carboxylate (1u)

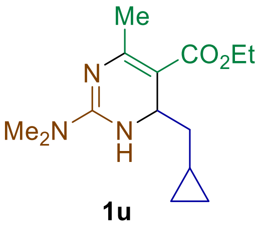

1u

Following GP 1b in MeOH instead of MeCN with 2-cyclopropylacetaldehyde (421 mg, 5.0 mmol. 1.0 equiv.), ethyl acetoacetate (0.644 mL, 5 mmol, 1.0 equiv.) and 1,1-dimethylguanidine sulfate (2:1) salt (0.681 g, 2.5 mmol, 0.5 equiv.), compound **1u** was afforded as a yellowish solid (366 mg, 1.37 mmol, 27%).

^{1}H NMR (400 MHz, $CDCl_3$) δ 5.24 (s, 1H), 4.39 (dd, *J* = 8.9, 3.6 Hz, 1H), 4.19 – 4.02 (m, 2H), 3.05 (s, 6H), 2.30 (s, 3H), 1.50 – 1.37 (m, 1H), 1.31 – 1.16 (m, 4H), 0.70 – 0.56 (m, 1H), 0.51 – 0.32 (m, 2H), 0.06 – -0.04 (m, 2H).

^{13}C NMR (101 MHz, $CDCl_3$) δ 167.2, 154.2, 99.8, 59.1, 51.4, 40.8, 37.1, 23.7, 14.6, 7.2, 4.9, 3.4.

HR-MS (ESI): m/z calcd for $[M+H]^+$ 266.1863, found 266.1863.

Ni-catalyzed Giese addition: General procedure 2 (GP 2)

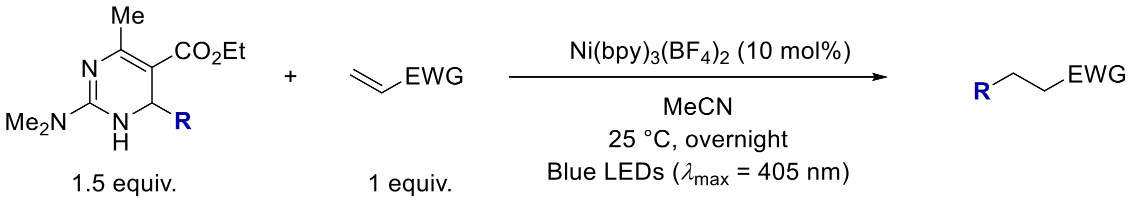

Following Melchiorre's conditions adapted to our lab,[5] to an oven-dried screw-capped Schlenk tube equipped with a magnetic stir bar was added $Ni(bpy)_3(BF_4)_2$ (0.05 mmol, 0.1 equiv., 35 mg) and dihydropyrimidine **1** (0.75 mmol, 1.5 equiv.). After evacuating and refilling with argon for at least three cycles, dry MeCN (1 mL) and dry DCM (1 mL) was added, followed by the alkene (0.5 mmol, 1.0 equiv.) and the reaction was degassed for 5 min. The reaction was allowed to stir in the photoreactor with 405 nm wavelength for 24 h. The sample was transferred to a round-bottom flask, concentrated *in vacuo* and absorbed on silica gel for subsequent column chromatography.

Dimethyl 2-cyclohexylsuccinate (3a)

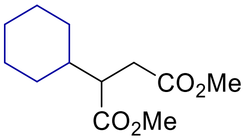

3a

Synthesized following GP 2 with ethyl 6-cyclohexyl-2-(dimethylamino)-4-methyl-1,6-dihydropyrimidine-5-carboxylate **1a** (220 mg, 0.75 mmol, 1.5 equiv.) and dimethyl fumarate (72.1 mg, 0.5 mmol, 1.0 equiv.). Purification by column chromatography on silica gel (*n*-pentane/EtOAc) afforded the product as a colorless solid (94 mg, 0.41 mmol, 83%).

R_f (*n*-pentane/EtOAc = 5/1): 0.69.

^{1}H NMR (400 MHz, $CDCl_3$) δ/ppm = 3.67 (s, 3H), 3.64 (s, 3H), 2.77 – 2.64 (m, 2H), 2.43 (dt, *J* = 13.1, 8.9 Hz, 1H), 1.77 – 1.67 (m, 2H), 1.67 – 1.52 (m, 4H), 1.27 – 0.93 (m, 5H).

^{13}C NMR (101 MHz, $CDCl_3$) δ/ppm = 175.1, 173.1, 51.5, 51.7, 47.1, 40.0, 33.3, 30.7, 30.2, 26.4, 26.2.

Spectral data are in accordance with the literature.[6]

The reaction was repeated as the following variations:

- in the presence of 1 equiv. TEMPO (complete shutdown of the reaction)
- under air, closed flask (66% yield of product **3a**)

- with 5 equiv. H_2O (74% yield of product **3a**)

Ethyl 4-cyclohexyl-2-oxochromane-3-carboxylate (3b)

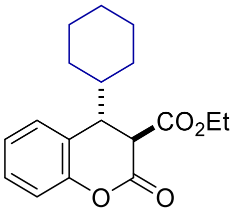

3b

Synthesized following GP 2 with ethyl 6-cyclohexyl-2-(dimethylamino)-4-methyl-1,6-dihydropyrimidine-5-carboxylate **1a** (220 mg, 0.75 mmol, 1.5 equiv.) and 3-carbethoxycoumarin (109.1 mg, 0.5 mmol, 1.0 equiv.). Purification by column chromatography on silica gel (*n*-pentane/EtOAc) afforded the product as a colorless solid (82 mg, 0.27 mmol, 54%).

$\mathbf{R}_f$ (*n*-pentane/EtOAc = 5/1): 0.55.

^{1}H NMR (400 MHz, $CDCl_3$) δ/ppm = 7.28 – 7.22 (m, 1H), 7.14 – 7.03 (m, 3H), 4.04 (dq, *J* = 10.8, 7.1 Hz, 1H), 4.00 – 3.93 (m, 1H), 3.92 (d, *J* = 1.7 Hz, 1H), 3.10 (dd, *J* = 8.1, 1.7 Hz, 1H), 1.87 – 1.79 (m, 1H), 1.79 – 1.66 (m, 2H), 1.64 – 1.53 (m, 2H), 1.40 (tdt, *J* = 11.3, 8.1, 3.3 Hz, 1H), 1.21 – 0.92 (m, 8H).

^{13}C NMR (101 MHz, $CDCl_3$) δ/ppm = 167.7, 165.2, 151.1, 130.0, 128.8, 124.4, 123.2, 117.0, 62.2, 49.9, 46.2, 41.3, 30.6, 30.1, 26.1, 26.0, 13.8.

Spectral data are in accordance with the literature.[7]

Benzyl 3-cyclohexyl-2-methylpropanoate (3c)

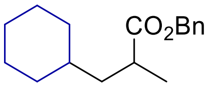

3c

Synthesized following GP 2 with ethyl 6-cyclohexyl-2-(dimethylamino)-4-methyl-1,6-dihydropyrimidine-5-carboxylate **1a** (220 mg, 0.75 mmol, 1.5 equiv.) and benzyl methacrylate (84 µL mg, 0.5 mmol, 1.0 equiv.). Purification by column chromatography on silica gel (*n*-pentane/TBME) afforded the product as a colorless oil (76 mg, 0.29 mmol, 58%).

$\mathbf{R}_f$ (*n*-pentane/TBME = 4/1): 0.53.

¹H NMR (400 MHz, $CDCl_3$) δ/ppm = 7.40 – 7.28 (m, 5H), 5.19 – 5.01 (m, 2H), 2.65 – 2.55 (m, 1H), 1.76 – 1.53 (m, 6H), 1.28 – 1.08 (m, 8H), 0.93 – 0.77 (m, 2H).

¹³C NMR (101 MHz, $CDCl_3$) δ/ppm = 177.2, 136.4, 128.7, 128.2, 66.1, 41.8, 37.1, 35.5, 33.4, 33.3, 26.7, 26.4, 17.8.

Spectral data are in accordance with the literature.[8]

Diethyl 2-(1-cyclohexylethyl)malonate (3d)

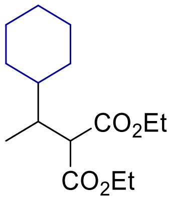

3d

Synthesized following GP 2 with ethyl 6-cyclohexyl-2-(dimethylamino)-4-methyl-1,6-dihydropyrimidine-5-carboxylate **1a** (220 mg, 0.75 mmol, 1.5 equiv.) and diethyl ethylidenemalonate (91.4 µL, 0.5 mmol, 1.0 equiv.). Purification by column chromatography on silica gel (*n*-pentane/EtOAc) afforded the product as a colorless oil (64 mg, 0.24 mmol, 47%).

$\mathbf{R}_f$ (*n*-pentane/EtOAc = 5/1): 0.50.

¹H NMR (300 MHz, $CDCl_3$) δ/ppm = 4.30 – 4.09 (m, 4H), 3.39 (d, *J* = 9.2 Hz, 1H), 2.26 – 2.08 (m, 1H), 1.81 – 1.51 (m, 5H), 1.33 – 1.02 (m, 12H), 0.90 (d, *J* = 7.0 Hz, 3H).

¹³C NMR (75 MHz, $CDCl_3$) δ/ppm = 169.5, 169.2, 61.3, 61.2, 56.0, 40.4, 38.7, 31.7, 27.6, 26.9, 26.7, 26.6, 14.3, 13.1.

Spectral data are in accordance with the literature.[9]

2-(Cyclohexyl(phenyl)methyl)malononitrile (3e)

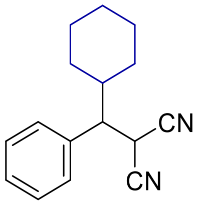

3e

Synthesized following GP 2 with ethyl 6-cyclohexyl-2-(dimethylamino)-4-methyl-1,6-dihydropyrimidine-5-carboxylate **1a** (220 mg, 0.75 mmol, 1.5 equiv.) and benzalmalononitrile (77.1 mg, 0.5 mmol, 1.0 equiv.). Purification by column chromatography on silica gel (*n*-pentane/EtOAc) afforded the product as a colorless solid (66 mg, 0.277 mmol, 55%).

R*f* (DCM): 0.89.

^{1}H NMR (400 MHz, $CDCl_3$) δ/ppm = 7.44 – 7.34 (m, 3H), 7.34 – 7.29 (m, 2H), 4.19 (d, *J* = 5.5 Hz, 1H), 2.89 (dd, *J* = 9.8, 5.5 Hz, 1H), 2.02 (tdt, *J* = 11.2, 9.7, 3.4 Hz, 1H), 1.96 – 1.89 (m, 1H), 1.85 (dtd, *J* = 13.1, 3.5, 1.8 Hz, 1H), 1.67 (dddt, *J* = 15.8, 12.1, 4.3, 2.2 Hz, 2H), 1.51 – 1.31 (m, 2H), 1.30 – 0.99 (m, 3H), 0.90 – 0.75 (m, 1H).

^{13}C NMR (101 MHz, $CDCl_3$) δ/ppm = 136.8, 129.2, 128.8, 128.4, 112.4, 112.1, 52.4, 39.3, 31.3, 30.7, 27.2, 25.9, 25.9, 25.8.

Spectral data are in accordance with the literature.[6]

Dimethyl 2-benzylsuccinate (3f)

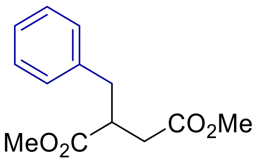

3f

Synthesized following GP 2 with ethyl 6-benzyl-2-(dimethylamino)-4-methyl-1,6-dihydropyrimidine-5-carboxylate **1r** (301 mg, 0.75 mmol, 1.5 equiv.) and dimethyl fumarate (72.1 mg, 0.5 mmol, 1.0 equiv.). Purification by column chromatography on silica gel (*n*-pentane/EtOAc) afforded the product as a colorless solid (66 mg, 0.277 mmol, 55%).

R*f* (*n*-pentane/EtOAc = 5/1): 0.65.

^{1}H NMR (400 MHz, $CDCl_3$) δ/ppm = 7.31 – 7.26 (m, 2H), 7.24 – 7.19 (m, 1H), 7.17 – 7.13 (m, 2H), 3.67 (s, 3H), 3.64 (s, 3H), 3.19 – 3.09 (m, 1H), 3.05 (dd, *J* = 13.5, 6.3 Hz, 1H), 2.76 (dd, *J* = 13.5, 8.3 Hz, 1H), 2.68 (dd, *J* = 16.8, 9.2 Hz, 1H), 2.41 (dd, *J* = 16.8, 4.9 Hz, 1H).

^{13}C NMR (101 MHz, $CDCl_3$) δ/ppm = 174.8, 172.4, 138.3, 129.1, 128.7, 126.9, 52.1, 51.9, 43.1, 37.9, 35.0.

Spectral data are in accordance with the literature.[10]

Dimethyl 2-cyclopentylsuccinate (3g)

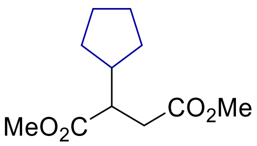

3g

Synthesized following GP 2 with ethyl 6-cyclohexyl-2-(dimethylamino)-4-methyl-1,6-dihydropyrimidine-5-carboxylate **1a** (220 mg, 0.75 mmol, 1.5 equiv.) and dimethyl fumarate (72.1 mg, 0.5 mmol, 1.0 equiv.). Purification by column chromatography on silica gel (*n*-pentane/EtOAc) afforded the product as a colorless oil (69 mg, 0.322 mmol, 64%).

R_f (*n*-pentane/EtOAc = 5/1): 0.65.

^{1}H NMR (400 MHz, $CDCl_3$) δ/ppm = 3.69 (s, 3H), 3.65 (s, 3H), 2.74 (dd, *J* = 15.7, 10.7 Hz, 1H), 2.70 – 2.63 (m, 1H), 2.53 – 2.46 (m, 1H), 2.03 – 1.89 (m, 1H), 1.81 – 1.72 (m, 1H), 1.72 – 1.45 (m, 5H), 1.33 – 1.22 (m, 1H), 1.22 – 1.11 (m, 1H).

^{13}C NMR (101 MHz, $CDCl_3$) δ/ppm = 175.5, 172.8, 51.9, 51.8, 46.3, 42.5, 35.5, 30.6, 30.6, 25.1, 25.0.

Spectral data are in accordance with the literature.[11]

Optimization of Ni/photoredox dual catalyzed alkylation of haloarenes

Optimization for *p*-iodoanisole as coupling partner

To an oven-dried screw-capped Schlenk tube equipped with a magnetic stir bar was added *p*-iodoanisole (23.4 mg, 0.1 mmol, 1.0 equiv.), dihydropyrimidine **1a** (58.7 mg, 0.2 mmol, 2.0 equiv.), 4CzIPN (0.8 mg, 0.001 mmol, 1 mol%), $NiCl_2$dme (2.2 mg, 0.01 mmol, 10 mol%), dtbbpy (4.0 mg, 0.015 mmol, 15 mol%), DMAP (18.3 mg, 0.15 mmol, 1.5 equiv.). After evacuating and refilling with argon at least three cycles, dry MeCN (1 mL) was added and the reaction is degassed for 5 min. The reaction then was allowed to stir in photoreactor with wavelength 450 nm for 24 h. Reactions for optimization were tested on a 0.1 mmol scale and the conversion to the desired product was determined via GC adding mesitylene as an internal standard. GC was calibrated using a five-point calibration of *p*-iodoanisole and **5a**, respectively.

Table S1. Base Screening

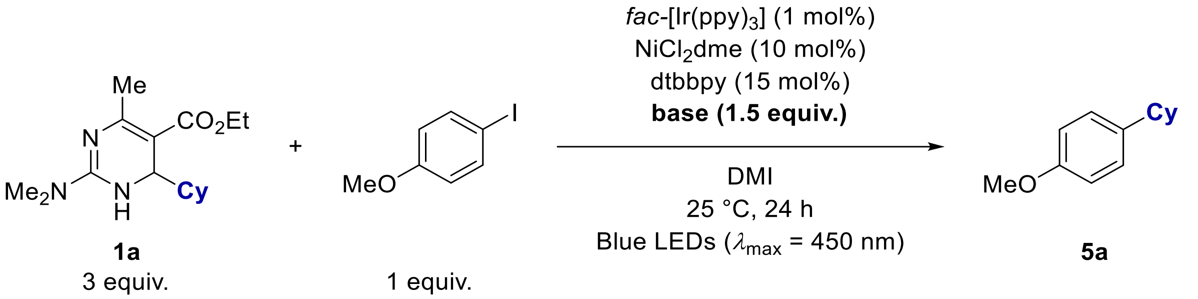

Entry	Base	GC conversion of *p*-iodoanisole / %	GC yield / %
1	No Variation	>95	9
2	K_3PO_4	>95	25
3	KH_2PO_4	>95	20
4	K_2CO_3	>95	42
5	Na_2SO_4	>95	26
6	Na_2CO_3	>95	27
7	K_2HPO_4	55	19
8	KOAc	>95	9
9	LiOH • H_2O	>95	13
10	Li_2CO_3	65	31
**11**	**DMAP**	>95	**82**
12	$Ba(OH)_2$	>95	3
13	DBU	>95	53

14	KOH	>95	31
15	$Ca(OH)_2$	>95	24

Table S2. Solvent screening with photocatalyst *fac*-[$Ir(ppy)_3$]

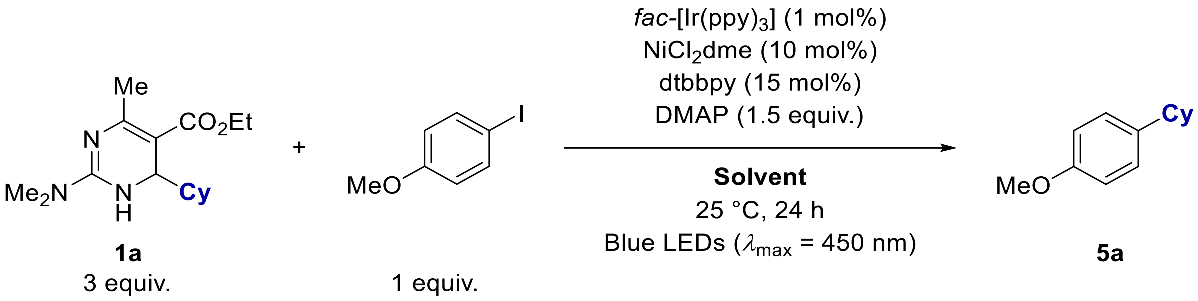

Entry	Solvent	GC conversion of *p*-iodoanisole / %	GC yield / %
**1**	**MeCN**	>95	**>95**
2	DMF	>95	94
3	DMSO	>95	95
4	DMA	>95	93
5	THF	>95	85
6	Acetone	>95	>95
7	MeOH	>95	84
8	Toluene	>95	92
9	1,4-dioxane	>95	92
10	DMI	>95	83

Table S3. Photocatalyst screening

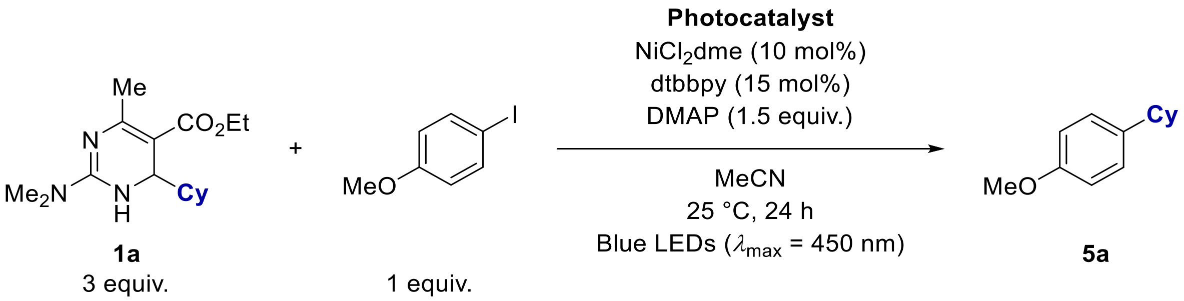

Entry	Photocatalyst	GC conversion of *p*-iodoanisole / %	GC yield / %
**1**	No photocatalyst	60	45
2	$Ir(dF(CF_3)ppy)_2dtbbpy\}PF_6$	>95	>95
3	$Ru(bpy)_3(PF_6)_2$	>95	>95
4	Rose bengal	>95	55

5	Riboflavin	12	2
6	Rhodamin 6G	>95	83
7	Eosin B	25	10
8	Eosin Y	>95	31
**9**	**4CzIPN**	>95	**>95**
10	Mes-Acr$^+$	>95	67

Table S4. Reaction time screening

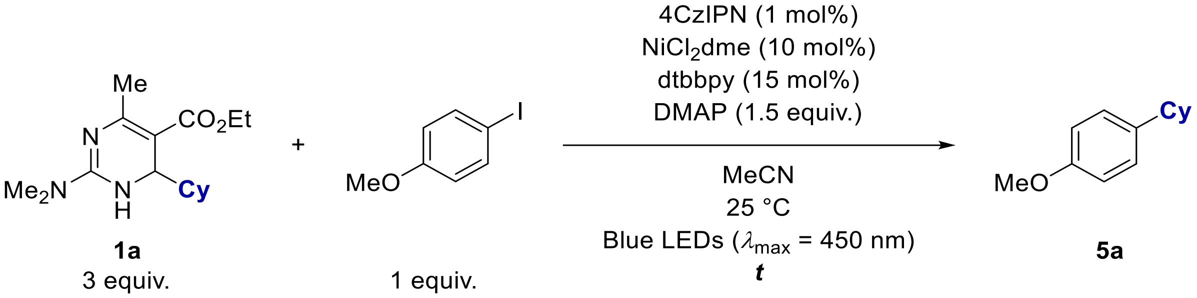

Entry	t / h	GC conversion of *p*-iodoanisole / %	GC yield / %
**1**	**2**	>95	**78**
2	4	>95	90
3	23	>95	95
4	24	>95	95

Table S5. Solvent screening with photocatalyst 4CzIPN

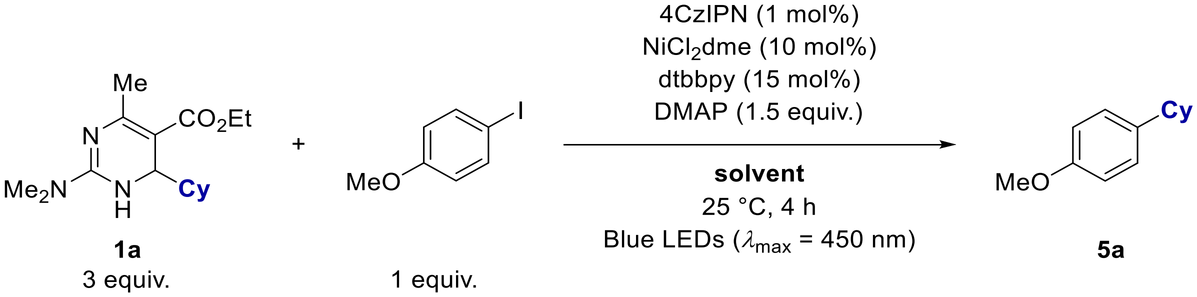

Entry	Solvent	GC conversion of *p*-iodoanisole / %	GC yield / %
**1**	**MeCN**	>95	**>95**
2	DMF	>95	73
3	Acetone	>95	56
4	DMA	>95	93

5	THF	>95	80
6	EtOH	>95	87
7	iPrOH	>95	71
8	Et_2O	>95	67
9	DMSO	>95	>95
10	2-MeTHF	>95	>95
11	1,4-dioxane	>95	>95
12	MeOH	>95	76

Table S6. Dihydropyrimidine Loading Optimization

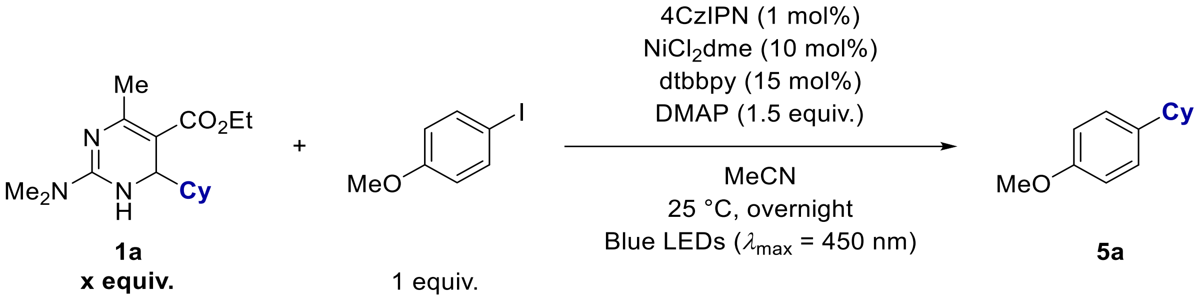

Entry	Dihydropyrimidine / equiv.	GC conversion of *p*-iodoanisole / %	GC yield / %
1	1.0	>95	49
2	1.2	>95	59
3	1.4	>95	71
4	1.6	>95	78
5	1.8	>95	90
**6**	**2.0**	>95	**89**
7	2.2	>95	89
8	2.4	>95	92
9	2.6	>95	92
10	2.8	>95	>95
11	3.0	>95	>95

Table S7. Control experiments with UV-A LEDs (λ_{max} = 365 nm)

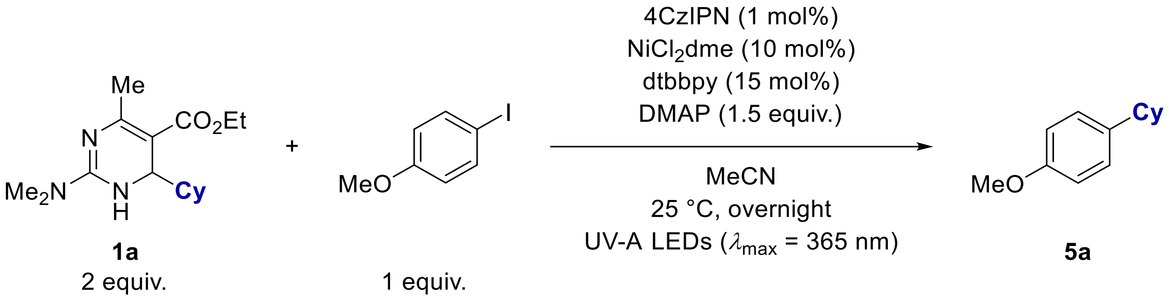

Entry	Variation	GC conversion of *p*-iodoanisole / %	GC yield / %
1	No variation	>95	86
2	No 4CzIPN	>95	30
3	No light	0	0

Table S8. Radical scavenger screening

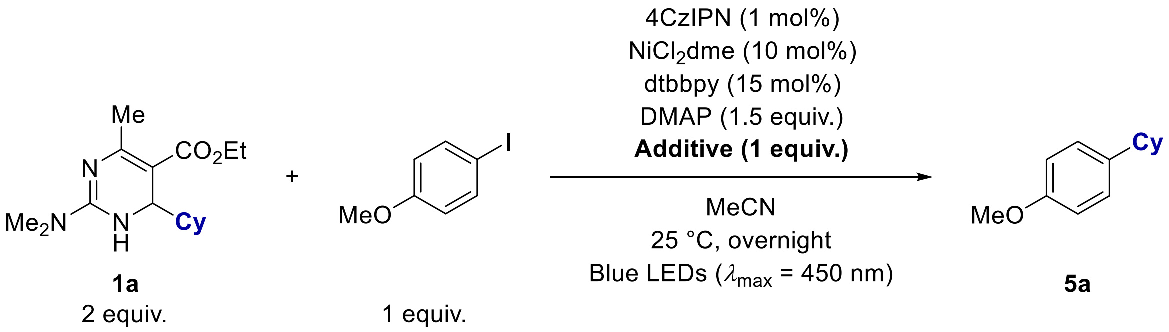

Entry	Additive	GC conversion of *p*-iodoanisole / %	GC yield / %
1*	TEMPO	29	1
2	BHT	>95	69
3	Galvinoxyl	31	0
4	Under air	25	2
5	H_2O (5.0 equiv.)	91	75
6	Without DMAP	35	11

*The cyclohexyl TEMPO adduct was identified by HRMS and GCMS.

Table S9. Nickel catalyst screening

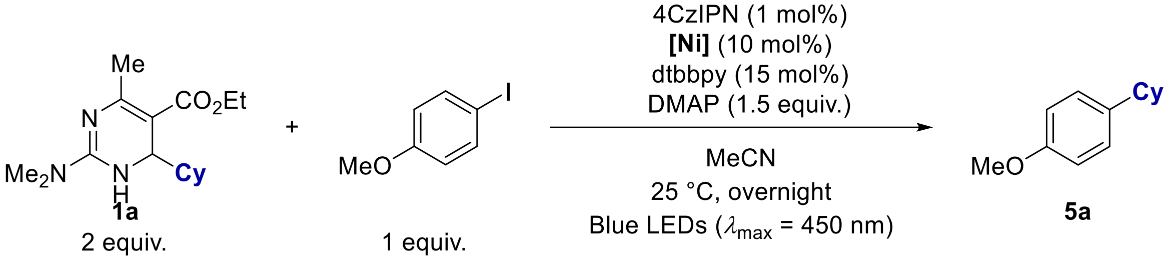

Entry	[Ni]	GC conversion of *p*-iodoanisole / %	GC yield / %
1	$NiCl_2dme$	>95	**89**
2	$NiBr_2dme$	>95	88

Table S10. Transfer reagent screening

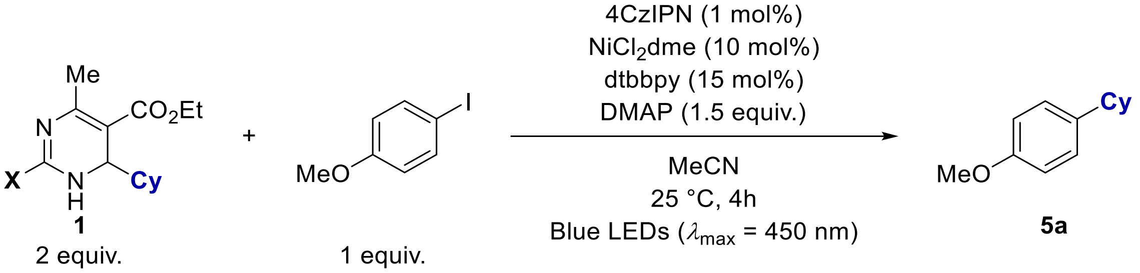

Entry	X =	GC conversion of *p*-iodoanisole / %	GC yield / %
1	**1a**	>95	77
2	**1b** instead of **1a**	>95	10
3	**1c** instead of **1a**	>95	20
4	**1d** instead of **1a**	>95	>95
7	**1e** instead of **1a**	45	7
10	**1g** instead of **1a**	32	2

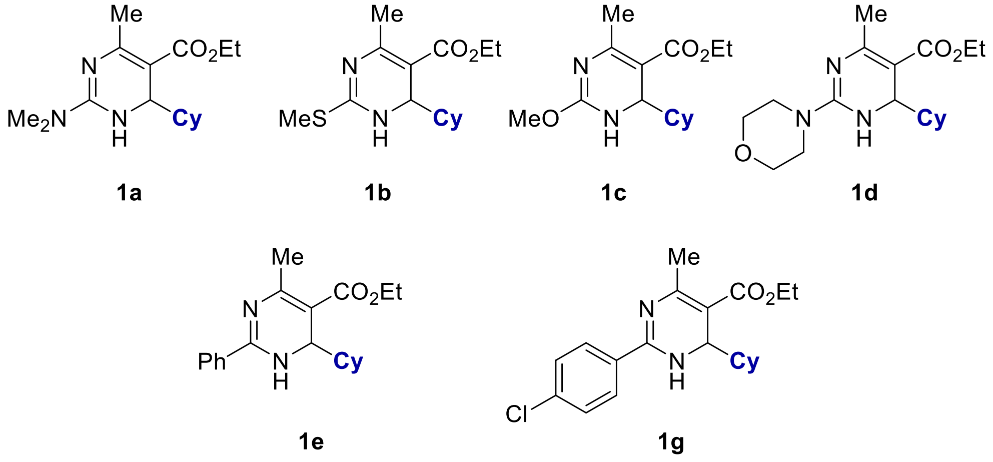

1a **1b** **1c** **1d** **1e** **1g**

Optimization of photocatalyst-free Ni/photoredox dual catalyzed alkylation of haloarenes

Table S11. Wavelength screening

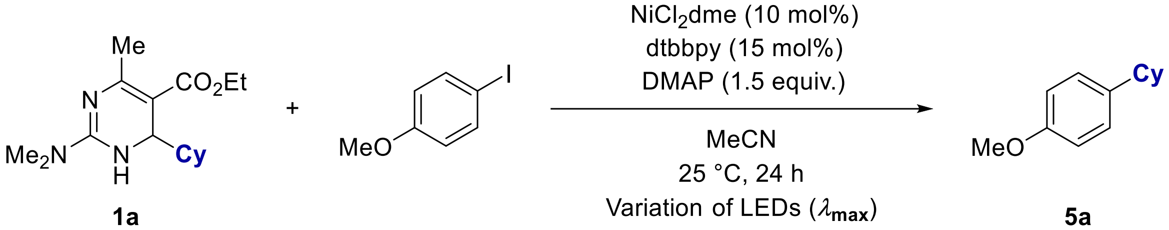

1a 3 equiv. | 1 equiv. | **5a**

Entry	Different wavelength	GC conversion of *p*-iodoanisole / %	GC yield / %
1	450 nm	49	28
2	390 nm	71	65
3	365 nm	32	28
4	390 nm, $NiBr_2dme$	94	**81**
5	405 nm, $NiBr_2dme$	87	61
6	6200K, $NiBr_2dme$	90	2
7	425 nm, $NiBr_2dme$	72	46

Table S12. Different variations

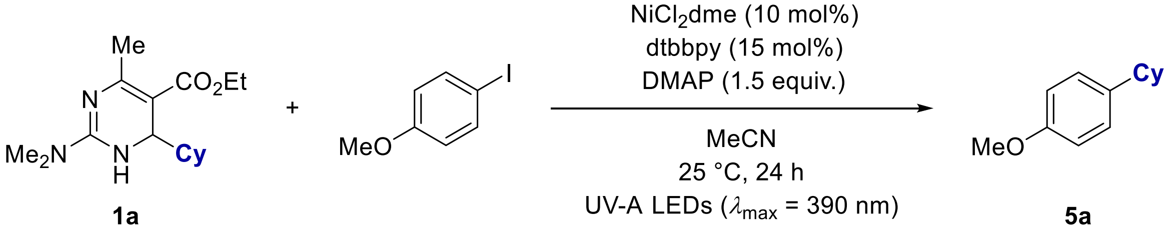

1a 3 equiv. | 1 equiv. | **5a**

Entry	Variations	GC conversion of *p*-iodoanisole / %	GC yield / %
1	No deviation	71	65
2	$NiBr_2dme$	91	**84**
3	Lutidine instead of DMAP	71	29
4	$NiBr_2dme$, without DMAP	35	29
5	$NiBr_2dme$, under air	37	29
6*	$NiBr_2dme$, with TEMPO (1 equiv.)	5	0
7	$NiBr_2dme$, with H_2O (5 equiv.)	>95	75

*The cyclohexyl TEMPO adduct was identified by HRMS.

Table S13. Reagent screening

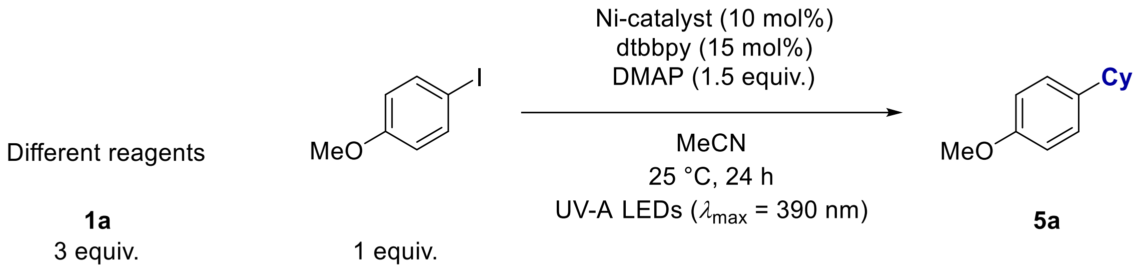

Entry	Reagents	Ni catalyst	GC conversion of *p*-iodoanisole / %	GC yield / %
1	**1a**	$NiCl_2dme$	71	65
2	**1a**	$NiBr_2dme$	94	**81**
3	**1b** instead of **1a**	$NiBr_2dme$	2	0
4	**1c** instead of **1a**	$NiBr_2dme$	11	0
5	**1d** instead of **1a**	$NiBr_2dme$	79	62
6	**1e** instead of **1a**	$NiBr_2dme$	1	0
7	**Cy-DHP** instead of **1a**	$NiCl_2dme$	4	4
8	**Cy-DHQz** instead of **1a**	$NiCl_2dme$	12	0

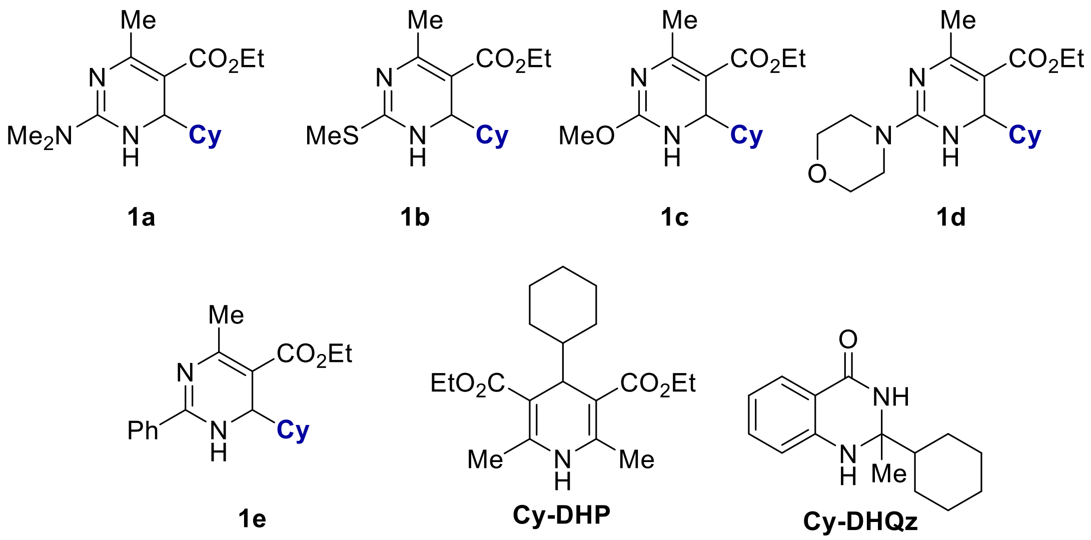

Table S14. Solvent screening

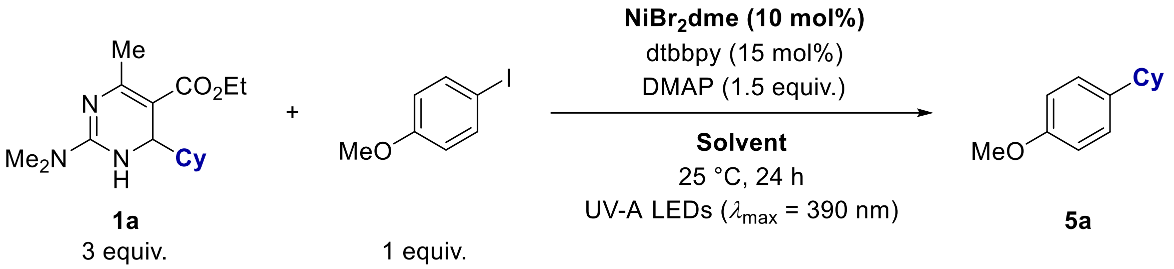

Entry	Solvent	GC conversion of *p*-iodoanisole / %	GC yield / %
1	MeCN	91	**80**
2	THF	46	26
3	Acetone	0	0
4	EtOH	62	60
5	PhMe	36	23
6	DCM	0	0
7	MeOH	75	69
8	DMSO	68	56
9	DMF	55	41
10	EtOAc	35	22

Table S15. Ni-complex screening

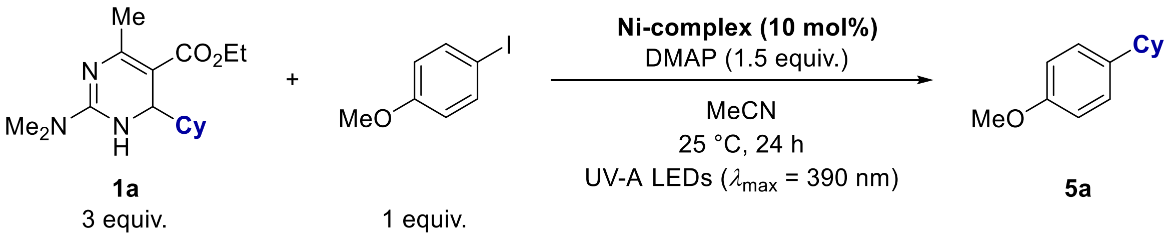

Entry	Ni-complex	GC conversion of *p*-iodoanisole / %	GC yield / %
1	$NiBr_2$dme (10 mol%), dtbbpy (15 mol%),	>95	**88**
2	$NiBr_2$dme (20 mol%), dtbbpy (30 mol%)	84	74
3	$NiCl_2$(dtbbpy)	60	45
4	$NiBr_2$(dtbbpy)	67	44
5	$Ni(bpy)_3$ (BF_4)	88	75

Optimization for *p*-bromoanisole as coupling partner

To an oven-dried screw-capped Schlenk tube equipped with a magnetic stir bar was added *p*-bromoanisole (12.5 µL, 0.1 mmol, 1.0 equiv.), dihydropyrimidine **1a** (88.0 mg, 0.3 mmol, 3.0 equiv.), 4CzIPN (0.8 mg, 0.001 mmol, 1 mol%), $NiCl_2dme$ (2.2 mg, 0.01 mmol, 10 mol%), dtbbpy (4.0 mg, 0.015 mmol, 15 mol%), 2,6-lutidine (17.4 µL, 0.15 mmol, 1.5 equiv.). After evacuating and refilling with argon at least three cycles, dry MeCN (1 mL) was added and the reaction is degassed for 5 min. The reaction then was allowed to stir in photoreactor with wavelength 450 nm for 24 h. Reactions for optimization were tested on a 0.1 mmol scale and the conversion to the desired product was determined via GC adding mesitylene as an internal standard. GC was calibrated using a five-point calibration of *p*-bromoanisole and **5a**, respectively.

Table S16. Photocatalyst screening

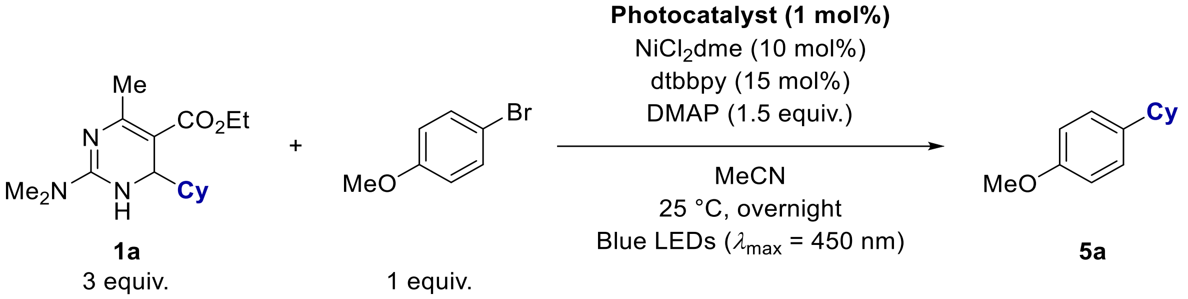

Entry	Photocatalyst	Conversion of *p*-bromoanisole / %	GC yield / %
1	4CzIPN	>95	62
2	*fac*-[Ir(ppy)$_3$]	>95	59
3	Ir(dF(CF$_3$)ppy)$_2$dtbbpy}PF$_6$	>95	65
4	Ru(bpy)$_3$(PF$_6$)$_2$	>95	30
5	Rhodamin 6G	44	8
6	Rose bengal	29	1
7	Eosin B	8	2
8	Eosin Y	34	4

Table S17. Solvent screening

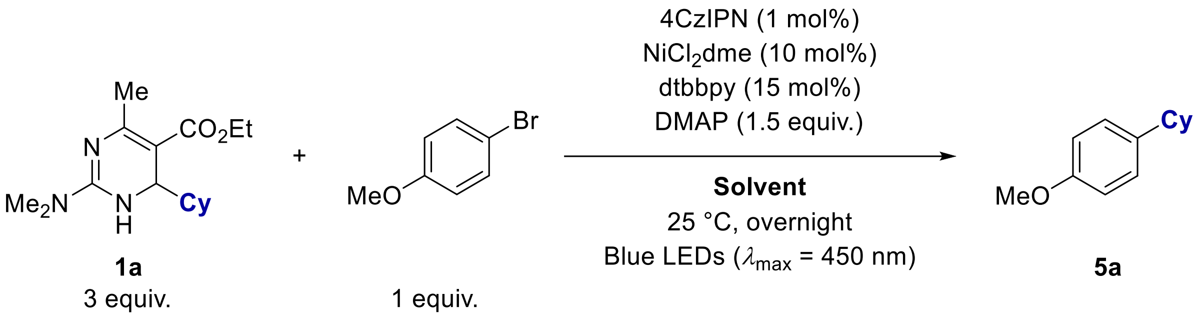

Entry	Solvent	Conversion of *p*-bromoanisole / %	GC yield / %
1	MeCN	>95	62
2	Acetone	>95	71
3	THF	>95	56
4	DMF	>95	86
5	DMI	>95	88

Table S18. Base screening

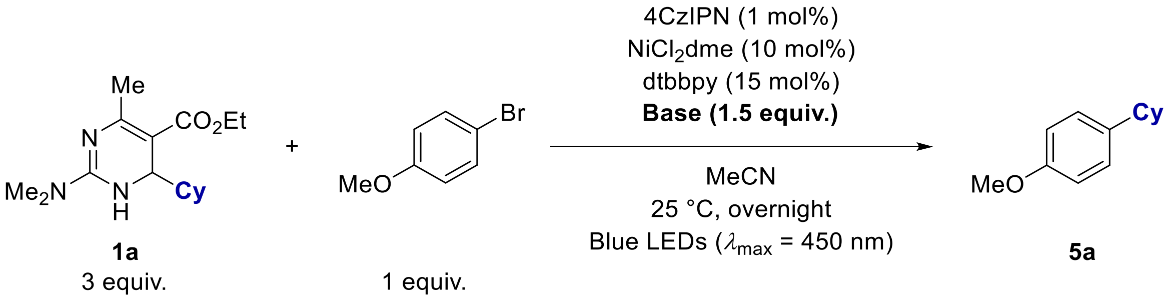

Entry	Base	Conversion of *p*-bromoanisole / %	GC yield / %
1	DMAP	>95	45
**2**	**2,6-Lutidine**	>95	**87**
3	Cs_2CO_3	>95	0
4	Pyridine	>95	77

Table S19. Dihydropyrimidine Screening

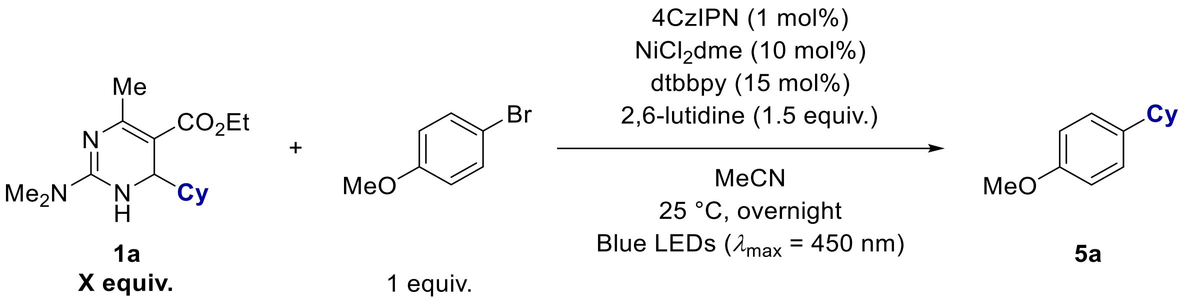

Entry	Dihydropyrimidine / equiv.	Conversion of *p*-bromoanisole / %	GC yield / %
1	1.0	52	47
2	1.2	78	56
3	1.4	82	60
4	1.6	92	67
5	1.8	92	77
6	2.0	>95	84
7	2.2	>95	85
8	2.4	>95	>95
9	2.6	>95	86
10	2.8	>95	>95
11	3.0	>95	>95

Table S20. Radical scavenger screening

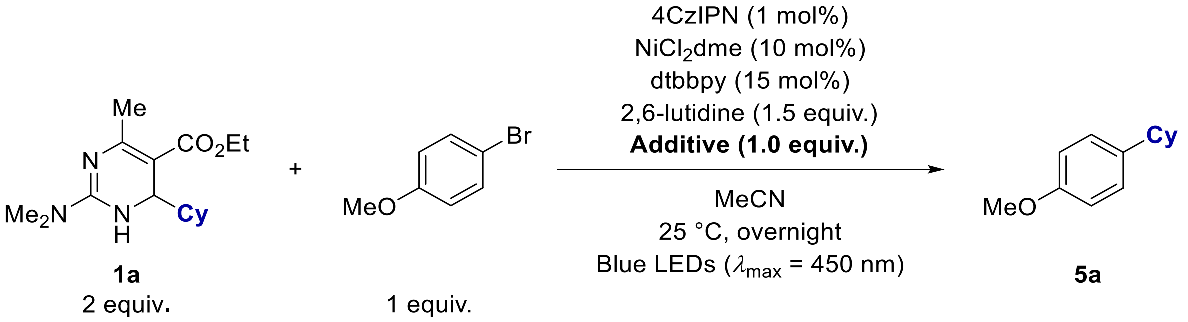

Entry	Additive	Conversion of *p*-bromoanisole / %	GC yield / %
1	TEMPO	41	0
2	BHT	74	9
3	Galvinoxyl	>95	0

Optimization for bromoaniline as coupling partner

To an oven-dried screw-capped Schlenk tube equipped with a magnetic stir bar was added bromoaniline (0.1 mmol, 1.0 equiv.), dihydropyrimidine (1.5 mmol, 3.0 equiv.), *fac*-Ir(ppy)$_3$ (3.2 mg, 0.005 mmol, 1 mol%), $NiCl_2$dme (22.0 mg, 0.1 mmol, 20 mol%), dtbbpy (40.2 mg, 0.15 mmol, 30 mol%), DMAP(91.6 mg, 0.75 mmol, 1.5 equiv.). After evacuating and refilling with argon at least three cycles, dry MeCN (5 mL) was added and the reaction is degassed for 5 min. The reaction then was allowed to stir in photoreactor with wavelength 450 nm for 24 h. Reactions for optimization were tested on a 0.1 mmol scale and the conversion to the desired product was determined via GC adding mesitylene as an internal standard. GC was calibrated using a five-point calibration of bromoaniline and **5s**, respectively.

Table S21. Solvent screening

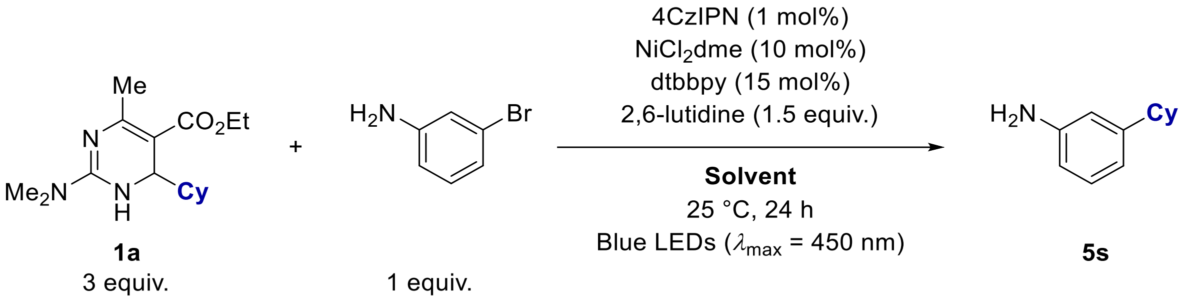

Entry	Solvent	Conversion of *m*-bromoaniline / %	GC yield / %
1	MeCN	>95	24
2	Acetone	>95	0
3	THF	>95	4
4	DMF	>95	7

Table S22. Photocatalyst screening

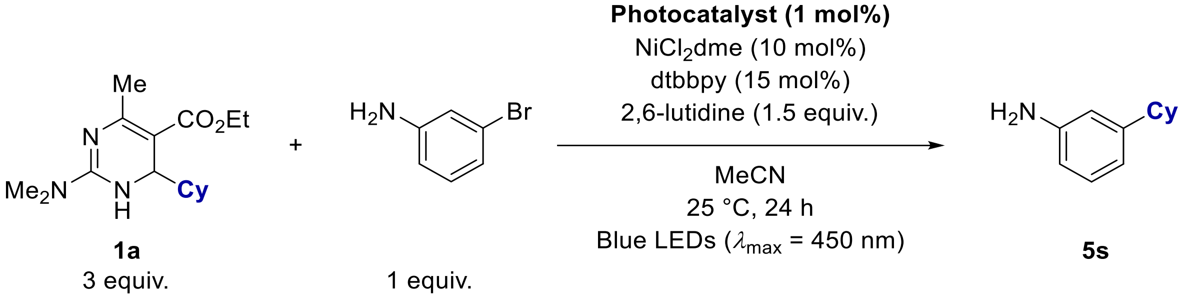

Entry	Photocatalyst	Conversion of *m*-bromoaniline / %	GC yield / %
1	4CzIPN	>95	24
2	*fac*-[Ir(ppy)$_3$]	>95	27
3	[Ir{dF(CF$_3$)ppy}$_2$(dtbpy)]PF$_6$	>95	12
4	Ru(bpy)$_3$(PF$_6$)$_2$	>95	23
5	Rose bengal	>95	20
6	Rhodamin 6G	>95	16

Table S23. Catalyst and ligand loading

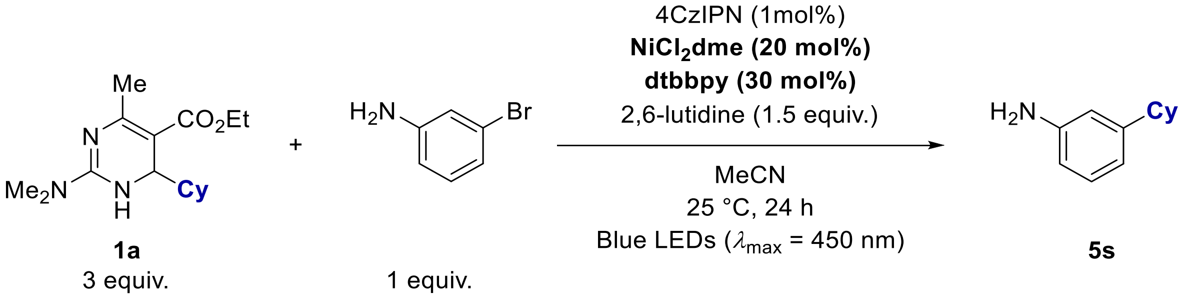

Entry	Photocatalyst	Conversion of *m*-bromoaniline / %	GC yield / %
1	4CzIPN	>95	27
2	*fac*-[Ir(ppy)$_3$]	>95	37

Table S24. Base screening

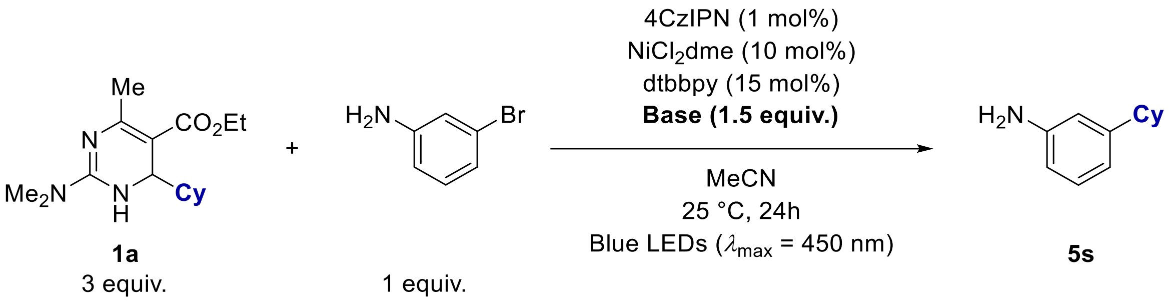

Entry	Base	Conversion of *m*-bromoaniline / %	GC yield / %
1	DBU	>95	2
2	2,6-lutidine	>95	24
3	DIPEA	>95	5

Table S25. Photocatalyst Screening

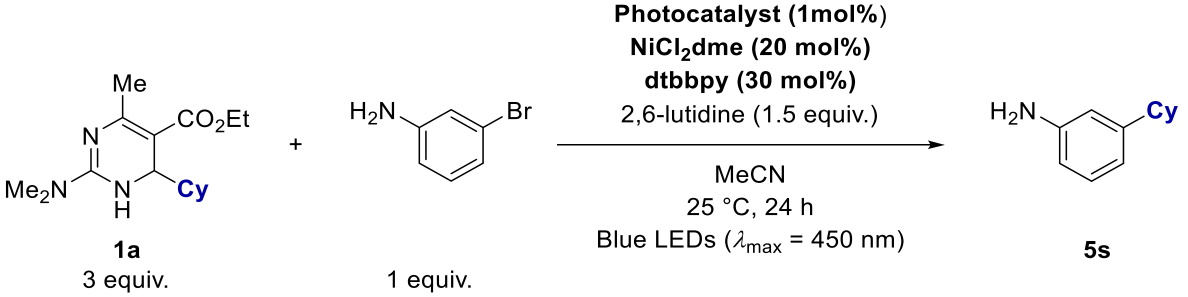

Entry	Photocatalyst	Conversion of *m*-bromoaniline / %	GC yield / %
1	4CzIPN	>95	20
2	*fac*-[Ir(ppy)$_3$]	>95	37
3	*fac*-[Ir(ppy)$_3$] + DMAP	>95	54

Table S26. Base screening

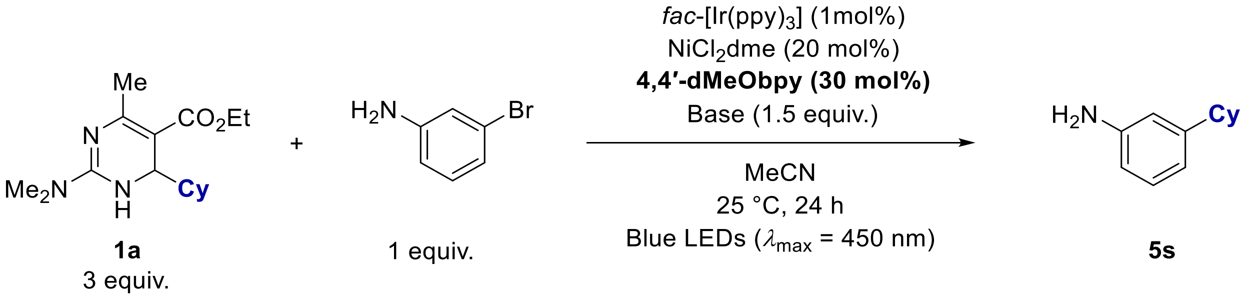

Entry	Base	Conversion of *m*-bromoaniline / %	GC yield / %
1	2,6-lutidine	>95	41

2	2,4,6-collidine	>95	32
3	2,6-lutidine (3 equiv.)	>95	42
4	2,4,6-collidine (3 equiv.)	>95	42
5	2,6-lutidine, 48 h	>95	32

Table S27. Comparison of Dihydropyrimidines to Hantzsch ester in separate experiments

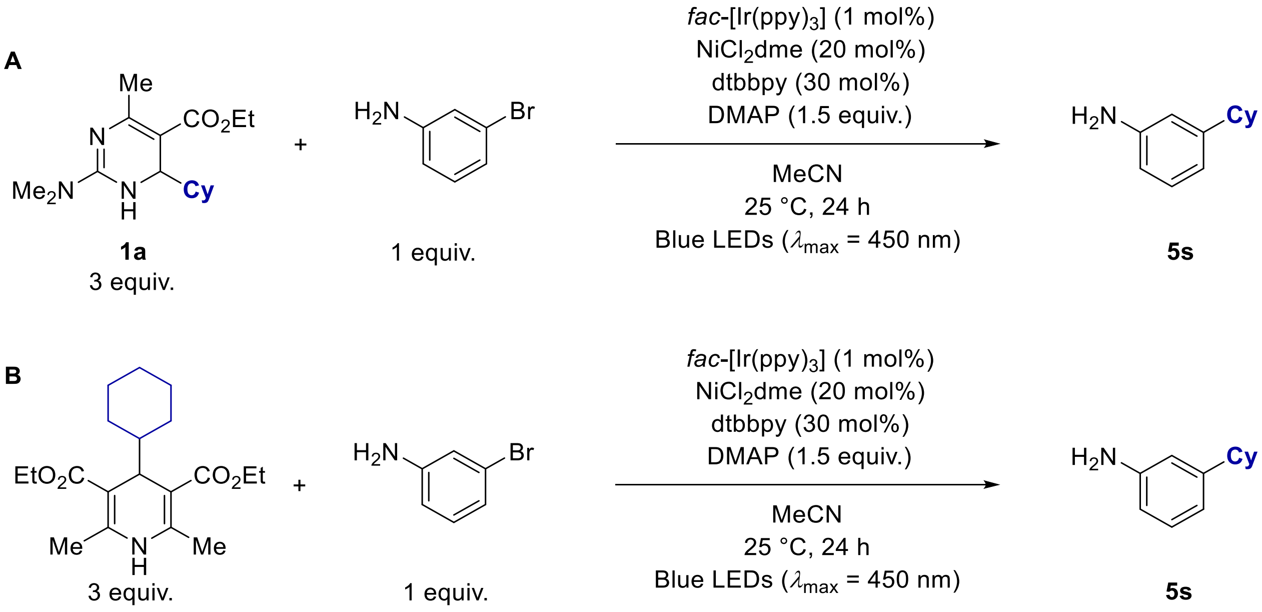

Entry	Variation	Conversion of *m*-bromoaniline / %	GC yield / %
1	No variation (**A**)	>95	54
2	Hantzsch Ester (**B**)	>95	28

Dual Ni/PC-catalyzed coupling with iodobenzenes: General procedure 3a (GP 3a)

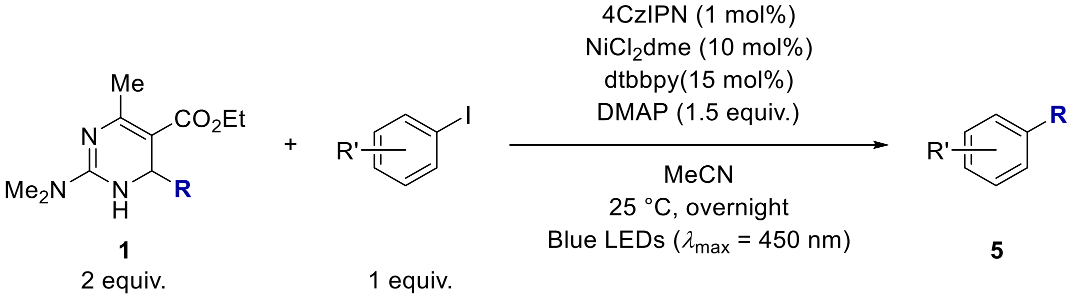

To an oven-dried screw-capped Schlenk tube equipped with a magnetic stir bar was added iodobenzene derivative (0.5 mmol, 1.0 equiv.), dihydropyrimidine (1 mmol, 2.0 equiv.), 4CzIPN (3.9 mg, 0.005 mmol, 1 mol%), $NiCl_2$·dme (11.0 mg, 0.05 mmol, 10 mol%), dtbbpy (20.1 mg, 0.08 mmol, 15 mol%), DMAP (91.6 mg, 0.75 mmol, 1.5 equiv.). After evacuating and refilling with argon at least three cycles, dry MeCN (5 mL) was added and the reaction is degassed for 5 min. The reaction then was allowed to stir in photoreactor with wavelength 450 nm for 24 h. The sample was completely transferred in a round-bottom flask, concentrated *in vacuo* and absorbed on silica gel for subsequent column chromatography.

Alternative work-up: Pyrimidine byproducts can be completely removed by quenching of the crude mixture with 25 mL HCl (1M) and extraction with pentane (3 x 25 mL) prior to column chromatography or crude-NMR.

Dual Ni/PC-catalyzed coupling with Bromobenzenes: General procedure 3b (GP 3b)

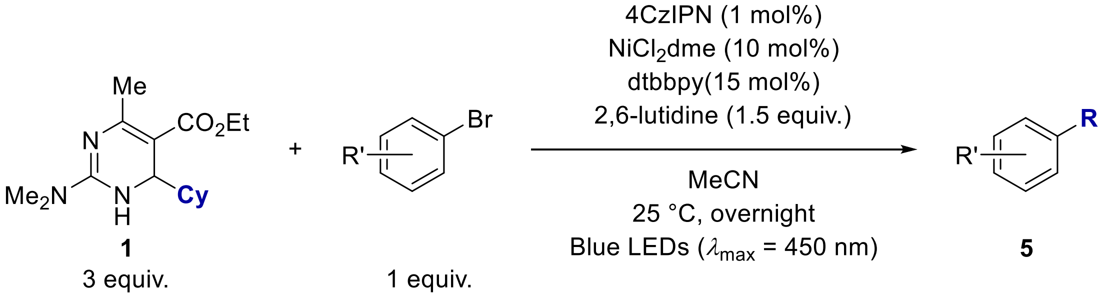

To an oven-dried screw-capped Schlenk tube equipped with a magnetic stir bar was added bromobenzene derivative (0.5 mmol, 1.0 equiv.), dihydropyrimidine (1.5 mmol, 3.0 equiv.), 4CzIPN (3.9 mg, 0.005 mmol, 1 mol%), $NiCl_2$·dme (11.0 mg, 0.05 mmol, 10 mol%), dtbbpy (20.1 mg, 0.08 mmol, 15 mol%), 2,6-lutidine (86.9 µL, 0.75 mmol, 1.5 equiv.). After evacuating and refilling with argon at least three cycles, dry MeCN (5 mL) was added and the reaction is degassed for 5 min. The reaction then was allowed to stir in photoreactor with wavelength 450

nm for 24 h. The sample was completely transferred in a round-bottom flask, concentrated *in vacuo* and absorbed on silica gel for subsequent column chromatography.

Dual Ni/PC-catalyzed coupling with bromoanilines: General procedure 3c (GP 3c)

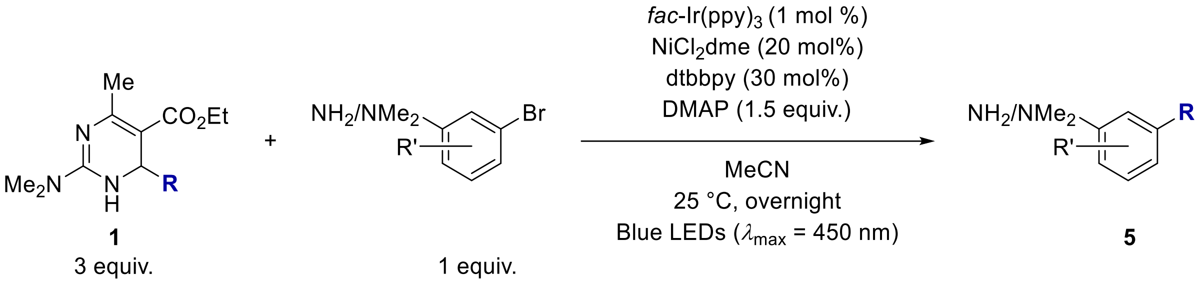

To an oven-dried screw-capped Schlenk tube equipped with a magnetic stir bar was added bromoaniline (0.5 mmol, 1.0 equiv.), dihydropyrimidine (1.5 mmol, 3.0 equiv.), *fac*-Ir(ppy)$_3$ (3.2 mg, 0.005 mmol, 1 mol%), $NiCl_2$·dme (22.0 mg, 0.1 mmol, 20 mol%), dtbbpy (40.2 mg, 0.15 mmol, 30 mol%), DMAP(91.6 mg, 0.75 mmol, 1.5 equiv.). After evacuating and refilling with argon at least three cycles, dry MeCN (5 mL) was added and the reaction is degassed for 5 min. The reaction then was allowed to stir in photoreactor with wavelength 450 nm for 24 h. The sample was completely transferred in a round-bottom flask, concentrated *in vacuo* and absorbed on celite for subsequent column chromatography on silica in *n*-pentane + 2 Vol% of Et_3N/ EtOAc + 2 Vol% of Et_3N.

Photocatalyst-free Ni-catalyzed coupling with iodobenzenes: General procedure 3d (GP 3d)

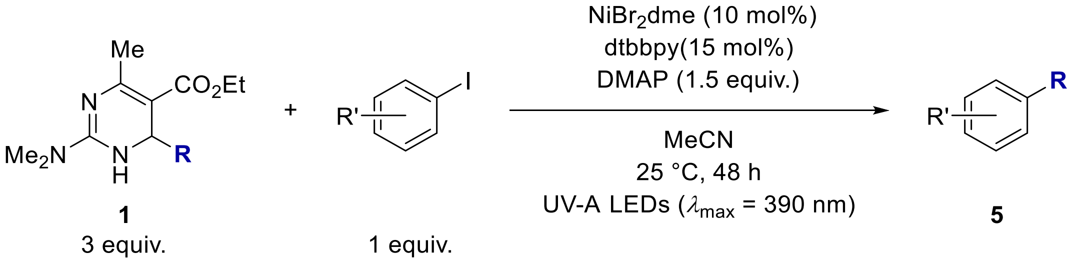

To an oven-dried screw-capped Schlenk tube equipped with a magnetic stir bar was added iodobenzene derivative (0.2 mmol, 1.0 equiv.), dihydropyrimidine (0.6 mmol, 3.0 equiv.), $NiBr_2$·dme (6.2 mg, 0.02 mmol, 10 mol%), dtbbpy (8.1 mg, 0.03 mmol, 15 mol%), DMAP (36.6 mg, 0.30 mmol, 1.5 equiv.). After evacuating and refilling with argon at least three cycles, dry MeCN (2 mL) was added and the reaction is degassed for 5 min. The reaction then was allowed to stir in photoreactor with wavelength 390 nm for 48 h. The sample was completely

transferred to a round-bottom flask, concentrated *in vacuo* and absorbed on silica gel for subsequent column chromatography.

1-Cyclohexyl-4-methoxybenzene (5a)

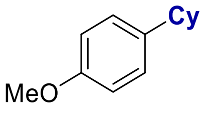

5a

Synthesized following GP 3a with ethyl 6-cyclohexyl-2-(dimethylamino)-4-methyl-1,6-dihydropyrimidine-5-carboxylate **1a** (293.4 mg, 1 mmol, 2.0 equiv.) and 4-iodoanisole (117.0 mg, 0.5 mmol, 1.0 equiv.). Purification by column chromatography on silica gel (*n*-pentane) afforded the product as a colorless solid (91.6 mg, 0.48 mmol, 96%).

Isolated yield using GP3d on 0.2 mmol scale (photocatalyst-free version): 33 mg, 0.173 mmol, 87%

$\mathbf{R_f}$ (*n*-pentane/EtOAc = 10/1): 0.62.

^{1}H NMR (400 MHz, $CDCl_3$) δ/ppm = 7.15 (d, *J* = 8.5 Hz, 2H), 6.84 (d, *J* = 8.6 Hz, 2H), 3.79 (s, 3H), 2.50 – 2.39 (m, 1H), 1.91 – 1.68 (m, 5H), 1.48 – 1.18 (m, 5H).

^{13}C NMR (101 MHz, $CDCl_3$) δ/ppm = 157.7, 140.5, 127.7, 113.7, 55.3, 43.8, 34.8, 27.1, 26.3.

Spectral data are in accordance with the literature.[12]

1-Cyclohexyl-4-(trifluoromethyl)benzene (5b)

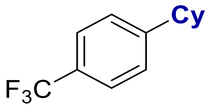

5b

Synthesized following GP 3a with ethyl 6-cyclohexyl-2-(dimethylamino)-4-methyl-1,6-dihydropyrimidine-5-carboxylate **1a** (293.4 mg, 1 mmol, 2.0 equiv.) and 1-iodo-4-(trifluoromethyl)benzene (73.4 µL, 0.5 mmol, 1.0 equiv.). Purification by column chromatography on silica gel (*n*-pentane) afforded the product as a colorless oil (89 mg, 0.39 mmol, 78%).

$\mathbf{R_f}$ (*n*-pentane/EtOAc = 20/1): 0.73.

^{1}H NMR (400 MHz, $CDCl_3$) δ/ppm = 7.54 (d, *J* = 8.0 Hz, 2H), 7.31 (d, *J* = 8.0 Hz, 2H), 2.56 (tt, *J* = 8.8 Hz, *J* = 3.4 Hz, 1H), 1.87 (dt, *J* = 5.3 Hz, *J* = 2.6 Hz, 4H), 1.78 (m, 1H), 1.42 (td, *J* = 10.3 Hz, *J* = 9.4 Hz, *J* = 4.0 Hz, 4H), 1.27 (m, 1H).

^{13}C NMR (101 MHz, $CDCl_3$) δ/ppm = 152.1, 128.2 (q, *J* = 32.3 Hz), 127.2, 125.3 (q, *J* = 3.7 Hz), 124 (q, *J* = 271.7 Hz), 44.6, 34.3, 26.8, 26.1.

^{19}F NMR (75 MHz, $CDCl_3$) δ/ppm = -62.23.

Spectral data are in accordance with the literature.[12]

1-Cyclohexyl-4-methylbenzene (5c)

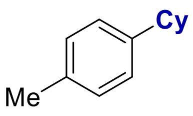

5c

Synthesized following GP 3a with ethyl 6-cyclohexyl-2-(dimethylamino)-4-methyl-1,6-dihydropyrimidine-5-carboxylate **1a** (293.4 mg, 1 mmol, 2.0 equiv.) and 1-iodo-4-methylbenzene (64.9 µL, 0.5 mmol, 1.0 equiv.). Purification by column chromatography on silica gel (*n*-pentane) afforded the product as a colorless oil (61.5 mg, 0.35 mmol, 70%).

Isolated yield using GP3d on 0.2 mmol scale (photocatalyst-free version): 16 mg, 0.091 mmol, 46%

R_f (*n*-pentane/EtOAc = 20/1): 0.65.

^{1}H NMR (400 MHz, $CDCl_3$) δ/ppm = 7.11 (s, 4H), 2.48 (m, 1H), 2.32 (s, 3H), 1.88 – 1.82 (m, 4H), 1.76 (m, 1H), 1.46 – 1.34 (m, 4H), 1.31 – 1.18 (m, 1H).

^{13}C NMR (101 MHz, $CDCl_3$) δ/ppm = 145.3, 135.3, 129.1, 126.8, 44.3, 34.7, 27.1, 26.3, 21.1.

Spectral data are in accordance with the literature.[13]

4-Cyclohexyl-1,2-dimethoxybenzene (5d)

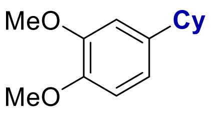

5d

Synthesized following GP 3a with ethyl 6-cyclohexyl-2-(dimethylamino)-4-methyl-1,6-dihydropyrimidine-5-carboxylate **1a** (293.4 mg, 1 mmol, 2.0 equiv.) and 4-iodo-1,2-

dimethoxybenzene (132.0 mg, 0.5 mmol, 1.0 equiv). Purification by column chromatography on silica gel (*n*-pentane) afforded the product as a pale yellow oil (92.1 mg, 0.41 mmol, 83%).

R_f (*n*-pentane/EtOAc = 5/1): 0.45.

^{1}H NMR (400 MHz, $CDCl_3$) δ/ppm = 6.82 – 6.74 (m, 3H), 3.88 (s, 3H), 3.86 (s, 3H), 2.45 (ddt, *J* = 8.3 Hz, *J* = 6.27 Hz, *J* = 3.39 Hz, 1H), 1.86 (m, 4H), 1.75 (ddd, *J* = 12.40 Hz, *J* = 3.09 Hz, *J* = 1.53 Hz, 1H), 1.43 – 1.37 (m, 4H), 1.28 – 1.24 (m, 1H).

^{13}C NMR (101 MHz, $CDCl_3$) δ/ppm = 148.7, 147.0, 141.0, 118.3, 111.1,110.3, 55.9, 55.8, 44.2, 34.7, 26.9, 26.2.

Spectral data are in accordance with the literature.[14]

1-Cyclohexyl-3-methoxybenzene (5e)

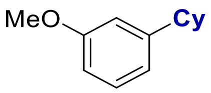

5e

Synthesized following GP 3a with ethyl 6-cyclohexyl-2-(dimethylamino)-4-methyl-1,6-dihydropyrimidine-5-carboxylate **1a** (293.4 mg, 1 mmol, 2.0 equiv.) and 1-iodo-3-methoxybenzene (59.5 µL, 0.5 mmol, 1.0 equiv). Purification by column chromatography on silica gel (*n*-pentane) afforded the product as a pale-yellow oil (92.4 mg, 0.48 mmol, 97%).

R_f (*n*-pentane/EtOAc = 10/1): 0.62.

^{1}H NMR (400 MHz, $CDCl_3$) δ/ppm = 7.21 (t, *J* = 7.86 Hz, 1H), 6.82 – 6.71 (m, 3H), 3.80 (s, 3H), 2.47 (td, *J* = 9.7 Hz, *J* = 8.1 Hz, *J* = 5.8 Hz, 1H), 1.90 – 1.81 (m, 4H), 1.78 – 1.68 (m, 1H), 1.49 – 1.32 (m, 4H), 1.29 – 1.19 (m, 1H)

^{13}C NMR (101 MHz, $CDCl_3$) δ/ppm = 159.7, 150.0, 129.3, 119.4, 112.4, 110.9, 55.2, 44.8, 34.5, 27.0, 26.3.

Spectral data are in accordance with the literature.[15]

1-Cyclohexyl-4-fluorobenzene (5f)

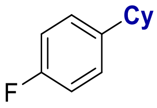

5f

Synthesized following GP 3a with ethyl 6-cyclohexyl-2-(dimethylamino)-4-methyl-1,6-dihydropyrimidine-5-carboxylate **1a** (293.4 mg, 1 mmol, 2.0 equiv.) and 1-fluoro-4-iodobenzene (57.6 µL, 0.5 mmol, 1.0 equiv.). Purification by column chromatography on silica gel (*n*-pentane) afforded the product as a pale-yellow oil (60.7 mg, 0.34 mmol, 68%).

R*f* (*n*-pentane/EtOAc = 20/1): 0.69.

^{1}H NMR (400 MHz, $CDCl_3$) δ/ppm = 7.17 – 7.14 (m, 2H), 6.99 – 6.94 (t, *J* = 8.7 Hz, 2H), 2.48 (tt, *J* = 8.3 Hz, *J* = 3.1 Hz, 1H), 1.86 – 1.83 (m, 4H), 1.76 – 1.72 (m, 1H), 1.45 – 1.32 (m, 4H), 1.29 – 1.17 (m, 1H).

^{13}C NMR (101 MHz, $CDCl_3$) δ/ppm = 161.2 (d, *J* = 242.9 Hz), 143.8 (d, *J* = 3.0 Hz), 128.2 (d, *J* = 7.6 Hz), 115.0 (d, *J* = 20.8 Hz), 43.9, 34.7, 27.0, 26.2.

^{19}F NMR (377 MHz, $CDCl_3$) δ/ppm = -118.0 (m).

Spectral data are in accordance with the literature.[14]

4-Cyclohexylphenol (5g)

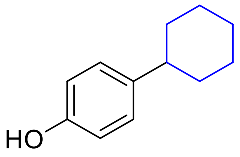

5g

Synthesized following GP 3a with ethyl 6-cyclohexyl-2-(dimethylamino)-4-methyl-1,6-dihydropyrimidine-5-carboxylate **1a** (293.4 mg, 1 mmol, 2.0 equiv.) and 4-iodophenol (110.0 mg, 0.5 mmol, 1 equiv.). Purification by column chromatography on silica gel afforded the product as a white solid (55.5 mg, 0.314 mmol, 62%).

R*f* (*n*-pentane/EtOAc = 2/1): 0.69.

^{1}H NMR (400 MHz, $CDCl_3$) δ 7.08 (d, *J* = 8.1 Hz, 2H), 6.76 (d, *J* = 8.6 Hz, 2H), 4.60 (br s, 1H), 2.49 – 3.80 (m, 1H), 1.90 – 1.78 (m, 4H), 1.77 – 1.69 (m, 1H), 1.41 – 1.18 (m, 5H).

^{13}C NMR (101 MHz, $CDCl_3$) δ 153.5, 140.7, 127.9, 115.1, 43.8, 34.8, 27.0, 26.3

Spectral data are in accordance with the literature.[16]

1-Cyclohexyl-3-(trifluoromethyl)benzene (5h)

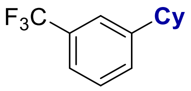

5h

Synthesized following GP 3b with ethyl 6-cyclohexyl-2-(dimethylamino)-4-methyl-1,6-dihydropyrimidine-5-carboxylate **1a** (440.1 mg, 1.5 mmol, 3.0 equiv.) and 1-bromo-3-(trifluoromethyl)benzene (69 µL, 0.5 mmol, 1 equiv.). Purification by column chromatography on silica gel (n-pentane) afforded the product as a colorless oil (93.6 mg, 0.41 mmol, 82%).

$\mathbf{R_f}$ (*n*-pentane/EtOAc = 20/1): 0.84.

^{1}H NMR (400 MHz, $CDCl_3$) δ/ppm = 7.52 – 7.37 (m, 4H), 2.64 – 2.51 (m, 1H), 1.98 – 1.71 (m, 5H), 1.52 – 1.17 (m, 5H).

^{13}C NMR (101 MHz, $CDCl_3$) δ/ppm = 149.0, 130.6 (q, *J* = 31.6 Hz), 130.4, 128.8, 124.2 (q, J = 273.6 Hz), 123.7 (q, *J* = 3.7 Hz) 122.7 (q, *J* = 3.8 Hz),44.6, 34.4, 26.9, 26.1.

^{19}F NMR (75 MHz, $CDCl_3$) δ/ppm = -62.46.

Spectral data are in accordance with the literature.[12]

1-Cyclohexyl-3-fluorobenzene (5i)

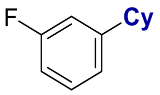

5i

Synthesized following GP 3b with ethyl 6-cyclohexyl-2-(dimethylamino)-4-methyl-1,6-dihydropyrimidine-5-carboxylate **1a** (440.1 mg, 1.5 mmol, 3.0 equiv.) and 1-bromo-3-fluorobenzene (56 µL, 0.5 mmol, 1 equiv.). Purification by column chromatography on silica gel (n-pentane) afforded the product as a pale yellow oil (87.6 mg, 0.49 mmol, 98%).

$\mathbf{R_f}$ (*n*-pentane/EtOAc = 20/1): 0.76.

^{1}H NMR (400 MHz, $CDCl_3$) δ/ppm = 7.24 – 7.21 (m, 1H), 6.98 (dt, *J* = 7.6 Hz, *J* = 1.3 Hz, 1H), 6.94 – 6.82 (m, 2H), 2.50 (m, 1H), 1.94 – 1.80 (m, 4H), 1.78 – 1.70 (m, 1H). 1.46 – 1.26 (m, 5H).

^{13}C NMR (101 MHz, $CDCl_3$) δ/ppm = 163.1 (d, *J* = 244.6 Hz), 150.9 (d, *J* = 6.6 Hz) 129.7(d, *J* = 8.5 Hz), 122.6 (d, *J* = 2.7 Hz), 113.7 (d, *J* = 20.7 Hz), 112.6 (d, *J* = 21.0 Hz), 44.4 (d, *J* = 1.7 Hz), 34.4, 26.9, 26.2.

^{19}F NMR (75 MHz, $CDCl_3$) δ/ppm = -113.91.

Spectral data are in accordance with the literature.[14]

3-Cyclohexylquinoline (5j)

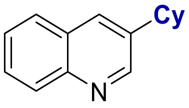

5j

Synthesized following GP 3b with ethyl 6-cyclohexyl-2-(dimethylamino)-4-methyl-1,6-dihydropyrimidine-5-carboxylate **1a** (440.1 mg, 1.5 mmol, 3.0 equiv.) and 3-bromoquinoline (68 µL, 0.5 mmol, 1 equiv.). Purification by column chromatography on silica gel (*n*-pentane) afforded the product as a pale yellow solid (84.7 mg, 0.40 mmol, 80%).

$\mathbf{R_f}$ (*n*-pentane/EtOAc = 5/1): 0.33.

^{1}H NMR (400 MHz, $CDCl_3$) δ/ppm = 8.80 (d, *J* = 2.3 Hz, 1H), 8.06 (dd, *J* = 8.3 Hz, *J* = 1.0 Hz, 1H), 7.88 (d, *J* = 2.2 Hz, 1H), 7.75 (dd, , *J* = 8.1 Hz, *J* = 1.4 Hz, 1H), 7.62 (ddd, *J* = 8.4 Hz, *J* = 6.8 Hz, *J* = 1.4 Hz, 1H), 7.48 (ddd, *J* = 8.0 Hz, *J* = 6.8 Hz, *J* = 1.2 Hz, 1H), 2.72 – 2.64 (m, 1H), 1.98 – 1.83 (m, 4H), 1.82 –1.70 (dd, *J* = 3.1 Hz, *J* = 1.5 Hz, 1H), 1.57–1.37 (m, 4H), 1.28 (dd, *J* = 12.51 Hz, *J* = 3.4 Hz, 1H)

^{13}C NMR (101 MHz, $CDCl_3$) δ/ppm = 151.5, 147.0, 140.3, 132.2, 129.1, 128.5, 128.3, 127.5, 126.4, 42.0, 34.2, 26.8, 26.1.

Spectral data are in accordance with the literature.[17]

Methyl 4-cyclohexylbenzoate (5k)

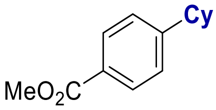

5k

Synthesized following GP 3b with ethyl 6-cyclohexyl-2-(dimethylamino)-4-methyl-1,6-dihydropyrimidine-5-carboxylate **1a** (440.1 mg, 1.5 mmol, 3.0 equiv.) and methyl 4-bromobenzoate (107.5 mg, 0.5 mmol, 1 equiv.). Purification by column chromatography on

silica gel (*n*-pentane) afforded the product as a colorless crystalline solid (105.7 mg, 0.48 mmol, 97%).

R_f (*n*-pentane/EtOAc = 5/1): 0.66.

^{1}H NMR (400 MHz, $CDCl_3$) δ/ppm = 7.96 (m, 2H), 7.27 (m, 2H), 3.90 (s, 3H), 2.59 – 2.49 (m, 1H), 1.93 -1.81 (q, *J* = 3.5 Hz, *J* = 3.0 Hz, 4H), 1.80 – 1.71 (m, 1H), 1.50 – 1.33 (dd, *J* = 6.9 Hz, *J* = 3.6 Hz, 4H), 1.32 – 1.16 (m, 1H).

^{13}C NMR (101 MHz, $CDCl_3$) δ/ppm = 167.3, 153.6, 129.8, 127.8, 127.0, 52.0, 44.8, 34.2, 26.8, 26.3.

Spectral data are in accordance with the literature.[18]

1-Cyclohexyl-3,5-dimethylbenzene (5l)

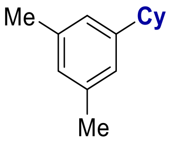

5l

Synthesized following GP 3b with ethyl 6-cyclohexyl-2-(dimethylamino)-4-methyl-1,6-dihydropyrimidine-5-carboxylate **1a** (440.1 mg, 1.5 mmol, 3.0 equiv.) and 1-bromo-3,5-dimethylbenzene (67.9 µL, 0.5 mmol, 1 equiv.). Purification by column chromatography on silica gel (*n*-pentane) afforded the product as a colorless oil (82.8 mg, 0.43 mmol, 87%).

R_f (*n*-pentane/EtOAc = 5/1): 0.91.

^{1}H NMR (400 MHz, $CDCl_3$) δ/ppm = 6.84 (s, 3H), 2.48 – 2.38 (ddd, *J* = 11.6 Hz, *J* = 8.2 Hz, *J* = 3.3 Hz, 1H), 2.30 (s, 6H), 1.91 – 1.79 (m, 4H), 1.76 – 1.69 (m, 1H), 1.48 – 1.31 (m, 4H), 1.31 – 1.17 (m, 1H)

^{13}C NMR (101 MHz, $CDCl_3$) δ/ppm = 148.2, 137.8, 127.6, 124.8, 44.6, 34.6, 27.1, 26.3, 21.5.

Spectral data are in accordance with the literature.[17]

4-Cyclohexylbenzonitrile (5m)

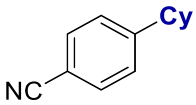

5m

Synthesized following GP 3b with ethyl 2-(dimethylamino)-4-methyl-6-pentyl-1,6-dihydropyrimidine-5-carboxylate (400.7 mg, 1.5 mmol, 3 equiv.) and 4-bromobenzonitrile (51.5 mg, 0.5 mmol, 1 equiv.). Purification by column chromatography on silica gel afforded the product as a yellowish solid (58.9 mg, 0.31 mmol, 63%).

Isolated yield using GP3d on 0.2 mmol scale (photocatalyst-free version): 32.5 mg, 0.175 mmol, 88%

R*f* (*n*-pentane/EtOAc = 5/1): 0.52.

^{1}H NMR (300 MHz, $CDCl_3$) δ/ppm = 7.57 (d, *J* = 8.2 Hz, 2H), 7.29 (d, *J* = 8.2 Hz, 2H), 2.66 – 2.44 (m, 1H), 1.86 (dd, *J* = 7.3 Hz, *J* = 2.0 Hz, 4H),1.81 –1.72 (m, 1H), 1.49 – 1.13 (m, 5H).

^{13}C NMR (101 MHz, $CDCl_3$) δ/ppm = 153.6, 132.3, 127.7, 119.3, 109.6, 44.8, 34.1, 26.7, 26.0

Spectral data are in accordance with the literature.[19]

6-Cyclohexyl-2,3-dihydrobenzo[*b*][1,4]dioxine (5n)

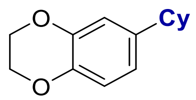

5n

Synthesized following GP 3b with ethyl 6-cyclohexyl-2-(dimethylamino)-4-methyl-1,6-dihydropyrimidine-5-carboxylate **1a** (440.1 mg, 1.5 mmol, 3.0 equiv.) and 6-bromo-2,3-dihydrobenzo[*b*][1,4]dioxine (67.28 µL, 0.5 mmol, 1 equiv.). Purification by column chromatography on silica gel (*n*-pentane) afforded the product as a pale-yellow oil (94 mg, 0.43 mmol, 86%).

R*f* (*n*-pentane/EtOAc = 5/1): 0.62.

^{1}H NMR (300 MHz, $CDCl_3$) δ/ppm = 6.81 (d, *J* = 8.2 Hz, 1H), 6.75– 6.65 (m, 2H), 4.26 (m, 4H), 2.49 – 2.33 (m, 1H), 1.94 – 1.68 (m, 5H), 1.50 – 1.14 (tt, *J* = 8.9 Hz, *J* = 3.0 Hz, 5H).

^{13}C NMR (101 MHz, $CDCl_3$) δ/ppm = 143.3, 141.8, 141.6, 119.8, 117.0, 115.4, 64.5, 64.4, 43.9, 34.7, 27.0, 26.2.

Spectral data are in accordance with the literature.[20]

5-Cyclohexyl-2,2-difluorobenzo[*d*][1,3]dioxole (5o)

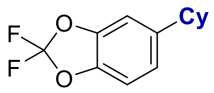

5o

Synthesized following GP 3b with ethyl 6-cyclohexyl-2-(dimethylamino)-4-methyl-1,6-dihydropyrimidine-5-carboxylate **1a** (440.1 mg, 1.5 mmol, 3.0 equiv.) and 5-bromo-2,2-difluorobenzo[*d*][1,3]dioxole (118.5 mg, 0.5 mmol, 1 equiv). Purification by column chromatography on silica gel (*n*-pentane) afforded the product as a pale colorless oil (100.5 mg, 0.41 mmol, 83 %).

R*f* (*n*-pentane/EtOAc = 5/1): 0.83.

^{1}H NMR (400 MHz, $CDCl_3$) δ/ppm = 6.98 – 6.86 (m, 3H), 2.57 – 2.42 (dd, *J* = 6.2 Hz, *J* = 3.3 Hz, 1H), 1.91- 1.68 (m, 5H), 1.48 – 1.16 (m, 5H).

^{13}C NMR (101 MHz, $CDCl_3$) δ/ppm = 144.5, 143.9, 141.9, 131.8 (t, *J* = 253.4 Hz), 121.7, 109.1, 108.1, 44.6, 34.8, 26.9, 26.1.

^{19}F NMR (377 MHz, $CDCl_3$) δ/ppm = -50.06.

Spectral data are in accordance with the literature.[17]

7-Cyclohexyl-1*H*-indole (5p)

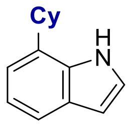

5p

Synthesized following GP 3b with ethyl 6-cyclohexyl-2-(dimethylamino)-4-methyl-1,6-dihydropyrimidine-5-carboxylate **1a** (440.1 mg, 1.5 mmol, 3.0 equiv.) and 7-bromo-1*H*-indole (98.0 mg, 0.5 mmol, 1 equiv.). Purification by column chromatography on silica gel afforded the product as a pale-yellow oil (77 mg, 0.38 mmol, 77%).

R*f* (*n*-pentane/EtOAc = 5/1): 0.58.

^{1}H NMR (400 MHz, $CDCl_3$) δ/ppm = 8.16 (s, 1H), 7.50 (dt, *J* = 7.6 Hz, *J* = 1.4 Hz, 1H), 7.22 – 7.18 (t, *J* = 2.8 Hz, 1H), 7.12- 7.03 (m, 2H), 6.58 – 6.55 (dt, *J* = 3.2, 2.0 Hz, 1H), 2.87 – 2.76 (m, 1H), 2.08 – 1.76 (m, 5H), 1.67 – 1.24 (m, 5H).

^{13}C NMR (101 MHz, $CDCl_3$) δ/ppm = 134.2, 130.5, 127.8, 123.6, 120.2, 118.5, 118.4, 103.2, 40.1, 33.4, 27.2, 26.4.

IR (ATR, cm^{-1}): 3416, 3057, 2924, 2850, 1605, 1587, 1491, 1447, 1430, 1410, 1341, 1280, 1263, 1246, 1202, 1180, 1110, 1068, 976, 887, 839, 794, 734, 722, 623, 595, 559, 478, 458, 448, 405.

HR-MS (ESI): m/z calcd for $[M+H]^+$ 200.1434, found 200.1431.

1-Chloro-4-cyclohexylbenzene (5q)

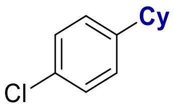

5q

Synthesized following GP 3b with ethyl 6-cyclohexyl-2-(dimethylamino)-4-methyl-1,6-dihydropyrimidine-5-carboxylate **1a** (440.1 mg, 1.5 mmol, 3.0 equiv.) and 1-bromo-4-chlorobenzene (95.7 mg, 0.5 mmol, 1 equiv.). Purification by column chromatography on silica gel (n-pentane) afforded the product as a colorless oil (87.0 mg, 0.44 mmol, 89%).

$\mathbf{R}_f$ (*n*-pentane/EtOAc = 20/1): 0.76.

^{1}H NMR (400 MHz, $CDCl_3$) δ/ppm = 7.27 – 7.24 (m, 1H), 7.23 – 7.22 (d, *J* = 2.0 Hz, 1H), 7.15 – 7.10 (d, *J* = 8.3 Hz, 2H), 2.53 – 2.39 (qd, , *J* = 6.8 Hz, *J* = 6.4 Hz, J = 3.1 Hz, 1H), 1.91 – 1.79 (m, 4H), 1.77 -1.70 (ddd, J = 4.4 Hz, J = 3.1 Hz, J = 1.4 Hz, 1H), 1.45 – 1.31 (m, 4H), 1.29- 1.15 (m, 1H).

^{13}C NMR (101 MHz, $CDCl_3$) δ/ppm = 146.6, 131.4, 128.4, 128.3, 44.1, 34.5, 26.9, 26.2.

Spectral data are in accordance with the literature.[21]

6-Cyclohexyl-2H-benzo[*b*][1,4]oxazin-3(4*H*)-one (5r)

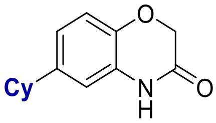

5r

Synthesized following GP 3c with ethyl 6-cyclohexyl-2-(dimethylamino)-4-methyl-1,6-dihydropyrimidine-5-carboxylate (440.1 mg, 1.5 mmol, 3 equiv.) and 6-bromo-2*H*-benzo[*b*][1,4]oxazin-3(4*H*)-one (114.0 mg, 0.5 mmol, 1 equiv.). Purification by column chromatography on silica gel (*n*-pentane + 2 Vol% of Et_3N/ EtOAc + 2 Vol% of Et_3N) afforded the product as a pale yellow crystalline solid (28.3 mg, 0.12 mmol, 24%).

R_f (*n*-pentane/EtOAc = 2/1): 0.48.

^{1}H NMR (400 MHz, $CDCl_3$) δ 8.08 (br s, 1H), 6.89 (d, *J* = 8.3 Hz, 1H), 6.82 (dd, *J* = 8.3 Hz, *J* = 1.9 Hz, 1H), 6.62 (d, *J* = 1.9 Hz, 1H), 4.59 (s, 2H), 2.50 – 2.36 (m, 1H), 1.88 – 1.68 (m, 5H), 1.42 –1.16 (m, 5H).

^{13}C NMR (101 MHz, $CDCl_3$) δ 166.1, 143.2, 141.8, 125.8, 122.5, 116.6, 114.3, 67.5, 44.0, 34.7, 26.9, 26.1

IR (ATR, cm-1): 3198, 3174, 3098, 3043, 2978, 2923, 2913, 2848, 1740, 1687, 1607, 1562, 1522, 1497, 1447, 1405, 1373, 1317, 1302, 1282, 1245, 1228, 1212, 1180, 1124, 1047, 1019, 967, 896, 865, 813, 783, 759, 695, 624, 545, 496, 454, 439, 384

HR-MS (ESI): m/z calcd for $[M+Na]^+$ 254.1156, found 254.1151.

3-Cyclohexylaniline (5s)

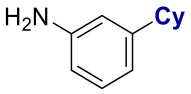

5s

Synthesized following GP 3c with ethyl 6-cyclohexyl-2-(dimethylamino)-4-methyl-1,6-dihydropyrimidine-5-carboxylate **1a** (440.1 mg, 1.5 mmol, 3.0 equiv.) and 3-bromoaniline (54.4 µL, 0.5 mmol, 1 equiv.). Purification by column chromatography on silica gel (*n*-pentane + 2 Vol% of Et_3N/ EtOAc + 2 Vol% of Et_3N) afforded the product as a pale yellow crystalline solid (54.5 mg, 0.3 mmol, 62%).

R_f (*n*-pentane/EtOAc = 2/1): 0.66.

^{1}H NMR (400 MHz, $CDCl_3$) δ/ppm = 7.07 (t, *J* = 7.7 Hz, 1H), 6.64 – 6.60 (dt, *J* = 7.5 Hz, *J* = 1.3 Hz, 1H), 6.56 – 6.53 (t, *J* = 1.9 Hz, 1H), 6.53 – 6.49 (ddd, *J* = 7.8 Hz, *J* = 2.3, *J* =1.0 Hz, 1H), 3.6 (s, 2H), 2.44 – 2.35 (m, 1H), 1.91 –1.69 (m, 5H), 1.45 -1.15 (m, 5H).

^{13}C NMR (101 MHz, $CDCl_3$) δ/ppm = 149.6, 146.4, 129.2, 117.4, 113.9, 112.8, 44.7, 34.5, 27.0, 26.3.

Spectral data are in accordance with the literature.[22]

4-Cyclohexyl-2-methoxyaniline (5t)

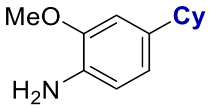

5t

Synthesized following GP 3c with ethyl 6-cyclohexyl-2-(dimethylamino)-4-methyl-1,6-dihydropyrimidine-5-carboxylate **1a** (440.1 mg, 1.5 mmol, 3.0 equiv.) and 4-bromo-2-methoxyaniline (101.0 mg, 0.5 mmol, 1 equiv.). Purification by column chromatography on silica gel (*n*-pentane + 2 Vol% of Et_3N/ EtOAc + 2 Vol% of Et_3N) afforded the product as a pale yellow crystalline solid (52.2 mg, 0.25mmol, 50%).

R*f* (*n*-pentane/EtOAc = 2/1): 0.56.

^{1}H NMR (400 MHz, $CDCl_3$) δ/ppm = 6.68 – 6.61 (m, 3H), 3.85 (s, 3H), 3.65 (br s, 2H), 2.46 – 2.35 (m, 1H), 1.92 – 1.68 (m, 5H), 1.44 –1.16 (m, 5H).

^{13}C NMR (101 MHz, $CDCl_3$) δ/ppm = 147.4, 139.1, 133.9, 118.9, 115.1, 109.5, 55.5, 44.3, 34.9, 30.7, 27.1, 26.3.

IR (ATR, cm^{-1}): 3402, 3302, 3206, 3051, 3012, 2917, 2846, 1634, 1623, 1587, 1518, 1464, 1448, 1425, 1365, 1351, 1280, 1264, 1241, 1221, 1193, 1158, 1128, 1091, 1030, 934, 890, 874, 821, 809, 737, 702, 630, 595, 562, 537, 453, 440, 416, 397

HR-MS (ESI): m/z calcd for $[M+Na]^+$ 228.1359, found 228.1354.

3-Cyclohexyl-4-methylaniline (5u)

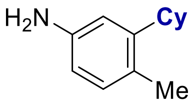

5u

Synthesized following GP 3c with ethyl 6-cyclohexyl-2-(dimethylamino)-4-methyl-1,6-dihydropyrimidine-5-carboxylate **1a** (440.1 mg, 1.5 mmol, 3.0 equiv.) and 3-bromo-4-methylaniline (62.0 µL, 0.5 mmol, 1 equiv.). Purification by column chromatography on silica gel (*n*-pentane + 2 Vol% of Et_3N/ EtOAc + 2 Vol% of Et_3N) afforded the product as a pale-yellow crystalline solid (48.3 mg, 0.25 mmol, 51%).

R*f* (*n*-pentane/EtOAc = 2/1): 0.54

^{1}H NMR (400 MHz, $CDCl_3$) δ/ppm = 6.92 (d, *J* = 7.9 Hz, 1H), 6.59 (d, *J* = 2.4 Hz, 1H), 6.45 (dd, *J* = 8.0 Hz, *J* = 2.5 Hz, 1H), 3.50 (br s, 2H), 2.70 – 2.57 (m, 1H), 2.21 (s, 3H), 1.91 – 1.74 (m, 5H), 1.41 –1.27 (m, 5H).

^{13}C NMR (101 MHz, $CDCl_3$) δ/ppm = 146.9, 144.5, 130.9, 125.3, 113.0,112.6, 40.3,33.7, 27.3, 26.5, 18.5.

IR (ATR, cm^{-1}): 3442, 3426, 3349, 3214, 3016, 2923, 2849, 1614, 1612, 1580, 1545, 1502, 1500, 1447, 1377, 1367, 1305, 1265, 1209, 1165, 1139, 1098, 1030, 1015, 988, 946, 891, 857, 805, 670, 632, 597, 595, 502, 462.

HR-MS (ESI): m/z calcd for $[M+H]^+$ 190.1590, found 190.1597.

3-Cyclohexyl-2-methylaniline (5v)

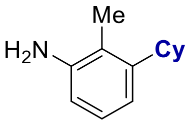

5v

Synthesized following GP 3c with ethyl 6-cyclohexyl-2-(dimethylamino)-4-methyl-1,6-dihydropyrimidine-5-carboxylate **1a** (440.1 mg, 1.5 mmol, 3.0 equiv.) and 3-bromo-2-methylaniline (61.6 µL, 0.5 mmol, 1 equiv.). Purification by column chromatography on silica gel (*n*-pentane + 2 Vol% of Et_3N/ EtOAc + 2 Vol% of Et_3N) afforded the product as a pale yellow oil (60 mg, 0.3 mmol, 63%).

R_f (*n*-pentane/EtOAc = 2/1): 0.56.

^{1}H NMR (400 MHz, $CDCl_3$) δ/ppm = 7.07 – 6.96 (t, *J* = 7.8 Hz, 1H), 6.72 (dd, *J* = 7.8 Hz, *J* = 1.2 Hz, 1H), 6.56 (dd, *J* = 7.7 Hz, *J* = 1.2 Hz, 1H), 3.57(br s, 2H), 2.79 –2.68 (m, 1H), 2.11 (s, 3H), 1.91 –1.70 (m, 5H), 1.48 – 1.21 (m, 5H).

^{13}C NMR (75 MHz, $CDCl_3$) δ/ppm = 146.6, 144.7, 126.3, 119.9, 116.3, 113.0, 40.6, 33.9, 27.3, 26.5, 12.2.

IR (ATR, cm^{-1}): 3460, 3370, 3068, 3024, 3003, 2925, 2849, 1619, 1587, 1468, 1447, 1379, 1363, 1328, 1295, 1294, 1284, 1254, 1136, 1064, 1038, 994, 891, 778, 716, 655, 479, 394.

HR-MS (ESI): m/z calcd for $[M+H]^+$ 190.1590, found 190.1587.

2-Cyclohexyl-*N,N*-dimethylaniline (5w)

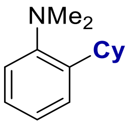

5w

Synthesized following GP 3c with ethyl 6-cyclohexyl-2-(dimethylamino)-4-methyl-1,6-dihydropyrimidine-5-carboxylate **1a** (440.1 mg, 1.5 mmol, 3.0 equiv.) and 2-bromo-*N*,*N*-dimethylaniline (72.0 µL, 0.5 mmol, 1 equiv.). Purification by column chromatography on celite (n-pentane/EtOAc) afforded the product as a pale-yellow oil (50.2 mg, 0.24 mmol, 49%).

R*f* (*n*-pentane/EtOAc = 10/1): 0.58.

^{1}H NMR (400 MHz, $CDCl_3$) δ/ppm = 7.18 (t, *J* = 7.9 Hz, 1H), 6.63 – 6.57 (m, 3H), 2.95 (s, 6H), 2.50 – 2.41 (m, 1H), 1.93 –1.70 (m, 5H), 1.51 –1.18 (m, 5H).

^{13}C NMR (101 MHz, $CDCl_3$) δ/ppm = 150.8, 149.2, 129.0, 115.4, 111.6, 110.5, 45.3, 40.9, 34.6, 27.1, 26.4.

IR (ATR, cm^{-1}): 2922, 2850, 2800, 1740, 1685, 1601, 1579, 1500, 1499, 1447, 1438, 1436, 1349, 1260, 1227, 1178, 1155, 1060, 1016, 993, 969, 909, 892, 842, 771, 732, 696, 646, 513, 464, 438, 401.

HR-MS (ESI): m/z calcd for $[M+H]^+$ 204.1747, found 204.1751.

1-Cyclopentyl-4-methoxybenzene (5aa)

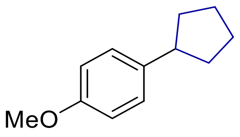

5aa

Synthesized following GP 3a with ethyl 6-cyclopentyl-2-(dimethylamino)-4-methyl-1,6-dihydropyrimidine-5-carboxylate **1i** (279.3 mg, 1.0 mmol, 2 equiv.) and 4-iodoanisole (117.0 mg, 0.5 mmol, 1.0 equiv.). Purification by column chromatography on silica gel (*n*-pentane) afforded the product as a colorless oil (94.0 mg, 0.43 mmol, 86%).

Isolated yield using GP3d on 0.2 mmol scale (photocatalyst-free version): 11 mg, 0.062 mmol, 31%

R*f* (*n*-pentane/EtOAc = 5/1): 0.50.

^{1}H NMR (400 MHz, $CDCl_3$) δ/ppm = 7.17 (m, 2H), 6.84 (m, 2H), 3.79 (s, 3H), 2.99 – 2.88 (tt, *J* = 9.7 Hz, *J* = 7.3 Hz, 1H), 2.09 – 1.99 (m, 2H), 1.85 – 1.73 (tdd, *J* = 6.7 Hz, *J* = 3.7 Hz, *J* = 1.4 Hz, 2H), 1.72 – 1.62 (m, 2H), 1.60 – 1.47 (m, 2H).

13C NMR (101 MHz, $CDCl_3$) δ/ppm = 157.7, 138.7, 128.0, 113.7, 55.4, 45.3, 34.8, 25.5.

Spectral data are in accordance with the literature.[23]

***Tert*-butyl 3-(4-methoxyphenyl)pyrrolidine-1-carboxylate (5ab)**

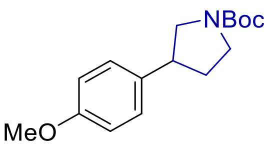

5ab

Synthesized following GP 3a with ethyl 6-(1-(tert-butoxycarbonyl)pyrrolidin-3-yl)-2-(dimethylamino)-4-methyl-1,6-dihydropyrimidine-5-carboxylate **1r** (380.4 mg, 1 mmol, 2 equiv.) and 1-iodo-4-methoxybenzene (117.0 mg, 0.5 mmol, 1 equiv.). Purification by column chromatography on silica gel afforded the product as a yellowish oil (44.4 mg, 0.16 mmol, 32%).

R_f (*n*-pentane/EtOAc = 2/1): 0.56.

^{1}H NMR (400 MHz, $CDCl_3$) δ 7.16 (d, *J* = 8.6 Hz, 2H), 6.86 (d, *J* = 8.6 Hz, 2H), 3.79 (s, 3H), 3.76 – 3.14(m, 5H), 2.28 – 2.13 (m, 1H), 2.0 –1.85 (m, 1H), 1.46 (s, 9H).

^{13}C NMR (101 MHz, $CDCl_3$) δ 158.5, 154.6, 133.5, 128.1, 114.0, 79.2, 55.3, 52.8, 52.1, 46.0, 45.7, 43.6, 42.7, 33.6, 32.7, 28.6.

Spectral data are in accordance with the literature.[16]

1-Butyl-4-methoxybenzene (5ac)

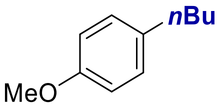

5ac

Synthesized following GP 3b with ethyl 6-butyl-2-(dimethylamino)-4-methyl-1,6-dihydropyrimidine-5-carboxylate **1s** (200.3 mg, 0.75 mmol, 3 equiv.) and 1-bromo-4-methoxybenzene (31.4 µL, 0.25 mmol, 1 equiv.). Purification by column chromatography on silica gel afforded the product as a yellowish oil (6.9 mg, 0.042 mmol, 17%).

R_f (*n*-pentane/EtOAc = 10/1): 0.54.

^{1}H NMR (400 MHz, $CDCl_3$) δ/ppm = 7.10 (m, 2H), 6.83 (m, 2H), 3.79 (s, 3H), 2.55 (m, 2H),1.62 –1.50 (m, 2H), 1.39 – 1.29 (m, 2H), 0.92 (t, *J* = 7.3 Hz, 3H).

^{13}C NMR (101 MHz, $CDCl_3$) δ/ppm = 157.7, 135.1, 129.4, 113.7, 55.4, 34.8, 34.0, 22.4, 14.1.

Spectral data are in accordance with the literature.[19]

1-Isopropyl-4-(2-(4-methoxyphenyl)propyl)benzene (5ad)

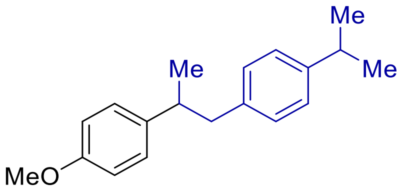

5ad

Synthesized following GP 3a with ethyl 2-(dimethylamino)-6-(1-(4-isopropylphenyl)propan-2-yl)-4-methyl-1,6-dihydropyrimidine-5-carboxylate **1o** (371.5 mg, 1.0 mmol, 2 equiv.) and 4-iodoanisole (117.0 mg, 0.5 mmol, 1.0 equiv.). Purification by column chromatography on silica gel afforded the product as a colorless oil (108.8 mg, 0.41 mmol, 82%).

R*f* (*n*-pentane/EtOAc = 20/1): 0.52.

^{1}H NMR (400 MHz, $CDCl_3$) δ/ppm = 7.14 – 7.07 (dd, *J* = 8.4 Hz, *J* = 6.4 Hz, 4H), 7.04 – 6.99 (d, *J* = 8.1 Hz, *J* = 1.4 Hz, 2H), 6.86 – 6.80 (m, 2H), 3.80 (s, 3H), 2.99 – 2.80 (m, 3H), 2.72 – 2.64 (dd *J* = 12.9 Hz, *J* = 8.1 Hz, 1H), 1.25 – 1.27 (dd, *J* = 3.0 Hz, *J* = 6.8 Hz, 9H).

^{13}C NMR (101 MHz, $CDCl_3$) δ/ppm = 157.9, 146.4, 139.5, 138.3, 129.1, 128.3, 126.2, 113.7, 55.3, 44.9, 41.0, 33.8, 24.2, 21.4.

IR (ATR, cm^{-1}): 3006, 2957, 2926, 2870, 2834, 1612, 1584, 1511, 1462, 1419, 1363, 1302, 1244, 1177, 1110, 1038, 1019, 872, 827, 803, 755, 714, 639, 616, 582, 580, 552, 550, 472, 406, 392.

HR-MS (ESI): m/z calcd for $[M+Na]^+$ 291.1719, found 291.1725

1-Benzyl-4-methoxybenzene (5ae)

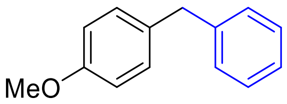

5ae

Synthesized following GP 3a with ethyl 6-benzyl-2-(dimethylamino)-4-methyl-1,6-dihydropyrimidine-5-carboxylate **1p** (119.3 mg, 0.4 mmol, 2.0 equiv.) and 1-iodo-4-methoxybenzene (46.8 mg, 0.2 mmol, 1 equiv.). Purification by column chromatography on silica gel afforded the product as a colorless oil (17.8 mg, 0.09 mmol, 45%).

R_f (*n*-pentane/EtOAc = 5/1): 0.77.

^{1}H NMR (400 MHz, $CDCl_3$) δ 7.31 – 7.26 (m, 2H), 7.21 – 7.16 (m, 3H), 7.11 (d, *J* = 8.8 Hz, 2H), 6.83 (d, *J* = 8.6 Hz, 2H), 3.93 (s, 2H), 3.79 (s, 3H).

^{13}C NMR (101 MHz, $CDCl_3$) δ 158.1, 141.7, 133.4, 130.0, 128.9, 128.5, 126.1, 114.0, 55.4, 41.1

Spectral data are in accordance with the literature.[16]

1-Methoxy-4-(6-methylhept-5-en-2-yl)benzene (5af)

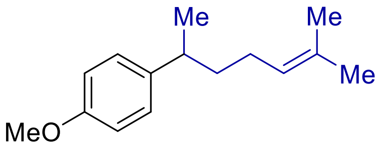

5af

Synthesized following GP 3a with ethyl 2-(dimethylamino)-4-methyl-6-(6-methylhept-5-en-2-yl)-1,6-dihydropyrimidine-5-carboxylate **1n** (321.3 mg, 1 mmol, 2.0 equiv.) and 1-iodo-4-methoxybenzene (117 mg, 0.5 mmol, 1 equiv.). Purification by column chromatography on silica gel afforded the product as a colorless oil (68 mg, 0.31 mmol, 62%).

Isolated yield using GP3d on 0.2 mmol scale (photocatalyst-free version): 28 mg, 0.128 mmol, 64%

R_f (*n*-pentane/EtOAc = 20/1): 0.34.

^{1}H NMR (400 MHz, $CDCl_3$) δ/ppm = 7.13 – 7.06 (m, 2H), 6.87 – 6.81 (m, 2H), 5.15 – 5.02 (m, 1H), 3.79 (s, 3H), 2.65 (h, *J* = 7.0 Hz, 1H), 1.92 – 1.80 (m, 2H), 1.71 – 1.63 (m, 3H), 1.61 – 1.49 (m, 5H), 1.21 (d, *J* = 6.9 Hz, 3H).

^{13}C NMR (101 MHz, $CDCl_3$) δ/ppm = 157.8, 139.9, 131.5, 128.0, 124.7, 113.8, 55.4, 38.7, 26.3, 25.9, 22.7, 17.8.

HR-MS (GC-MS): m/z calcd for $[M]^+$ 218.1665, found 218.1669.

4-(4-Methoxyphenyl)-2,2-dimethyl-1,3-dioxolane (5ag)

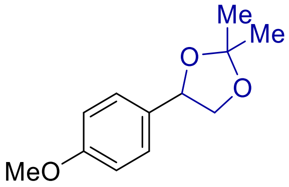

5ag

Synthesized following GP 3a with ethyl 6-(2,2-dimethyl-1,3-dioxolan-4-yl)-2-(dimethylamino)-4-methyl-1,6-dihydropyrimidine-5-carboxylate **1k** (311 mg, 1.0 mmol, 2 equiv.) and 4-iodoanisole (117.0 mg, 0.5 mmol, 1.0 equiv.). Purification by column chromatography on silica gel afforded the product as a colorless oil (61.5 mg, 0.30 mmol, 59%).

Isolated yield using GP3d on 0.2 mmol scale (photocatalyst-free version): 5 mg, 0.024 mmol, 12%

R$_f$ (*n*-pentane/EtOAc = 10/1): 0.24.

^{1}H NMR (400 MHz, $CDCl_3$) δ/ppm = 7.36 – 7.23 (m, 2H), 6.95 – 6.84 (m, 2H), 5.02 (dd, *J* = 8.2, 6.1 Hz, 1H), 4.25 (dd, *J* = 8.2, 6.1 Hz, 1H), 3.80 (s, 3H), 3.69 (t, *J* = 8.2 Hz, 1H), 1.54 (d, *J* = 0.7 Hz, 3H), 1.48 (d, *J* = 0.7 Hz, 3H).

^{13}C NMR (101 MHz, $CDCl_3$) δ/ppm = 159.7, 131.0, 127.8, 114.1, 109.6, 77.9, 71.8, 55.4, 26.9, 26.1.

HR-MS (ESI): m/z calcd for $[M+Na]^+$ 231.0992, found 231.0626.

3-(4-Methoxyphenyl)tetrahydrofuran (5ah)

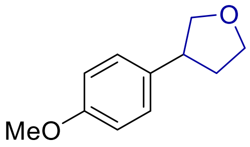

5ah

Synthesized following GP 3a with ethyl 2-(dimethylamino)-4-methyl-6-(tetrahydrofuran-3-yl)-1,6-dihydropyrimidine-5-carboxylate **1l** (281 mg, 1.0 mmol, 2 equiv.) and 4-iodoanisole (117.0 mg, 0.5 mmol, 1.0 equiv.). Purification by column chromatography on silica gel afforded the product as a colorless oil (47 mg, 0.27 mmol, 53%).

R$_f$ (*n*-pentane/EtOAc = 10/1): 0.31.

^{1}H NMR (400 MHz, $CDCl_3$) δ/ppm = 7.23 – 7.12 (m, 2H), 6.91 – 6.80 (m, 2H), 4.18 – 3.99 (m, 2H), 3.91 (td, *J* = 8.2, 7.2 Hz, 1H), 3.80 (s, 3H), 3.67 (t, *J* = 8.0 Hz, 1H), 3.36 (p, *J* = 7.9 Hz, 1H), 2.42 – 2.25 (m, 1H), 1.97 (dq, *J* = 12.2, 8.2 Hz, 1H).

^{13}**C NMR** (101 MHz, $CDCl_3$) δ/ppm = 158.4, 134.7, 128.3, 114.1, 74.9, 68.6, 55.4, 44.4, 34.9.

HR-MS (GC-MS): m/z calcd for $[M]^+$ 178.0988, found 178.0991.

5-(4-methoxyphenyl)-2,2,7,7-tetramethyltetrahydro-5H-bis([1,3]dioxolo)[4,5-b:4',5'-d]pyran (5ai)

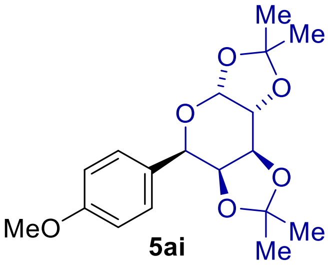

5ai

Synthesized following GP 3a with ethyl 2-(dimethylamino)-4-methyl-6-((3aS,5aR,8aR,8bS)-2,2,7,7-tetramethyltetrahydro-5H-bis([1,3]dioxolo)[4,5-b:4',5'-d]pyran-5-yl)-1,6-dihydropyrimidine-5-carboxylate **1t** (439.2 mg, 1 mmol, 2.0 equiv.) and 1-iodo-4-methoxybenzene (117.02 mg, 0.5 mmol, 1 equiv.). Purification by column chromatography on silica gel afforded the product as a pale yellowish solid (23.5 mg, 0.07 mmol, 14%).

$\mathbf{R}_f$ (*n*-pentane/EtOAc = 2/1): 0.60

^{1}**H NMR** (400 MHz, $CDCl_3$) δ 7.33 (d, *J* = 8.2 Hz, 2H), 6.88 (d, *J* = 8.7 Hz, 2H), 5.69 (d, *J* = 4.9 Hz, 1H), 4.84 (d, *J* = 1.8 Hz, 1H), 4.70 (dd, *J* = 7.8 Hz, *J* = 2.3 Hz, 1H),4.42– 4.35 (m, 2H),3.79 (s, 3H),1.57 (s ,3H),1.48 (s, 3H), 1.37 (s, 3H), 1.30 (s, 3H).

^{13}**C NMR** (101 MHz, $CDCl_3$) 159.1, 129.9, 129.2, 128.4, 114.6, 113.6, 109.3, 108.7, 97.1, 74.0, 71.2, 70.8, 69.2, 55.3, 29.8, 26.3, 26.1, 25.1, 24.3, 22.9.

HR-MS (ESI): m/z calcd for $[M+Na]^+$ 359.1465, found 359.1469.

Reductive alkylation of nitroarenes:
General procedure 4 (GP 4)

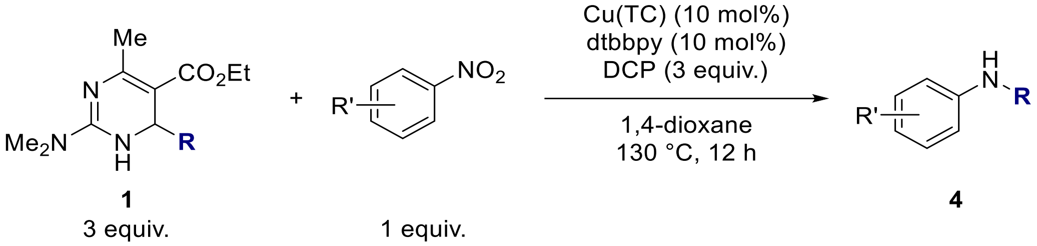

In analogy to the literature,[24] to an oven-dried screw-capped Schlenk tube equipped with a magnetic stir bar was added dihydropyrimidine **1** (0.6 mmol, 3.0 equiv.), nitrobenzene derivative (0.2 mmol, 1.0 equiv.), Cu(TC) (3.8 mg, 0.02 mmol, 10 mol%), dtbbpy (5.4 mg, 0.02 mmol, 10 mol%) and dicumyl peroxide (DCP) (162.2 mg, 0.6 mmol, 3 equiv.). After evacuating and refilling with argon at least three cycles, dry 1,4-dioxane (2.0 mL) was added under argon and the mixture was stirred in a pre-heated oil bath at 130 °C for 12 h.

N-Cyclohexylaniline (7a)

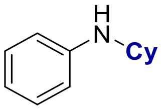

7a

Synthesized following GP 4 with dihydropyrimidine **1a** (176.0 mg, 0.6 mmol, 3 equiv.) and nitrobenzene (20.5 µL, 0.2 mmol, 1 equiv.). Purification by column chromatography on silica gel (*n*-pentane/EtOAc) afforded the product as a yellow oil (20.2 mg, 0.12 mmol, 58%). Dihydropyrimidine **1b** and **1c** afforded the product in (20.1 mg, 0.12 mmol, 58%) respectively (13.3 mg, 0.08 mmol, 38%).

$\mathbf{R}_f$ (EtOAc/EtOH = 3/1): 0.40.

^{1}H NMR (400 MHz, $CDCl_3$) δ/ppm = 7.18 – 7.12 (m, 2H), 6.65 (t, *J* = 7.3 Hz, 1H), 6.59 (d, *J* = 7.5 Hz, 2H), 3.52 (s, 1H), 3.31 – 3.20 (m, 1H), 2.10 – 2.01 (m, 2H), 1.80 – 1.72 (m, 2H), 1.69 – 1.61 (m, 1H), 1.43 – 1.32 (m, 2H), 1.25 – 1.10 (m, 3H).

^{13}C NMR (101 MHz, $CDCl_3$) δ/ppm = 147.5, 129.4, 116.9, 113.3, 51.8, 33.6, 26.1, 25.2.

Spectral data are in accordance with the literature.[24]

The reaction was repeated as the following variations:

- in the presence of 1 equiv. TEMPO (67% yield of product **7a**)

- in the presence of 3 equiv. TEMPO (11% yield of product **7a**)
- under air, closed flask (73% yield of product **7a**)
- with 5 equiv. H_2O (75% yield of product **7a**)

***N*-Cyclohexyl-2-methoxyaniline (7b)**

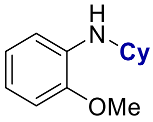

7b

Synthesized following GP 4 with dihydropyrimidine **1a** (176.0 mg, 0.6 mmol, 3 equiv.) and 1-methoxy-2-nitrobenzene (24.4 µL, 0.2 mmol, 1 equiv.). Purification by column chromatography on silica gel (*n*-pentane/EtOAc) afforded the product as a yellow oil (21.1 mg, 0.10 mmol, 51%).

R_f (EtOAc/EtOH = 3/1): 0.40.

^{1}H NMR (300 MHz, $CDCl_3$) δ/ppm = 6.86 (td, *J* = 7.6, 1.5 Hz, 1H), 6.80 – 6.74 (m, 1H), 6.67 – 6.59 (m, 2H), 4.22 – 4.07 (m, 1H), 3.84 (s, 3H), 3.32 – 3.18 (m, 1H), 2.13 – 2.02 (m, 2H), 1.84 – 1.73 (m, 2H), 1.71 – 1.60 (m, 1H), 1.43 – 1.31 (m, 2H), 1.27 – 1.13 (m, 3H).

^{13}C NMR (75 MHz, $CDCl_3$) δ/ppm = 146.8, 137.4, 121.4, 115.9, 110.3, 109.7, 55.5, 51.5, 33.6, 26.2, 25.2.

Spectral data are in accordance with the literature.[25]

4-Bromo-*N*-cyclohexylaniline (7c)

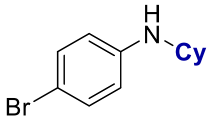

7c

Synthesized following GP 4 with dihydropyrimidine **1a** (176.0 mg, 0.6 mmol, 3 equiv.) and 1-bromo-4-nitrobenzene (40.4 mg, 0.2 mmol, 1.0 equiv.). Purification by column chromatography on silica gel (*n*-pentane/EtOAc) afforded the product as a yellow oil (38.9 mg, 0.15 mmol, 76%).

R_f (EtOAc/EtOH = 3/1): 0.32.

^{1}**H NMR** (300 MHz, $CDCl_3$) δ/ppm = 7.22 (d, *J* = 8.8 Hz, 2H), 6.46 (d, *J* = 8.8 Hz, 2H), 3.54 (s, 1H), 3.27 – 3.13 (m, 1H), 2.11 – 1.96 (m, 2H), 1.81 – 1.71 (m, 2H), 1.70 – 1.60 (m, 1H), 1.42 – 1.28 (m, 2H), 1.22 – 1.07 (m, 3H).

^{13}**C NMR** (75 MHz, $CDCl_3$) δ/ppm = 146.5, 132.0, 114.8, 108.3, 51.9, 33.4, 26.0, 25.1.

Spectral data are in accordance with the literature.[24]

***N*-Cyclohexyl-3-methylaniline (7d)**

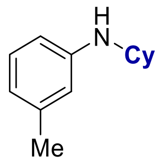

7d

Synthesized following GP 4 with dihydropyrimidine **1a** (176.0 mg, 0.6 mmol, 3 equiv.) and 1-methyl-3-nitrobenzene (23.7 µL, 0.2 mmol, 1 equiv.). Purification by column chromatography on silica gel (*n*-pentane/EtOAc) afforded the product as a yellow oil (29.6 mg, 0.16 mmol, 78%).

R*f* (EtOAc/EtOH = 3/1): 0.36.

^{1}**H NMR** (300 MHz, $CDCl_3$) δ/ppm = 7.11 – 6.99 (m, 1H), 6.49 (d, *J* = 7.4 Hz, 1H), 6.45 – 6.38 (m, 2H), 3.47 (s, 1H), 3.31 – 3.19 (m, 1H), 2.27 (s, 3H), 2.11 – 2.00 (m, 2H), 1.80 – 1.72 (m, 2H), 1.41 – 1.11 (m, 6H).

^{13}**C NMR** (75 MHz, $CDCl_3$) δ/ppm = 147.6, 139.1, 129.3, 117.9, 114.0, 110.4, 51.8, 33.7, 26.1, 25.2, 21.8.

Spectral data are in accordance with the literature.[26]

5-Bromo-*N*-cyclohexyl-2-fluoroaniline (7e)

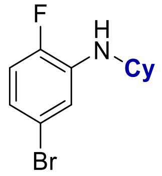

7e

Synthesized following GP 4 with dihydropyrimidine **1a** (176.0 mg, 0.6 mmol, 3 equiv.) and 4-bromo-1-fluoro-2-nitrobenzene (44.0 mg, 0.2 mmol, 1 equiv.). Purification by column

chromatography on silica gel (*n*-pentane/EtOAc) afforded the product as a yellow oil (16.7 mg, 0.06 mmol, 31%).

R$_f$ (EtOAc/EtOH = 3/1): 0.44.

^{1}H NMR (400 MHz, $CDCl_3$) δ/ppm = 6.88 – 6.78 (m, 2H), 6.72 – 6.67 (m, 1H), 3.88 (s, 1H), 3.32 – 3.20 (m, 1H), 2.12 – 2.04 (m, 2H), 1.86 – 1.78 (m, 2H), 1.75 – 1.67 (m, 1H), 1.49 – 1.37 (m, 2H), 1.34 – 1.18 (m, 3H).

^{13}C NMR (101 MHz, $CDCl_3$) δ/ppm = 151.7, 149.4, 137.4 (d, *J* = 12.7 Hz), 118.2 (d, *J* = 7.0 Hz), 117.4 (d, *J* = 3.1 Hz), 115.9, 115.7, 114.9 (d, *J* = 3.8 Hz), 51.4, 33.3, 25.9, 25.0.

^{19}F NMR (377 MHz, $CDCl_3$) δ/ppm = -138.9.

IR (ATR in $CDCl_3$, cm^{-1}): 2931, 2859, 2252, 1615, 1151, 1454, 1415, 1180, 904, 727, 650.

HR-MS (ESI): m/z calcd for $[M+H]^+$ 272.0445, found 272.0452.

2-Bromo-*N*-cyclohexylaniline (7f)

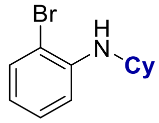

7f

Synthesized following GP 4 with dihydropyrimidine **1a** (176.0 mg, 0.6 mmol, 3 equiv.) and 1-bromo-2-nitrobenzene (40.4 mg, 0.2 mmol, 1 equiv.). Purification by column chromatography on silica gel (*n*-pentane/EtOAc) afforded the product as a yellow oil (22.2 mg, 0.09 mmol, 45%).

R$_f$ (EtOAc/EtOH = 3/1): 0.52.

^{1}H NMR (400 MHz, $CDCl_3$) δ/ppm = 7.41 (dd, *J* = 7.9, 1.5 Hz, 1H), 7.15 (ddd, *J* = 8.6, 7.2, 1.5 Hz, 1H), 6.65 (dd, *J* = 8.1, 1.5 Hz, 1H), 6.52 (td, *J* = 7.6, 1.5 Hz, 1H), 4.24 (s, 1H), 3.39 – 3.25 (m, 1H), 2.09 – 2.00 (m, 2H), 1.84 – 1.73 (m, 2H), 1.70 – 1.60 (m, 1H), 1.45 – 1.34 (m, 2H), 1.33 – 1.21 (m, 3H).

^{13}C NMR (101 MHz, $CDCl_3$) δ/ppm = 144.3, 132.7, 128.5, 117.2, 111.9, 109.9, 51.7, 33.2, 26.0, 25.0.

Spectral data are in accordance with the literature.[27]

2,5-Dibromo-*N*-cyclohexylaniline (7g)

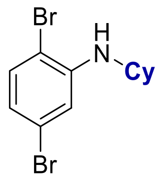

7g

Synthesized following GP 4 with dihydropyrimidine **1a** (176.0 mg, 0.6 mmol, 3 equiv.) and 1,4-dibromo-2-nitrobenzene (56.2 mg, 0.2 mmol, 1 equiv.). Purification by column chromatography on silica gel (*n*-pentane/EtOAc) afforded the product as a colorless oil (8.9 mg, 0.03 mmol, 15%)

R_f (EtOAc/EtOH = 3/1): 0.64.

^{1}H NMR (400 MHz, $CDCl_3$) δ/ppm = 7.23 (d, *J* = 8.4 Hz, 1H), 6.72 (d, *J* = 2.2 Hz, 1H), 6.62 (dd, *J* = 8.4, 2.2 Hz, 1H), 4.34 – 4.22 (m, 1H), 3.32 – 3.21 (m, 1H), 2.07 – 1.98 (m, 2H), 1.83 – 1.72 (m, 2H), 1.70 – 1.61 (m, 1H), 1.46 – 1.35 (m, 2H), 1.30 – 1.21 (m, 3H).

^{13}C NMR (101 MHz, $CDCl_3$) δ/ppm = 145.3, 133.5, 122.4, 119.8, 114.3, 108.3, 51.6, 33.1, 25.9, 24.9.

IR (ATR in $CDCl_3$, cm^{-1}): 3402, 2927, 2852, 1585, 1503, 1449, 1410, 1291, 1016, 829, 780.

HR-MS (ESI): m/z calcd for $[M+H]^+$ 331.9647, found 331.9644.

***N*-cyclohexyl-4-methylaniline (7h)**

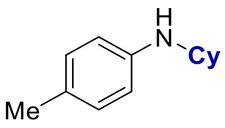

7h

Synthesized following GP 4 with dihydropyrimidine **1a** (176.0 mg, 0.6 mmol, 3 equiv.) and 1-methyl-4-nitrobenzene (27.4 mg, 0.2 mmol, 1 equiv.). Purification by column chromatography on silica gel (*n*-pentane/EtOAc) afforded the product as a yellow oil (28.2 mg, 0.15 mmol, 75%).

R_f (EtOAc/EtOH = 3/1): 0.32.

^{1}H NMR (400 MHz, $CDCl_3$) δ/ppm = 6.96 (d, *J* = 7.8 Hz, 2H), 6.52 (d, *J* = 8.5 Hz, 2H), 3.37 (s, 1H), 3.27 – 3.15 (m, 1H), 2.23 (s, 3H), 2.10 – 1.99 (m, 2H), 1.80 – 1.69 (m, 2H), 1.69 – 1.60 (m, 1H), 1.44 – 1.29 (m, 2H), 1.26 – 1.07 (m, 3H).

^{13}C NMR (101 MHz, $CDCl_3$) δ/ppm = 145.3, 129.9, 126.2, 113.6, 52.2, 33.7, 26.1, 25.2, 20.5.

Spectral data are in accordance with the literature.[28]

N-cyclohexyl-2-methoxy-4-methylaniline (7i)

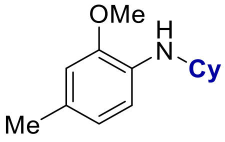

7i

Synthesized following GP 4 with dihydropyrimidine **1a** (176.0 mg, 0.6 mmol, 3 equiv.) and 2-methoxy-4-methyl-1-nitrobenzene (33.4 mg, 0.2 mmol, 1 equiv.). Purification by column chromatography on silica gel (*n*-pentane/EtOAc) afforded the product as a yellow oil (17.0 mg, 0.08 mmol, 40%).

R*f* (EtOAc/EtOH = 3/1): 0.32.

^{1}H NMR (400 MHz, $CDCl_3$) δ/ppm = 6.65 (d, *J* = 7.9 Hz, 1H), 6.60 (d, *J* = 1.8 Hz, 1H), 6.54 (d, *J* = 7.9 Hz, 1H), 3.83 (s, 3H), 3.22 (s, 1H), 2.27 (s, 3H), 2.11 – 2.02 (m, 2H), 1.81 – 1.71 (m, 2H), 1.69 – 1.61 (m, 1H), 1.43 – 1.31 (m, 2H), 1.29 – 1.13 (m, 3H).

^{13}C NMR (101 MHz, $CDCl_3$) δ/ppm = 147.0, 135.0, 125.5, 124.7, 121.3, 111.0, 110.6, 55.5, 51.7, 33.6, 29.0, 26.2, 25.2, 21.0.

IR (ATR in $CDCl_3$, cm^{-1}): 3419, 2928, 2852, 2186, 2304, 1617, 1521, 1453, 1040, 797.

HR-MS (ESI): m/z calcd for $[M+H]^+$ 220.1696, found 220.1697.

N-(1-(benzo[*d*][1,3]dioxol-5-yl)propan-2-yl)aniline (7j)

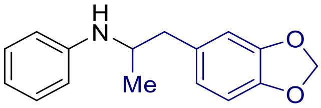

7j

Synthesized following GP 4 with dihydropyrimidine **1j** (224.1 mg, 0.6 mmol, 3 equiv.) and nitrobenzene (20.5 µL, 0.2 mmol, 1 equiv.). Purification by column chromatography on silica gel (*n*-pentane/EtOAc) afforded the product as a yellow oil (32.3 mg, 0.13 mmol, 65%).

R*f* (EtOAc/EtOH = 3/1): 0.20.

^{1}H NMR (400 MHz, $CDCl_3$) δ/ppm = 7.23 – 7.16 (m, 2H), 6.76 (d, *J* = 7.9 Hz, 1H), 6.73 – 6.67 (m, 2H), 6.67 – 6.60 (m, 3H), 5.94 (s, 2H), 3.78 – 3.66 (m, 1H), 3.53 (s, 1H), 2.85 (dd, *J* = 13.6, 4.9 Hz, 1H), 2.64 (dd, *J* = 13.5, 7.2 Hz, 1H).

^{13}C NMR (101 MHz, $CDCl_3$) δ/ppm = 160.2, 147.6, 147.2, 146.1, 132.3, 129.4, 122.4, 117.2, 113.4, 109.8, 108.2, 100.9, 71.6, 49.5, 42.0, 20.2.

IR (ATR in $CDCl_3$, cm^{-1}): 3402, 2964, 2923, 2886, 1601, 1502, 1441, 1247, 1188, 1039, 908, 730, 693.

HR-MS (ESI): m/z calcd for $[M+H]^+$ 256.1332, found 256.1338.

Cu-catalyzed alkylation of disulfides:

General procedure 5 (GP 5)

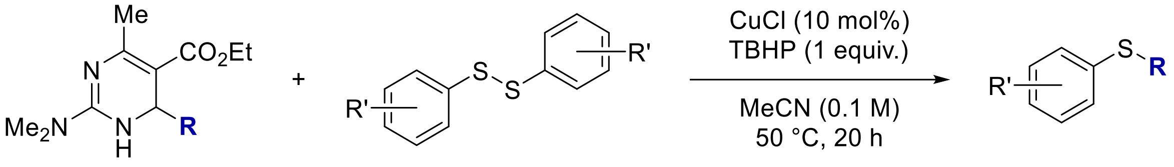

1 1 equiv. 1 equiv. **5**

To an oven-dried screw-capped Schlenk tube equipped with a magnetic stir bar was added dihydropyrimidine (0.2 mmol, 1.0 equiv.), disulfide (0.2 mmol, 1.0 equiv.) and CuCl (2.0 mg, 0.02 mmol, 10 mol%). After evacuating and refilling with argon at least three cycles, dry MeCN (0.1 M, 2 mL) and *tert*-Butyl hydroperoxide (20 µL, 0.2 mmol, 1.0 equiv.) was added under argon and the mixture was stirred in a pre-heated oil bath at 50 °C for 20 h. The reaction was cooled to ambient temperature and absorbed on silica gel for subsequent column chromatography.

Cyclohexyl(phenyl)sulfane (9a)

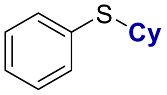

9a

Synthesized following GP 5 with dihydropyrimidine **1a** (58.7 mg, 0.2 mmol, 1 equiv.) and diphenyl disulfide (43.7 mg, 0.2 mmol, 1 equiv.). Purification by column chromatography on silica gel (*n*-pentane) afforded the product as a colorless oil (29.6 mg, 0.15 mmol, 76%).

R_f (*n*-Pentane): 0.42.

^{1}H NMR (300 MHz, $CDCl_3$) δ/ppm = 7.43 – 7.37 (m, 2H), 7.32 – 7.18 (m, 3H), 3.16 – 3.05 (m, 1H), 2.04 – 1.91 (m, 2H), 1.85 – 1.71 (m, 2H), 1.68 – 1.57 (m, 1H), 1.42 – 1.23 (m, 5H).

^{13}C NMR (75 MHz, $CDCl_3$) δ/ppm = 135.3, 132.0, 128.9, 126.7, 46.7, 33.5, 26.2, 25.9.

Spectral data are in accordance with the literature.[29]

The reaction was repeated as the following variations:

- in the presence of 1 equiv. TEMPO (complete shutdown of reaction)
- under air, closed flask (79% yield of product **9a**)
- with 5 equiv. H_2O (60% yield of product **9a**)

(4-Bromophenyl)(cyclohexyl)sulfane (9b)

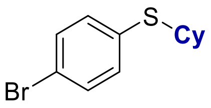

9b

Synthesized following GP 5 with dihydropyrimidine **1a** (58.7 mg, 0.2 mmol, 1 equiv.) and bis(4-bromophenyl) disulfide (75.2 mg, 0.2 mmol, 1 equiv.). Purification by column chromatography on silica gel (*n*-pentane) afforded the product as a colorless oil (41.0 mg, 0.15 mmol, 75%).

R_f (*n*-Pentane): 0.52.

^{1}H NMR (300 MHz, $CDCl_3$) δ/ppm = 7.42 – 7.37 (m, 2H), 7.28 – 7.22 (m, 2H), 3.12 – 3.01 (m, 1H), 2.01 – 1.91 (m, 2H), 1.82 – 1.71 (m, 2H), 1.66 – 1.56 (m, 1H), 1.41 – 1.21 (m, 5H).

^{13}C NMR (75 MHz, $CDCl_3$) δ/ppm = 134.5, 133.5, 132.0, 120.7, 46.9, 33.4, 26.1, 25.8.

Spectral data are in accordance with the literature.[30]

(4-Chlorophenyl)(cyclohexyl)sulfane (9c)

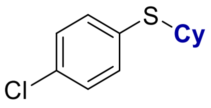

9c

Synthesized following GP 5 with dihydropyrimidine **1a** (58.7 mg, 0.2 mmol, 1 equiv.) and bis(4-chlorophenyl) disulfide (57.4 mg, 0.2 mmol, 1 equiv.). Purification by column chromatography on silica gel (*n*-pentane) afforded the product as a colorless oil (30.6 mg, 0.13 mmol, 67%).

R_f (*n*-Pentane): 0.47.

^{1}H NMR (300 MHz, $CDCl_3$) δ/ppm = 7.35 – 7.29 (m, 2H), 7.28 – 7.22 (m, 2H), 3.11 – 3.00 (m, 1H), 2.01 – 1.92 (m, 2H), 1.81 – 1.72 (m, 2H), 1.66 – 1.57 (m, 1H), 1.39 – 1.24 (m, 5H).

^{13}C NMR (75 MHz, $CDCl_3$) δ/ppm = 133.8, 133.4, 132.8, 129.0, 47.0, 33.4, 26.2, 25.8.

Spectral data are in accordance with the literature.[31]

(1-(4-*Iso*propylphenyl)propan-2-yl)(phenyl)sulfane (9d)

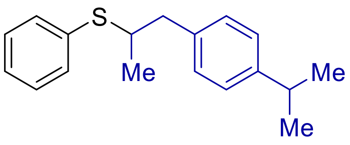

9d

Synthesized following GP 5 with dihydropyrimidine **1o** (74.3 mg, 0.2 mmol, 1 equiv.) and diphenyl disulfide (43.7 mg, 0.2 mmol, 1 equiv.). Purification by column chromatography on silica gel (*n*-pentane) afforded the product as a colorless oil (6.8 mg, 0.03 mmol, 13%).

R*f* (*n*-Pentane/EtOAc = 20/1): 0.36.

^{1}H NMR (300 MHz, $CDCl_3$) δ/ppm = 7.45 – 7.39 (m, 2H), 7.34 – 7.27 (m, 2H), 7.25 – 7.06 (m, 5H), 3.53 – 3.36 (m, 1H), 3.08 – 2.81 (m, 2H), 2.62 (dd, *J* = 13.6, 9.1 Hz, 1H), 1.24 (d, *J* = 6.8 Hz, 9H).

^{13}C NMR (101 MHz, $CDCl_3$) δ/ppm = 147.1, 136.7, 135.4, 132.1, 129.3, 129.0, 126.9, 126.5, 44.6, 42.9, 33.9, 24.2, 20.3.

Spectral data are in accordance with the literature.[32]

(6-Methylhept-5-en-2-yl)(phenyl)sulfane (9e)

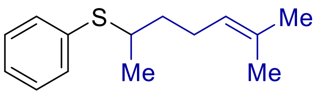

9e

Synthesized following GP 5 with dihydropyrimidine **1n** (64.3 mg, 0.2 mmol, 1 equiv.) and diphenyl disulfide (43.7 mg, 0.2 mmol, 1 equiv.). Purification by column chromatography on silica gel (*n*-pentane) afforded the product as a colorless oil (16.3 mg, 0.07 mmol, 37%).

R*f* (*n*-Pentane/EtOAc = 20/1): 0.57.

^{1}H NMR (400 MHz, $CDCl_3$) δ/ppm = 7.43 – 7.35 (m, 2H), 7.31 – 7.26 (m, 2H), 7.24 – 7.19 (m, 1H), 5.09 (tdp, *J* = 7.2, 2.9, 1.5 Hz, 1H), 3.28 – 3.15 (m, 1H), 2.25 – 2.05 (m, 2H), 1.69 (q, *J* = 1.4 Hz, 3H), 1.67 – 1.62 (m, 1H), 1.61 (d, *J* = 1.1 Hz, 3H), 1.58 – 1.48 (m, 1H), 1.28 (d, *J* = 6.7 Hz, 3H).

^{13}C NMR (101 MHz, $CDCl_3$) δ/ppm = 147.1, 136.7, 135.4, 132.1, 129.7, 129, 126.9, 126.5, 44.6, 42.9, 33.8, 24.2, 20.3.

IR (ATR, cm^{-1}): 3059, 2965, 2920, 2857, 1584, 1477, 1439, 1261, 1092, 1024, 824, 742.

HR-MS (EI): m/z calcd for $[M]^+$ 220.1280, found 220.1287.

2,2-Dimethyl-4-(phenylthio)-1,3-dioxolane (9f)

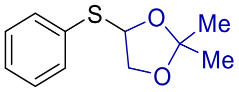

9f

Synthesized following GP 5 with dihydropyrimidine **1k** (62.3 mg, 0.2 mmol, 1 equiv.) and diphenyl disulfide (43.7 mg, 0.2 mmol, 1 equiv.). Purification by column chromatography on silica gel (*n*-pentane) afforded the product as a colorless oil (28.0 mg, 0.13 mmol, 67%).

$\mathbf{R}_f$ (*n*-Pentane/EtOAc = 5/1): 0.46.

^{1}**H NMR** (300 MHz, $CDCl_3$) δ/ppm = 7.52 – 7.46 (m, 2H), 7.33 – 7.26 (m, 2H), 7.26 – 7.21 (m, 1H), 5.56 (t, *J* = 5.9 Hz, 1H), 4.35 (dd, *J* = 9.2, 6.4 Hz, 1H), 3.96 (dd, *J* = 9.2, 5.6 Hz, 1H), 1.51 (s, 3H), 1.42 (s, 3H).

^{13}**C NMR** (75 MHz, $CDCl_3$) δ/ppm = 134.3, 131.9, 129.1, 127.5, 111.6, 83.8, 69.6, 26.4.

Spectral data are in accordance with the literature.[33]

(4-Bromophenyl)(butyl)sulfane (9g)

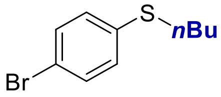

9g

Synthesized following GP 5 with dihydropyrimidine **1s** (53.5 mg, 0.2 mmol, 1 equiv.) and bis(4-bromophenyl) disulfide (75.2 mg, 0.2 mmol, 1 equiv.). Purification by column chromatography on silica gel (*n*-pentane) afforded the product as a colorless oil (7.3 mg, 0.03 mmol, 15%).

$\mathbf{R}_f$ (*n*-Pentane/EtOAc = 20/1): 0.36.

^{1}**H NMR** (400 MHz, $CDCl_3$) δ/ppm = 7.39 (d, *J* = 8.5 Hz, 2H), 7.18 (d, *J* = 8.6 Hz, 2H), 2.94 – 2.85 (m, 2H), 1.65 – 1.58 (m, 2H), 1.45 – 1.41 (m, 2H), 0.87 – 0.82 (m, 3H).

^{13}**C NMR** (101 MHz, $CDCl_3$) δ/ppm = 136.4, 132.0, 130.5, 119.5, 33.5, 31.2, 22.1, 13.8.

Spectral data are in accordance with the literature.[34]

Cyclohexyl(4-(trifluoromethyl)phenyl)sulfane (9h)

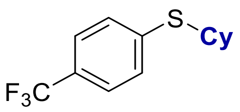

9h

Synthesized following GP 5 with dihydropyrimidine **1a** (58.7 mg, 0.2 mmol, 1 equiv.) and 1,2-bis(4-(trifluoromethyl)phenyl)disulfane (70.9 mg, 0.2 mmol, 1 equiv.). Purification by column chromatography on silica gel (*n*-pentane) afforded the product as white crystals (39.1 mg, 0.15 mmol, 75%).

R*f* (*n*-Pentane/EtOAc = 10/1): 0.48.

^{1}H NMR (300 MHz, $CDCl_3$) δ/ppm = 7.51 (d, *J* = 8.3 Hz, 2H), 7.41 (d, *J* = 8.3 Hz, 2H), 3.29 – 3.19 (m, 1H), 2.06 – 1.98 (m, 2H), 1.85 – 1.74 (m, 2H), 1.69 – 1.59 (m, 1H), 1.50 – 1.24 (m, 5H).

^{13}C NMR (101 MHz, $CDCl_3$) δ/ppm = 141.3(q, *J* = 1.1 Hz), 129.8, 128.0 (q, *J* = 32.3 Hz),125.7 (q, *J* = 3.8 Hz) , 124.3 (q, *f* = 271.8), 45.7, 33.2, 26.1, 25.8.

^{19}F NMR (377 MHz, $CDCl_3$) δ/ppm = -62.4.

Spectral data are in accordance with the literature.[35]

3-(Phenylthio)tetrahydrofuran (9i)

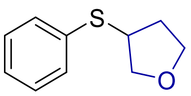

9i

Synthesized following GP 5 with dihydropyrimidine **1l** (56.3 mg, 0.2 mmol, 1 equiv.) and diphenyl disulfide (43.7 mg, 0.2 mmol, 1 equiv.). Purification by column chromatography on silica gel (*n*-pentane) afforded the product as an orange oil (13.5 mg, 0.07 mmol, 37%).

R*f* (*n*-Pentane/EtOAc = 10/1): 0.72.

^{1}H NMR (400 MHz, $CDCl_3$) δ/ppm = 7.41 – 7.36 (m, 2H), 7.30 (t, *J* = 7.6 Hz, 2H), 7.27 – 7.20 (m, 1H), 4.10 (dd, *J* = 9.2, 6.5 Hz, 1H), 3.99 – 3.91 (m, 1H), 3.90 – 3.77 (m, 2H), 3.69 (dd, *J* = 9.2, 5.5 Hz, 1H), 2.39 – 2.28 (m, 1H), 1.93 (ddt, *J* = 13.0, 7.5, 5.5 Hz, 1H).

^{13}C NMR (101 MHz, $CDCl_3$) δ/ppm = 135.7, 130.8, 129.2, 126.9, 73.7, 67.7, 44.9, 33.3.

Spectral data are in accordance with the literature.[36]

Isopropyl(4-(trifluoromethyl)phenyl)sulfane (9j)

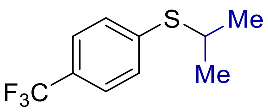

9j

Synthesized following GP 5 with dihydropyrimidine **1m** (50.7 mg, 0.2 mmol, 1 equiv.) and 1,2-bis(4-(trifluoromethyl)phenyl)disulfane (70.9 mg, 0.2 mmol, 1 equiv.). Purification by column chromatography on silica gel (*n*-pentane) afforded the product as a colorless oil (13.0 mg, 0.06 mmol, 30%).

R_f (*n*-Hexane): 0.54.

^{1}H NMR (400 MHz, $CDCl_3$) δ/ppm = 7.54 – 7.50 (m, 2H), 7.44 – 7.39 (m, 2H), 3.50 (hept, *J* = 6.7 Hz, 1H), 1.34 (d, *J* = 6.7 Hz, 6H).

^{13}C NMR (101 MHz, $CDCl_3$) δ/ppm = 141.6, 129.8, 128.1 (q, *J* = 32.7 Hz), 125.7 (q, *J* = 4.0 Hz), 124.3 (q, *J* = 271.9 Hz), 37.4, 23.1.

^{19}F NMR (377 MHz, $CDCl_3$) δ/ppm = -62.45.

Spectral data are in accordance with the literature.[37]

Cyclohexyl(*m*-tolyl)sulfane (9k)

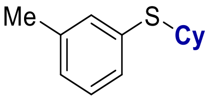

9k

Synthesized following GP 5 with dihydropyrimidine **1a** (58.7 mg, 0.2 mmol, 1 equiv.) and 1,2-di-*m*-tolyldisulfane (49.3 mg, 0.2 mmol, 1 equiv.). Purification by column chromatography on silica gel (*n*-pentane) afforded the product as a yellowish oil (27.5 mg, 0.13 mmol, 66%).

R_f (*n*-Pentane/EtOAc = 20/1): 0.62.

^{1}H NMR (400 MHz, $CDCl_3$) δ/ppm = 7.23 – 7.14 (m, 3H), 7.02 (dtd, *J* = 6.8, 1.8, 0.9 Hz, 1H), 3.09 (tt, *J* = 10.4, 3.7 Hz, 1H), 2.33 (s, 3H), 2.02 – 1.95 (m, 2H), 1.81 – 1.72 (m, 2H), 1.61 (tdd, *J* = 10.4, 4.7, 2.8 Hz, 1H), 1.45 – 1.19 (m, 5H).

^{13}C NMR (101 MHz, $CDCl_3$) δ/ppm = 138.6, 135.0, 132.7, 129.0, 128.7, 127.6, 46.7, 33.5, 26.2, 25.9, 21.5.

Spectral data are in accordance with the literature.[38]

Mechanistic investigations

1-(But-3-en-1-yl)-4-methoxybenzene (10)

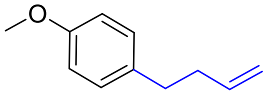

10

Synthesized following GP 3a with ethyl 6-(cyclopropylmethyl)-2-(dimethylamino)-4-methyl-1,6-dihydropyrimidine-5-carboxylate **1u** (132.6 mg, 0.5 mmol, 2.0 equiv.) and 1-iodo-4-methoxybenzene (58.5 mg, 0.25 mmol, 1 equiv.). Purification by column chromatography on silica gel afforded the product **10** as a colorless oil (5.7 mg, 0.035 mmol, 7%).

R*f* (*n*-pentane/EtOAc = 10/1): 0.55.

^{1}H NMR (400 MHz, $CDCl_3$) δ/ppm = 7.11 (d, *J* = 8.6 Hz, 2H), 6.84 (d, *J* = 8.6 Hz, 2H), 5.92 – 5.80 (m, 1H), 5.04 (dd, *J* = 17.0 Hz, *J* = 1.8 Hz, 1H), 4.98 (dd, *J* = 10.2 Hz, *J* = 2.0 Hz, 1H), 3.80 (s, 3H), 2.69 – 2.63 (m, 2H), 2.35 (dd, *J* = 7.8 Hz, *J* = 1.4 Hz, 2H).

^{13}C NMR (101 MHz, $CDCl_3$) δ/ppm = 157.8, 138.3, 134.1, 129.4, 114.9, 113.8, 55.3, 35.9, 34.6.

Spectral data are in accordance with the literature.[16]

GC-MS data of TEMPO Adduct formation

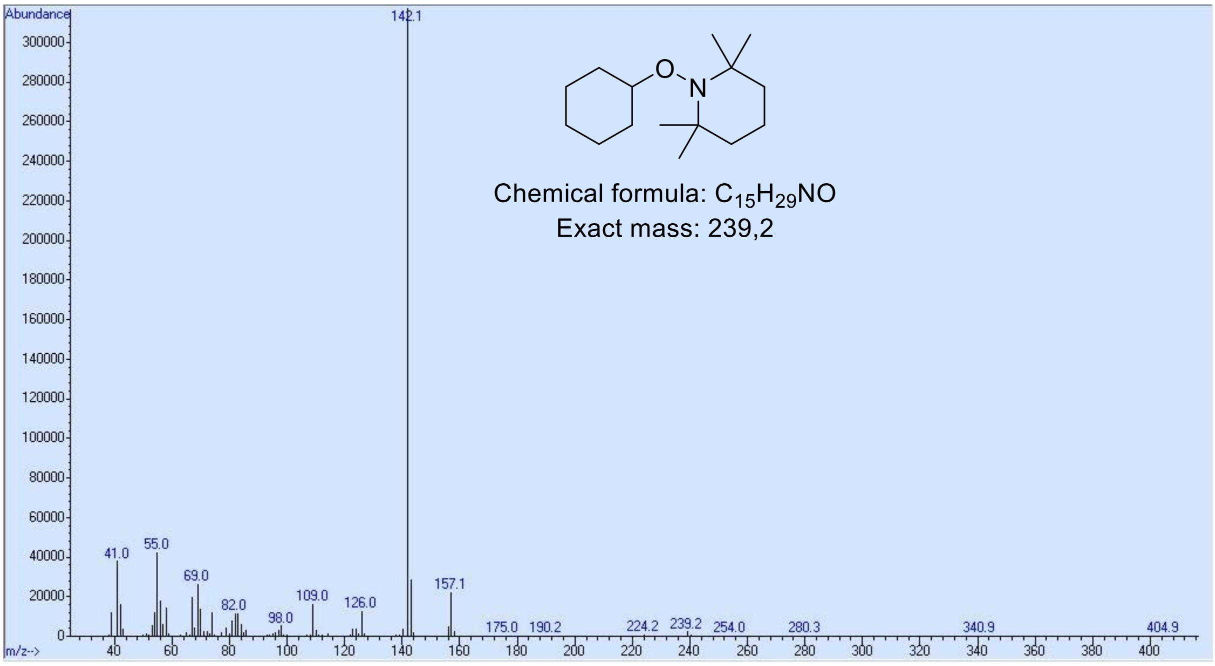

GC/EI-MS m/z (rel. int.): 239 (M^+, 1), 158 (1), 157 (7), 156 (2), 144 (1), 143 (9), 142 (100), 140 (1), 126 (4), 124 (1), 123 (1), 110 (1), 109 (5), 98 (2), 97 (1), 96 (1), 86 (1), 85 (1), 84 (2), 83

(4), 82 (4), 81 (3), 80 (1), 79 (2), 78 (1), 77 (1), 74 (4), 73 (1), 72 (1), 71 (1), 70 (5), 69 (9), 68 (1), 67 (7), 65 (1), 59 (1), 58 (5), 57 (2), 56 (6), 55 (14), 54 (4), 53 (2), 52 (1), 51 (1), 44 (3), 43 (3), 42 (6), 41 (13), 40 (1), 39 (4).

HRMS Data of TEMPO adduct formations

TEMPO adduct after dual PC/Ni-catalyzed cross-coupling with DHPym 1a

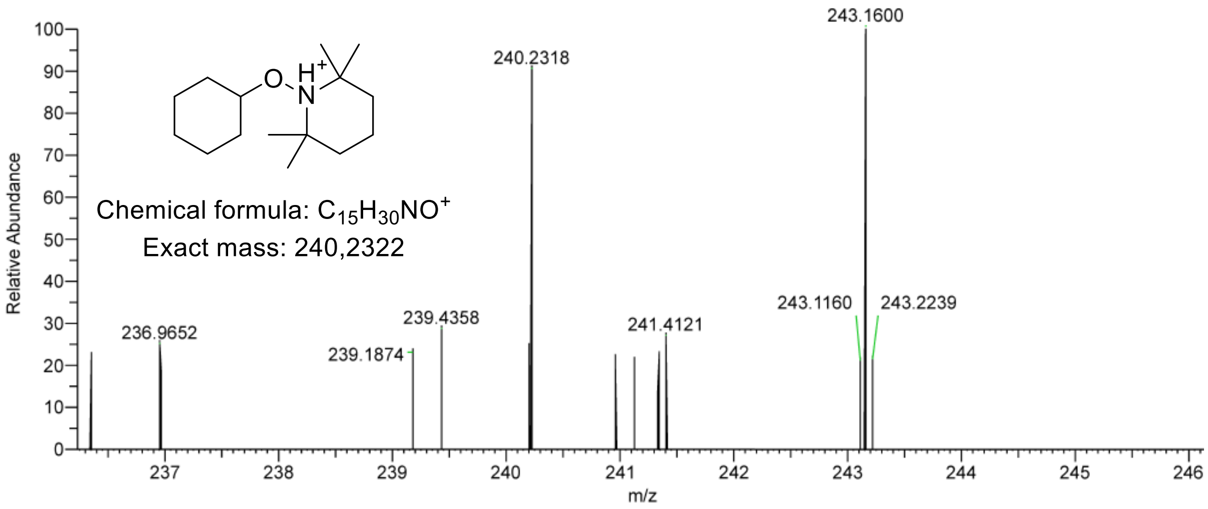

TEMPO adduct after PC-free Ni-catalyzed cross-coupling with DHPym 1a

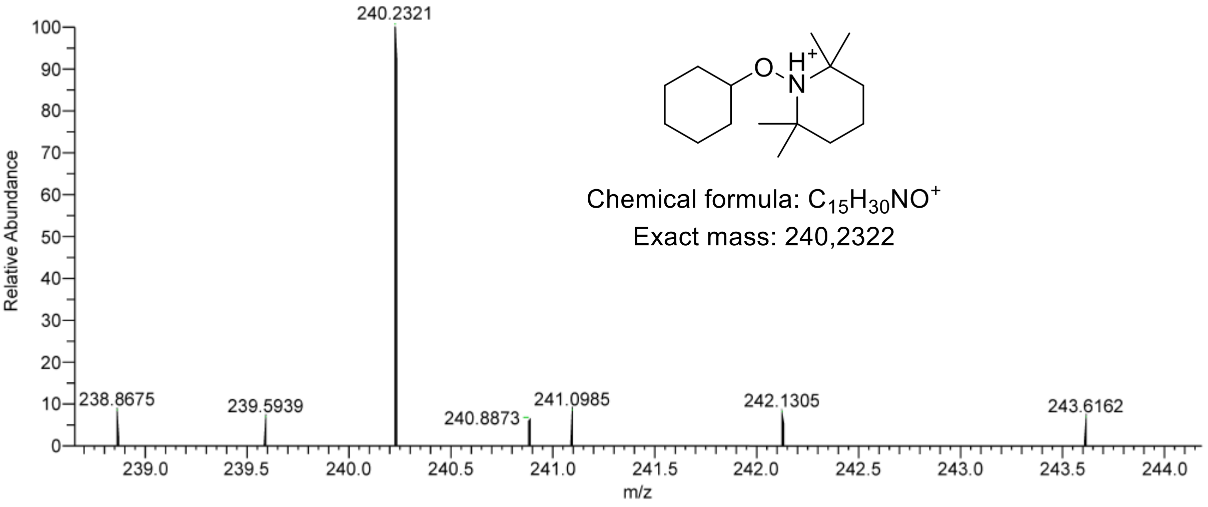

TEMPO adduct after Ni-catalyzed Giese addition

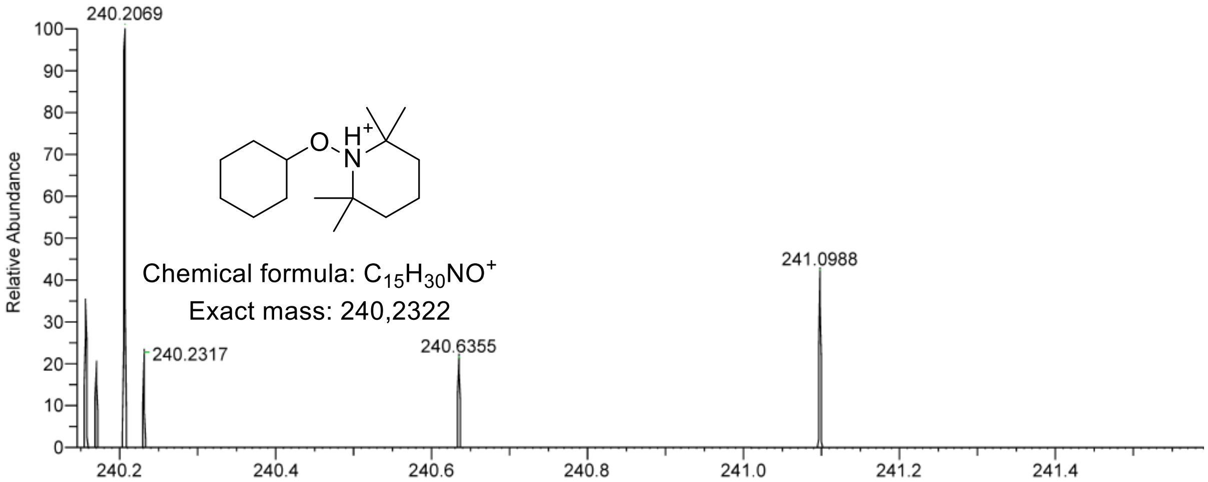

TEMPO adduct after Cu-catalyzed disulfide radical addition

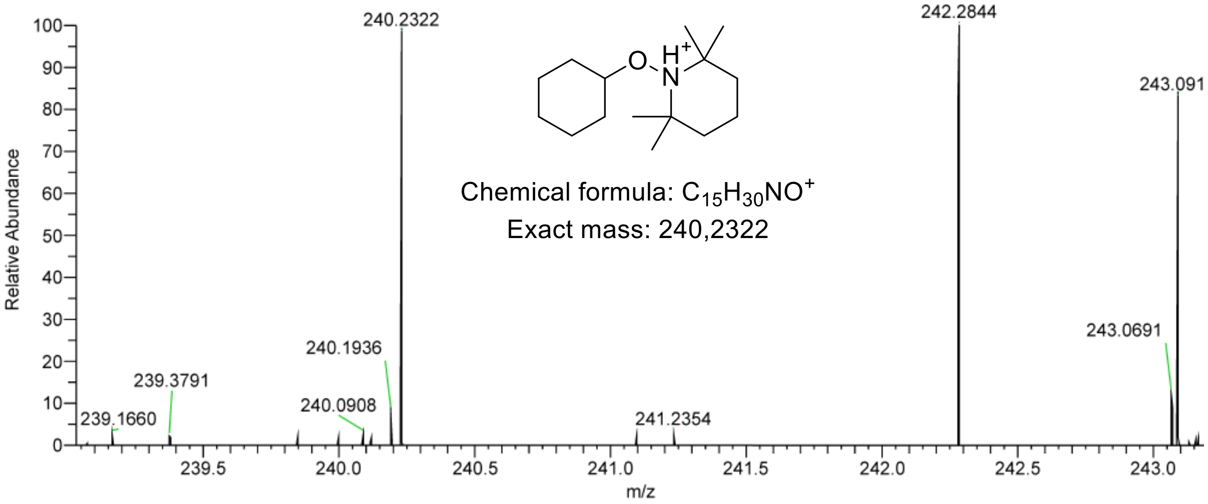

TEMPO adduct after Cu-catalyzed reductive alkylation of nitrobenzenes

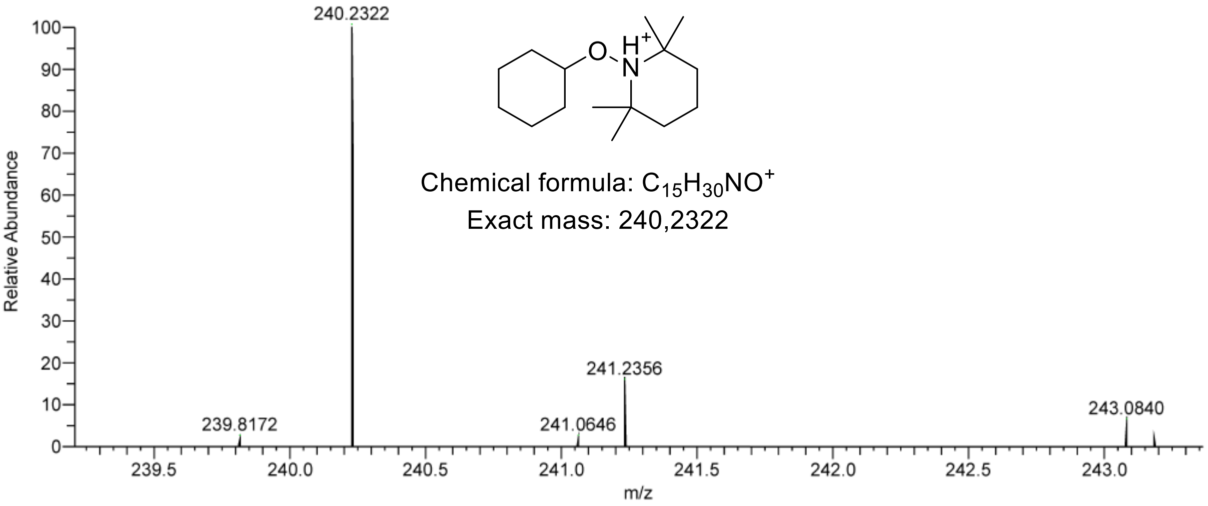

Conversion profile measurements

Competition reaction with Hantzsch ester and dihydropyrimidine for Ni-catalyzed cross-coupling

A

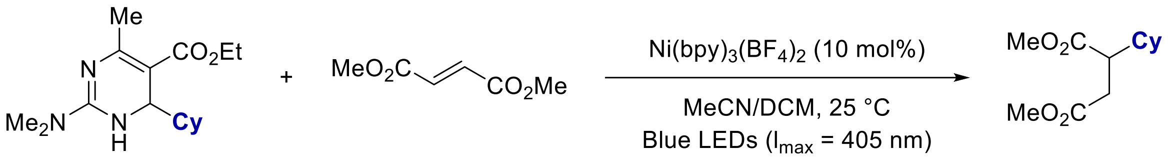

1a **2a** **3a**

B

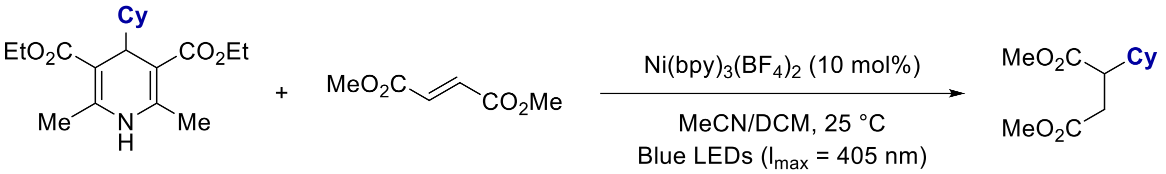

Cy-DHP **2a** **3a**

For the reaction A, to an oven-dried screw-capped Schlenk tube equipped with a magnetic stir bar was added $Ni(bpy)_3(BF_4)_2$ (0.05 mmol, 0.1 equiv., 35 mg) and dihydropyrimidine **1a** (220 mg, 0.75 mmol, 1.5 equiv.). After evacuating and refilling with argon for at least three cycles, dry MeCN (1 mL) and dry DCM (1 mL) was added, followed by dimethyl fumarate (72 mg, 0.5 mmol, 1.0 equiv.) and decane as internal standard and the reaction was degassed for 5 min.

For the reaction B, to an oven-dried screw-capped Schlenk tube equipped with a magnetic stir bar was added $Ni(bpy)_3(BF_4)_2$ (0.05 mmol, 0.1 equiv., 35 mg) and Cy-DHP (252 mg, 0.75 mmol, 1.5 equiv.). After evacuating and refilling with argon for at least three cycles, dry MeCN (1 mL) and dry DCM (1 mL) was added, followed by dimethyl fumarate (72 mg, 0.5 mmol, 1.0 equiv.) and decane as internal standard and the reaction was degassed for 5 min.

Both the reaction then was allowed to stir in photoreactor with wavelength 450 nm at the same time. The samples were taken every 30 min for GC measurements.

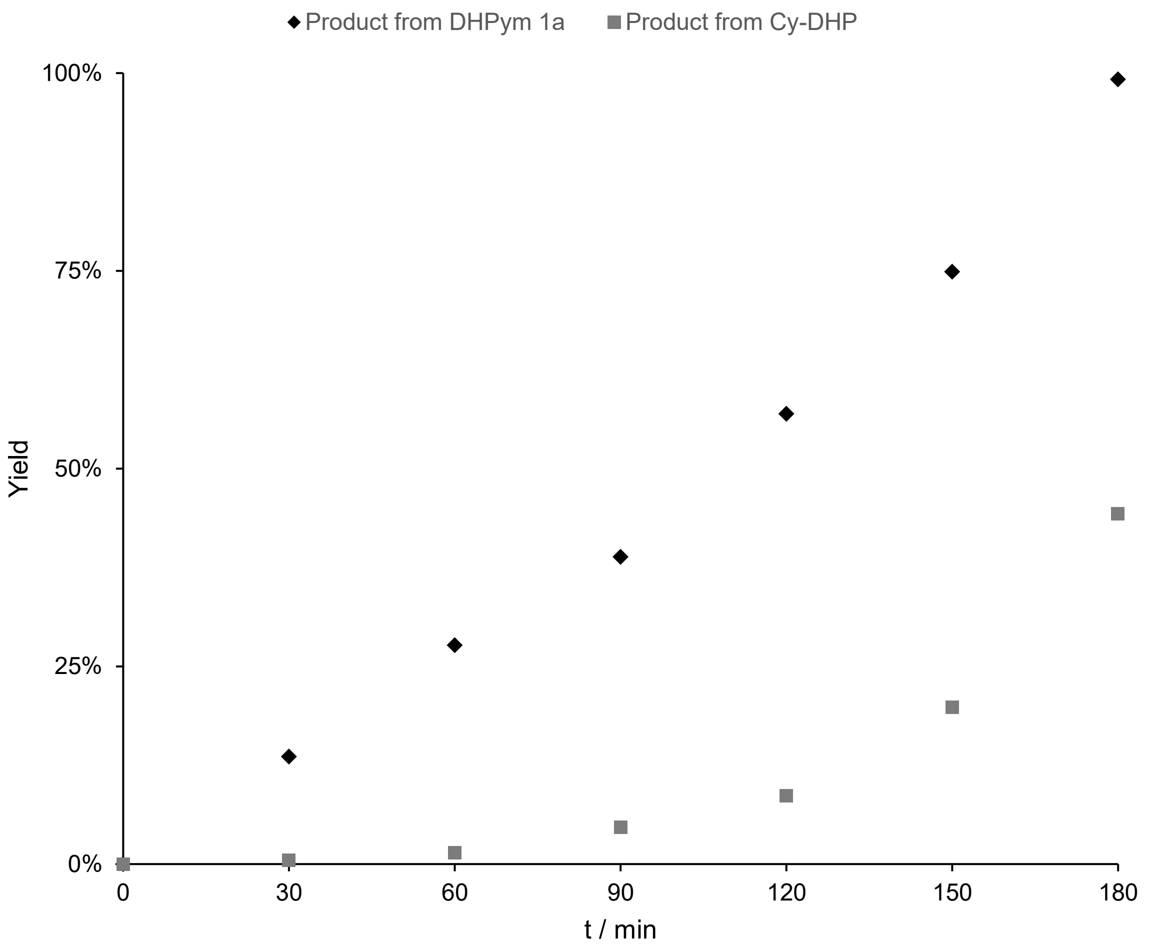

Competition reaction with Hantzsch ester and dihydropyrimidine for Ni-catalyzed cross-coupling in the same flask

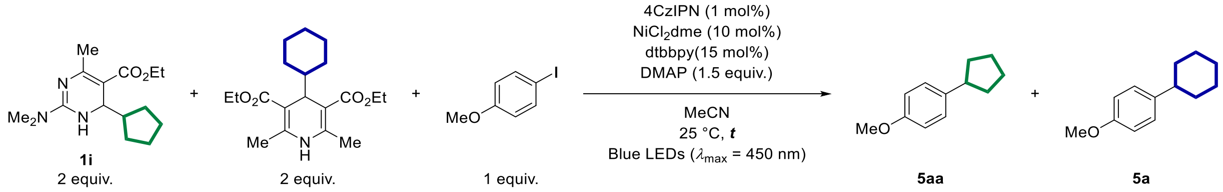

For the competition experiment, iodoanisole (46.8 mg, 0.2 mmol, 1 equiv.), Hantzsch ester (134.1 mg, 0.4 mmol, 2 equiv.), dihydropyrimidine (**1i**) (111.7 mg, 0.4 mmol, 2 equiv.), 4CzIPN (1 mol%), $NiCl_2$dme (10 mol%), dtbbpy (15 mol%), DMAP (1.5 equiv.) were added to the oven dried Schlenk tube. After evacuating and refilling with argon at least three cycles, dry MeCN (2 mL) and decane as internal standard was added and the reaction is degassed for 5 min. The reaction then was allowed to stir in photoreactor with wavelength 450 nm. The samples were taken every 10 min in the first hour then with interval of 30 min for GC measurements.

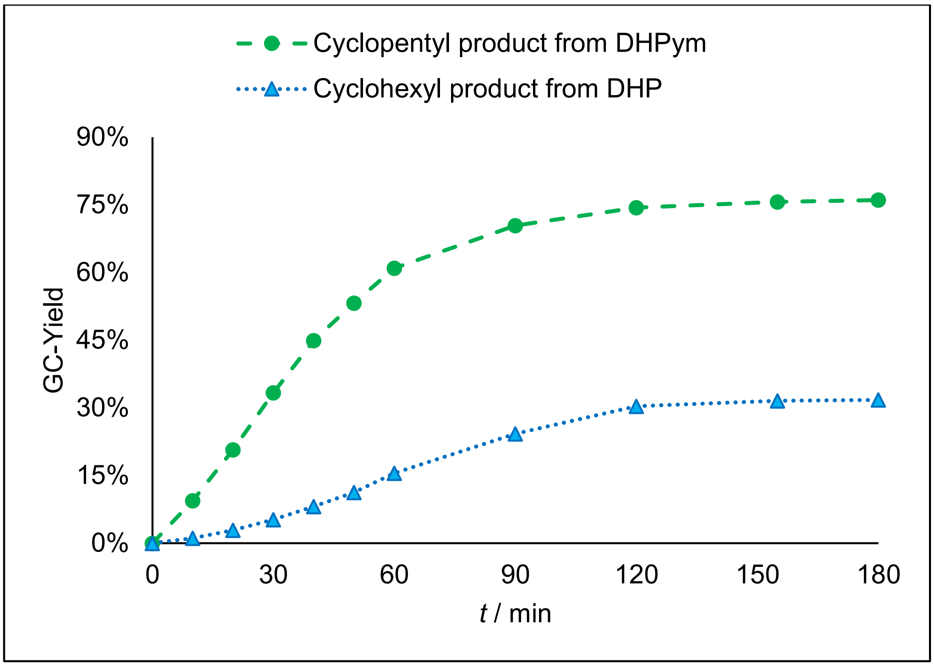

Reverse competition reaction with Hantzsch ester and dihydropyrimidine for Ni-catalyzed cross-coupling in the same flask

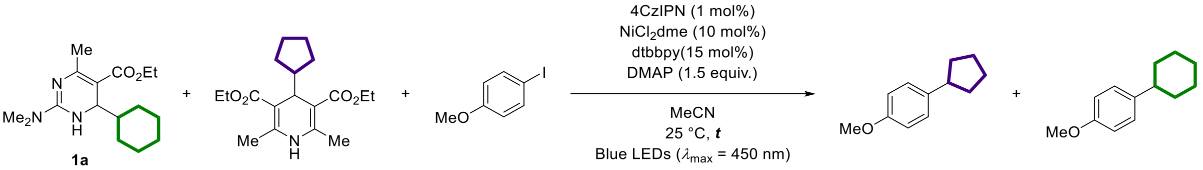

For the competition experiment, iodoanisole (46.8 mg, 0.2 mmol, 1 equiv.), cyclopentyl-substituted Hantzsch ester (128.5 mg, 0.4 mmol, 2 equiv.), dihydropyrimidine (**1a**) (117.3 mg, 0.4 mmol, 2 equiv.), 4CzIPN (1 mol%), $NiCl_2$dme (10 mol%), dtbbpy (15 mol%), DMAP (1.5 equiv.) were added to the oven dried Schlenk tube. After evacuating and refilling with argon at least three cycles, dry MeCN (2 mL) and decane as internal standard was added and the reaction is degassed for 5 min. The reaction then was allowed to stir in photoreactor with wavelength 450 nm. The samples were taken every 10 min in the first hour then with interval of 30 min for GC measurements

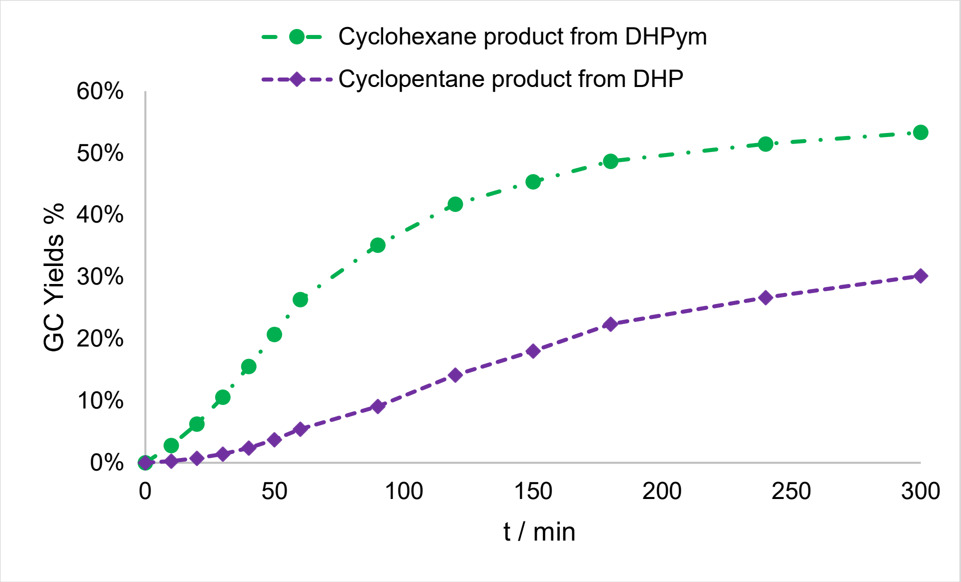

Competition reaction with Hantzsch ester and dihydropyrimidine for Ni-catalyzed cross-coupling in separate flasks

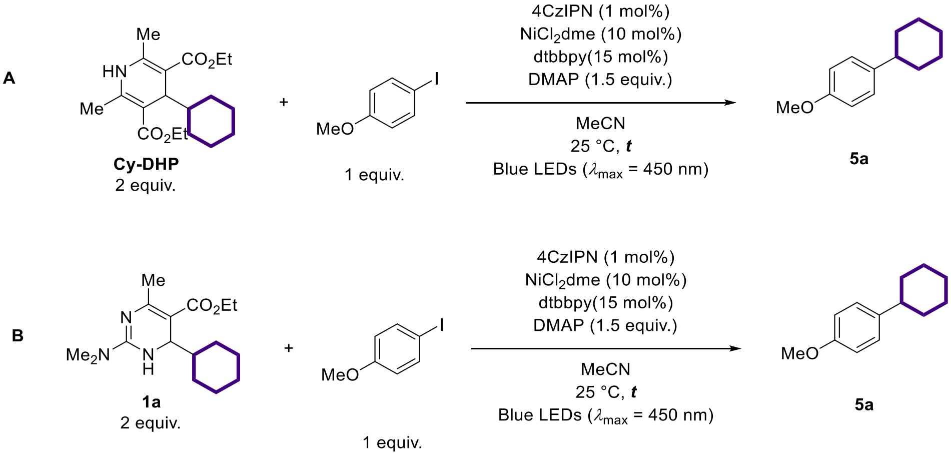

For the reaction A, iodoanisole (46.8 mg, 0.2 mmol, 1 equiv.), Hantzsch ester (134.1 mg, 0.4 mmol, 2 equiv.), 4CzIPN (1 mol%), $NiCl_2dme$ (10 mol%), dtbbpy (15 mol%), DMAP (1.5 equiv.) were added to the oven dried Schlenk tube. After evacuating and refilling with argon at least three cycles, dry MeCN (2 mL) and decane as internal standard was added and the reaction is degassed for 5 min.

For the reaction B, iodoanisole (46.8 mg, 0.2 mmol, 1 equiv.), dihydropyrimidine (**1a**) (117.3 mg, 0.4 mmol, 2 equiv.), 4CzIPN (1 mol%), $NiCl_2dme$ (10 mol%), dtbbpy (15 mol%), DMAP (1.5 equiv.) were added to the oven dried Schlenk tube. After evacuating and refilling with argon at least three cycles, dry MeCN (2 mL) and decane as internal standard was added and the reaction is degassed for 5 min.

Both the reaction then was allowed to stir in photoreactor with wavelength 450 nm at the same time. The samples were taken every 10 min in the first hour then with interval of 30 min for GC measurements

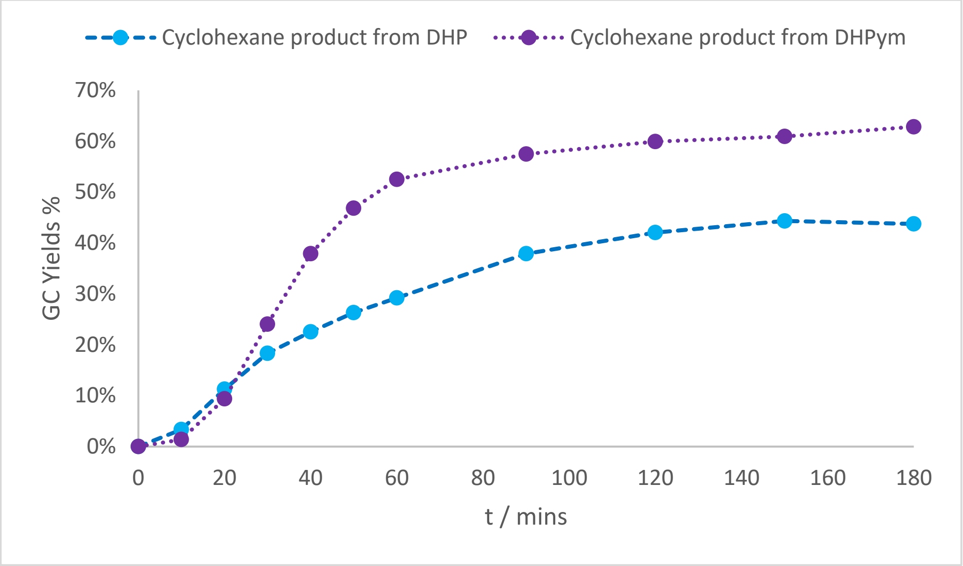

Competition reaction with 2-cyclohexyl-2-methyl-2,3-dihydroquinazolin-4(1H)-one and dihydropyrimidine

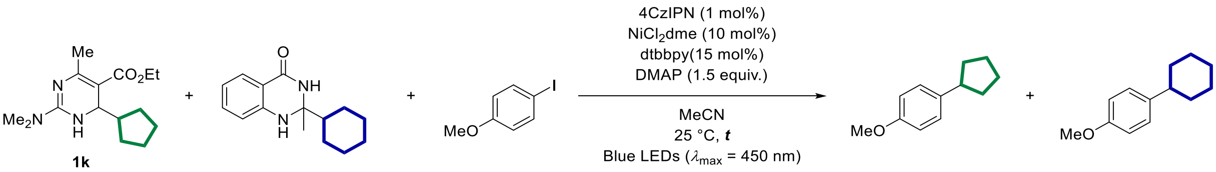

For the competition experiment, iodoanisole (46.8 mg, 0.2 mmol, 1 equiv.), 2-cyclohexyl-2-methyl-2,3-dihydroquinazolin-4(1H)-one (97.7 mg, 0.4 mmol, 2 equiv.), dihydropyrimidine (**1k**) (111.7 mg, 0.4 mmol, 2 equiv.), 4CzIPN (1 mol%), $NiCl_2$dme (10 mol%), dtbbpy (15 mol%), DMAP (1.5 equiv.) were added to the oven dried Schlenk tube. After evacuating and refilling with argon at least three cycles, dry MeCN (2 mL) and decane as internal standard was added and the reaction is degassed for 5 min. The reaction then was allowed to stir in photoreactor with wavelength 450 nm. The samples were taken every 10 min in the first hour then with interval of 30 min for GC measurements

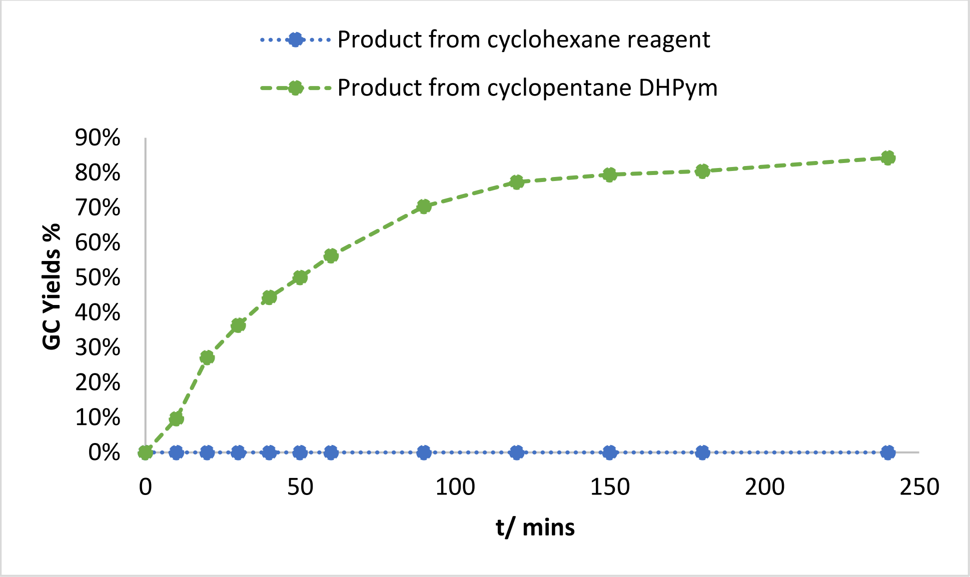

Competition reaction with cyclohexyltrifluoroborate and dihydropyrimidine

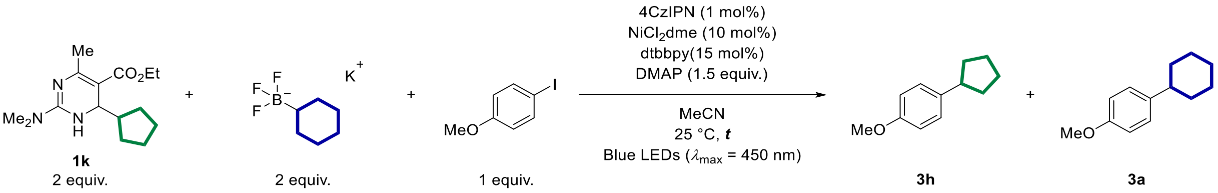

For the competition experiment, iodoanisole (46.8 mg, 0.2 mmol, 1 equiv.), cyclohexyltrifluoroborate (76.0 mg, 0.4 mmol, 2 equiv.), dihydropyrimidine (**1k**) (111.7 mg, 0.4 mmol, 2 equiv.), 4CzIPN (1 mol%), $NiCl_2$dme (10 mol%), dtbbpy (15 mol%), DMAP (1.5 equiv.) were added to the oven dried Schlenk tube. After evacuating and refilling with argon at least three cycles, dry MeCN (2 mL) and decane as internal standard was added and the reaction is degassed for 5 min. The reaction then was allowed to stir in photoreactor with wavelength 450 nm. The samples were taken every 10 min in the first hour then with interval of 30 min for GC measurements

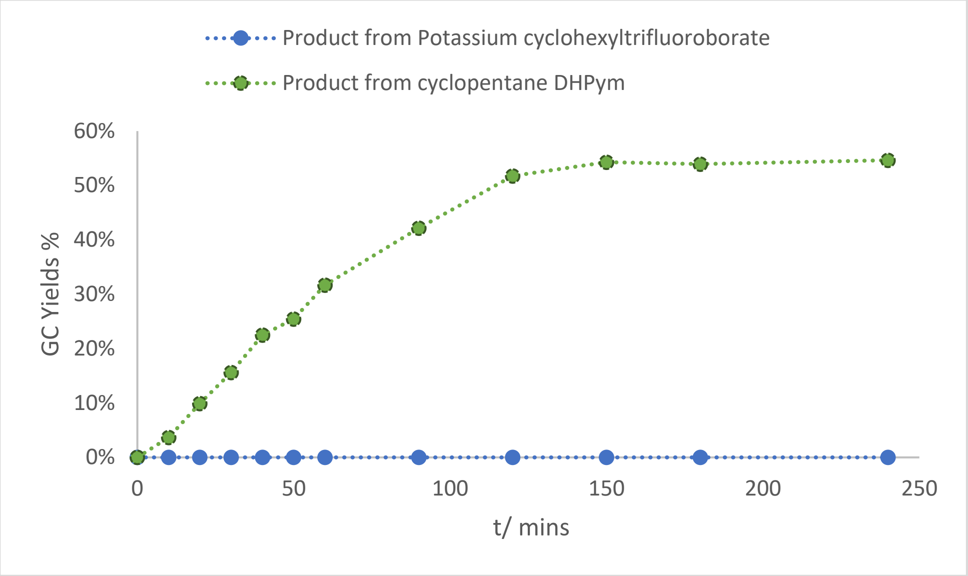

Pyrimidine byproduct

Ethyl 2-(dimethylamino)-4-methylpyrimidine-5-carboxylate

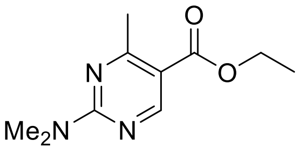

The pyrimidine byproduct after C–C bond cleavage was observed for all radical reactions involving dimethylamino-substituted DHPym reagents such as **1a**.

R*f* (*n*-pentane/EtOAc = 5/1): 0.48.

^{1}H NMR (400 MHz, $CDCl_3$) δ 8.68 (s, 1H), 4.23 – 4.14 (m, 2H), 3.12 (s, 6H), 2.53 (s, 3H), 1.25 (m, 3H).

^{13}C NMR (101 MHz, $CDCl_3$) δ 169.0, 165.9, 161.7, 160.6, 110.7, 60.0, 36.8, 24.9, 14.2.

IR (ATR, cm-1): 2980, 2931, 2906, 2871, 2797, 1712, 1709, 1580, 1544, 1519, 1518, 1478, 1444, 1406, 1389, 1365, 1356, 1281, 1247, 1215, 1212, 1095, 1084, 1082, 1032, 971, 893, 834, 801, 763, 729, 611, 577, 427, 399.

HR-MS (ESI): m/z calcd for $[M+Na]^+$ 210.1237, found 210.1239.

UV-vis spectroscopy

UV-vis spectra were recorded on a JASCO V-770 UV-Visible/NIR spectrophotometer operated using the software Spectra Manager™. All measurements were performed in $CHCl_3$.

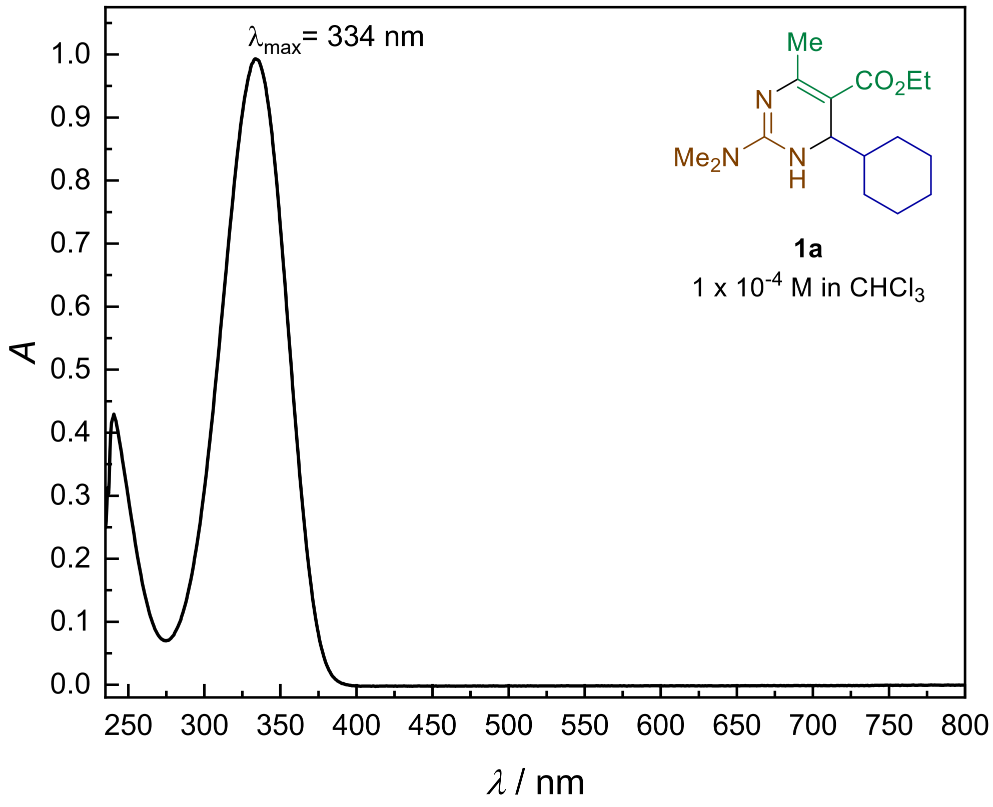

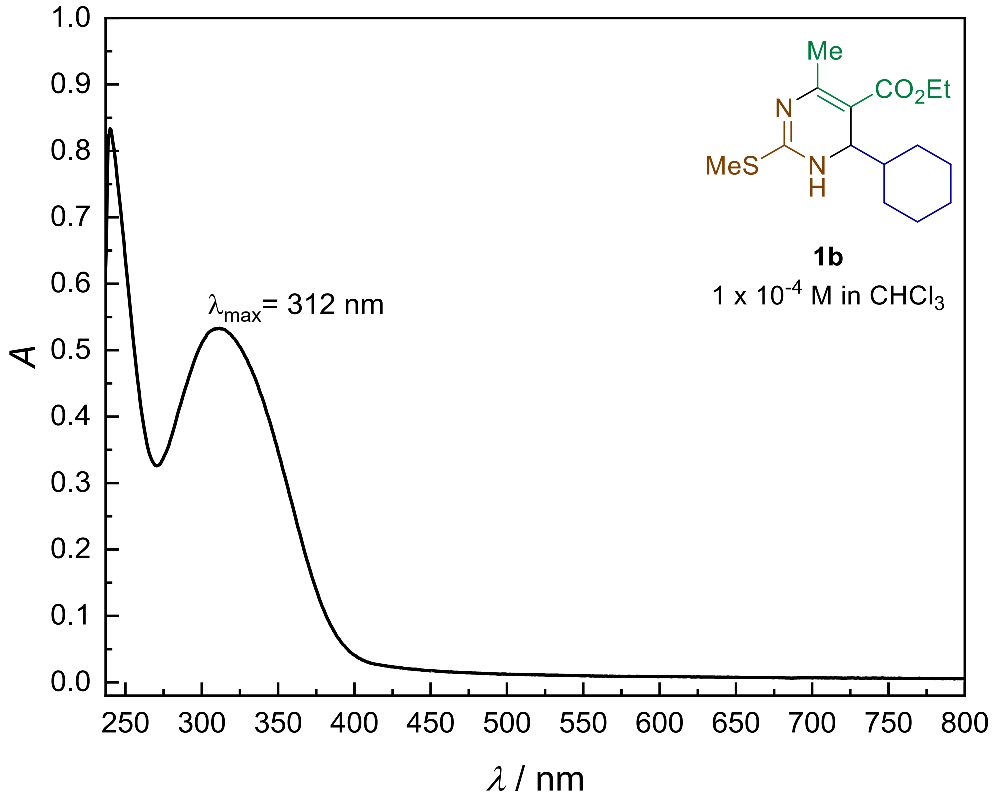

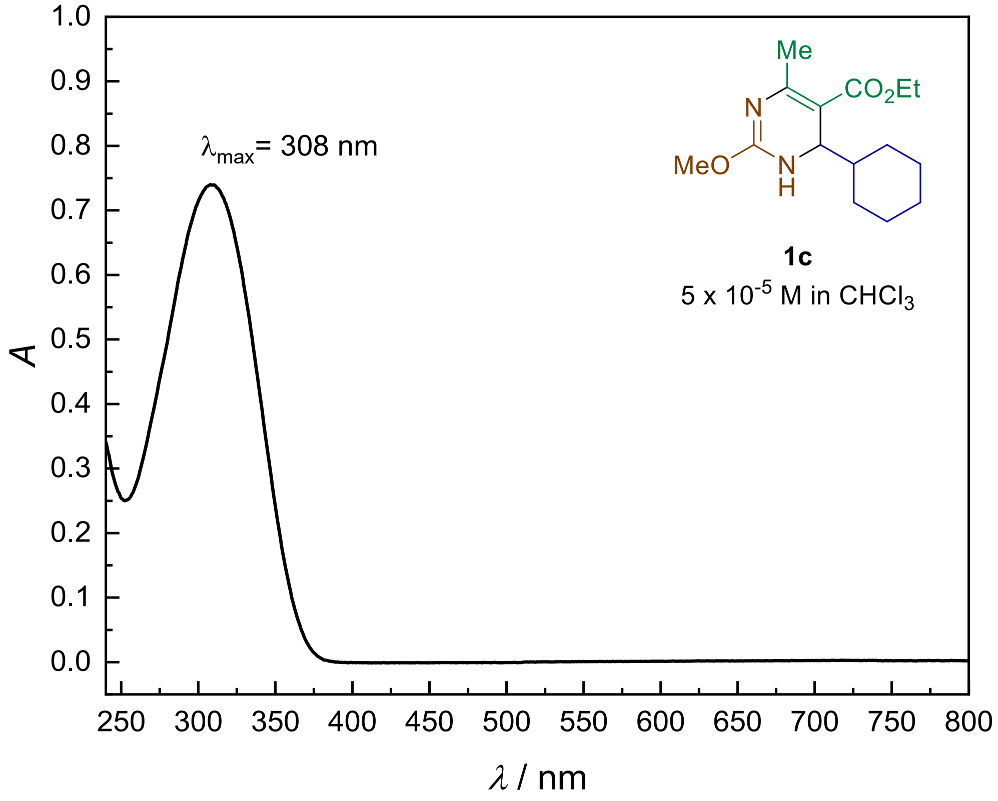

1.0
0.9
0.8
0.7
0.6
0.5
0.4
0.3
0.2
0.1
0.0
A
λmax= 308 nm
Me
CO2Et
N
MeO
N
H
1c
5 x 10-5 M in CHCl3
250
300
350
400
450
500
550
600
650
700
750
800
λ / nm

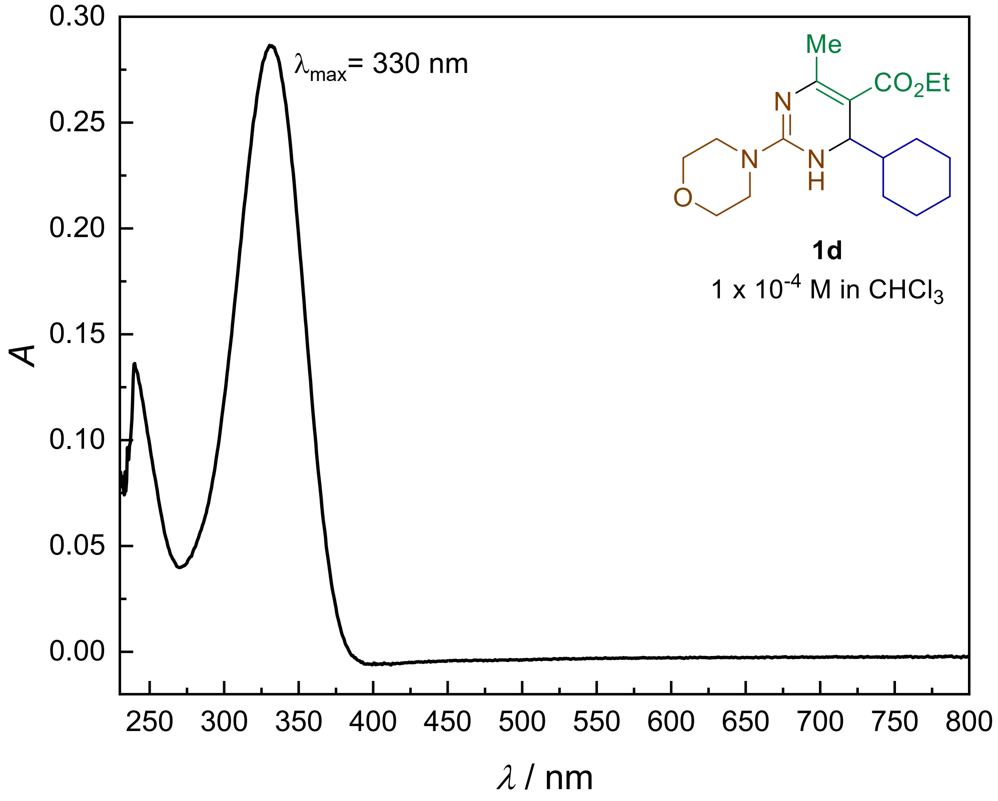

0.30
0.25
0.20
0.15
0.10
0.05
0.00
A
λmax= 330 nm
Me
CO2Et
N
N
O
N
H
1d
1 x 10-4 M in CHCl3
250
300
350
400
450
500
550
600
650
700
750
800
λ / nm

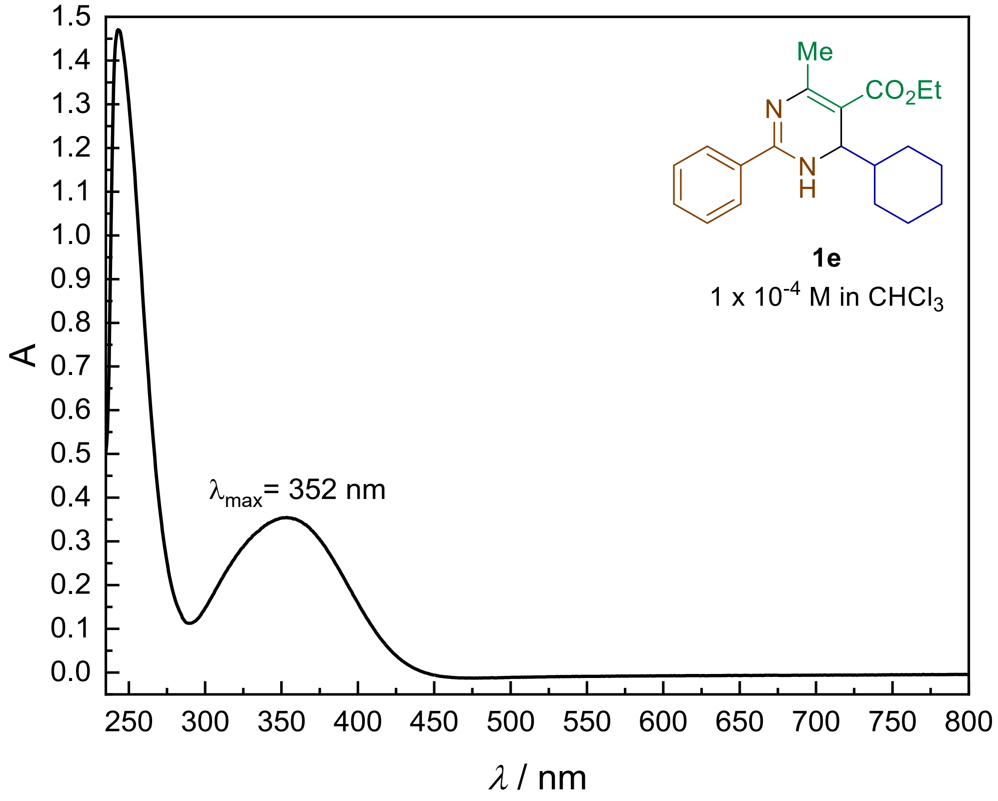

Me
CO_2Et
N
N
H
1e
1 x 10^{-4} M in $CHCl_3$
λ_{max}= 352 nm
A
λ / nm

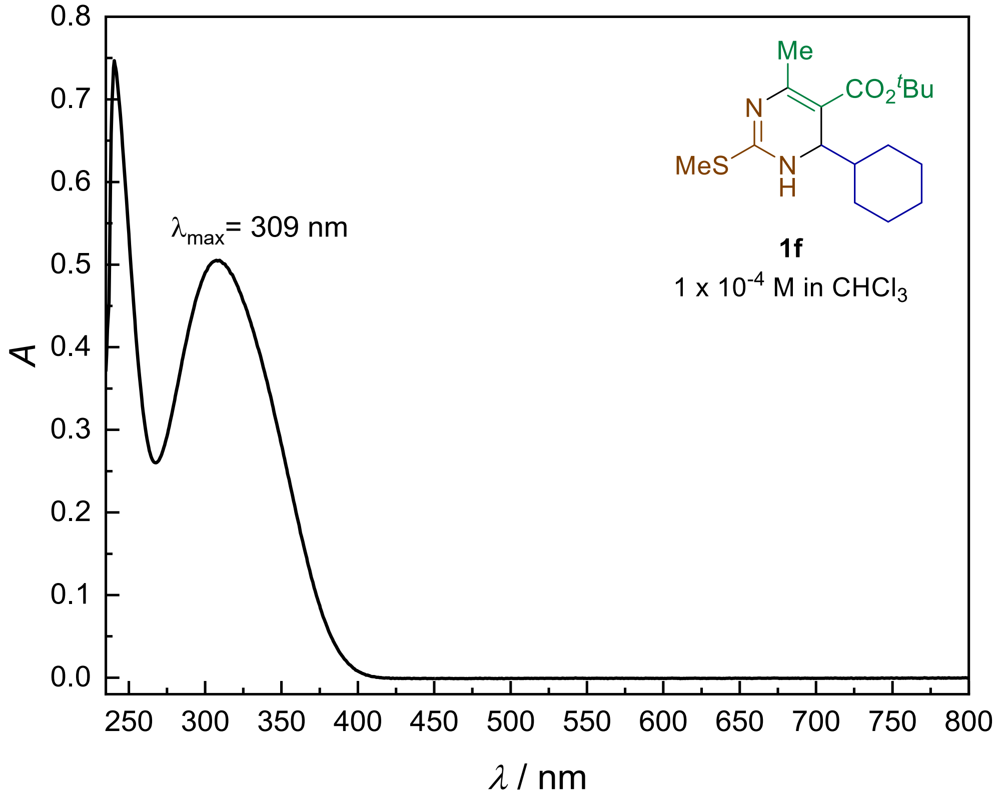

Me
$CO_2{}^tBu$
N
MeS
N
H
1f
1 x 10^{-4} M in $CHCl_3$
λ_{max}= 309 nm
A
λ / nm

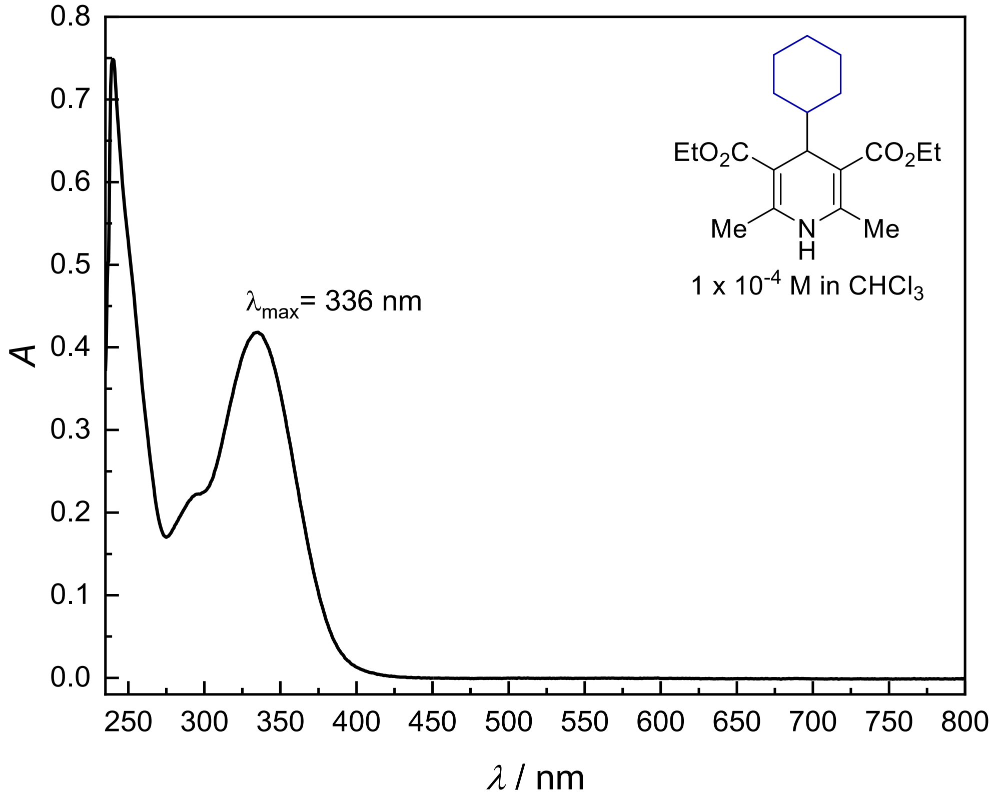

A
λ / nm
λmax= 336 nm
EtO2C
CO2Et
Me
N
H
Me
1 x 10-4 M in CHCl3

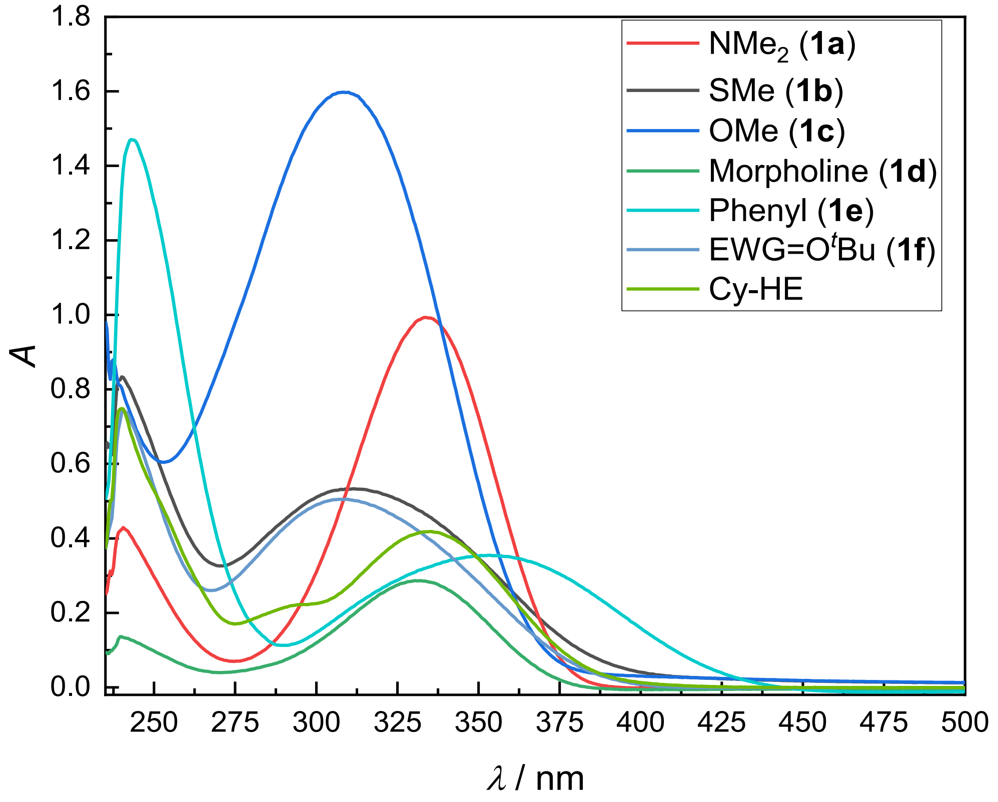

A
λ / nm
NMe2 (1a)
SMe (1b)
OMe (1c)
Morpholine (1d)
Phenyl (1e)
EWG=OtBu (1f)
Cy-HE

In analogy to recent literature on Hantzsch ester anions,[39,40] UV/vis analysis for the possible formation of DHPym anions was conducted with a $2 \cdot 10^{-4}$ M concentration of DHPym **1a** in DMSO. An excess of different bases was added to the solution, mixed by quick shaking and directly followed by UV/vis absorption measurements. Only KOtBu and KOH led to a red shift of the UV/vis absorption peak from 339 nm in DMSO to 366 nm, indicating that strong bases can lead to a DHPym anion. DMAP however, which is used for the cross-coupling of iodobenzenes, did not lead to a redshift of the DHPym absorption peak.

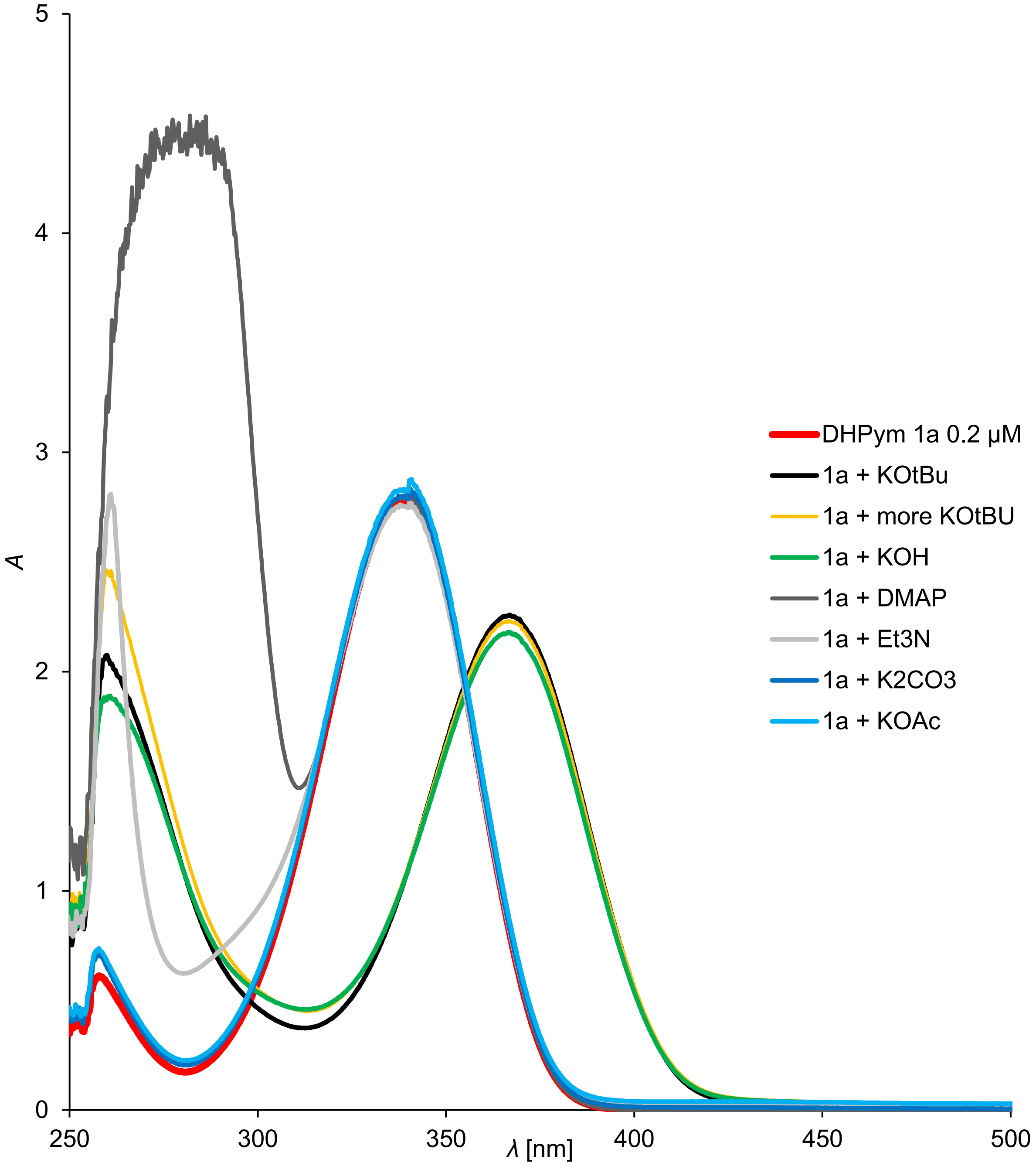

Luminescence emission spectroscopy

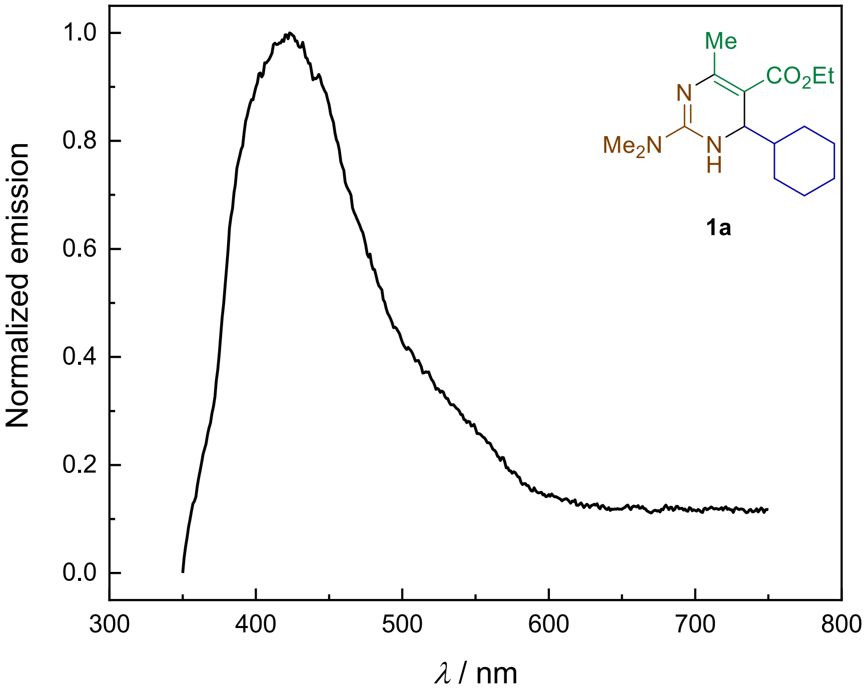

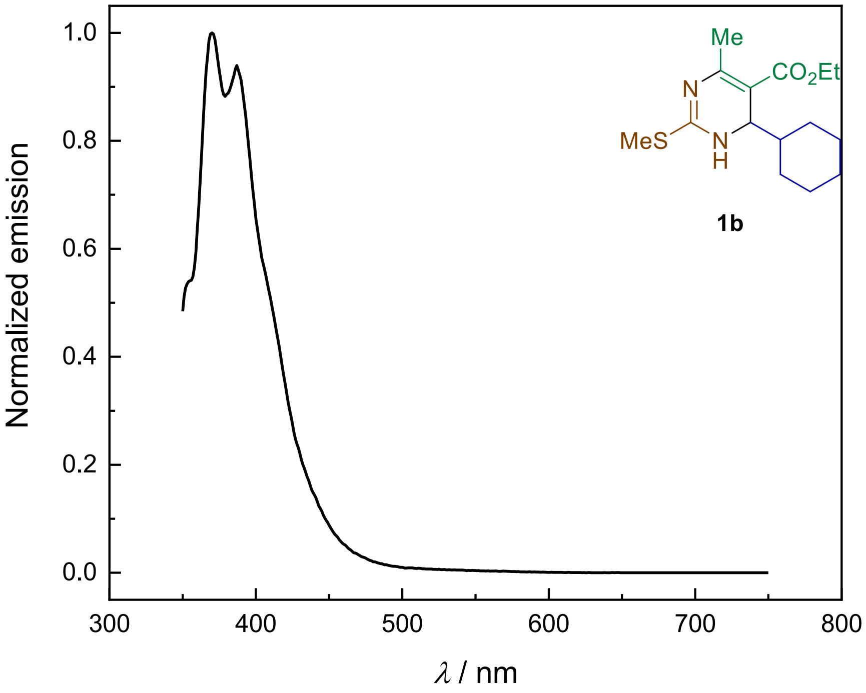

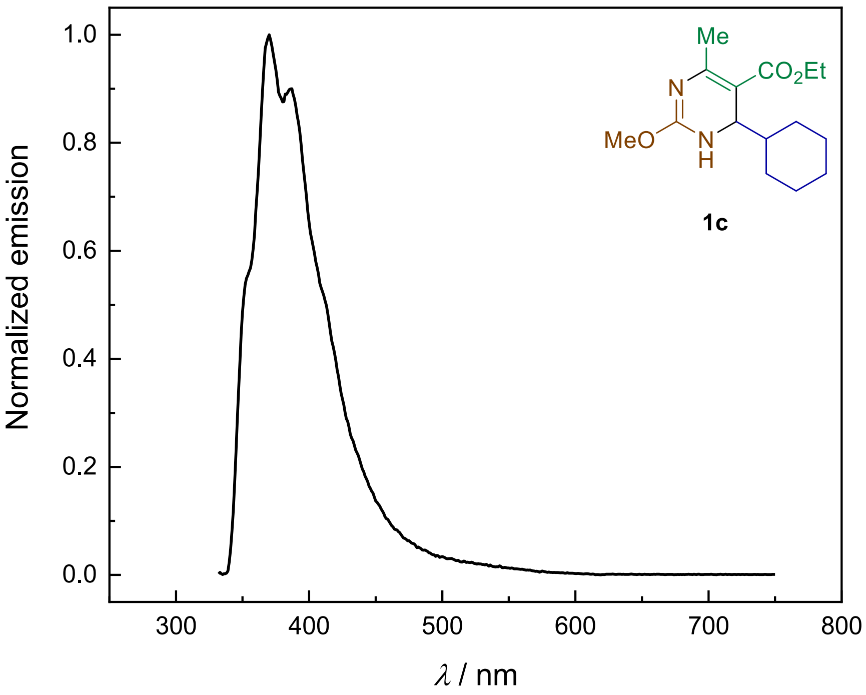

Me
CO2Et
N
MeO
N
H
1c
Normalized emission
λ / nm

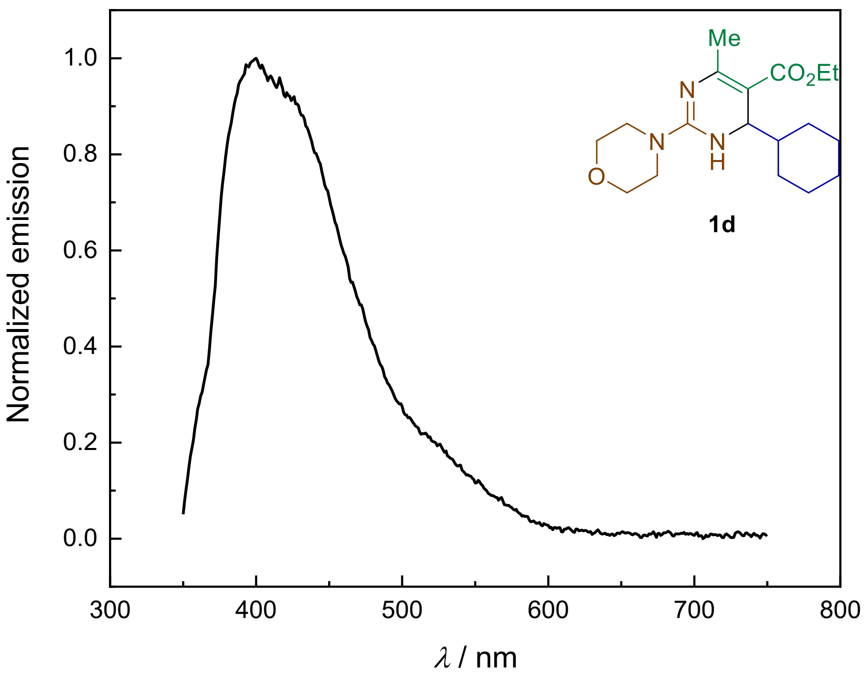

Me
CO2Et
N
N
O
N
H
1d
Normalized emission
λ / nm

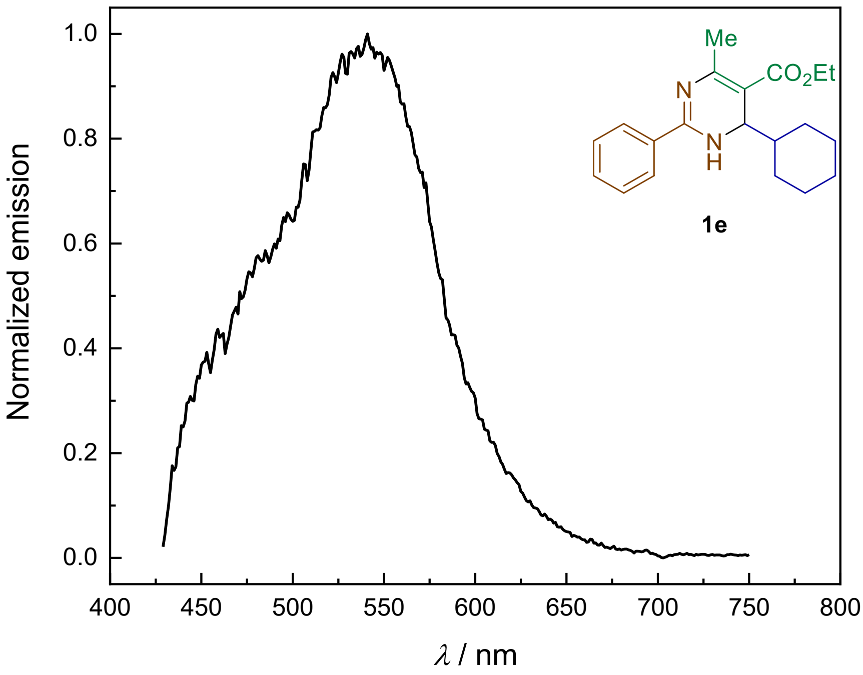
1.0
0.8
0.6
0.4
0.2
0.0
Normalized emission
400
450
500
550
600
650
700
750
800
λ / nm
Me
CO_2Et
N
N
H
1e

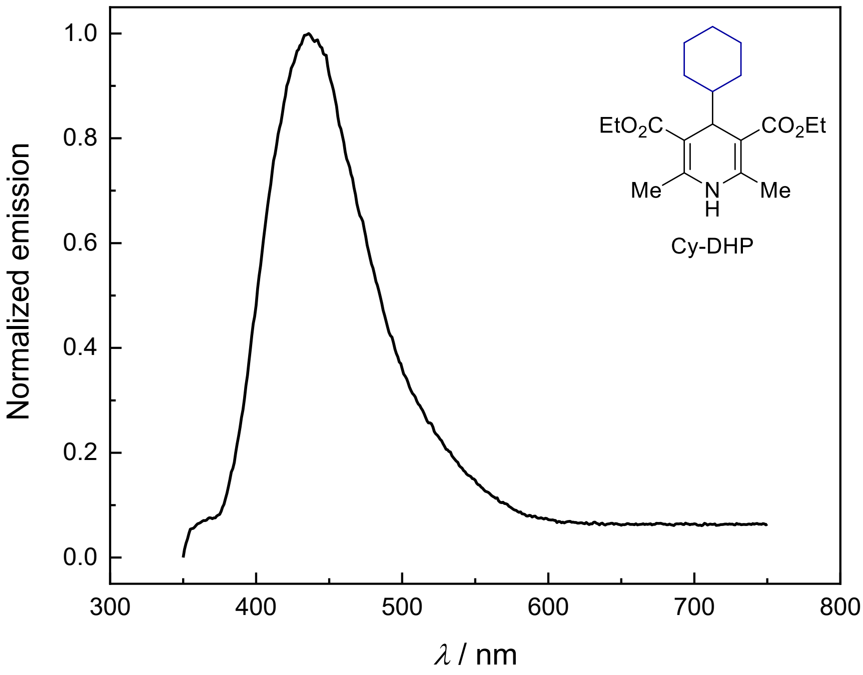
1.0
0.8
0.6
0.4
0.2
0.0
Normalized emission
300
400
500
600
700
800
λ / nm
EtO_2C
CO_2Et
Me
N
H
Me
Cy-DHP

Cyclic voltammetry

Cyclic voltammetry experiments were conducted to determine the oxidation potential (half-peak potential) of our dihydropyrimidine reagents which span from +0.68 to 1.08 V, markedly lower than the typical E^{ox} range of other heterocycles (0.86-1.20 V). The lowest potential could be achieved with NMe_2, from which it can be assumed that this specific reagent being the most reactive in our dihydropyrimidine library bearing the potential of overcoming limitations in existing methods.

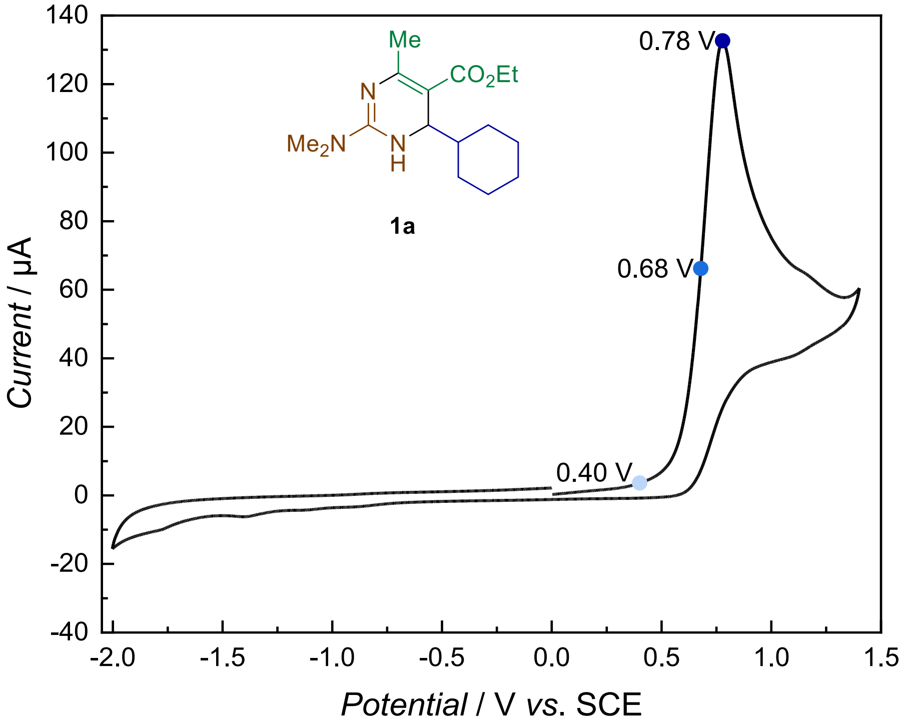

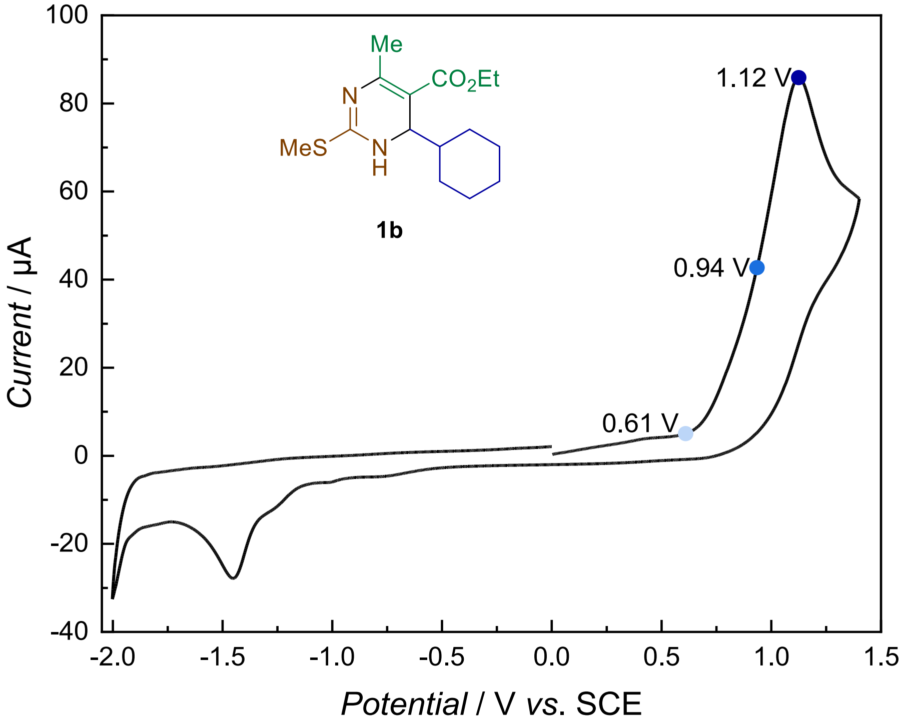

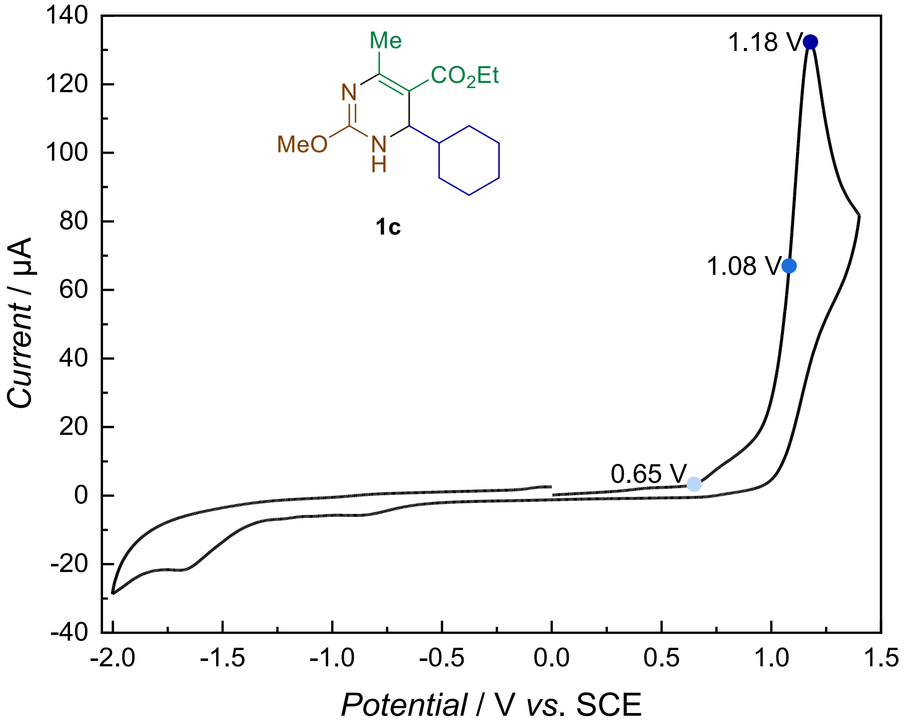

Me
CO2Et
N
MeO
N
H
1c
1.18 V
1.08 V
0.65 V
Current / μA
Potential / V vs. SCE

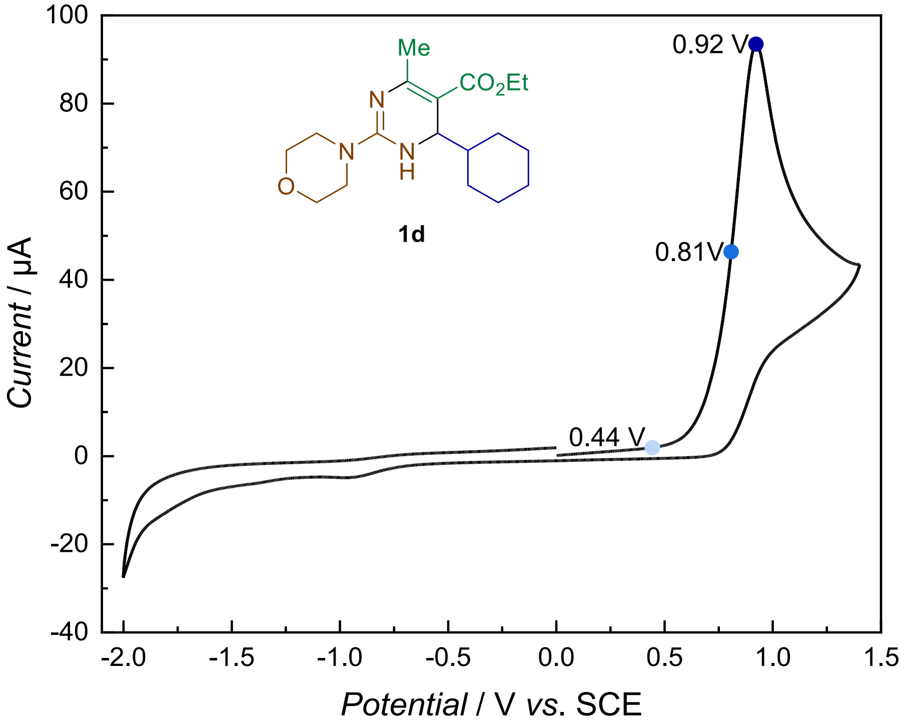

Me
CO2Et
N
N
O
N
H
1d
0.92 V
0.81V
0.44 V
Current / μA
Potential / V vs. SCE

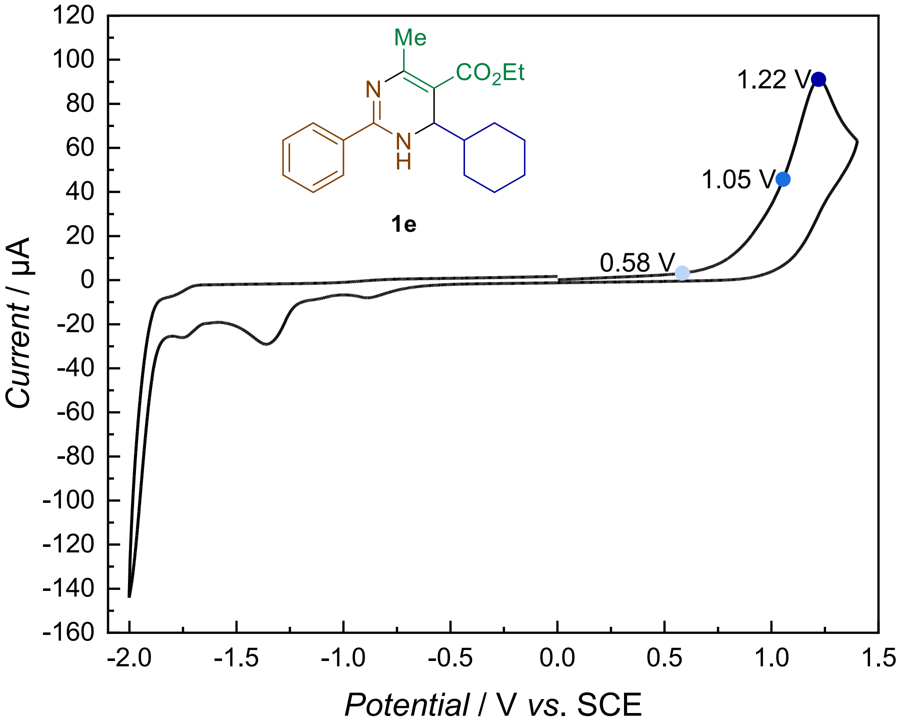

Me
CO_2Et
N
N
H
1e
1.22 V
1.05 V
0.58 V
Current / μA
Potential / V vs. SCE

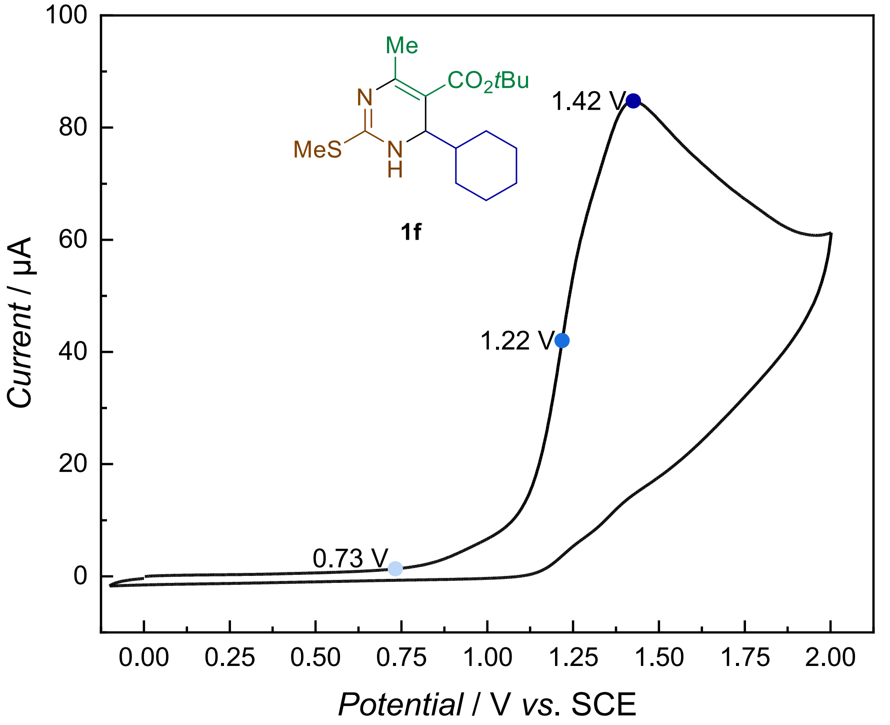

Me
CO_2tBu
N
MeS
N
H
1f
1.42 V
1.22 V
0.73 V
Current / μA
Potential / V vs. SCE

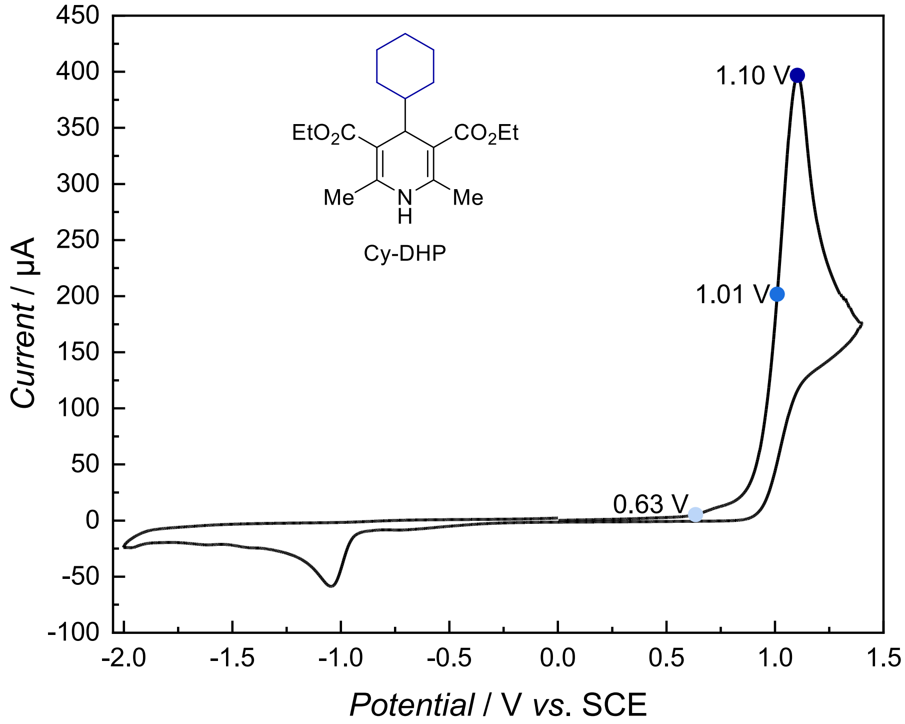
EtO2C
CO2Et
Me
N
H
Me
Cy-DHP
1.10 V
1.01 V
0.63 V
Current / μA
Potential / V vs. SCE

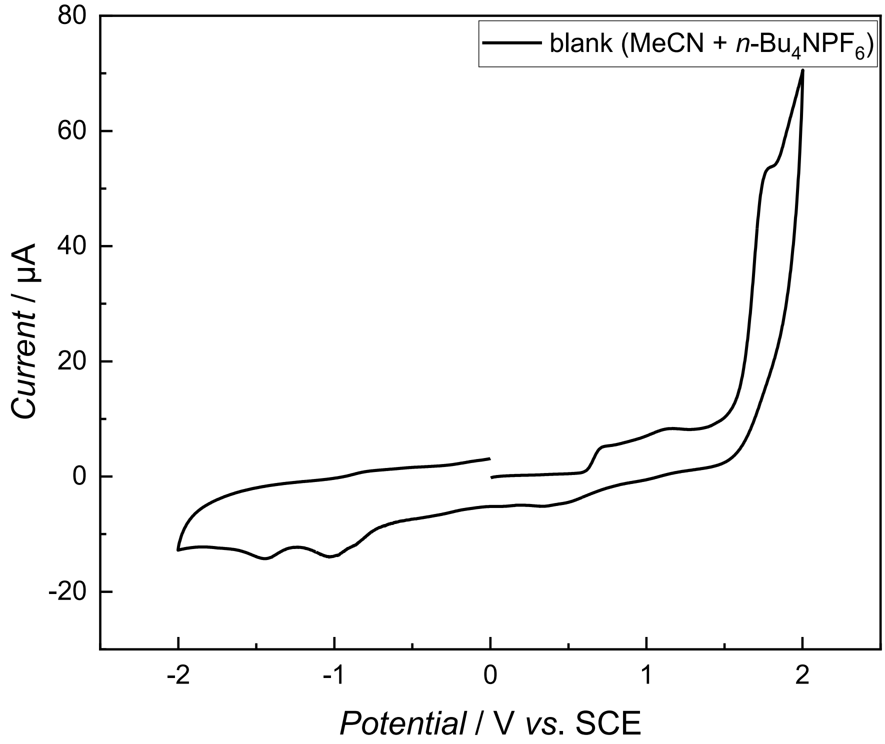
blank (MeCN + n-Bu4NPF6)
Current / μA
Potential / V vs. SCE

Excited state oxidation potential

The excited state oxidation potential E_{ox}^{*} was approximated by applying the equation (1) derived from the REHM-WELLER equation:

$$E_{ox}^{*} = E^{ox} - E_{0,0} \qquad (1)$$

E^{ox} was determined as the half-peak potential.

The 0–0 transition energy $E_{0,0}$ was estimated from the intercept of the UV-vis and fluorescence spectra.

Exemplary calculation of $E_{0,0}$ and E_{ox}^{*} for **1a**.

$$e \cdot E_{0,0} = h \cdot \nu = h \cdot \frac{c}{\lambda}$$

$$E_{0,0} = \frac{h \cdot c}{e \cdot \lambda} = \frac{6.626 \cdot 10^{-34}\ \mathrm{CVs} \cdot 3 \cdot 10^{8}\,\frac{\mathrm{m}}{\mathrm{s}}}{1.602 \cdot 10^{-19}\ \mathrm{C} \cdot 366 \cdot 10^{-9}\ \mathrm{m}} = 3.39\ \mathrm{V}$$

$$E_{ox}^{*} = 0.68\ \mathrm{V} - 3.39\ \mathrm{V} = -2.71\ \mathrm{V}$$

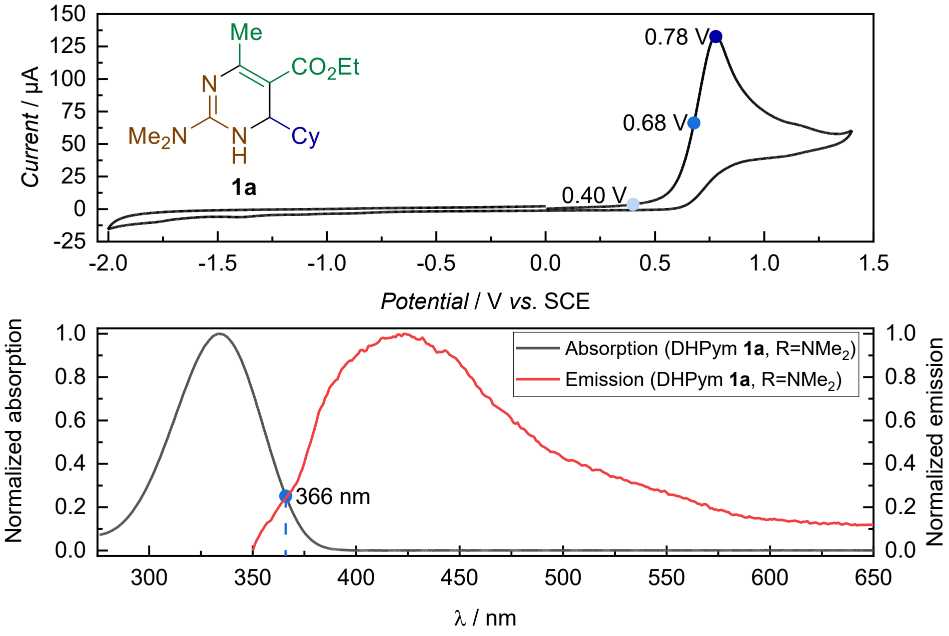
Me
CO_2Et
N
Me_2N
N
H
Cy
1a
0.78 V
0.68 V
0.40 V
Current / μA
Potential / V vs. SCE
Normalized absorption
Normalized emission
Absorption (DHPym 1a, R=NMe$_2$)
Emission (DHPym 1a, R=NMe$_2$)
366 nm
λ / nm

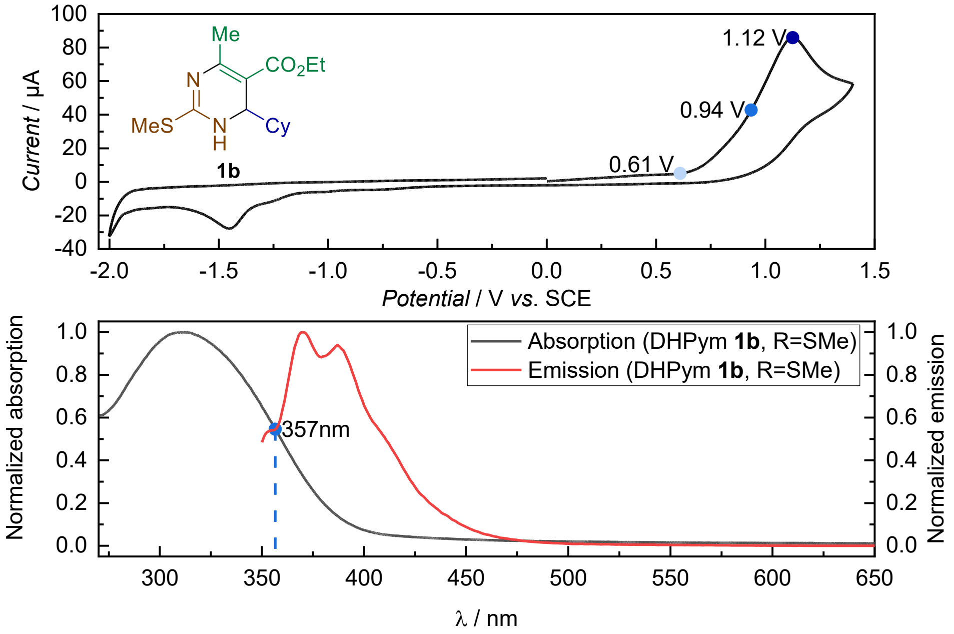
Me
CO_2Et
N
MeS
N
H
Cy
1b
1.12 V
0.94 V
0.61 V
Current / μA
Potential / V vs. SCE
Normalized absorption
Normalized emission
Absorption (DHPym 1b, R=SMe)
Emission (DHPym 1b, R=SMe)
357nm
λ / nm

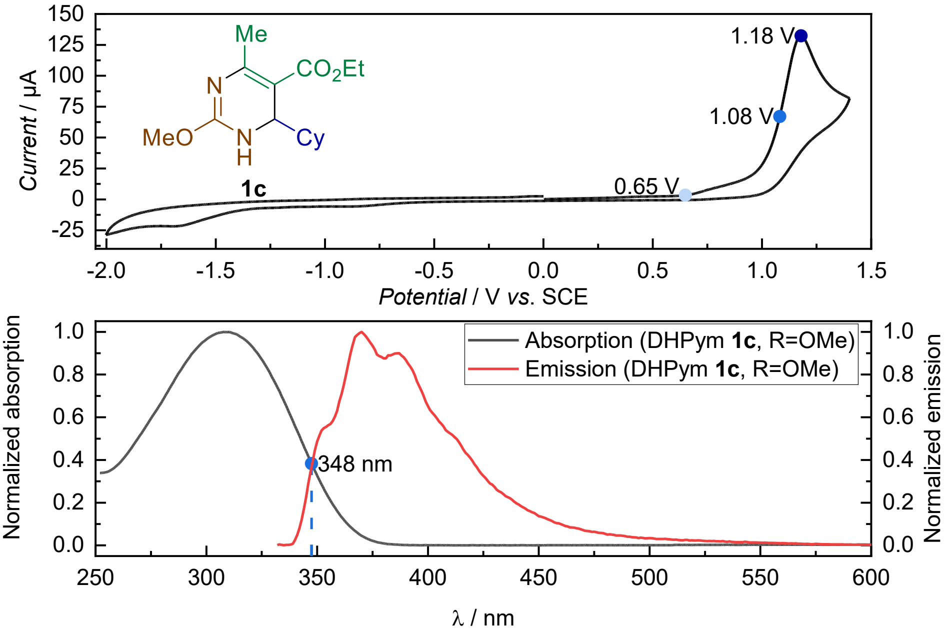
Me
CO_2Et
N
MeO
N
H
Cy
1c
1.18 V
1.08 V
0.65 V
Current / μA
Potential / V vs. SCE
Absorption (DHPym 1c, R=OMe)
Emission (DHPym 1c, R=OMe)
348 nm
Normalized absorption
Normalized emission
λ / nm

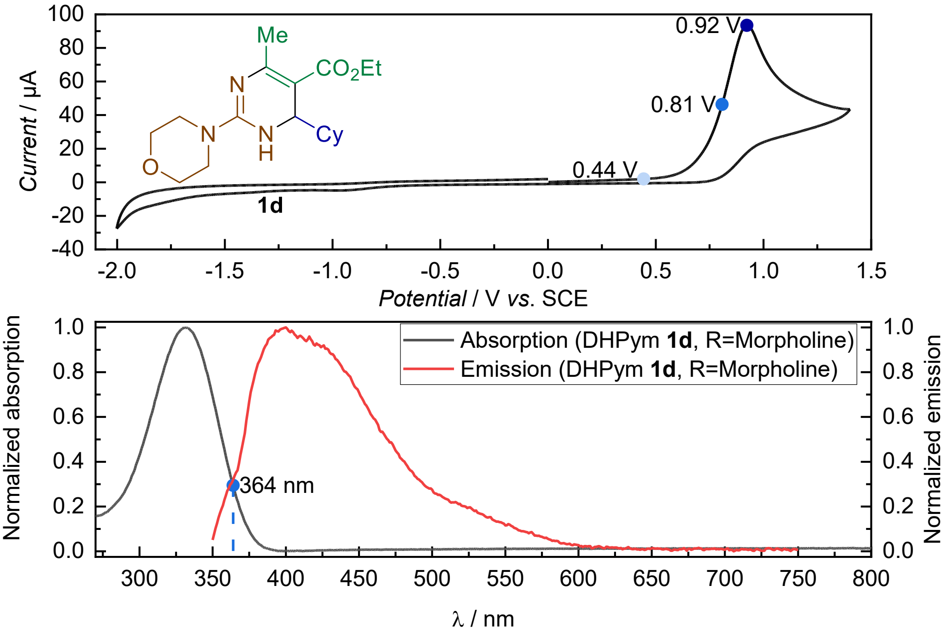
Me
CO_2Et
N
N
O
N
H
Cy
1d
0.92 V
0.81 V
0.44 V
Current / μA
Potential / V vs. SCE
Absorption (DHPym 1d, R=Morpholine)
Emission (DHPym 1d, R=Morpholine)
364 nm
Normalized absorption
Normalized emission
λ / nm

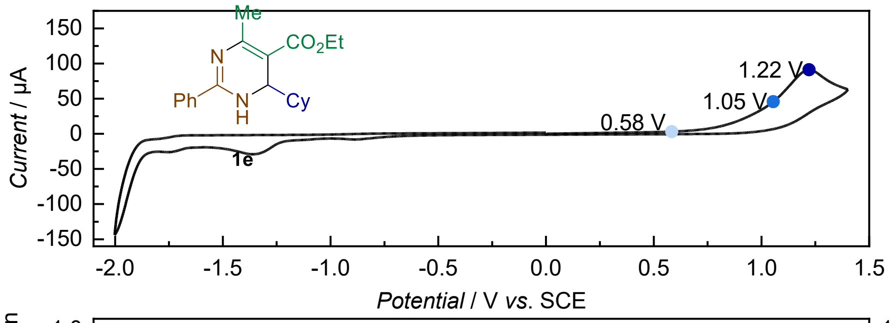

Current / µA
Potential / V vs. SCE
1e
Me
CO2Et
N
Ph
N
H
Cy
1.22 V
1.05 V
0.58 V

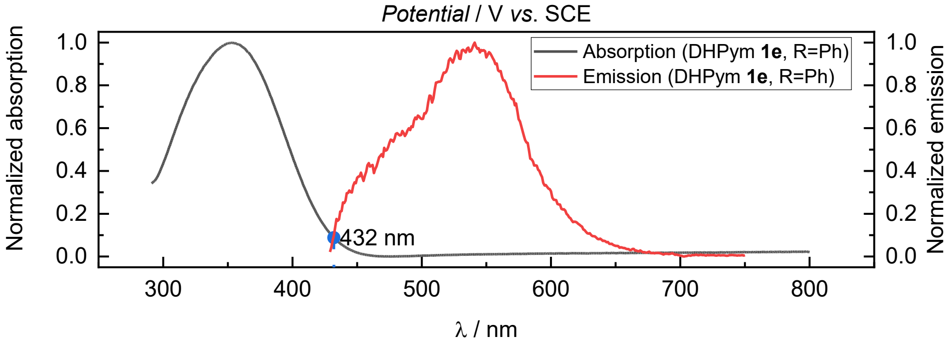

Normalized absorption
Normalized emission
λ / nm
Absorption (DHPym 1e, R=Ph)
Emission (DHPym 1e, R=Ph)
432 nm

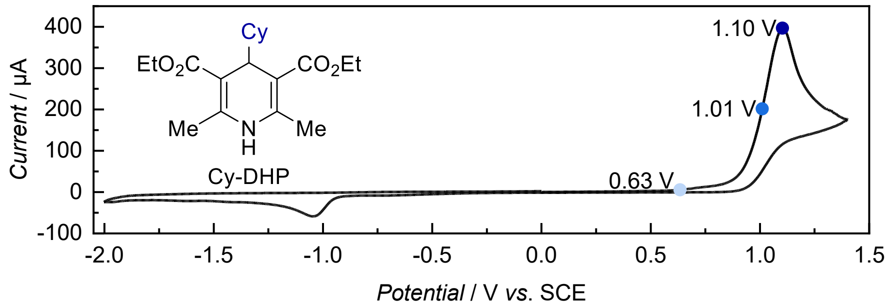

Current / µA
Potential / V vs. SCE
Cy
EtO2C
CO2Et
Me
N
H
Me
Cy-DHP
1.10 V
1.01 V
0.63 V

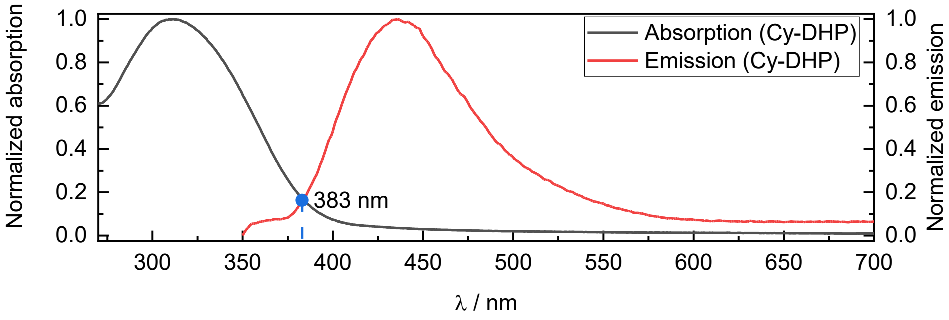

Normalized absorption
Normalized emission
λ / nm
Absorption (Cy-DHP)
Emission (Cy-DHP)
383 nm

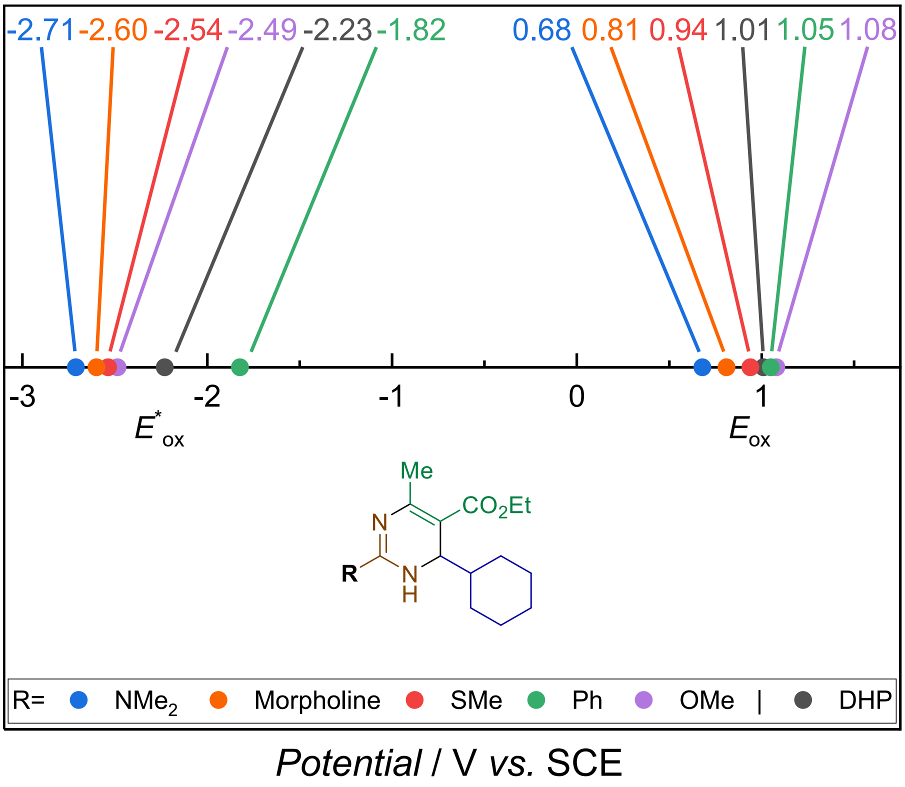

Experimentally determined ground-state and excited-state oxidation potentials of DHPyms and DHP.

Luminescence quenching

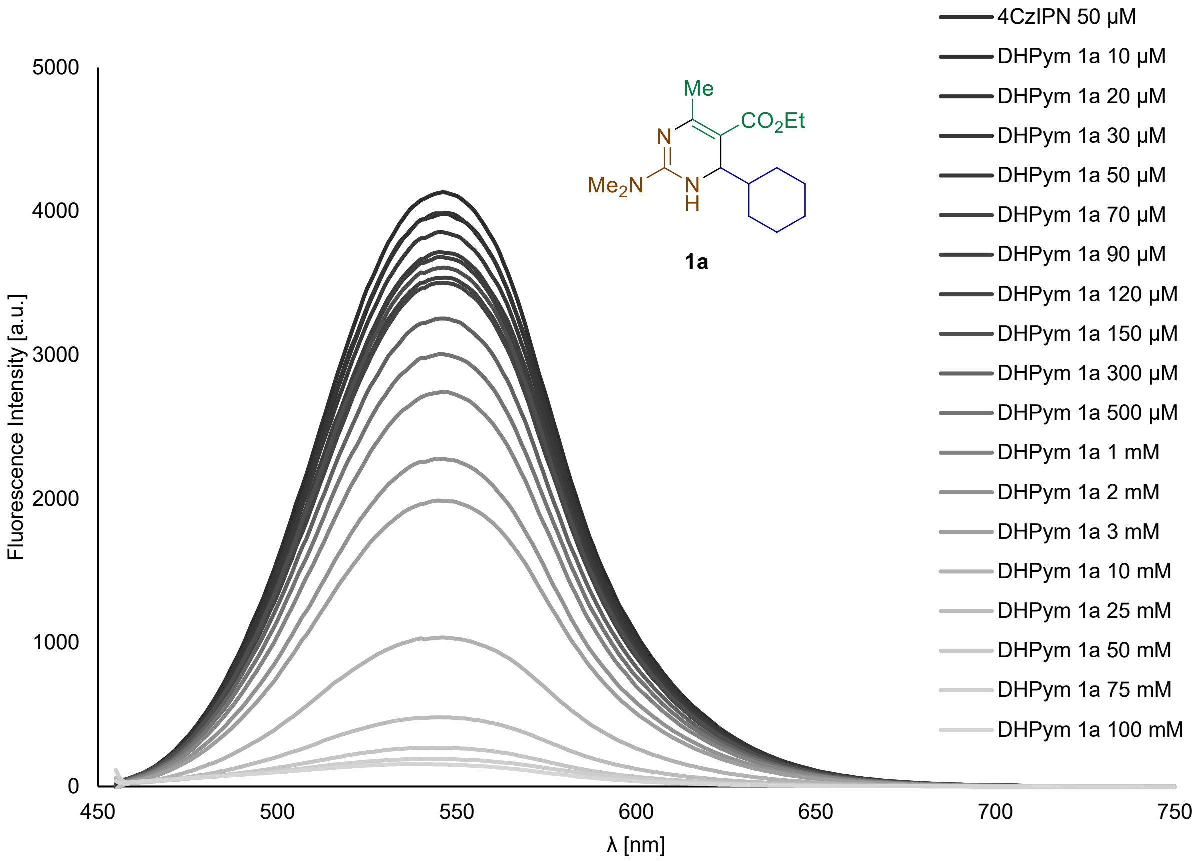

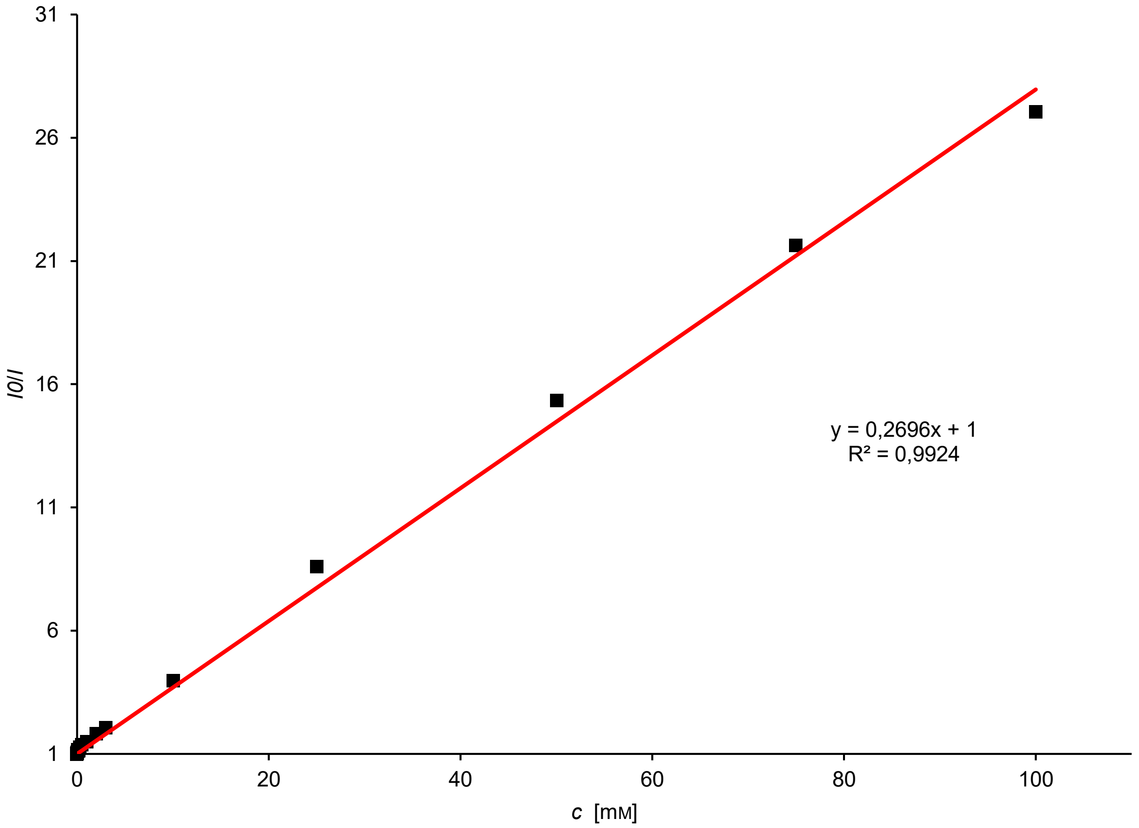

Stern-Volmer quenching experiment was conducted: A solution of the photocatalyst 4CzIPN (50 µM) in degassed MeCN was measured with increasing concentrations of DHPym **1a** as the fluorescence quencher. The samples were prepared under argon and degassed prior to measurement. Equation (2) was derived by linear regression.

$$\frac{I_0}{I} = 0.2696 \cdot c + 1 \qquad (2)$$

To investigate if the pyrimidine byproduct influences the Stern-Volmer quenching experiment, a fluorescence spectrum was recorded under identical conditions as the Stern-Volmer quenching experiment with a 10 mM concentration of the pyrimidine derivative in degassed MeCN without photocatalyst. The observed fluorescence was weak compared to 4CzIPN in 50µM concentration so it is unlikely to influence the Stern-Volmer quenching study

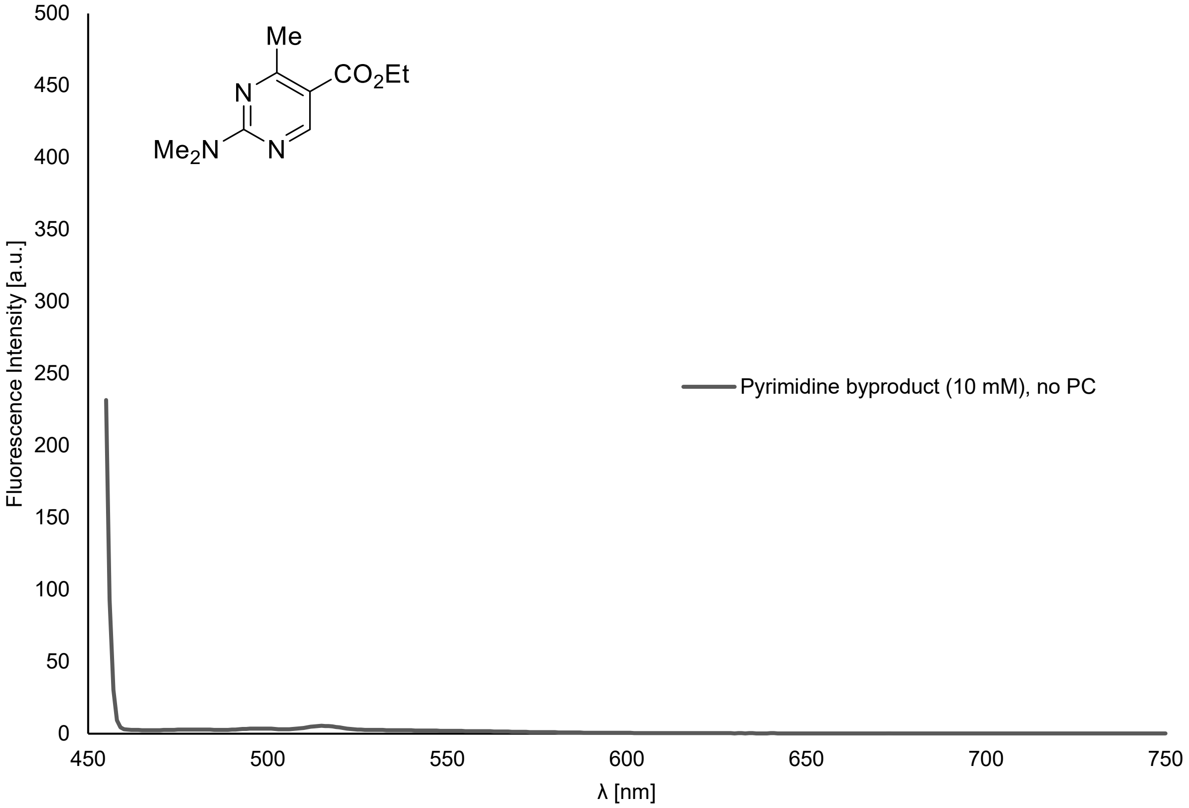

Proposed mechanisms

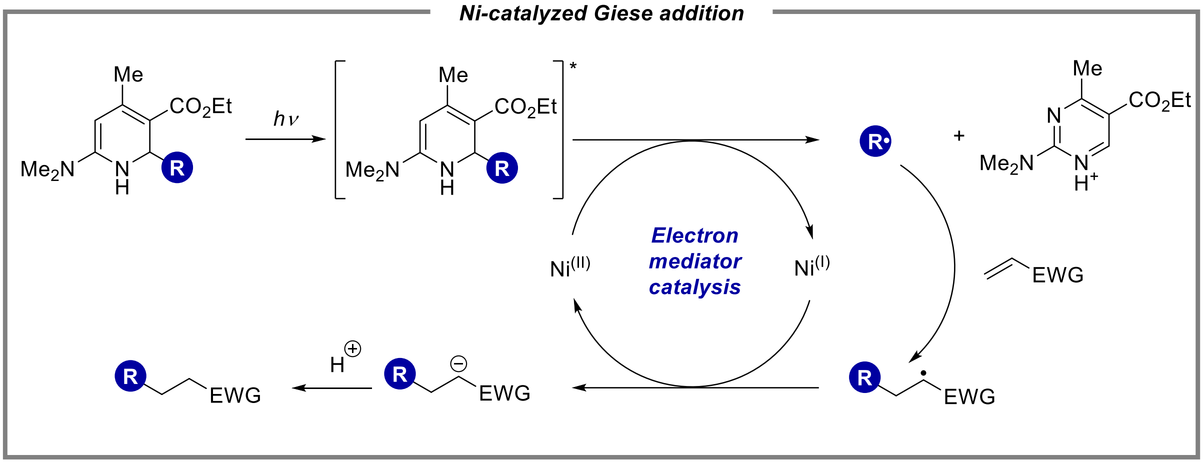

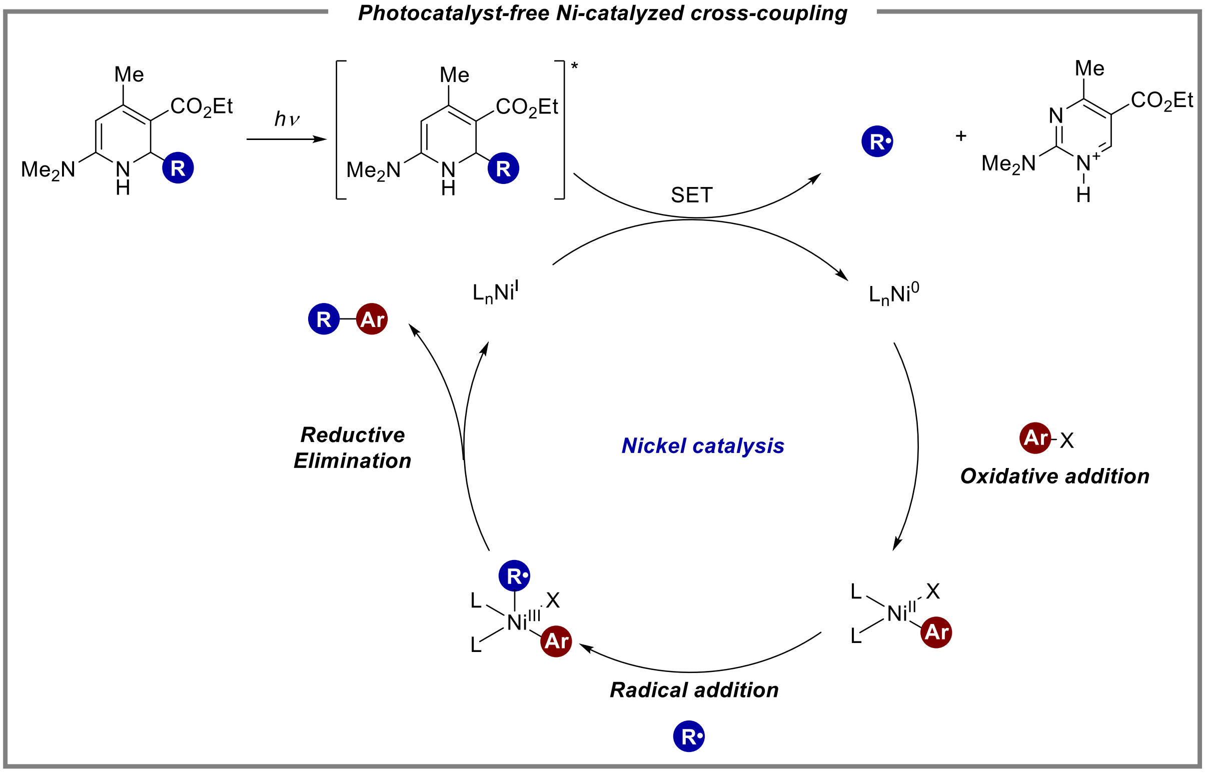

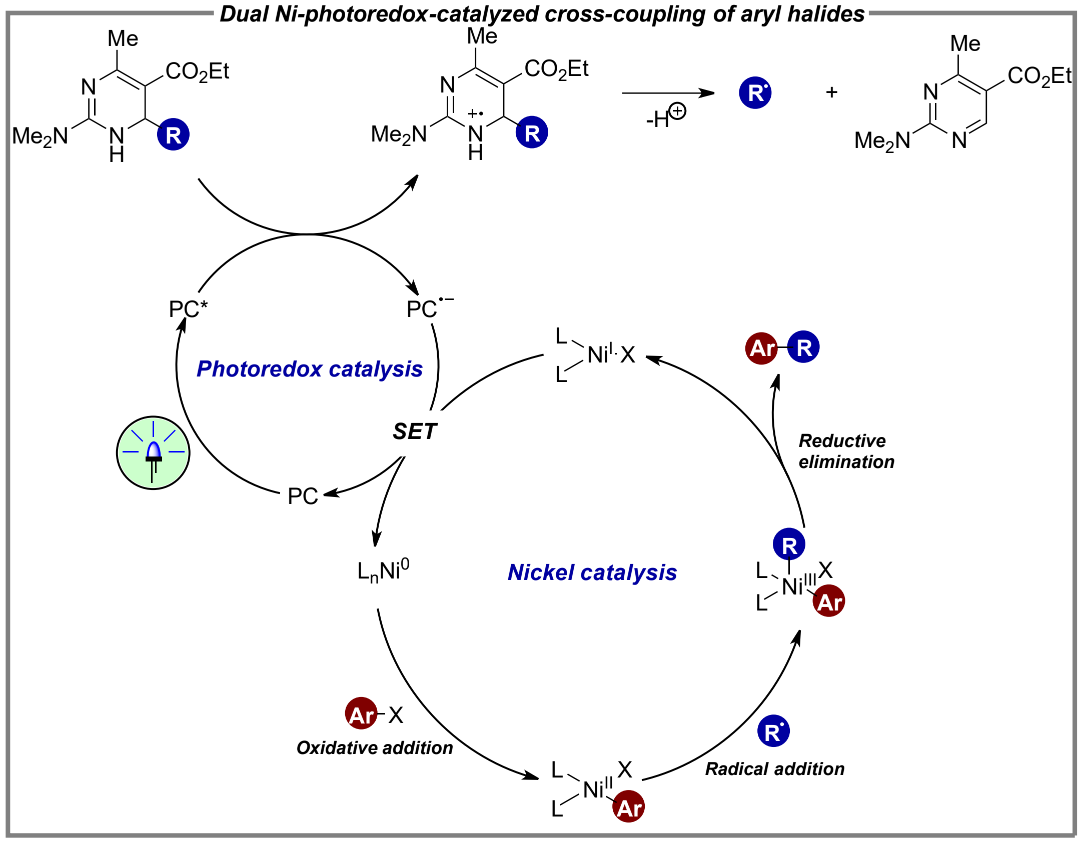
Dual Ni-photoredox-catalyzed cross-coupling of aryl halides
Photoredox catalysis
SET
Nickel catalysis
Reductive elimination
Oxidative addition
Radical addition

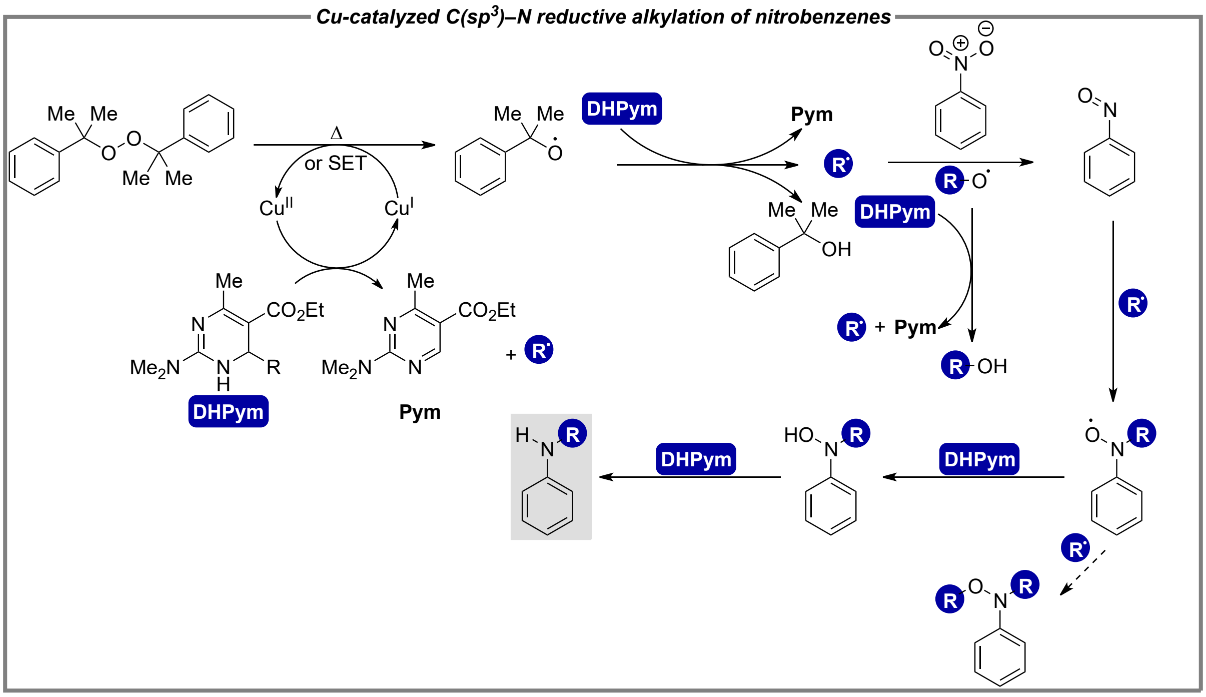
Cu-catalyzed C(sp3)–N reductive alkylation of nitrobenzenes
DHPym
Pym

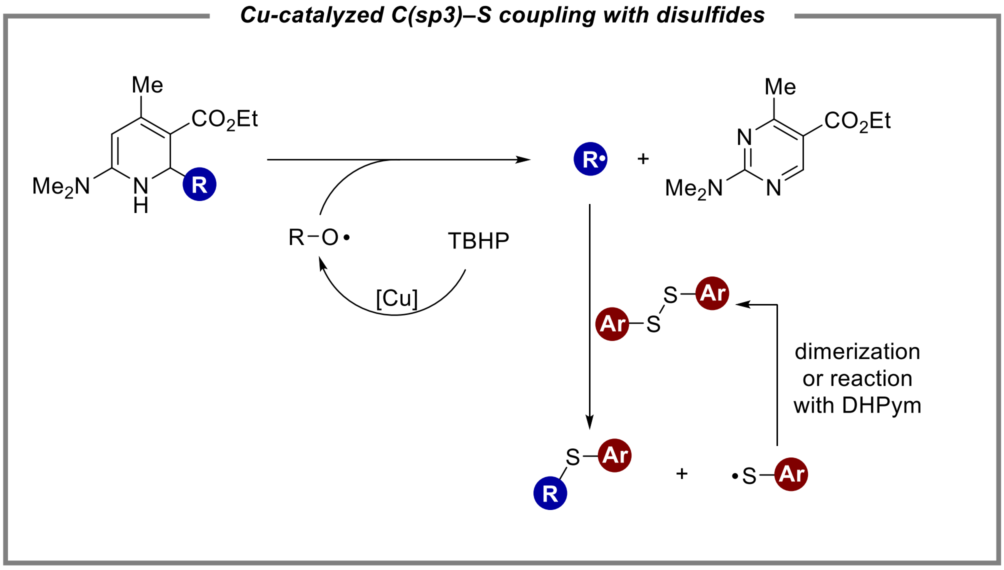
Cu-catalyzed C(sp3)–S coupling with disulfides
Me
CO2Et
Me2N
N
H
R
R•
+
Me
N
CO2Et
Me2N
N
R–O•
TBHP
[Cu]
S
Ar
Ar
S
dimerization
or reaction
with DHPym
S
Ar
R
+
•S
Ar

Crystallographic Data

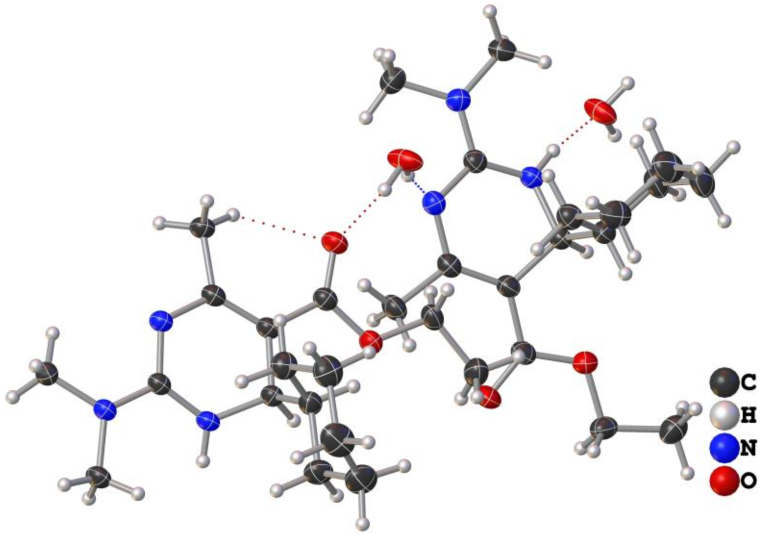

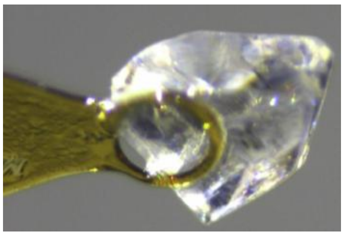

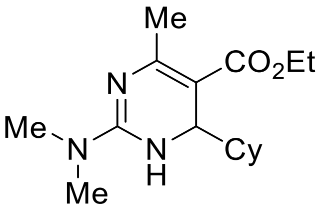

1a

Table S28. Crystal data and structure refinement for cu_2067_cg_0m_4

Identification code	cu_2067_cg_0m_4
CCDC number	2338773
Empirical formula	$C_{16}H_{29}N_3O_3$
Formula weight	311.42
Temperature [K]	100.00
Crystal system	monoclinic
Space group (number)	$P2_1/c$ (14)
a [Å]	11.7086(15)
b [Å]	9.5131(12)
c [Å]	31.662(4)
α [°]	90
β [°]	94.913(3)
γ [°]	90
Volume [Å^3]	3513.7(8)
Z	8
ρ_{calc} [gcm^{-3}]	1.177
μ [mm^{-1}]	0.658
F(000)	1360
Crystal size [mm^3]	1.045×0.604×0.372
Crystal colour	colourless
Crystal shape	block
Radiation	CuK_α (λ=1.54178 Å)
2θ range [°]	5.60 to 157.20 (0.79 Å)
Index ranges	$-14 \le h \le 14$<br>$0 \le k \le 12$<br>$0 \le l \le 39$
Reflections collected	8927
Independent reflections	8927<br>R_{int} = 0.1048<br>R_{sigma} = 0.0462

Completeness to<br>θ = 67.679°	99.5 %
Data / Restraints / Parameters	8927/0/431
Goodness-of-fit on F^2	1.091
Final *R* indexes<br>[I≥2σ(I)]	R_1 = 0.0693<br>wR_2 = 0.2155
Final *R* indexes<br>[all data]	R_1 = 0.0744<br>wR_2 = 0.2248
Largest peak/hole [eÅ^{-3}]	0.37/-0.31

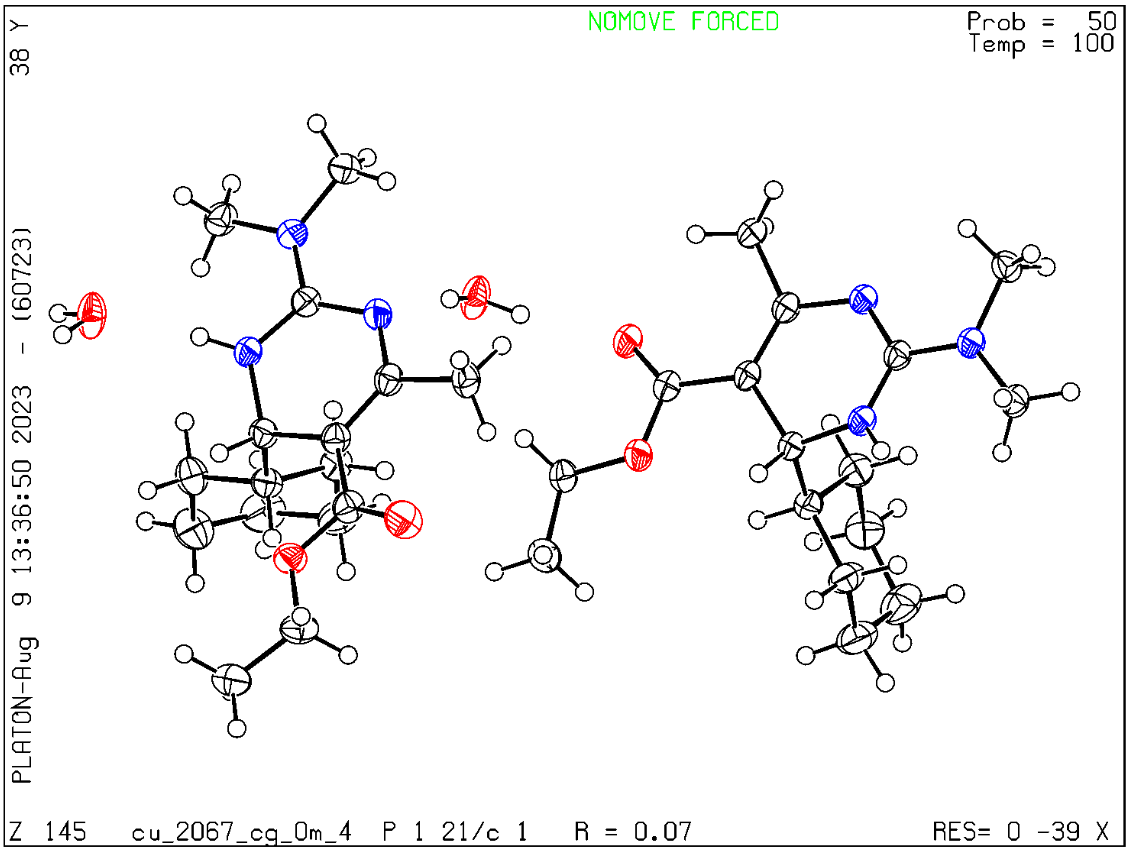

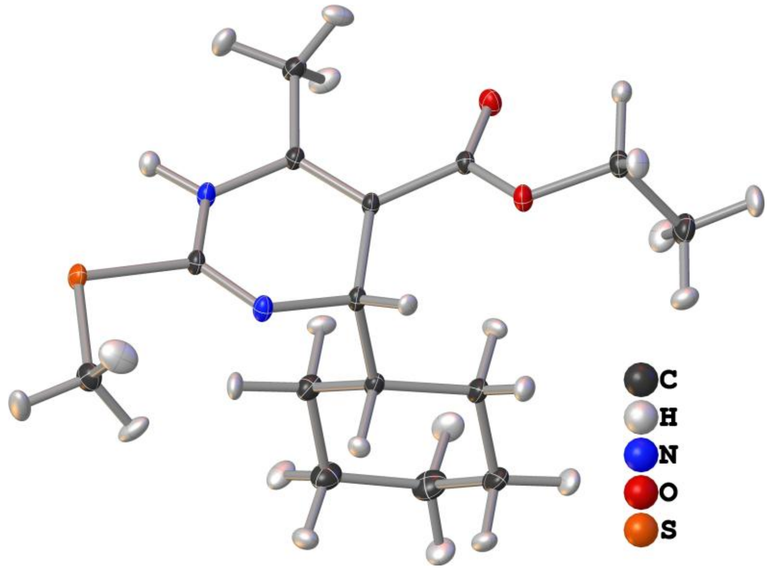

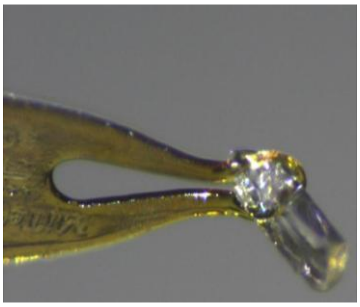

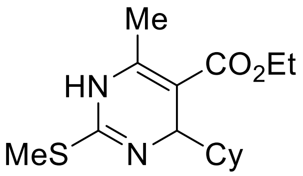

1b

Table S29. Crystal data and structure refinement for 2076_P21c

Identification code	2076_P21c
CCDC number	2338774
Empirical formula	$C_{15}H_{24}N_2O_2S$
Formula weight	296.436
Temperature [K]	100.00
Crystal system	monoclinic
Space group (number)	$P2_1/c$ (14)
a [Å]	13.3817(7)
b [Å]	9.0674(5)
c [Å]	12.4298(8)
α [°]	90
β [°]	92.322(2)
γ [°]	90
Volume [Å^3]	1506.96(15)
Z	4
ρ_{calc} [gcm^{-3}]	1.307
μ [mm^{-1}]	0.219
F(000)	640.849
Crystal size [mm^3]	0.594×0.226×0.102
Crystal colour	colourless
Crystal shape	block
Radiation	Mo K_α (λ=0.71073 Å)
2θ range [°]	5.42 to 66.24 (0.65 Å)
Index ranges	−17 ≤ h ≤ 20<br>−13 ≤ k ≤ 13<br>−18 ≤ l ≤ 18
Reflections collected	53044
Independent reflections	5461<br>R_{int} = 0.0358<br>R_{sigma} = 0.0169

Completeness to θ = 25.2417°	100.0 %
Data / Restraints / Parameters	5461/0/397
Goodness-of-fit on F^2	1.1160
Final R indexes [I≥2σ(I)]	R_1 = 0.0144<br>wR_2 = 0.0325
Final R indexes [all data]	R_1 = 0.0162<br>wR_2 = 0.0334
Largest peak/hole [eÅ^{-3}]	0.17/-0.14

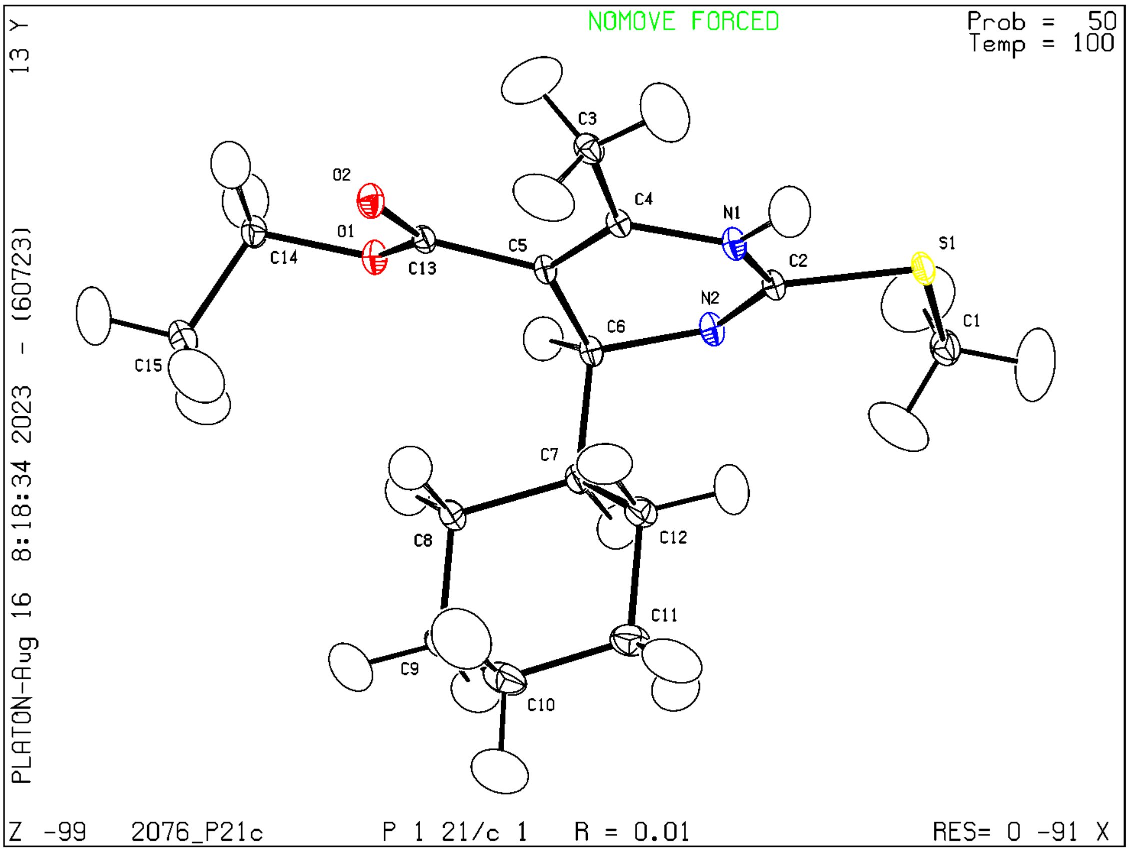

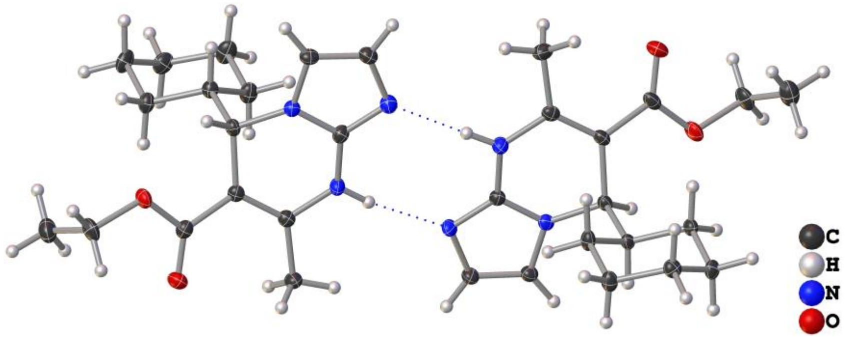

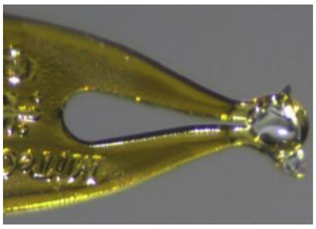

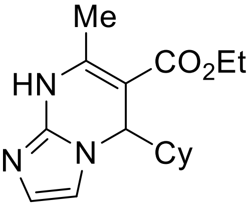

1h

Table S30. Crystal data and structure refinement for 2234_pna21

Identification code	2234_pna21
CCDC number	2338775
Empirical formula	$C_{16}H_{23}N_3O_2$
Formula weight	289.37
Temperature [K]	100.00
Crystal system	orthorhombic
Space group (number)	$Pna2_1$ (33)
a [Å]	18.2950(5)
b [Å]	10.1722(3)
c [Å]	16.2289(5)
α [°]	90
β [°]	90
γ [°]	90
Volume [Å^3]	3020.21(15)
Z	8
ρ_{calc} [gcm^{-3}]	1.273
μ [mm^{-1}]	0.085
F(000)	1248
Crystal size [mm^3]	0.299×0.158×0.102
Crystal colour	colourless
Crystal shape	block
Radiation	MoK_{α} (λ=0.71073 Å)
2θ range [°]	4.45 to 55.82 (0.76 Å)
Index ranges	−24 ≤ h ≤ 24<br>−12 ≤ k ≤ 13<br>−21 ≤ l ≤ 21
Reflections collected	55755
Independent reflections	7221<br>R_{int} = 0.0601<br>R_{sigma} = 0.0346
Completeness to<br>θ = 25.242°	100.0 %
Data / Restraints / Parameters	7221/3/389

Absorption correction T_{min}/T_{max} (method)	0.8155/1.0000 (numerical)
Goodness-of-fit on F^2	1.070
Final R indexes [I≥2σ(I)]	R_1 = 0.0515<br>wR_2 = 0.1313
Final R indexes [all data]	R_1 = 0.0586<br>wR_2 = 0.1371
Largest peak/hole [eÅ^{-3}]	0.44/−0.28
Flack X parameter	0.2(4)

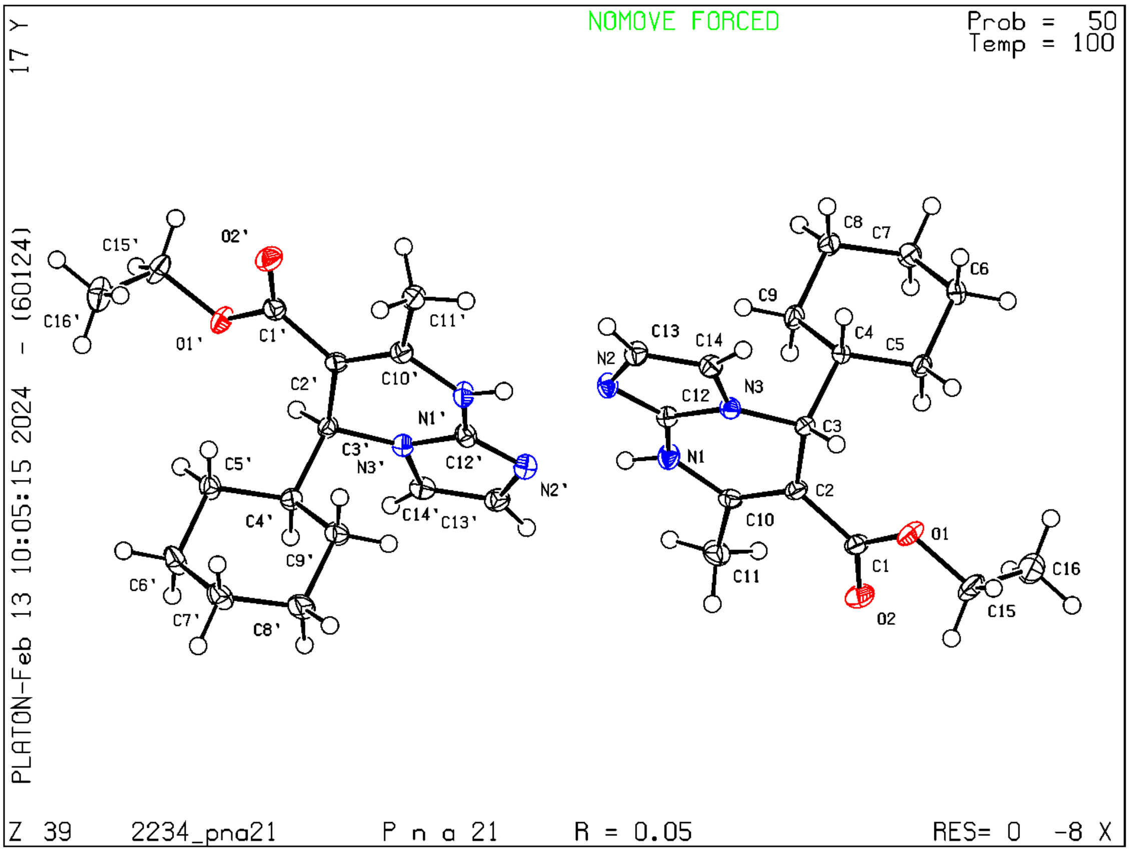

Spectral data

Ethyl 6-cyclohexyl-2-(dimethylamino)-4-methyl-1,6-dihydropyrimidine-5-carboxylate (1a)

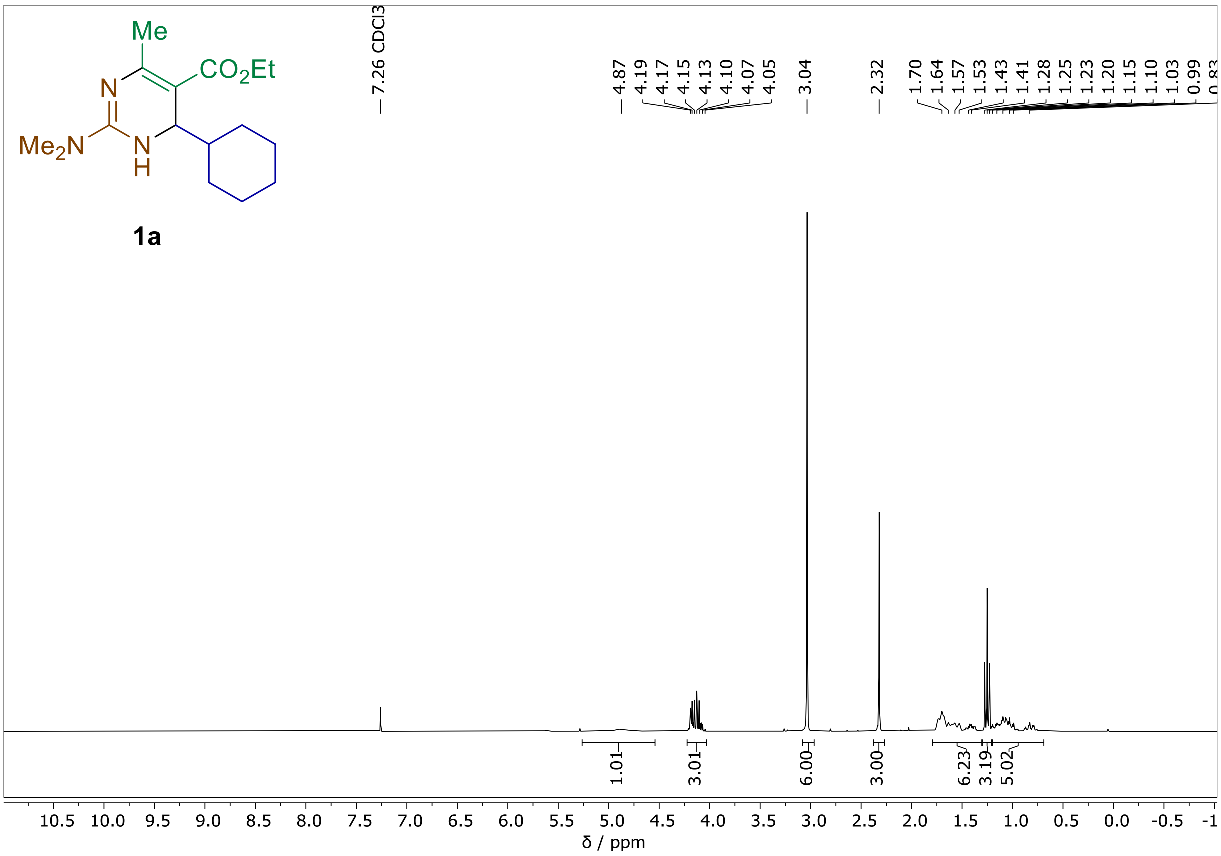

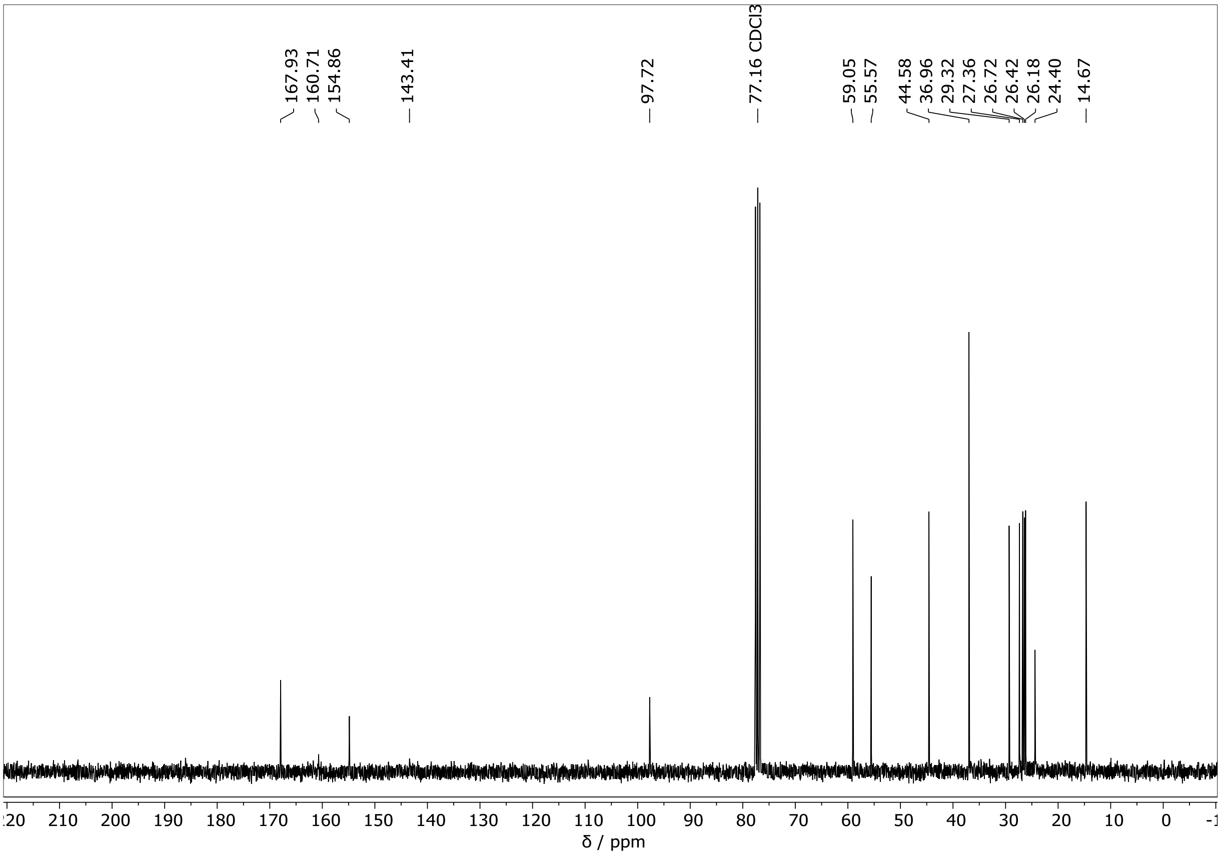

Ethyl 6-cyclohexyl-4-methyl-2-(methylthio)-1,6-dihydropyrimidine-5-carboxylate & ethyl 4-cyclohexyl-6-methyl-2-(methylthio)-1,4-dihydropyrimidine-5-carboxylate (1b)

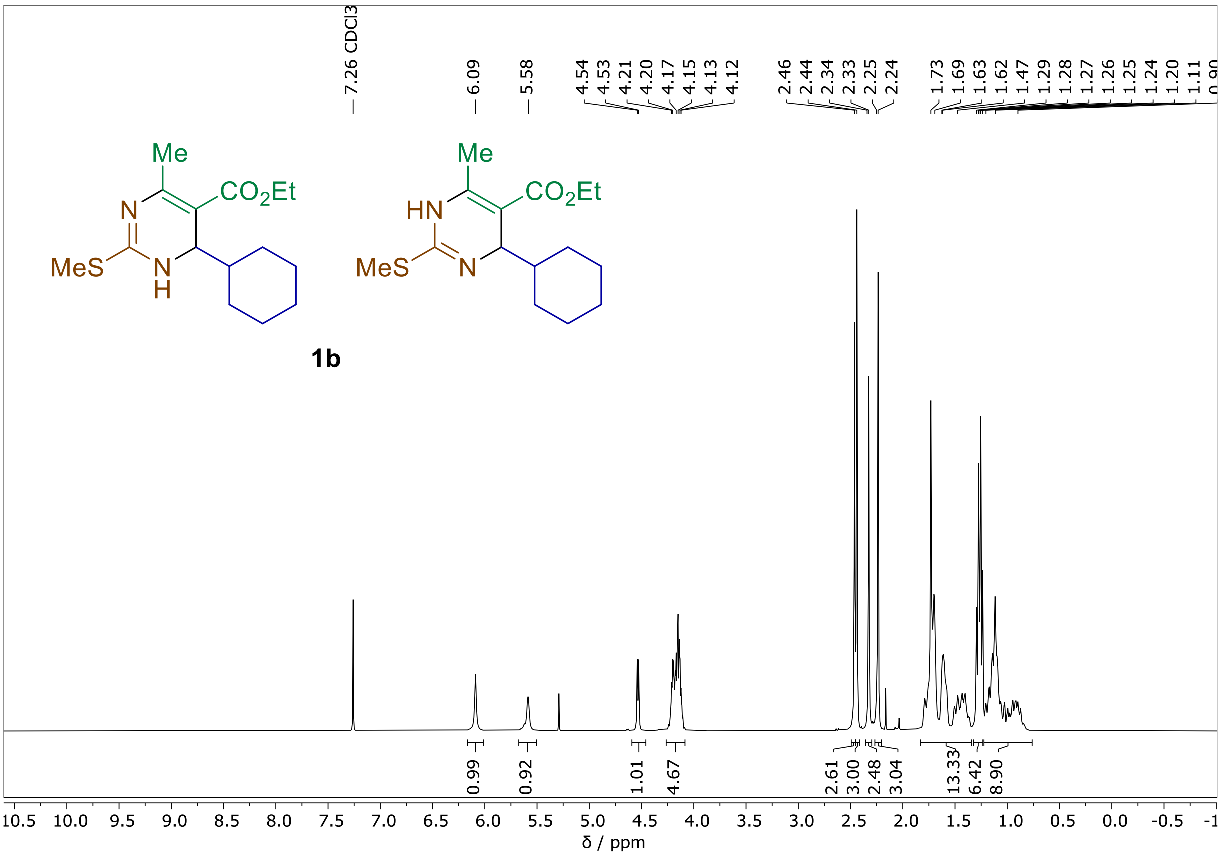

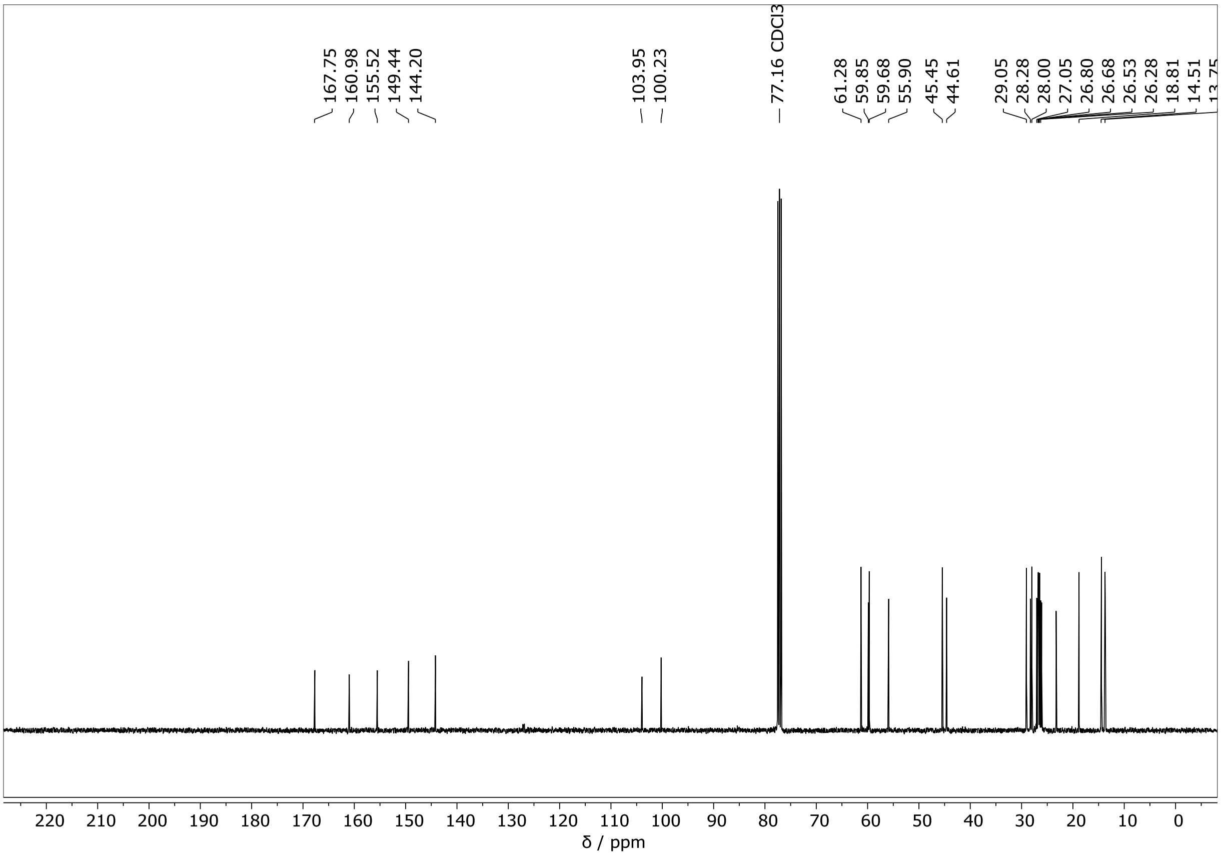

Ethyl 6-cyclohexyl-2-methoxy-4-methyl-1,6-dihydropyrimidine-5-carboxylate & ethyl 4-cyclohexyl-2-methoxy-6-methyl-1,4-dihydropyrimidine-5-carboxylate (1c)

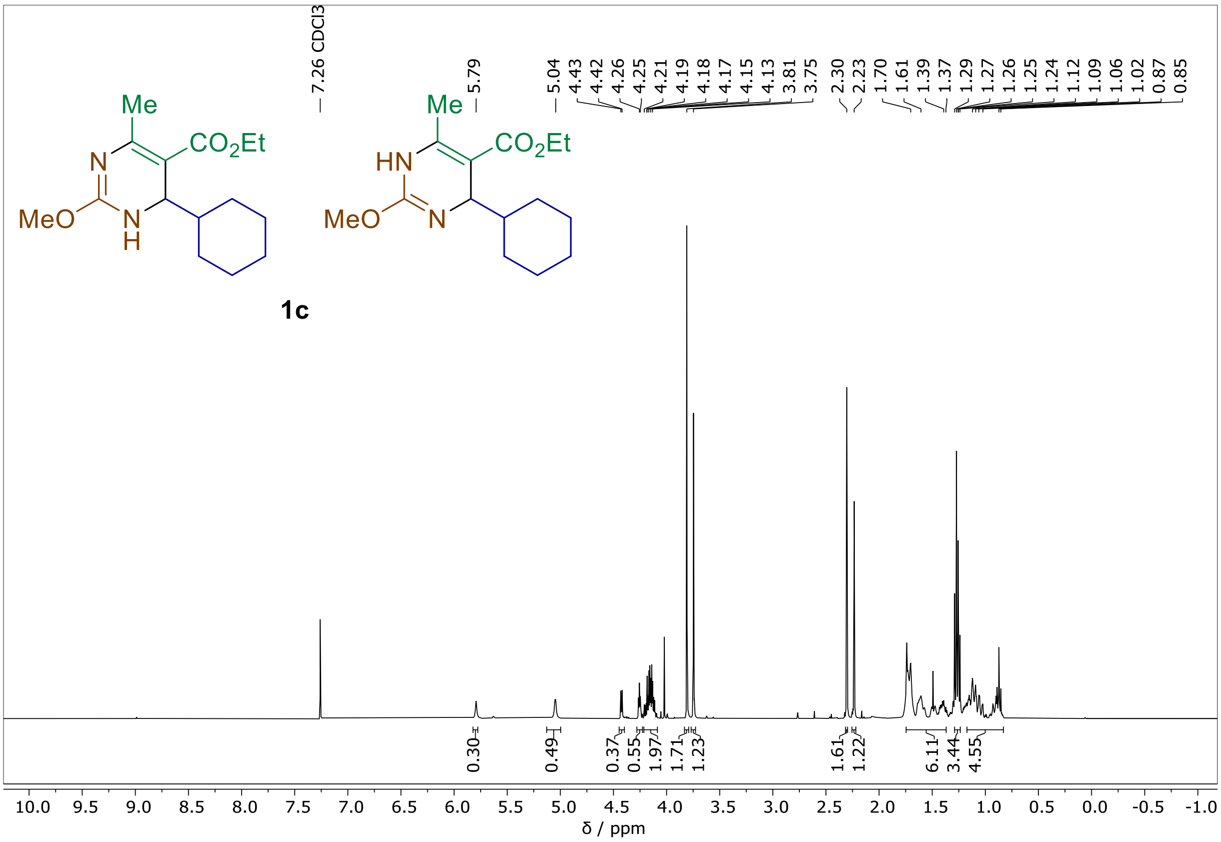

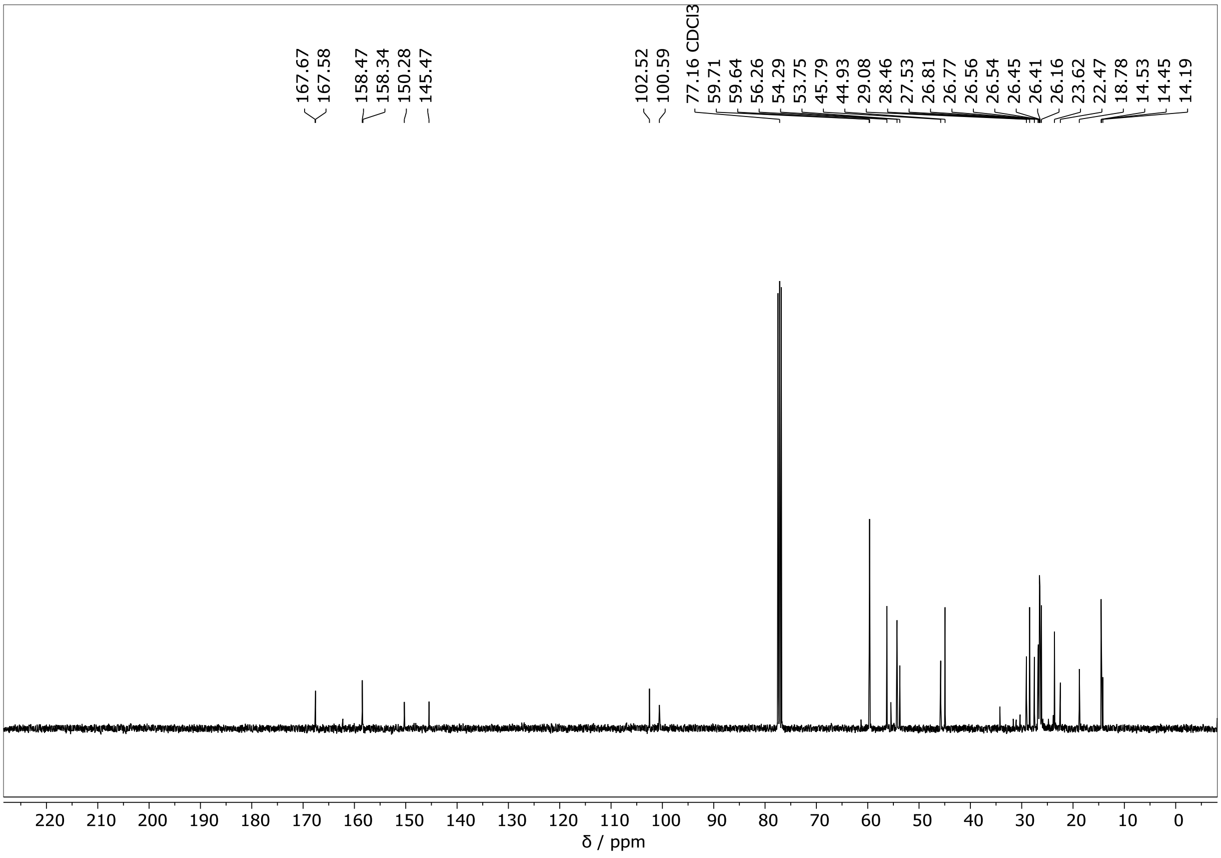

Ethyl 6-cyclohexyl-4-methyl-2-morpholino-1,6-dihydropyrimidine-5-carboxylate (1d)

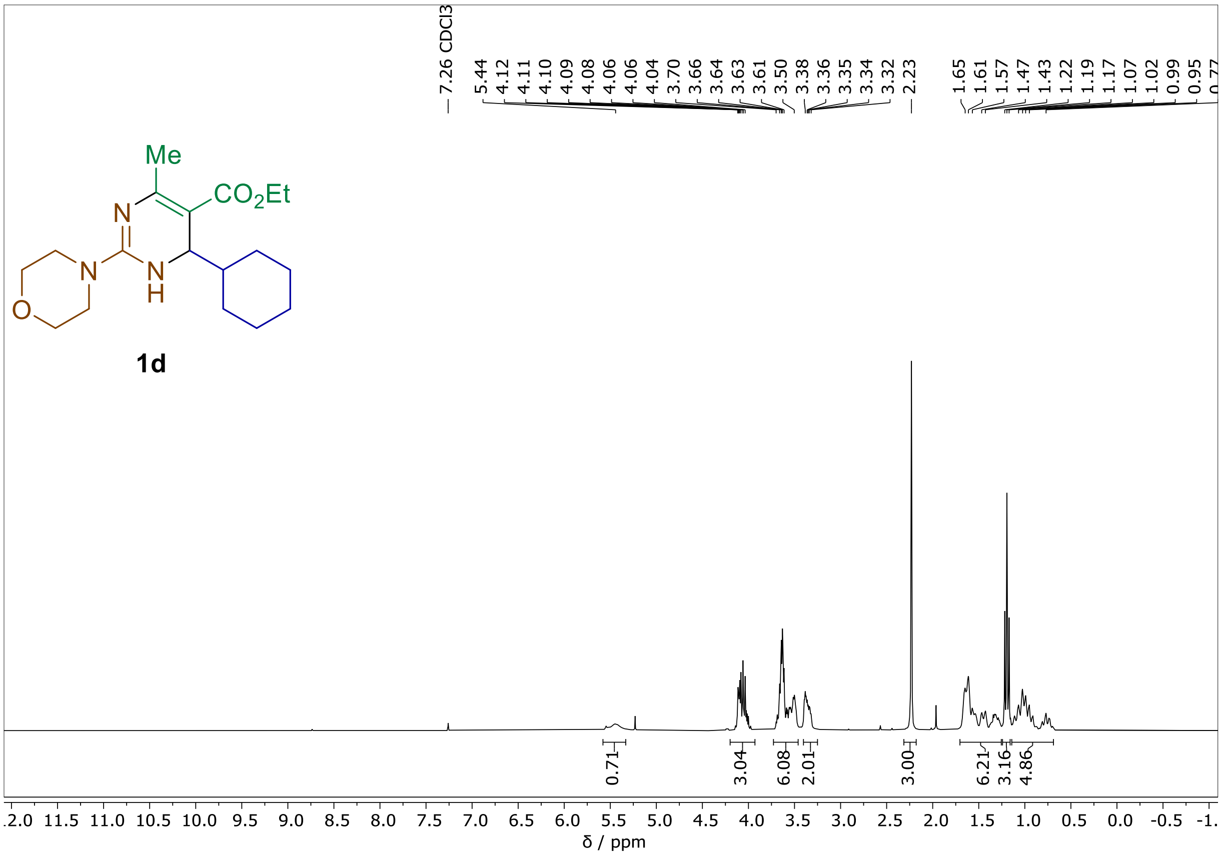

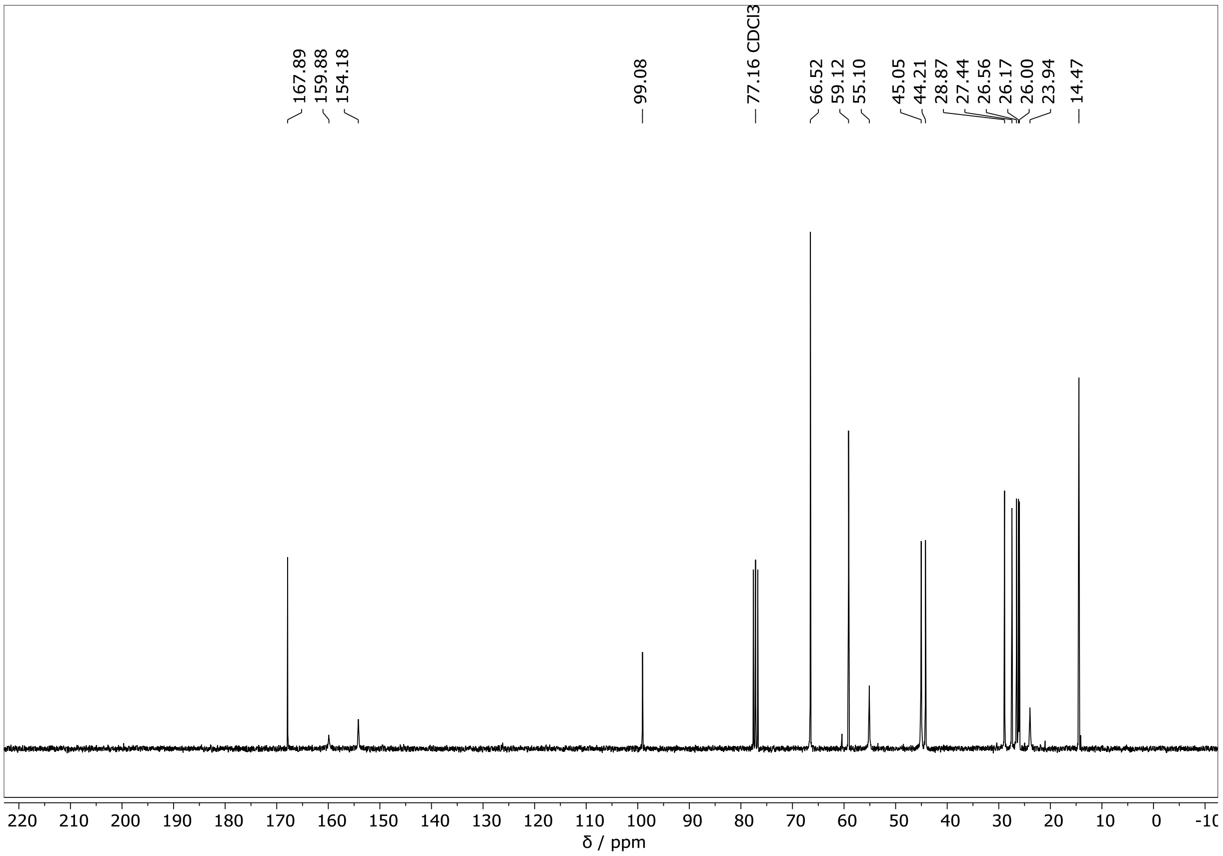

Ethyl 6-cyclohexyl-4-methyl-2-phenyl-1,6-dihydropyrimidine-5-carboxylate (1e)

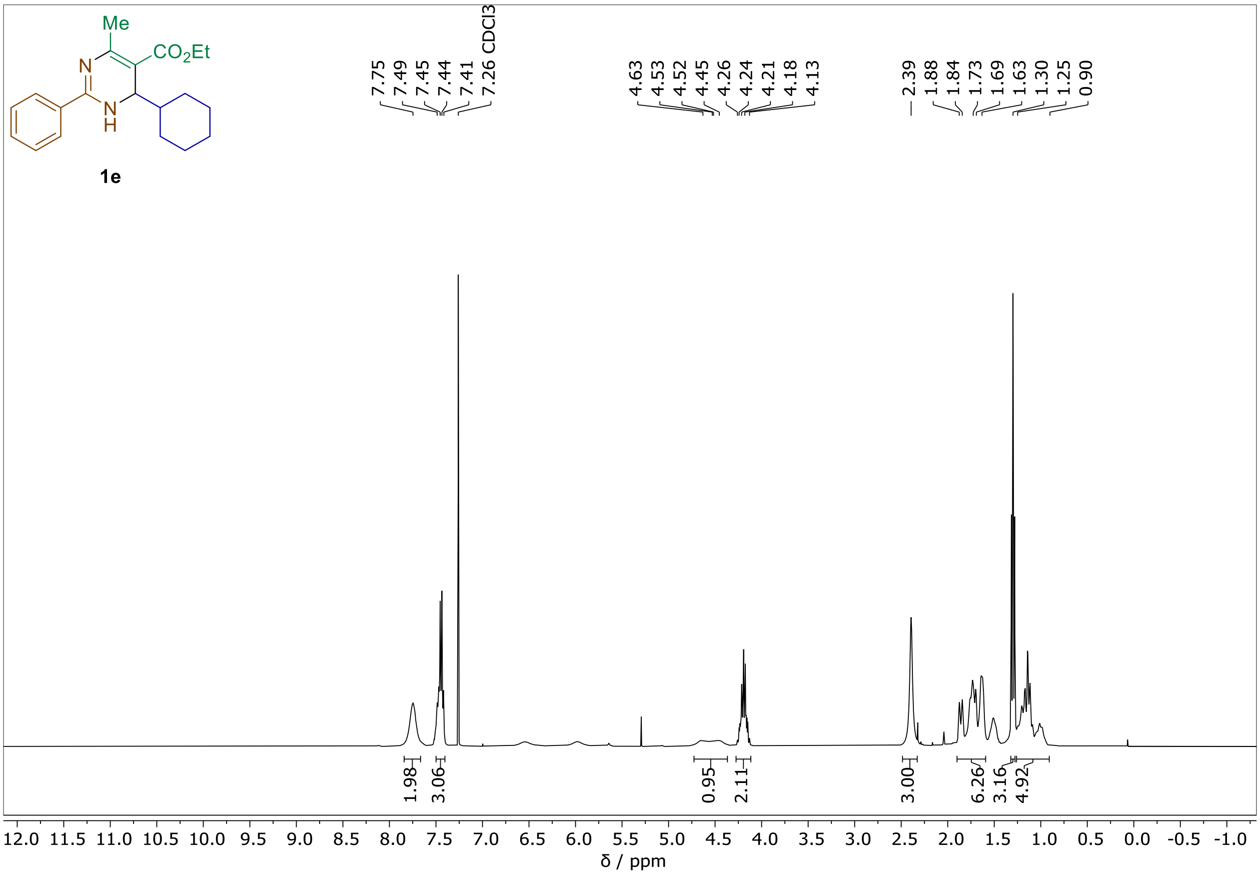

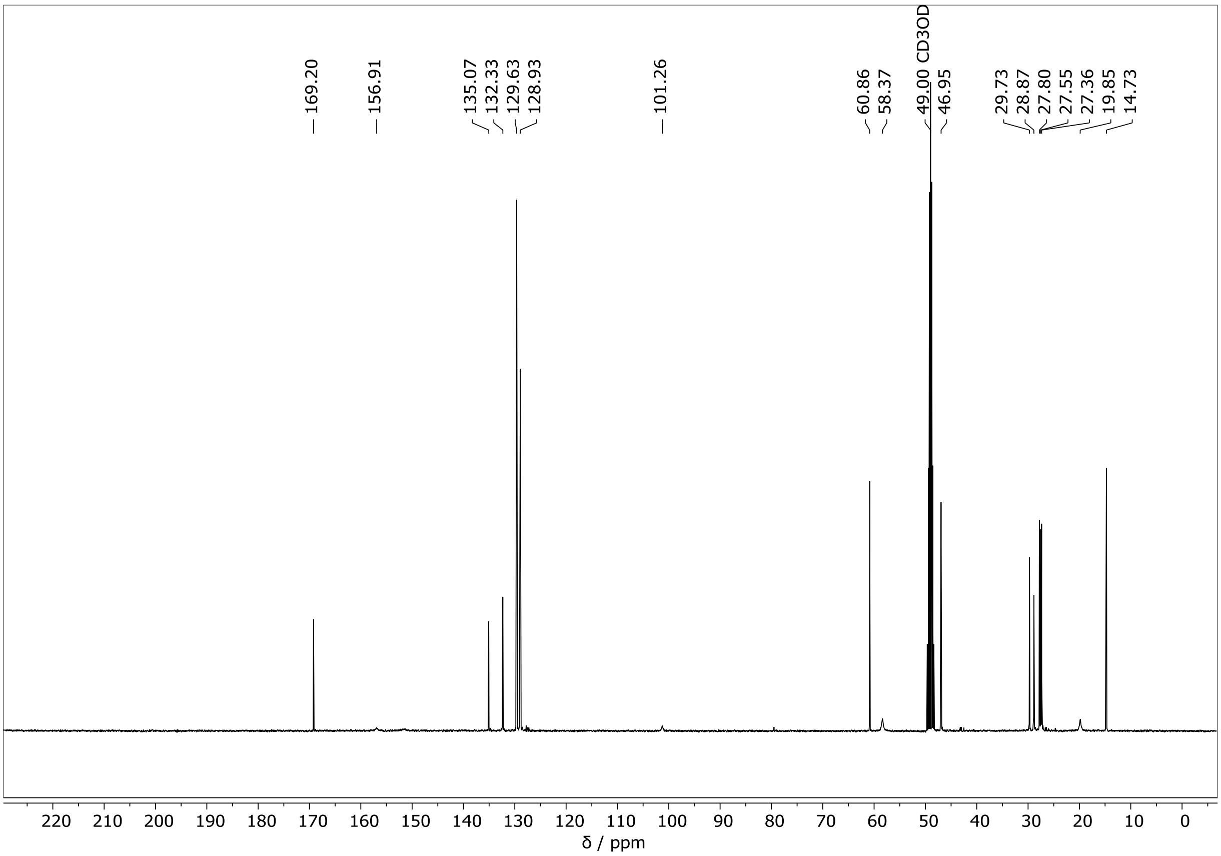

***Tert*-butyl 6-cyclohexyl-4-methyl-2-(methylthio)-1,6-dihydropyrimidine-5-carboxylate & *tert*-butyl 4-cyclohexyl-6-methyl-2-(methylthio)-1,4-dihydropyrimidine-5-carboxylate (1f)**

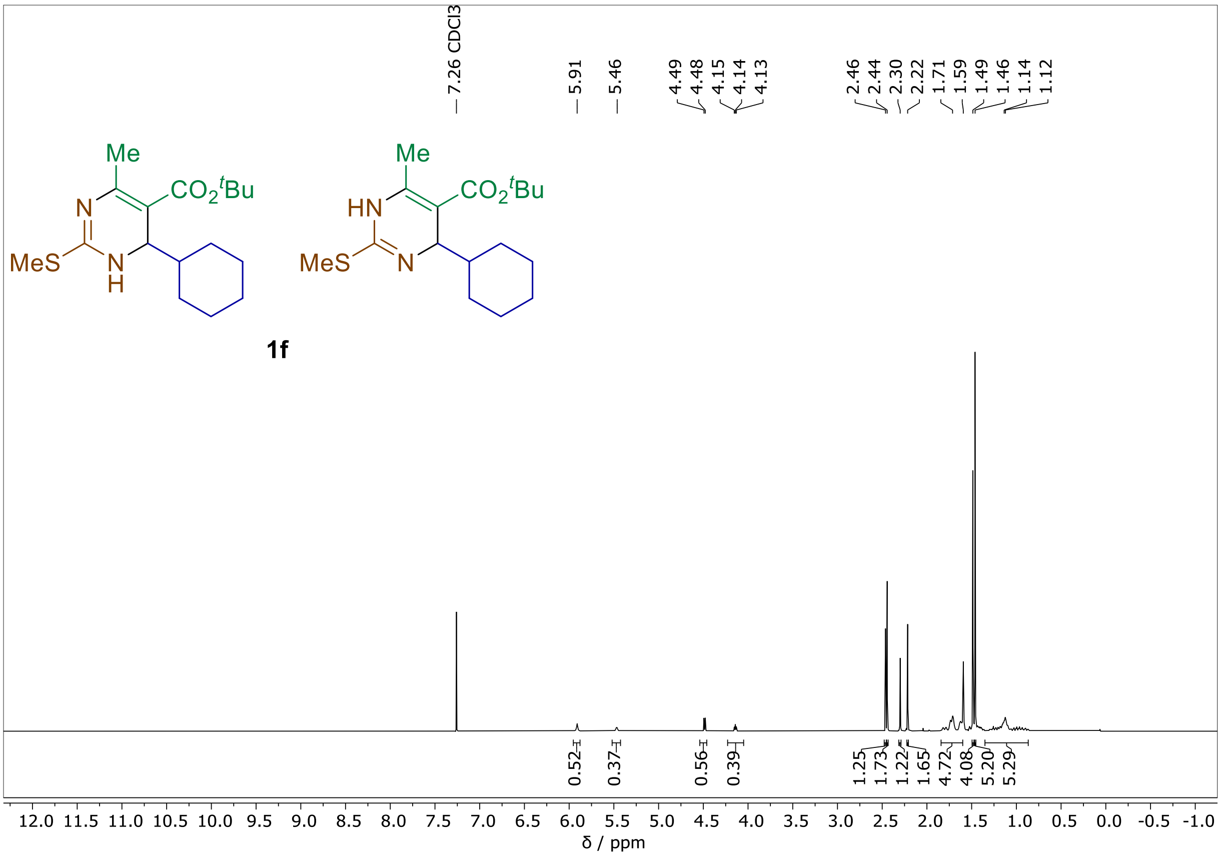

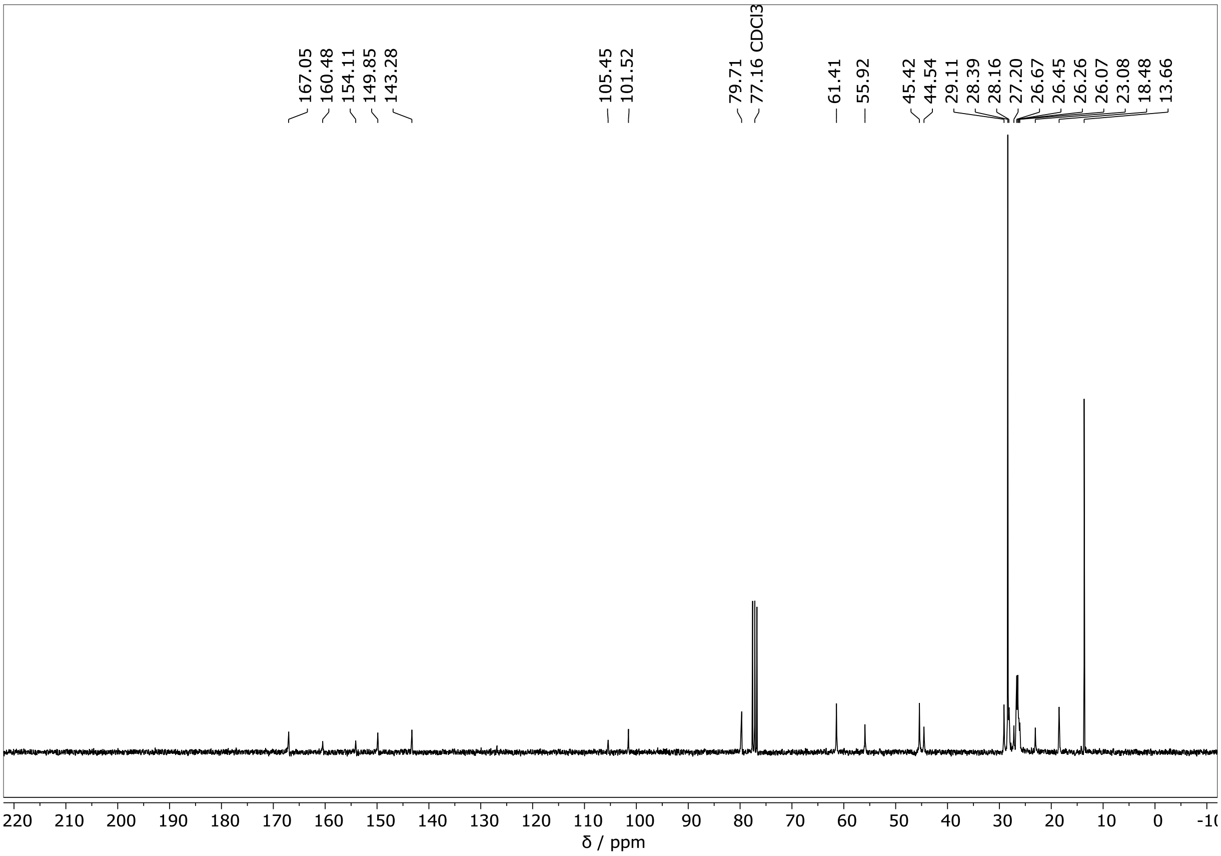

Ethyl 2-(4-chlorophenyl)-6-cyclohexyl-4-methyl-1,6-dihydropyrimidine-5-carboxylate (1g)

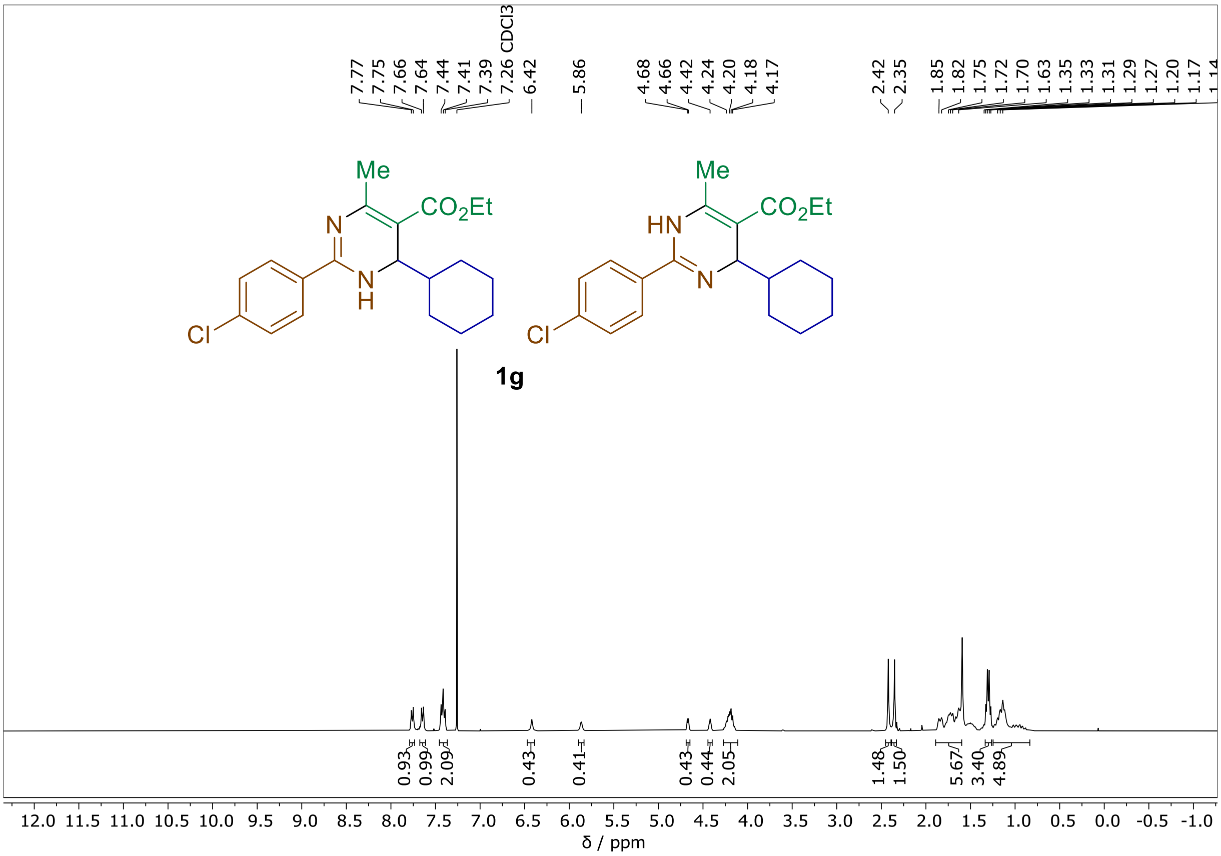

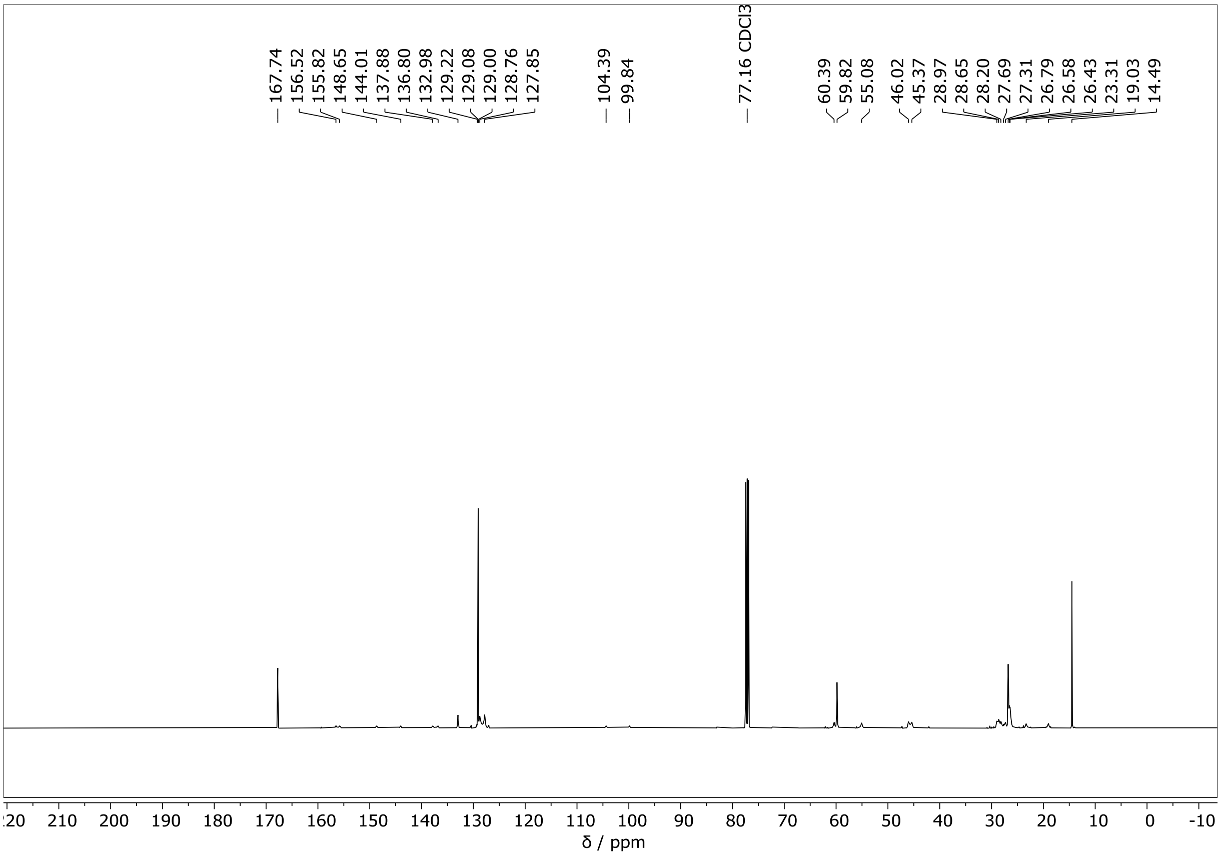

Ethyl 5-cyclohexyl-7-methyl-5,8-dihydroimidazo[1,2-*a*]pyrimidine-6-carboxylate (1h)

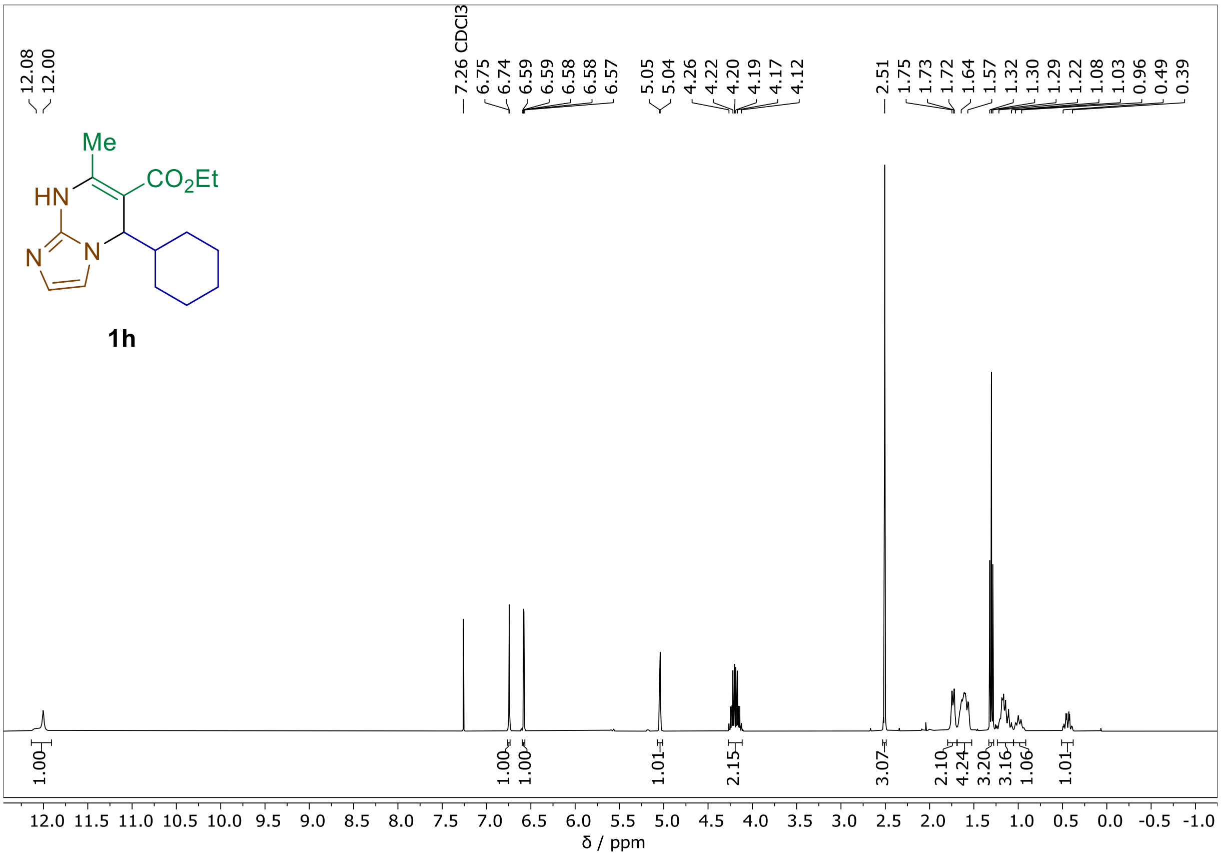

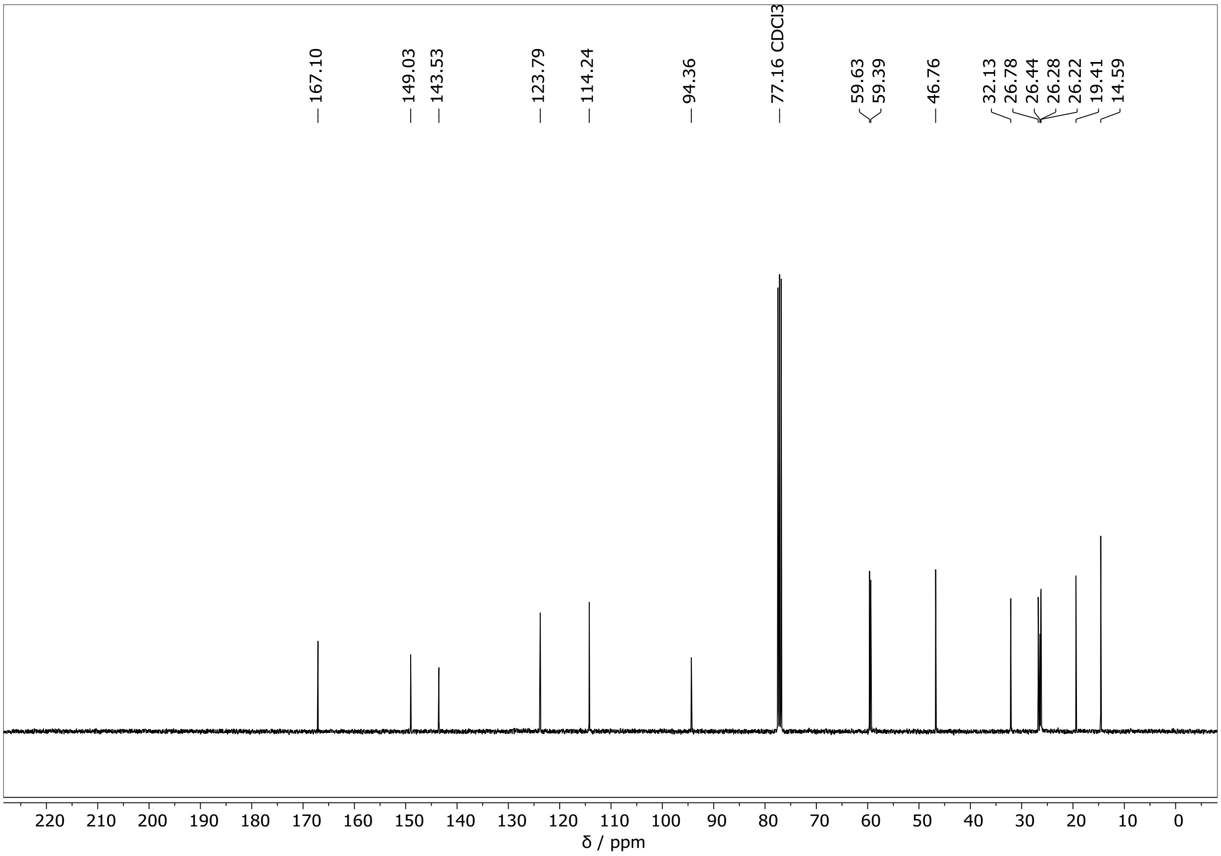

Ethyl 6-cyclopentyl-2-(dimethylamino)-4-methyl-1,6-dihydropyrimidine-5-carboxylate (1i)

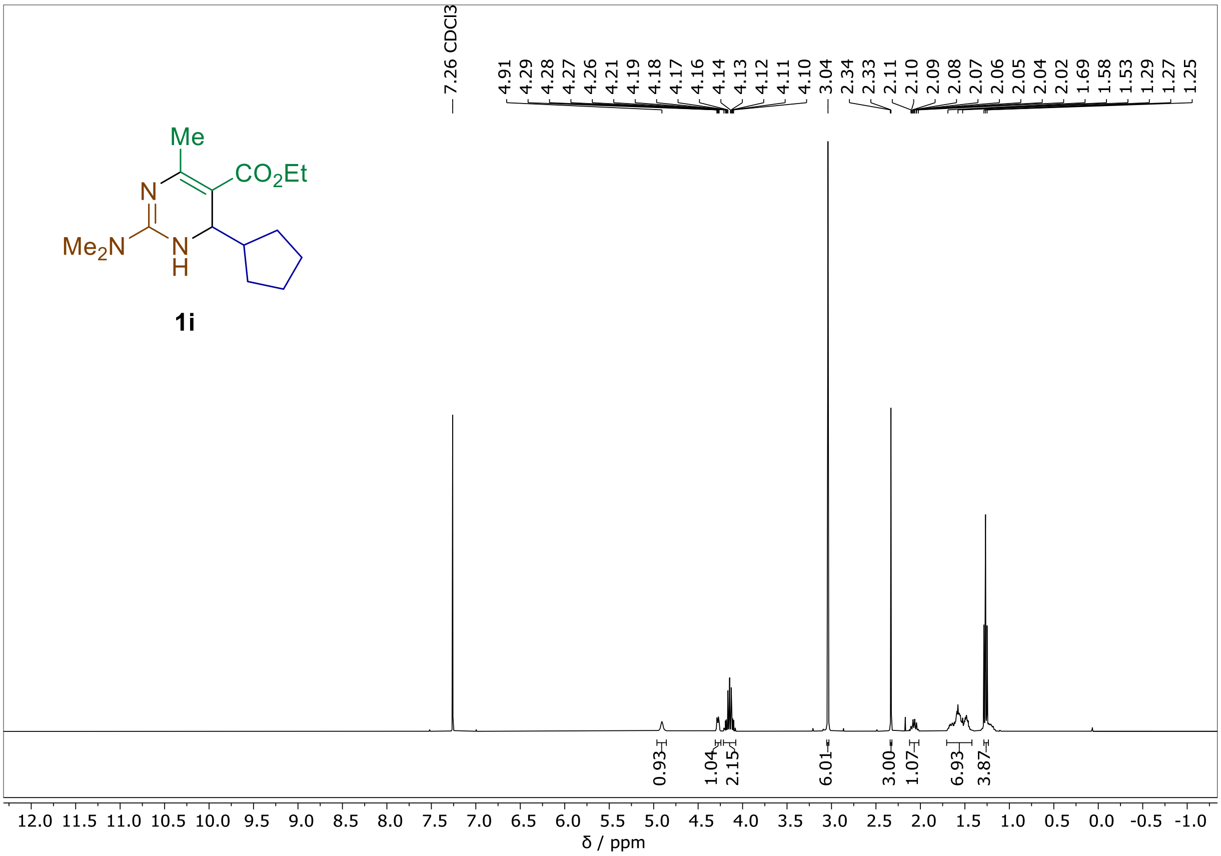

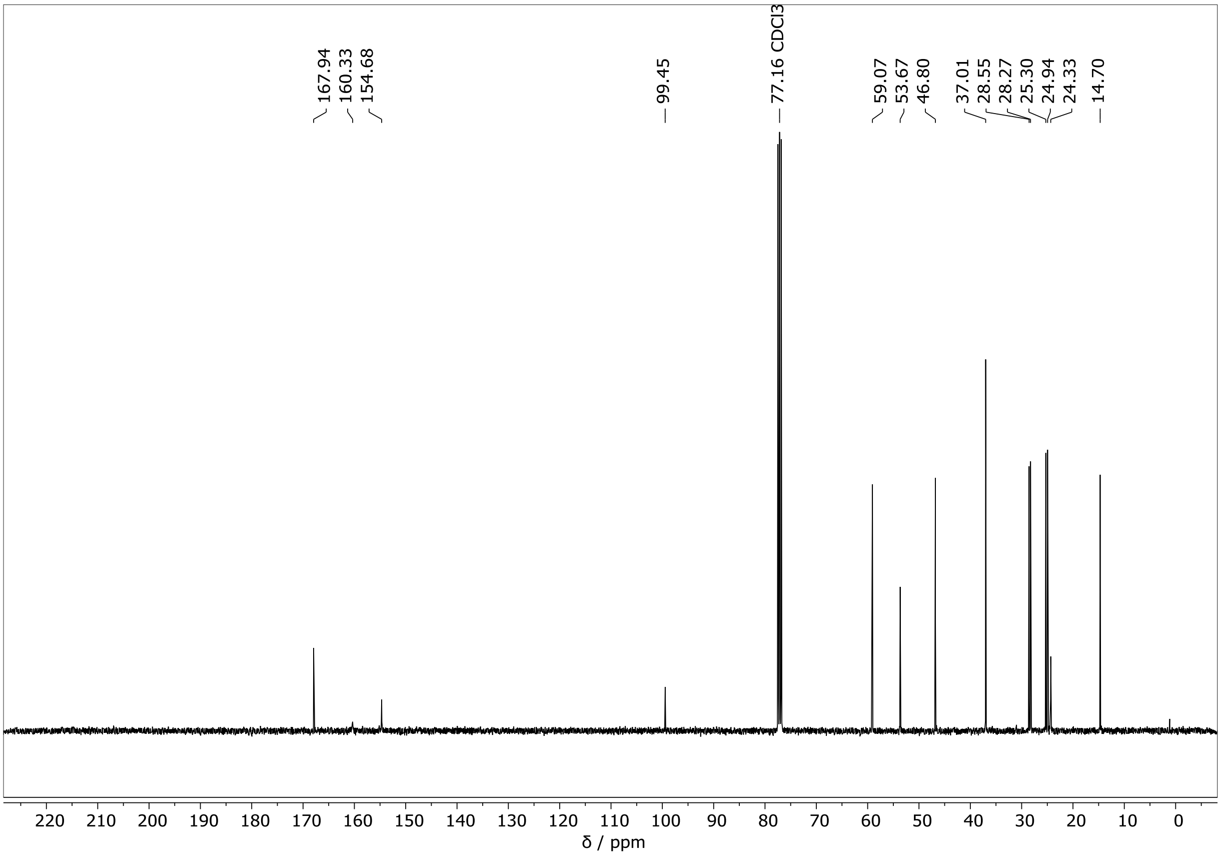

Ethyl 6-(1-(benzo[*d*][1,3]dioxol-5-yl)propan-2-yl)-2-(dimethylamino)-4-methyl-1,6-dihydropyrimidine-5-carboxylate (1j)

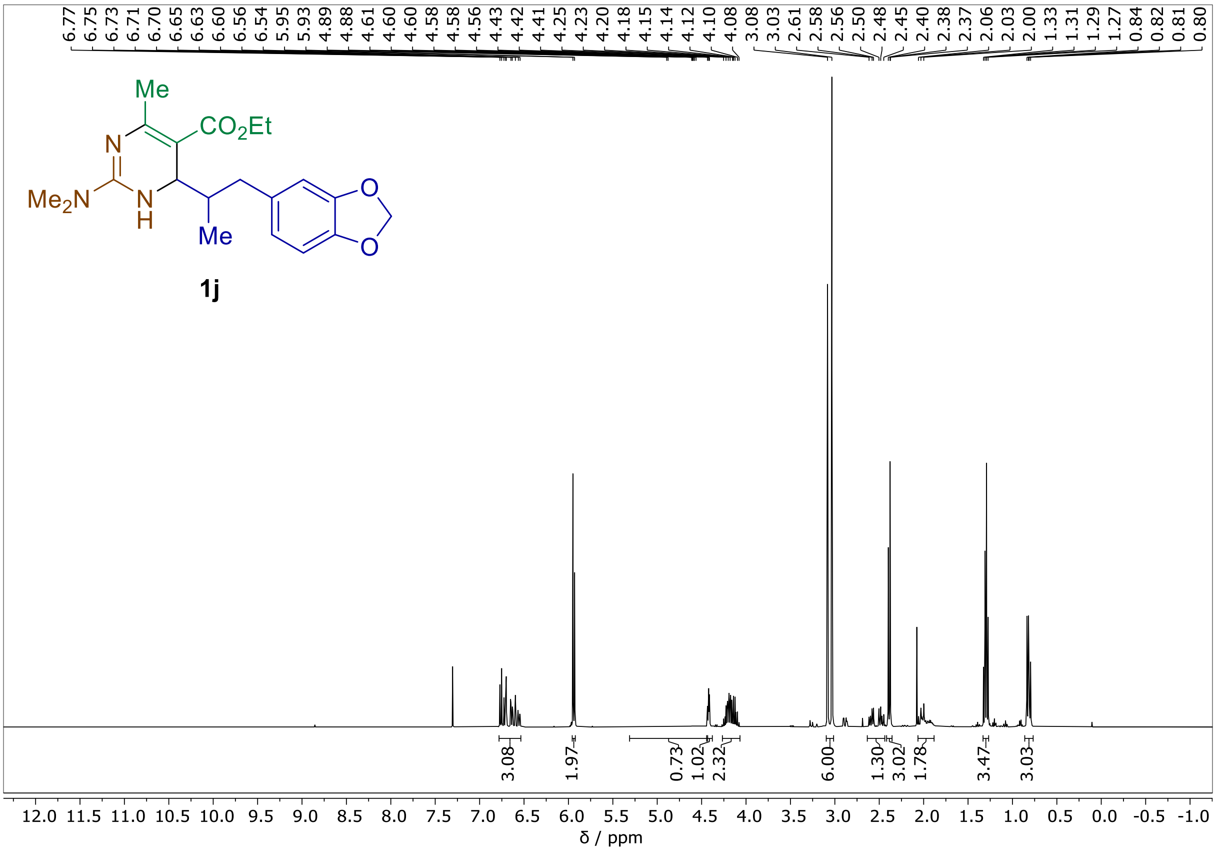

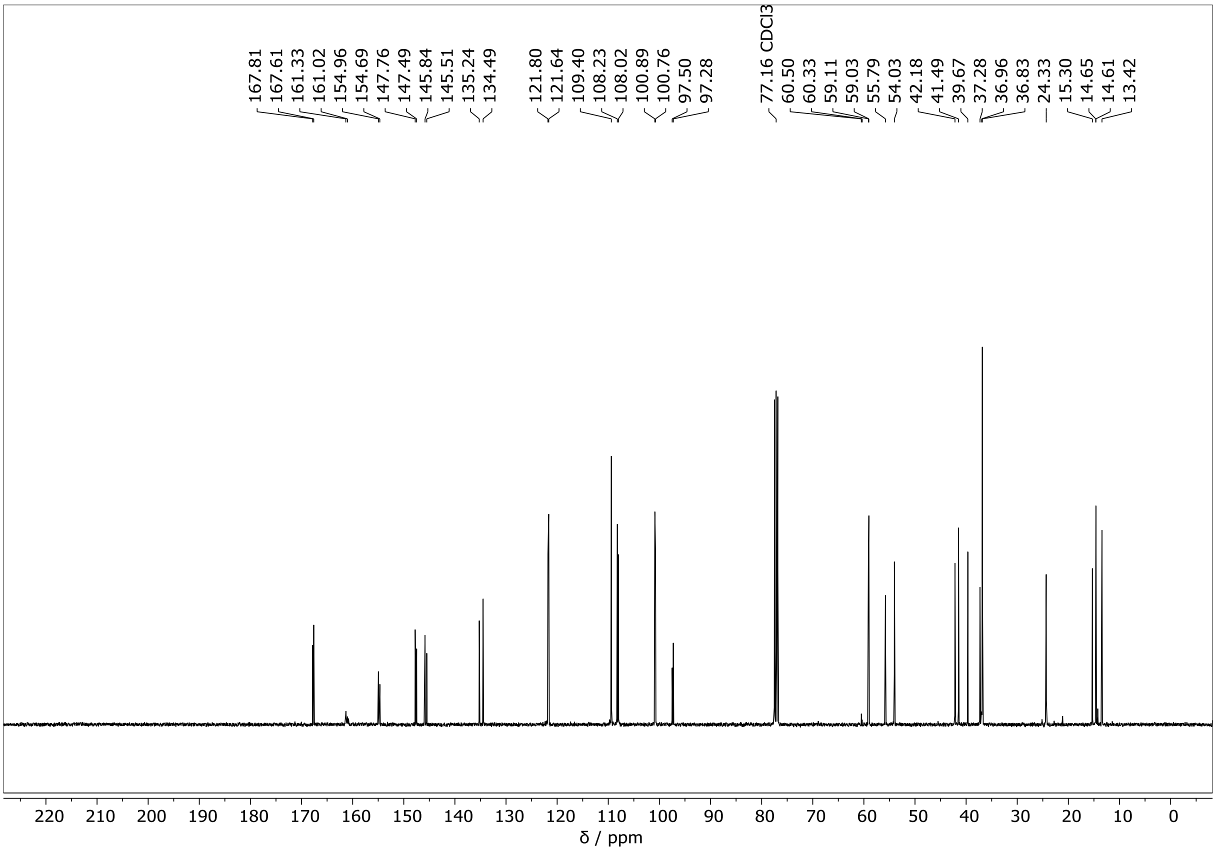

Ethyl 6-(2,2-dimethyl-1,3-dioxolan-4-yl)-2-(dimethylamino)-4-methyl-1,6-dihydropyrimidine-5-carboxylate (1k)

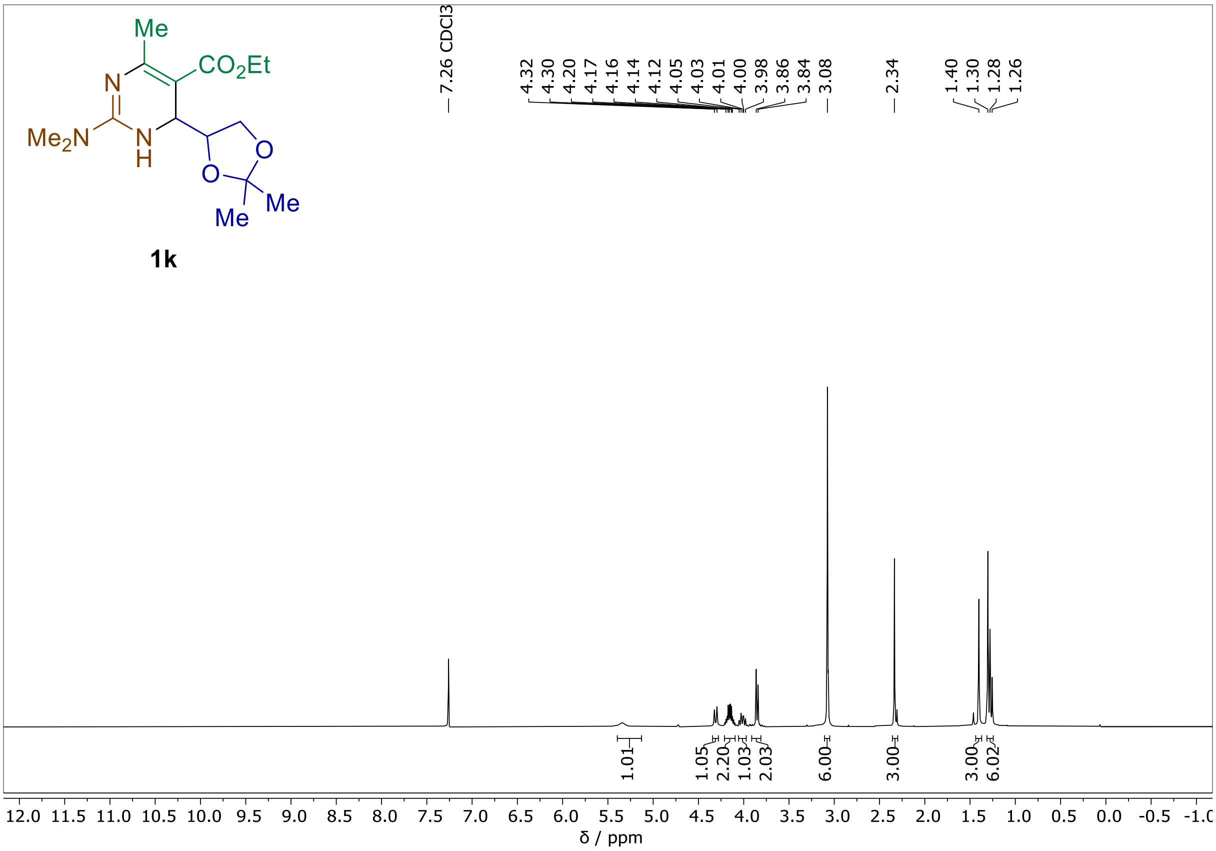

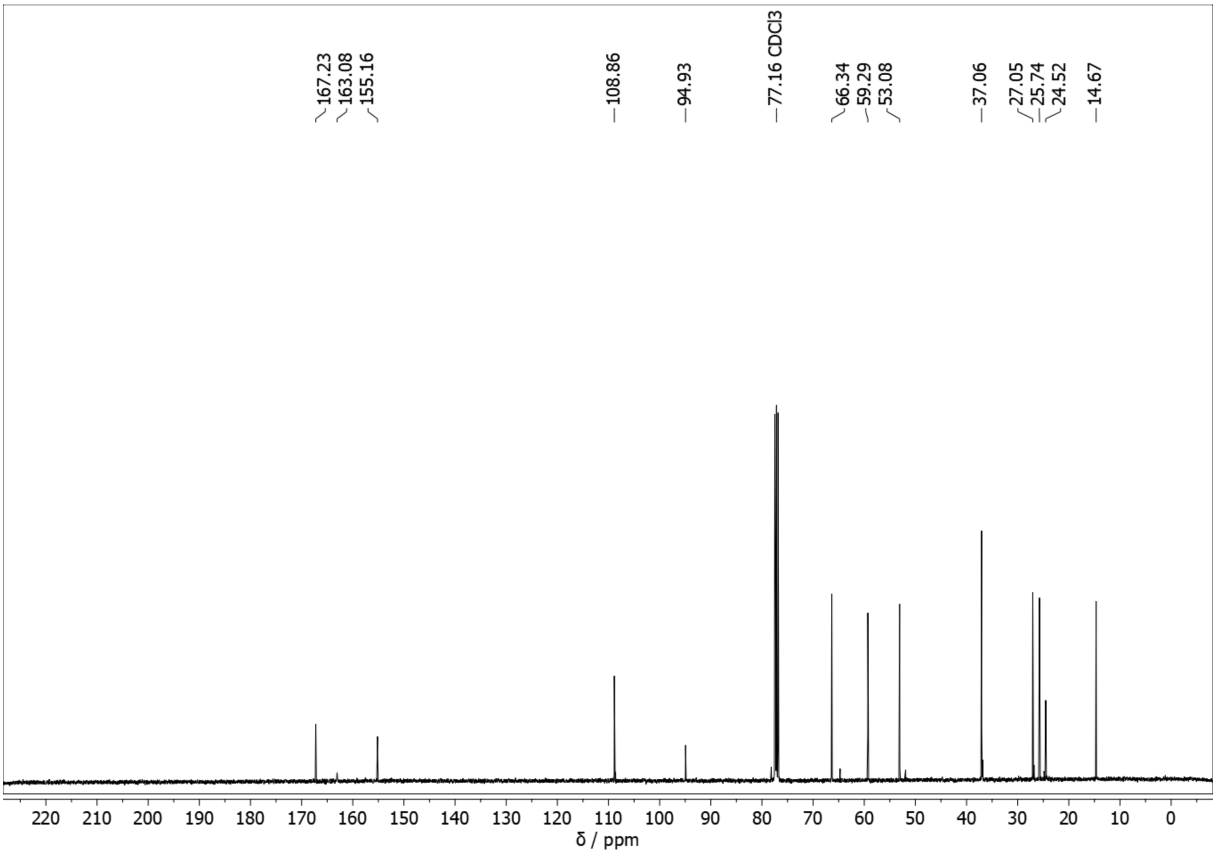

Ethyl 2-(dimethylamino)-4-methyl-6-(tetrahydrofuran-3-yl)-1,6-dihydropyrimidine-5-carboxylate (1l)

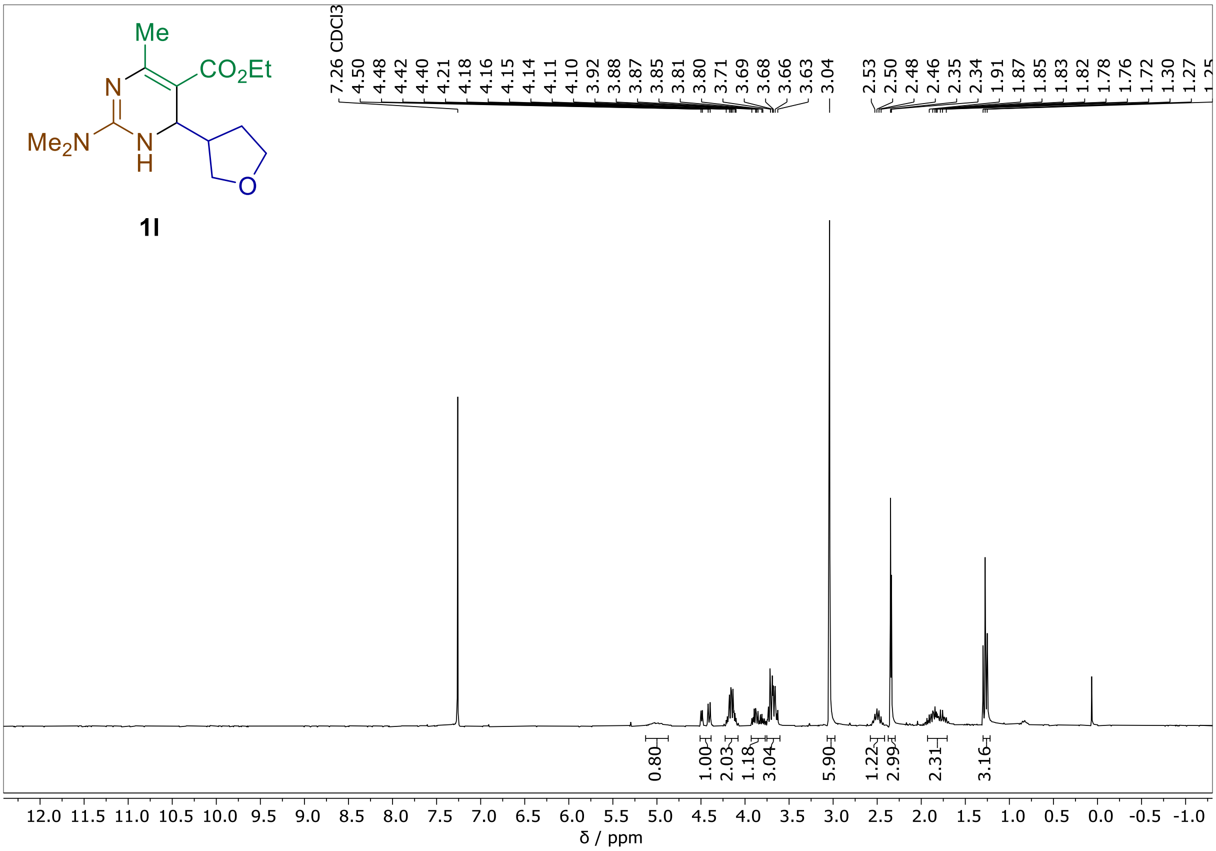

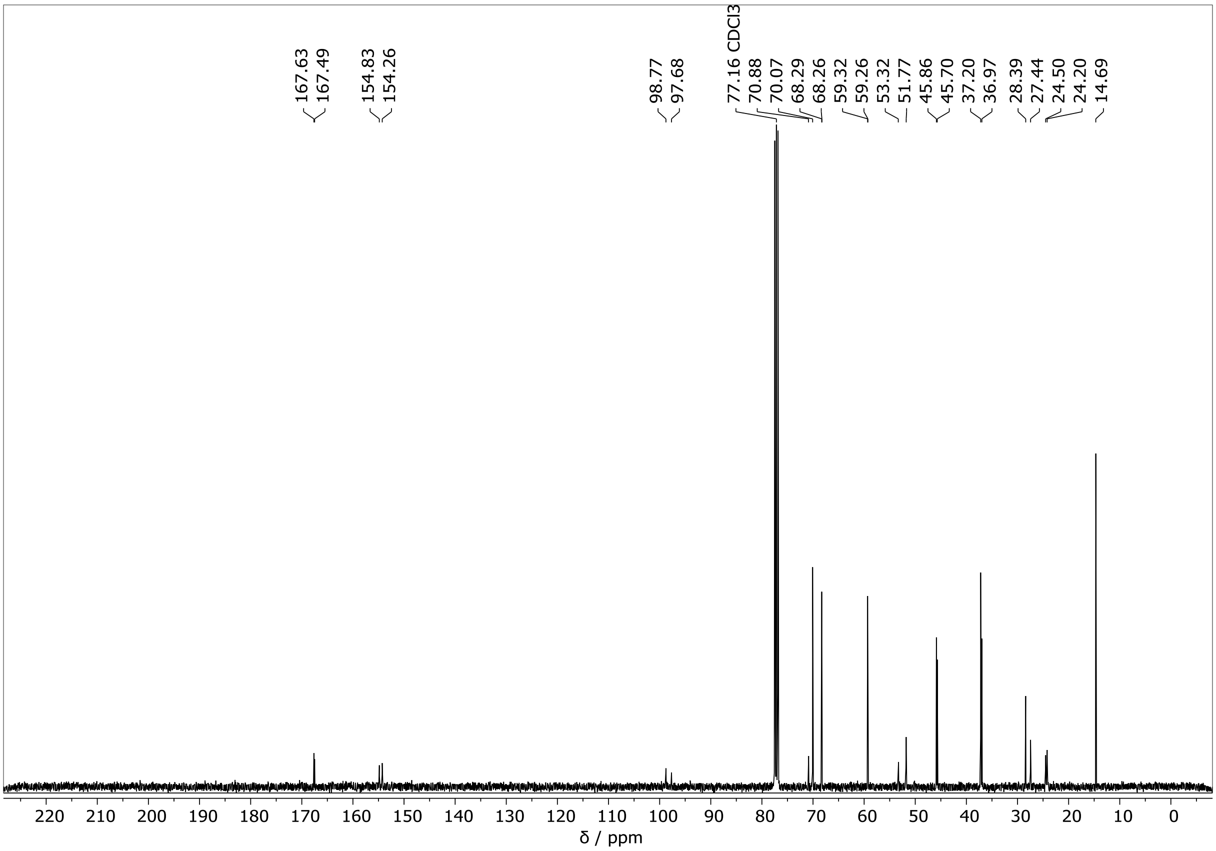

Ethyl 2-(dimethylamino)-6-isopropyl-4-methyl-1,6-dihydropyrimidine-5-carboxylate (1m)

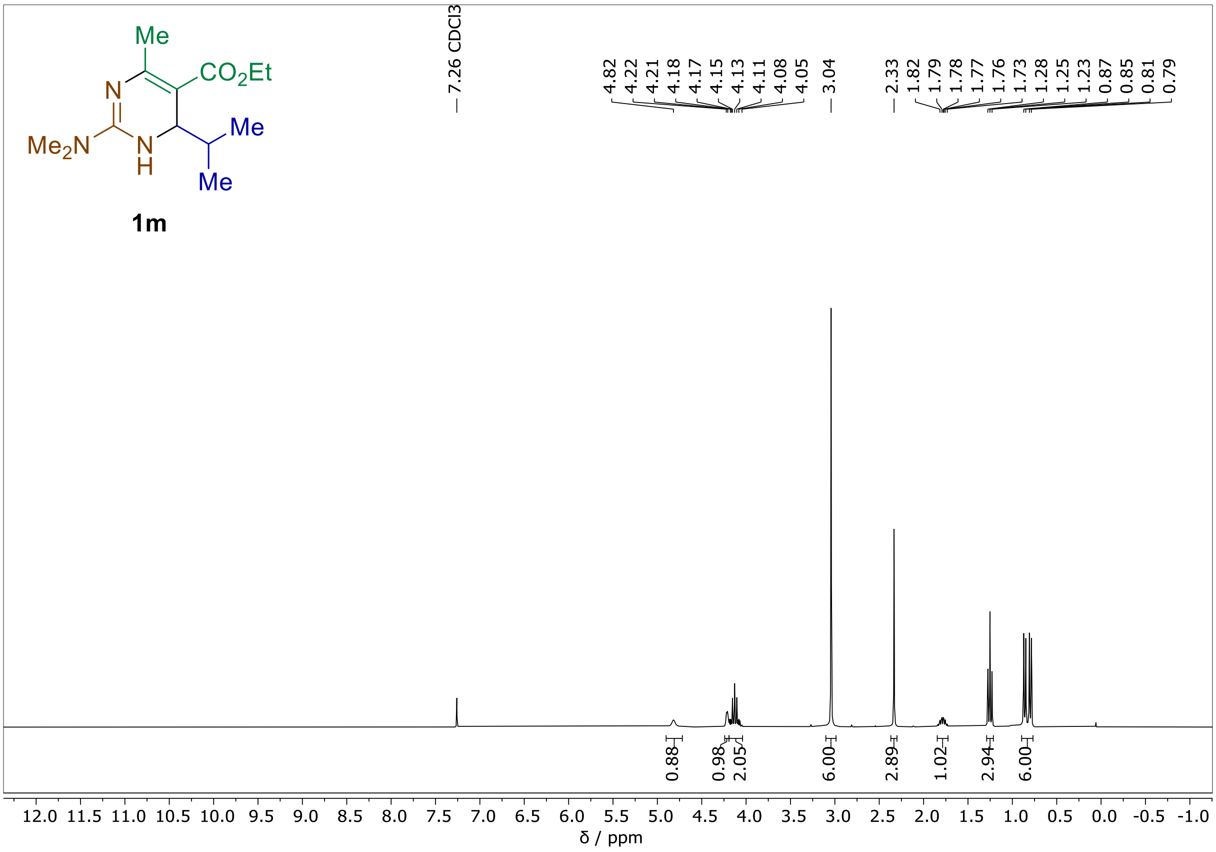

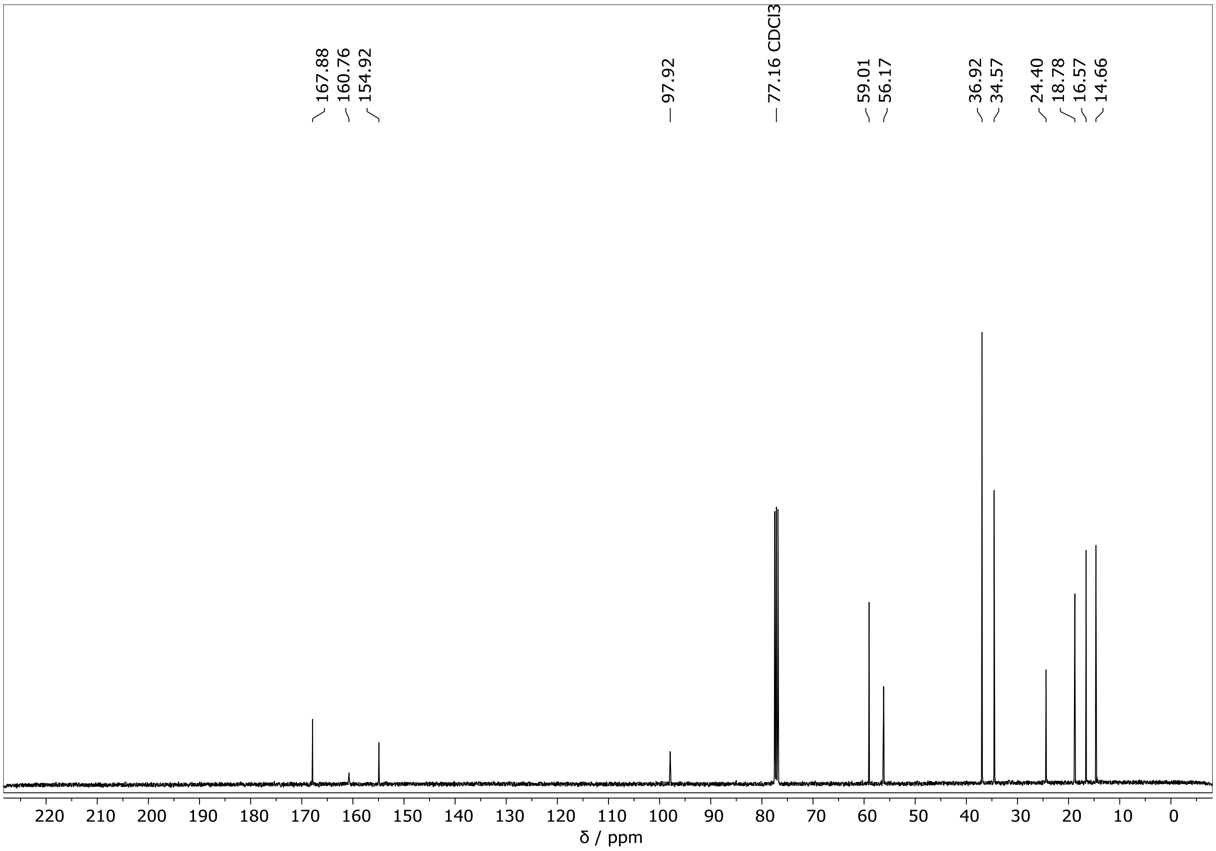

Ethyl 2-(dimethylamino)-4-methyl-6-(6-methylhept-5-en-2-yl)-1,6-dihydropyrimidine-5-carboxylate (1n)

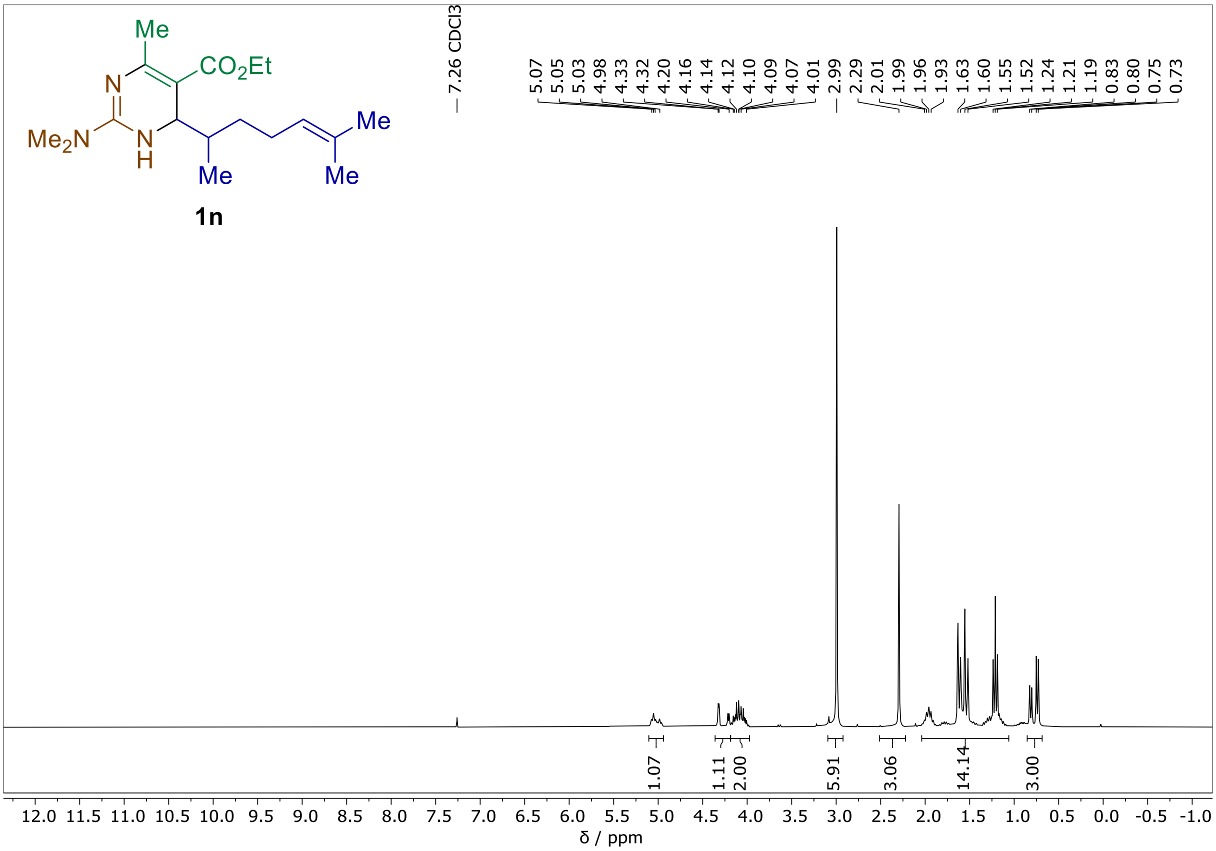

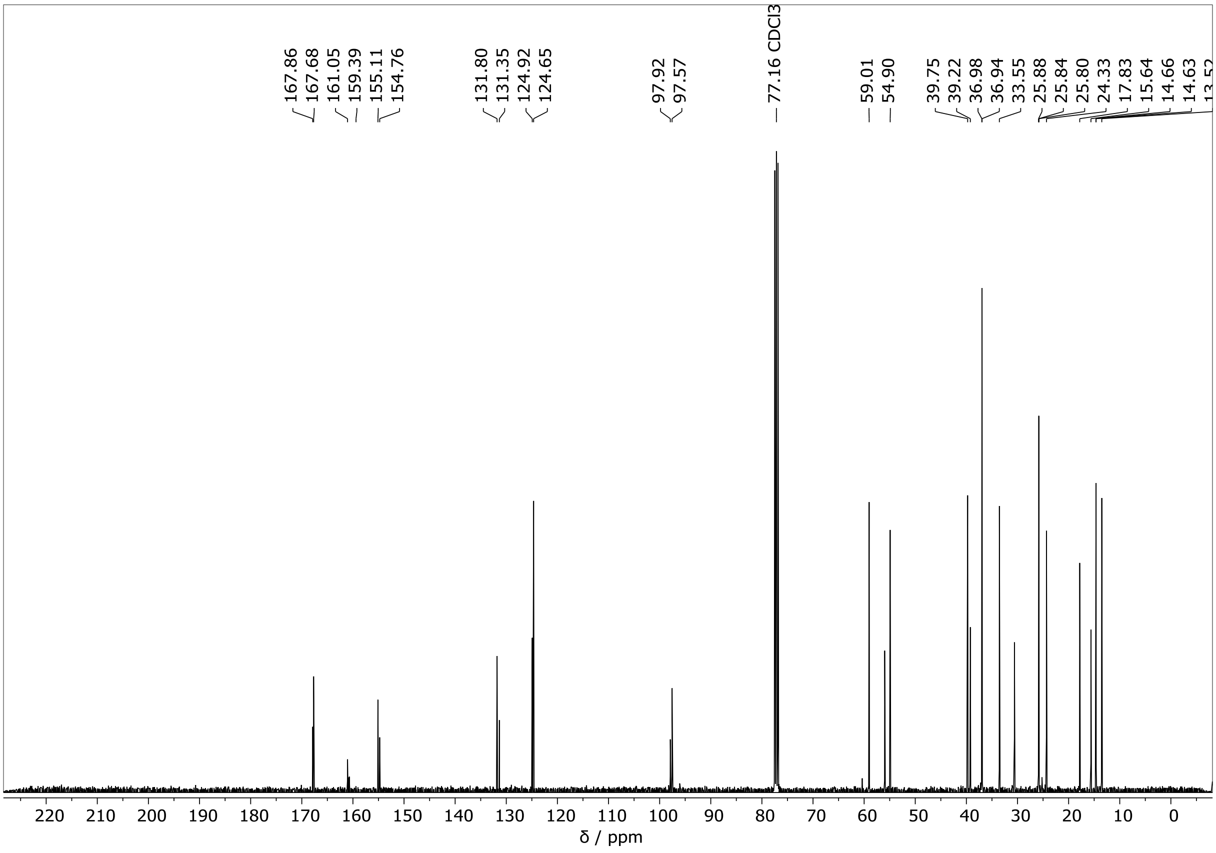

Ethyl 2-(dimethylamino)-6-(1-(4-isopropylphenyl)propan-2-yl)-4-methyl-1,6-dihydropyrimidine-5-carboxylate (1o)

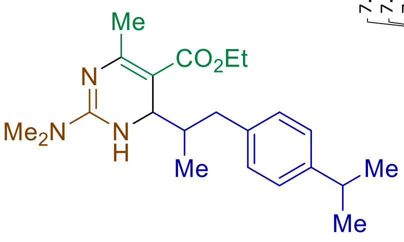

1o

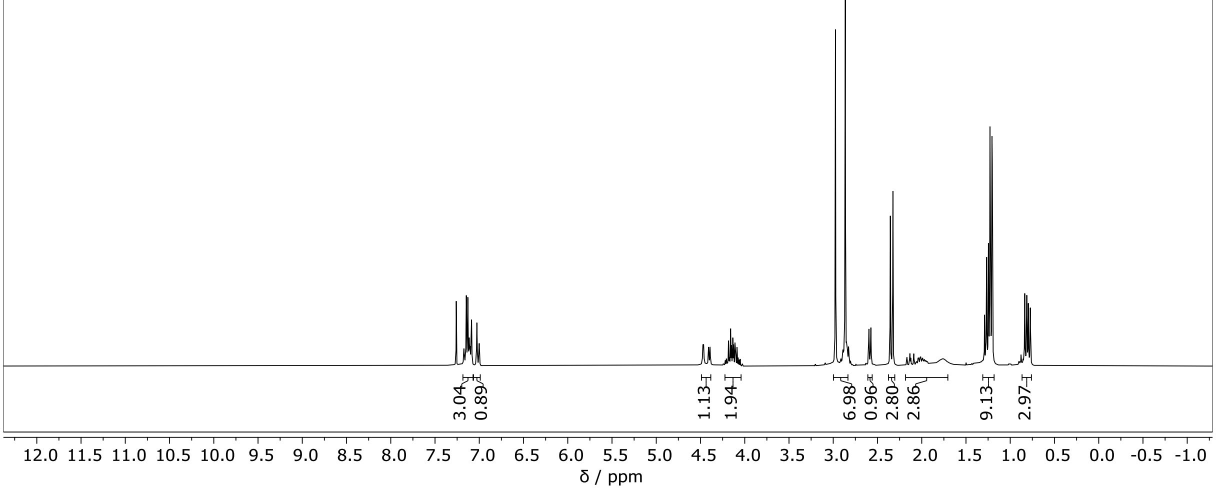

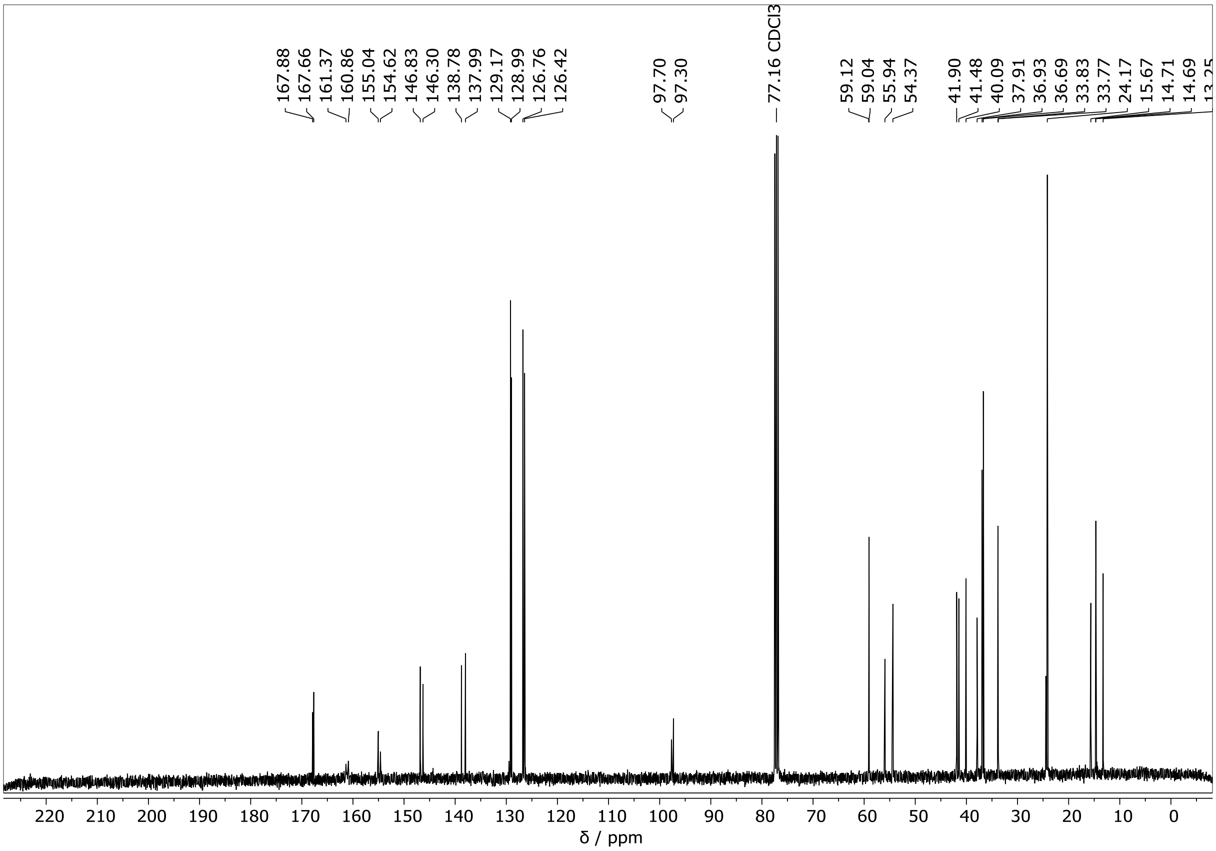

Ethyl 6-benzyl-2-(dimethylamino)-4-methyl-1,6-dihydropyrimidine-5-carboxylate (1p)

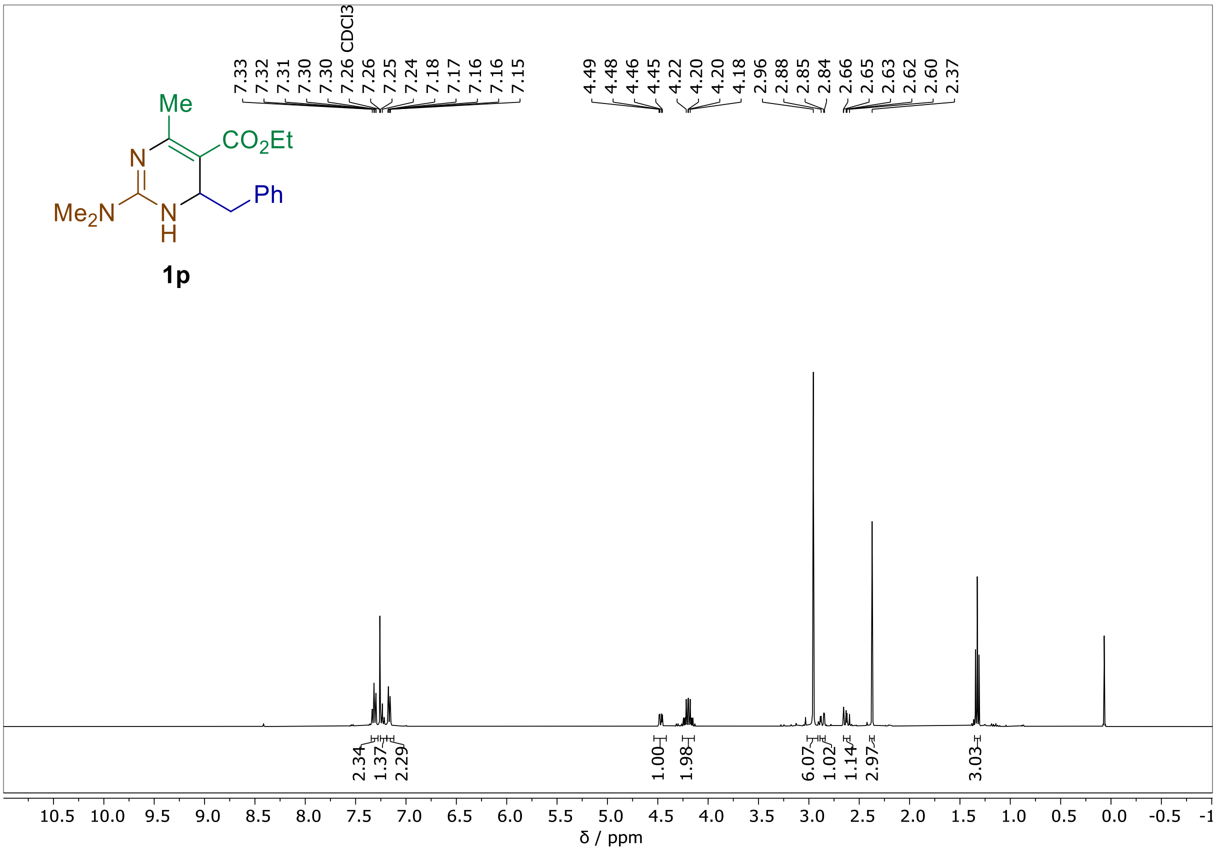

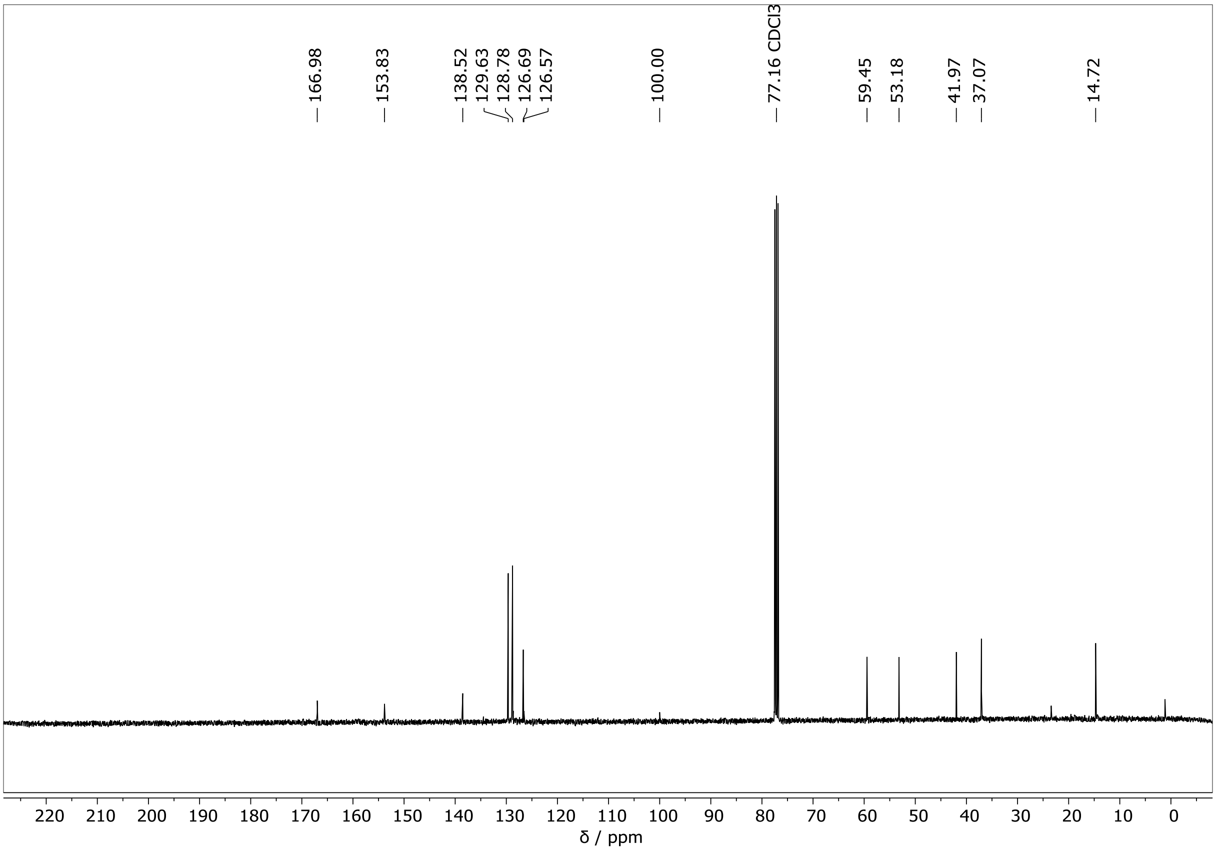

Ethyl 6-(*tert*-butyl)-2-(dimethylamino)-4-methyl-1,6-dihydropyrimidine-5-carboxylate (1q)

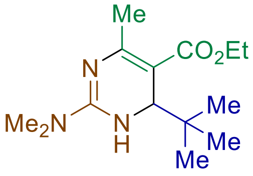

1q

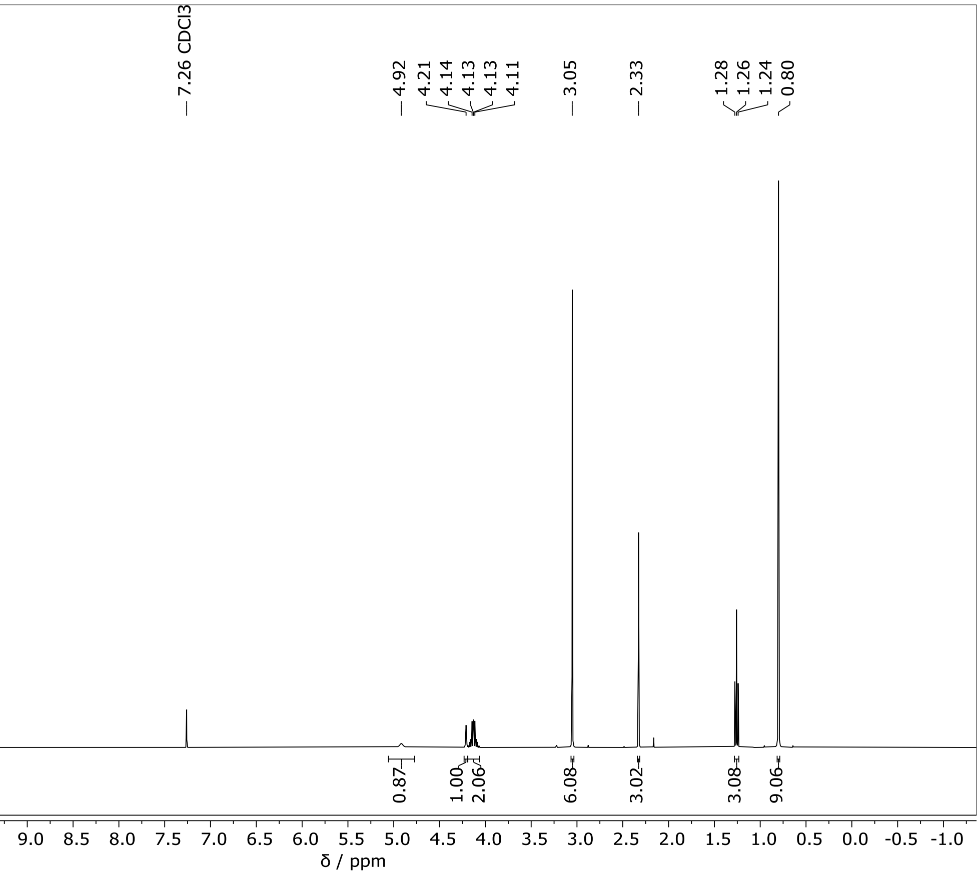

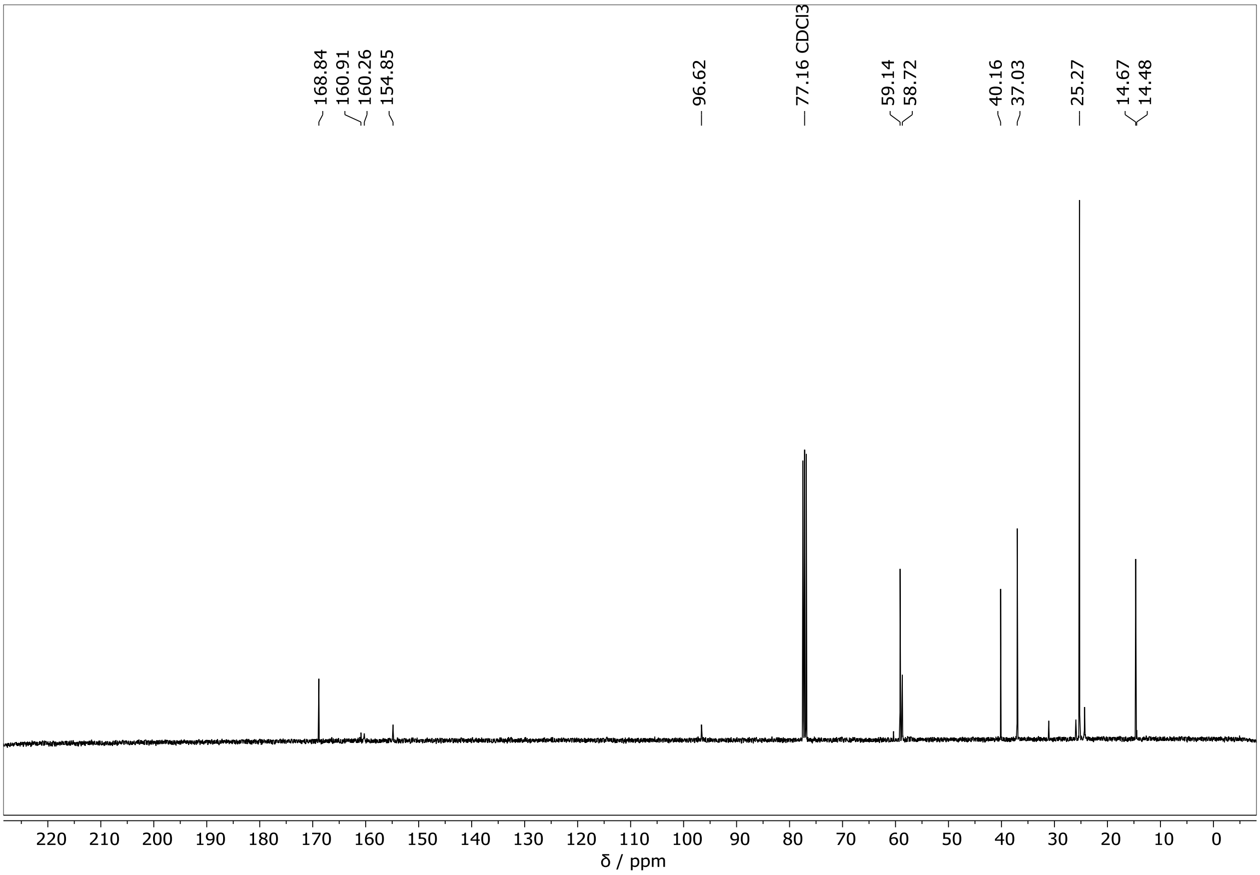

Ethyl 6-(1-(*tert*-butoxycarbonyl)pyrrolidin-3-yl)-2-(dimethylamino)-4-methyl-1,6-dihydropyrimidine-5-carboxylate (1r)

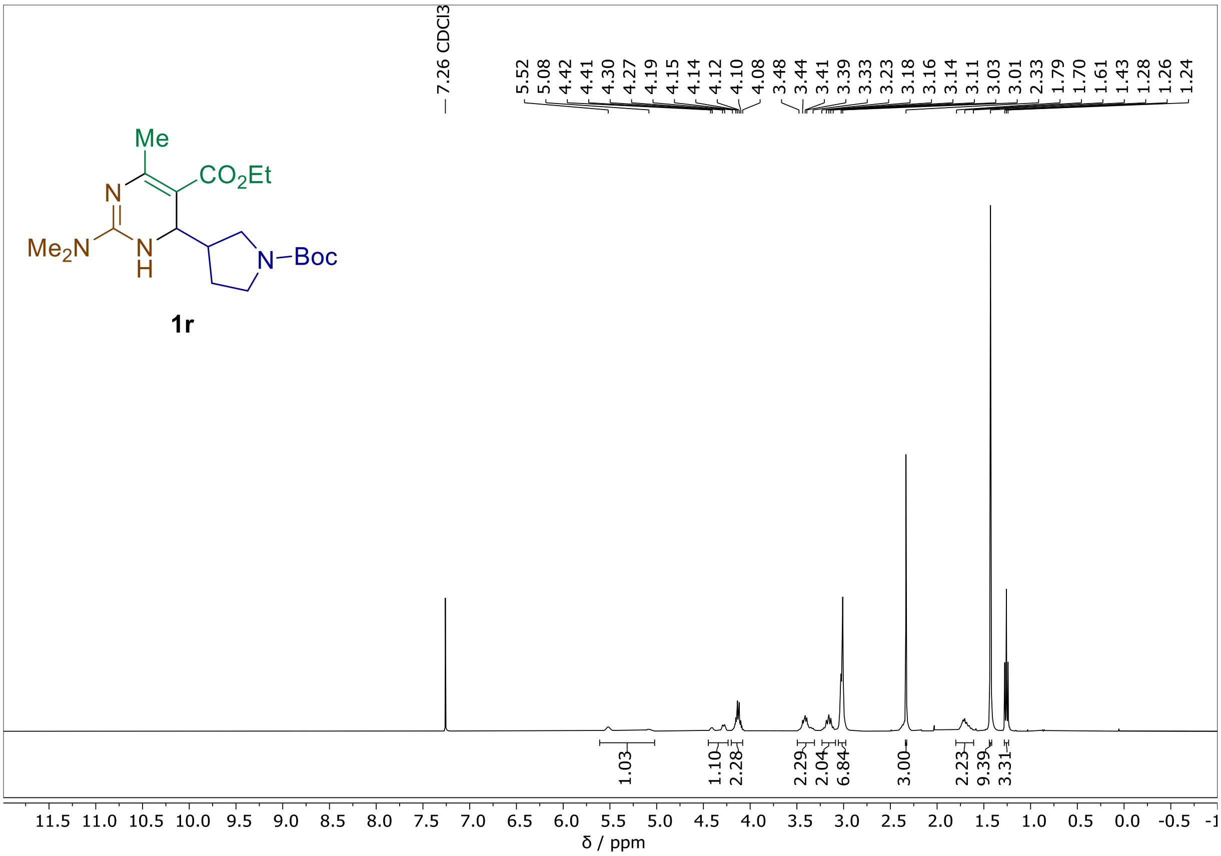

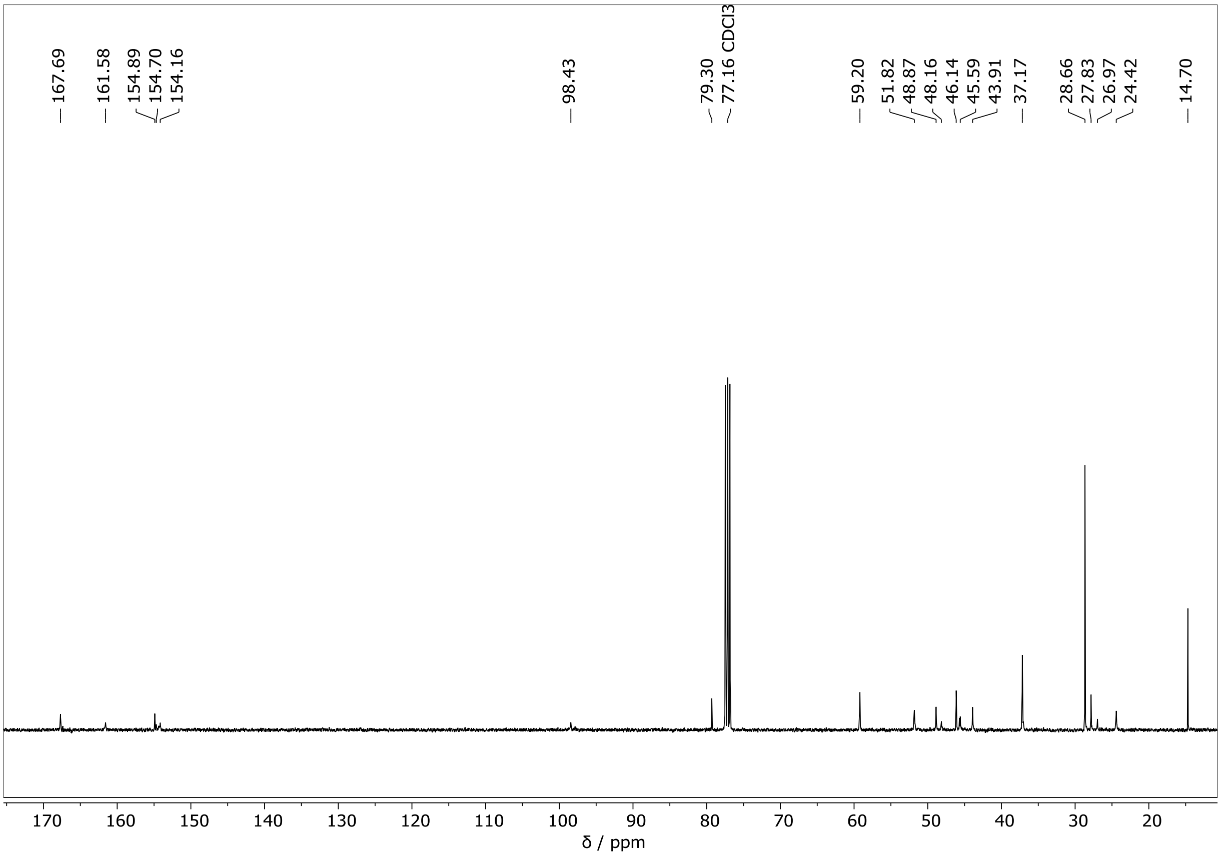

Ethyl 6-butyl-2-(dimethylamino)-4-methyl-1,6-dihydropyrimidine-5-carboxylate (1s)

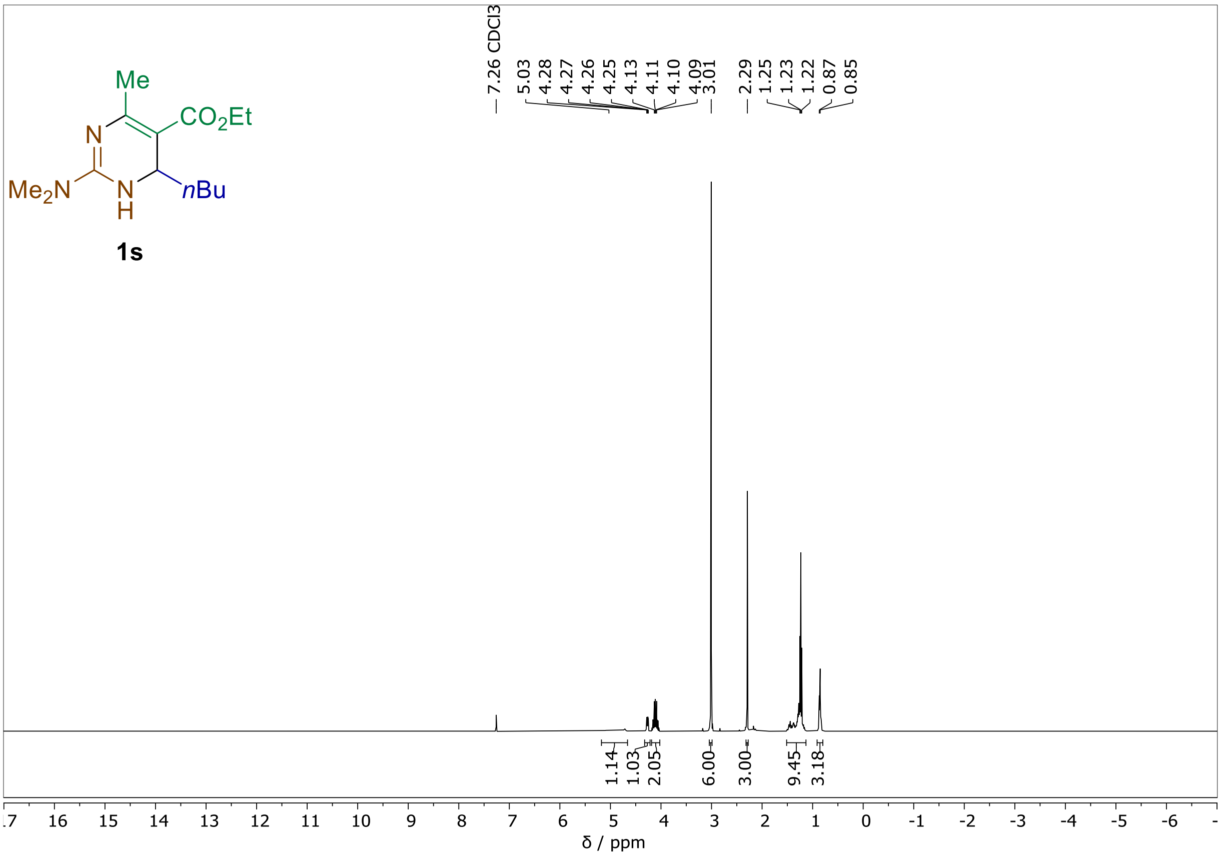

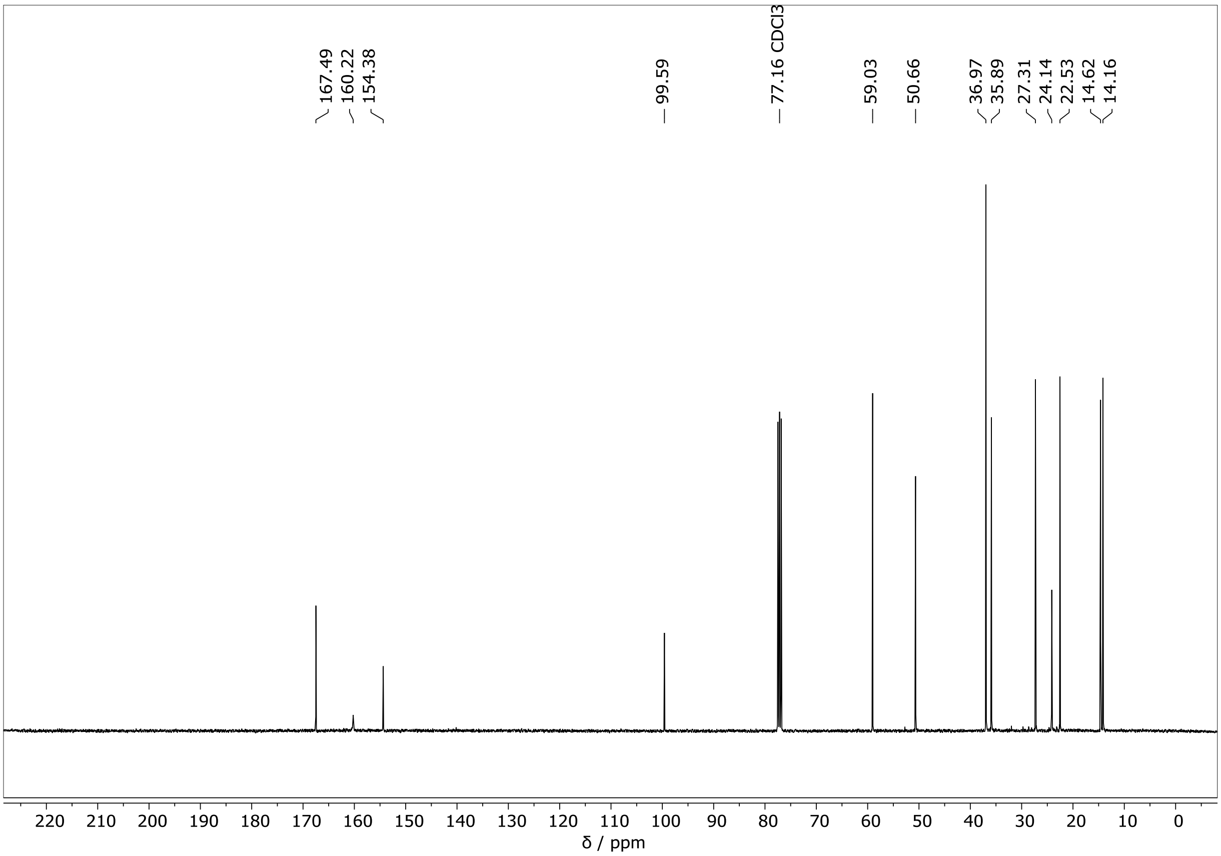

Ethyl 2-(dimethylamino)-4-methyl-6-((3a*S*,5a*R*,8a*R*,8b*S*)-2,2,7,7-tetramethyltetrahydro-5*H*-bis([1,3]dioxolo)[4,5-*b*:4',5'-*d*]pyran-5-yl)-1,6-dihydropyrimidine-5-carboxylate (1t)

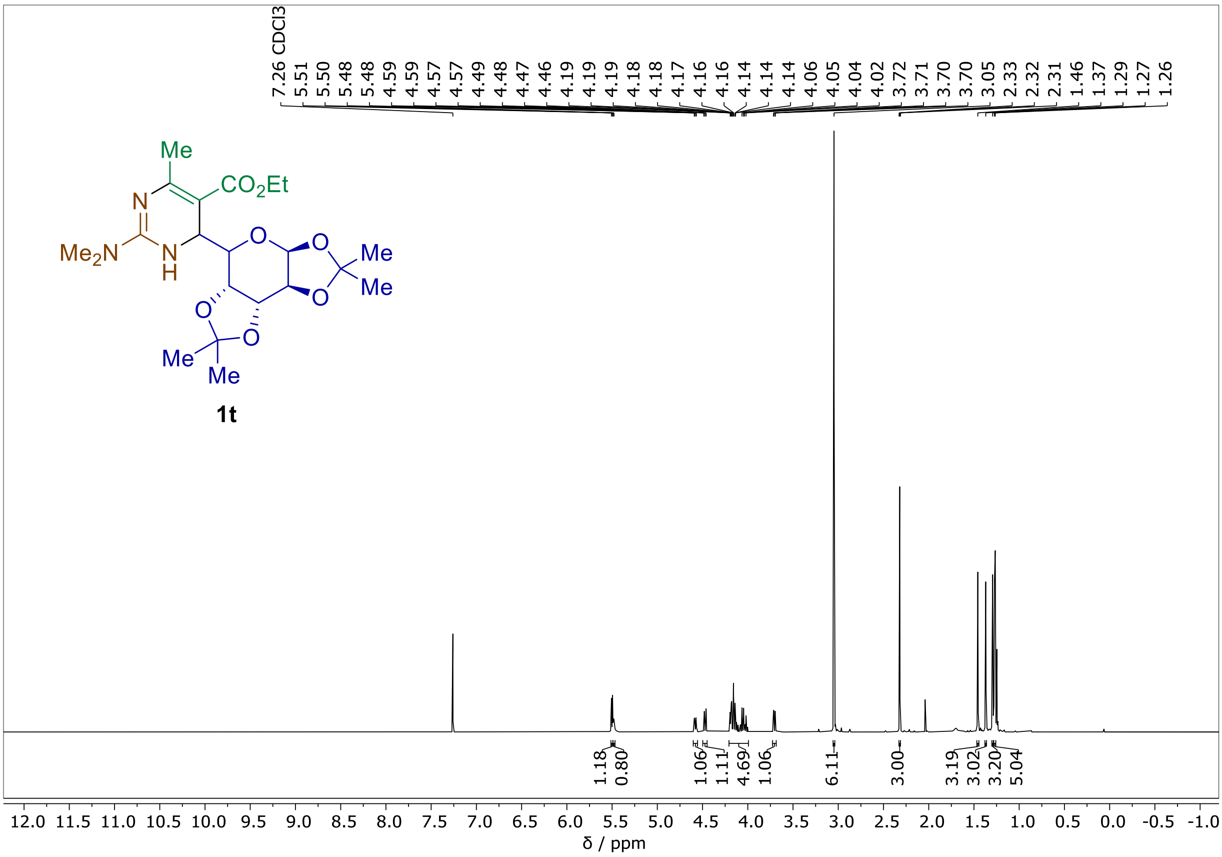

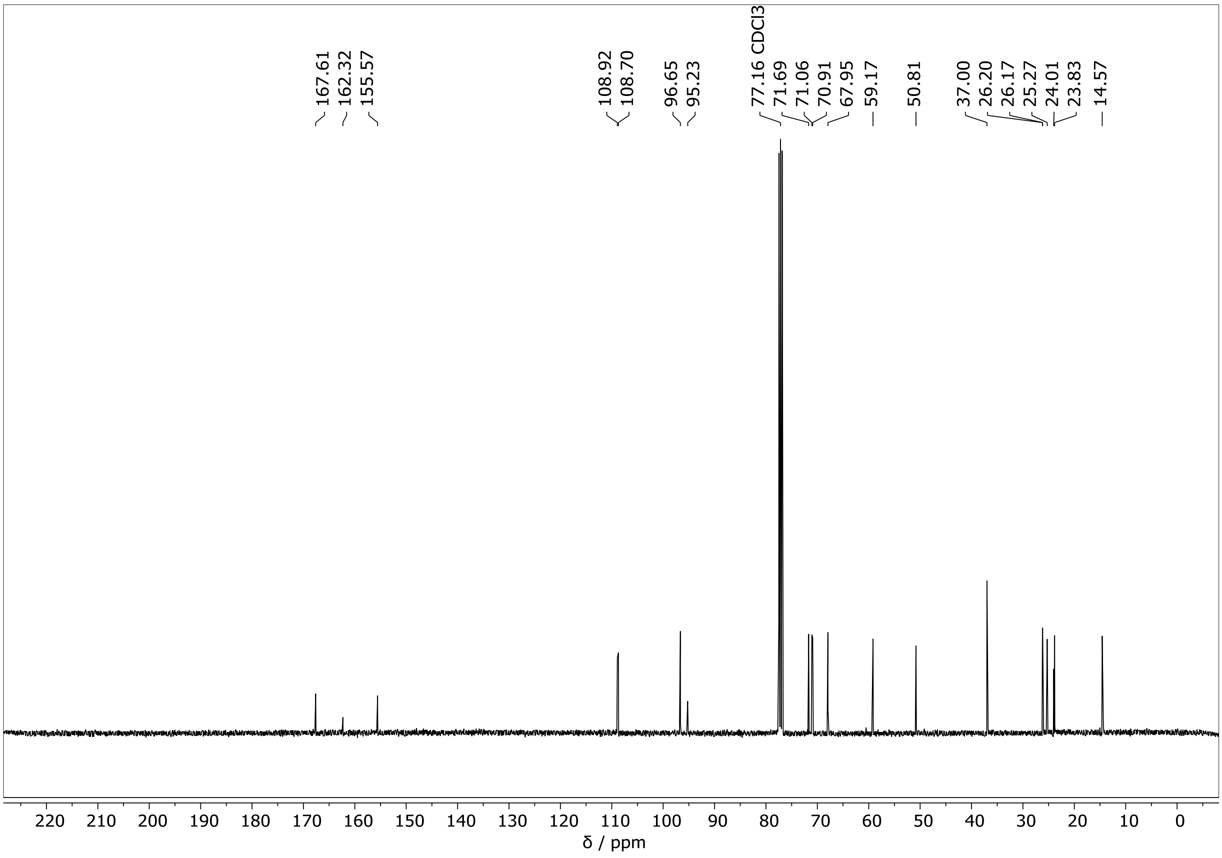

Ethyl 6-(cyclopropylmethyl)-2-(dimethylamino)-4-methyl-1,6-dihydropyrimidine-5-carboxylate (1u)

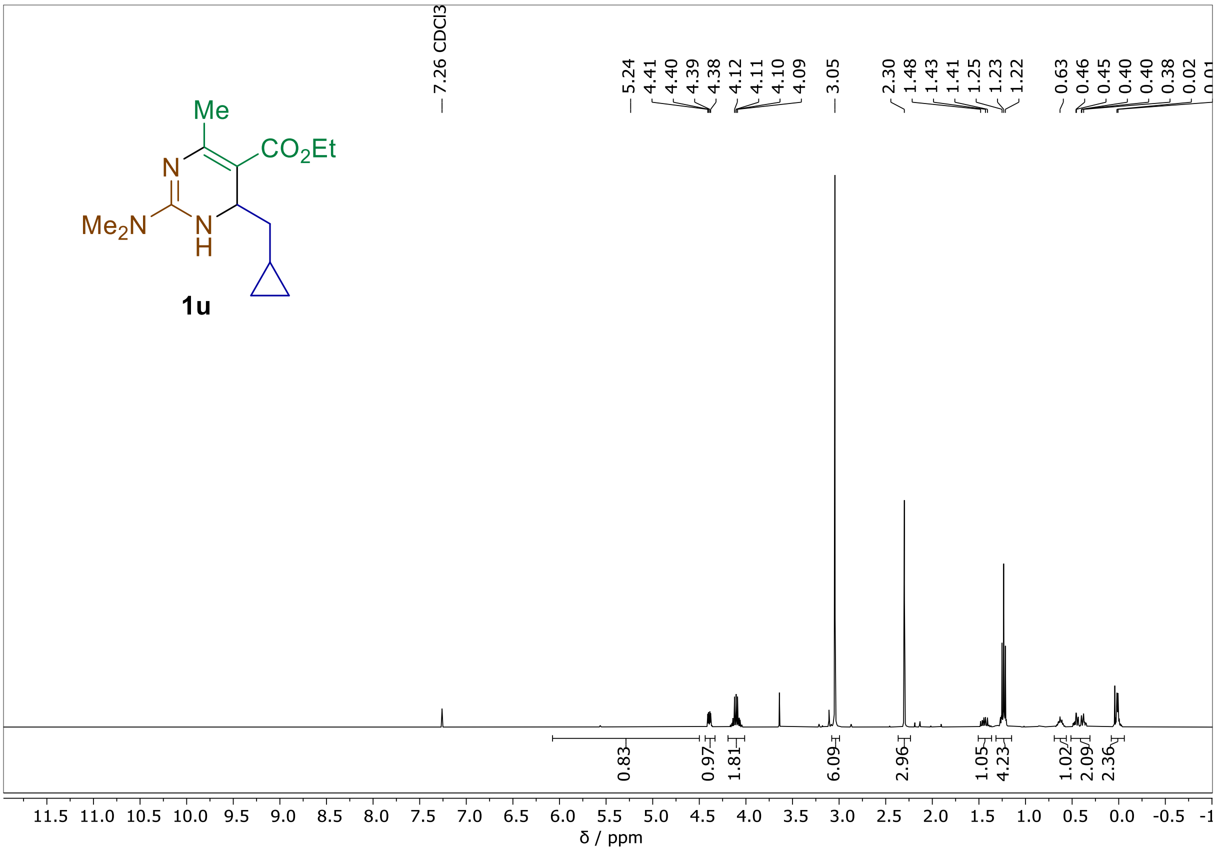

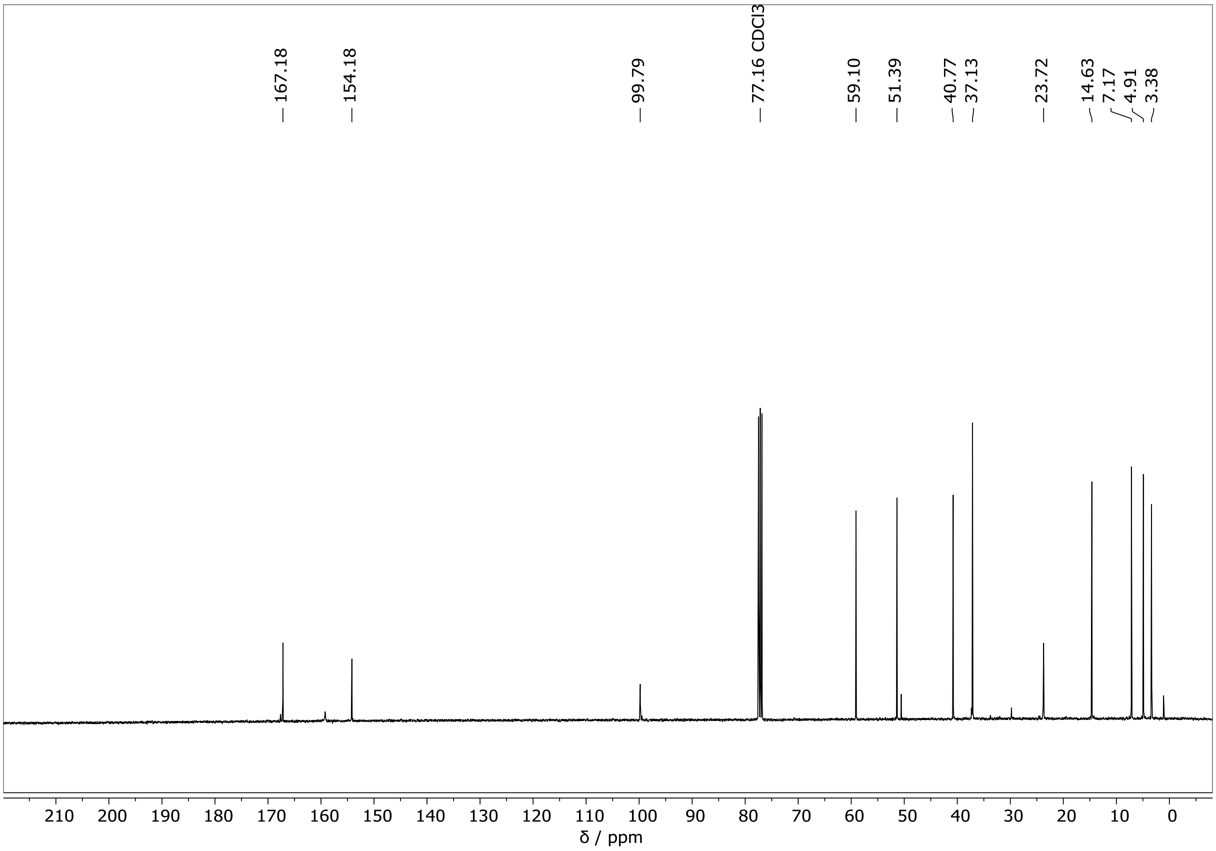

Giese addition products

Dimethyl 2-cyclohexylsuccinate (3a)

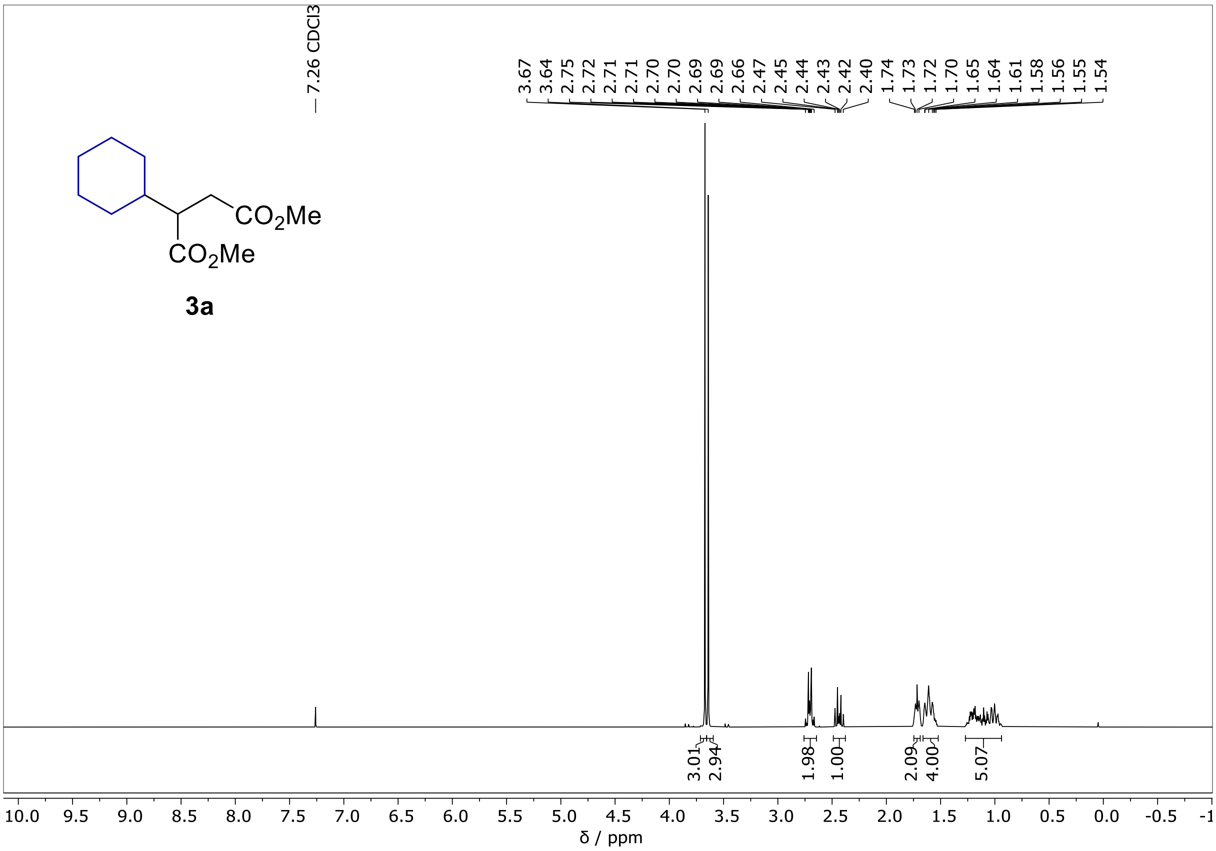

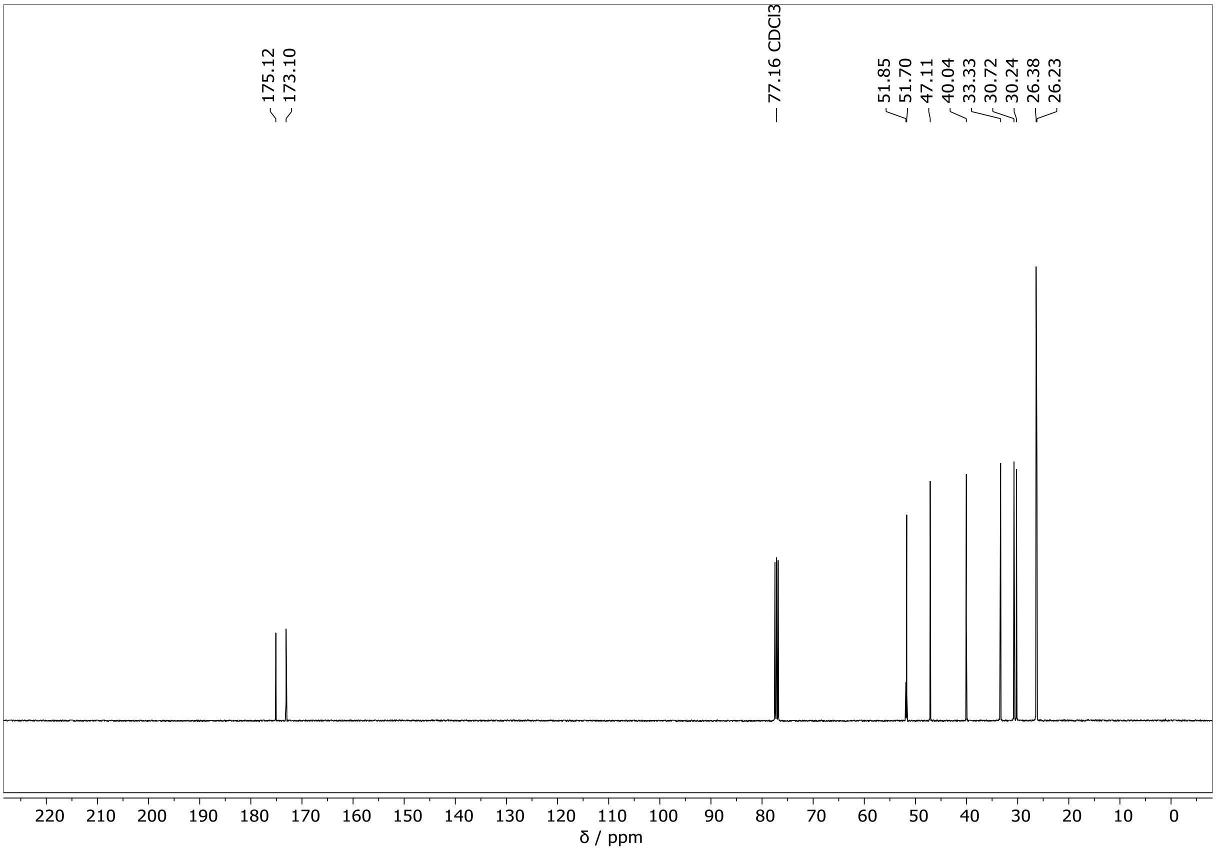

Ethyl 4-cyclohexyl-2-oxochromane-3-carboxylate (3b)

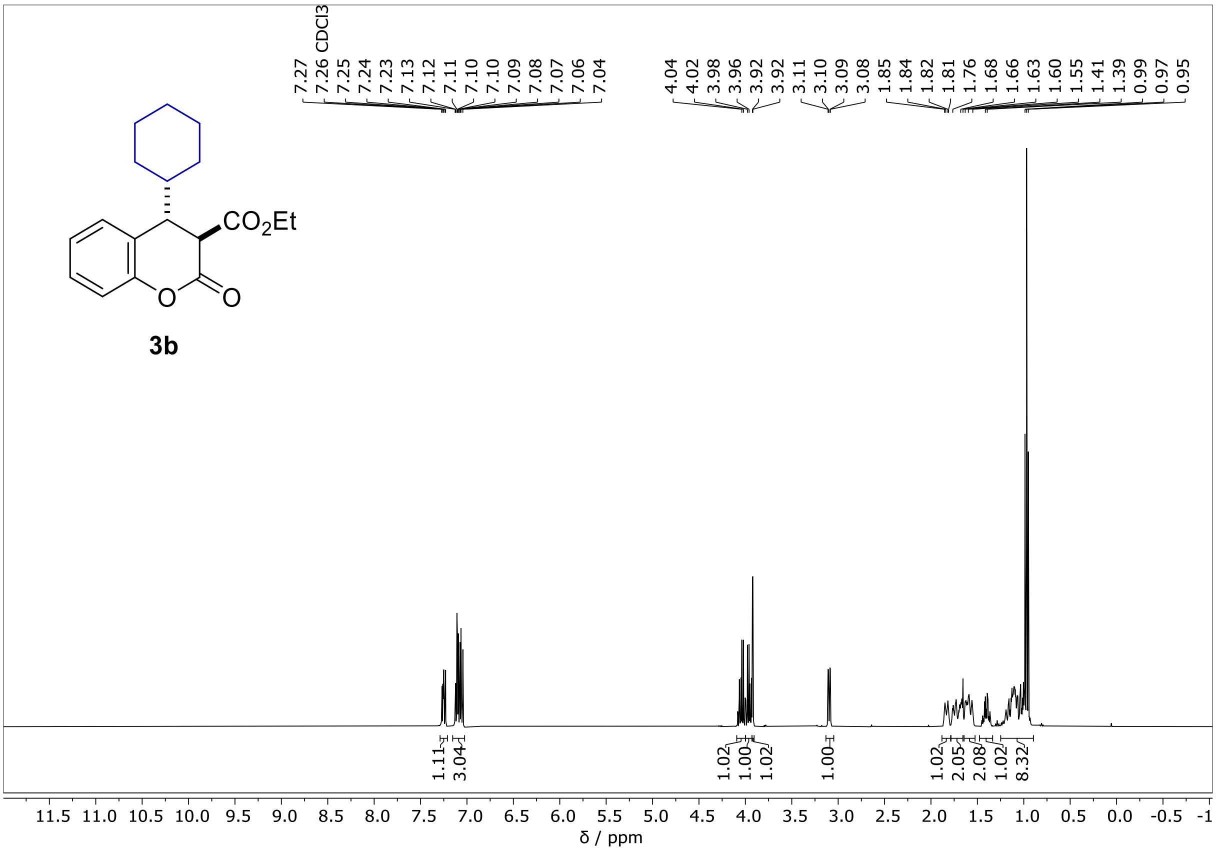

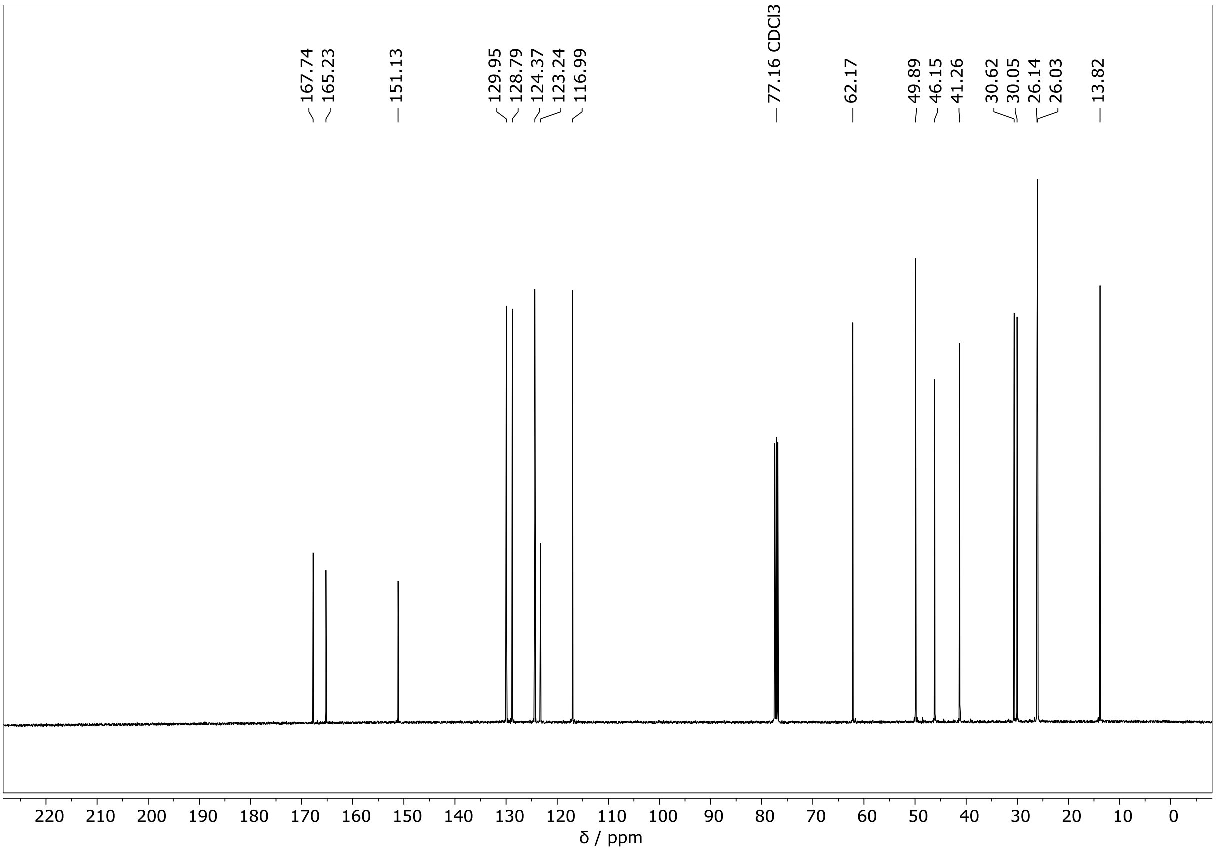

Benzyl 3-cyclohexyl-2-methylpropanoate (3c)

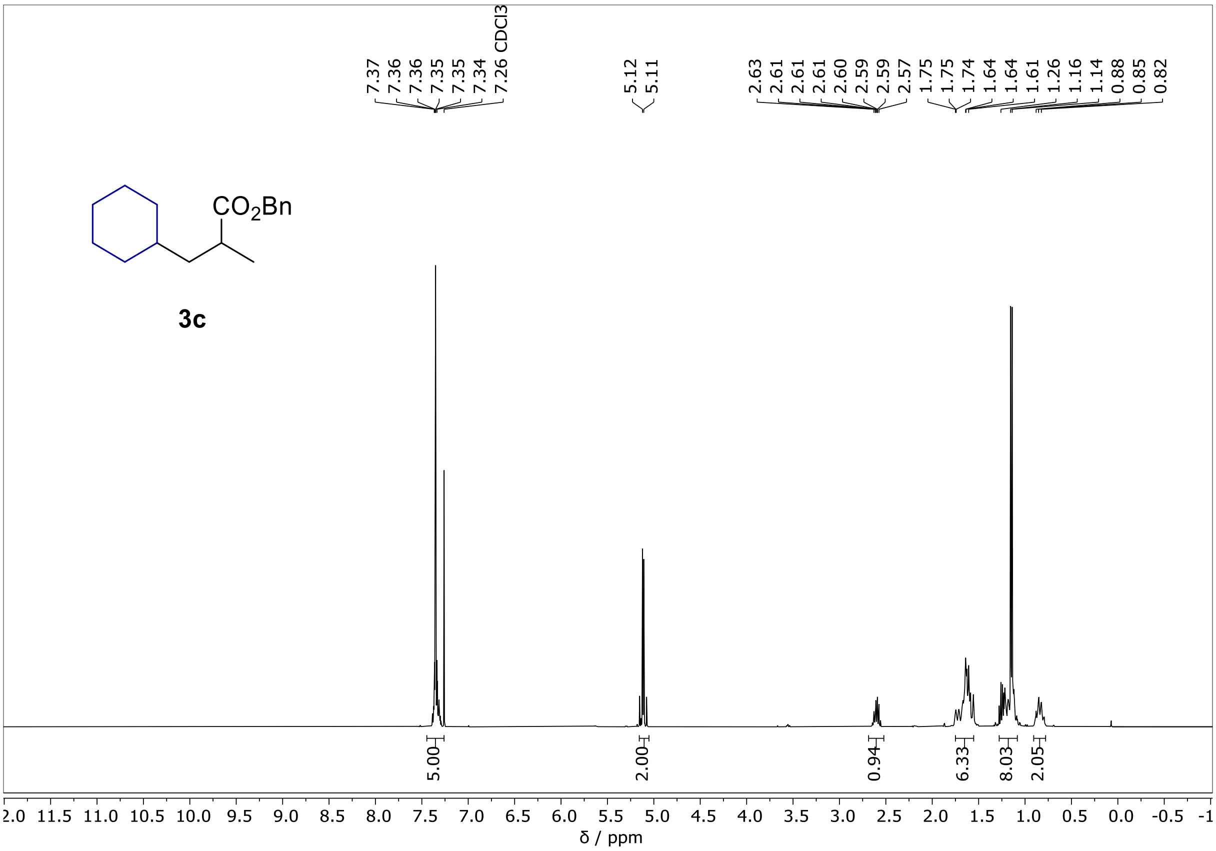

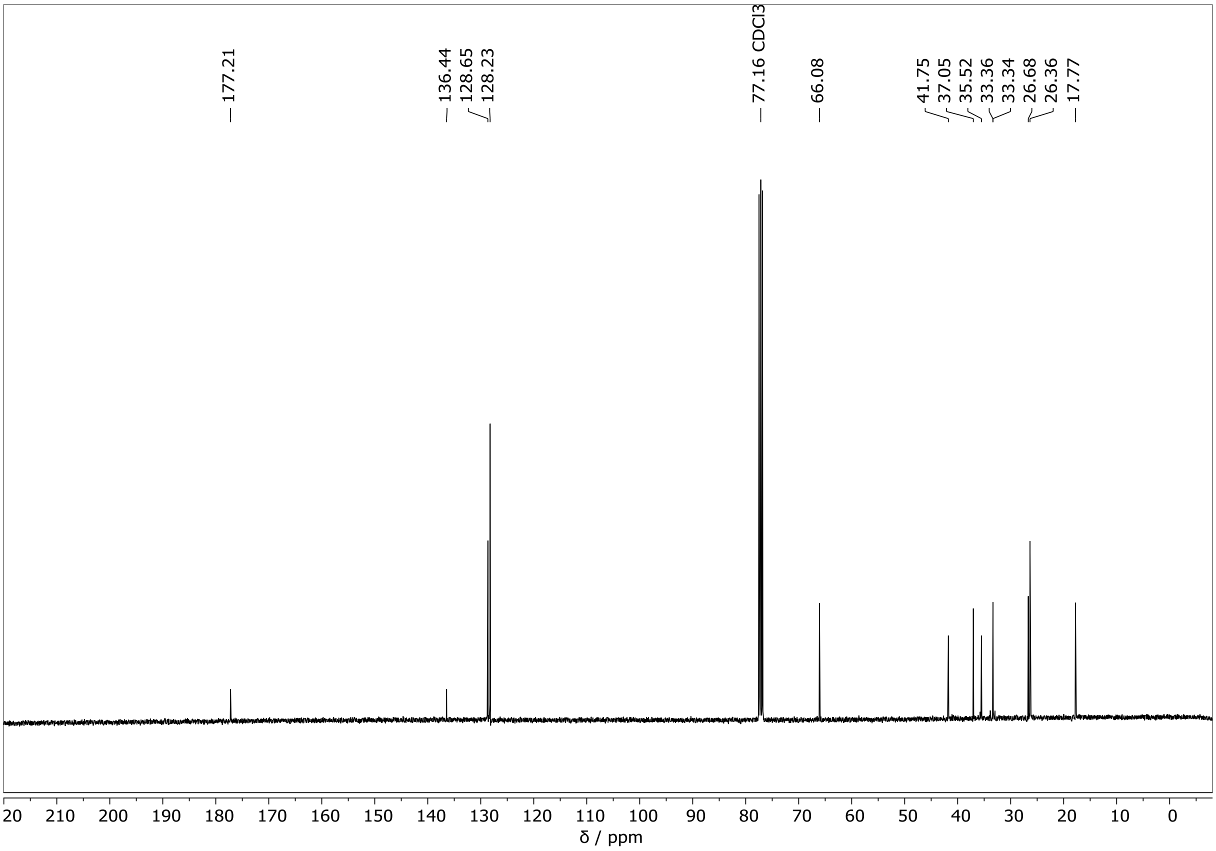

Diethyl 2-(1-cyclohexylethyl)malonate (3d)

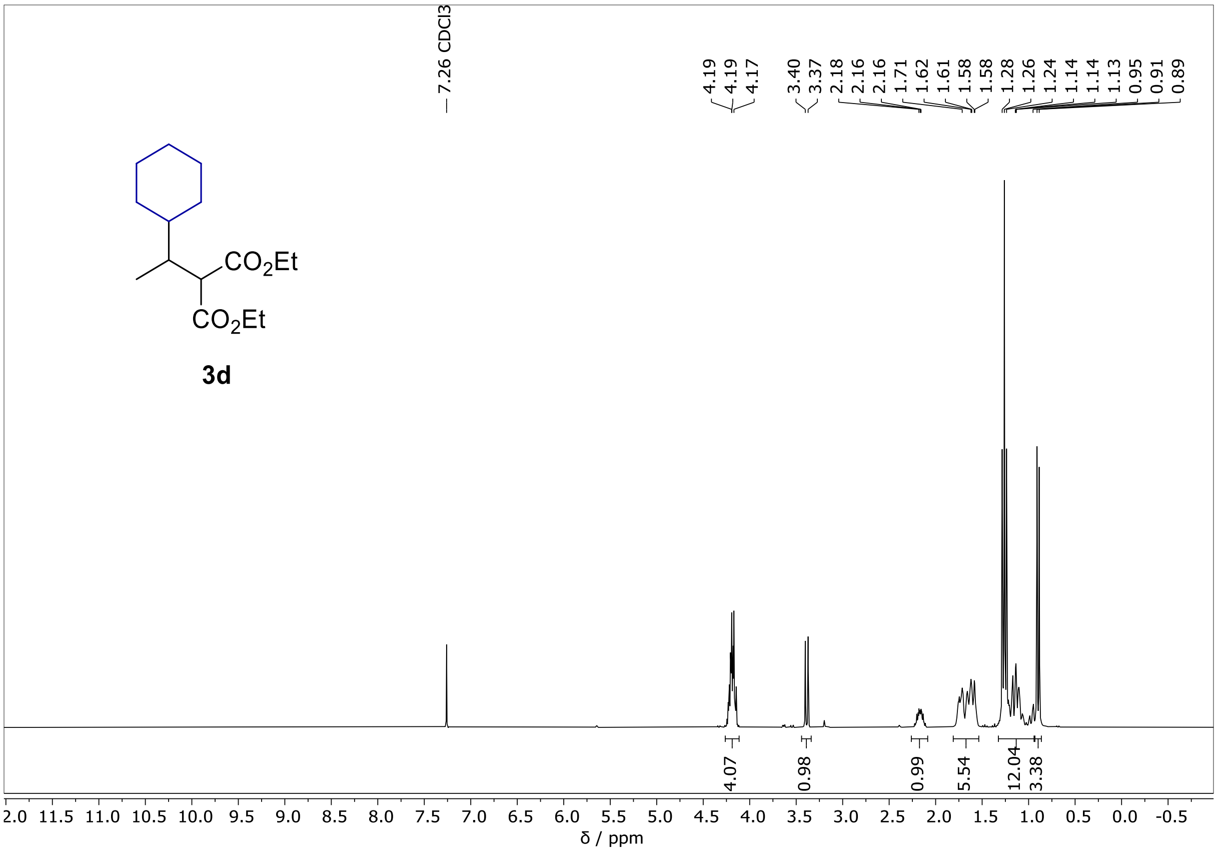

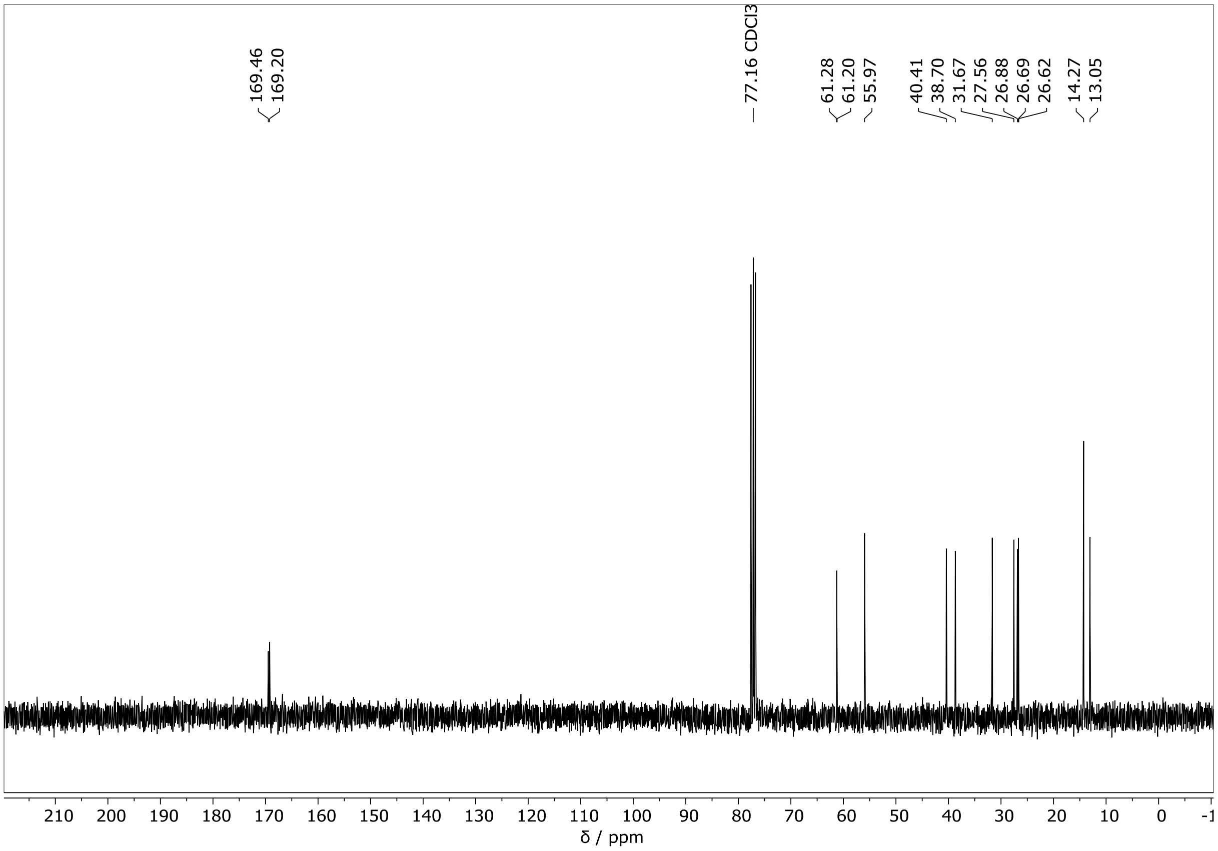

2-(Cyclohexyl(phenyl)methyl)malononitrile (3e)

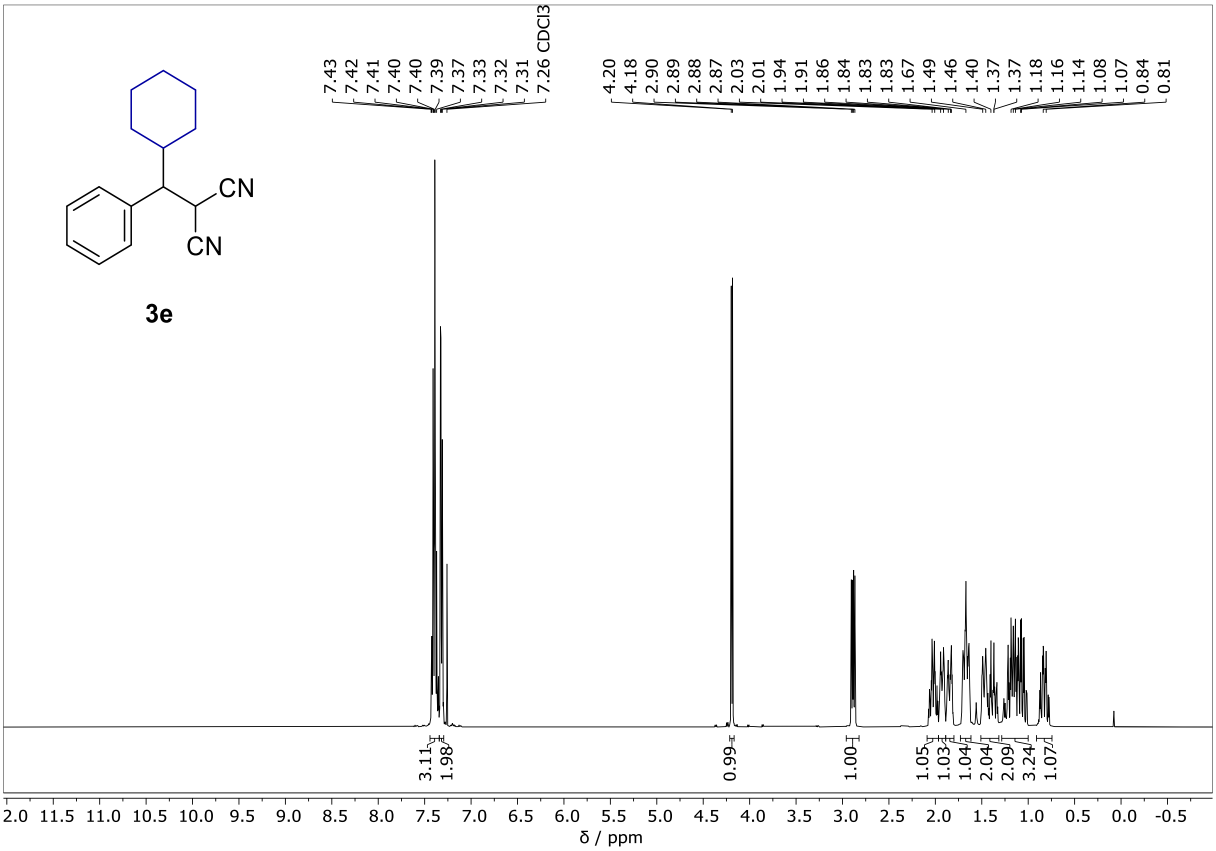

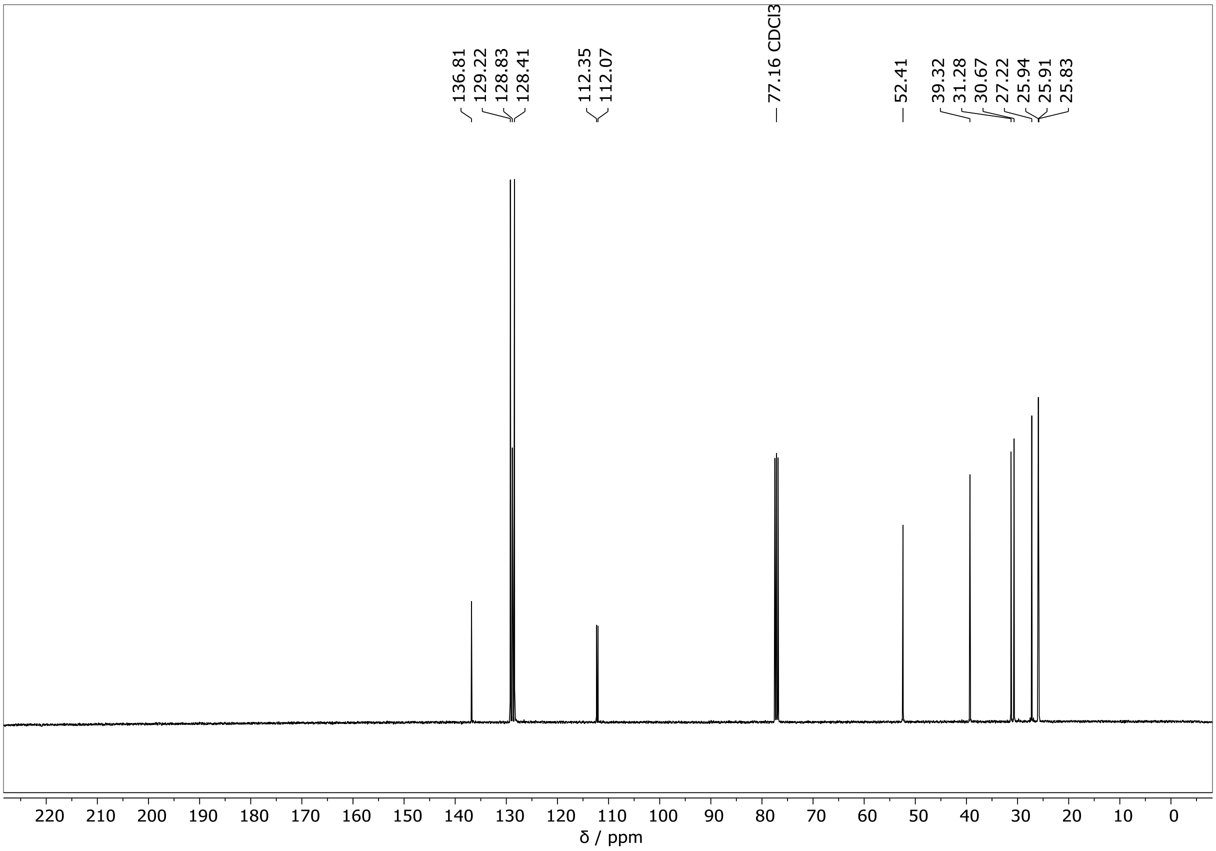

Dimethyl 2-benzylsuccinate (3f)

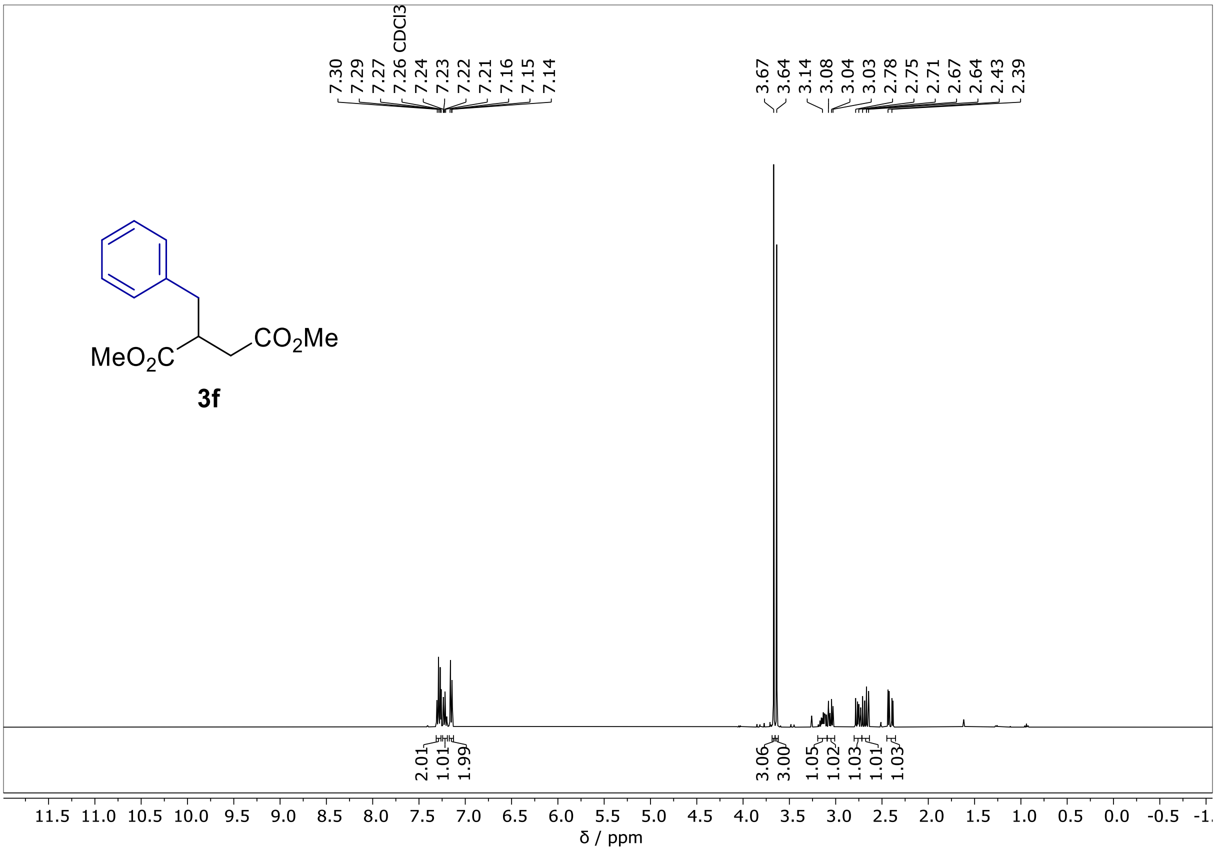

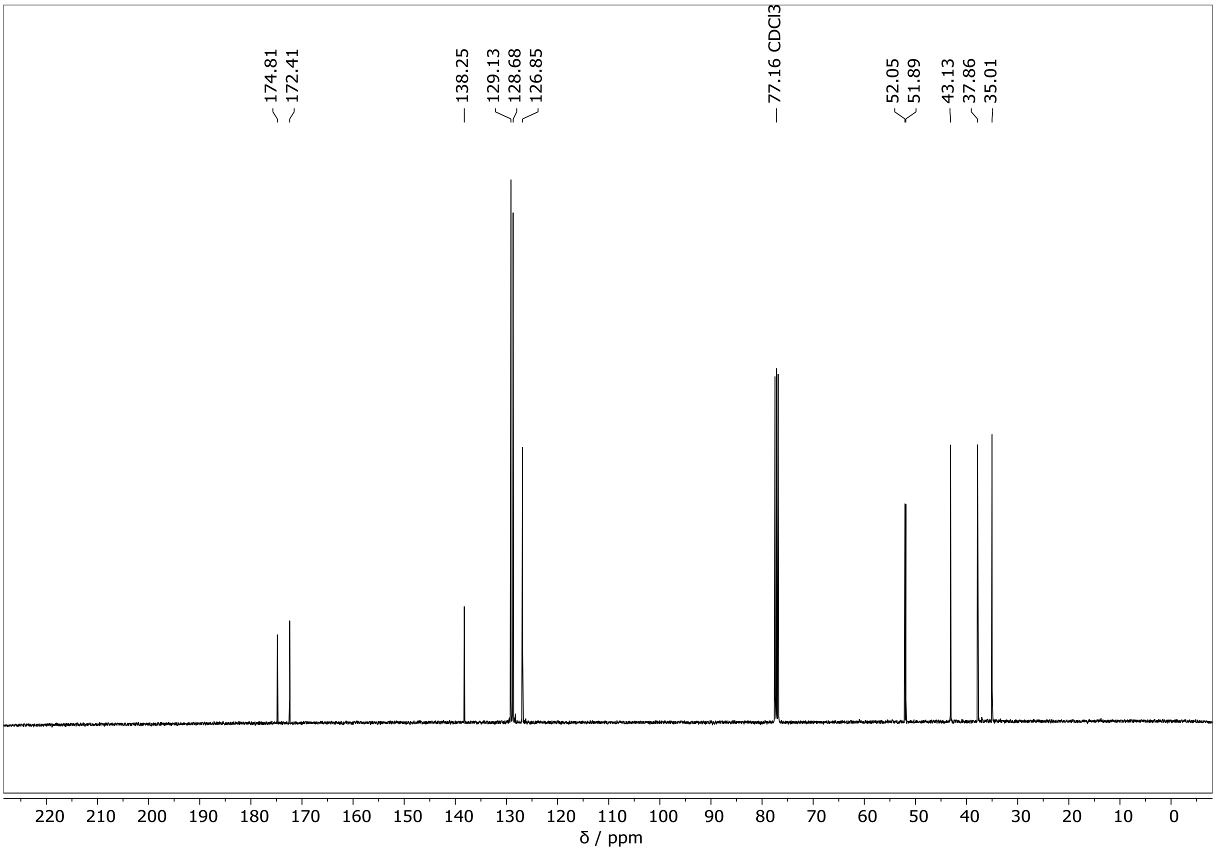

Dimethyl 2-cyclopentylsuccinate (3g)

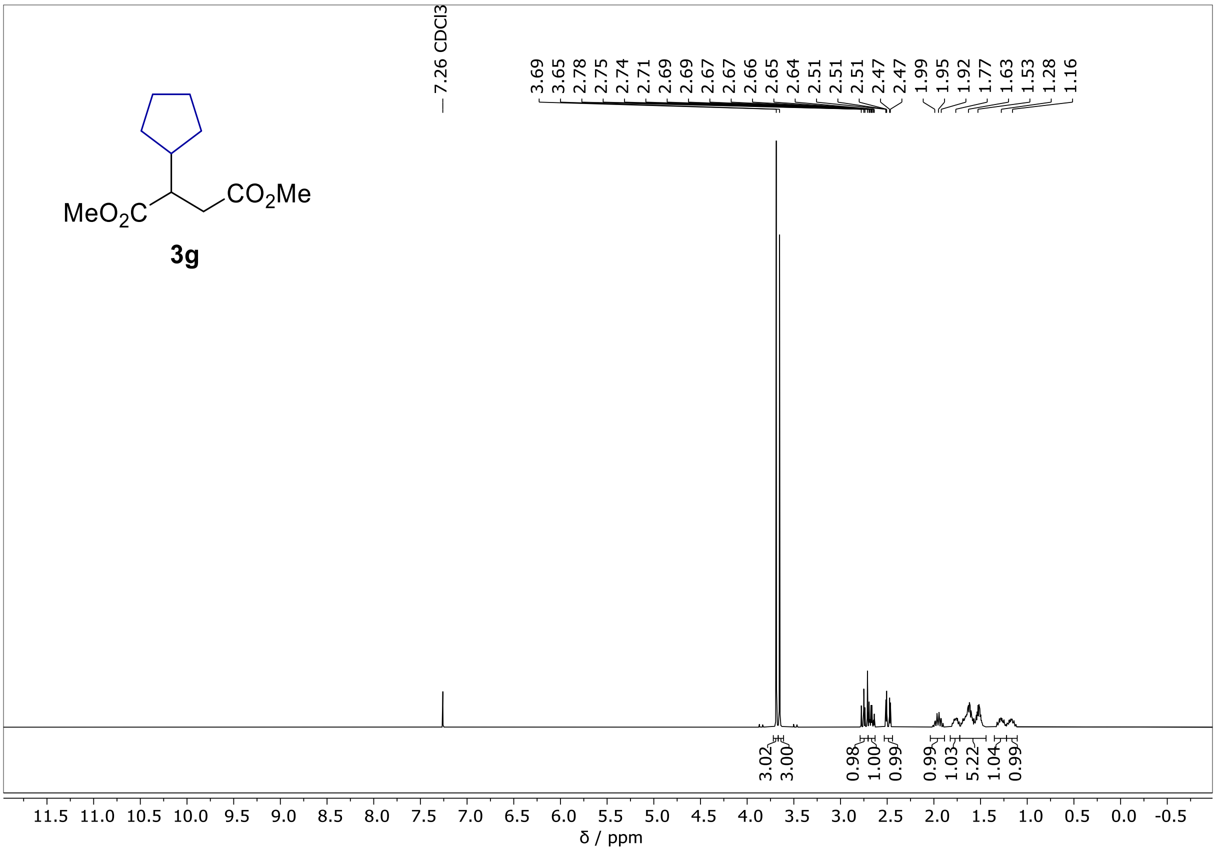

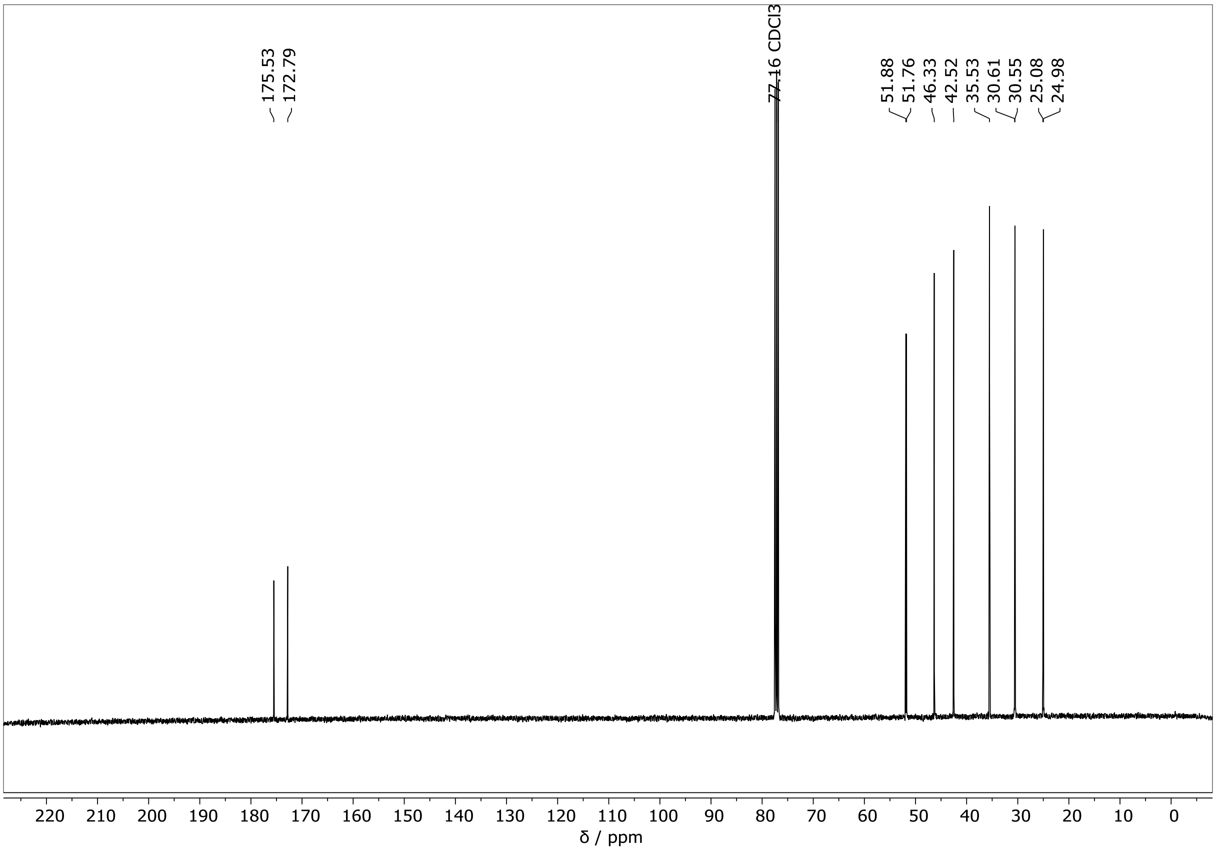

Alkylated haloarenes in the coupling with iodobenzenes

1-Cyclohexyl-4-methoxybenzene (5a)

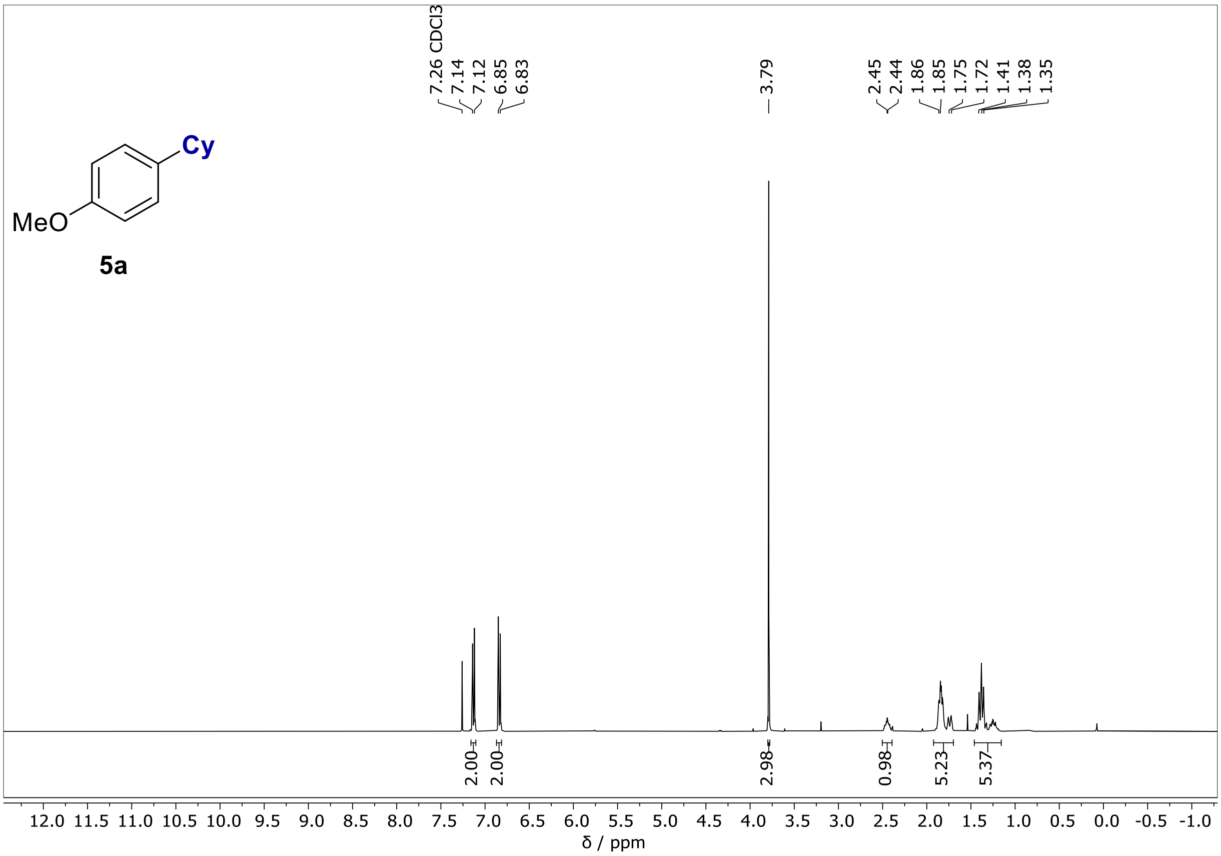

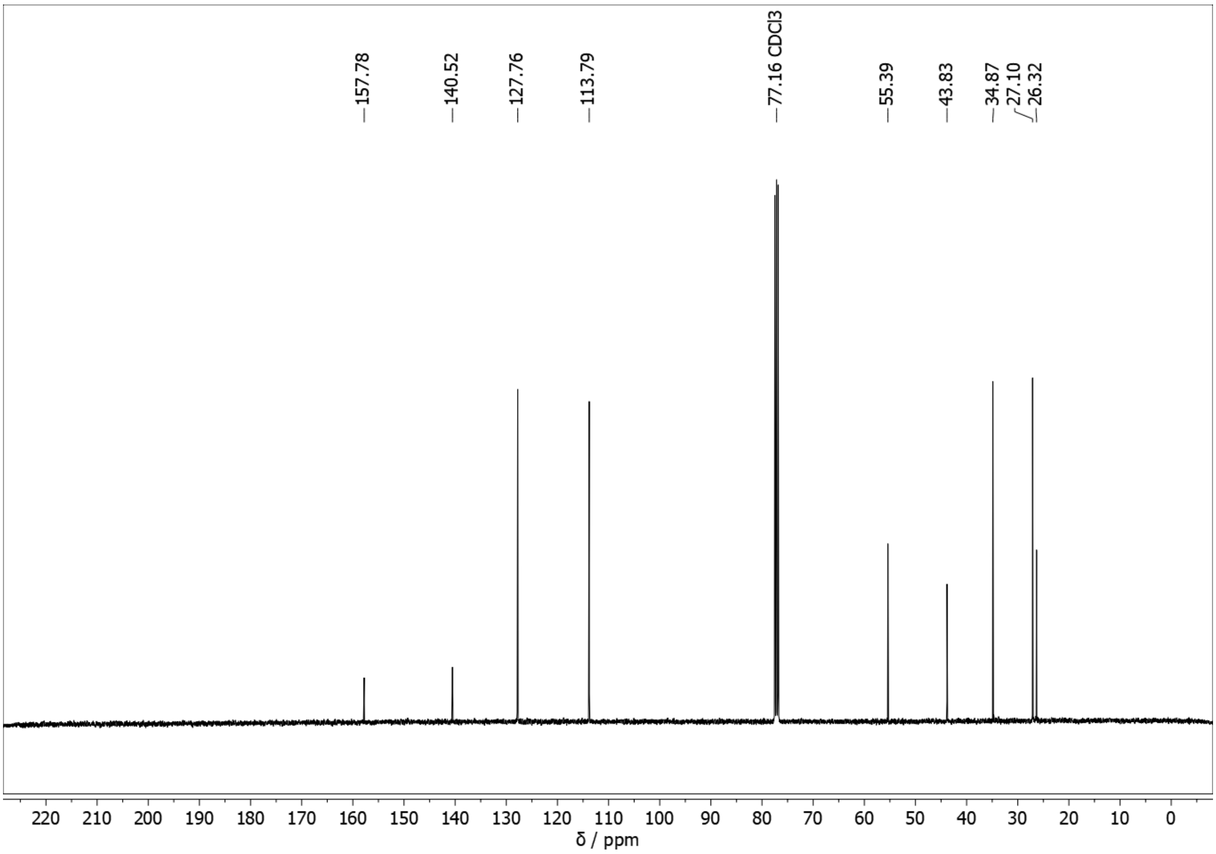

1-Cyclohexyl-4-(trifluoromethyl)benzene (5b)

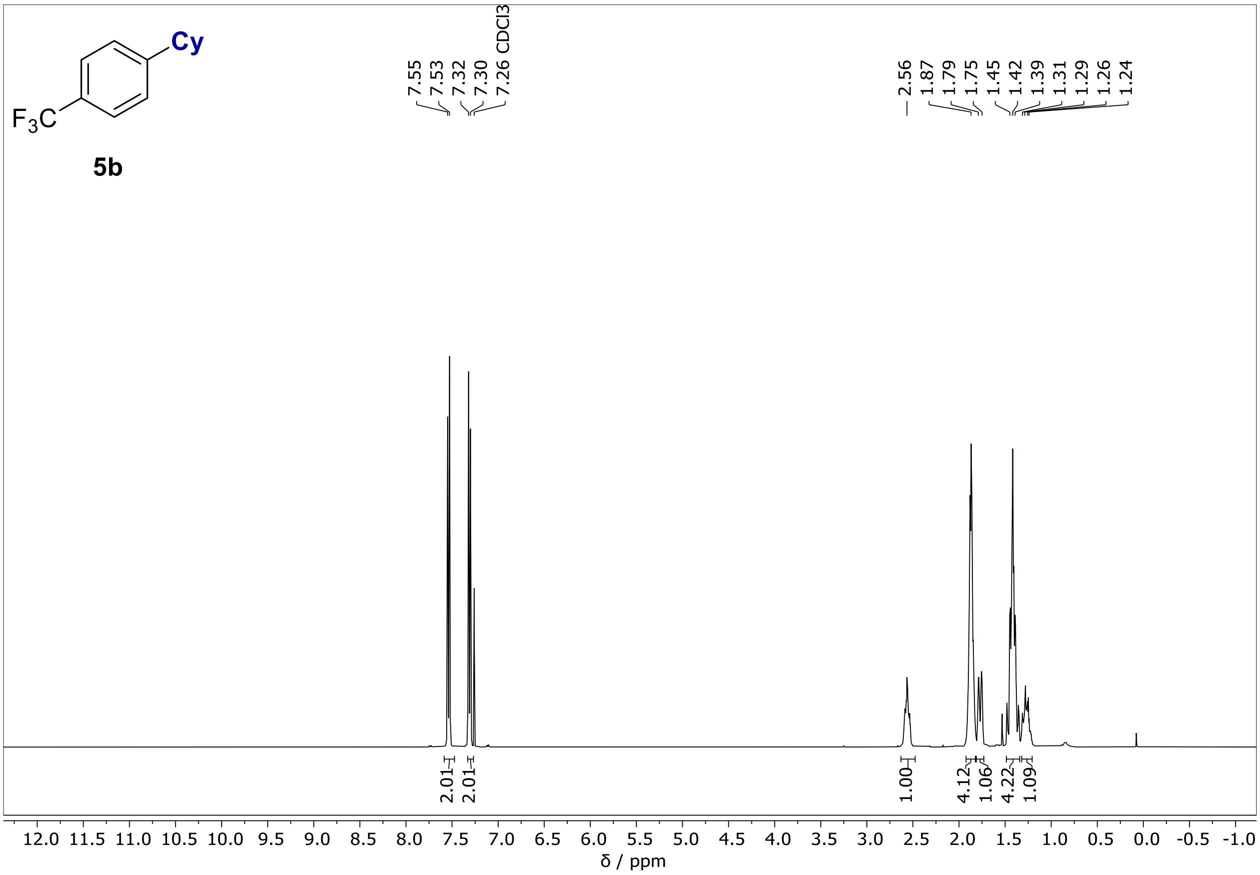

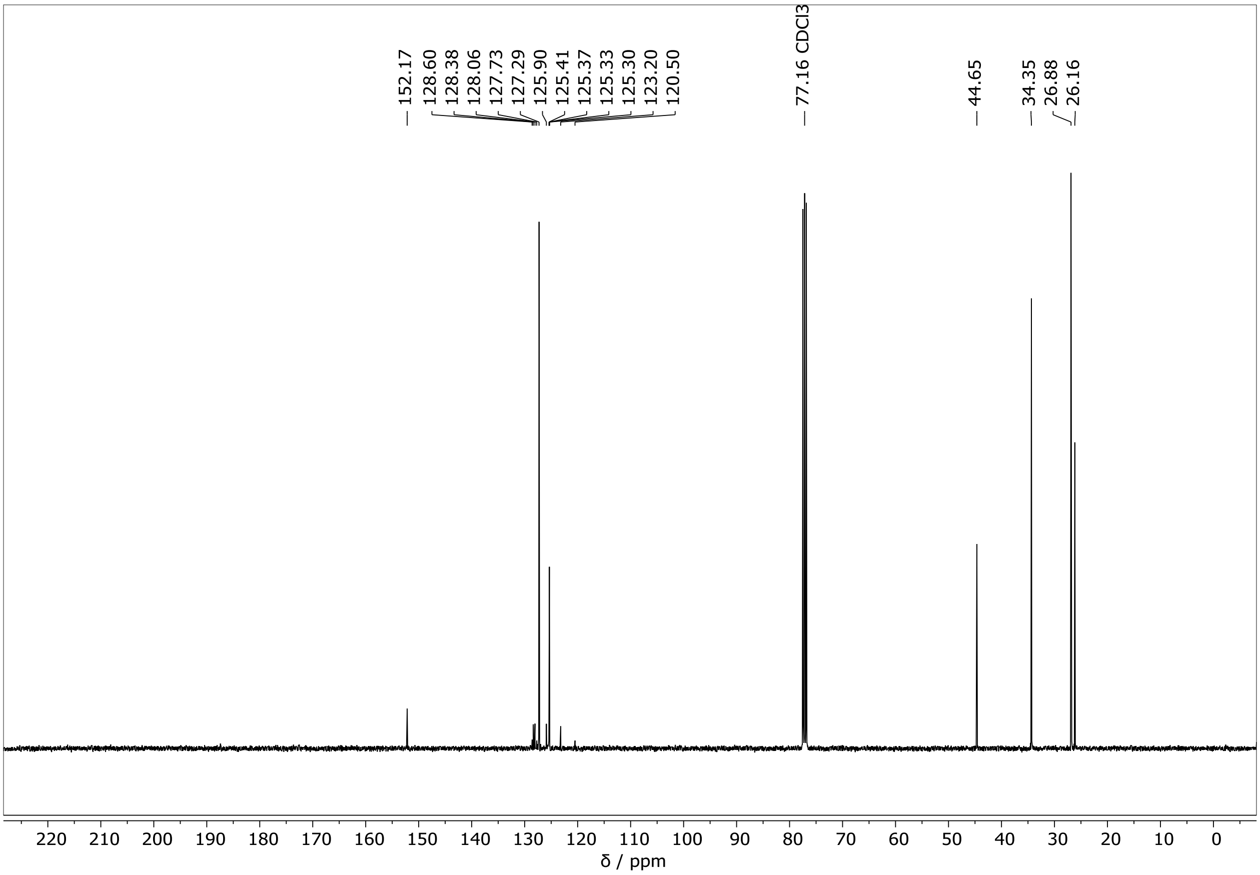

1-Cyclohexyl-4-methylbenzene (5c)

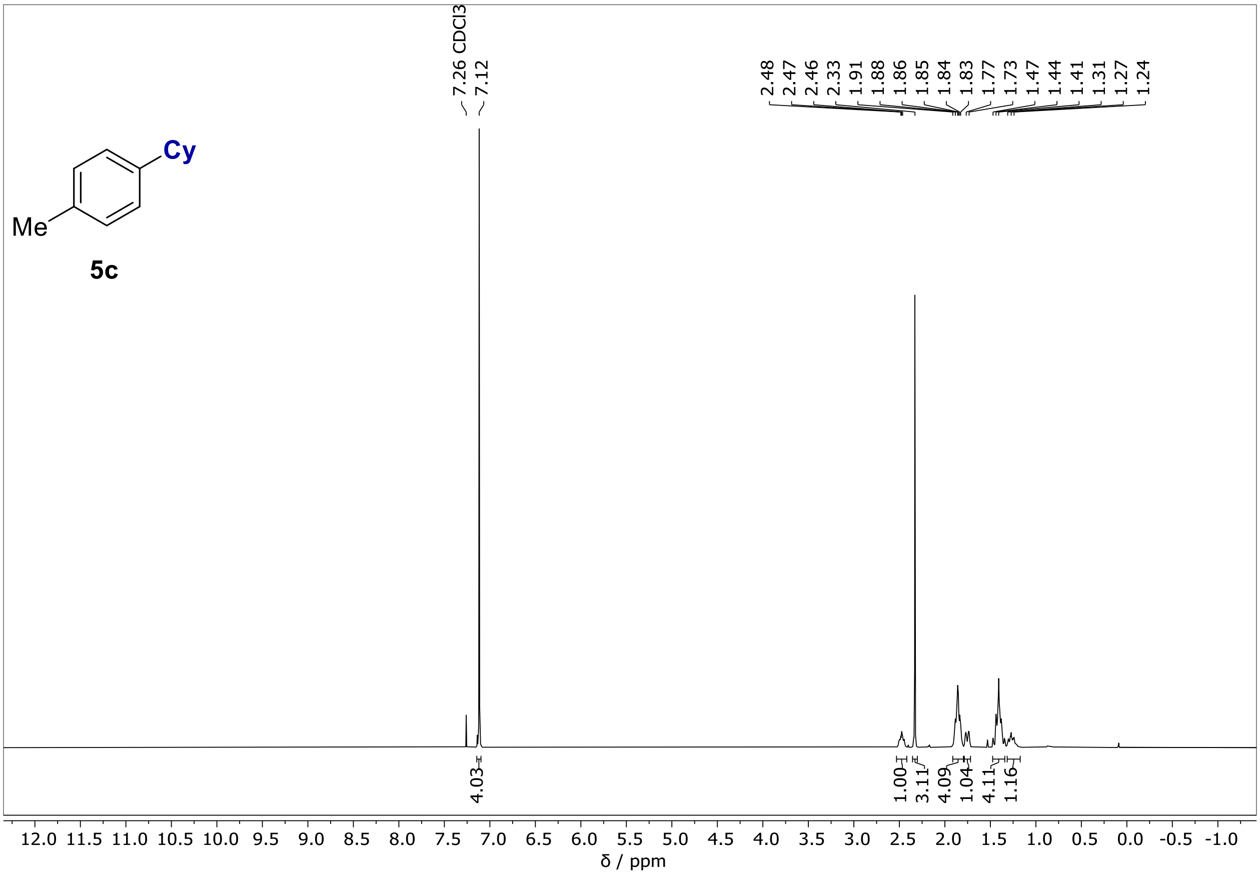

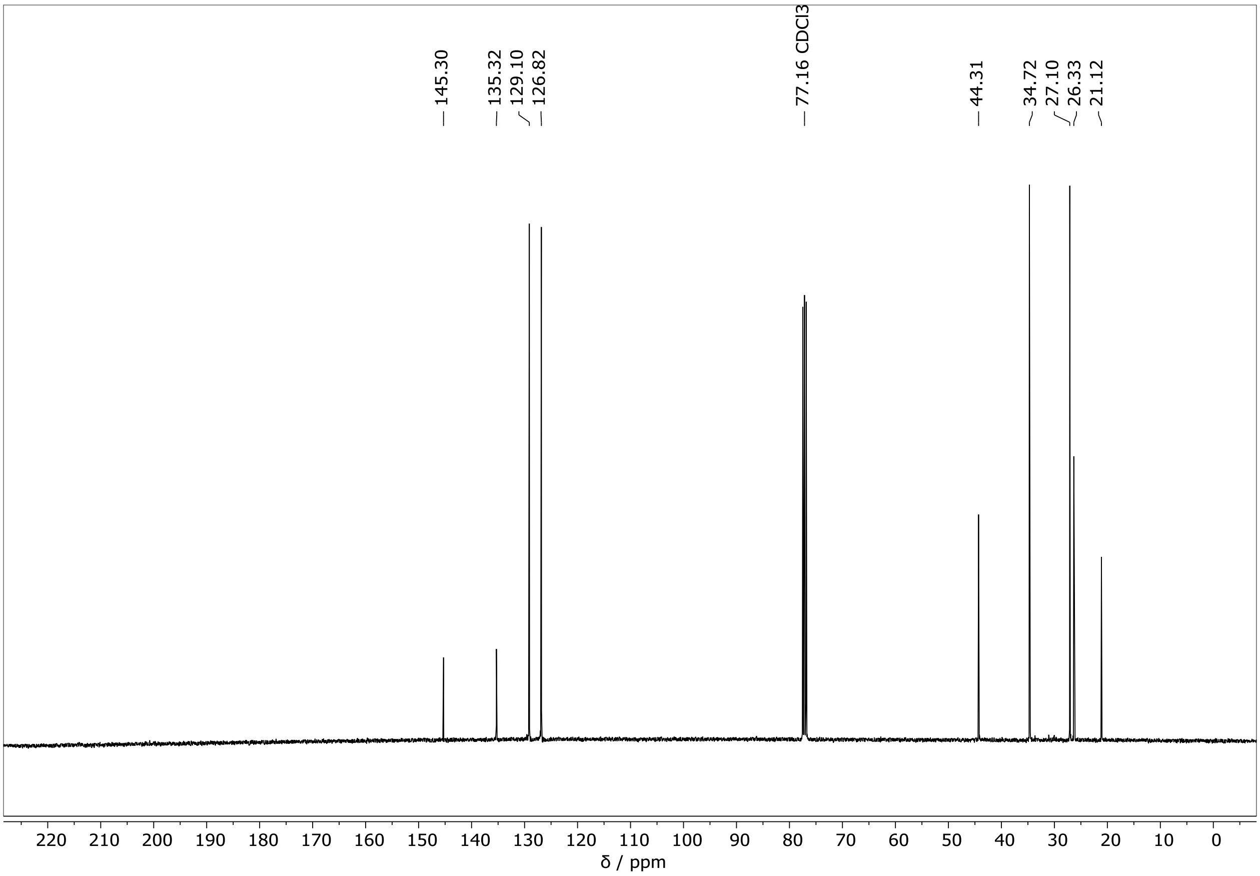

4-Cyclohexyl-1,2-dimethoxybenzene (5d)

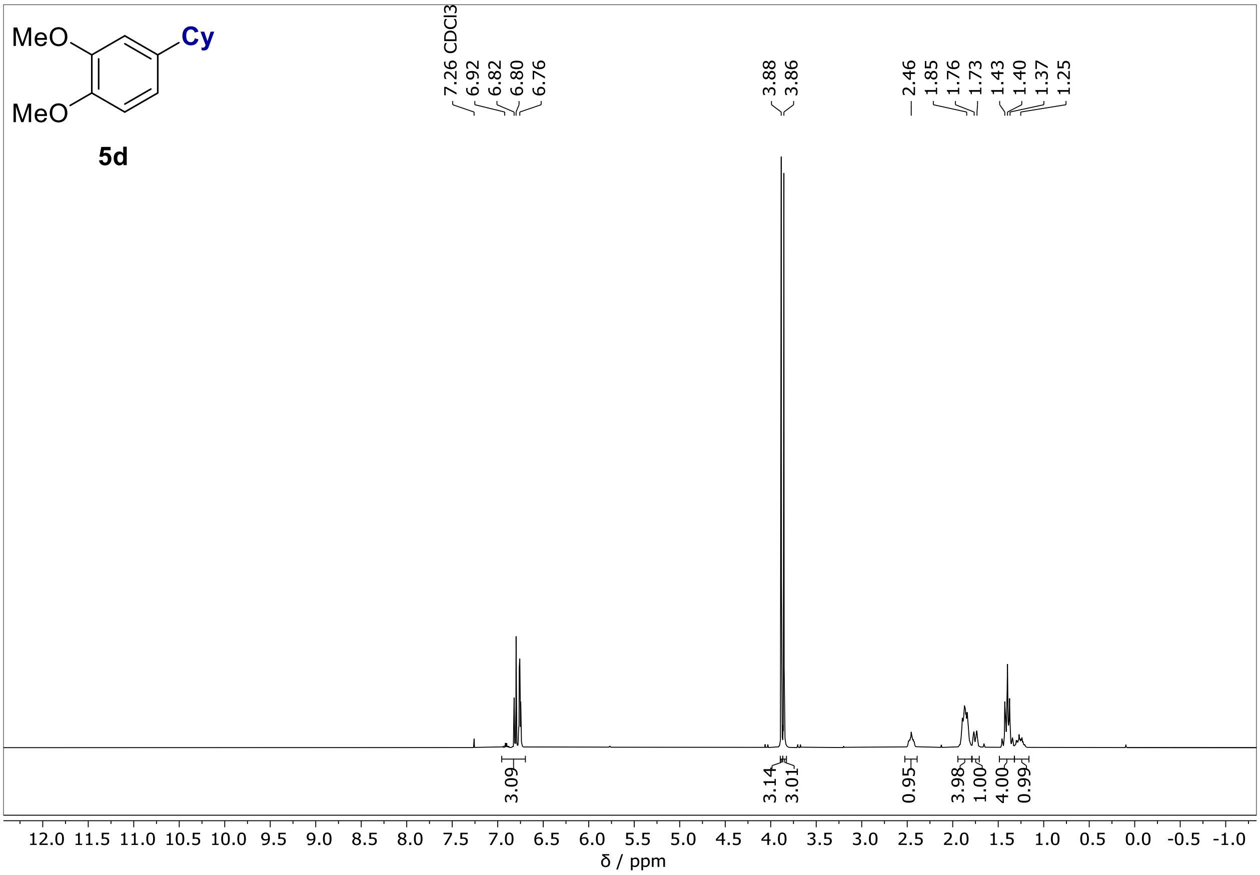

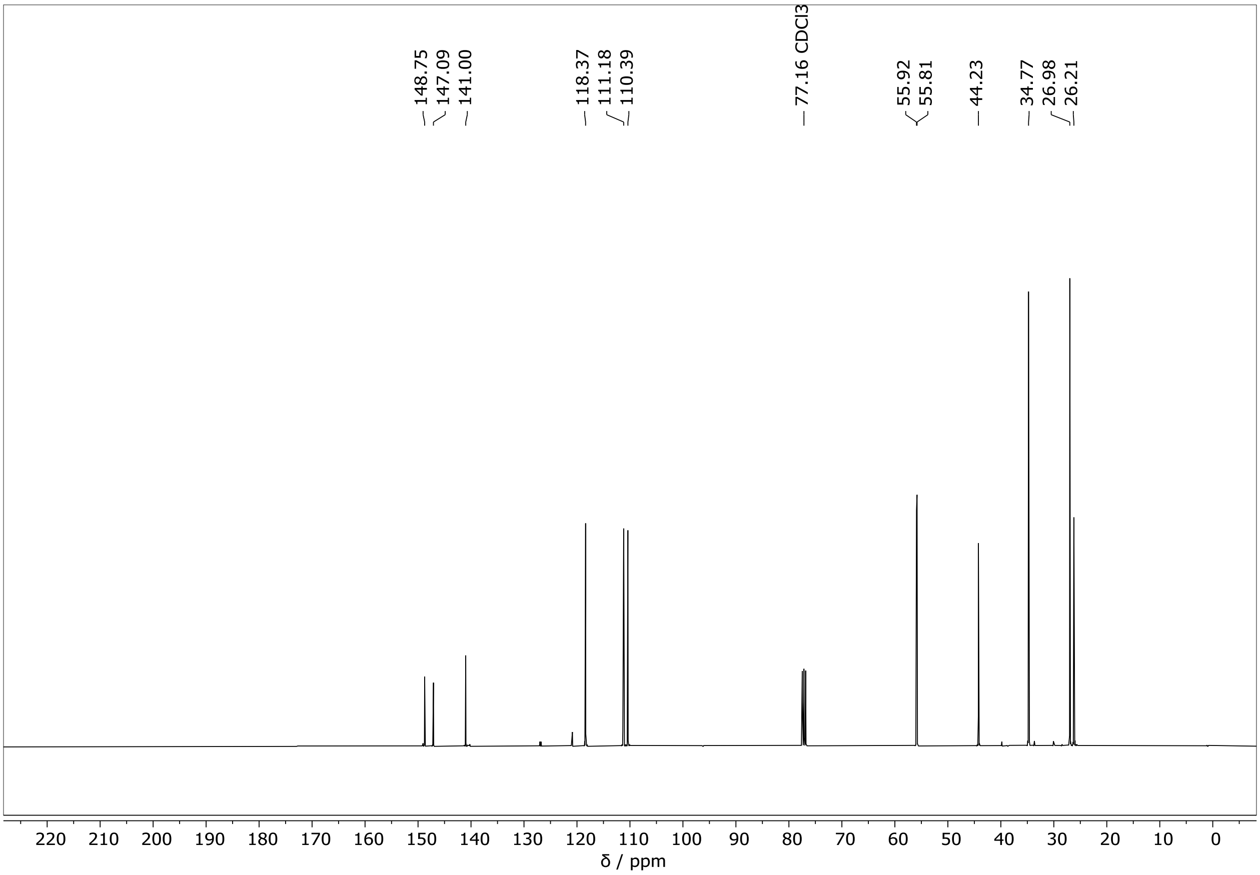

1-Cyclohexyl-3-methoxybenzene (5e)

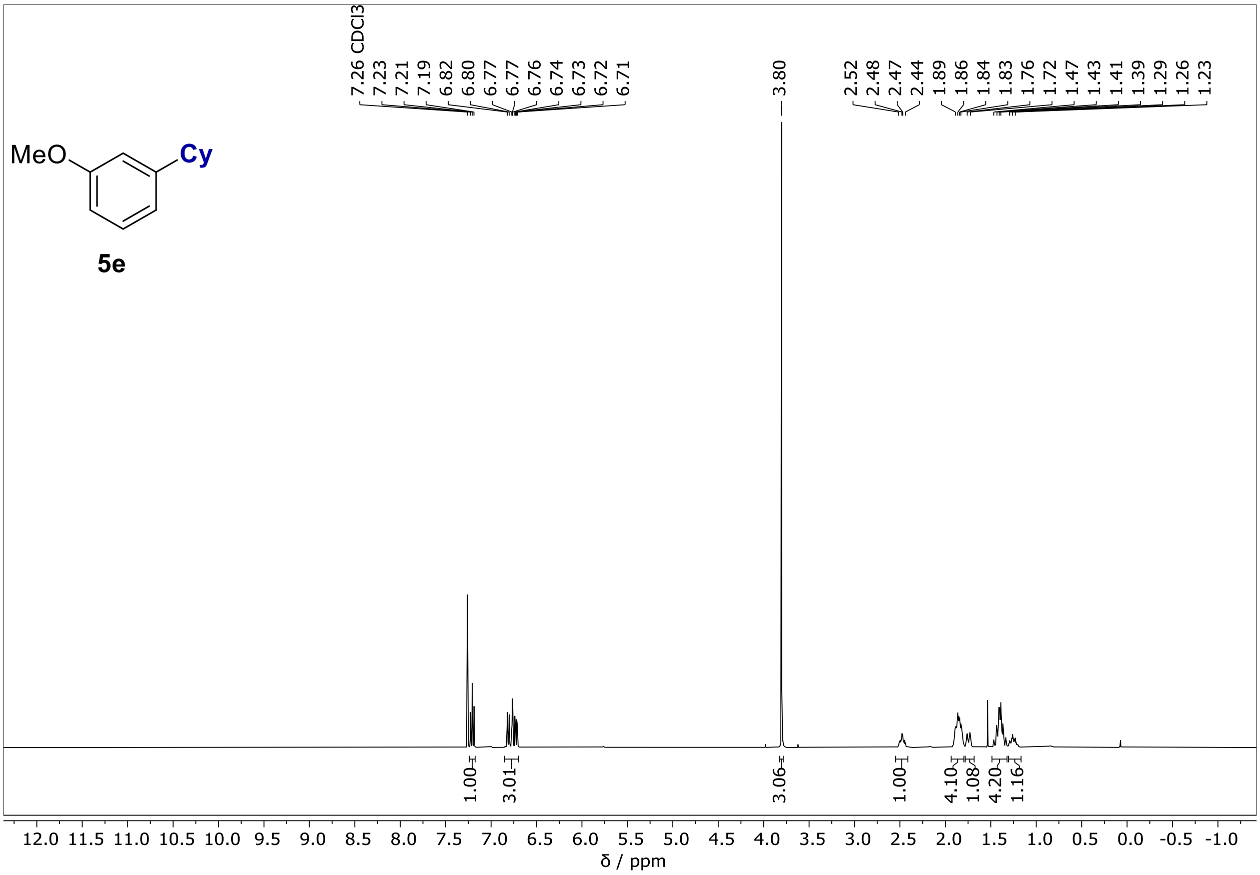

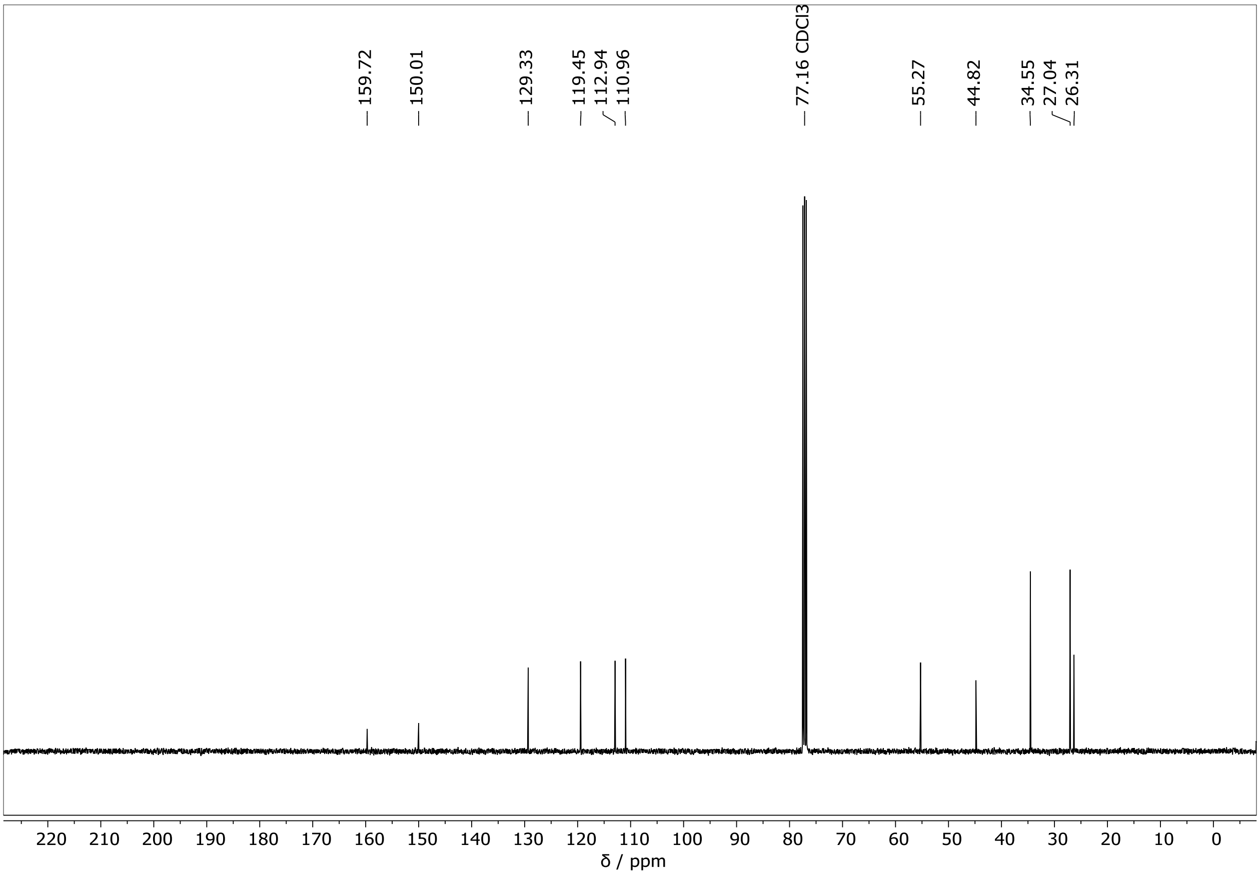

1-Cyclohexyl-4-fluorobenzene (5f)

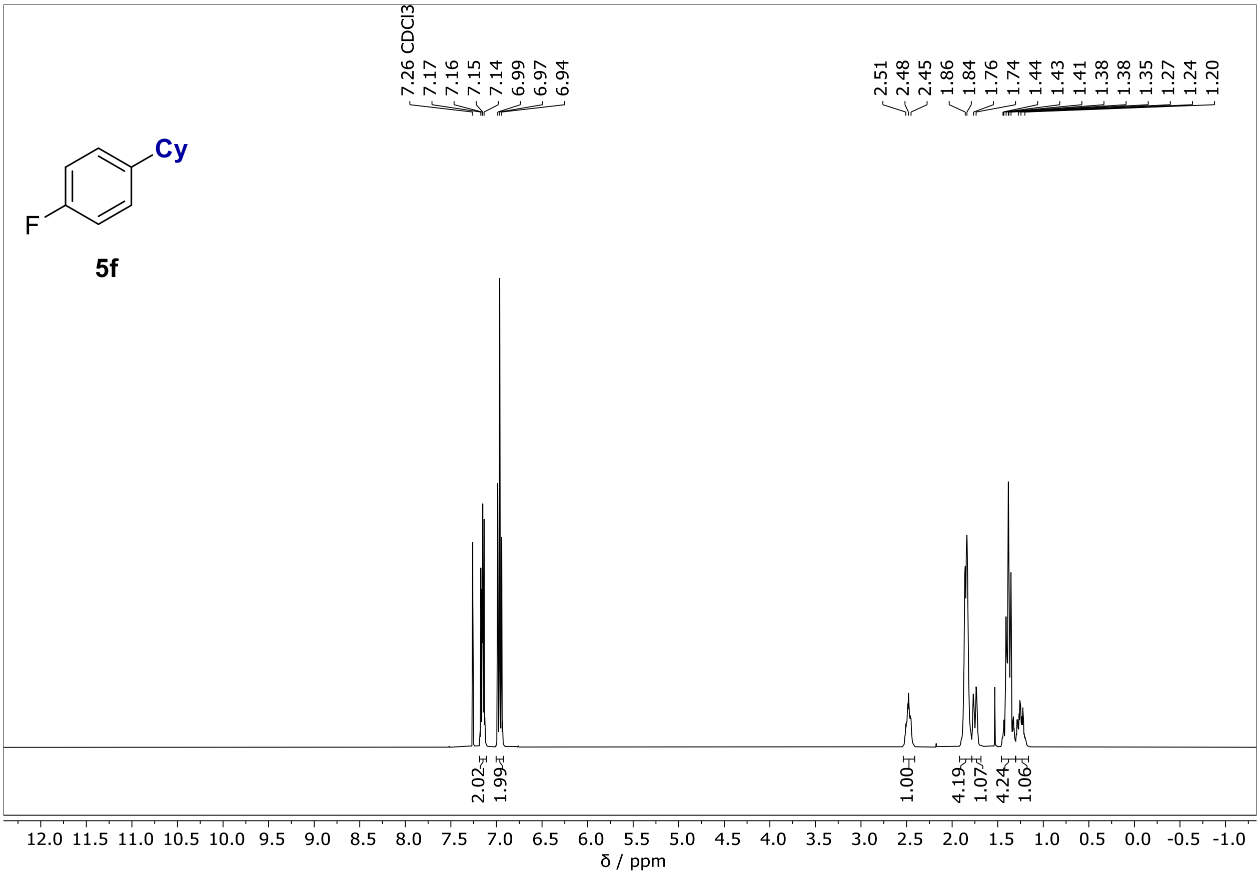

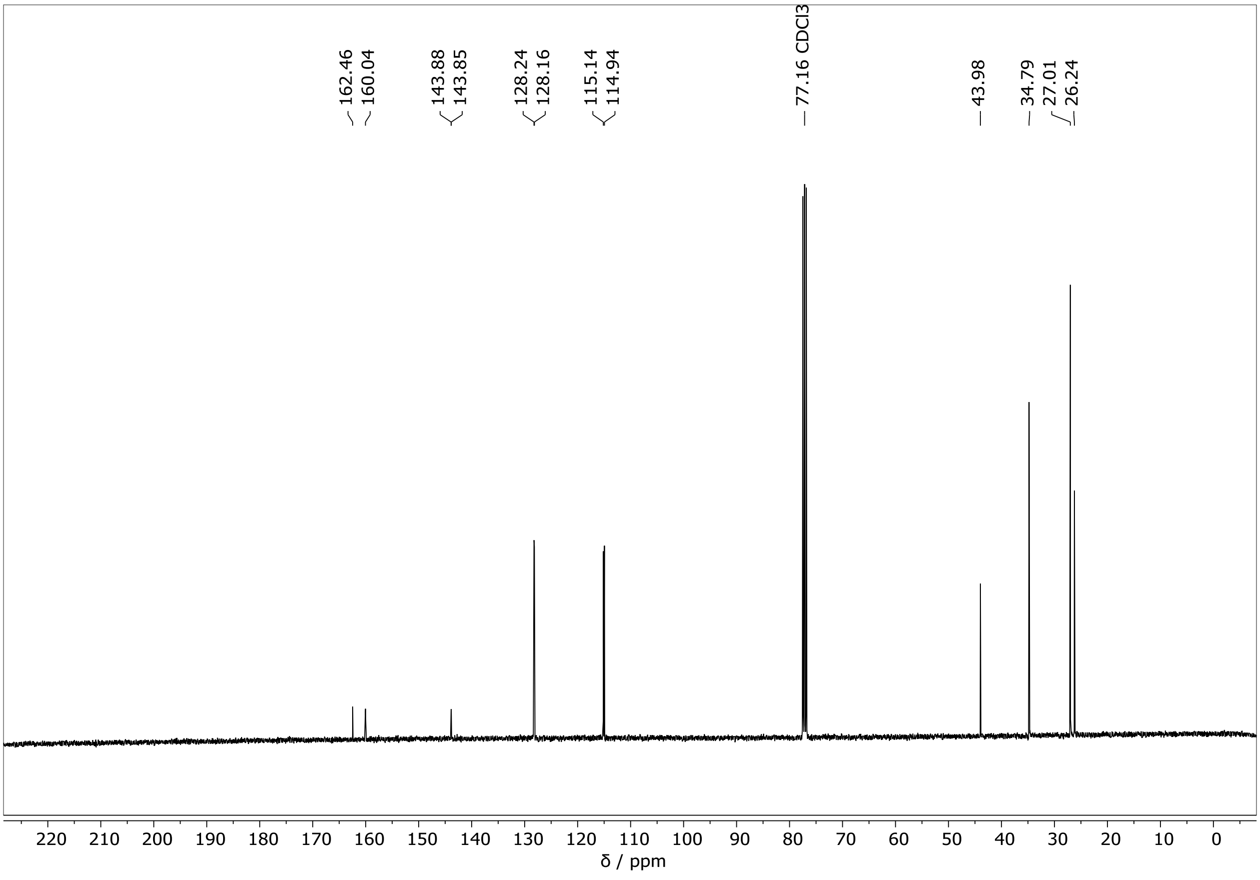

4-Cyclohexylphenol (5g)

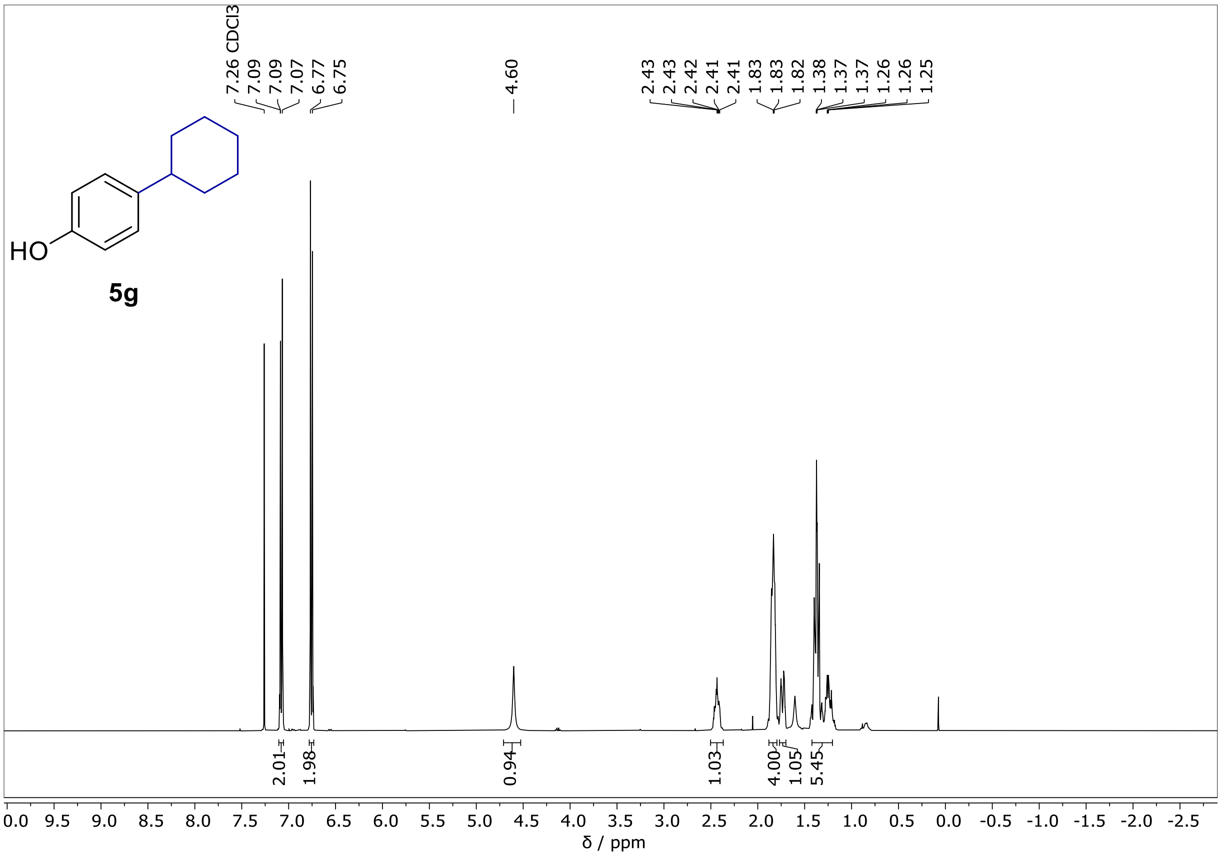

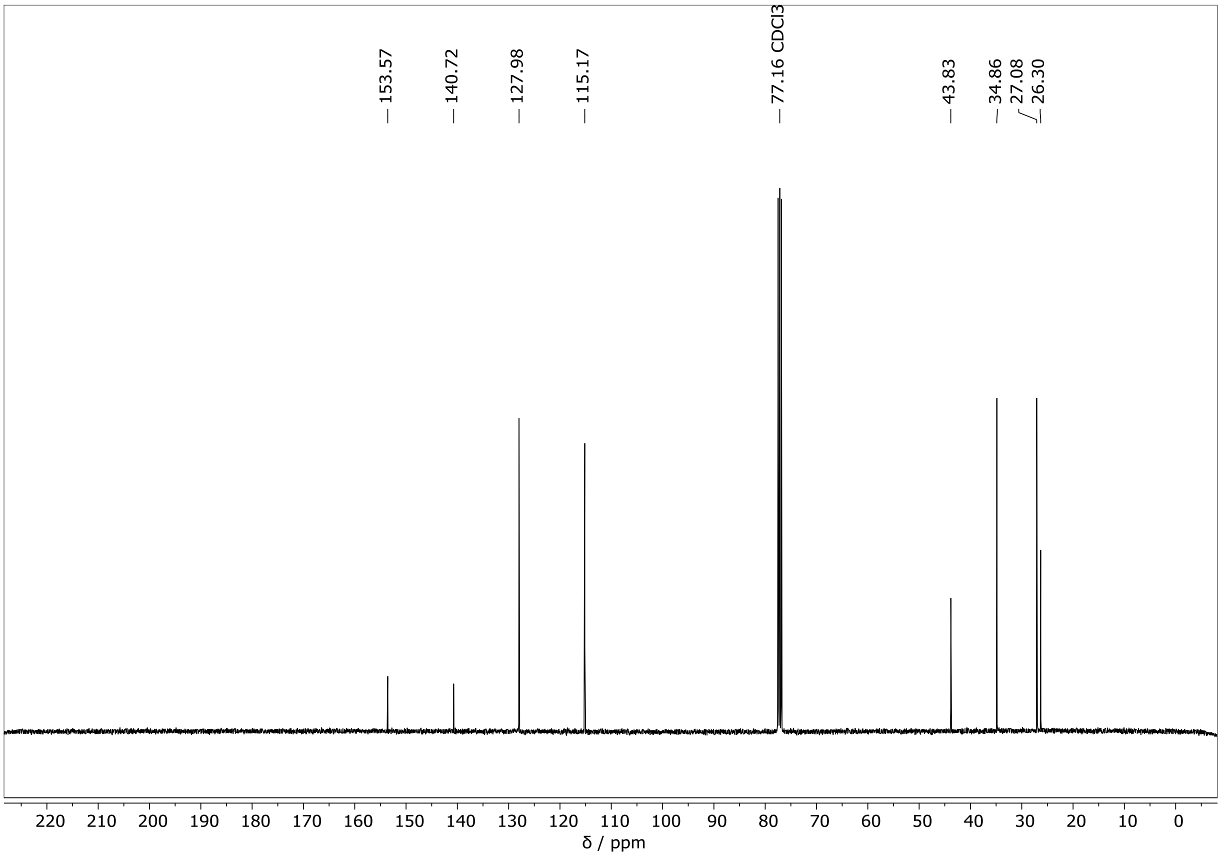

Alkylated haloarenes in the coupling with bromobenzenes

1-Cyclohexyl-3-(trifluoromethyl)benzene (5h)

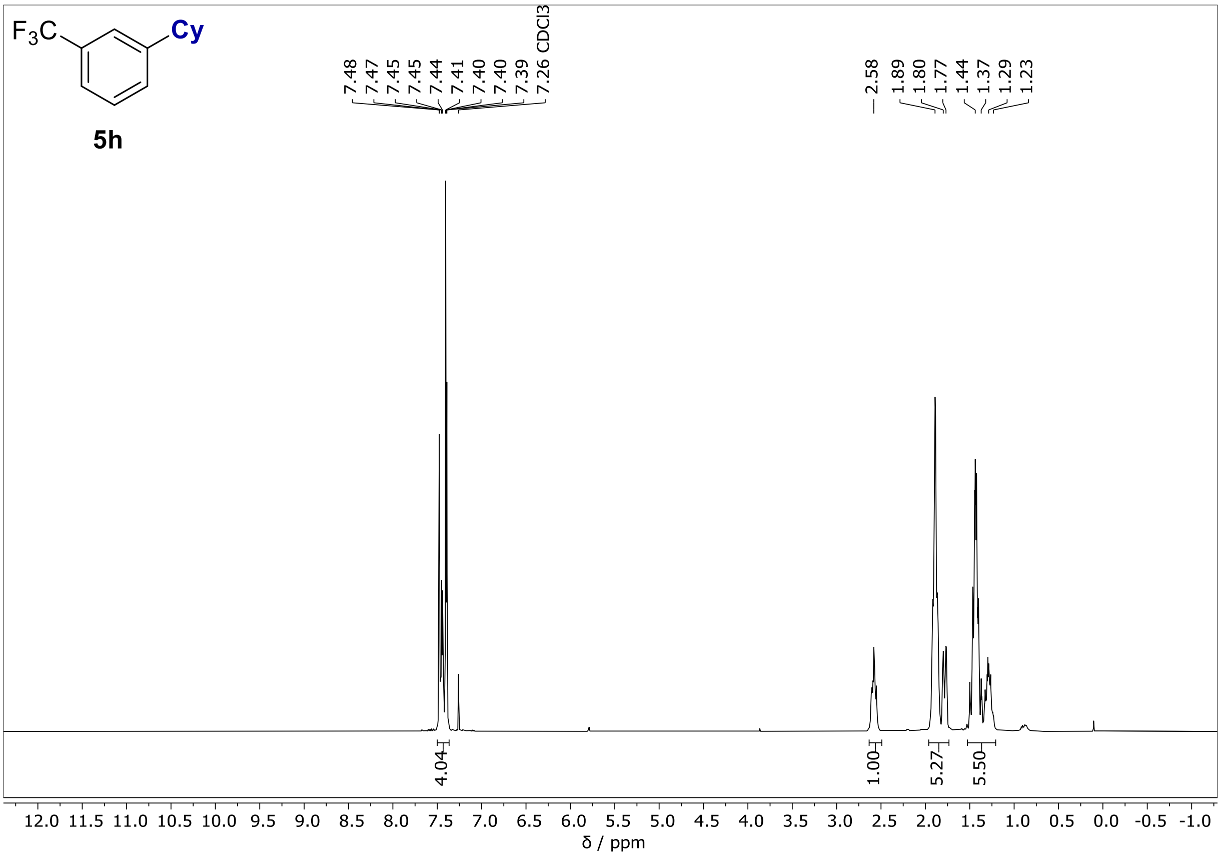

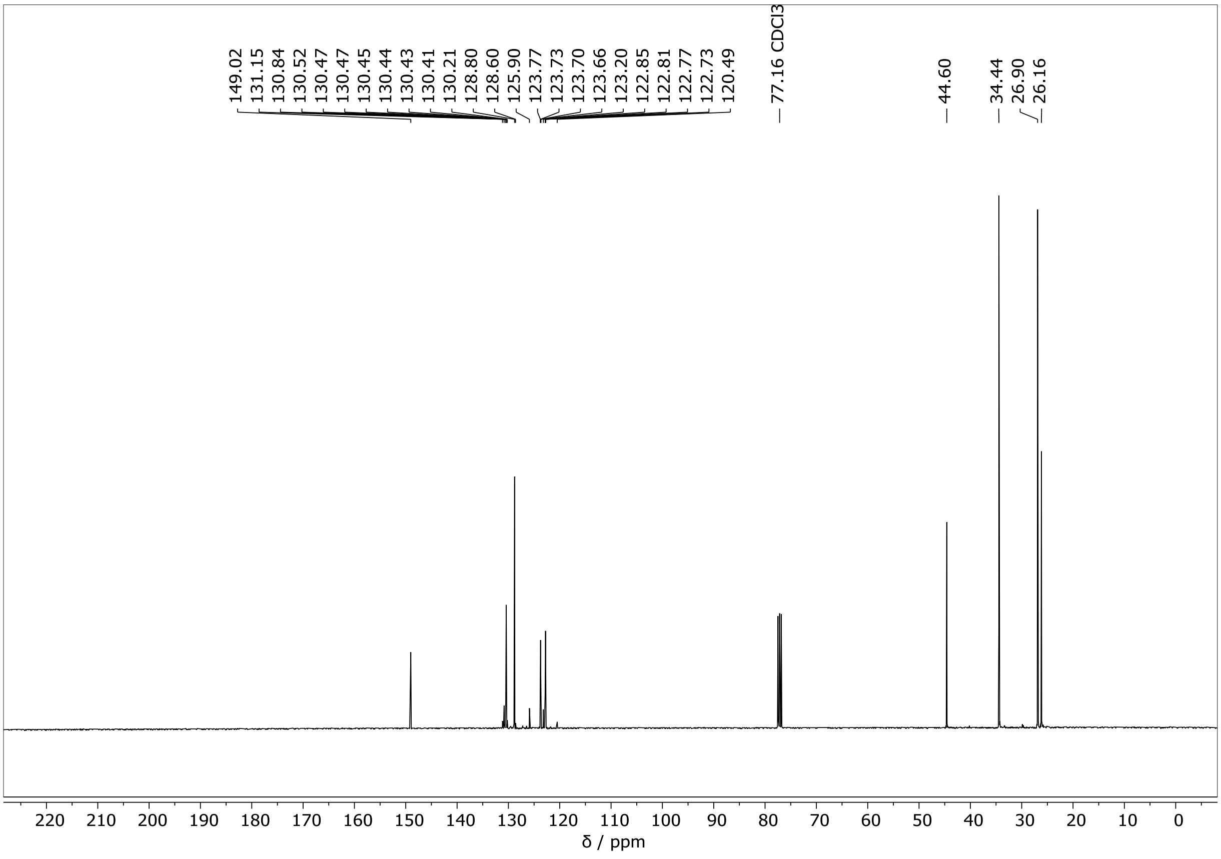

1-Cyclohexyl-3-fluorobenzene (5i)

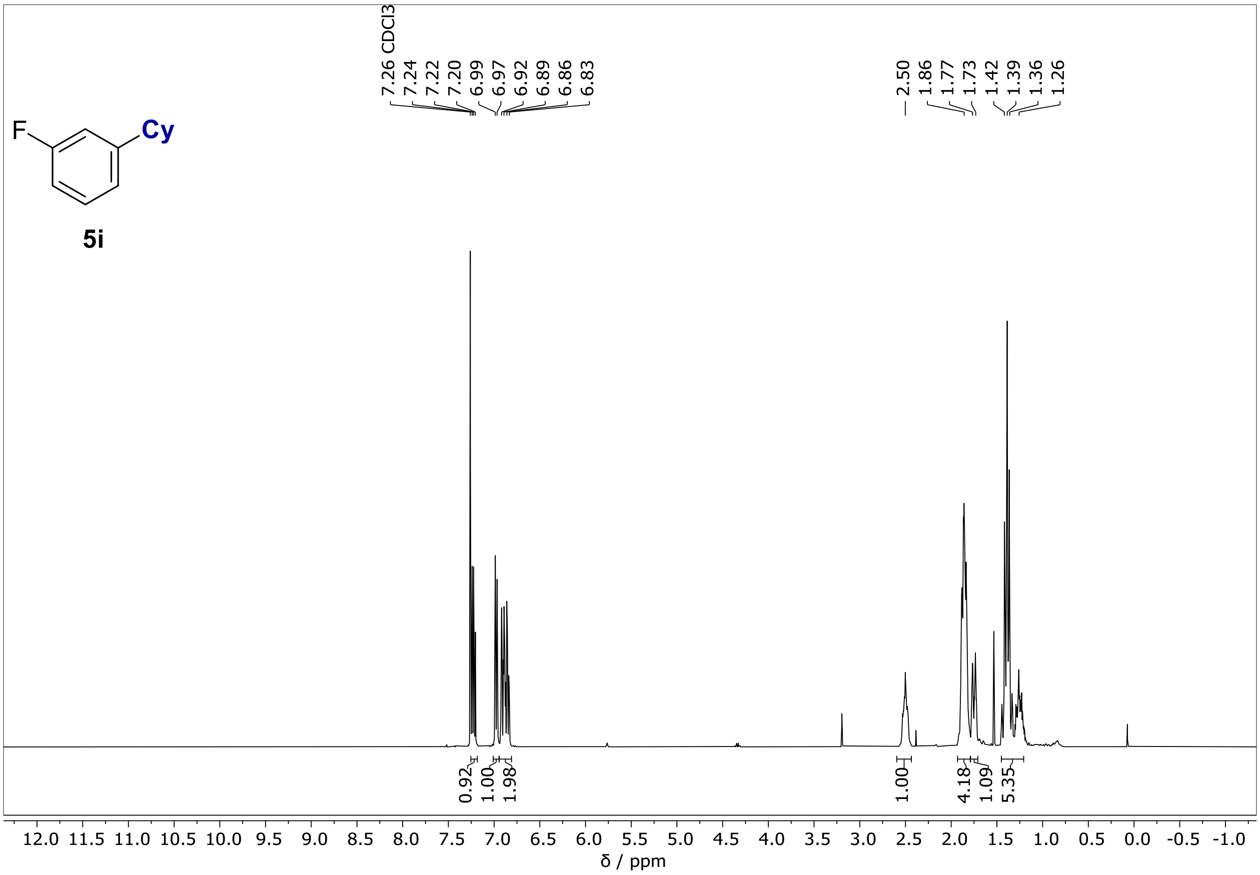

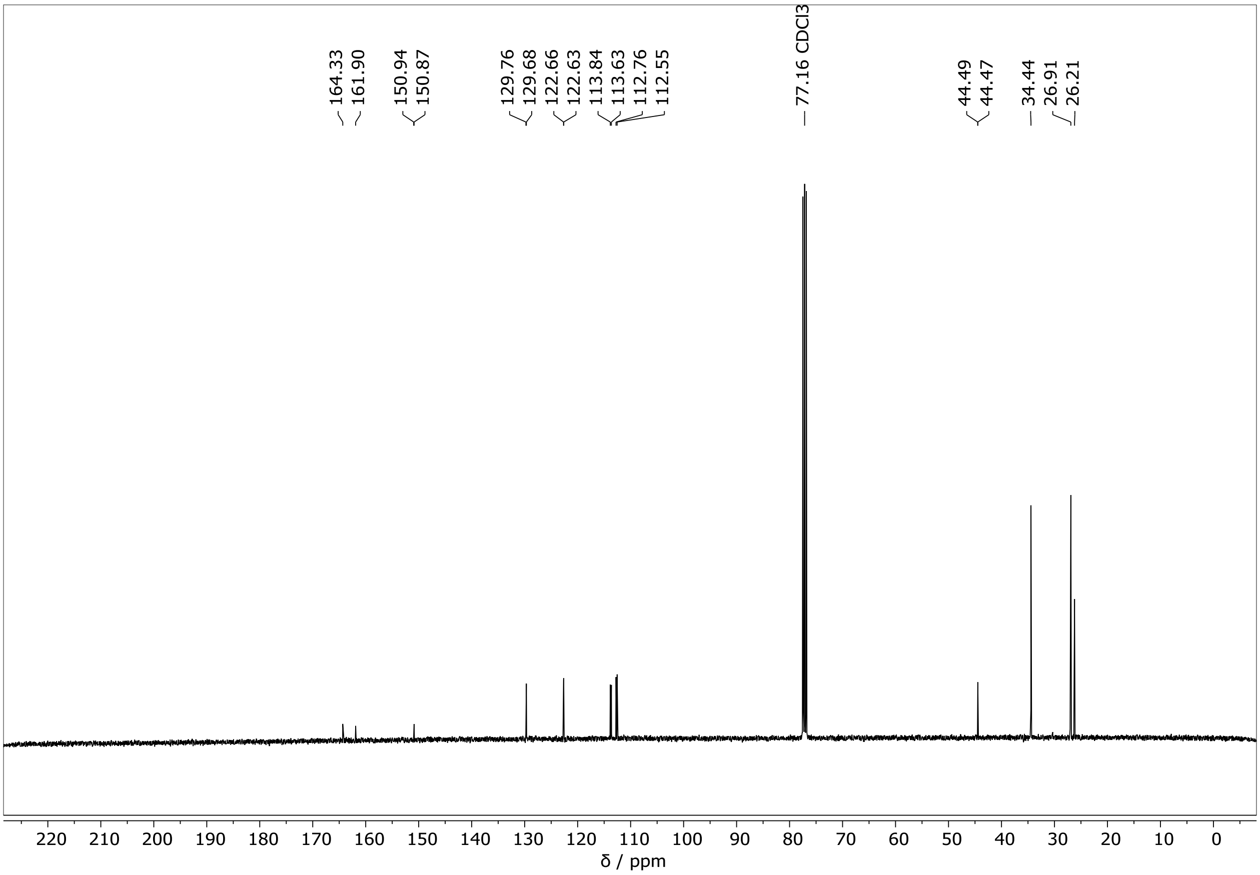

3-Cyclohexylquinoline (5j)

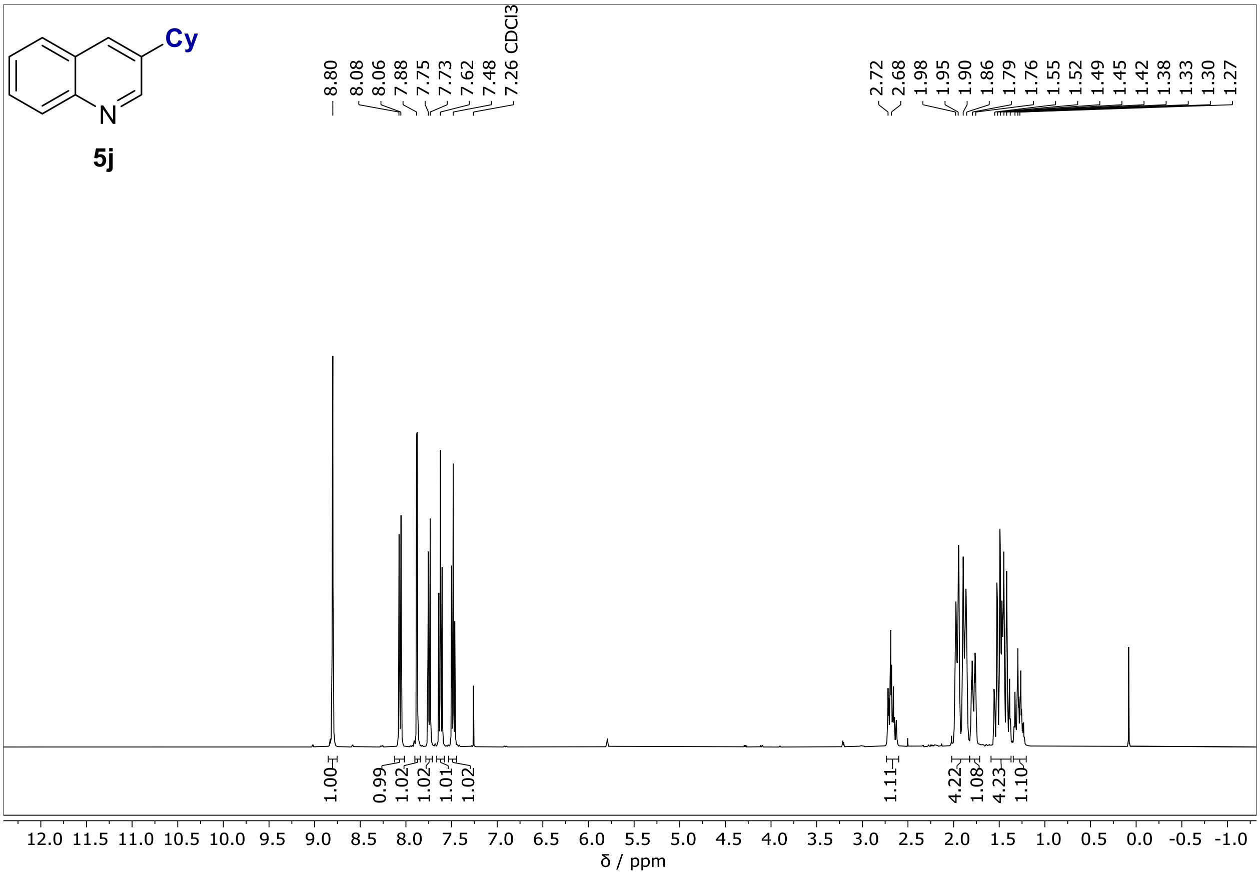

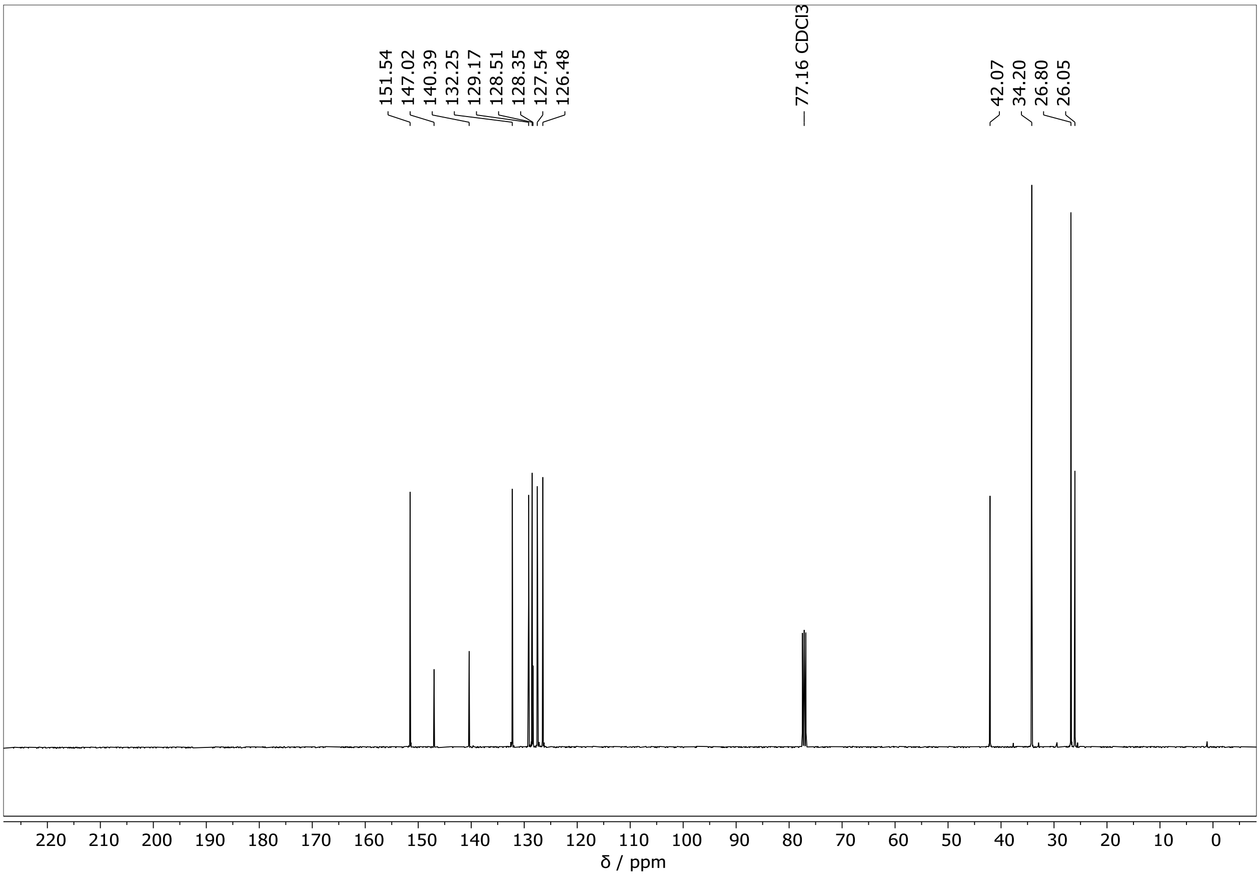

Methyl 4-cyclohexylbenzoate (5k)

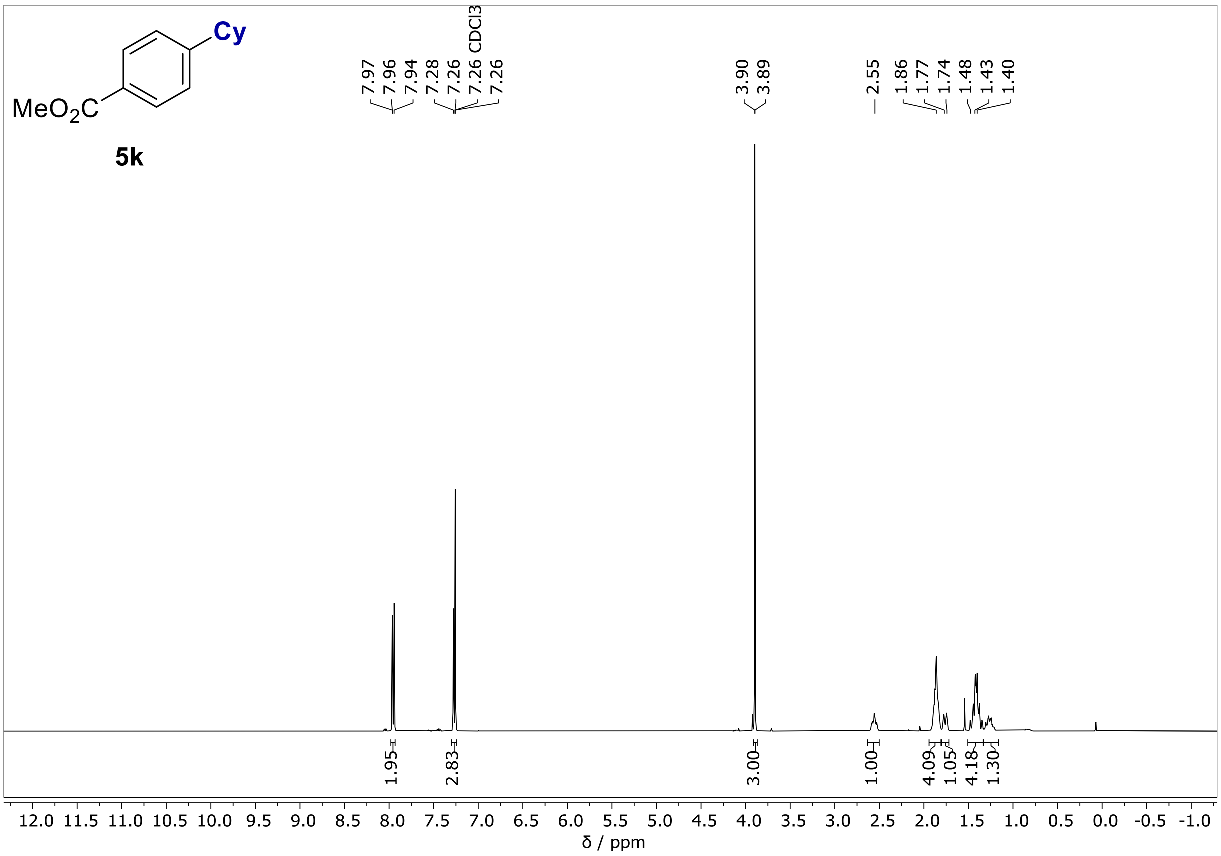

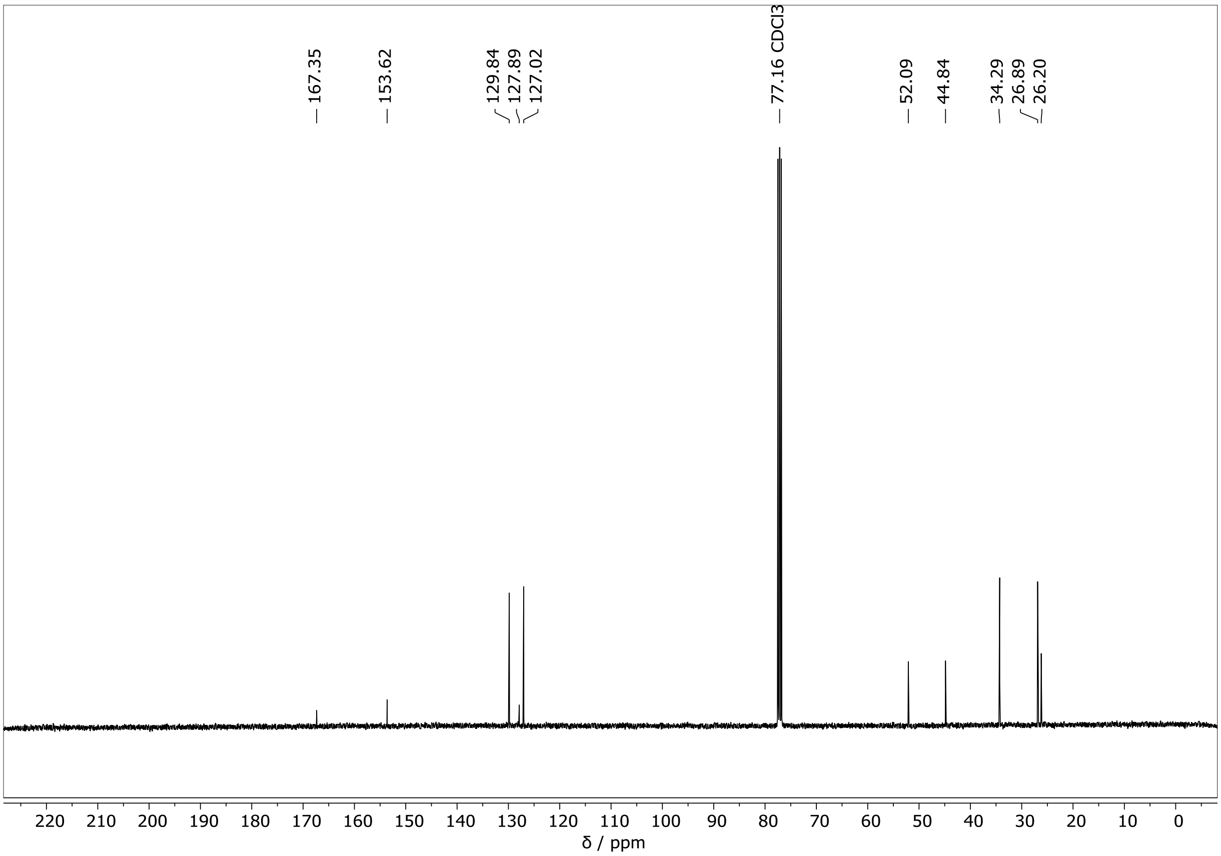

1-Cyclohexyl-3,5-dimethylbenzene (5l)

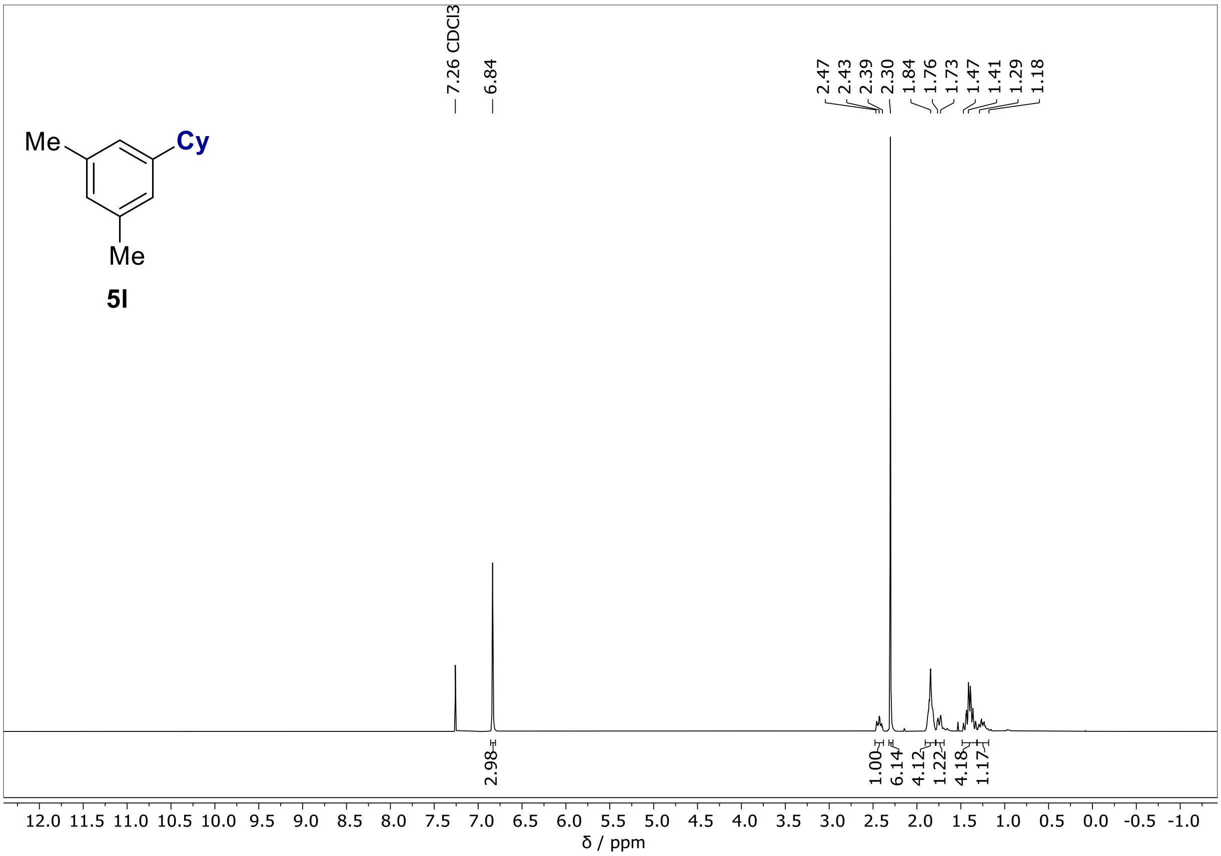

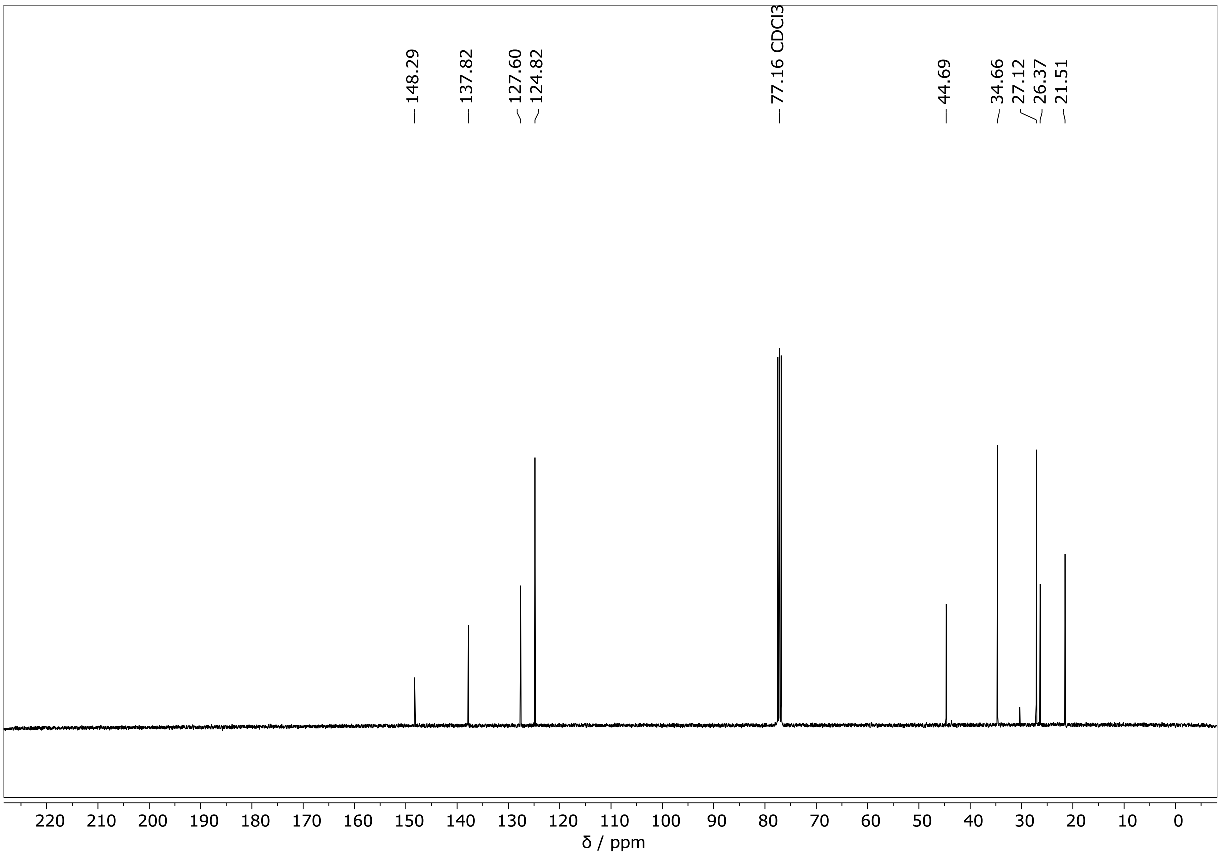

4-Cyclohexylbenzonitrile (5m)

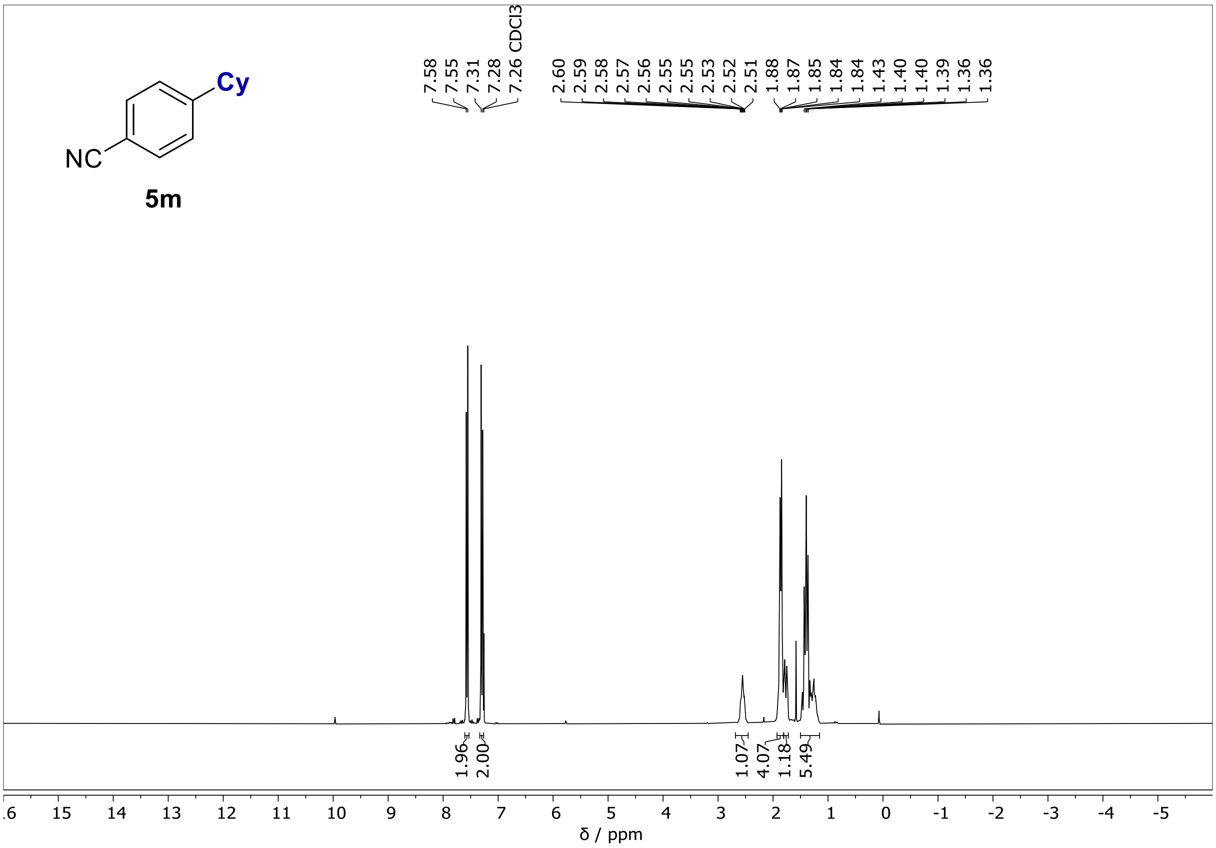

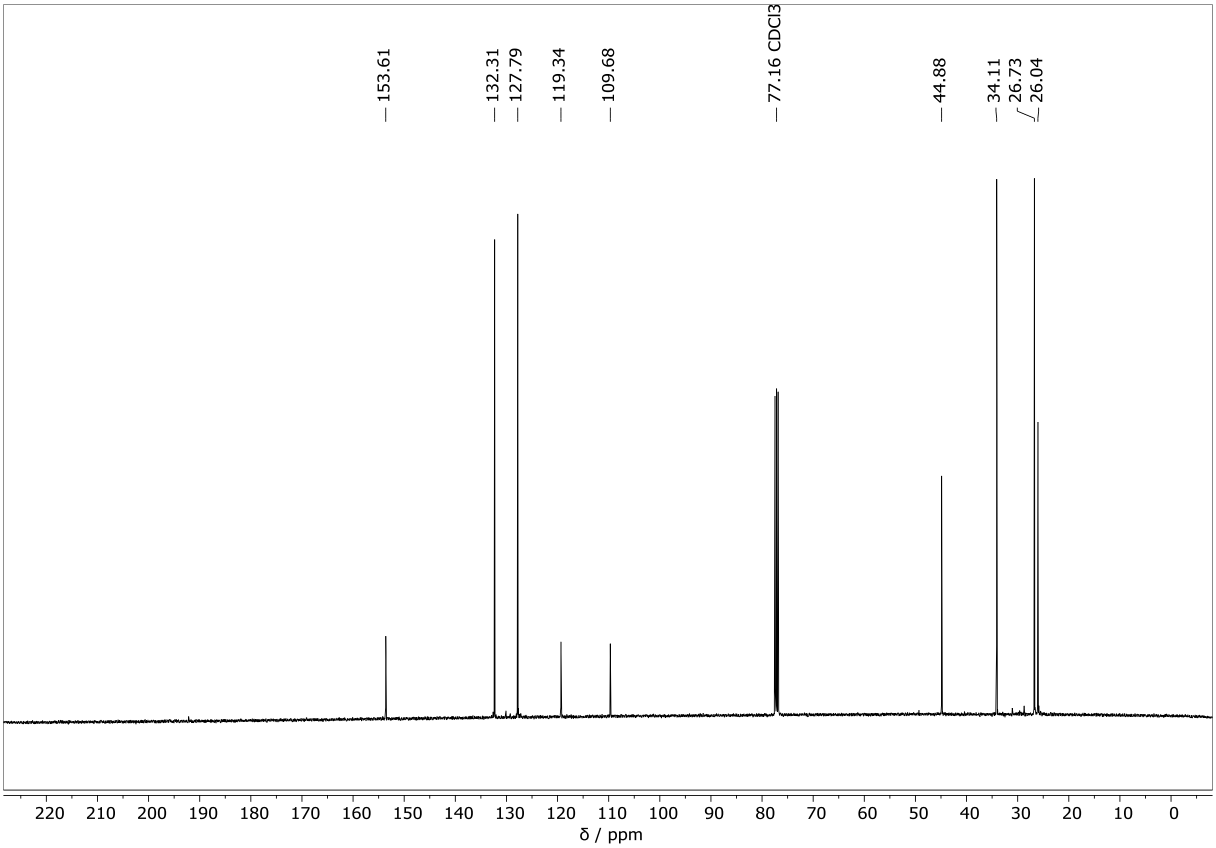

6-Cyclohexyl-2,3-dihydrobenzo[*b*][1,4]dioxine (5n)

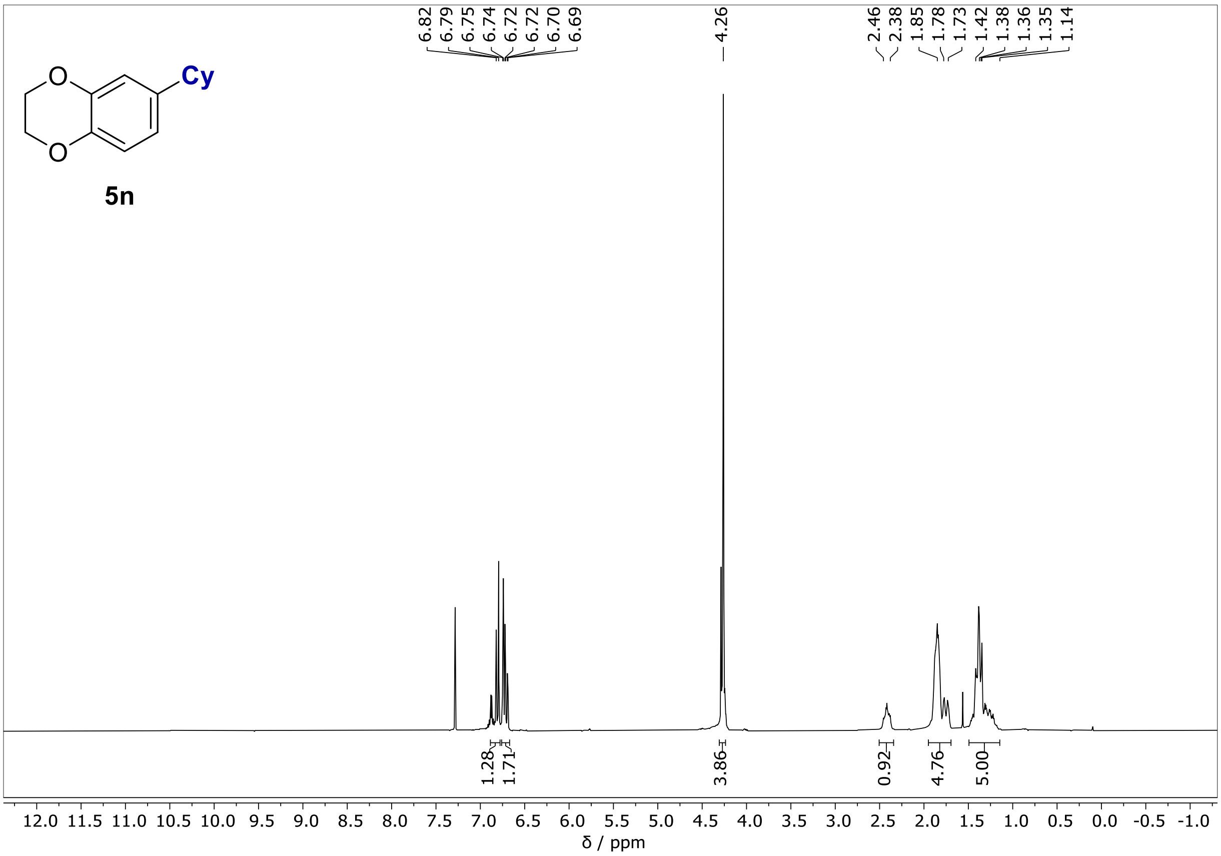

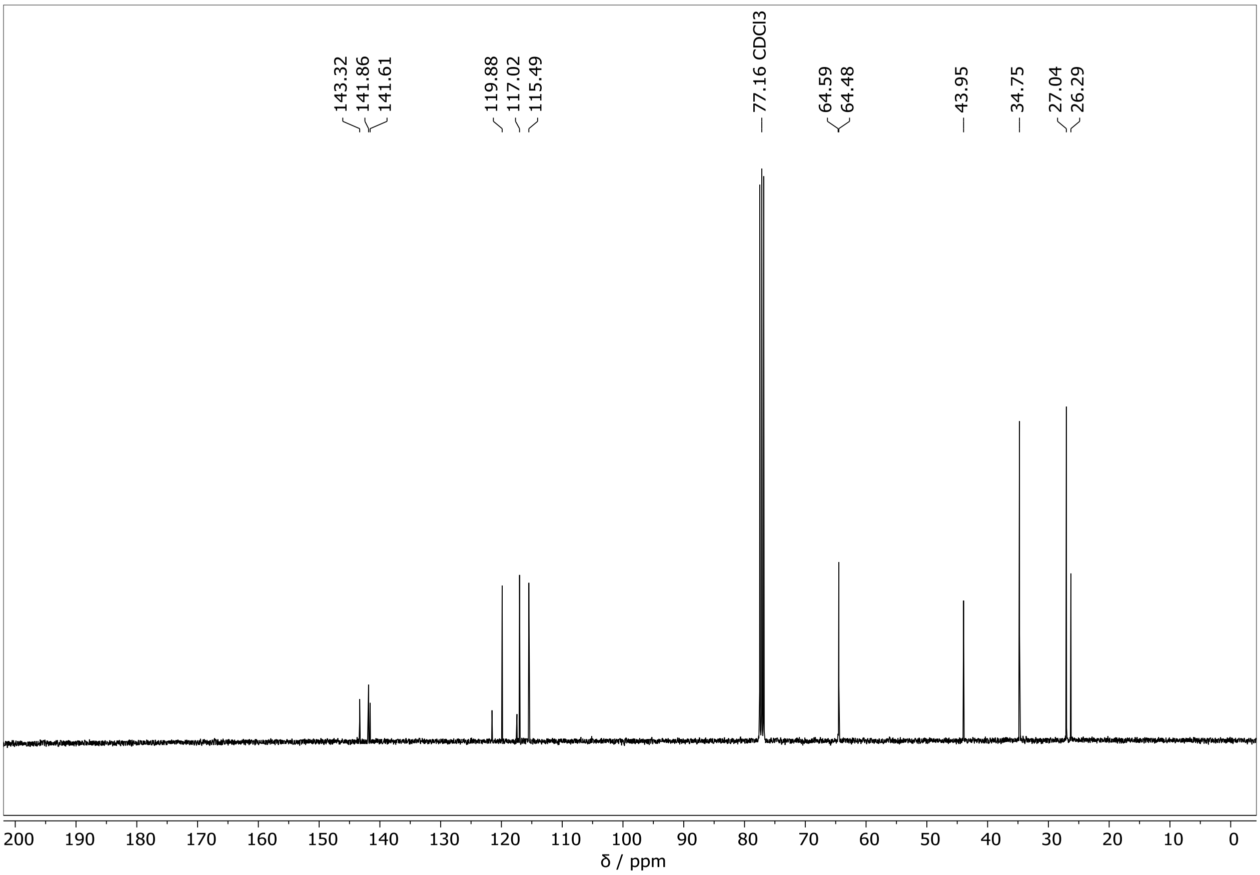

5-Cyclohexyl-2,2-difluorobenzo[*d*][1,3]dioxole (5o)

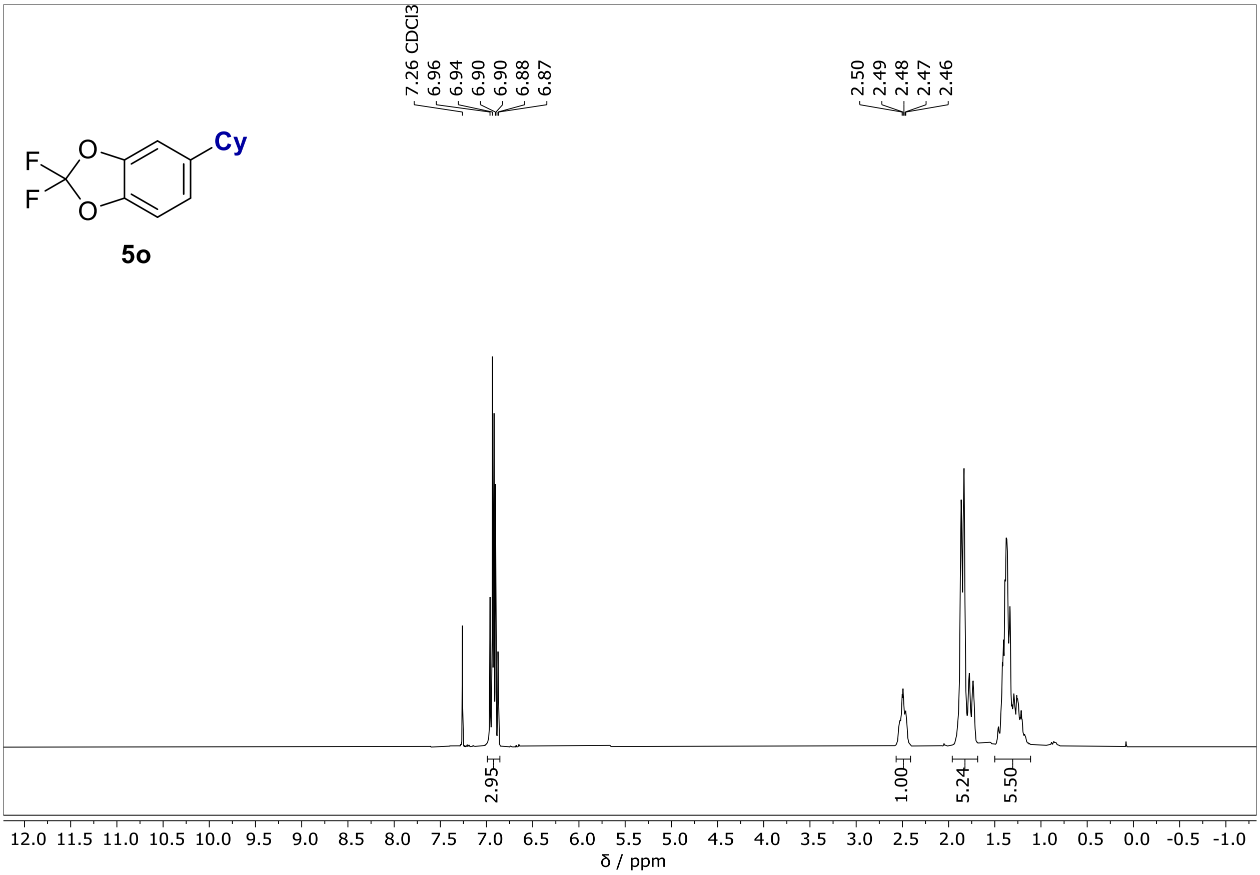

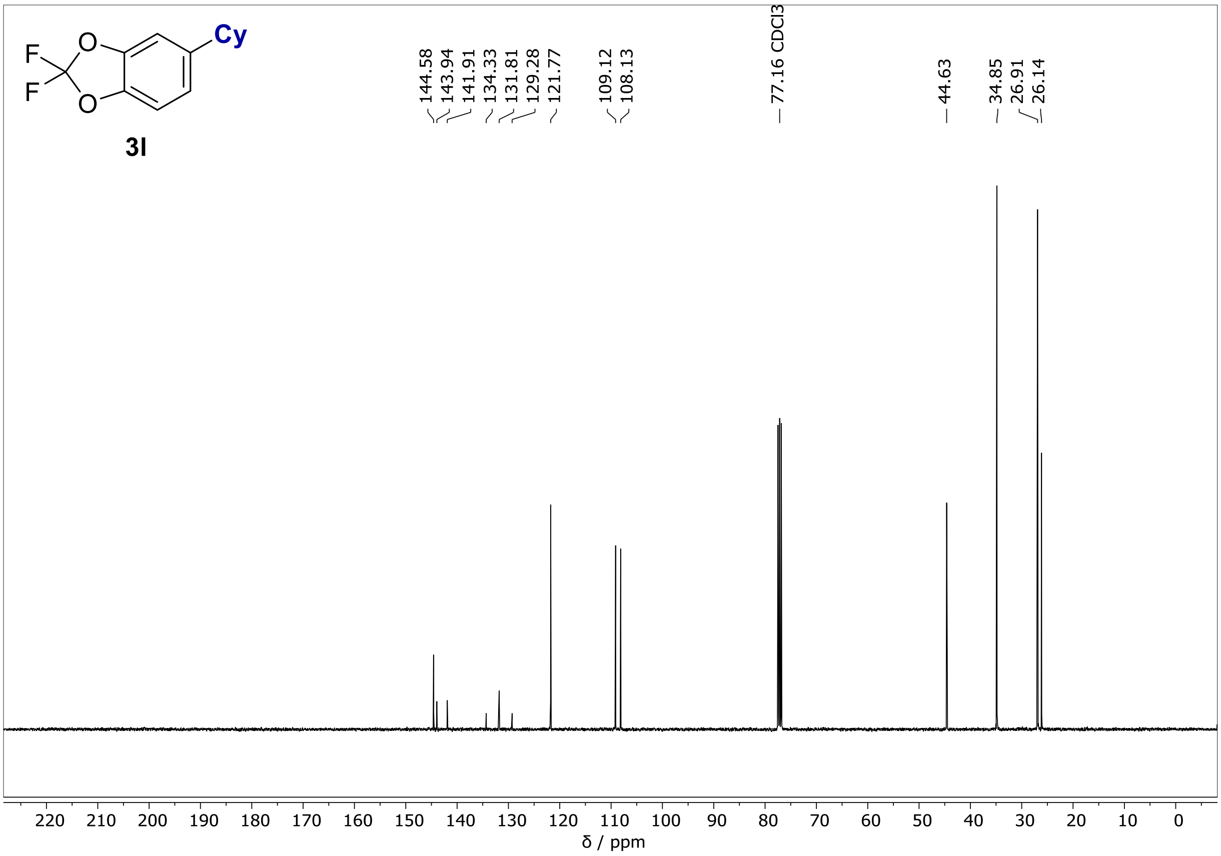

7-Cyclohexyl-1*H*-indole (5p)

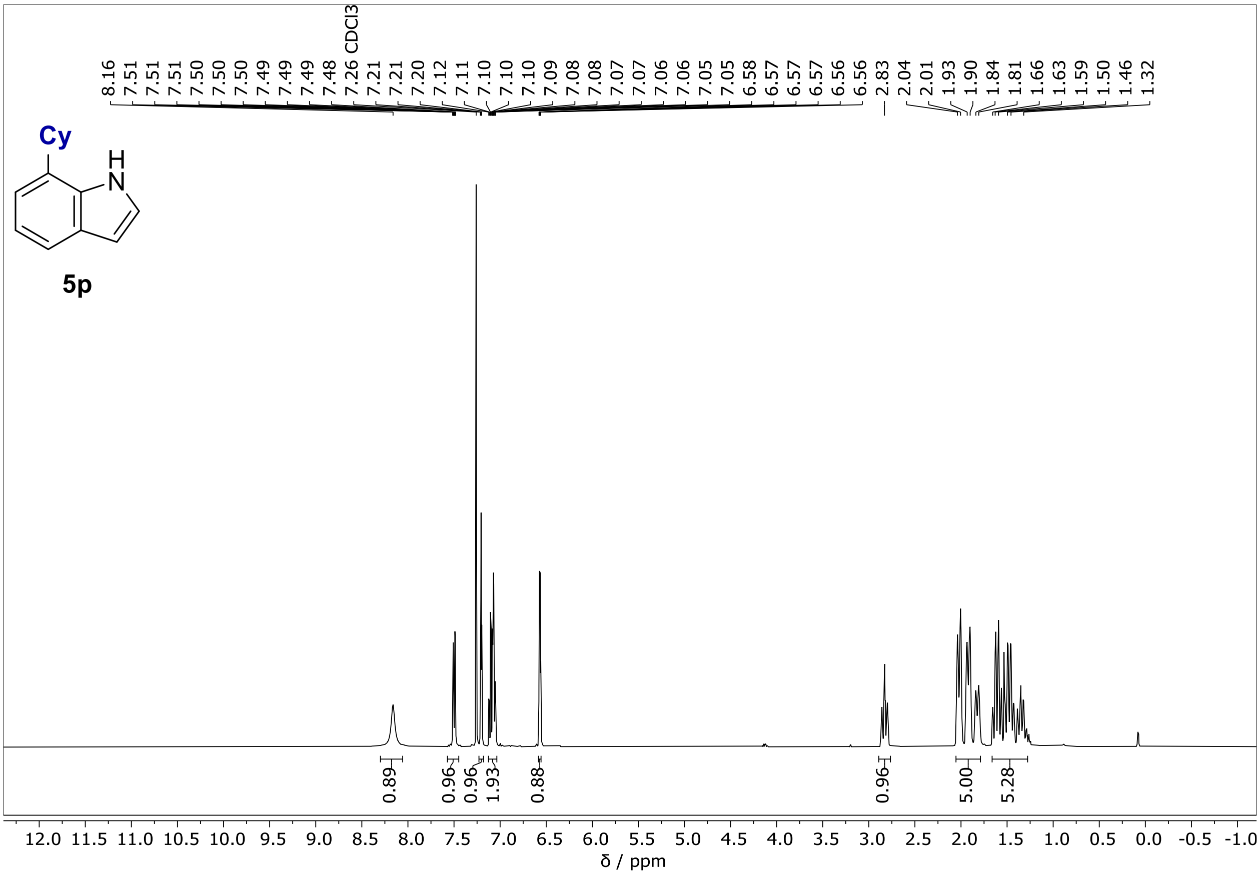

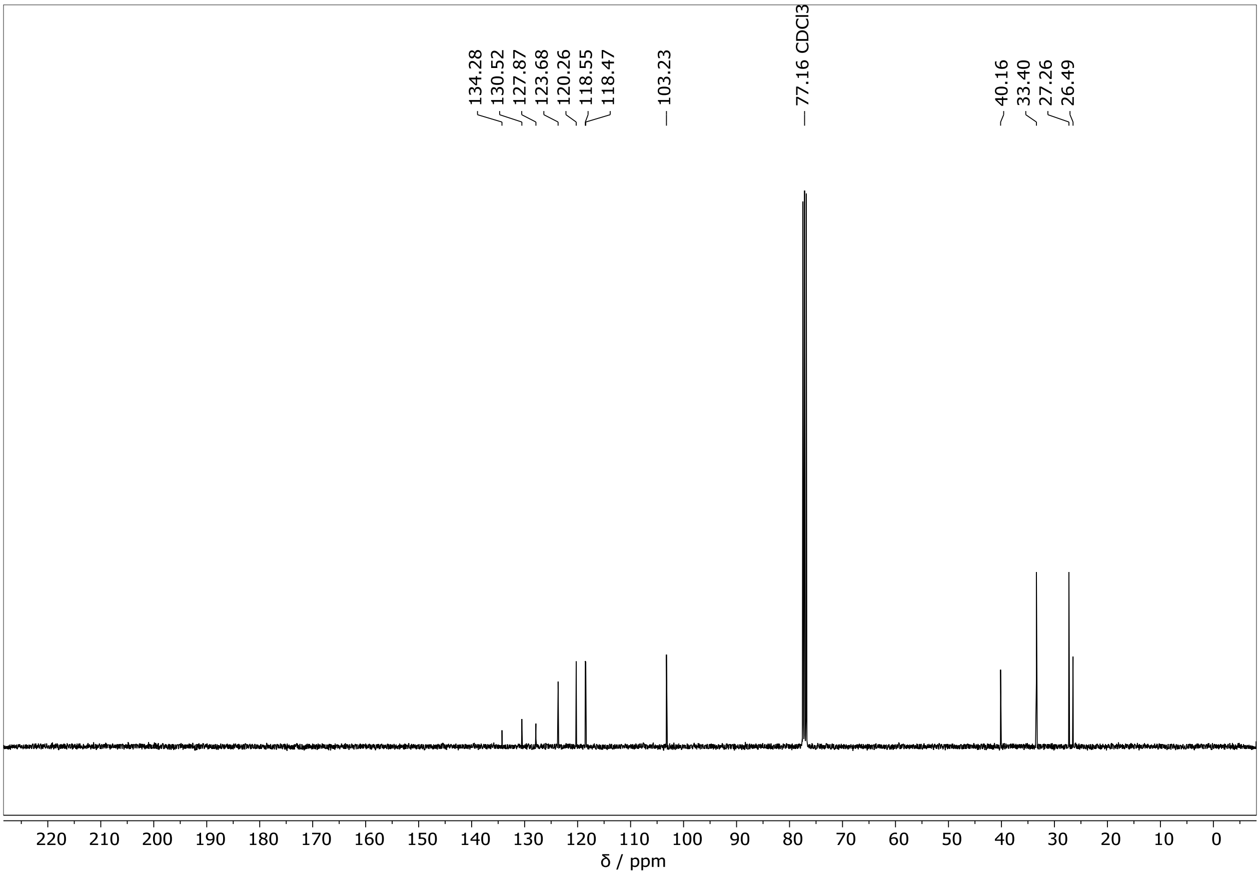

1-Chloro-4-cyclohexylbenzene (5q)

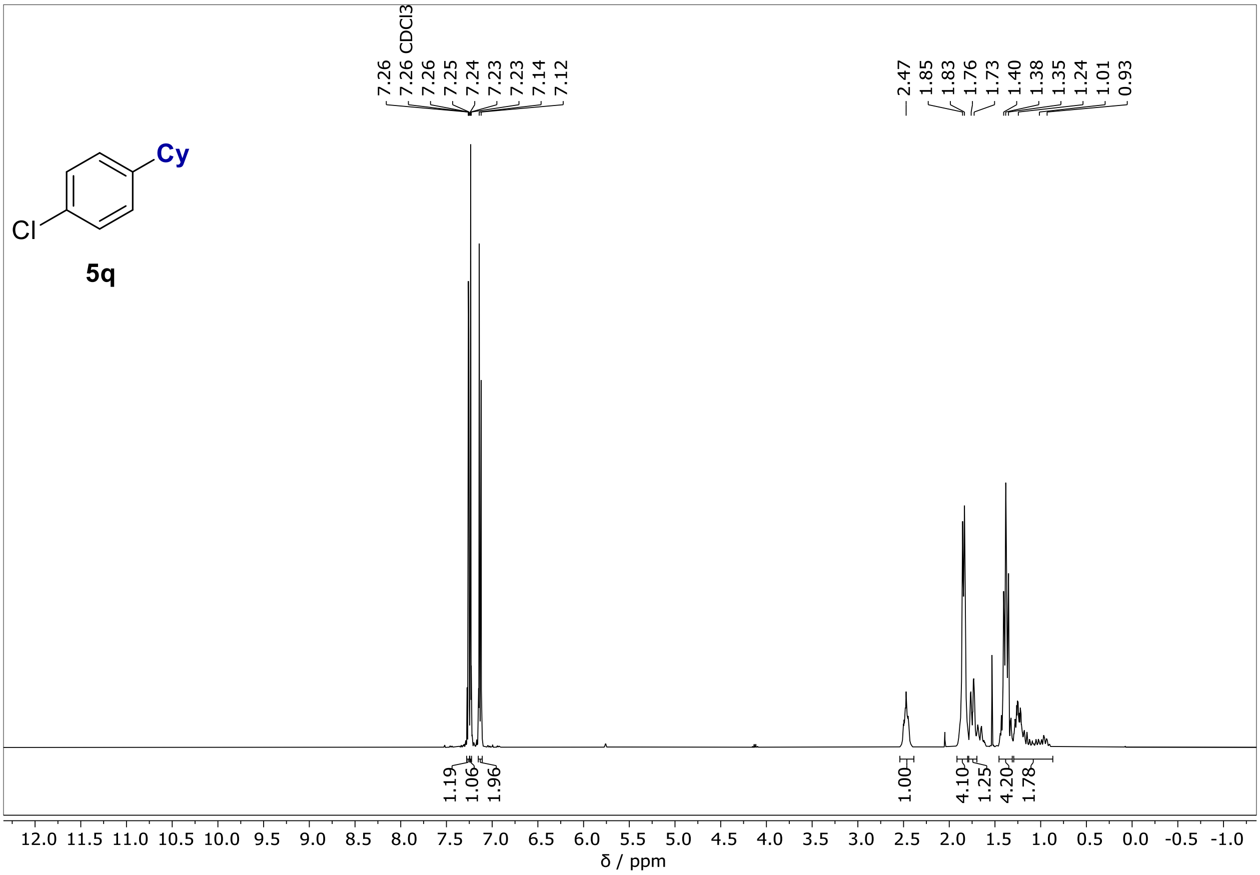

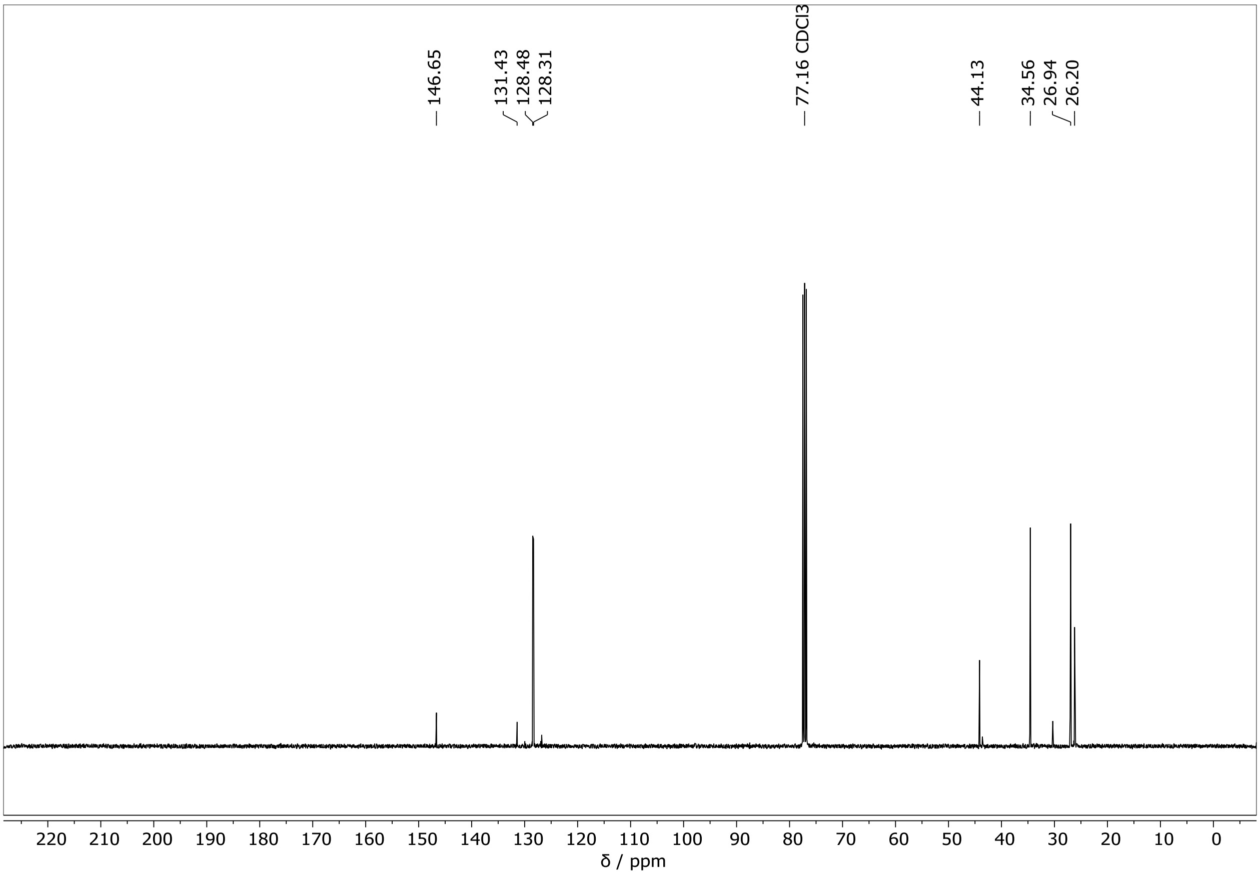

6-Cyclohexyl-2*H*-benzo[*b*][1,4]oxazin-3(4*H*)-one (5r)

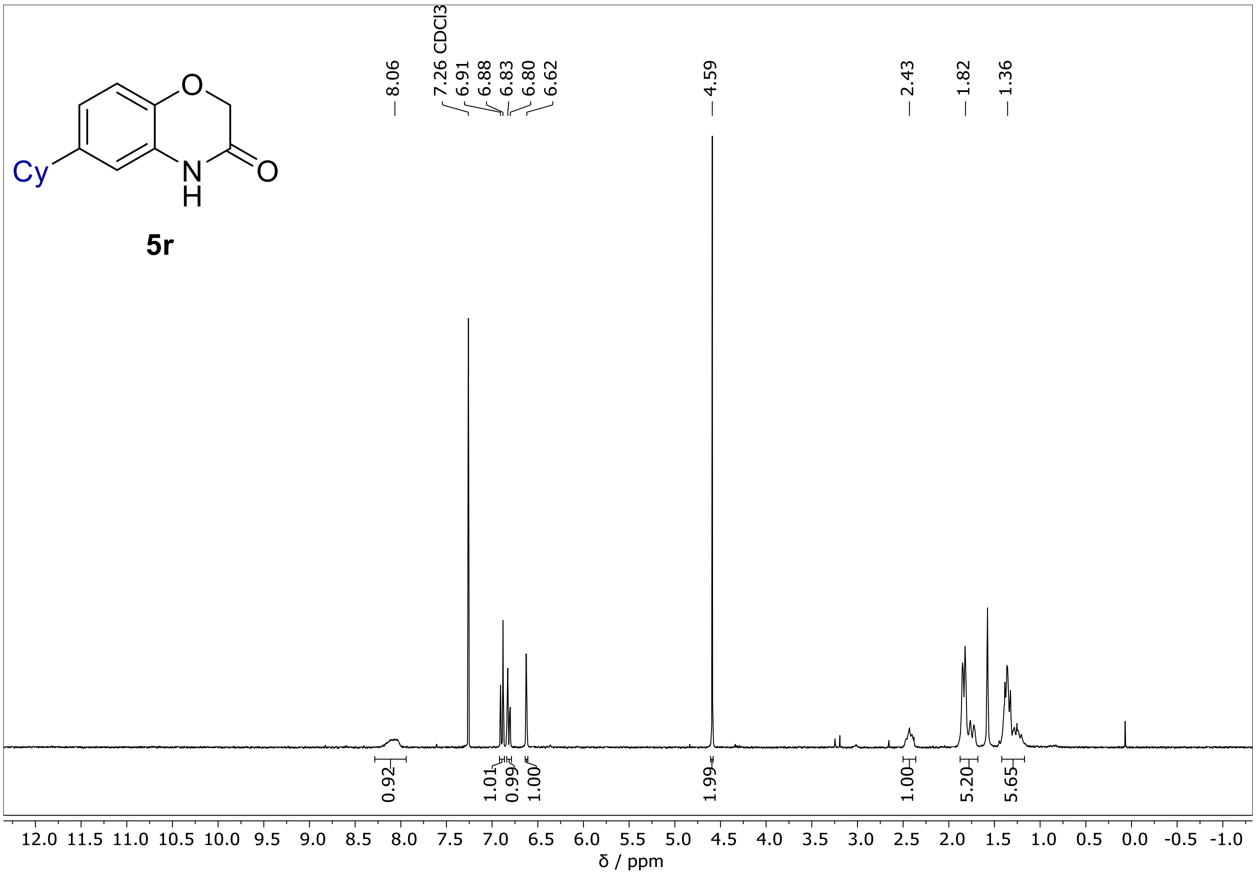

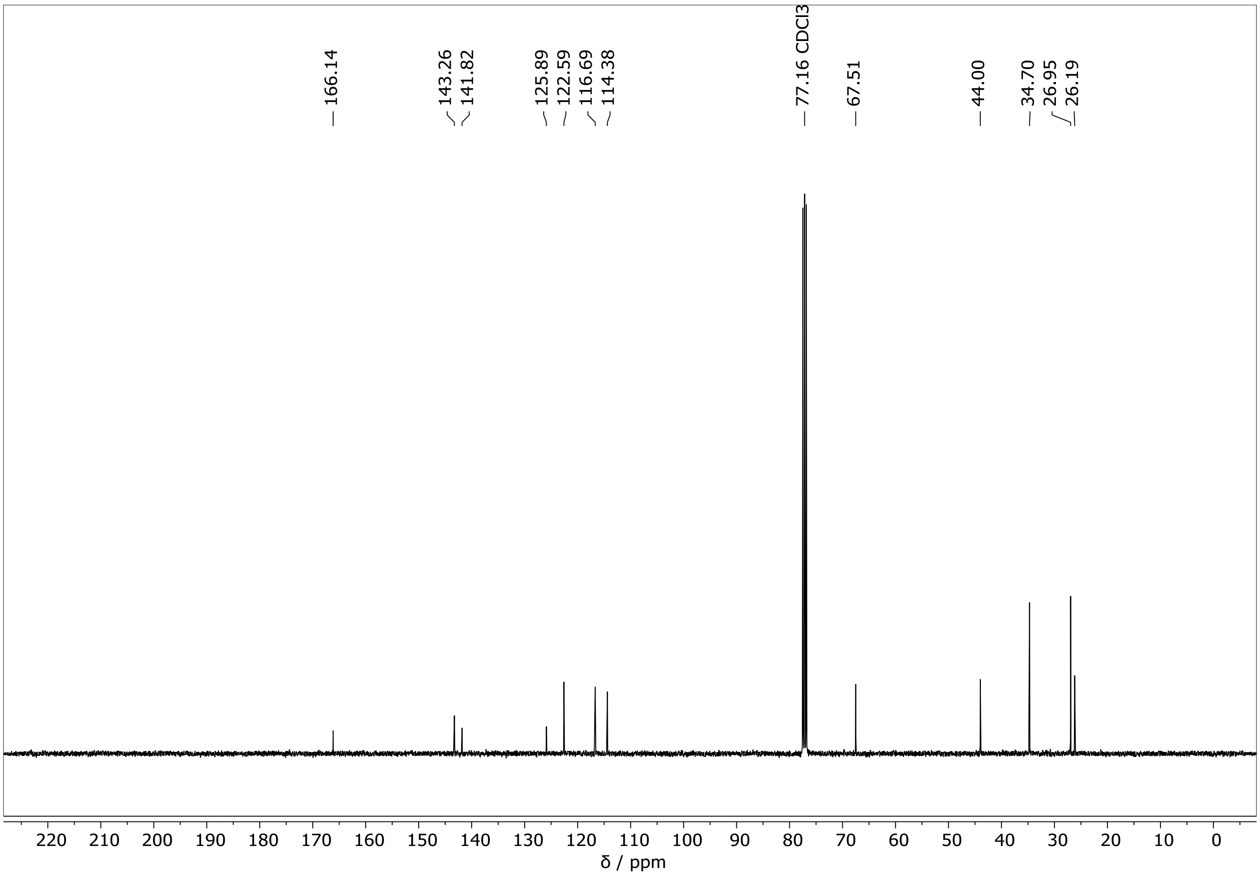

3-Cyclohexylaniline (5s)

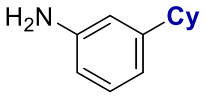

5s

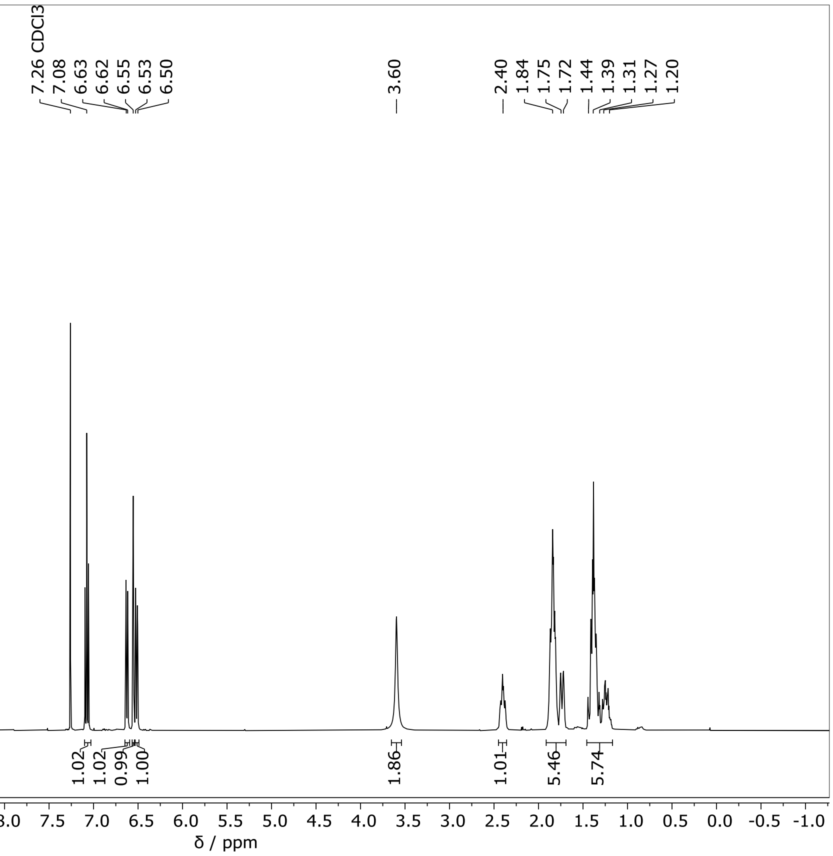

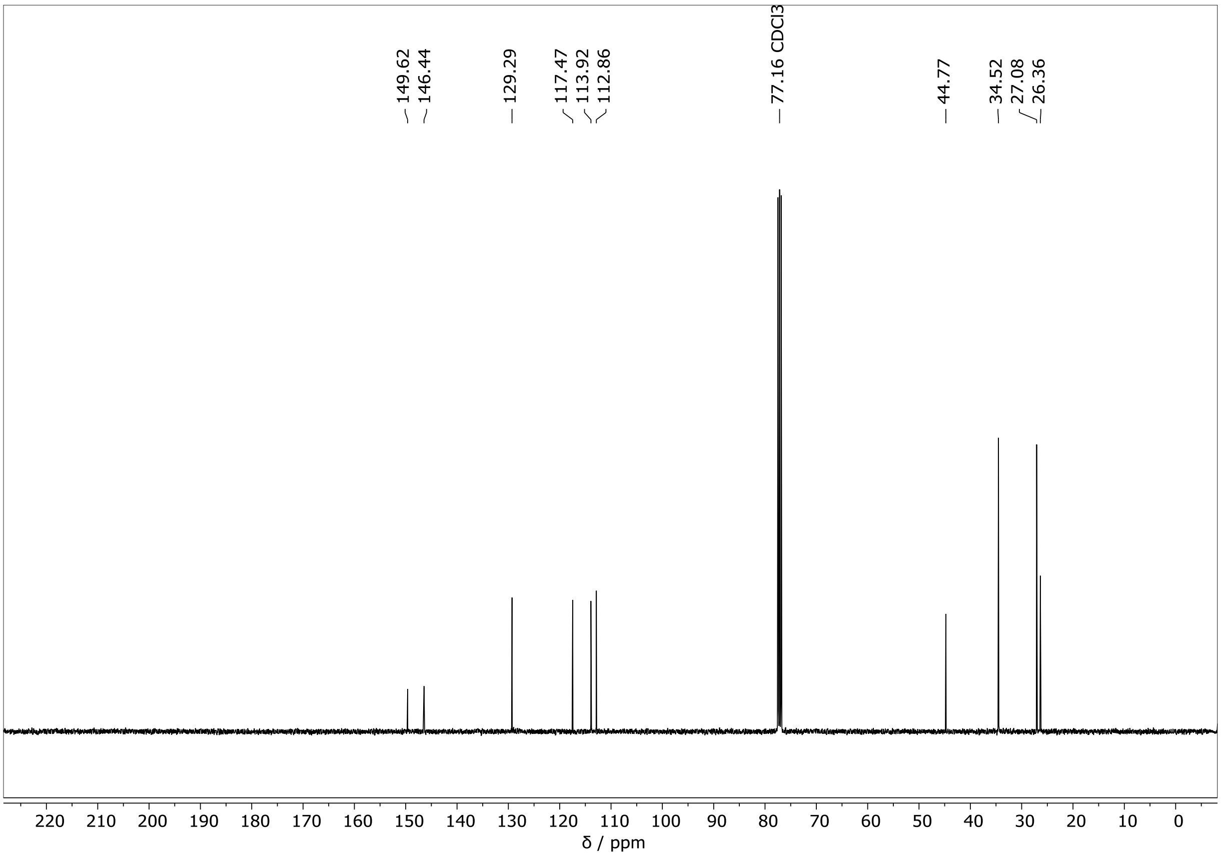

4-Cyclohexyl-2-methoxyaniline (5t)

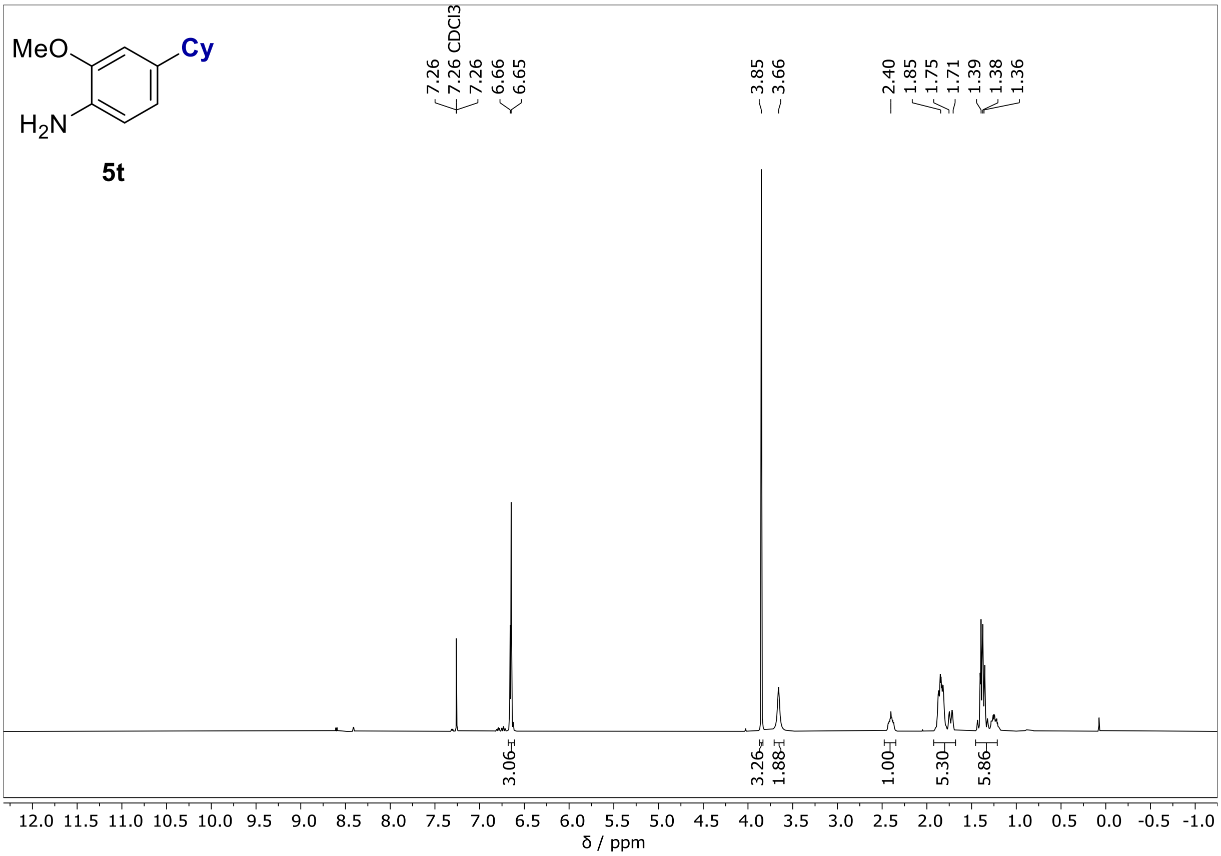

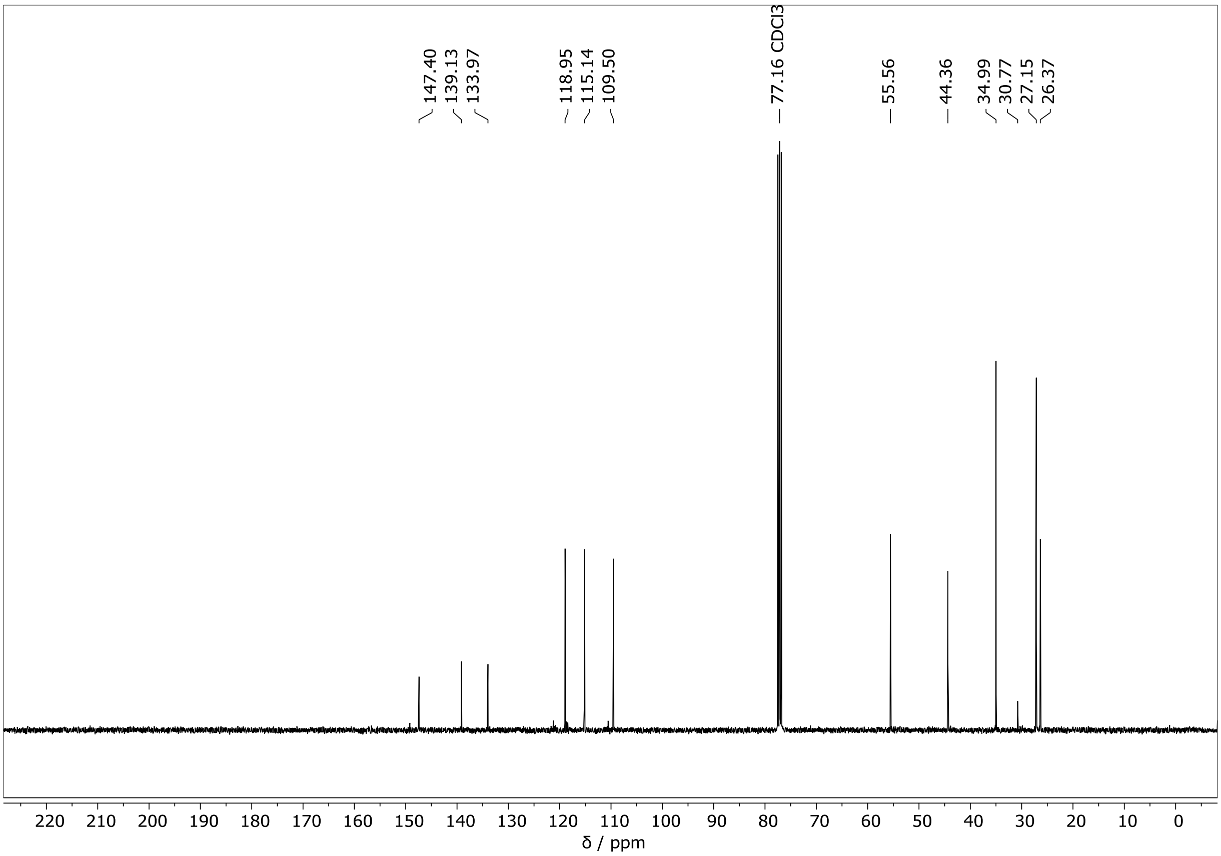

3-Cyclohexyl-4-methylaniline (5u)

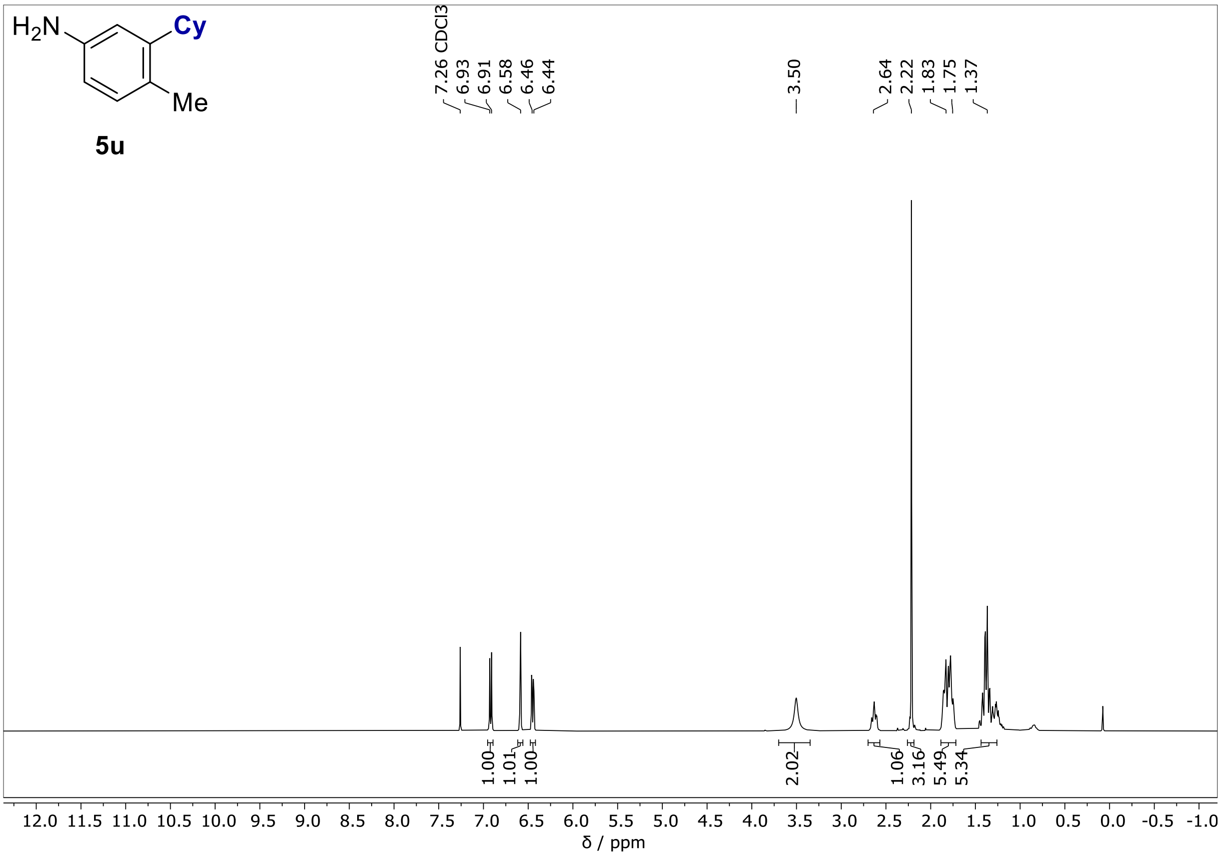

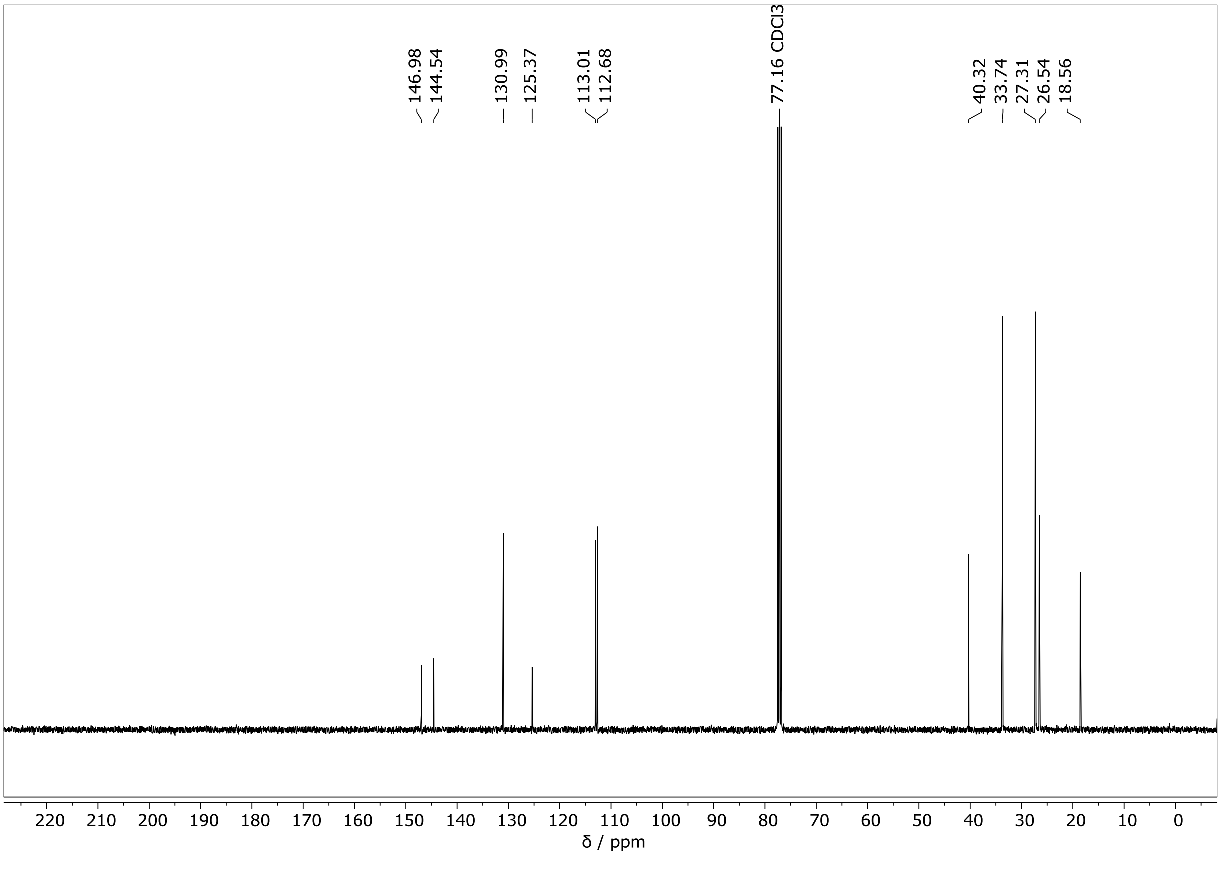

3-Cyclohexyl-2-methylaniline (5v)

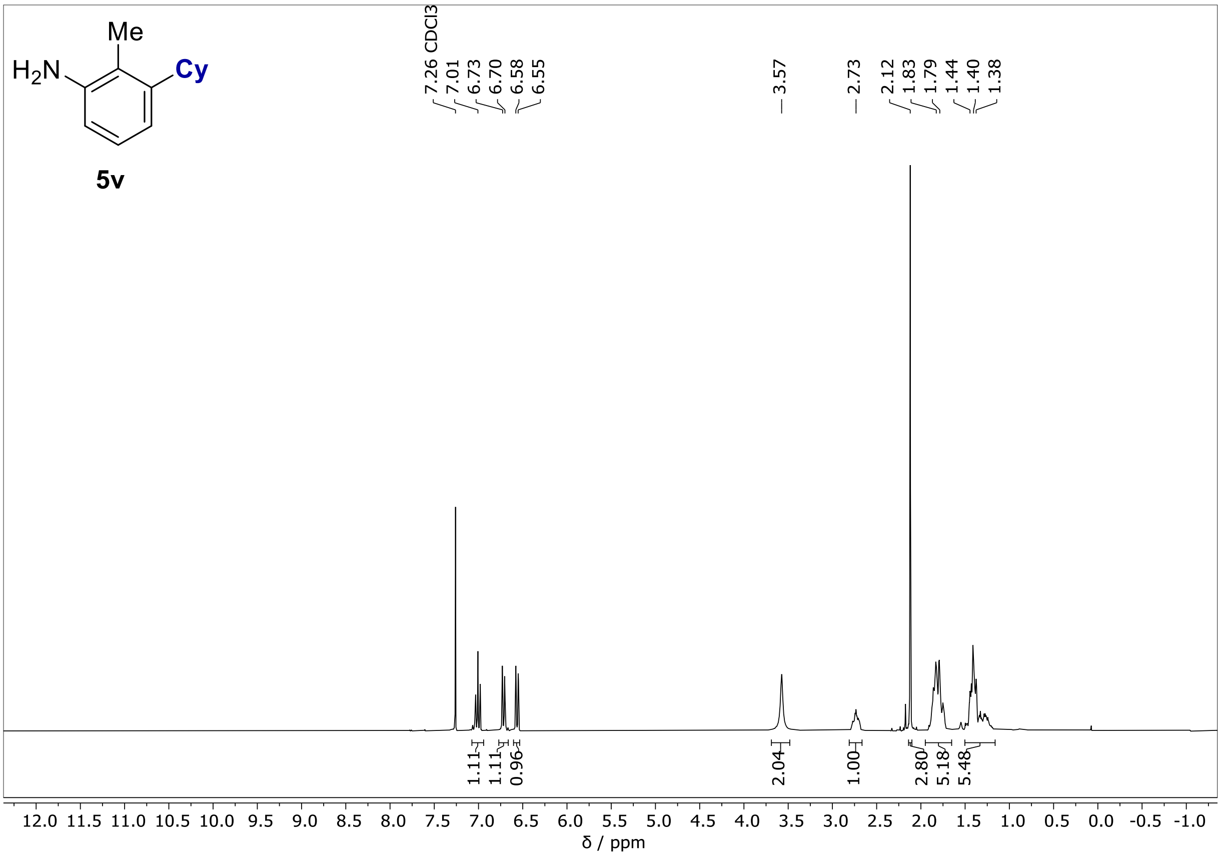

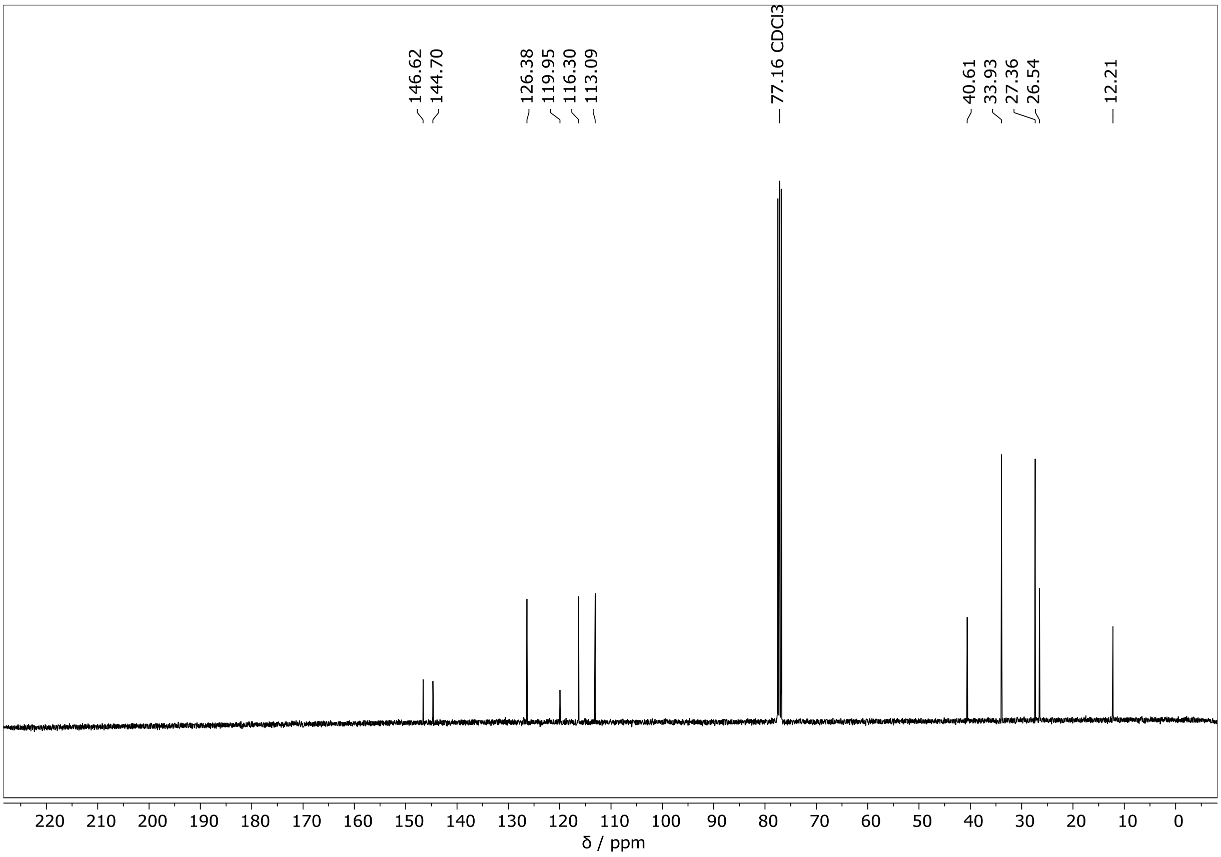

2-Cyclohexyl-*N*,*N*-dimethylaniline (5w)

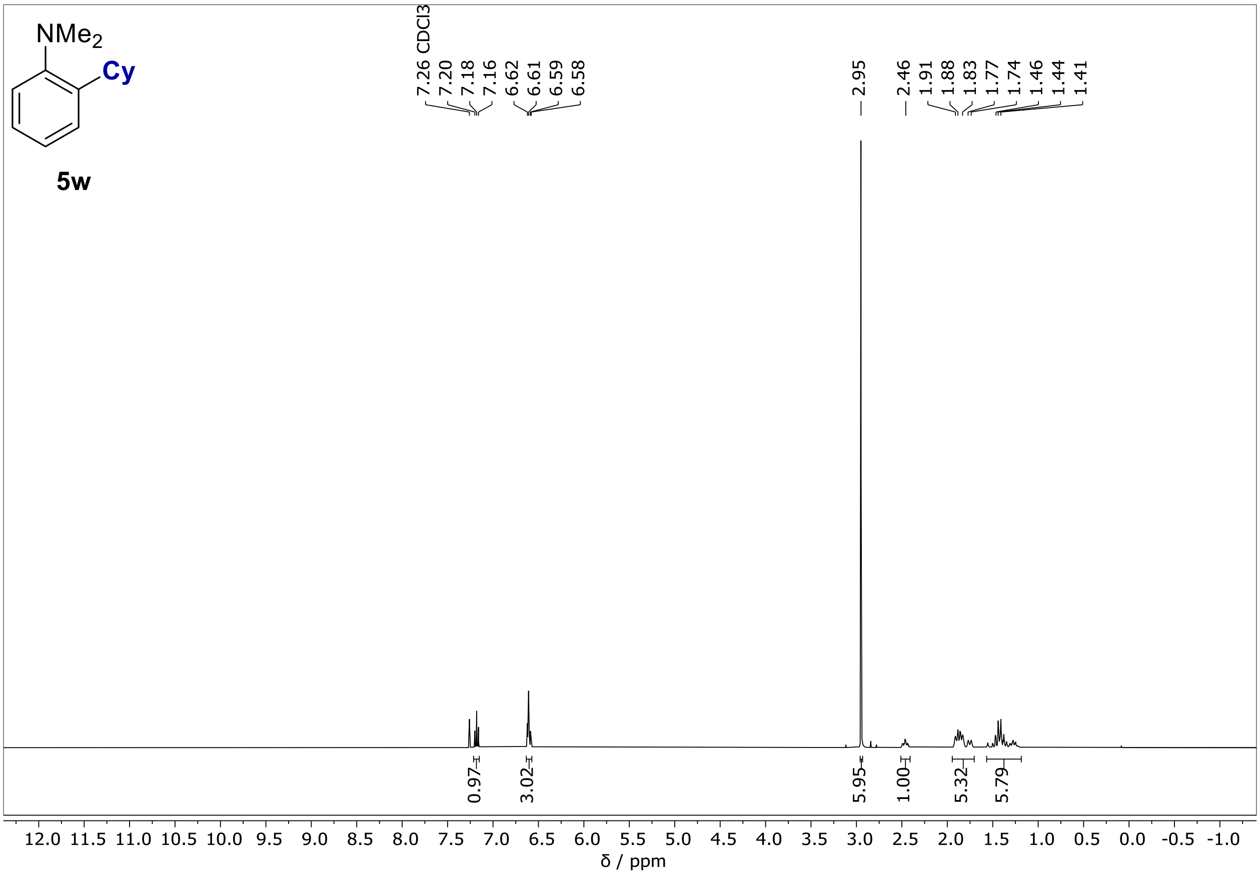

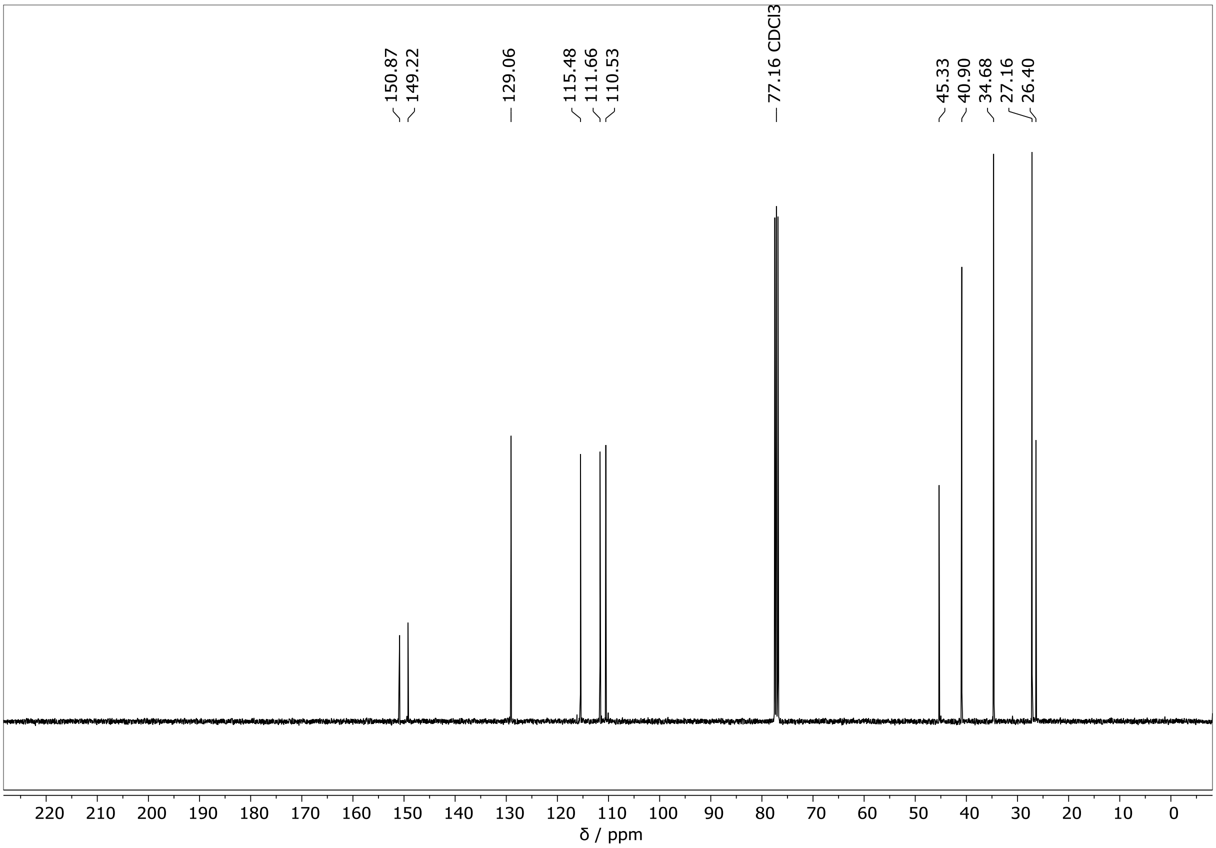

Alkyl radical scope

1-Cyclopentyl-4-methoxybenzene (5aa)

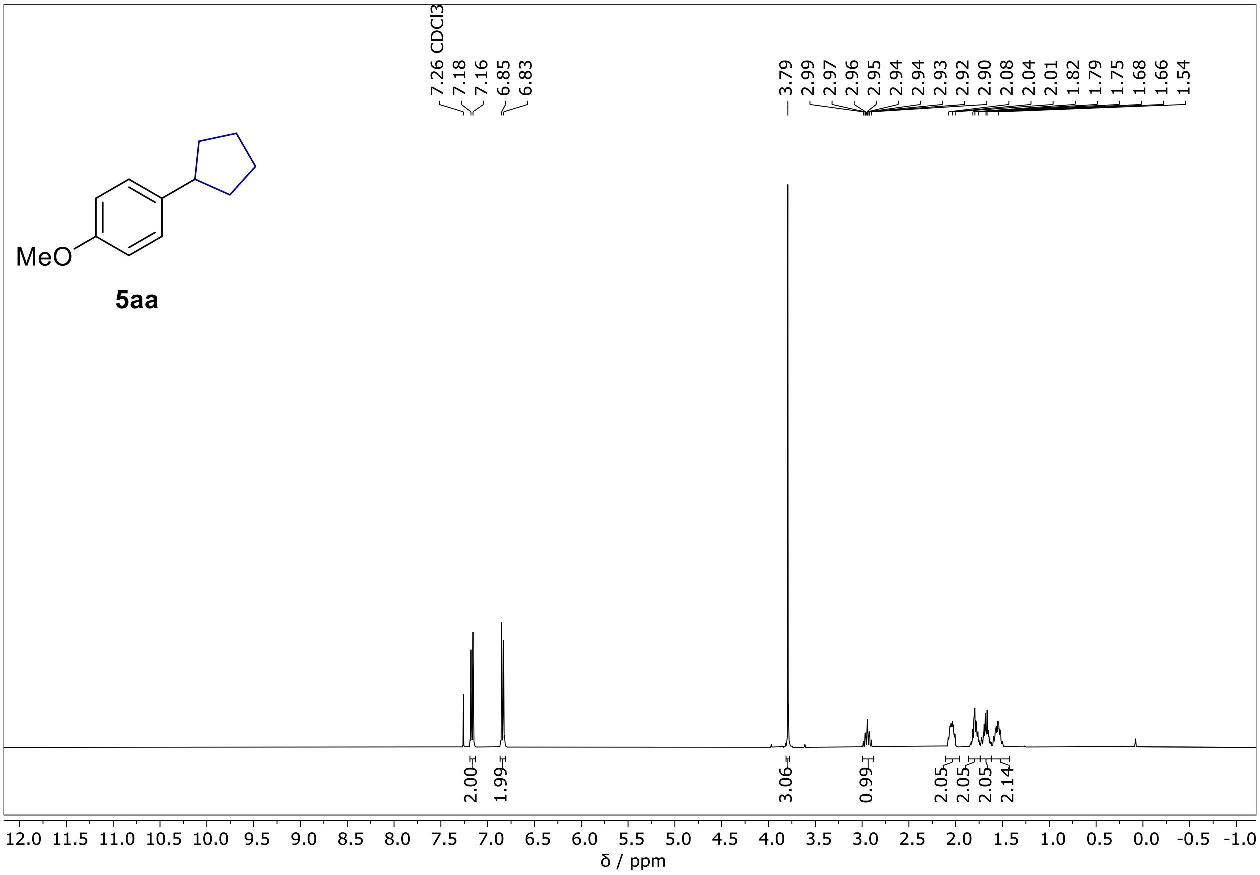

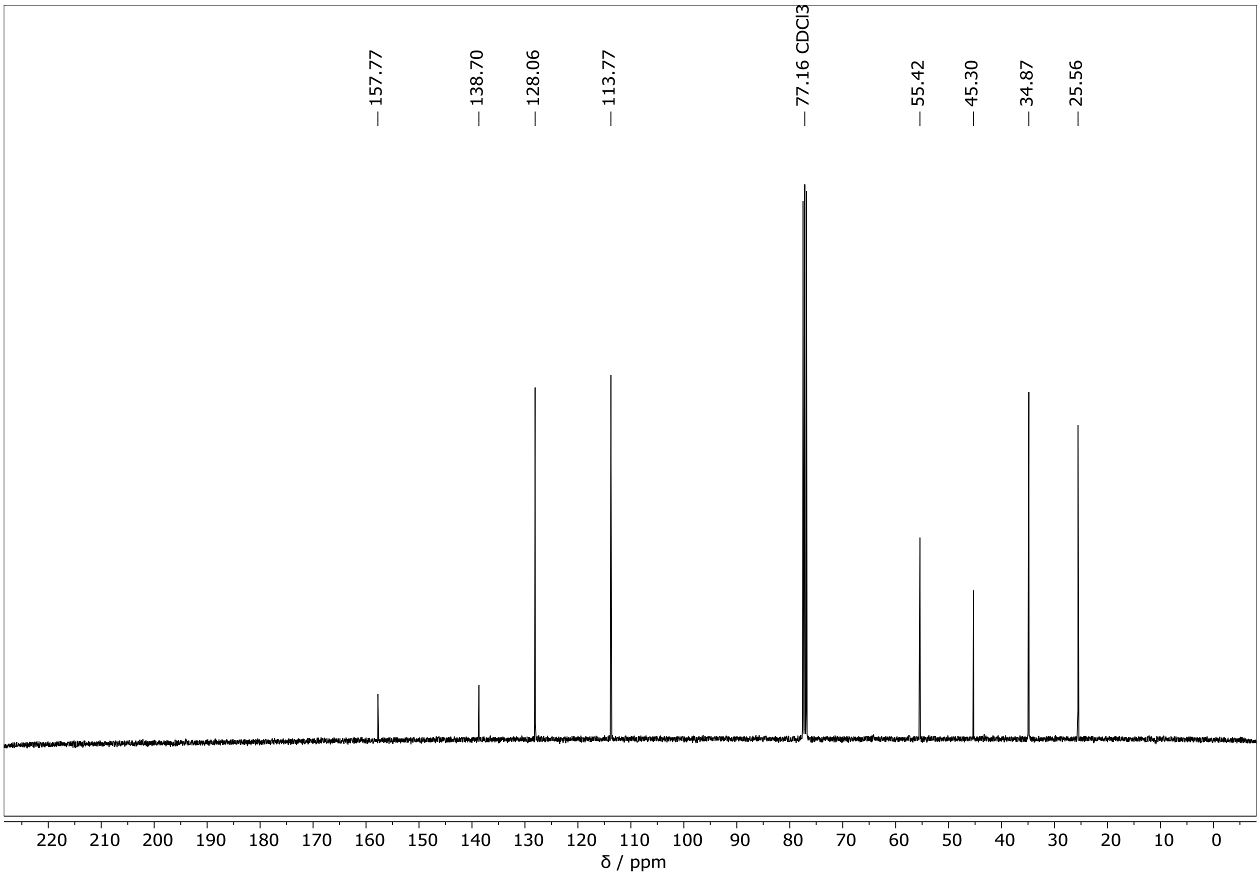
157.77
138.70
128.06
113.77
77.16 CDCl3
55.42
45.30
34.87
25.56
220 210 200 190 180 170 160 150 140 130 120 110 100 90 80 70 60 50 40 30 20 10 0
δ / ppm

Tert-butyl 3-(4-methoxyphenyl)pyrrolidine-1-carboxylate (5ab)

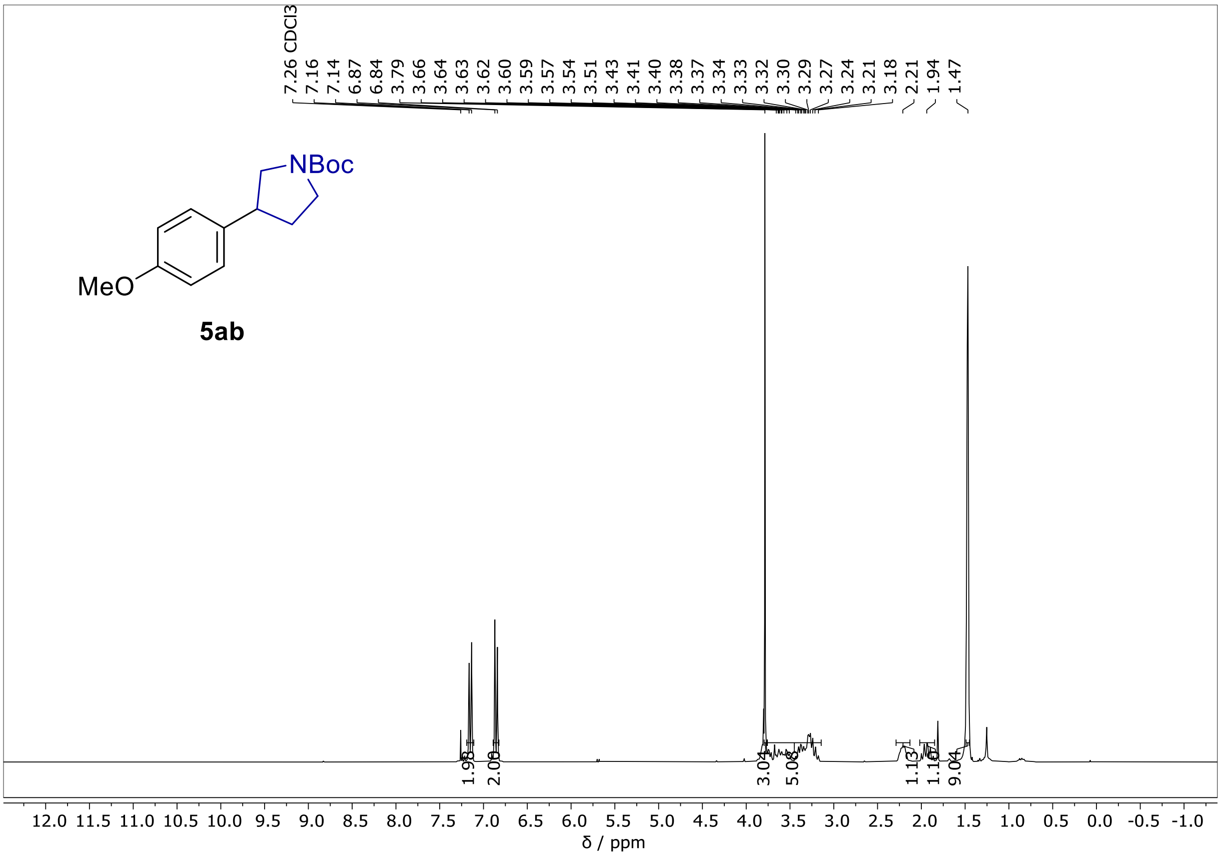

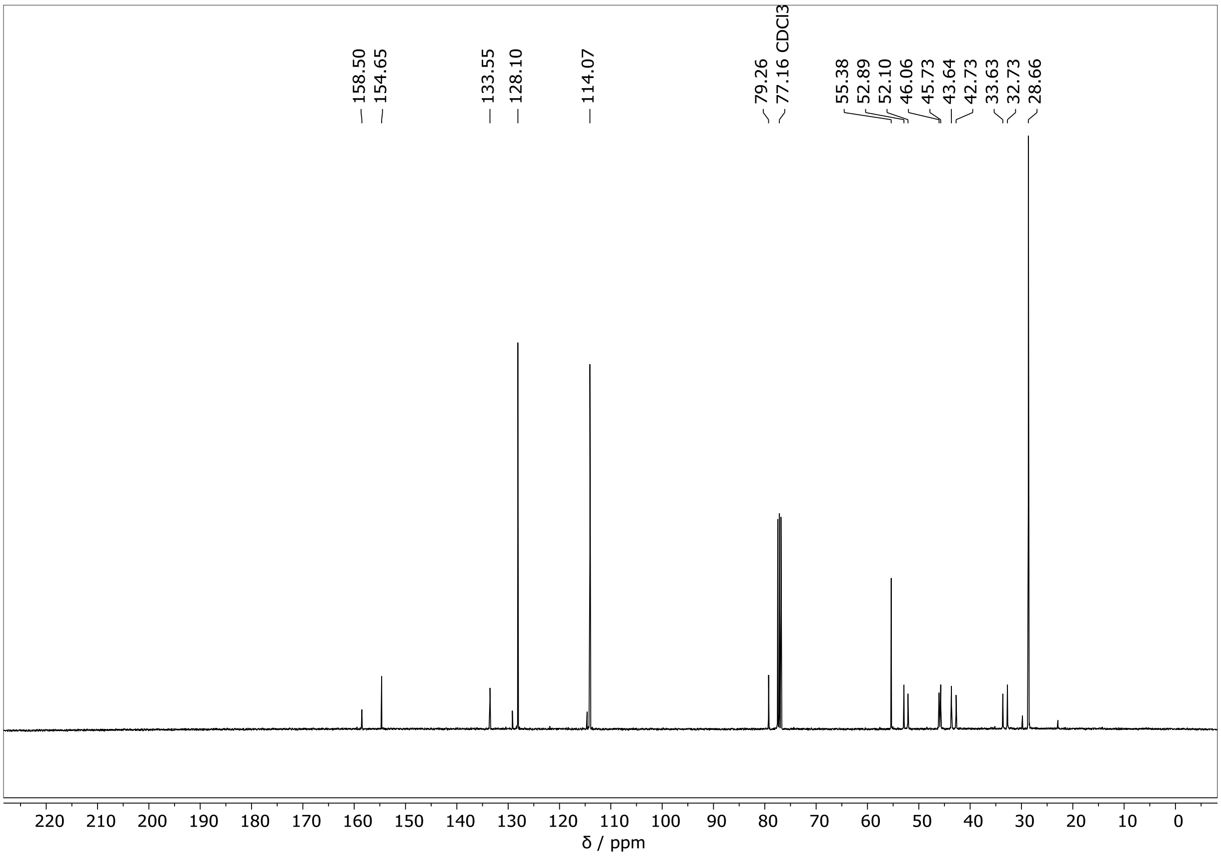

1-Butyl-4-methoxybenzene (5ac)

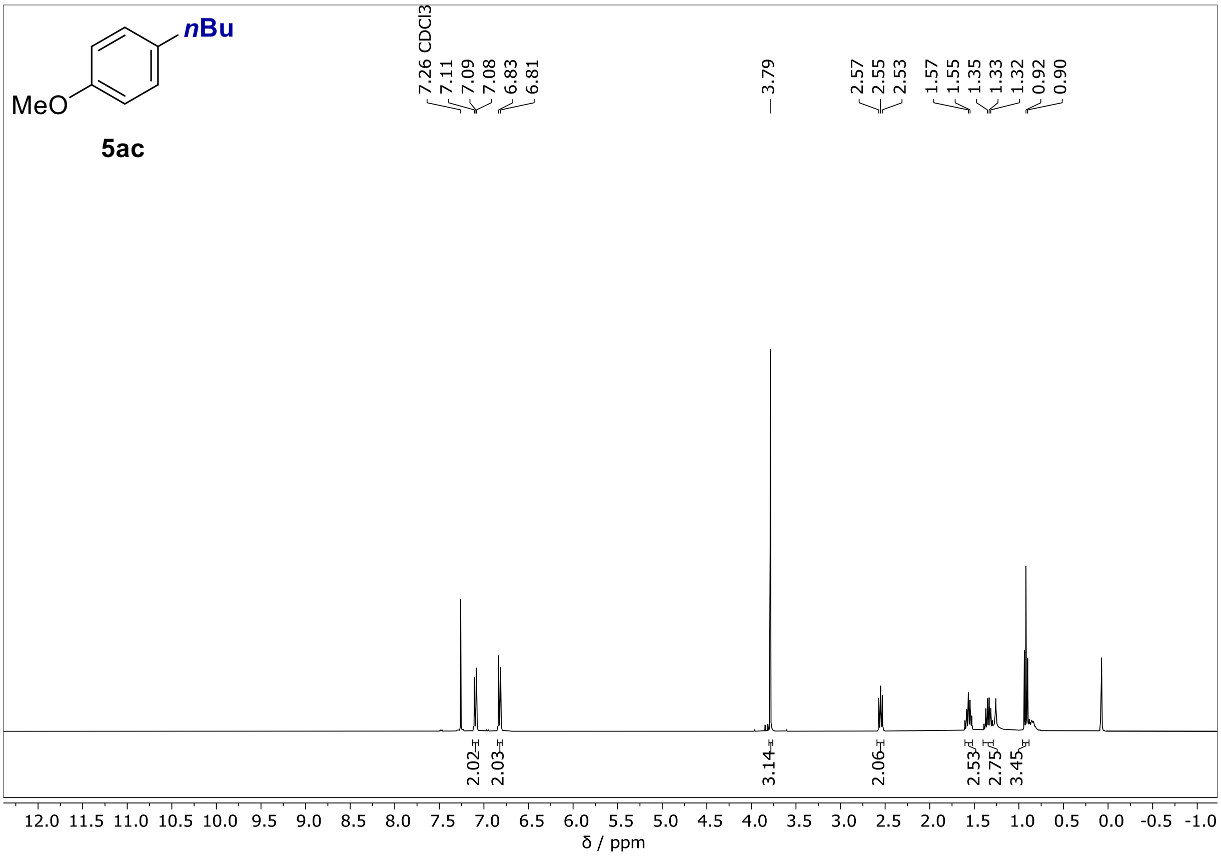

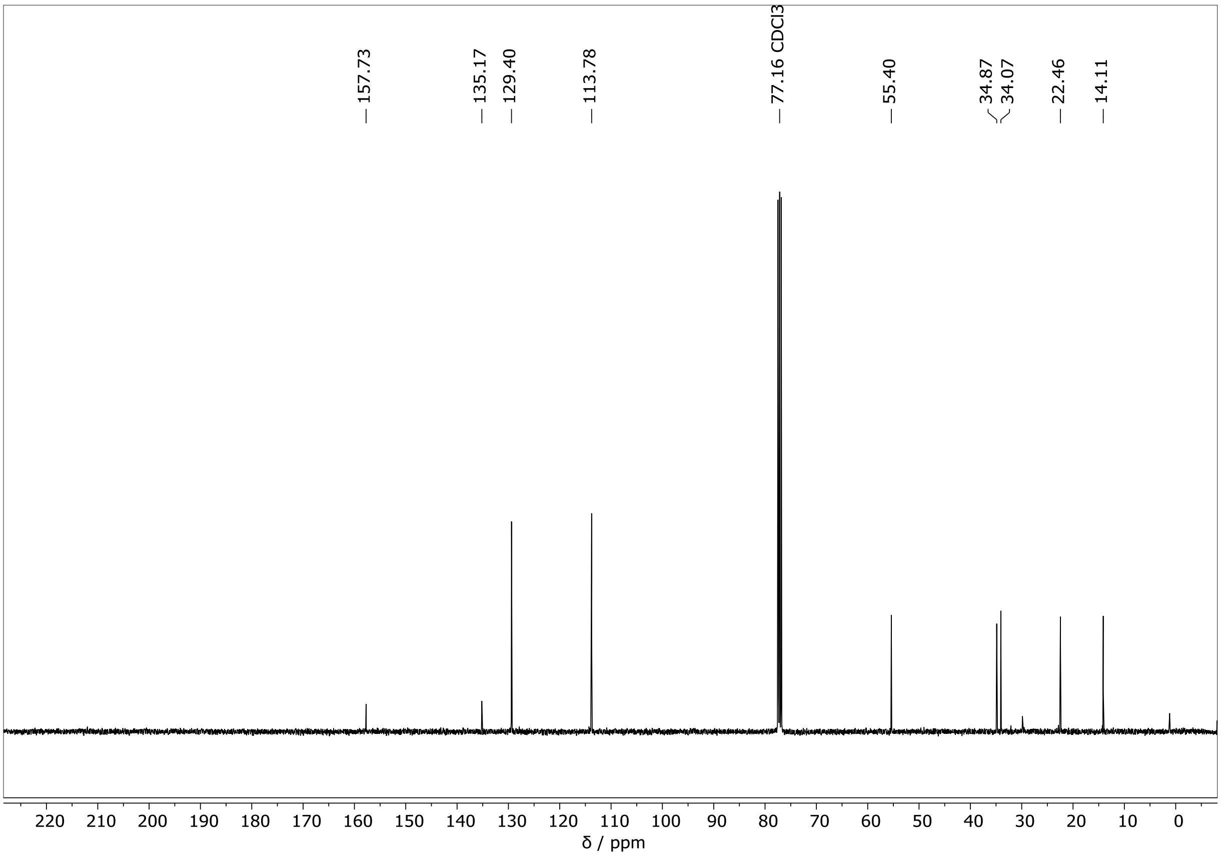

1-Isopropyl-4-(2-(4-methoxyphenyl)propyl)benzene (5ad)

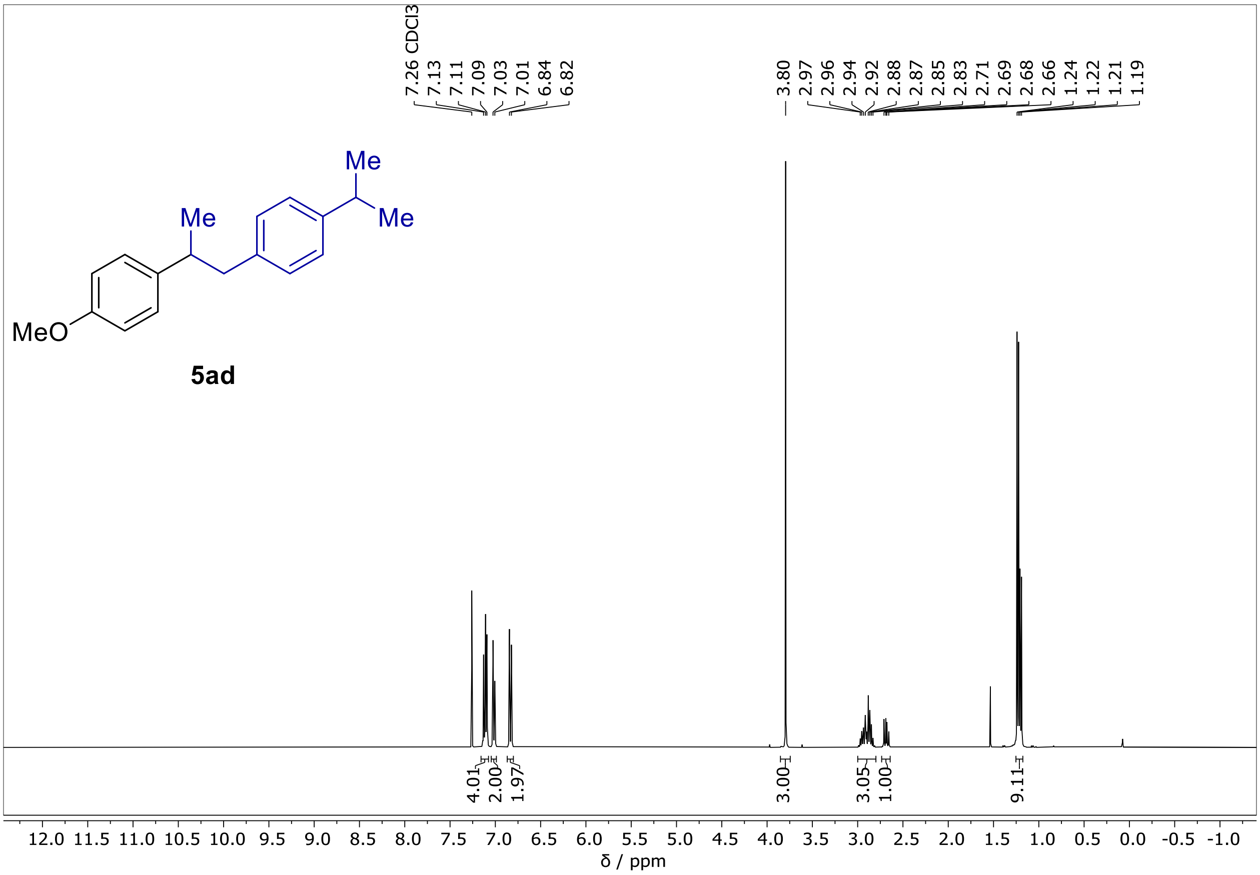

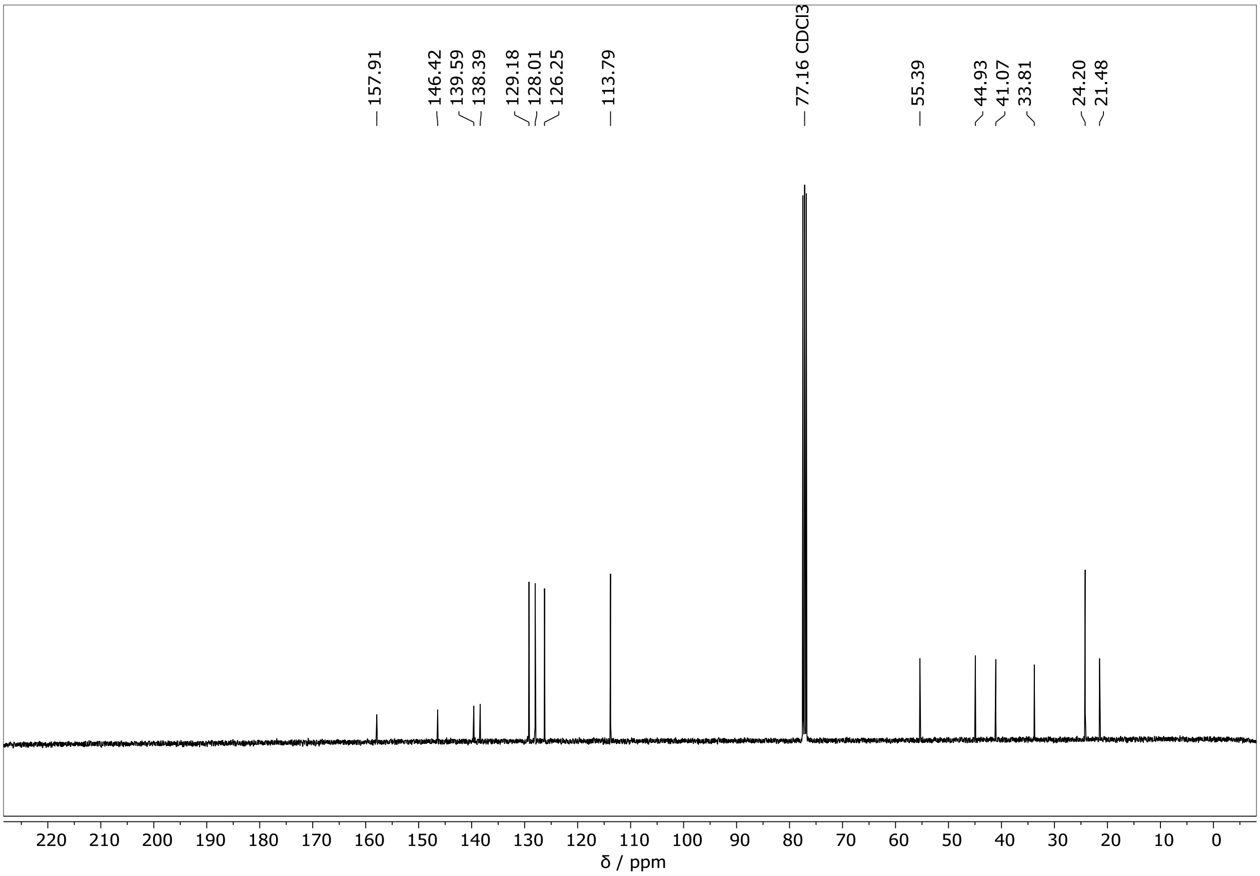

1-Benzyl-4-methoxybenzene (5ae)

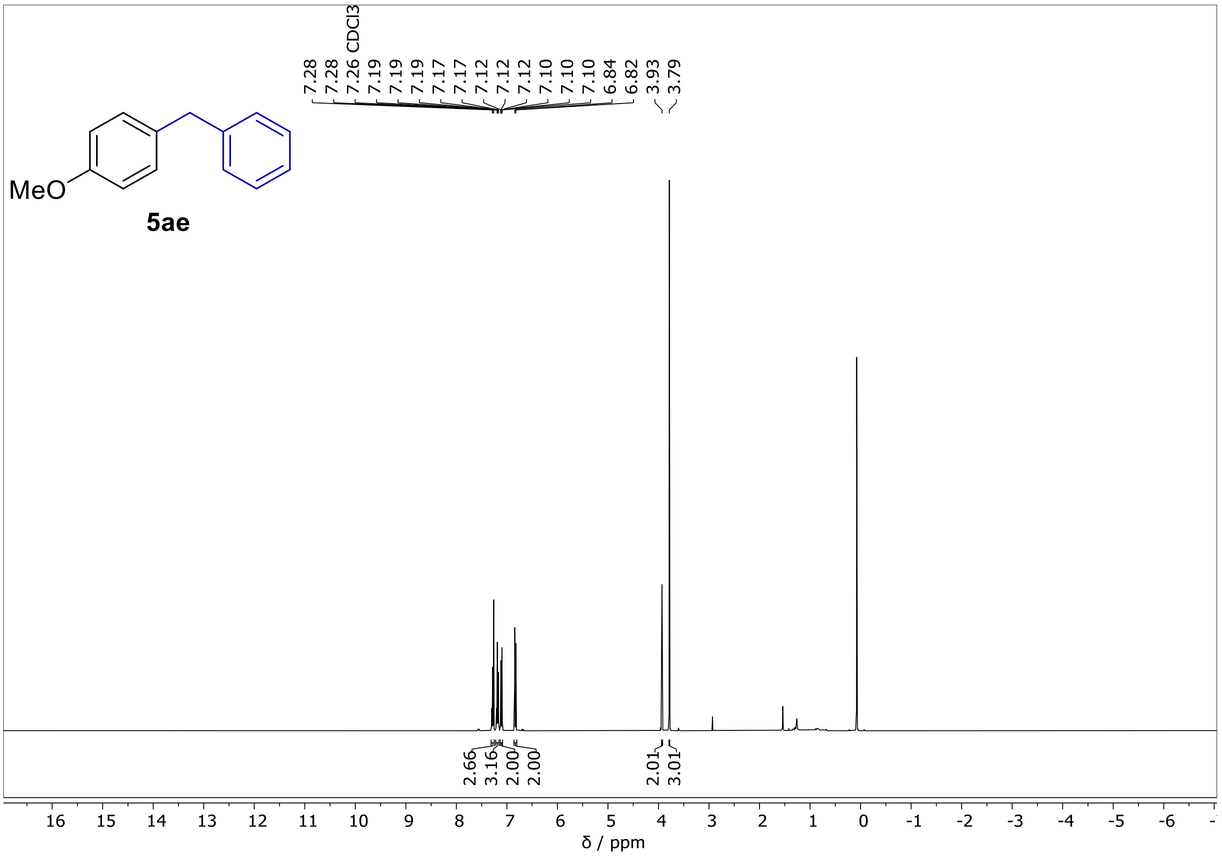

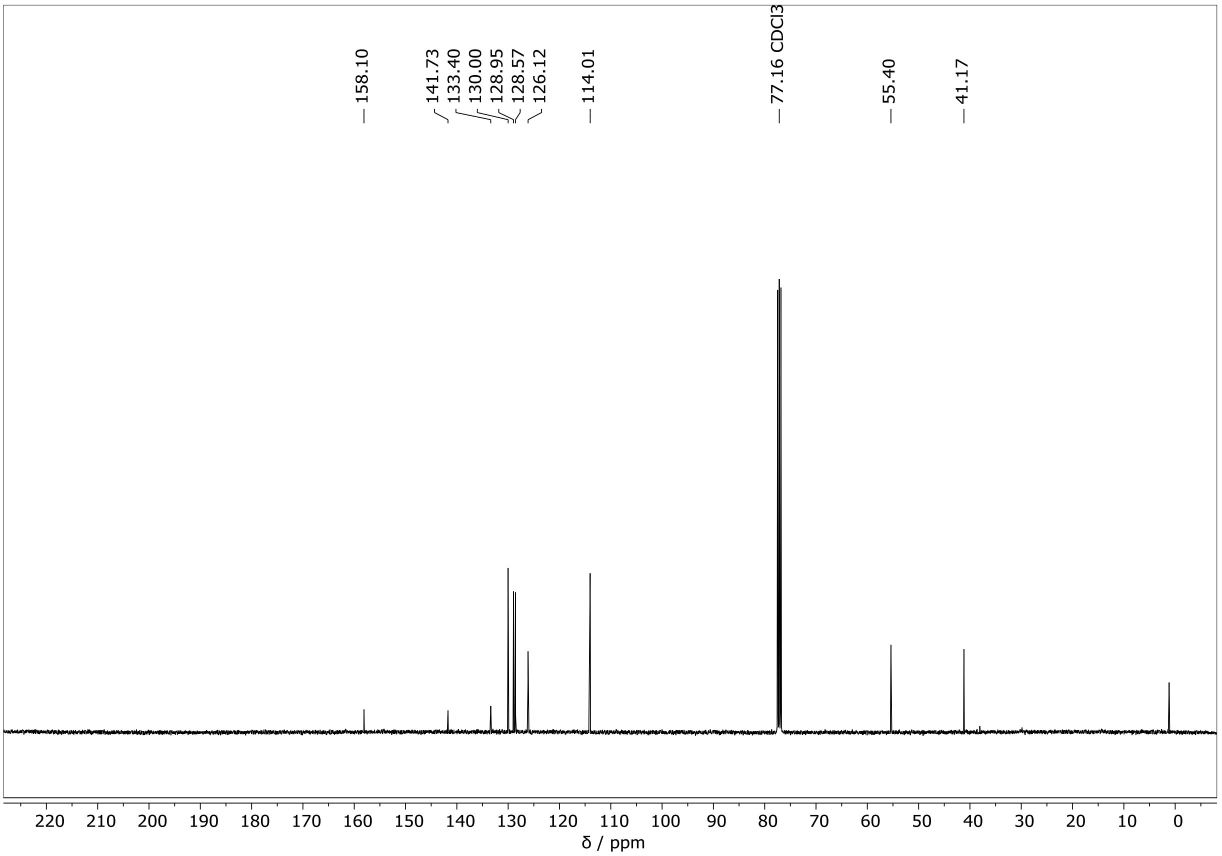

1-Methoxy-4-(6-methylhept-5-en-2-yl)benzene (5af)

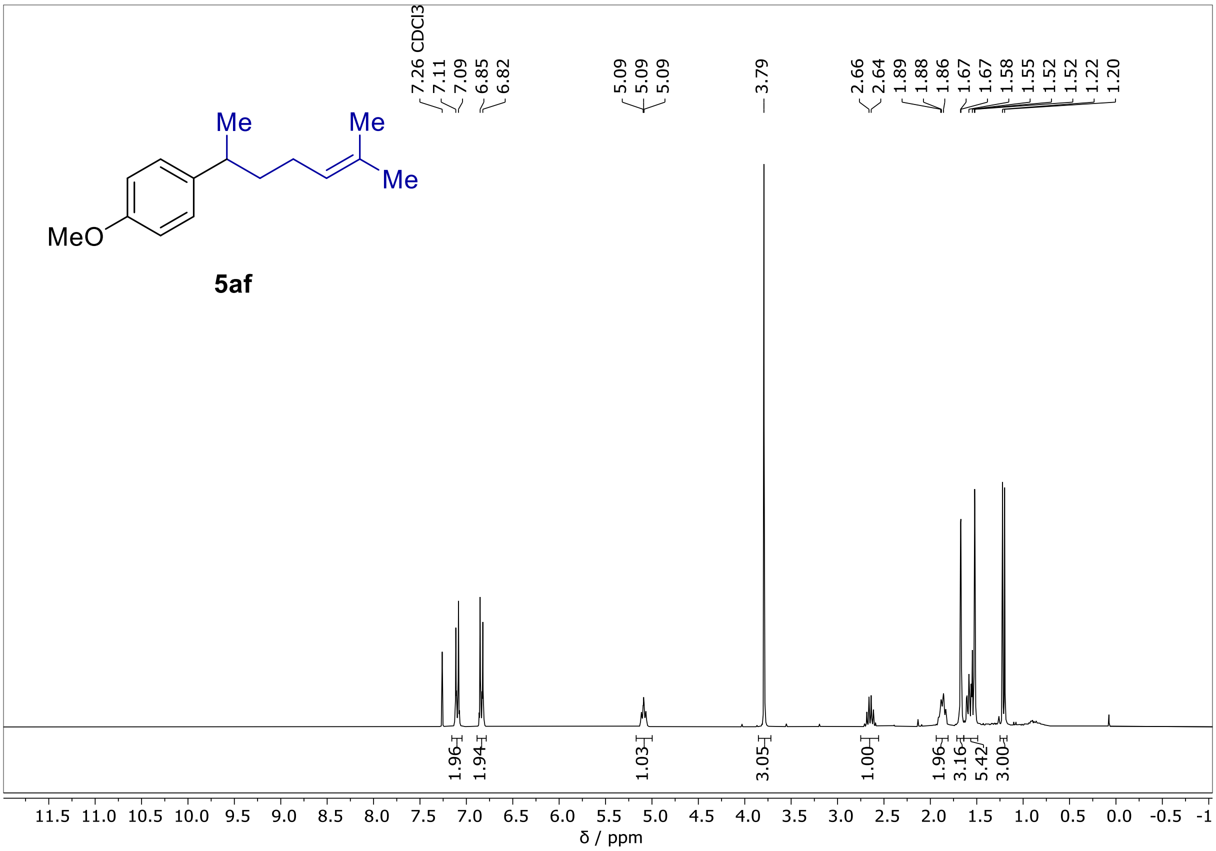

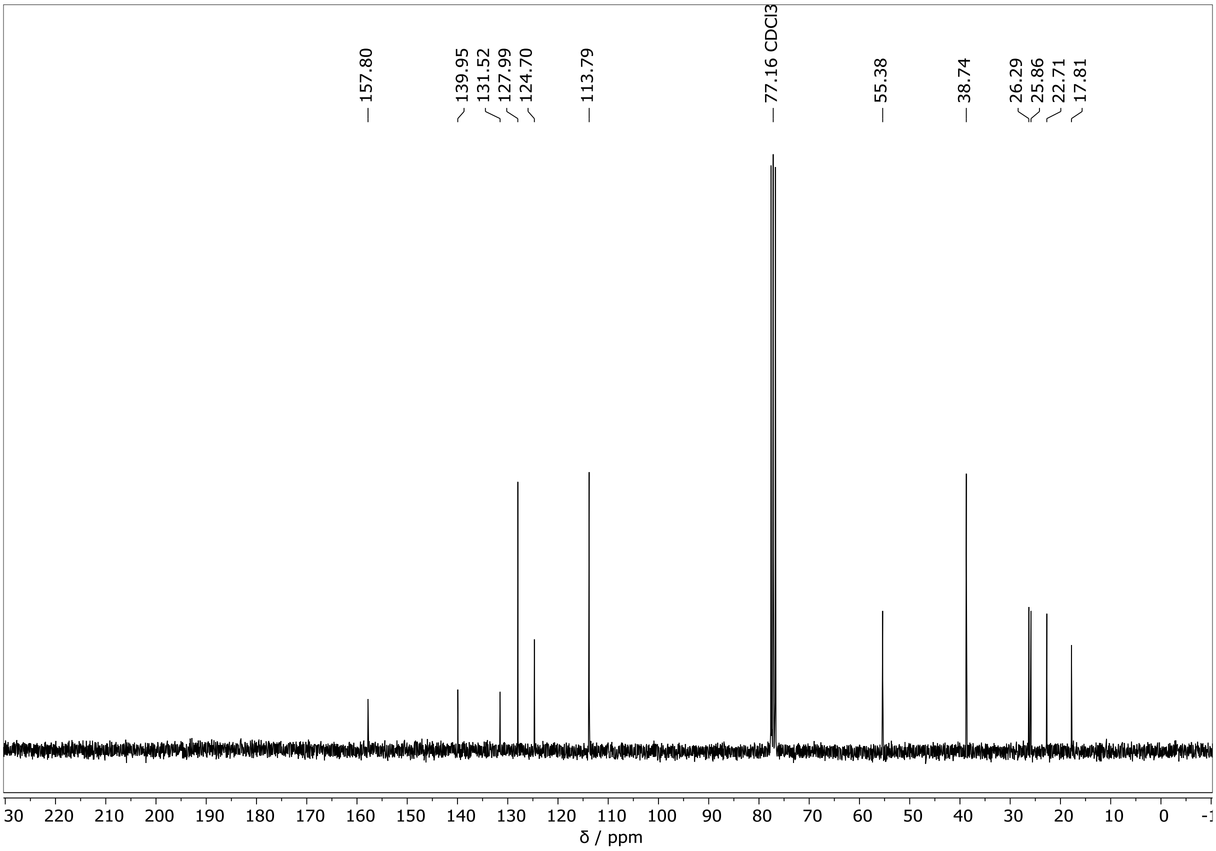

4-(4-Methoxyphenyl)-2,2-dimethyl-1,3-dioxolane (5ag)

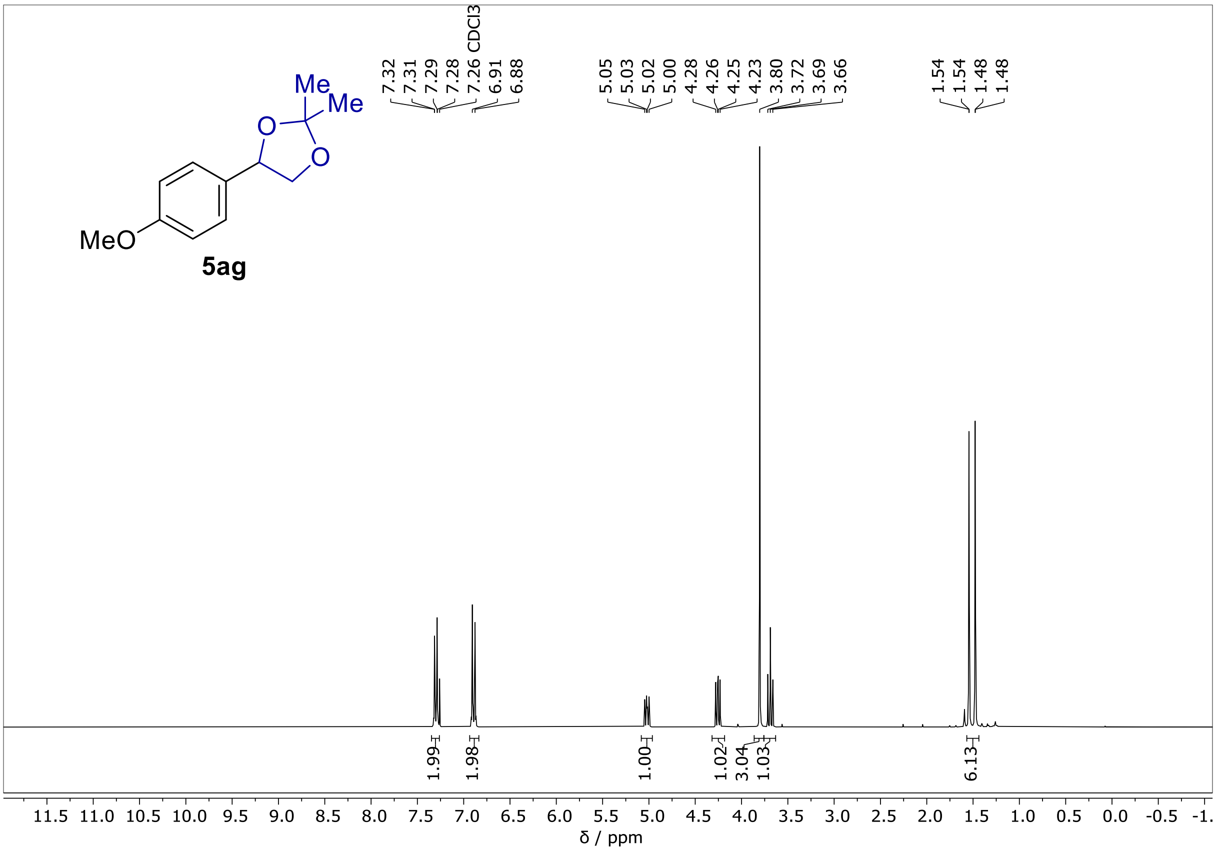

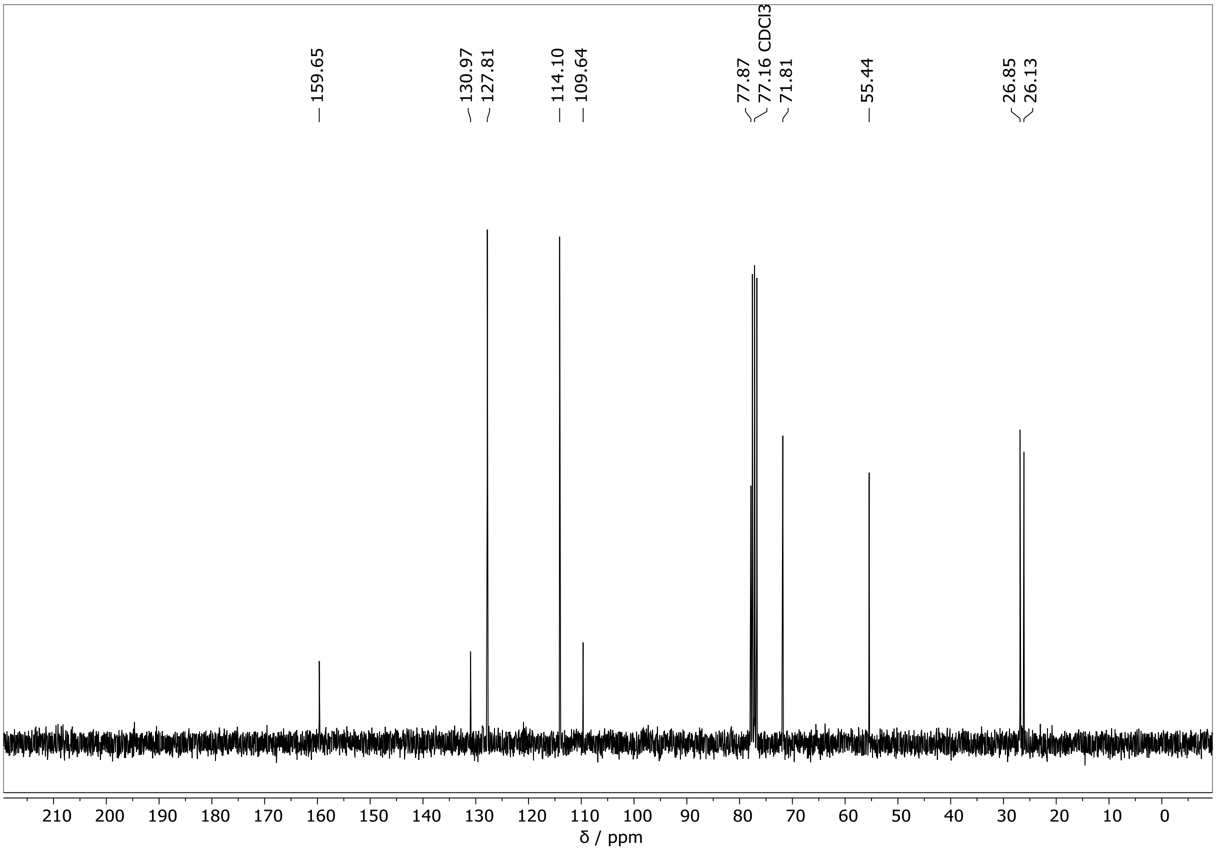

3-(4-Methoxyphenyl)tetrahydrofuran (5ah)

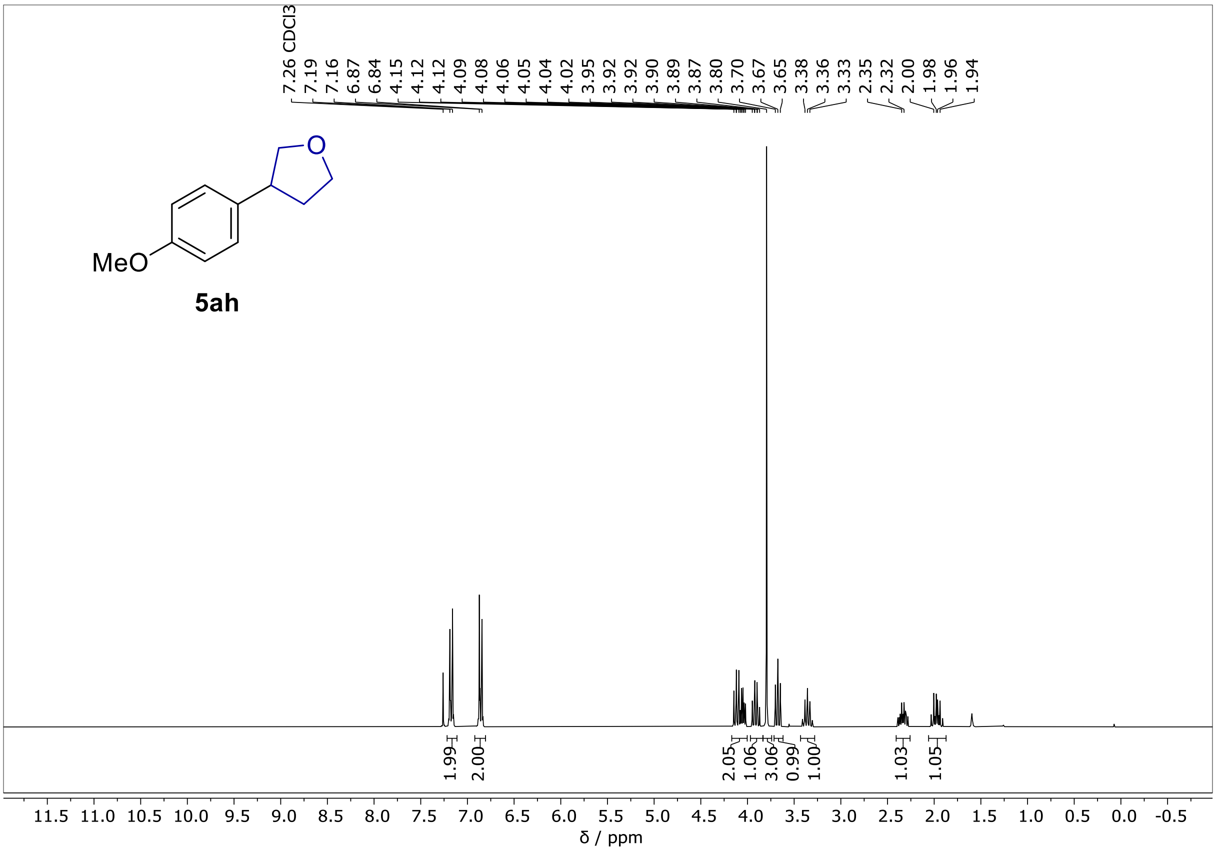

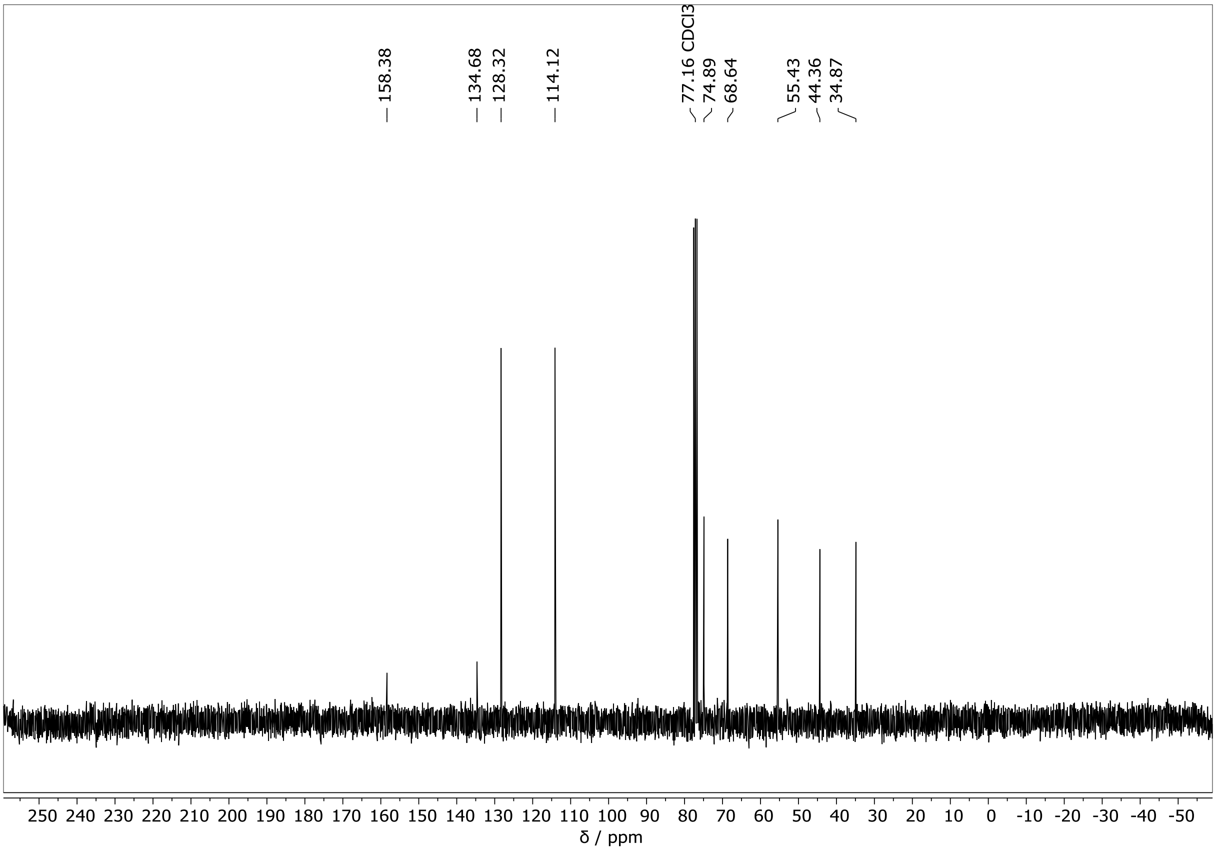

5-(4-Methoxyphenyl)-2,2,7,7-tetramethyltetrahydro-5H-bis([1,3]dioxolo)[4,5-b:4',5'-d]pyran (5ai)

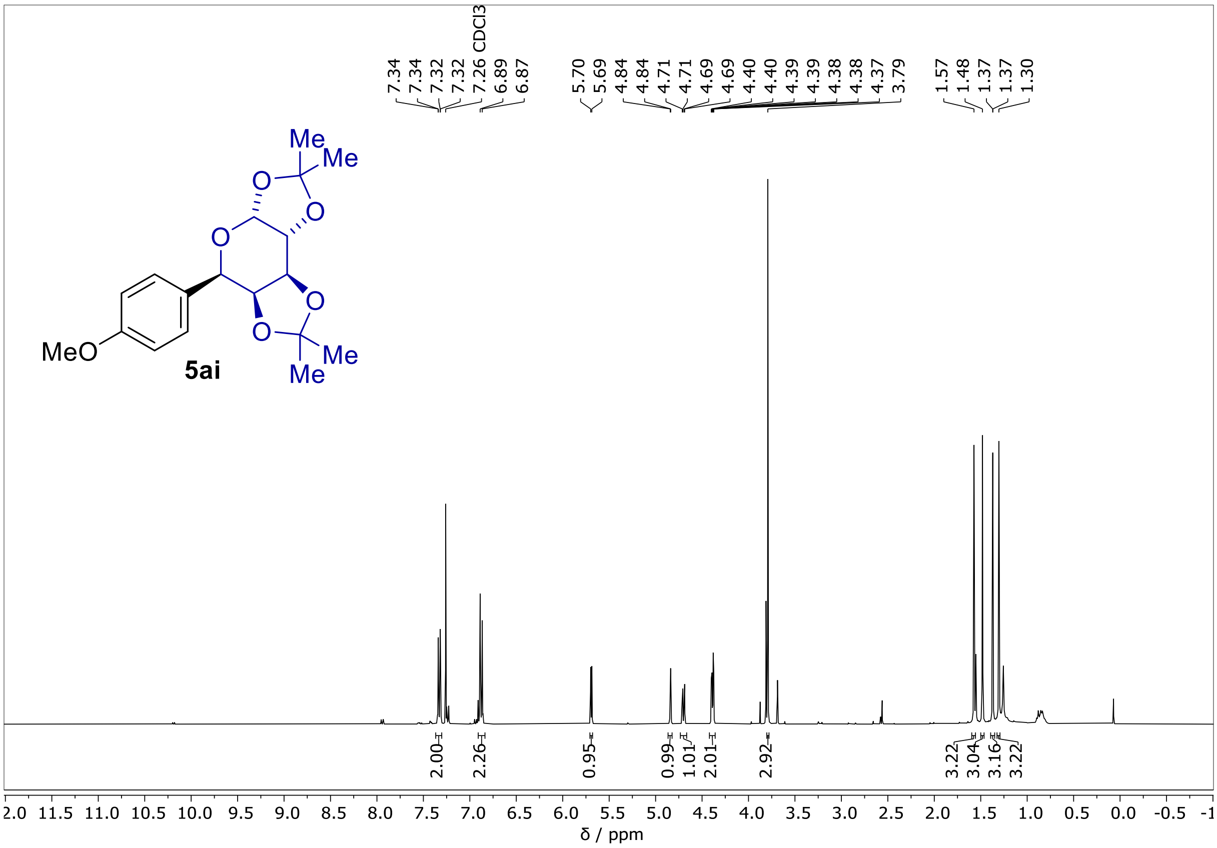

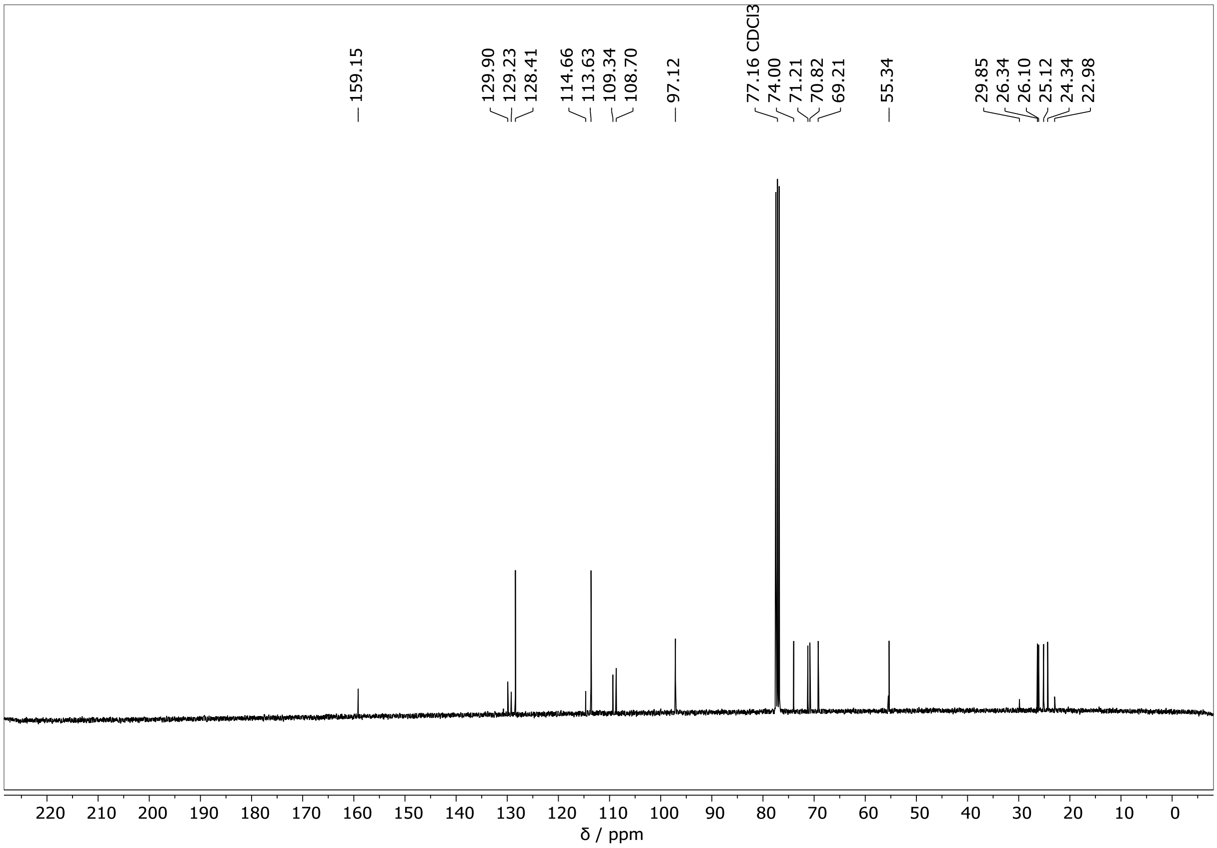

***N*-alkyl amines**

***N*-Cyclohexylaniline (7a)**

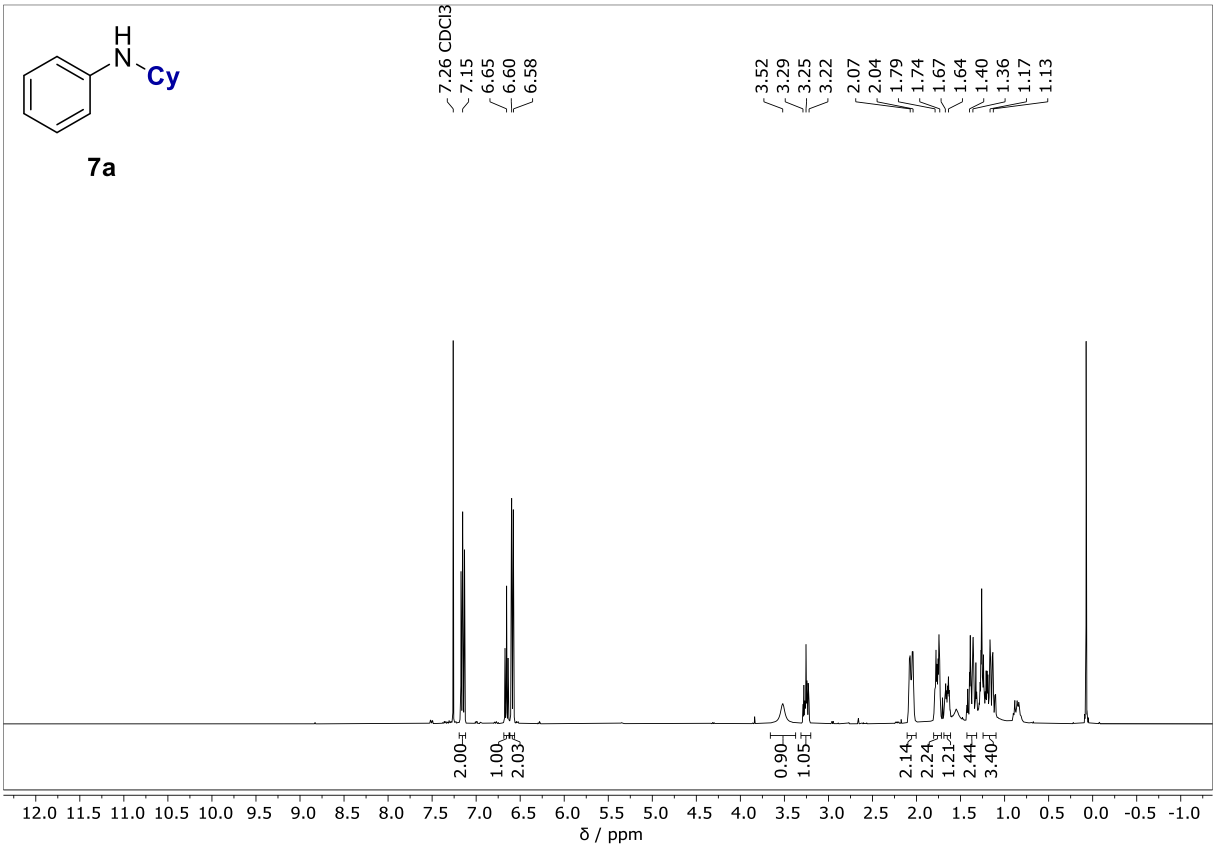

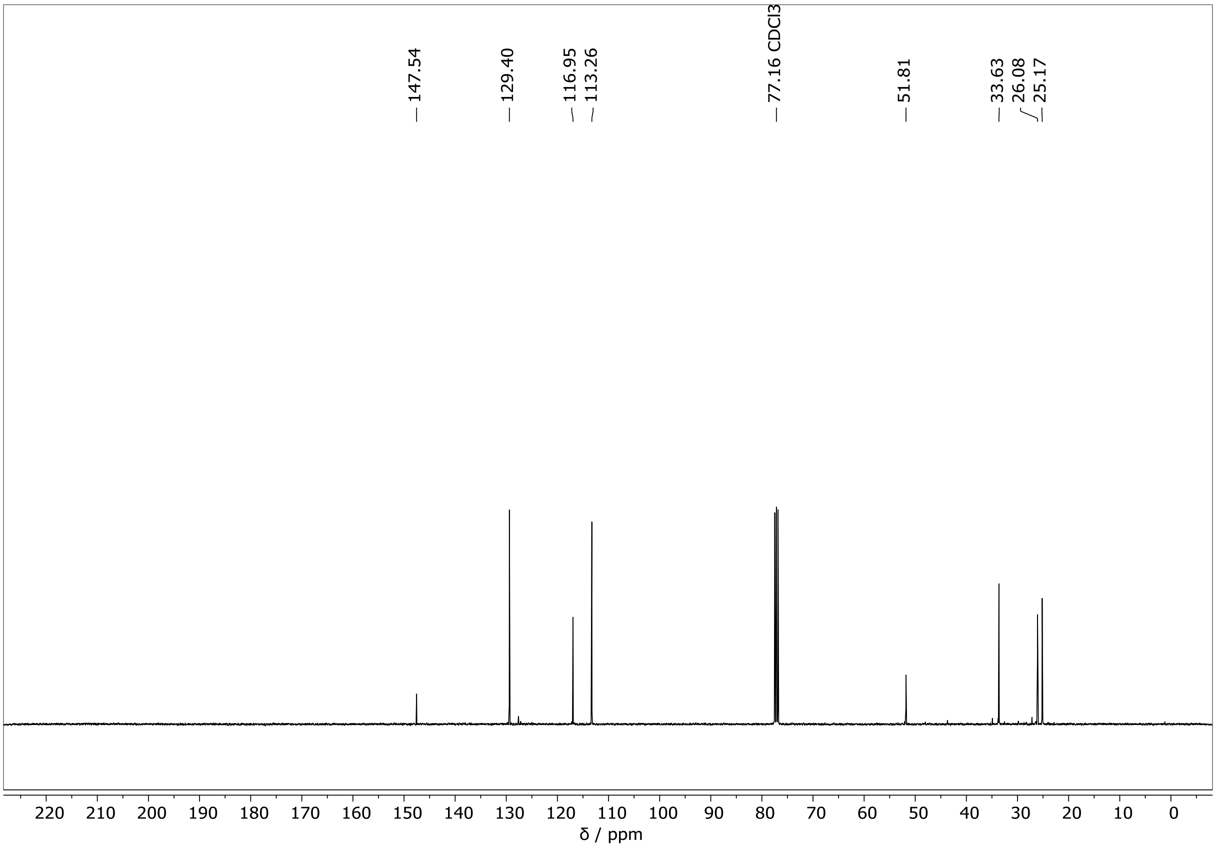

_N_-Cyclohexyl-2-methoxyaniline (7b)

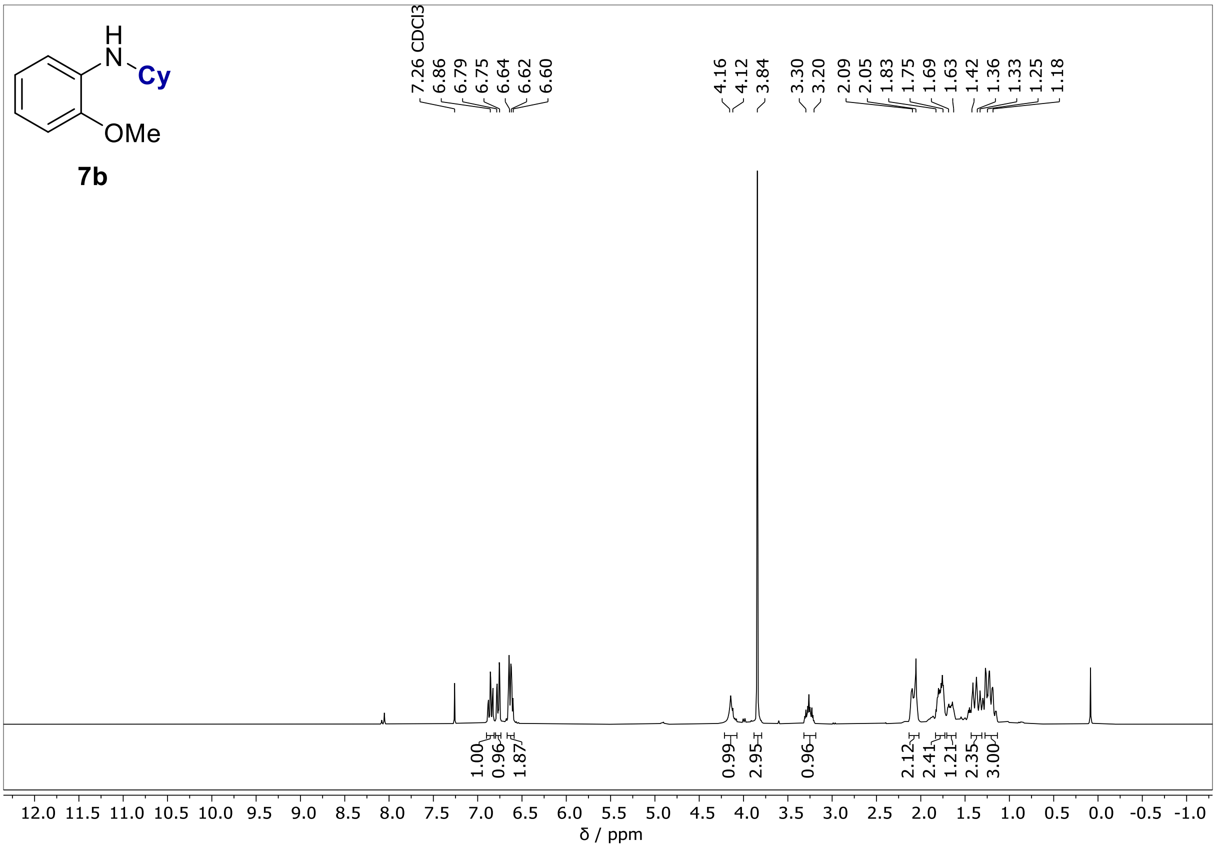

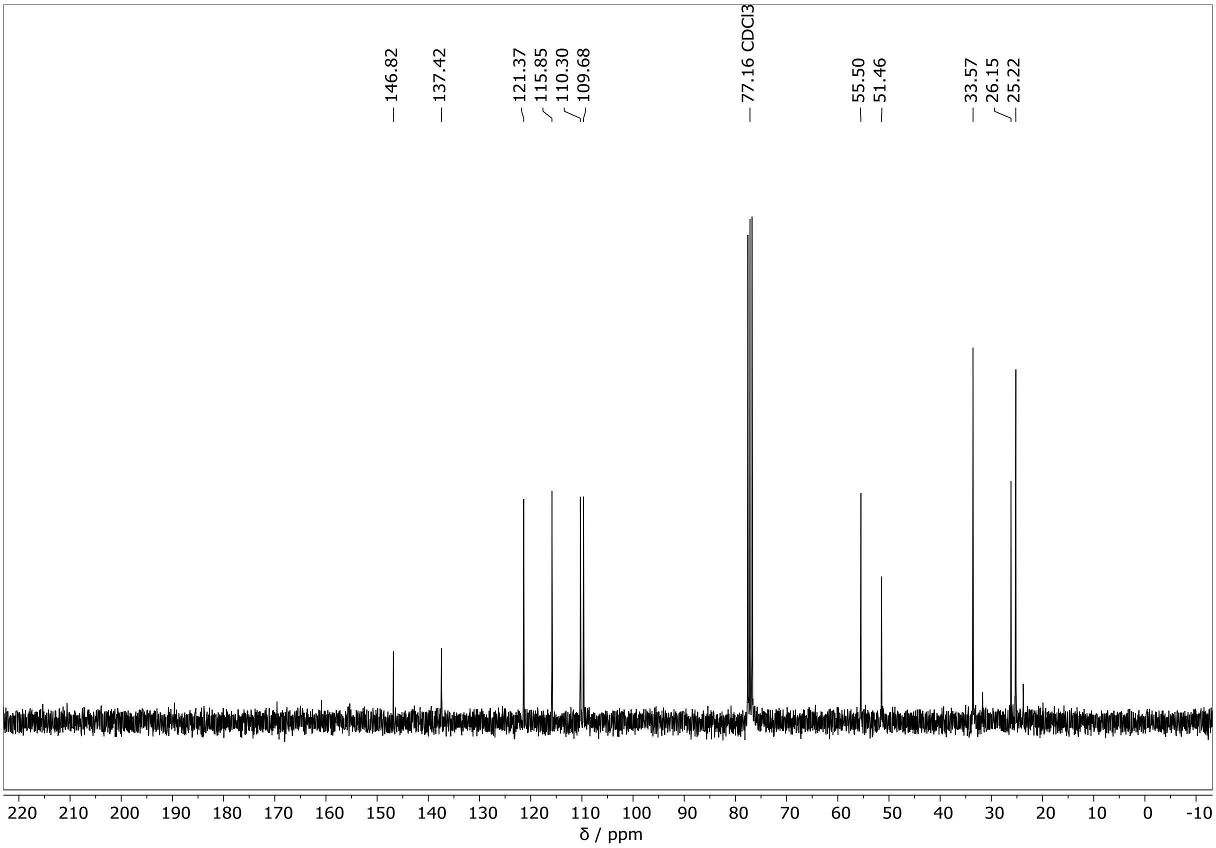

4-Bromo-*N*-cyclohexylaniline (7c)

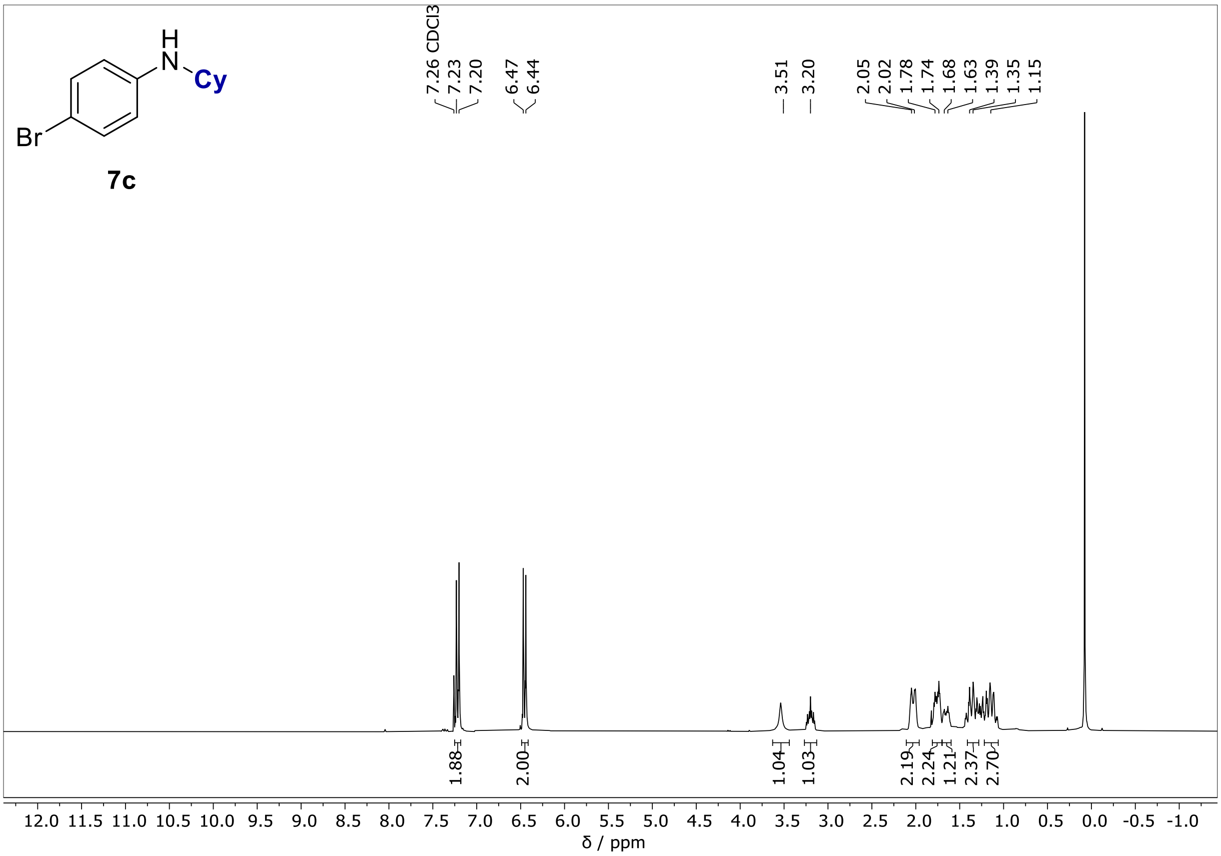

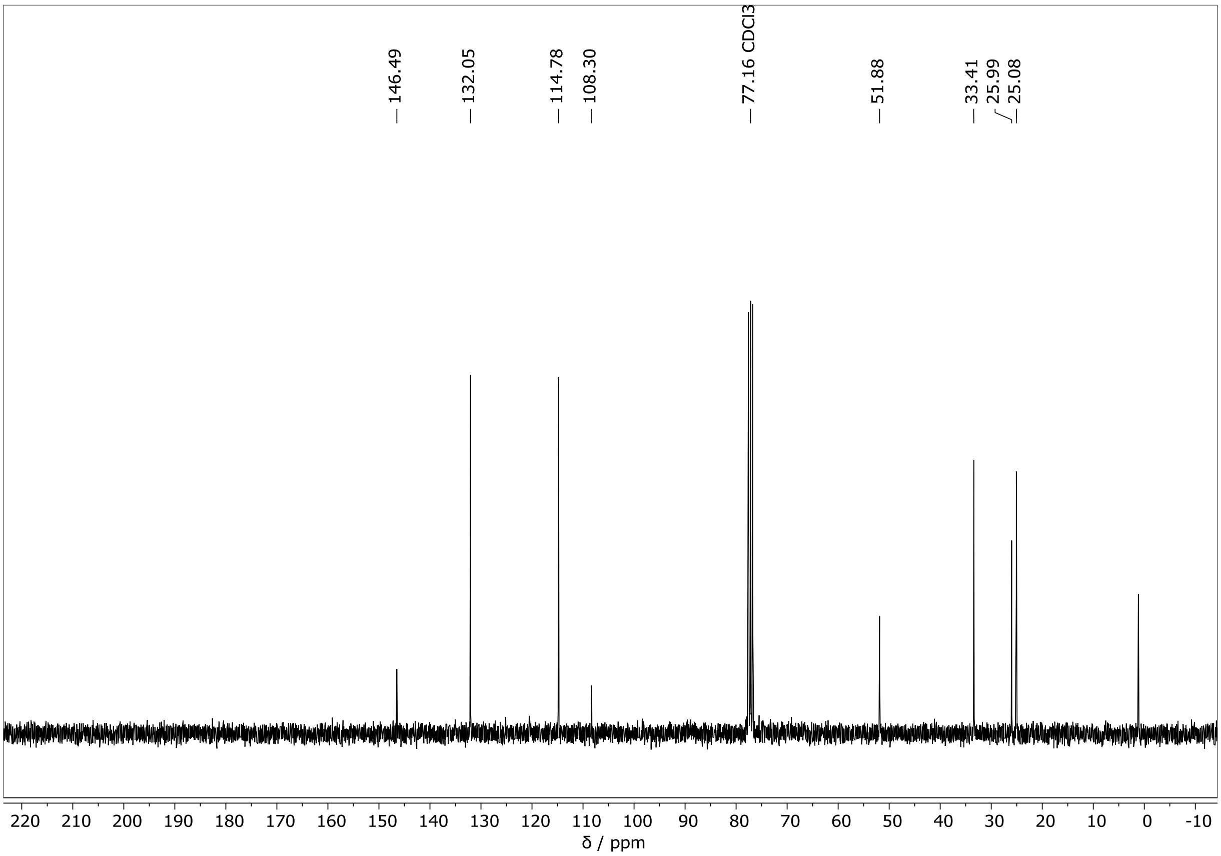

N-Cyclohexyl-3-methylaniline (7d)

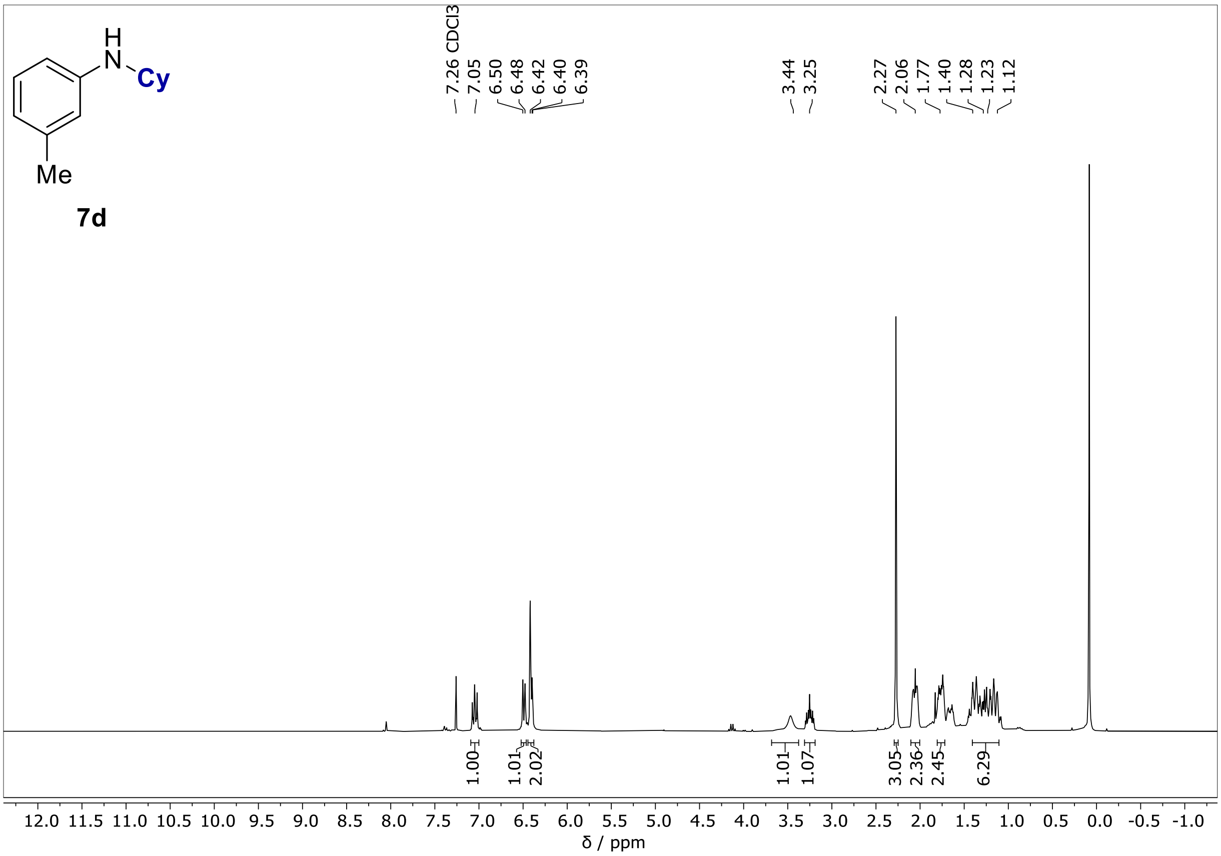

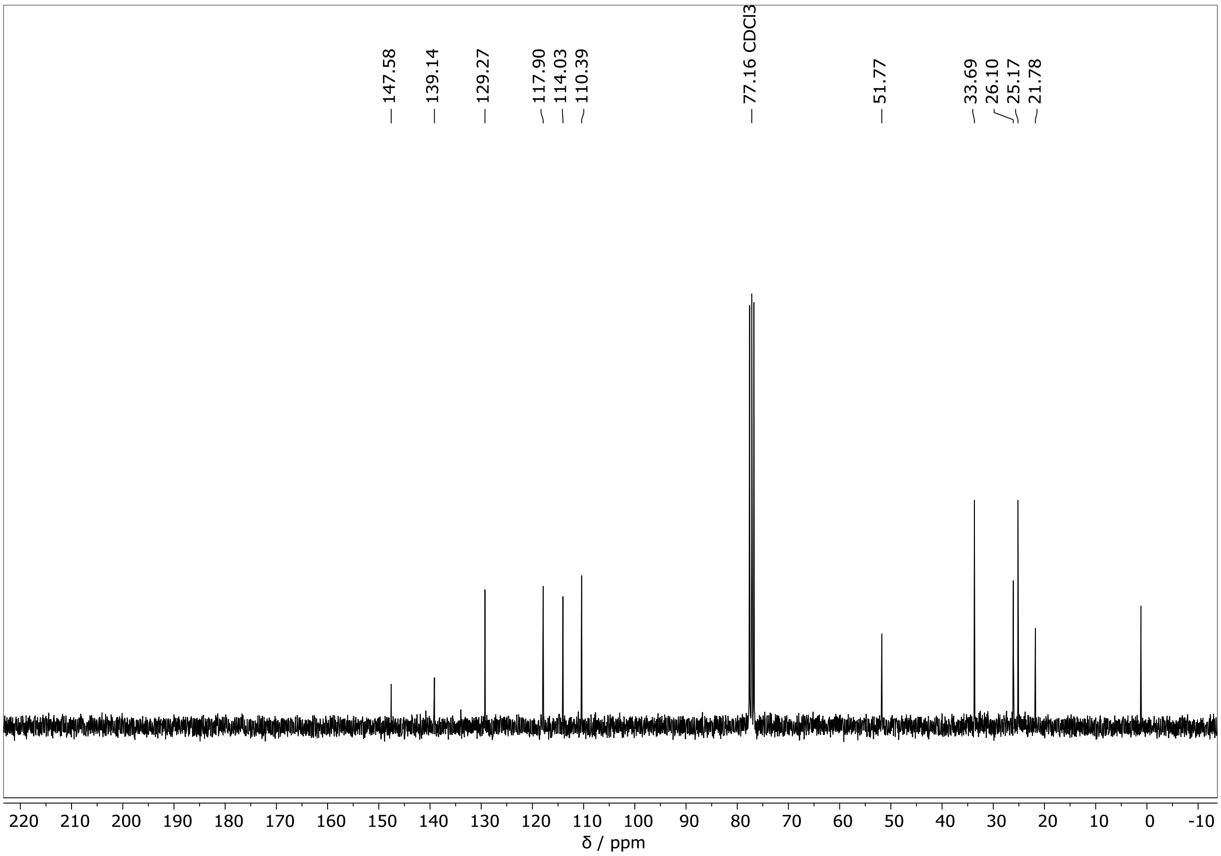

5-Bromo-*N*-cyclohexyl-2-fluoroaniline (7e)

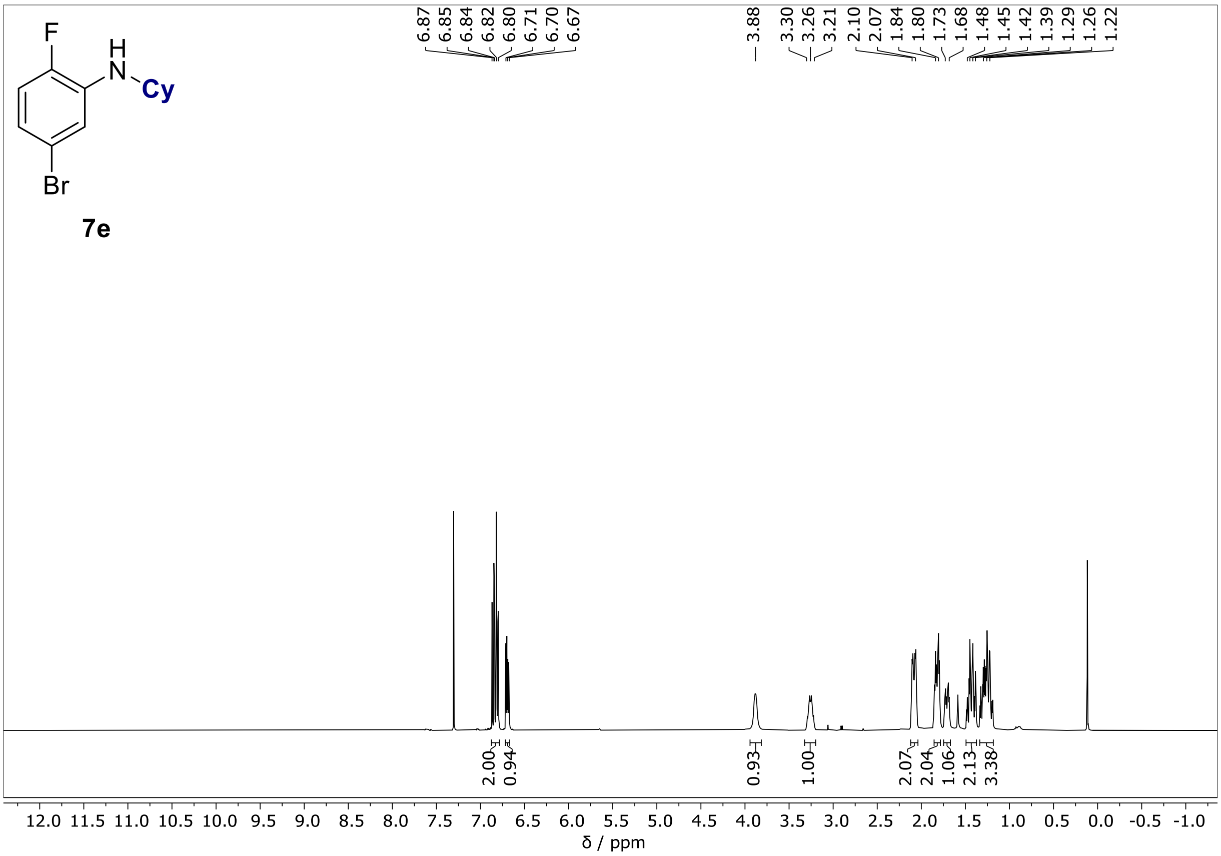

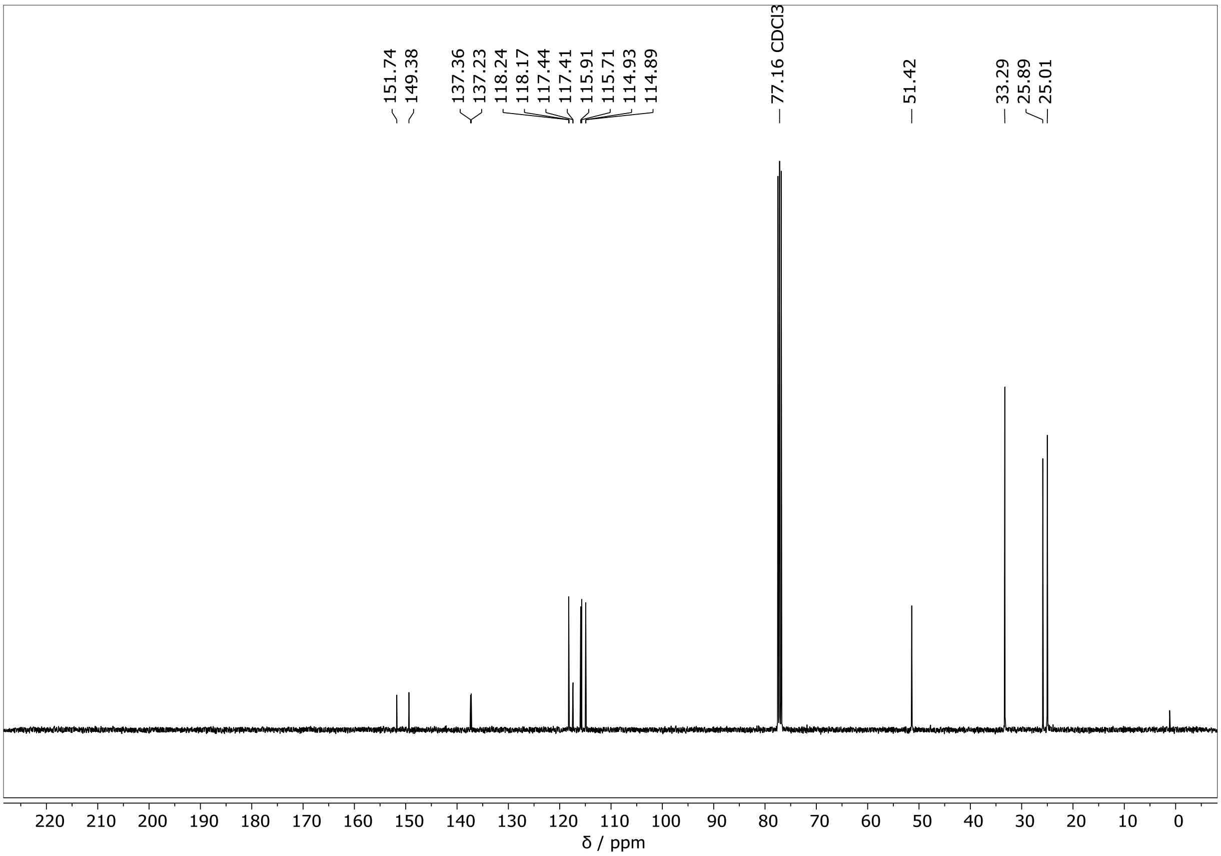

2-Bromo-*N*-cyclohexylaniline (7f)

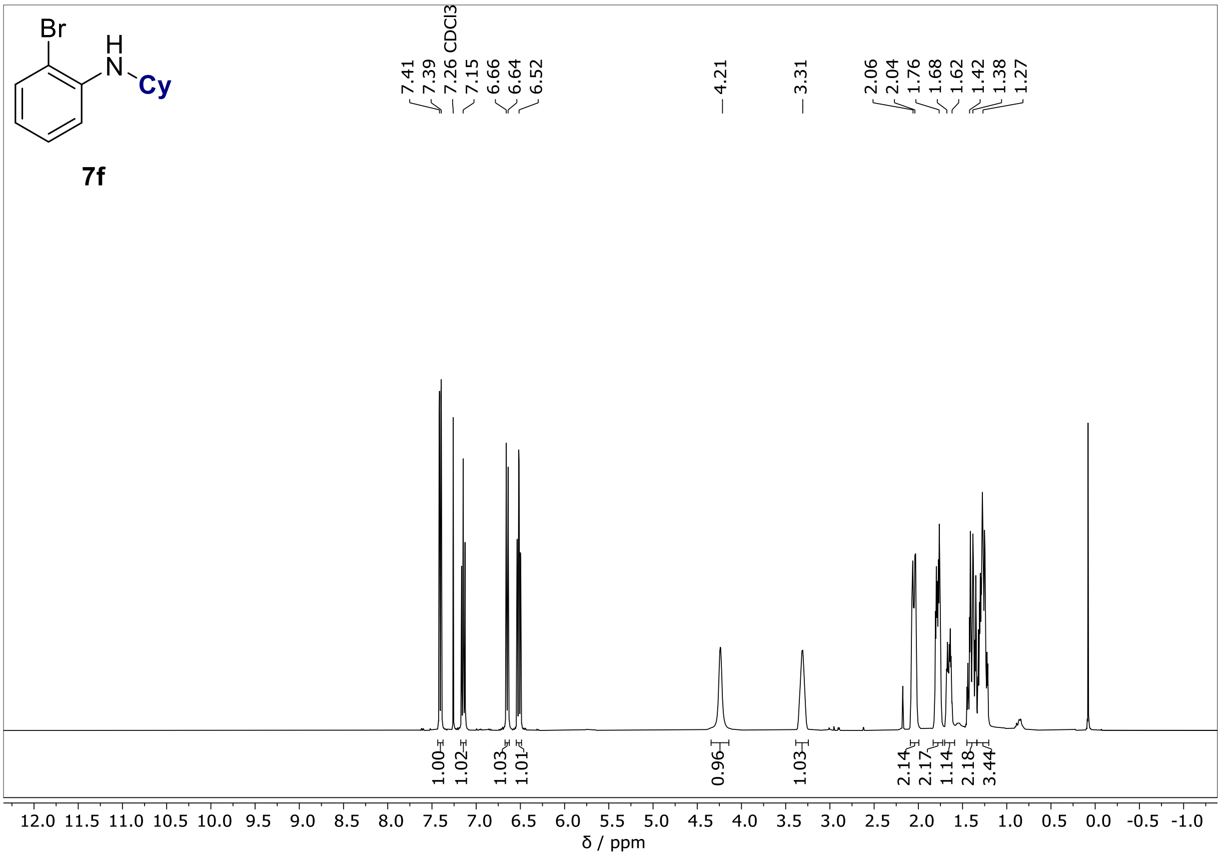

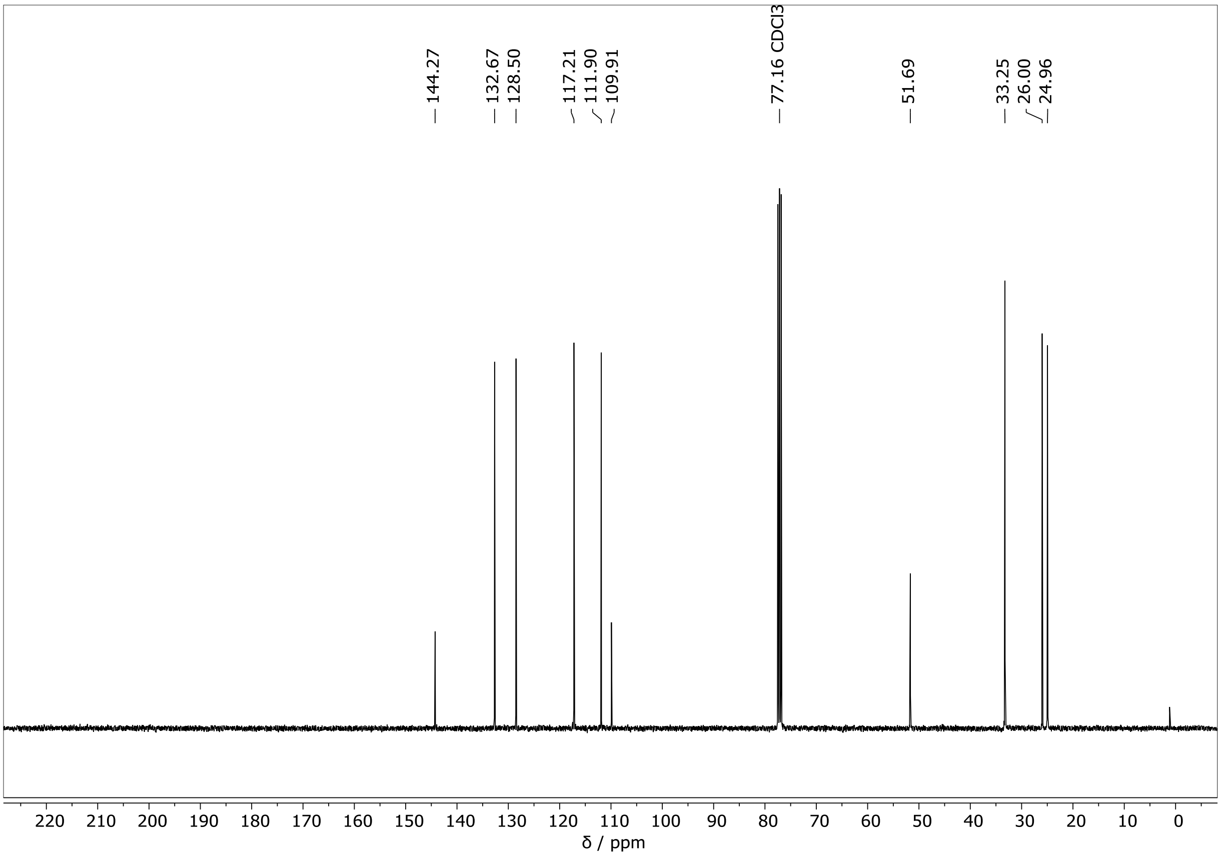

2,5-Dibromo-*N*-cyclohexylaniline (7g)

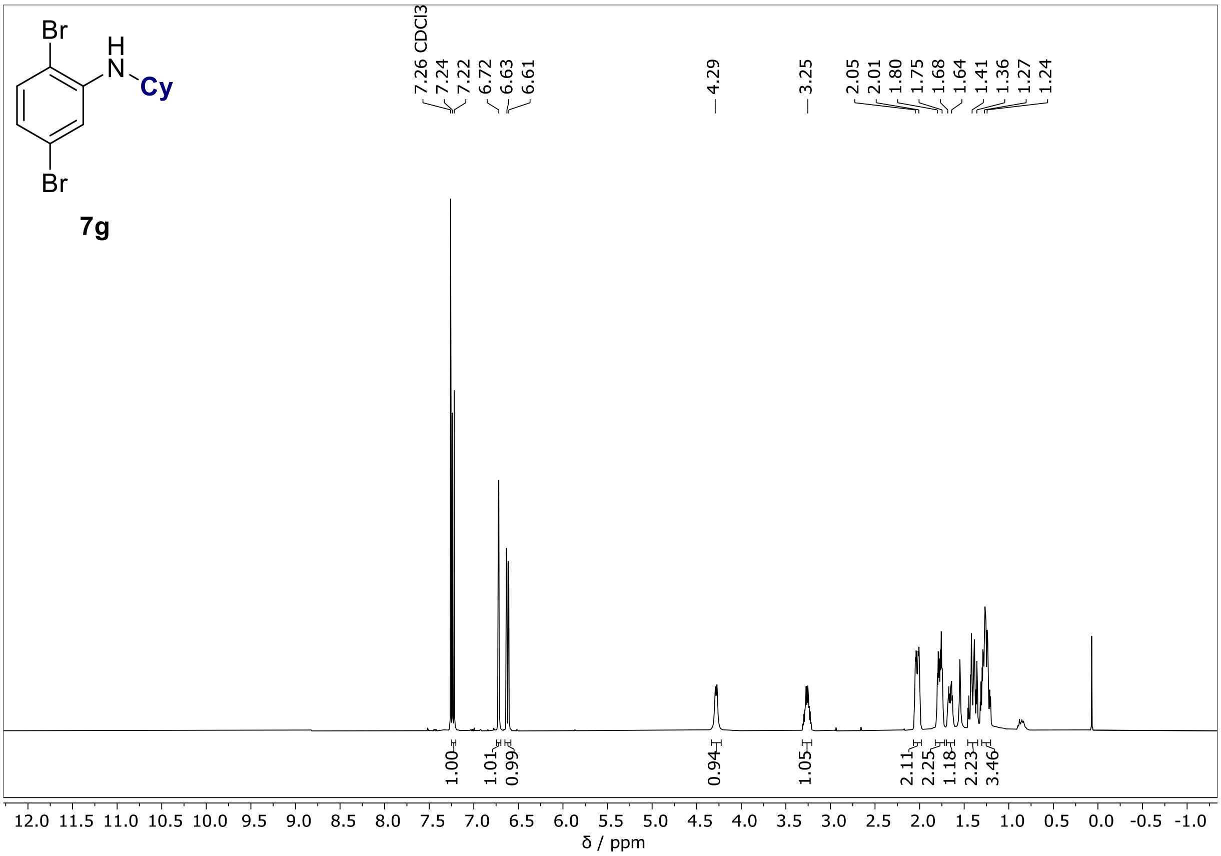

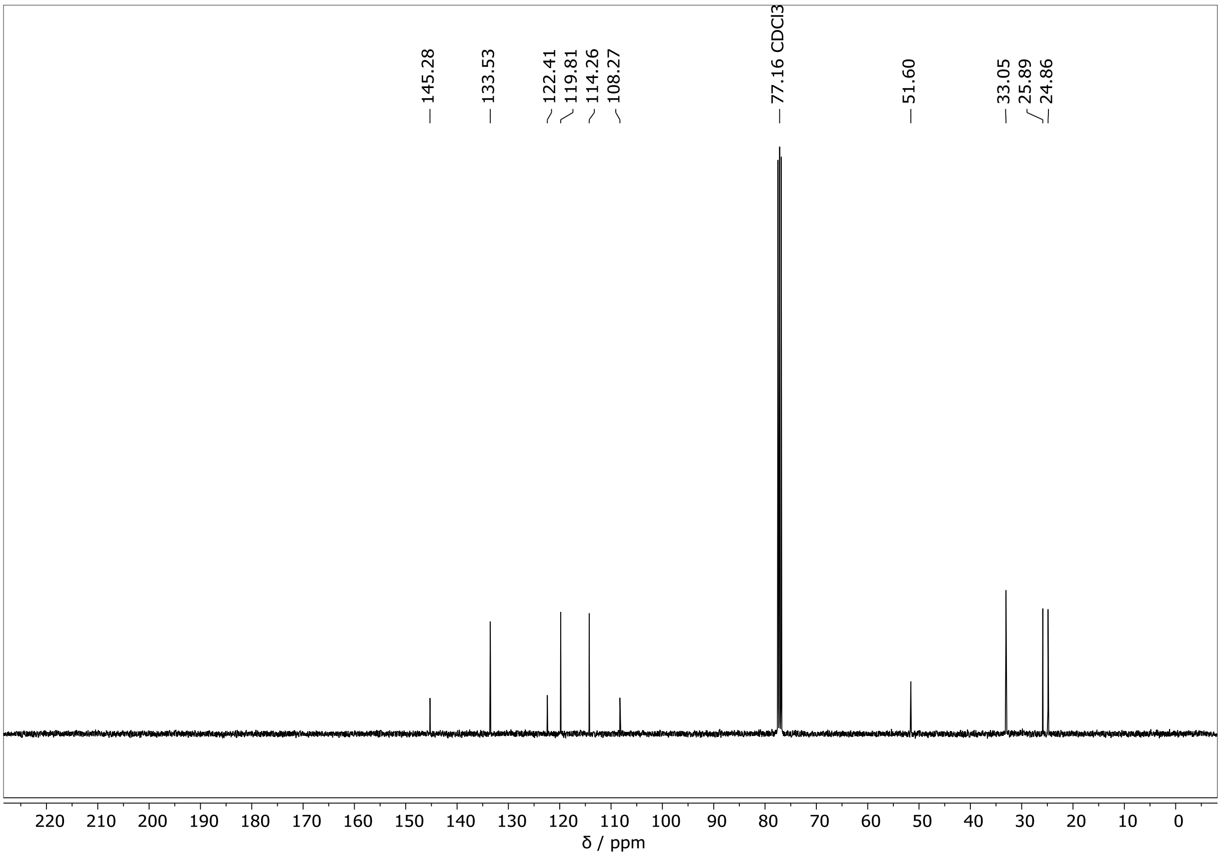

***N*-cyclohexyl-4-methylaniline (7h)**

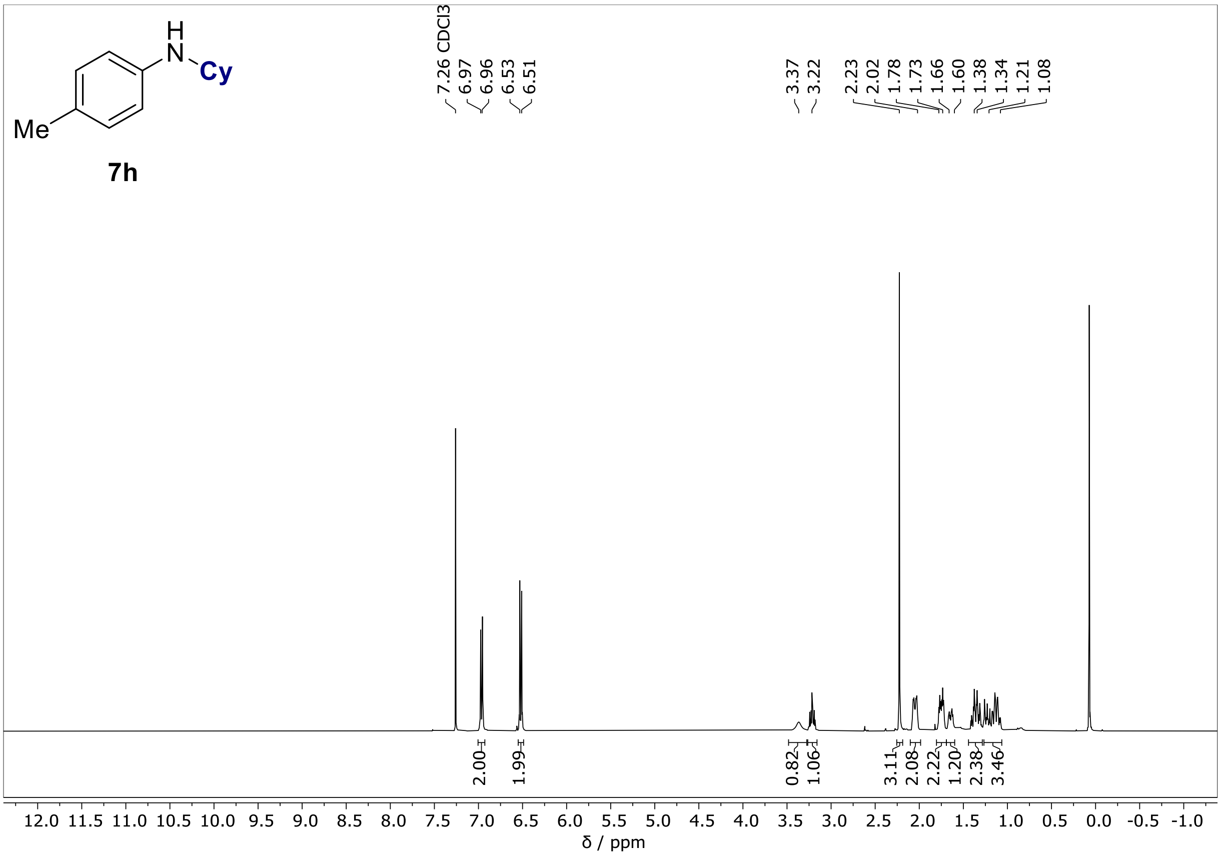

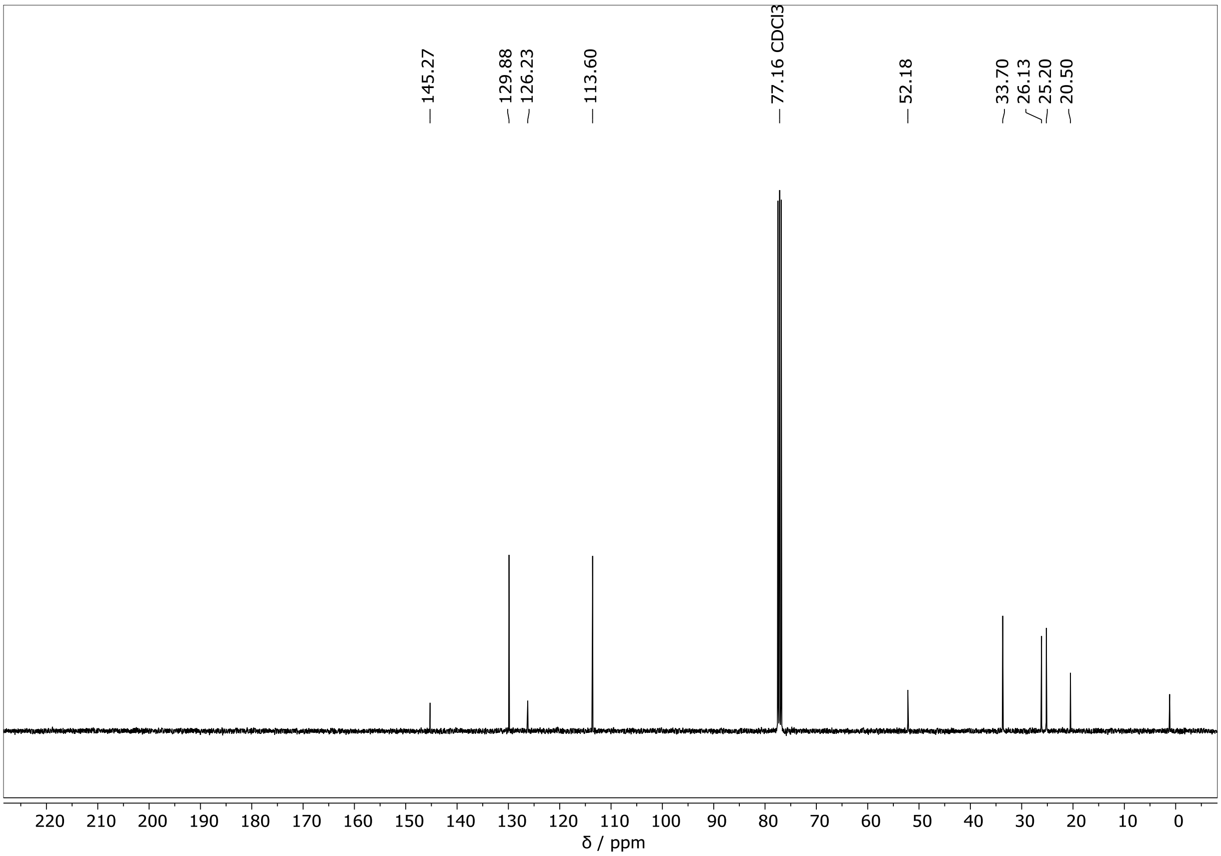

N-cyclohexyl-2-methoxy-4-methylaniline (7i)

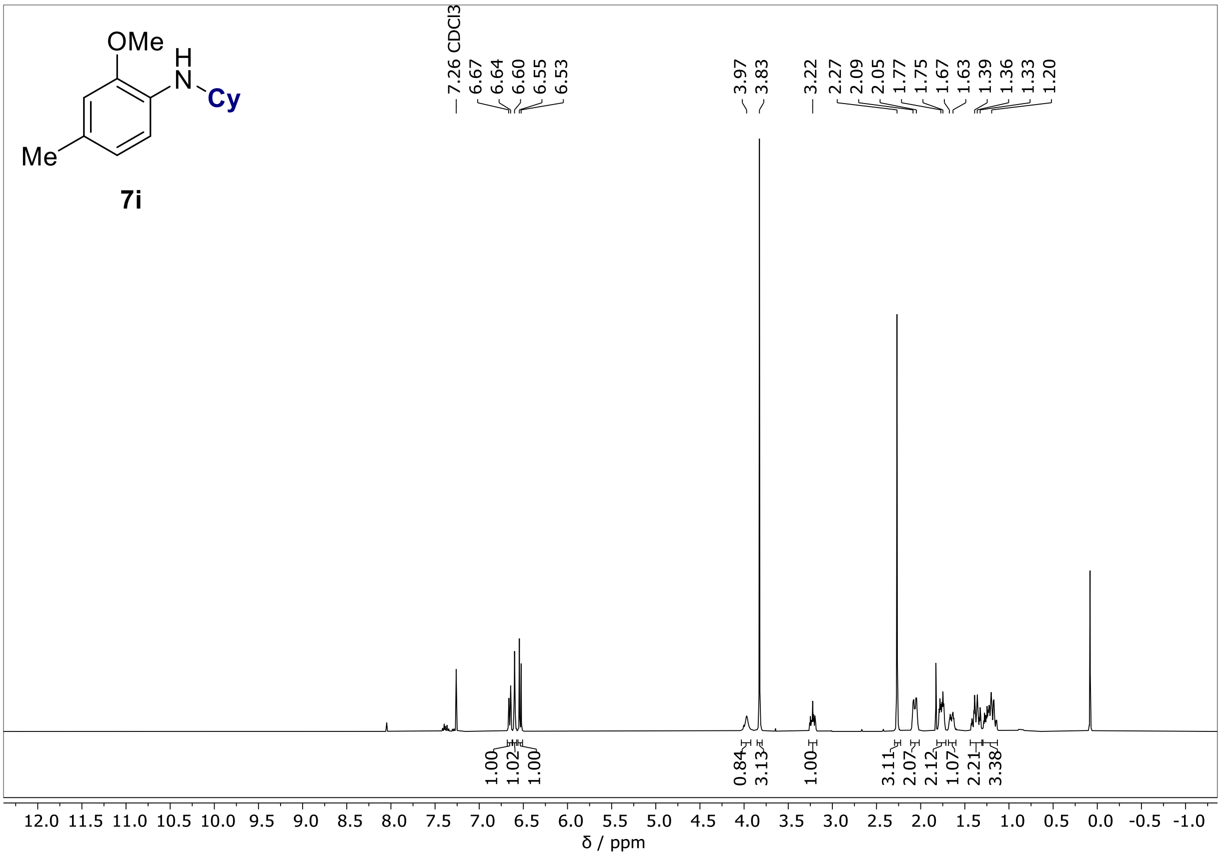

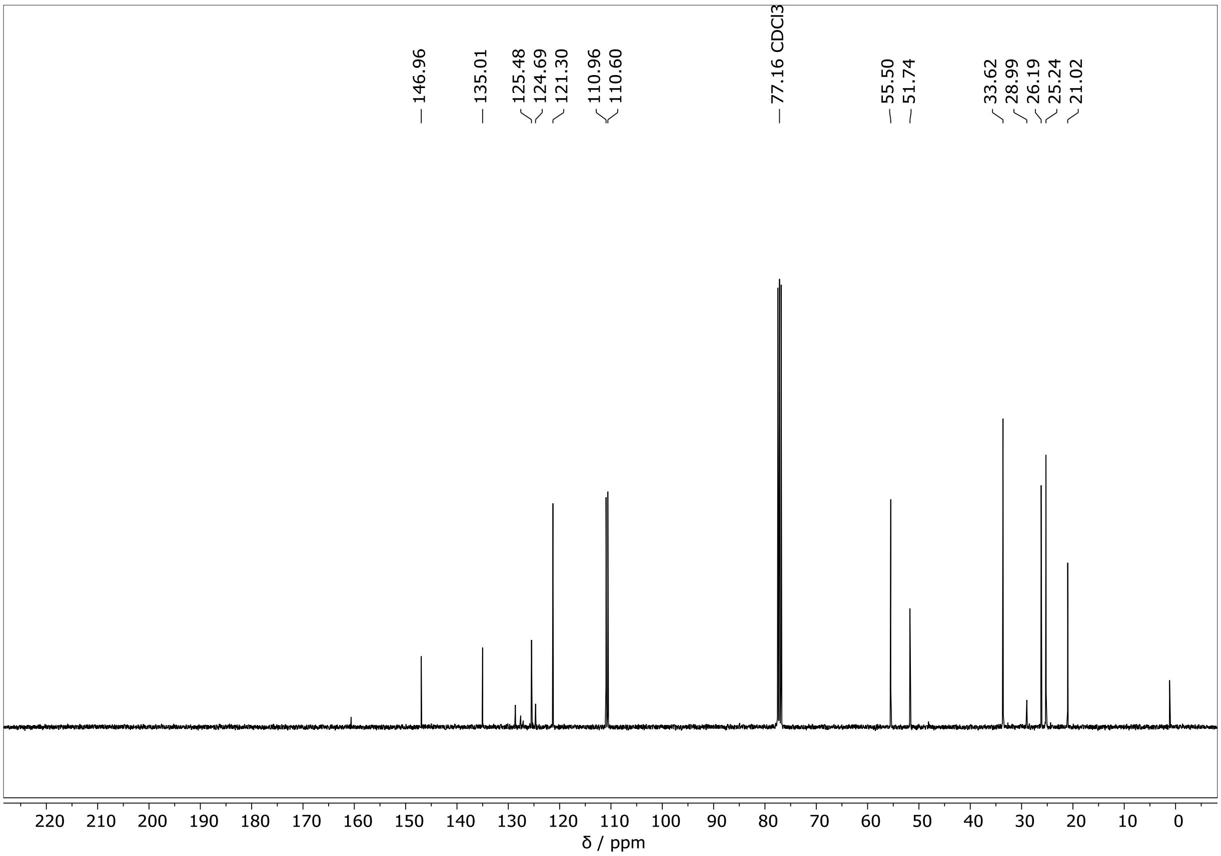

N-(1-(benzo[*d*][1,3]dioxol-5-yl)propan-2-yl)aniline (7j)

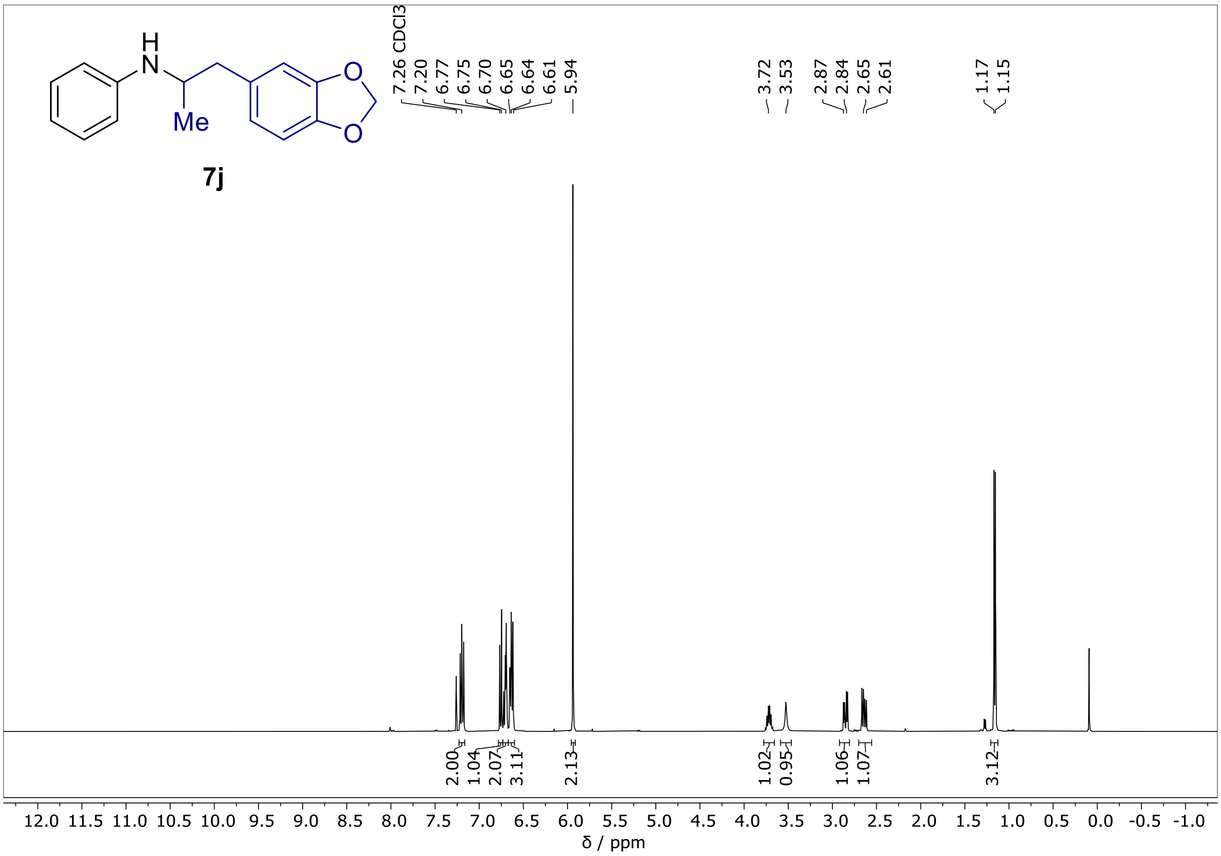

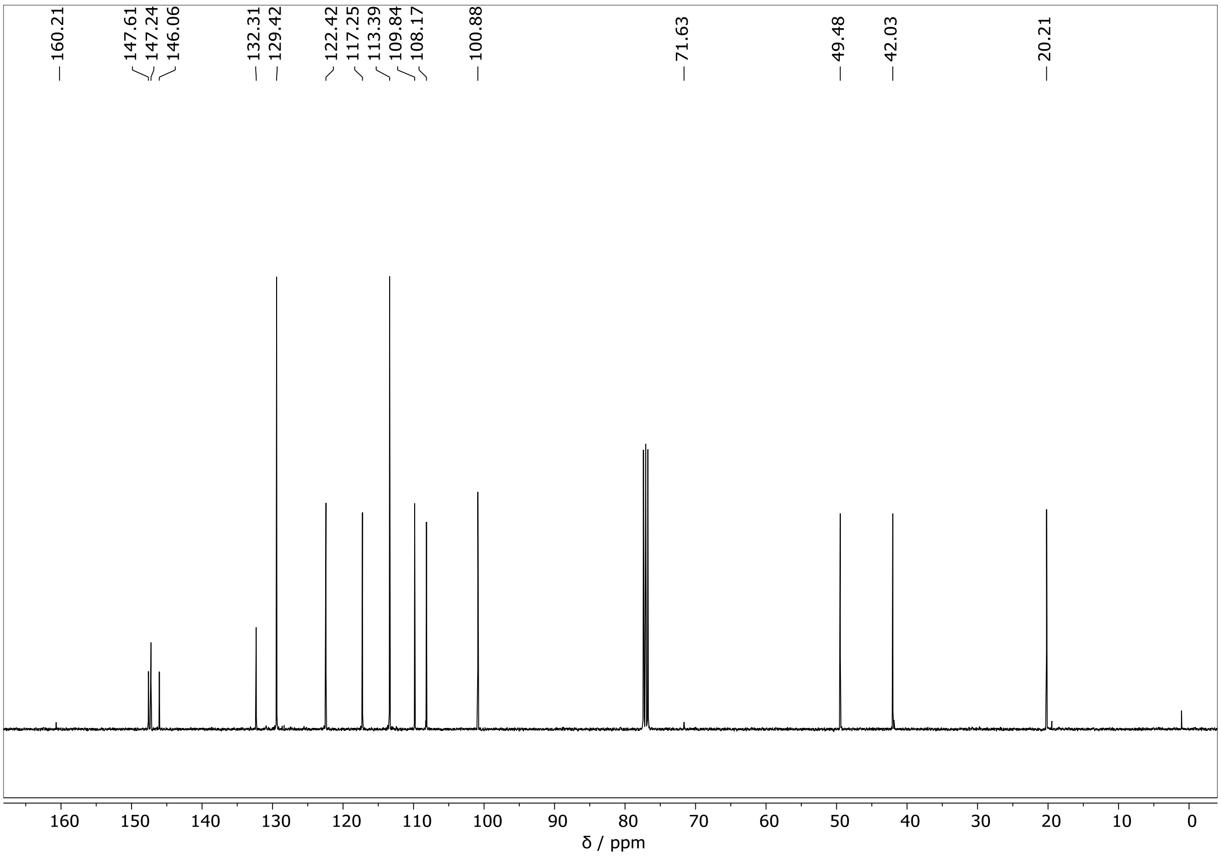

Thioethers

Cyclohexyl(phenyl)sulfane (9a)

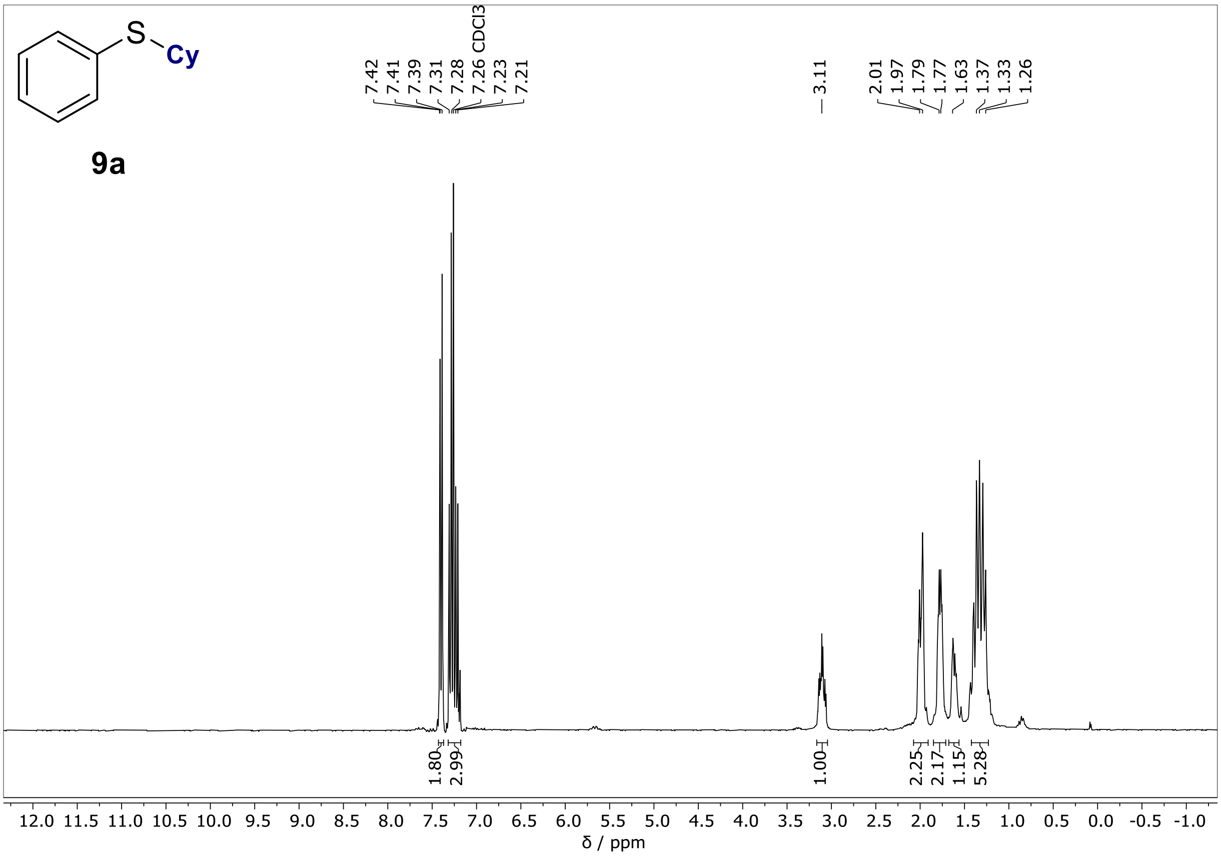

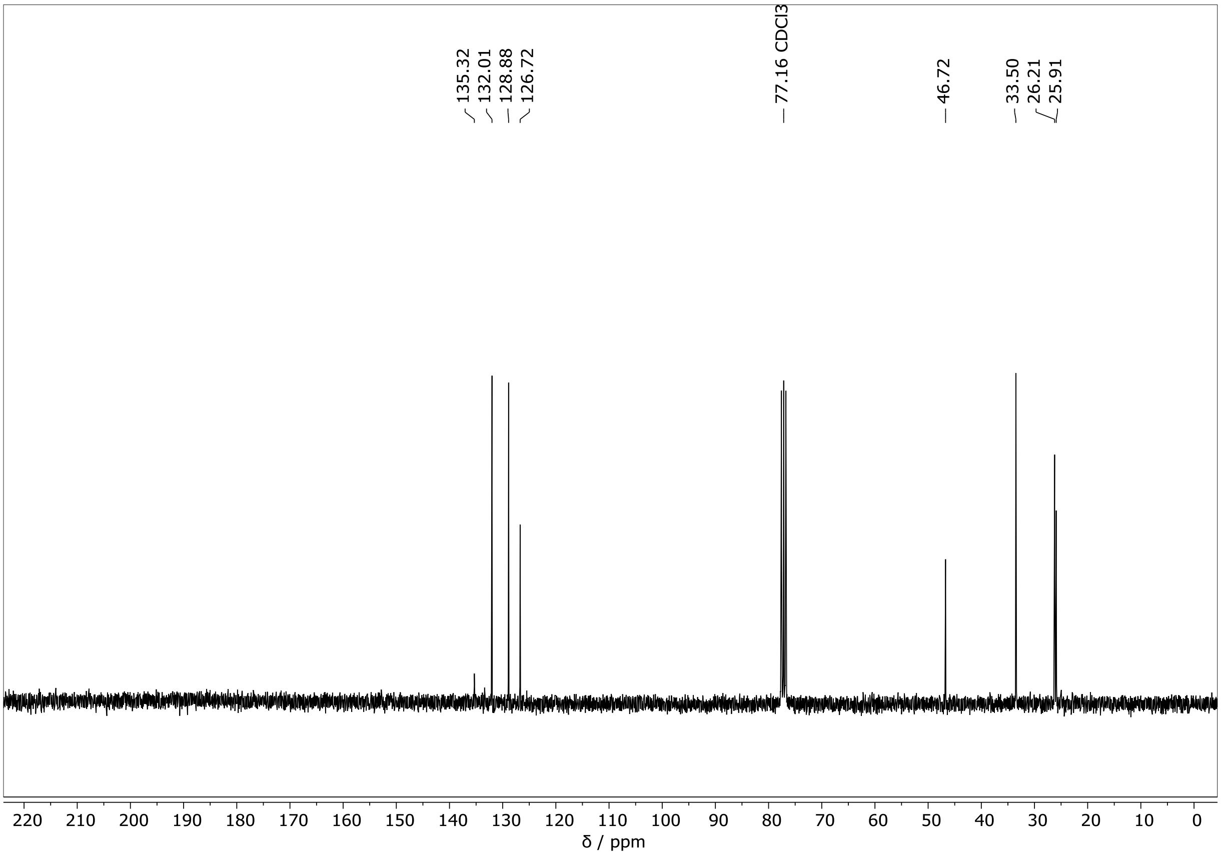

(4-Bromophenyl)(cyclohexyl)sulfane (9b)

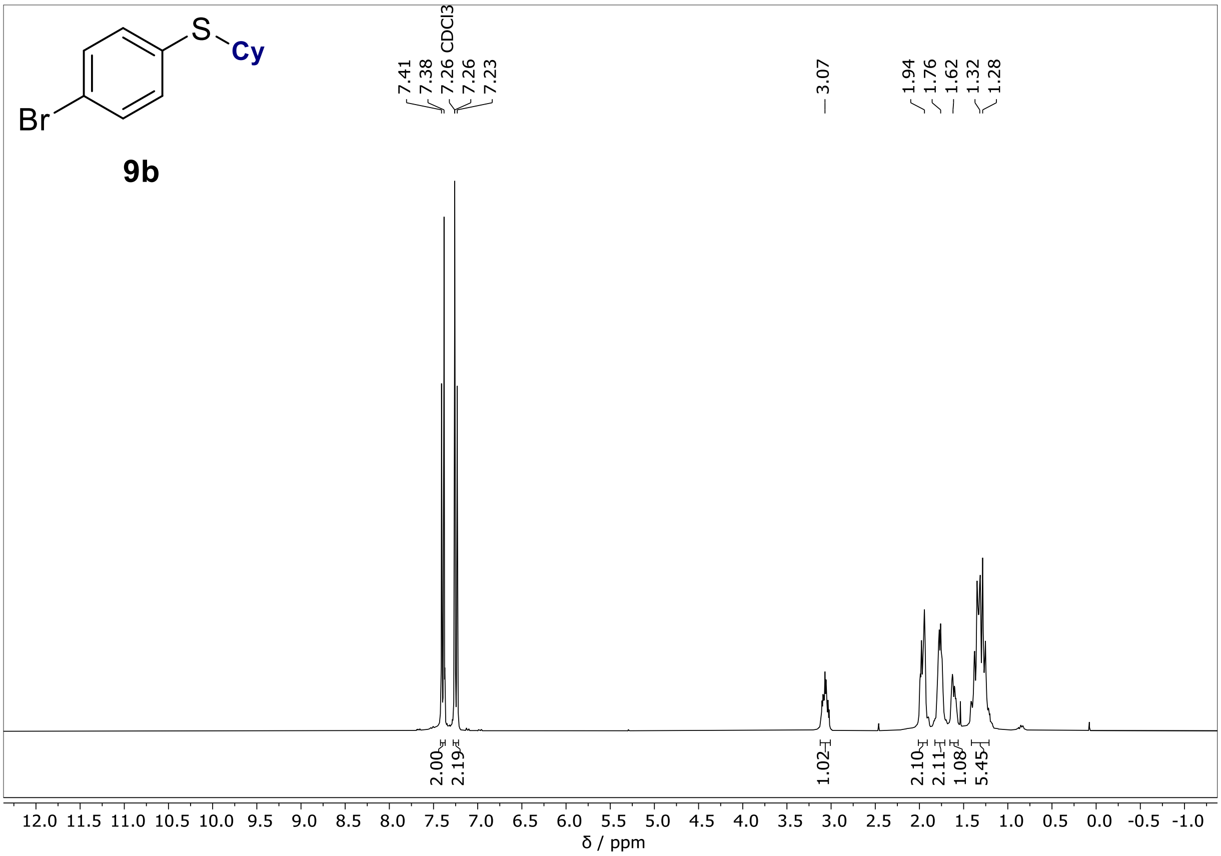

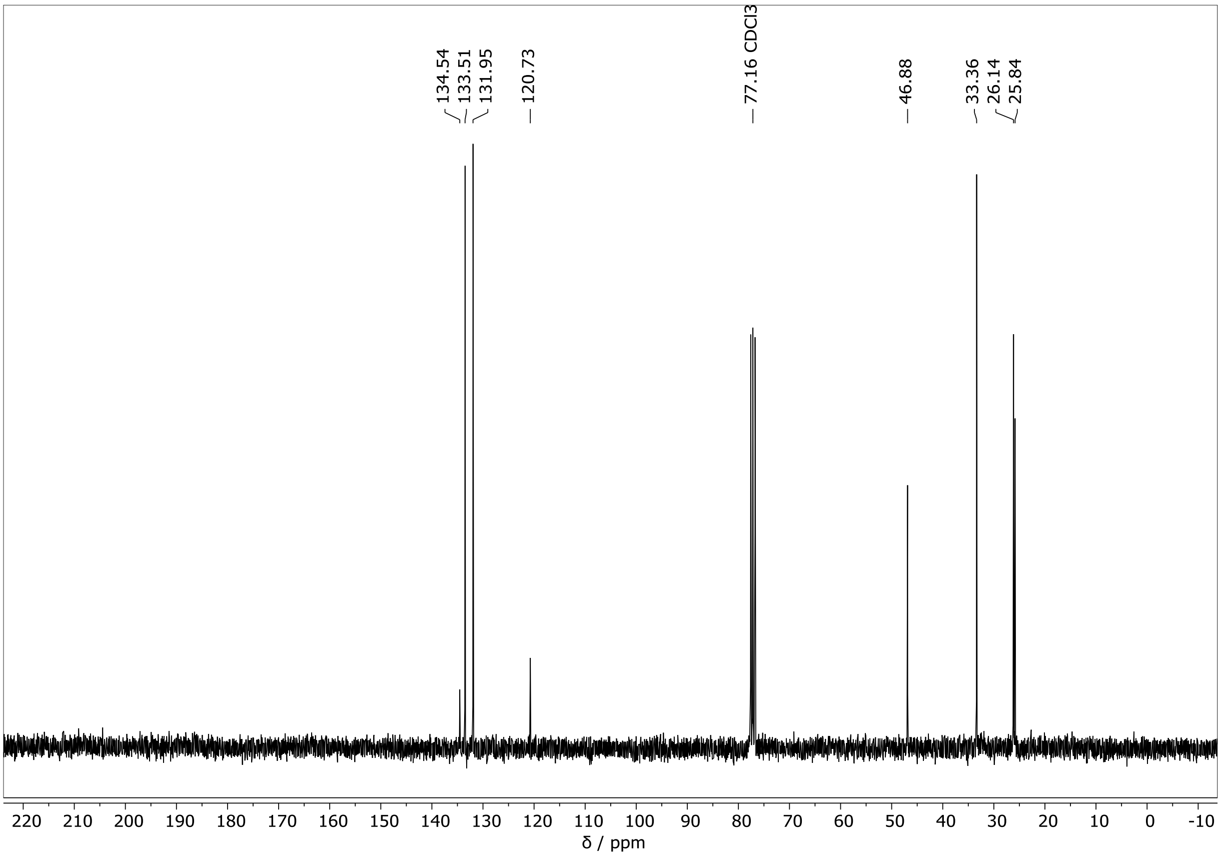

(4-Chlorophenyl)(cyclohexyl)sulfane (9c)

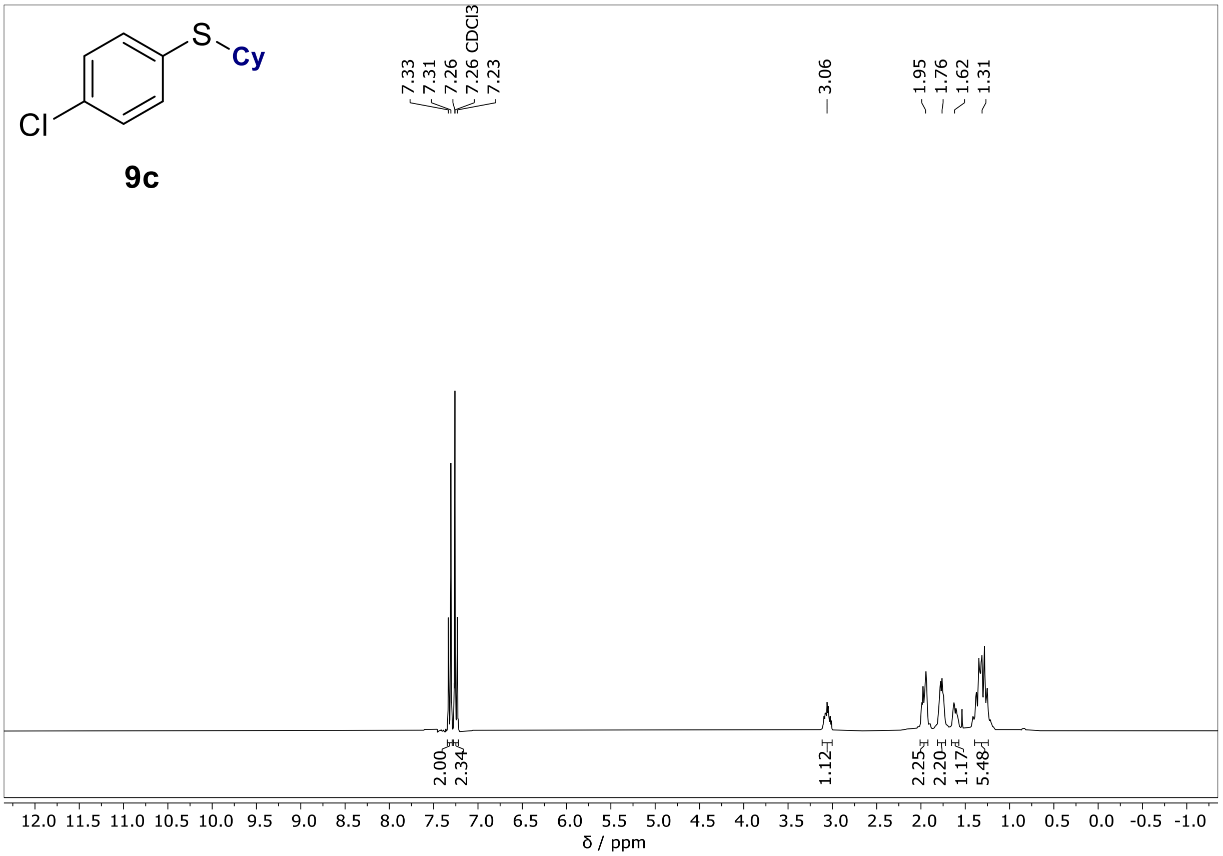

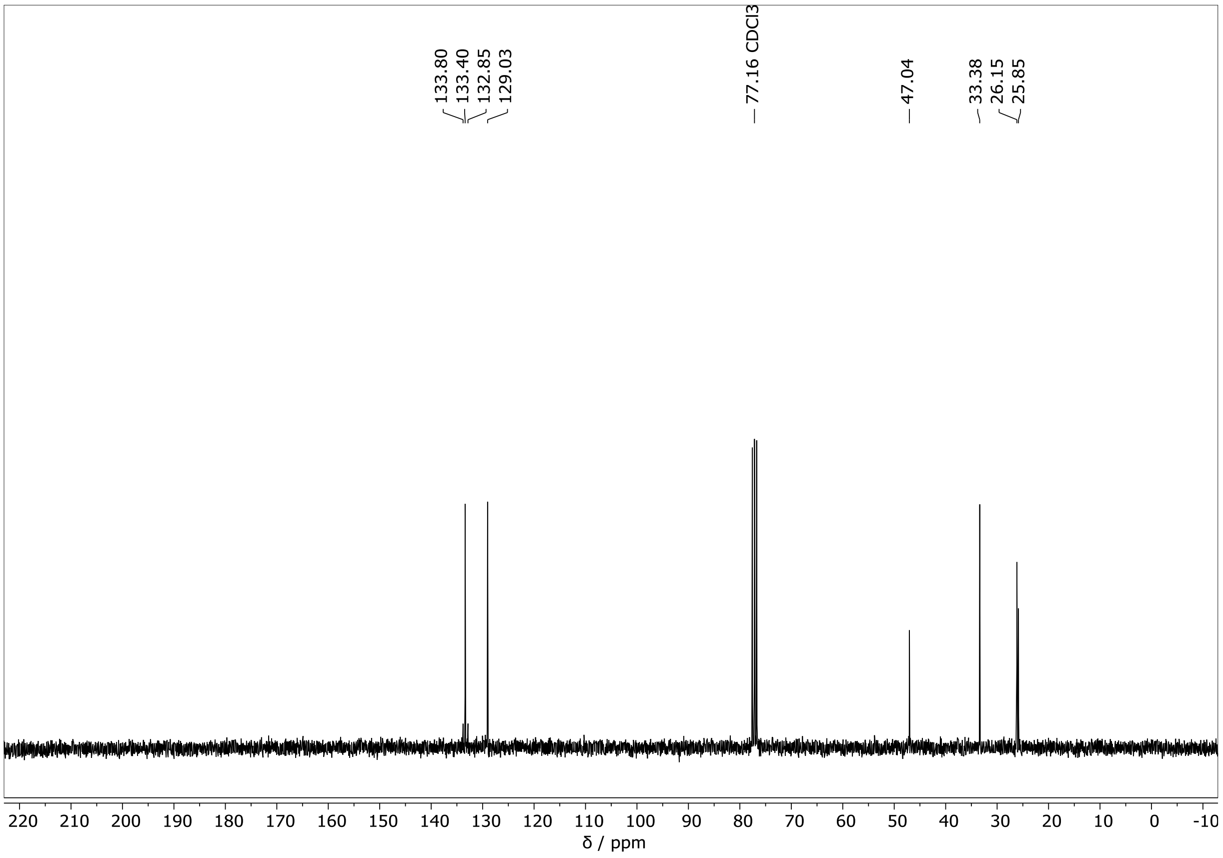

(1-(4-Isopropylphenyl)propan-2-yl)(phenyl)sulfane (9d)

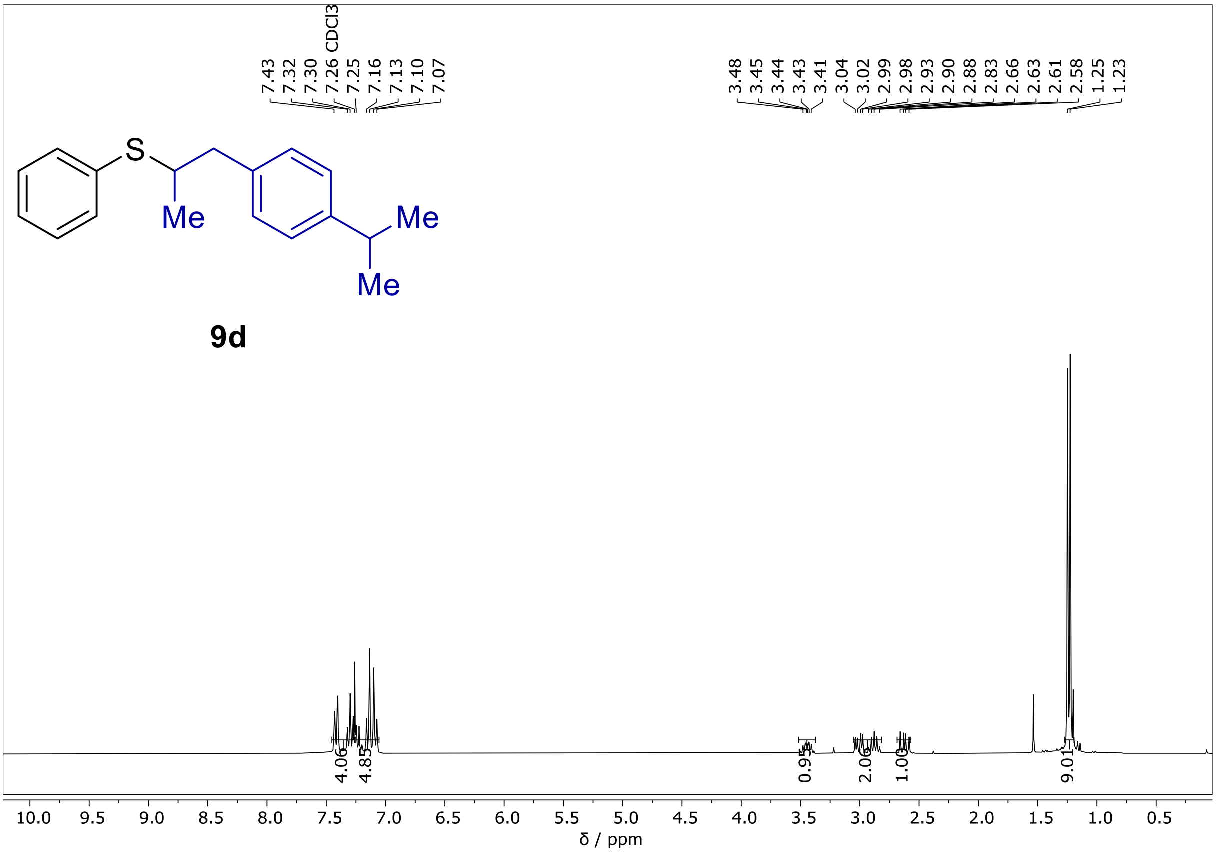

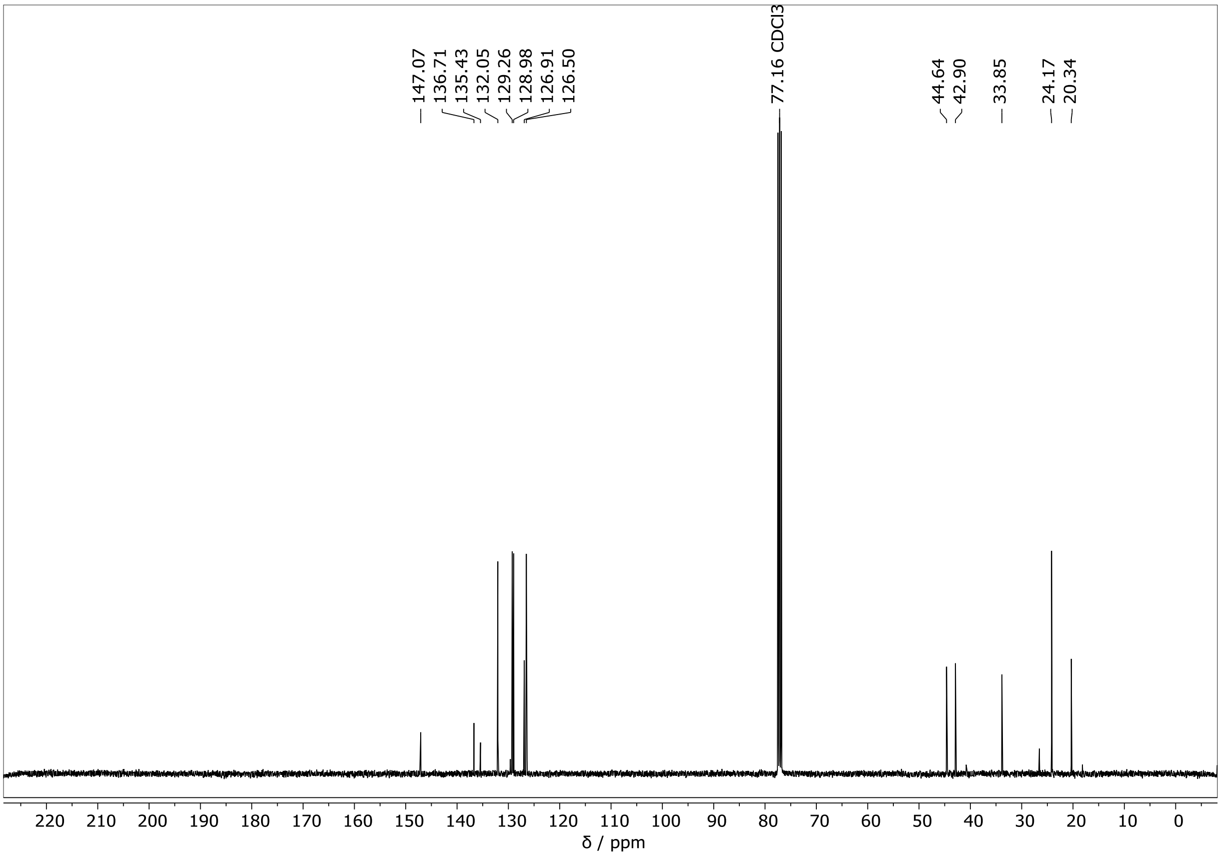

(6-Methylhept-5-en-2-yl)(phenyl)sulfane (9e)

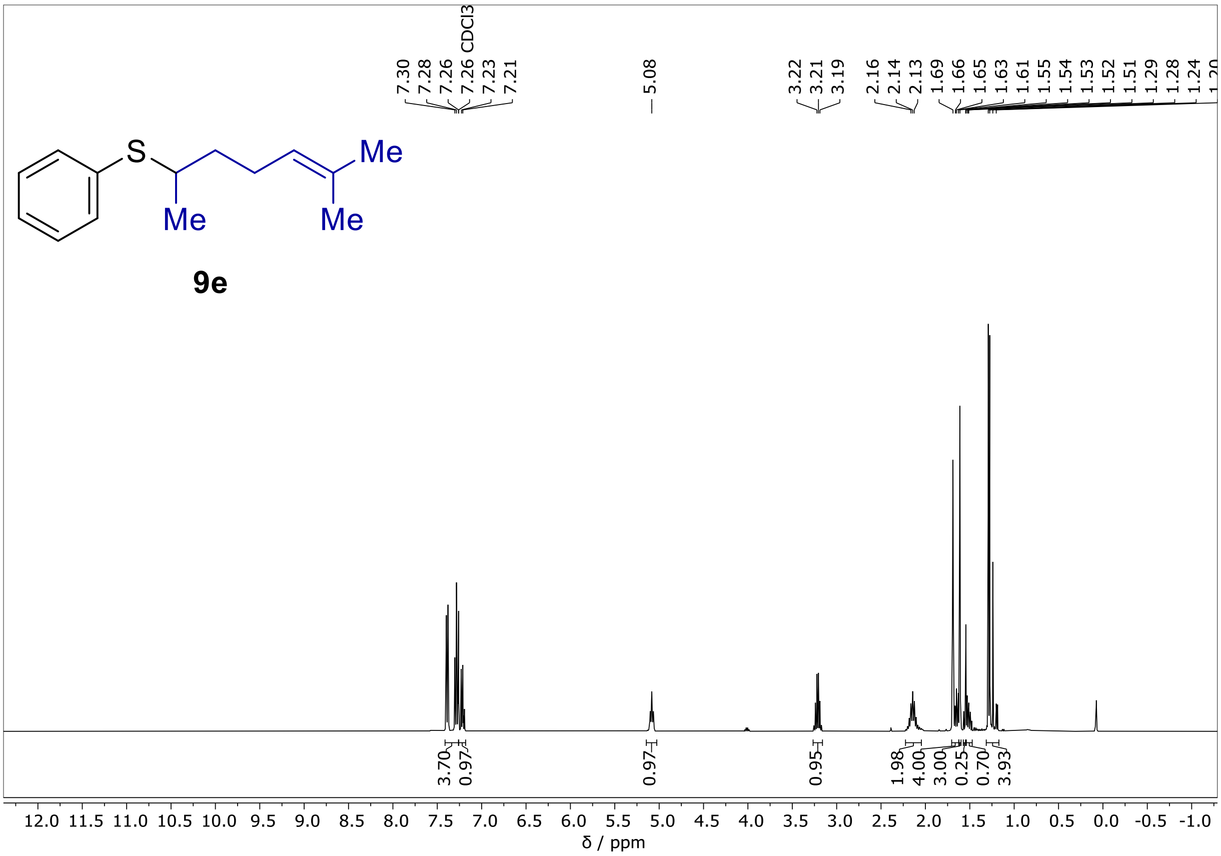

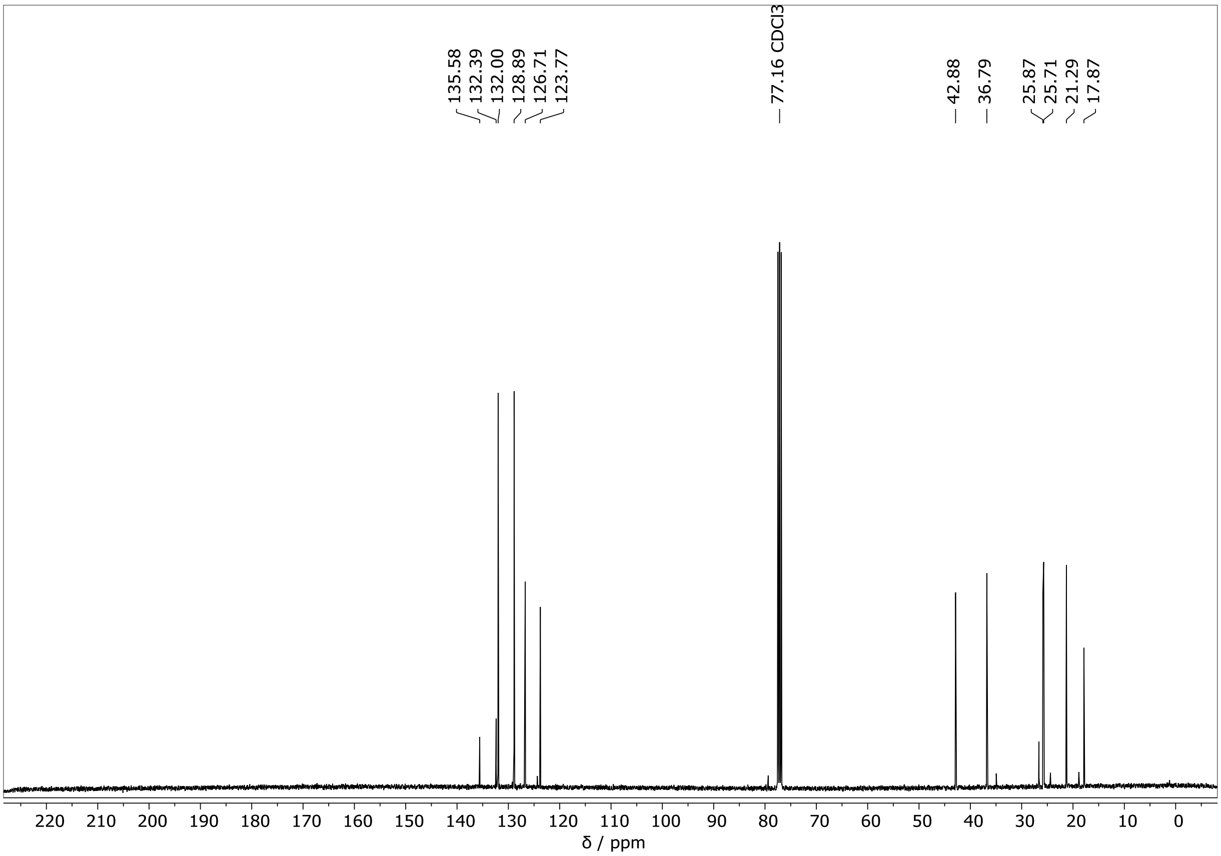

2,2-Dimethyl-4-(phenylthio)-1,3-dioxolane (9f)

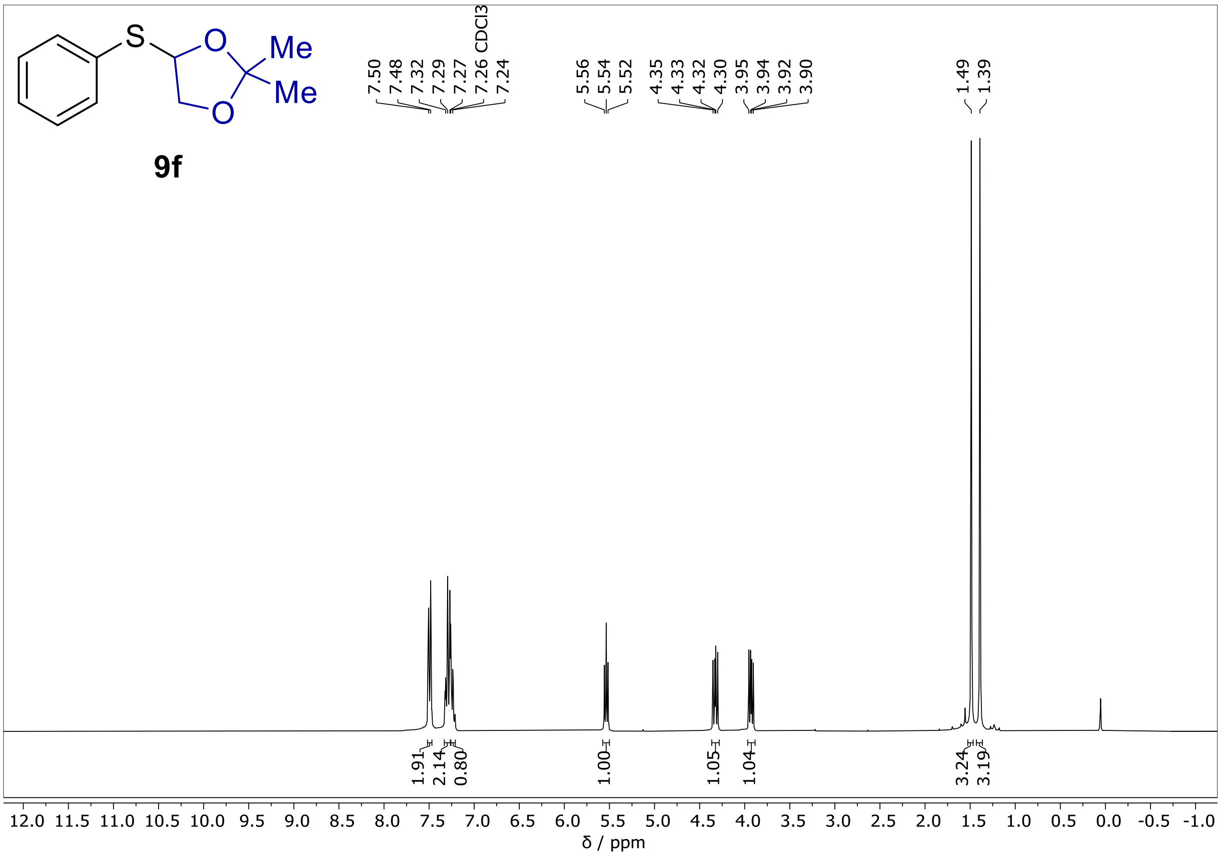

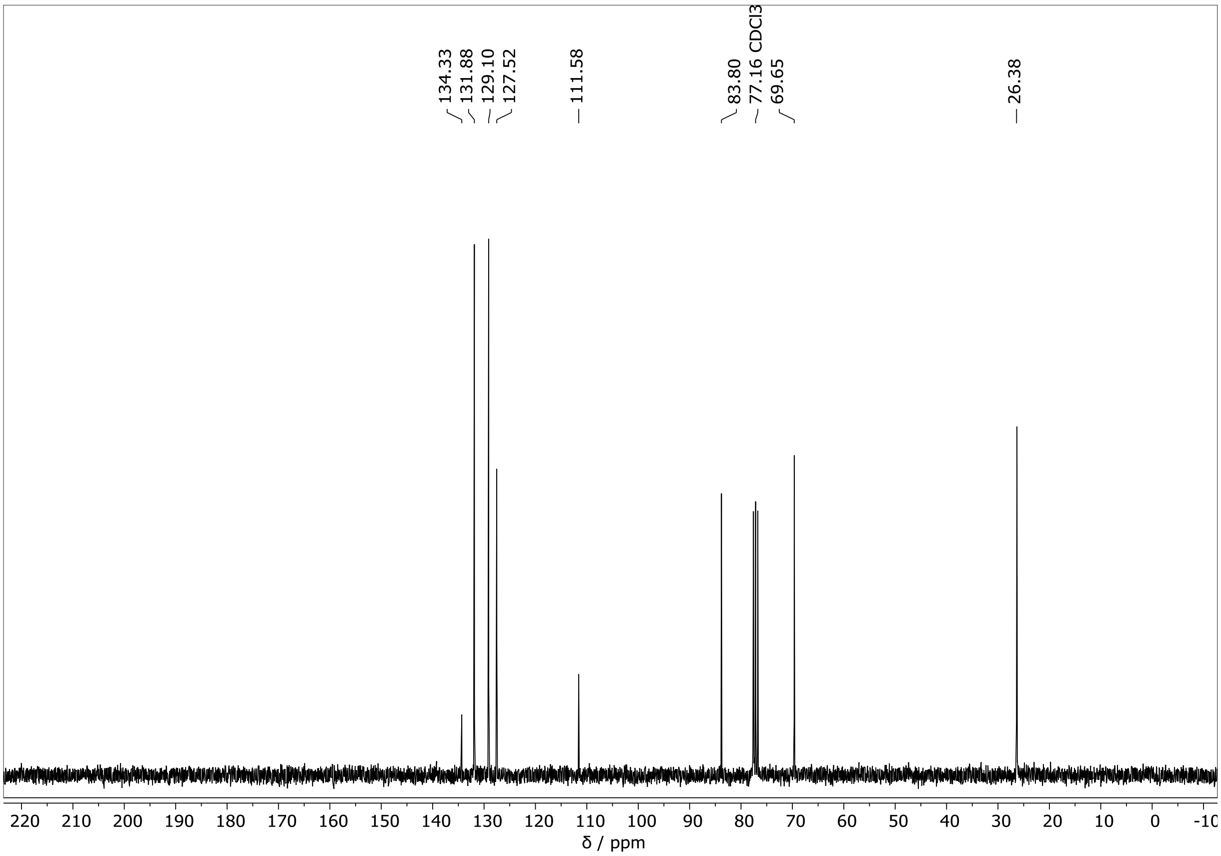

(4-Bromophenyl)(butyl)sulfane (9g)

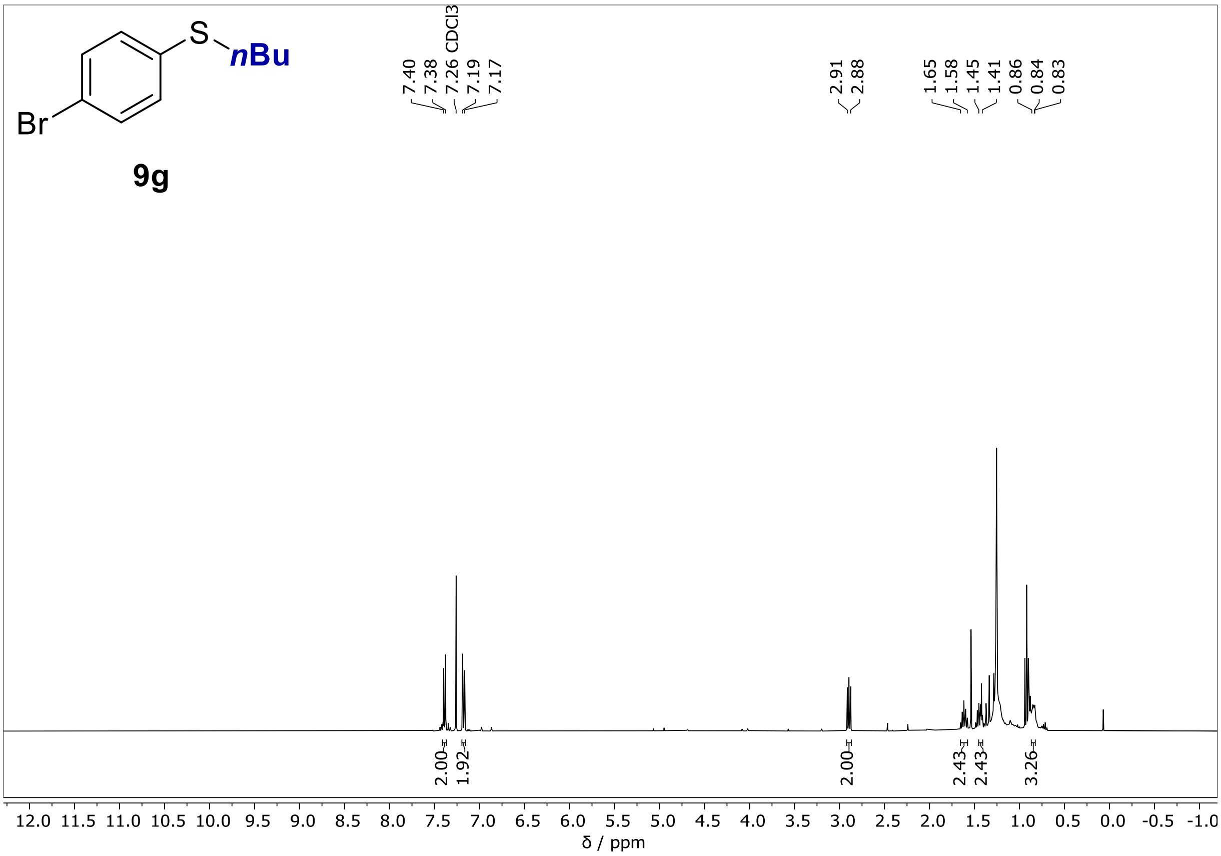

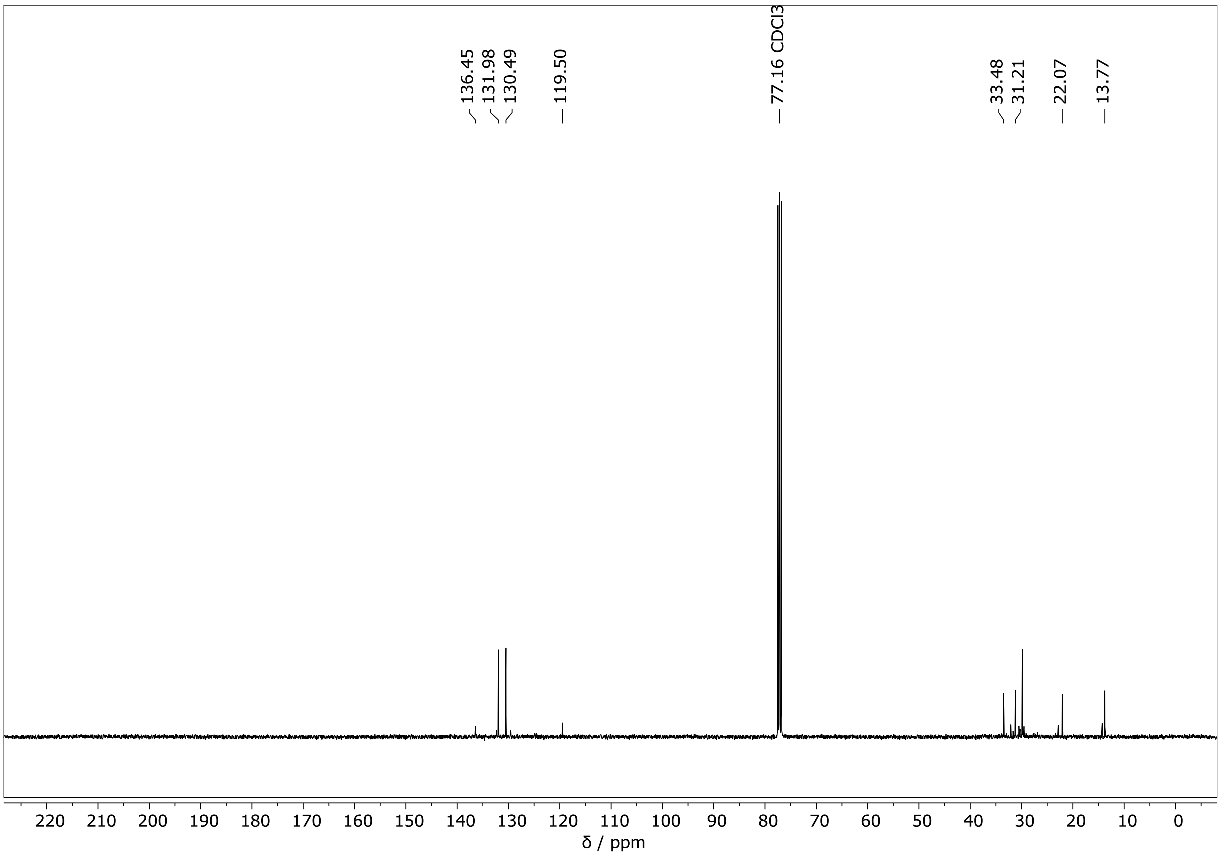

Cyclohexyl(4-(trifluoromethyl)phenyl)sulfane (9h)

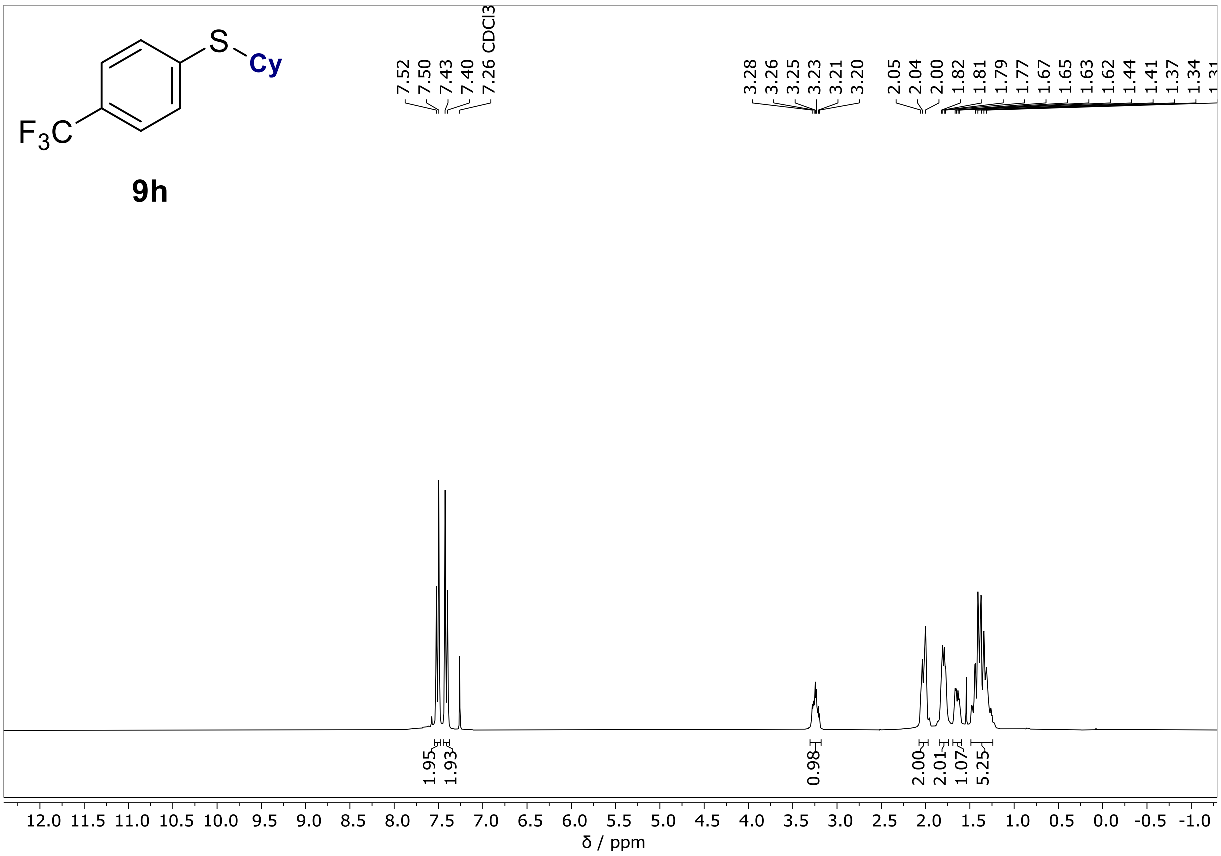

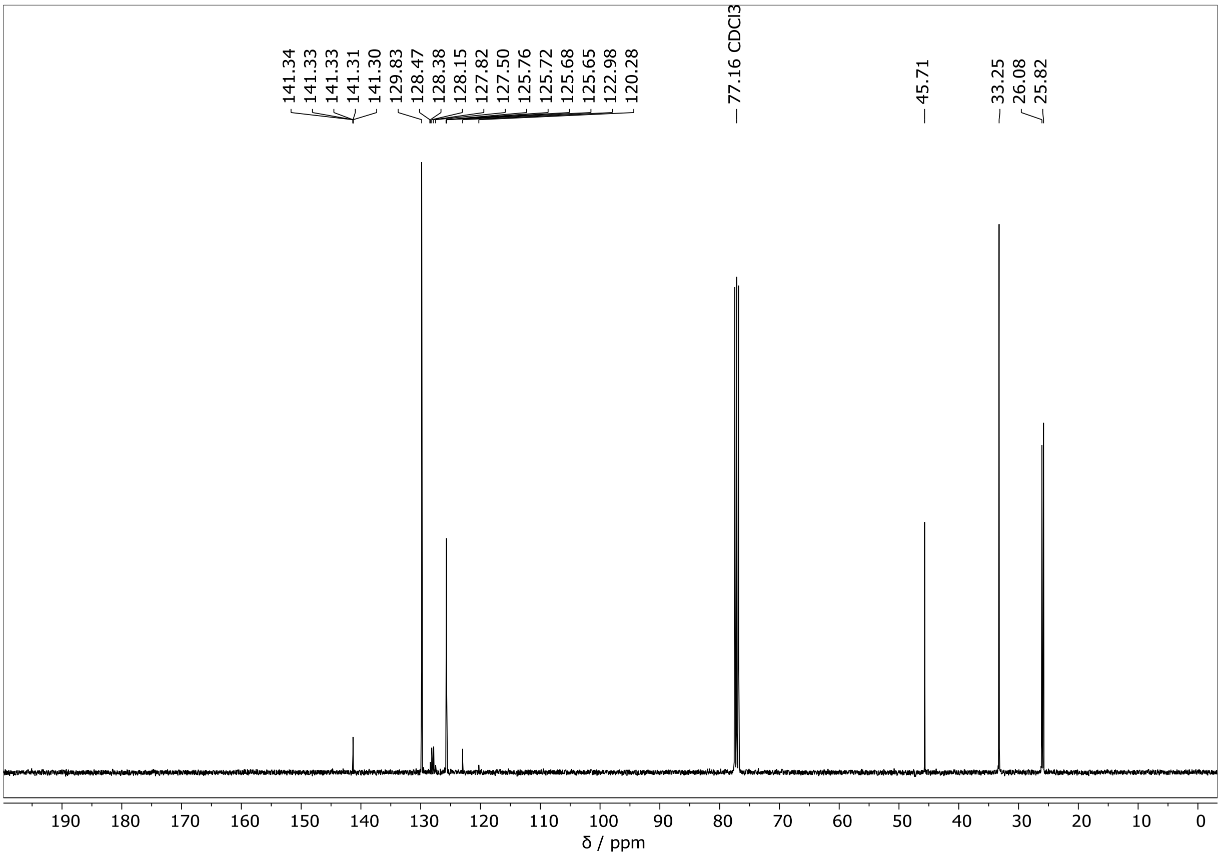

3-(Phenylthio)tetrahydrofuran (9i)

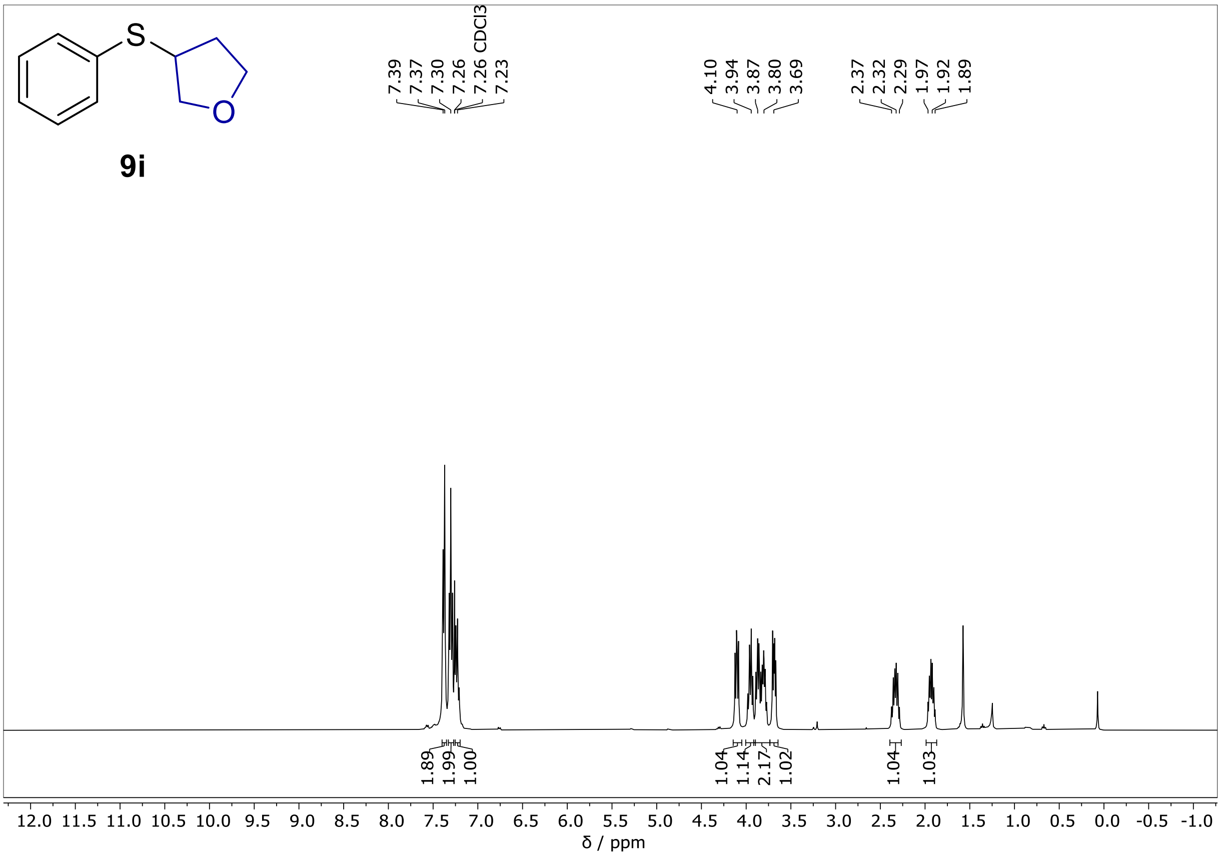

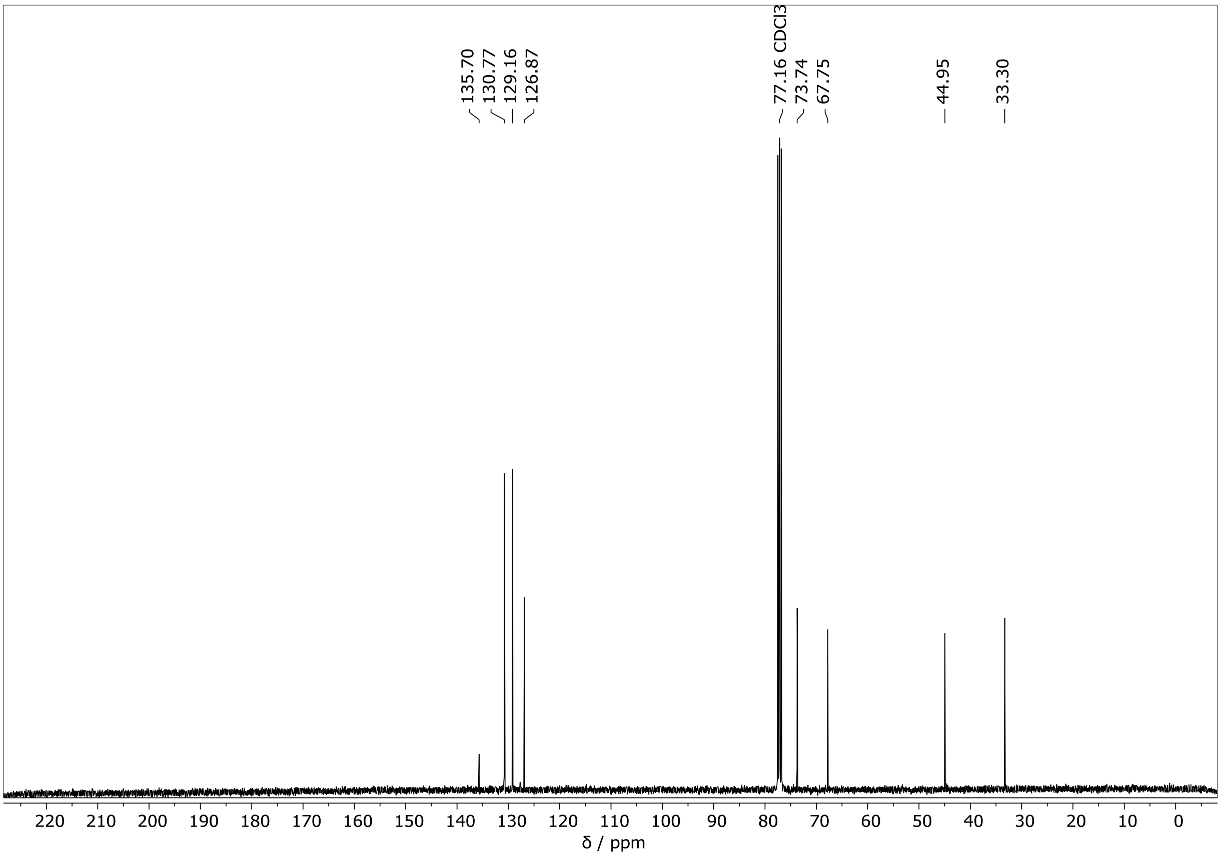

Isopropyl(4-(trifluoromethyl)phenyl)sulfane (9j)

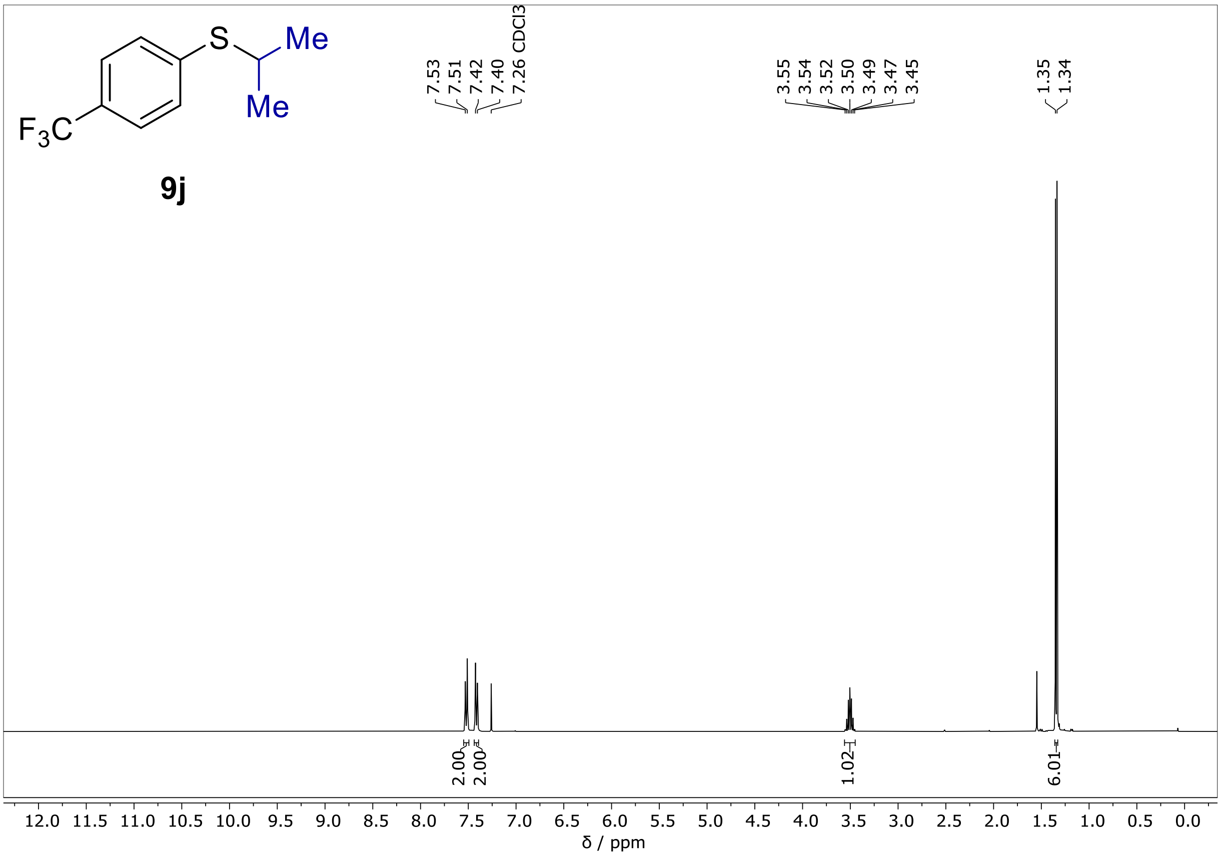

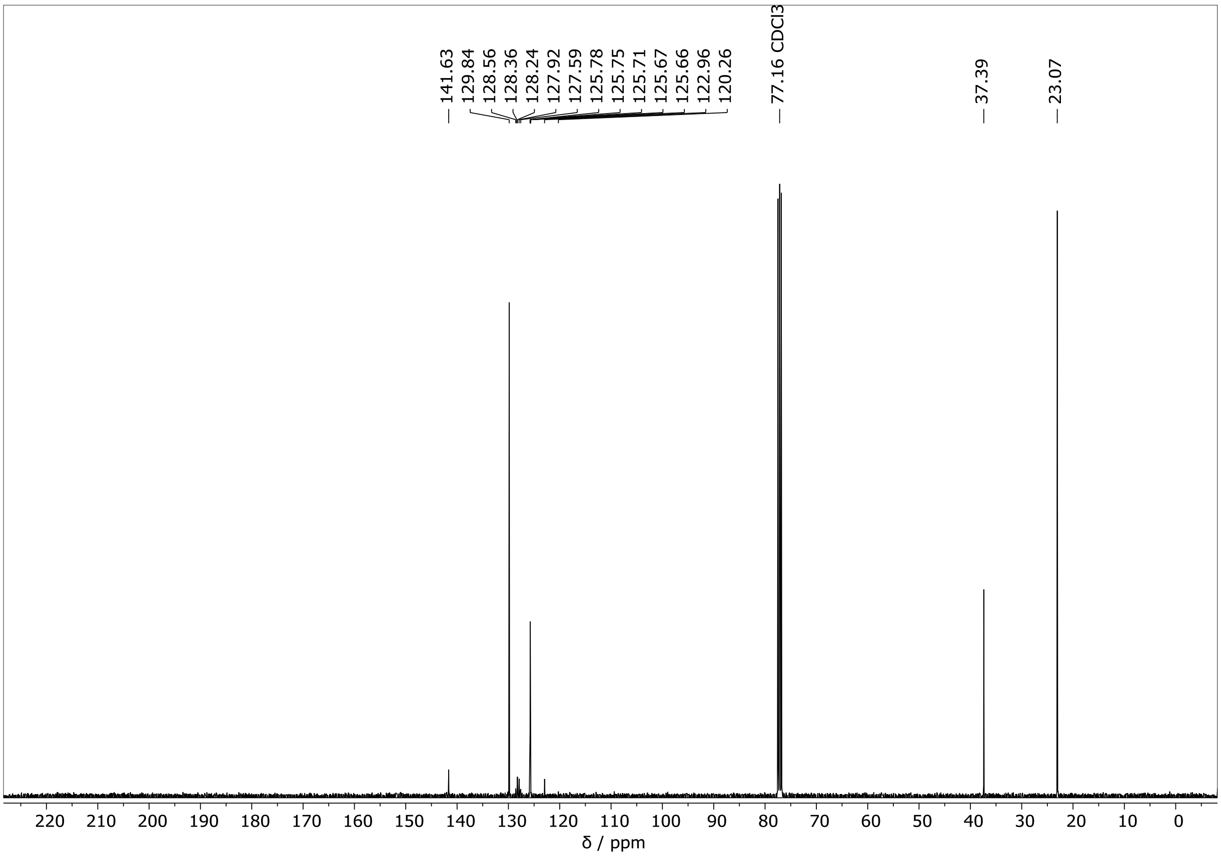

Cyclohexyl(*m*-tolyl)sulfane (9k)

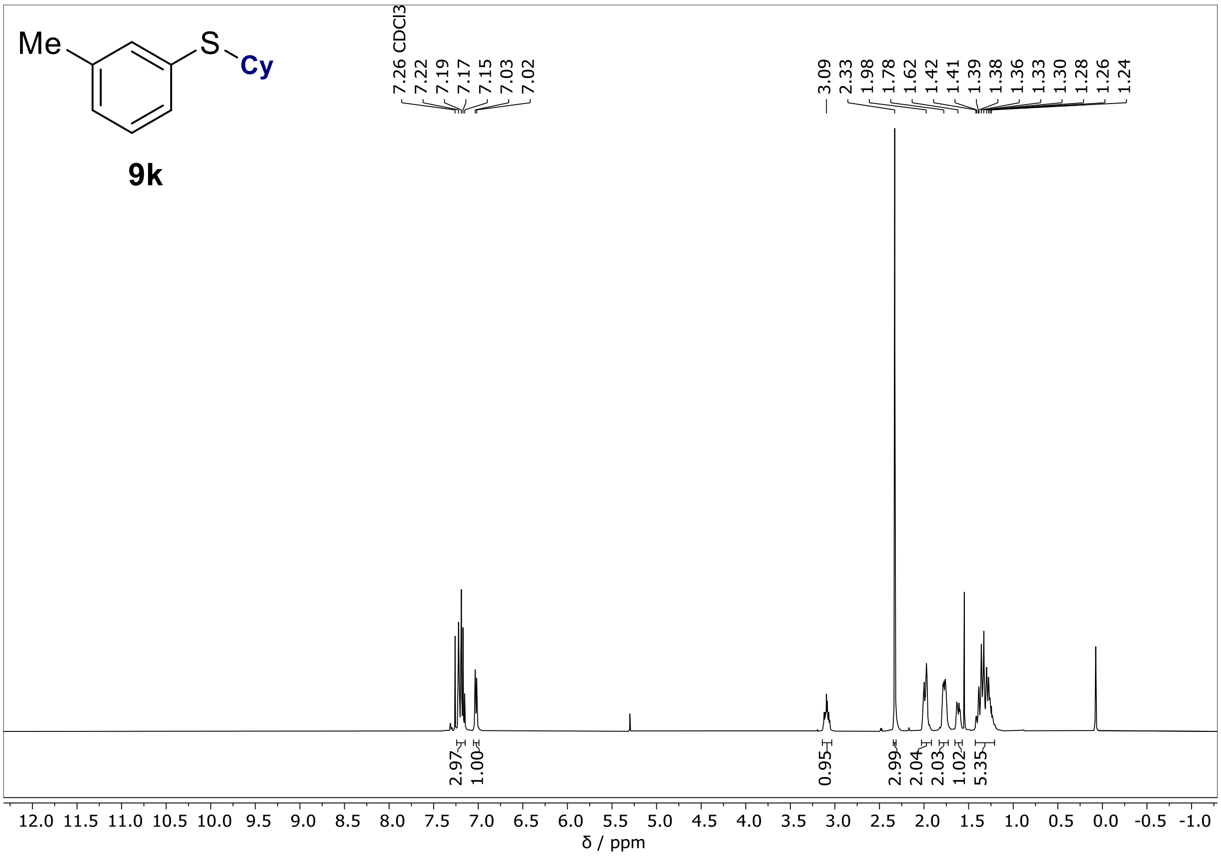

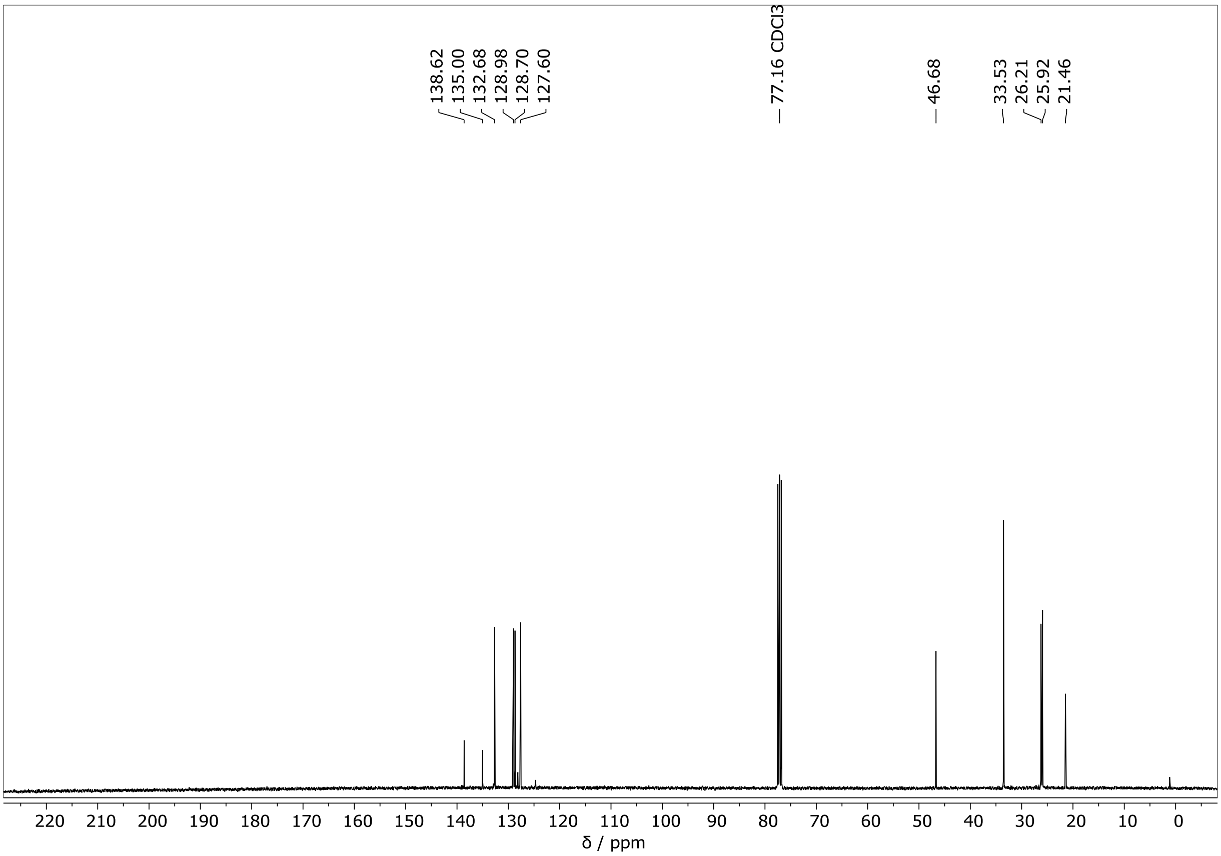

1-(But-3-en-1-yl)-4-methoxybenzene (10)

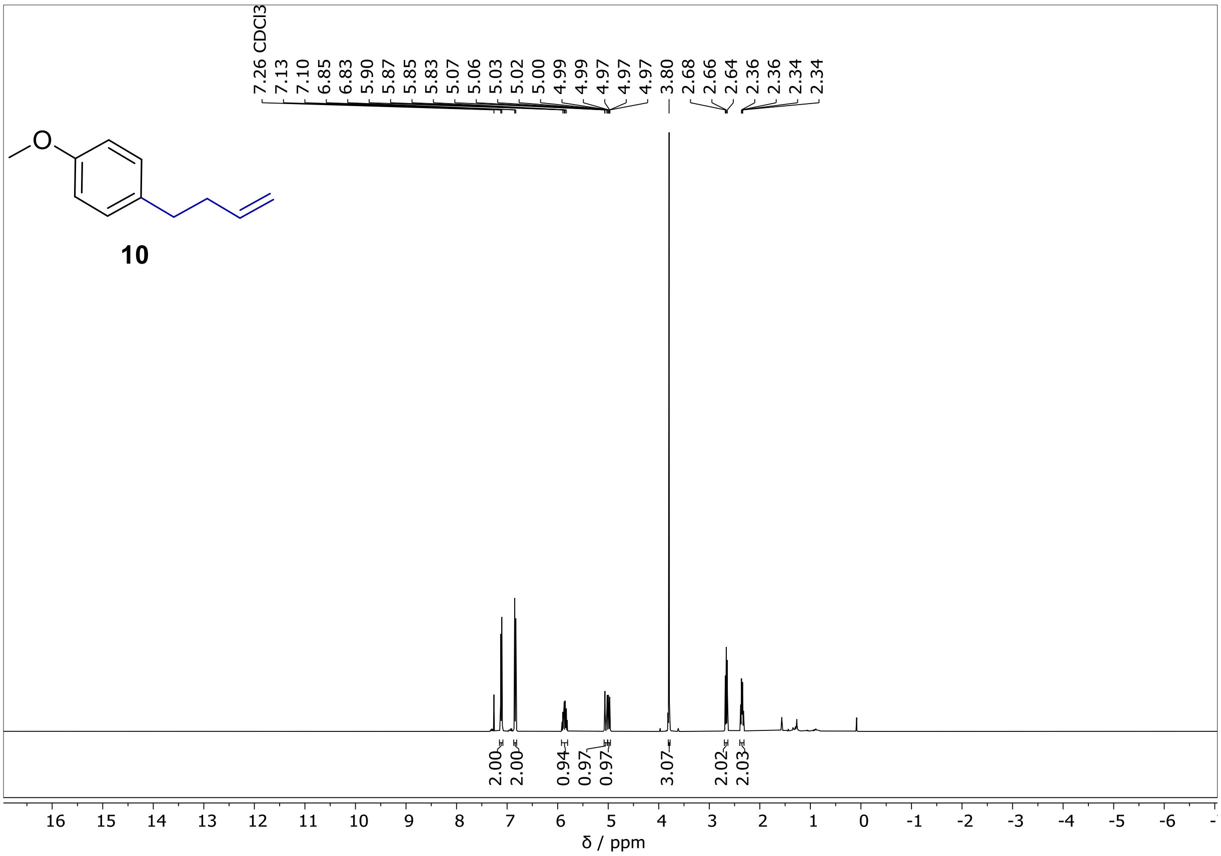

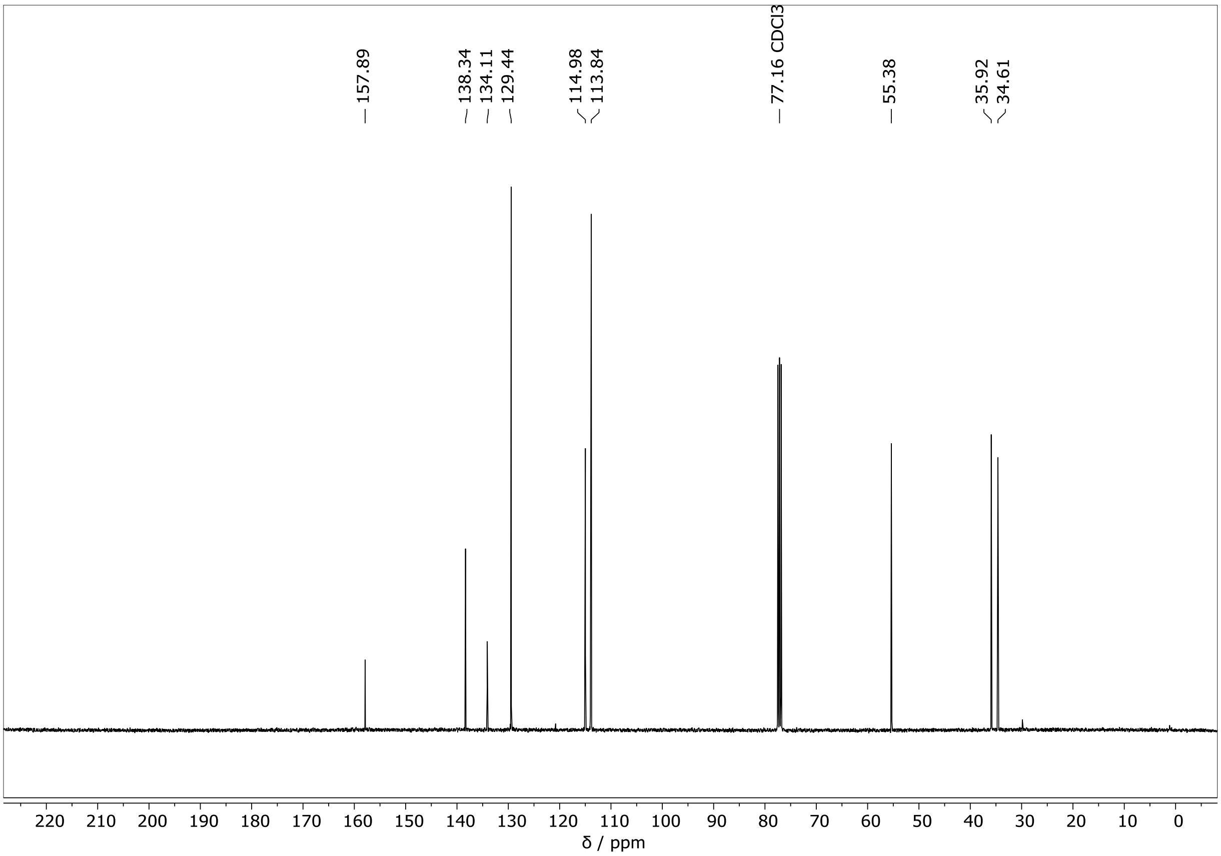

Ethyl 2-(dimethylamino)-4-methylpyrimidine-5-carboxylate

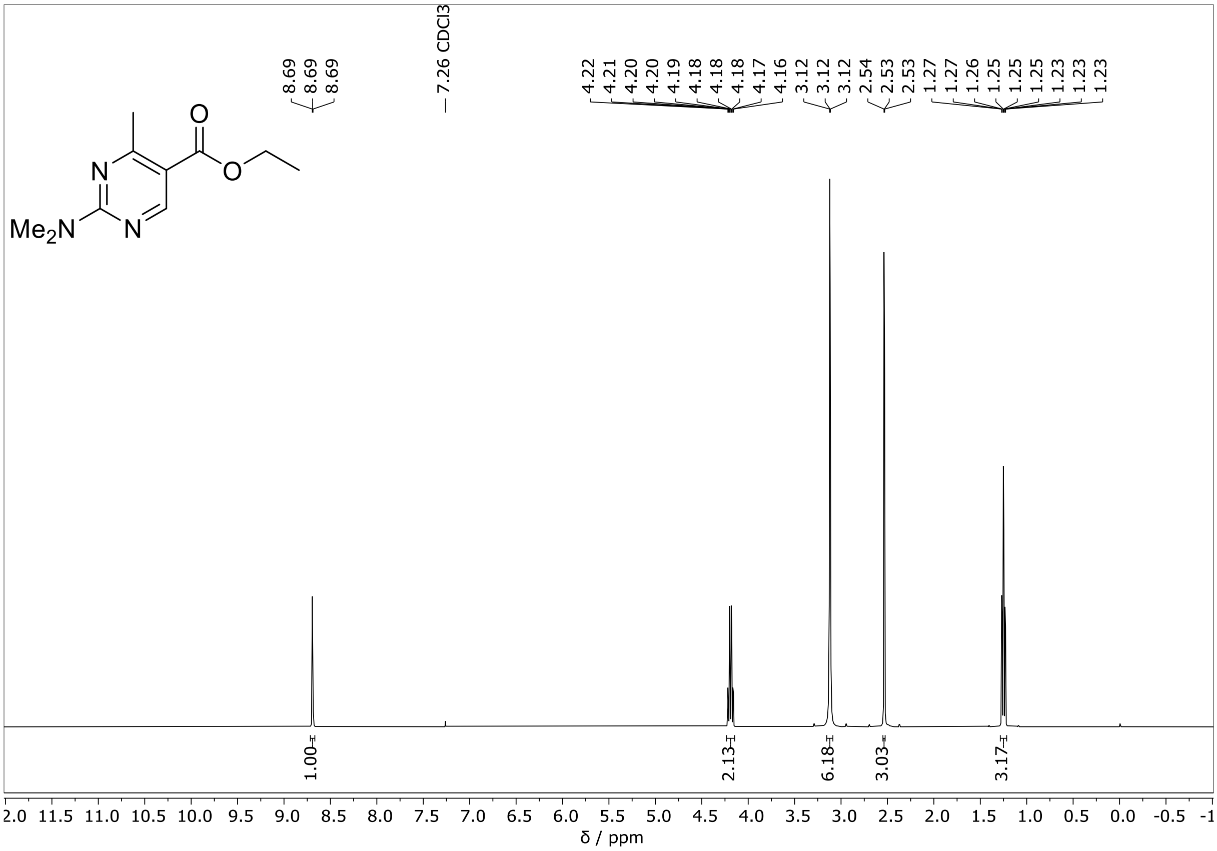

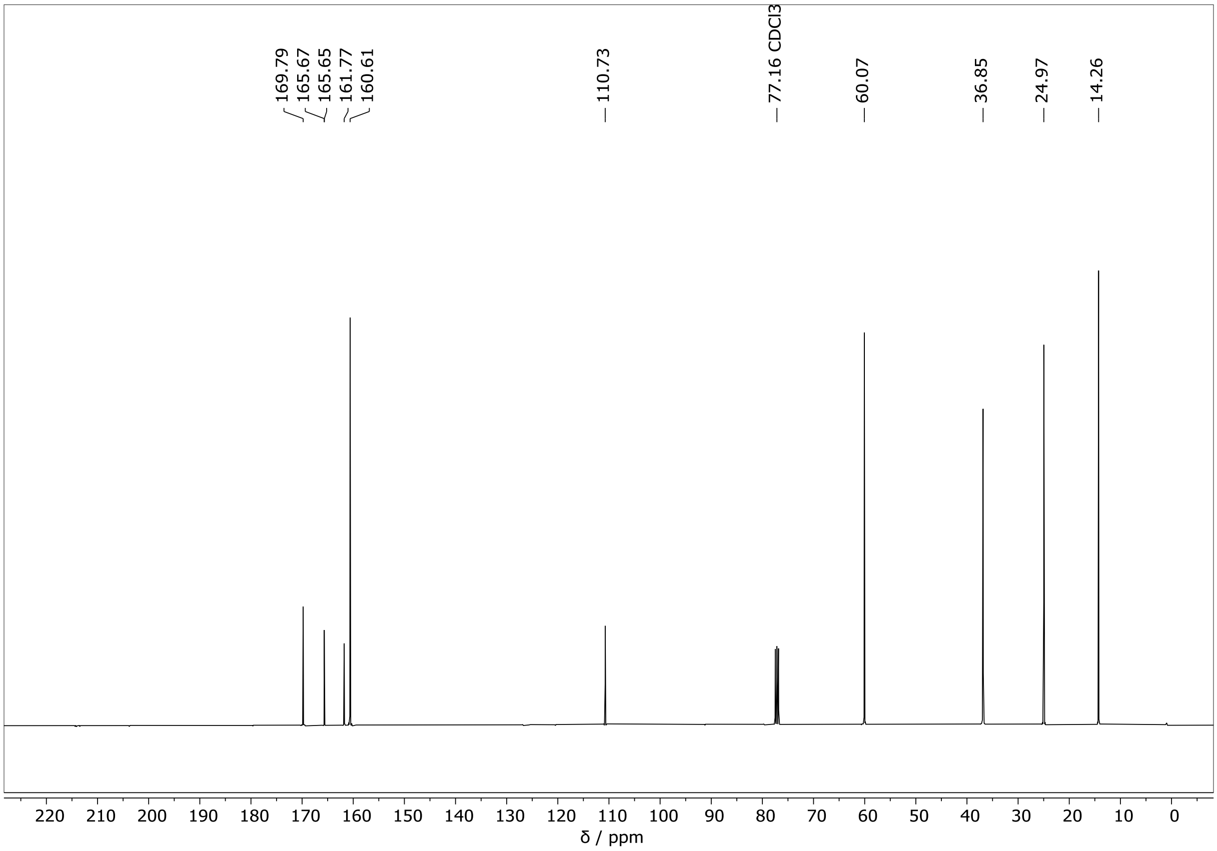

References

[1] Y. Sun, N. Zhang, J. Ren, H. Huang, X. Luan, Z. Zuo, *Org. Lett.* **2024**, *26*, 35.

[2] M. N. Khrizanforov, S. V. Fedorenko, S. O. Strekalova, K. V. Kholin, A. R. Mustafina, M. Y. Zhilkin, V. V. Khrizanforova, Y. N. Osin, V. V. Salnikov, T. V. Gryaznova et al., *Dalton Trans.* **2016**, *45*, 11976.

[3] C. Oliver Kappe, J. Jacques Vanden Eynde, N. Audiart, V. Canonne, S. Michel, Y. van Haverbeke, *HETEROCYCLES* **1997**, *45*, 1967.

[4] Sár, C. P., Hankovszky, O. H., Jerkovich, G., Pallagi, I., Hideg, K., *ACH - Models Chem.* **1994**, *131*, 363.

[5] T. van Leeuwen, L. Buzzetti, L. A. Perego, P. Melchiorre, *Angew. Chem. Int. Ed.* **2019**, *58*, 4953.

[6] X.-Z. Fan, J.-W. Rong, H.-L. Wu, Q. Zhou, H.-P. Deng, J. Da Tan, C.-W. Xue, L.-Z. Wu, H.-R. Tao, J. Wu, *Angew. Chem. Int. Ed.* **2018**, *57*, 8514.

[7] P. Sharma, T. Singh, N. Rawat, A. Singh, *J. Org. Chem.* **2025**, *90*, 5574.

[8] T. Chinzei, K. Miyazawa, Y. Yasu, T. Koike, M. Akita, *RSC Adv.* **2015**, *5*, 21297.

[9] L. Chu, C. Ohta, Z. Zuo, D. W. C. MacMillan, *J. Am. Chem. Soc.* **2014**, *136*, 10886.

[10] M. Ueda, E. Kondoh, Y. Ito, H. Shono, M. Kakiuchi, Y. Ichii, T. Kimura, T. Miyoshi, T. Naito, O. Miyata, *Org. Biomol. Chem.* **2011**, *9*, 2062.

[11] S. Rohe, A. O. Morris, T. McCallum, L. Barriault, *Angew. Chem. Int. Ed.* **2018**, *57*, 15664.

[12] L. Zou, S. Xiang, R. Sun, Q. Lu, *Nat. Commun.* **2023**, *14*, 7992.

[13] P. G. N. Neate, B. Zhang, J. Conforti, W. W. Brennessel, M. L. Neidig, *Org. Lett.* **2021**, *23*, 5958.

[14] X.-J. Wei, I. Abdiaj, C. Sambiagio, C. Li, E. Zysman-Colman, J. Alcázar, T. Noël, *Angew. Chem. Int. Ed.* **2019**, *58*, 13030.

[15] L. R. Mills, D. Gygi, J. R. Ludwig, E. M. Simmons, S. R. Wisniewski, J. Kim, P. J. Chirik, *ACS Catal.* **2022**, *12*, 1905.

[16] V. R. Bhonde, B. T. O'Neill, S. L. Buchwald, *Angew. Chem. Int. Ed.* **2016**, *55*, 1849.

[17] X. Tian, J. Kaur, S. Yakubov, J. P. Barham, *ChemSusChem* **2022**, *15*, e202200906.

[18] Y. Gong, L. Su, Z. Zhu, Y. Ye, H. Gong, *Angew. Chem. Int. Ed.* **2022**, *61*, e202201662.

[19] W. Du, F. Zhao, R. Yang, Z. Xia, *Org. Lett.* **2024**, *26*, 3145.

[20] X. Zhang, C. Yang, *Adv. Synth. Catal.* **2015**, *357*, 2721.

[21] K. A. Xie, E. Bednarova, C. L. Joe, C. Lin, T. C. Sherwood, E. M. Simmons, B. C. Lainhart, T. Rovis, *J. Am. Chem. Soc.* **2023**, *145*, 19925.

[22] J. S. Fortin, M.-F. Côté, J. Lacroix, M. Desjardins, E. Petitclerc, R. C-Gaudreault, *Bioorg. Med. Chem.* **2008**, *16*, 7277.

[23] A. A. Leushukou, A. V. Krech, A. L. Hurski, *Org. Lett.* **2022**, *24*, 6277.

[24] H.-M. Jiang, J.-H. Qin, Q. Sun, D. Zhang, J.-P. Jiang, X.-H. Ouyang, R.-J. Song, J.-H. Li, *Org. Chem. Front.* **2022**, *9*, 4070.
[25] D.-Z. Zheng, H.-G. Xiong, A.-X. Song, H.-G. Yao, C. Xu, *Org. Biomol. Chem.* **2022**, *20*, 2096.
[26] M. Bollenbach, P. Wagner, P. G. V. Aquino, J.-J. Bourguignon, F. Bihel, C. Salomé, M. Schmitt, *ChemSusChem* **2016**, *9*, 3244.
[27] M. Nakanishi, D. Katayev, C. Besnard, E. P. Kündig, *Angew. Chem. Int. Ed.* **2011**, *50*, 7438.
[28] K. Kiyokawa, K. Kawanaka, S. Minakata, *Angew. Chem. Int. Ed.* **2024**, *63*, e202319048.
[29] R. M. Oechsner, I. H. Lindenmaier, I. Fleischer, *Org. Lett.* **2023**, *25*, 1655.
[30] B. Du, B. Jin, P. Sun, *Org. Lett.* **2014**, *16*, 3032.
[31] T. Yamada, Y. Hashimoto, K. Tanaka, N. Morita, O. Tamura, *J. Org. Chem.* **2020**, *85*, 12315.
[32] Y. Liu, Z. Zhang, Z. Wei, Z. Zhong, S. Liu, Y. Yang, X. Zeng, *New J. Chem.* **2023**, *47*, 7369.
[33] T. Uchikura, Y. Hara, K. Tsubono, T. Akiyama, *ACS Org. Inorg. Au* **2021**, *1*, 23.
[34] H. Wu, S. Chen, C. Liu, Q. Zhao, Z. Wang, Q. Jin, S. Sun, J. Guo, X. He, P. J. Walsh et al., *Angew. Chem. Int. Ed.* **2024**, *63*, e202314790.
[35] Y.-Y. Zhu, G. Lan, Y. Fan, S. S. Veroneau, Y. Song, D. Micheroni, W. Lin, *Angew. Chem. Int. Ed.* **2018**, *57*, 14090.
[36] A.-M. Hu, J.-L. Tu, M. Luo, C. Yang, L. Guo, W. Xia, *Org. Chem. Front.* **2023**, *10*, 4764.
[37] P. Boehm, P. Müller, P. Finkelstein, M. A. Rivero-Crespo, M.-O. Ebert, N. Trapp, B. Morandi, *J. Am. Chem. Soc.* **2022**, *144*, 13096.
[38] T. Delcaillau, A. Bismuto, Z. Lian, B. Morandi, *Angew. Chem. Int. Ed.* **2020**, *59*, 2110.
[39] N. Li, J.-L. Si, M.-Y. Xu, *Chem. Eur. J.* **2025**, *31*, e202404116.
[40] P. C. Gallage, M. G. McKee, S. P. Pitre, *Org. Lett.* **2024**, *26*, 1975.
